# Meta-analysis of gene expression studies in endometrial cancer identifies gene expression profiles associated with aggressive disease and patient outcome

**DOI:** 10.1038/srep36677

**Published:** 2016-11-10

**Authors:** Tracy A. O’Mara, Min Zhao, Amanda B. Spurdle

**Affiliations:** 1 Genetics and Computational Biology Department, QIMR Berghofer Medical Research Institute, Herston, QLD 4006, Australia; 2 School of Engineering, Faculty of Science, Health, Education and Engineering, University of the Sunshine Coast, Queensland, 4558, Australia

## Abstract

Although endometrioid endometrial cancer (EEC; comprising ~80% of all endometrial cancers diagnosed) is typically associated with favourable patient outcome, a significant portion (~20%) of women with this subtype will relapse. We hypothesised that gene expression predictors of the more aggressive non-endometrioid endometrial cancers (NEEC) could be used to predict EEC patients with poor prognosis. To explore this hypothesis, we performed meta-analysis of 12 gene expression microarray studies followed by validation using RNA-Seq data from The Cancer Genome Atlas (TCGA) and identified 1,253 genes differentially expressed between EEC and NEEC. Analysis found 121 genes were associated with poor outcome among EEC patients. Forward selection likelihood-based modelling identified a 9-gene signature associated with EEC outcome in our discovery RNA-Seq dataset which remained significant after adjustment for clinical covariates, but was not significant in a smaller RNA-Seq dataset. Our study demonstrates the value of employing meta-analysis to improve the power of gene expression microarray data, and highlight genes and molecular pathways of importance for endometrial cancer therapy.

Endometrial cancer is the most commonly diagnosed gynecological cancer in developed countries, accounting for approximately 7% of new cancer cases in women worldwide^1^. Unlike most other cancer in females, age-standardized rated are steadily increasing^2^. Endometrioid endometrial cancers (EECs) are the most commonly reported histological subtype of endometrial cancer (~80% of all new cases), are estrogen-related tumors, and generally associated with good prognosis. Conversely, non-endometrioid endometrial cancers (NEECs; commonly serous papillary or clear cell histology) are estrogen-independent, and tend to be high-grade, clinically aggressive tumors^3^. A subset of EEC patients (~20%) will suffer recurrent tumors, with a 5-year survival rate reduced from 75–80% to less than 10%^4^. Although a recent study has reported the utility of *POLE* mutation status for identifying women with good prognosis^5^, there is currently no accepted method to identify markers that predict EEC patients with poor clinical outcome. Markers to predict EEC patients with poor prognosis will identify those women requiring more extensive surgery and adjuvant therapy to improve patient outcome. Such biomarkers may be discovered by comparing “global” molecular data for poor and good outcome EEC patients, but unfortunately few public datasets have been annotated for this phenotype. We hypothesized that a comparison of all EEC patients with poor-outcome NEEC patients might provide an alternative, better powered, strategy to identify biomarkers of EEC patients with poor outcome.

Global gene expression analysis is recognized as an effective strategy for determining profiles that could be used to classify cancer tissues into clinically meaningful subgroups. For example, the classification of breast cancers into luminal A, luminal B, normal, HER2 and basal-like subtypes, and the discovery of two distinct types of B-cell lymphoma (germinal center B-cell like lymphoma and activated B-cell like lymphoma) resulted from gene expression microarray studies^6^,^7^. It is recognized that results reported from individual microarray studies often display variability^8^. Indeed, variability can be observed for results from endometrial cancer microarray studies. For example, in total ~1,300 genes have been reported as differentially expressed across microarray studies assessing gene expression profiles between EEC and NEEC tumors^9^,^10^,^11^,^12^,^13^,^14^,^15^,^16^, however only 160 genes were reported in more than one study and no gene was reported by more than four studies.

To overcome the discrepancy and low reproducibility of individual microarray studies of endometrial cancer, we have performed a meta-analysis of 12 microarray gene expression studies to assess genes differentially expressed between NEEC and EEC cancers, as a means to identify genes that are important for development of aggressive endometrial cancer subtypes. The differential expression of these genes was validated using an independent endometrial cancer set with RNA-Seq data from The Cancer Genome Atlas (TCGA). We then explored the hypothesis that the aggressive gene signature identified by expression profiles associated with NEEC tumors can be used to predict EEC patients with poor prognosis and used validated aggressive signature genes to construct survival prediction models for EEC patients in the TCGA cohort. Our study demonstrates the value of employing meta-analysis for gene expression microarray data, and has highlighted genes and molecular pathways of importance for endometrial cancer prognosis and therapy.

## Results

**Figure 1:**
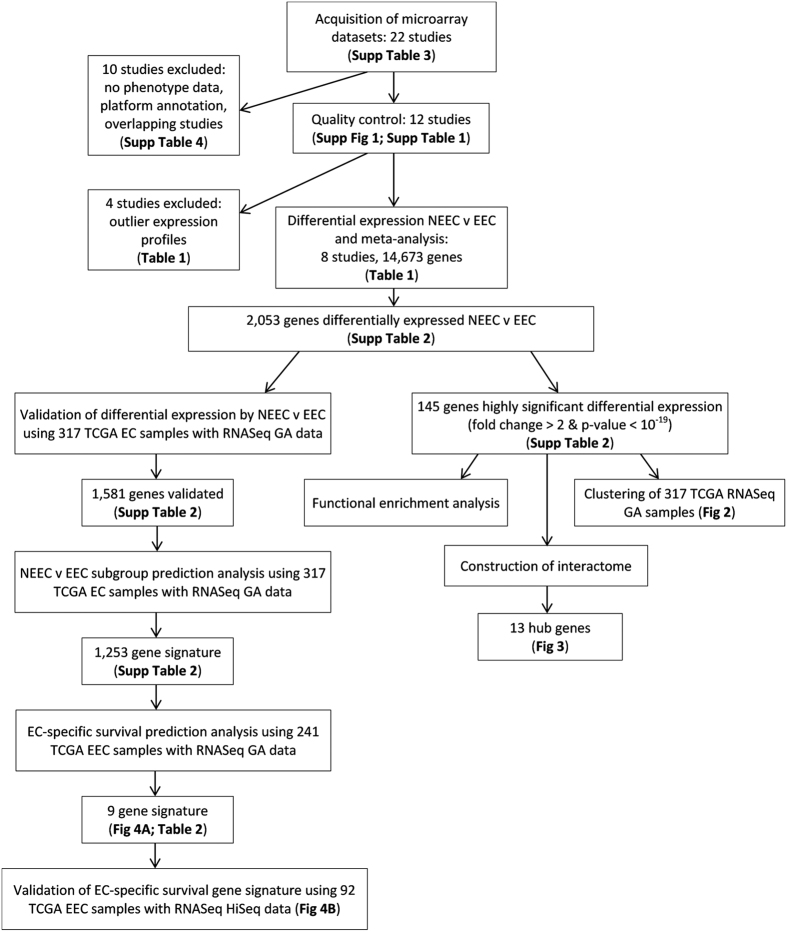
Study Overview. EEC - endometrioid endometrial cancer; NEEC - non-endometrioid endometrial cancer; TCGA - The Cancer Genome Atlas; EC-specific survival - endometrial cancer-specific survival.

An overview of the study design can be found in Fig. 1.

### Microarray Studies and Meta-Analysis

Following a literature review and repository search, twelve endometrial cancer microarray studies (Table 1) were merged and probes for 3,176 genes were extracted as being common across at least 10 studies. Principal components analysis using co-expression profiling and reproducibility estimates identified three studies as outliers (Supplementary Figure 1 and Supplementary Table 1). After considering sample size and number of probes assessed by each platform, an additional study (study 10) was removed from further analysis. The remaining eight studies were remerged, increasing the number of probes to 14,673 genes common across all studies. Genes displaying differential expression between NEEC and EEC tissue were identified for each study. Meta-analysis of individual study results found 2,053 genes (1126 upregulated, 927 downregulated) to be significantly differentially expressed between EEC and NEEC (Adjusted P-value < 0.05; Supplementary Table 2), and a consistent direction of effect observed across all eight studies.

### TCGA RNA-Seq Validation

**Figure 2:**
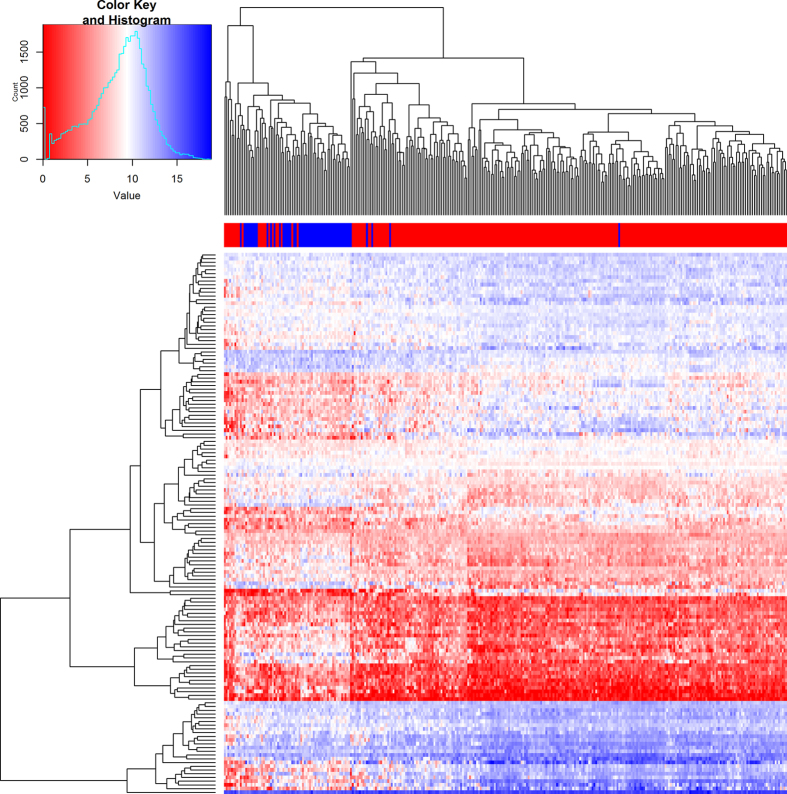
Gene expression patterns in endometrial cancer patients using RNA-Seq data from The Cancer Genome Atlas. Unsupervised hierarchical clustering and heatmap showing individual expression pattern in 145 most significantly differentially expressed genes identified by microarray meta-analysis. Patient subgroup (NEEC - blue, EEC - red) is depicted by the bar across the top of the heat map. Normalized expression value is displayed by the heatmap, where blue represents upregulated genes and red represents downregulated genes. EEC - endometrioid endometrial cancer; NEEC - non-endometrioid endometrial cancer.

Analysis of differential expression between NEEC and EEC tissue in 317 independent samples from the TCGA Illumina GA RNA-Seq dataset validated the result for 1,581 genes from the 2,053 genes (77%) identified by microarray meta-analysis (Adjusted P-value < 0.05 and same direction of effect; Supplementary Table 2). Class prediction analysis predicted 1,253 from the 1,581 genes would be able to distinguish the subtype (EEC or NEEC) of new tumors tested using compound covariate predictor and leave-one-out cross-validation. Pathway analysis found these 1,253 genes to be enriched in pathways for cell cycle (Adjusted P-value = 5.4 × 10^−7^), mitotic cell cycle (Adjusted P-value = 9.34 × 10^−7^), progesterone-mediated oocyte maturation (Adjusted P-value = 7.9 × 10^−5^) and oocyte meiosis (Adjusted P-value = 2.3 × 10^−4^). Restricting to the 145 most significantly differentially expressed genes identified by meta-analysis (P-value < 10^−19^ and standardized fold change >2) was able to cluster NEEC and EEC samples in k-means cluster analysis (83.2% accuracy; Fisher’s Exact P-value < 2.2 × 10^−16^; Fig. 2). Similar clustering was observed in analysis of TCGA RNA-Seq data from 92 EEC and 57 NEEC tumor samples generated by HiSeq (82.6% accuracy; Fisher’s Exact P-value < 2.2 × 10^−16^; Supplementary Figure 2).

### Functional enrichment and network analyses of the 145 most significantly differentially expressed genes

Because of computational limitations, functional enrichment analyses were restricted to the 145 most significantly differentially expressed genes identified by meta-analysis (P-value < 10^−19^ and standardized fold change >2). Since we have a total of 14,673 genes shared across all eight studies for differential expression analysis, we used these 14,673 genes as background for the calculation of significant P-values. In total, we found three significant functional terms: N4-(beta-N-acetylglucosaminyl)-L-asparaginase activity, Mucin type O-Glycan biosynthesis, and Walt’s disease. In our background gene list, there are only two genes (*AGA* and *ASRGL1*) related to N4-(beta-N-acetylglucosaminyl)-L-asparaginase activity. Both of these were detected in the 145 genes (GO:0003948, corrected P-value = 0.0192). For the KEGG pathway Mucin type O-Glycan biosynthesis, three genes (*GALNT4*, *GCNT3*, and *ST6GALNAC1*) were detected in our 145 genes (corrected P-value = 0.0456). The change of structure of mucin-type O-glycans can alter the adhesive properties of cells as well as cells’ potential to invade and metastasize in colon and breast cancers^17^. More interesting, we found five genes (*CDKN2A*, *COL8A2*, *RASSF6*, *TMC4*, and *TMC5*) from our 145 genes are associated with Walt’s disease (corrected P-value = 0.0040), which are infections in the skin caused by the human papillomavirus (HPV). In fact, the infection of HPV could precede the endometrial cancer progression^18^.

**Figure 3:**
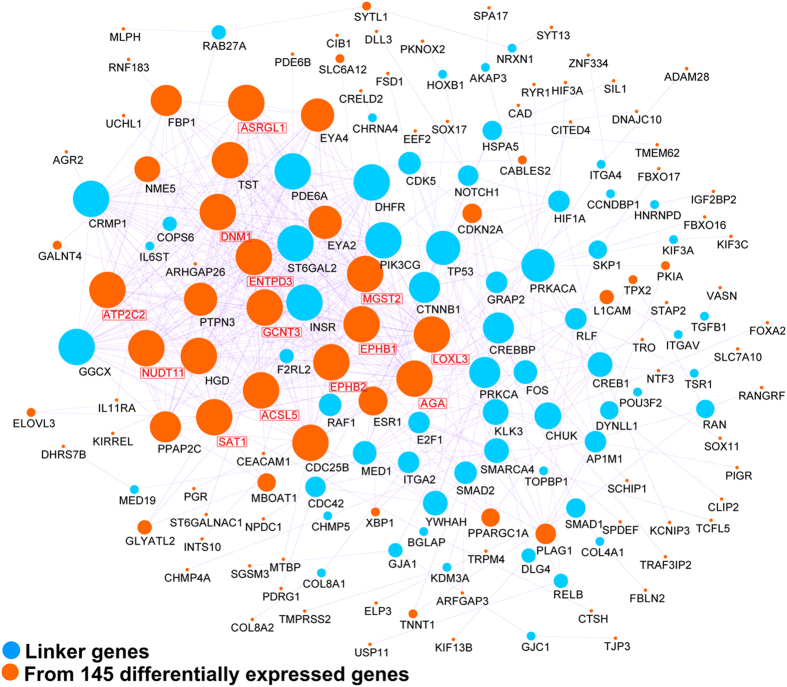
Network reconstruction and mutation analysis of the 145 most significantly differentially expressed genes between EEC and NEEC. (**A**) Reconstructed network using protein-protein interaction data. Genes shown in orange (n = 106) are from the 145-gene list. The remaining genes in blue (n = 62) are linker genes that bridge the 106 genes into the network. Hub genes have been denoted with red text and boxes. EEC - endometrioid endometrial cancer; NEEC - non-endometrioid endometrial cancer.

To explore the global interaction features of the 145 most significantly differentially expressed genes, we further mapped this gene list to the human pathway-based interactome. As shown in Fig. 3, we were able to reconstruct a network of 168 genes, of which 106 (63%) were from the 145 gene list, and 570 gene-gene interactions. The majority of genes in the reconstructed map are linked to each other and the vast majority of genes (~90%) in the network are connected by less than five steps. Twenty hub genes (defined as nodes with 20 or more connections) were identified in our network, of which 13 (65%) were from the 145 gene list: *AGA* [33], *DNM1* [32], *EPHB2* [30], *ATP2C2* [29], *ENTPD3* [28], *NUDT11* [28], *ASRGL1* [27], *ACSL5* [27], *LOXL3* [27], *EPHB1* [26], *SAT1* [26], *GCNT3* [23], *MGST2* [22]. Interestingly, both *AGA* and *ASRGL1*, related to N4-(beta-N-acetylglucosaminyl)-L-asparaginase activity, are highly connected in the reconstructed network, which may provide clues as to how these two genes interact with other cancer genes.

### EEC-only Survival Analysis

**Figure 4:**
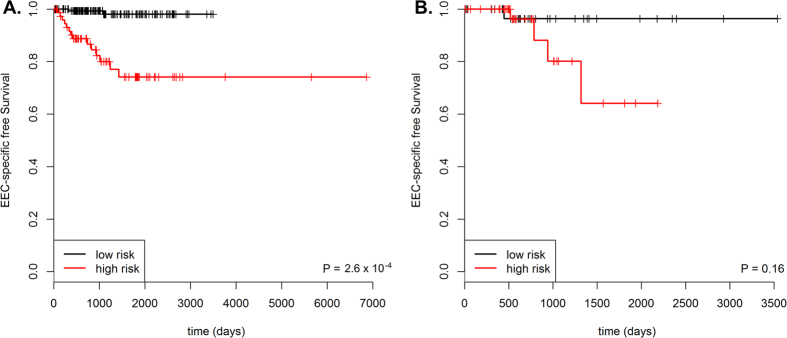
Kaplan-Meier plots for EC-specific survival for high- and low-risk EEC patient groups identified using 31-gene prognostic signature. (**A**) 241 samples with RNA-Seq data generated by the Illumina GA platform from the TCGA. (**B**) 92 samples with RNA-Seq data generated by the Illumina HiSeq platform from the TCGA. EEC - endometrioid endometrial cancer; TCGA - The Cancer Genome Atlas; EC-specific survival - endometrial cancer-specific survival.

Expression from 121 of the 1,581 genes validated as differentially expressed between NEEC and EEC associated with EC-specific survival in EEC patients at a P-value < 0.005 (Table 2). Twenty-three of these 121 genes were among the 145 most significantly differentially expressed genes (Supplementary Table 2). Using these 121 genes as input, forward selection, likelihood-based modelling selected a 9-gene signature as being associated with EC-specific survival among EEC patients. The expression of the 9-gene signature were used to construct a prognostic index for each patient (see methods), which associated with poorer survival (log-rank P-value = 2.6 × 10^−4^, Fig. 4A). This association remained significant after multivariate analysis, adjusting for clinical covariates, stage and grade (HR 8.2; 95% CI 1.7–40.7; P-value = 0.01). Prognostic indexes were calculated for 92 non-overlapping TCGA patients with RNA-Seq data generated using the Illumina HiSeq platform, however the difference in EC-specific survival between the high and low risk groups was non-significant in this smaller dataset (log-rank P-value = 0.16; Fig. 4B).

## Discussion

In this study we have investigated differential gene expression between NEEC and EEC to identify 1,253 genes that are involved in aggressive disease, thus providing insight into the biological underpinnings of these two groups of endometrial cancer. By taking a meta-analysis approach, using stringent selection criteria and performing validation in a large, independent RNA-Seq data, we have minimized false positive associations and produced genes robustly associated with NEEC. The reliability of this analysis was indicated by the validation of 77% of the identified genes in RNA-Seq data from an independent set of TCGA samples. We then further explored whether genes associated with aggressive disease were associated with poor prognosis among women with EEC, identifying a 9-gene signature which was able to group EEC patients as high- or low-risk, which remained significant after adjustment for clinical features, stage and grade.

We identified 601 genes to be upregulated in EEC compared to NEEC. Unsurprisingly, given the accepted relationship of EEC with unopposed estrogen exposure, the most significantly upregulated genes included estrogen responsive genes (*KIAA1324*, *TFF3*, *MLPH*) and genes involved in estrogen-related processes (*FOXA2*, *ESR1*, *PGR*). Expression of genes involved in epithelial (Ca2+) signaling (*ATP2C2*, *TRPM4*) were also found to be highly associated with EEC, a pathway thought to be important for epithelial cancer cells^19^. Two other genes identified as upregulated in ECC have previously been reported to be overexpressed in EEC by numerous studies; *TFF3*^10^,^13^,^14^,^15^ and *CEACAM1*^10^,^14^. Both are involved in extra-cellular matrix processes and cell-adhesion pathways and have been implicated in other cancer types reviewed in refs 20 and 21.

There were 652 genes found to be upregulated in NEEC tissue compared with EEC. A number of genes are involved in cell-cycle processes, such as *GPR19*, *CDKN2A*, *USP11* and *MX2*. *GPR19* encodes for a G protein-coupled receptor and is reported to be associated with lung cancer and melanoma^22^. It is suggested that G protein-coupled receptors are the most “druggable” family of proteins^23^. The significant association of *GPR19* expression in NEEC observed warrants further investigation into the utility of drugs targeting *GPR19* in treatment of this disease. Defects in the mitotic spindle checkpoint genes have been implicated in aneuploidy, a well-recognized feature of NEECs, and a previous gene expression study^12^ found that genes involved in the regulation of the mitotic spindle checkpoint were overexpressed in NEEC. Our results are consistent with this previous study, with mitotic cell cycle pathway genes found to be enriched in pathway analyses of differentially expressed genes.

Network reconstruction identified two N4-(beta-N-acetylglucosaminyl)-L-asparaginase activity genes (*AGA* and *ASRGL1*) as hub genes. This is the first observation of the significant differential expression of these two N4-(beta-N-acetylglucosaminyl)-L-asparaginase genes, across multiple endometrial cancer expression datasets. The additional high connections in the constructed network also implicate these two genes as potentially promising biomarkers for NEEC.

The most significantly upregulated gene in NEEC was *L1CAM* (L1 cell adhesion molecule), a member of the immunoglobulin super family, which is involved in embryonic brain development^24^. *L1CAM* is thought to be implicated in epithelial-to-mesenchymal transition, a critical event in tumor progression^25^ and its expression has been reported be associated with many cancers including breast, gastric and colorectal cancers reviewed by ref. 26. Expression of *L1CAM* has been reported to be associated with aggressive subtypes of endometrial cancer, including NEECs^27^. Furthermore, *L1CAM* has been reported to have utility as a predictor of clinical outcome in endometrial cancer^27^,^28^. Consistent with these publications, *L1CAM* was found to be significantly associated with EC-specific survival among EEC patients (P-value = 8.7 × 10^−4^).

The 9-gene EC-specific survival signature included genes previously implicated in other cancers, particularly colorectal cancer. Reduced expression of *EPHB2*^29^ and *PDLIM1*^30^ are reported to be indicators of poor prognosis of colorectal cancer. Both genes appear to exhibit tissue-specific effects, with upregulation of *EPHB2* reported to be associated with poor breast cancer survival^31^ and elevated expression of *PDLIM1* reported to promote metastatic processes in breast and glioma^32^,^33^. *C4BPA* and *NLRC3*, genes involved in immune processes, are reported to be dysregulated in pancreatic^34^ and colorectal cancer, respectively^35^. *FBP1* plays a role in glucose metabolism and aerobic glycolysis, and has been reported to be downregulated in hepatocellular carcinoma, colorectal, breast, gastric, and renal cancer, reviewed in ref. 36. Downregulation of *FBP1* is reported to contribute to tumor progression and poor survival of hepatocellular carcinoma^36^ and renal cell carcinoma patients^37^ and has been touted as a target for therapeutic interventions for these diseases. Given the results for *FBP1* expression in our study, it is conceivable that therapeutics developed targeting *FBP1* may also be beneficial in the treatment of EEC.

In conclusion, we have used a stringent meta-analysis and validation approach to identify distinct gene expression profiles in EEC and NEEC tumors. Importantly, a 9-gene signature was associated with poorer EC-specific survival in EEC patients, indicating its utility to predict prognosis. These genes may also provide new targets for therapy or the opportunity for the repositioning of currently available drugs. Results from this study contribute to the understanding of the molecular mechanisms of endometrial cancer subtypes, and have identified avenues to develop improved methods for identifying and treating poor prognosis patients with this disease.

## Materials and Methods

### Acquisition of Microarray Expression Datasets

A literature review and repository search was conducted up to September 2015 to identify endometrial cancer microarray expression studies. Twenty-one endometrial cancer microarray studies were accessed from publication supplementary data, the NCBI Gene Expression Omnibus (GEO; http://www.ncbi.nlm.nih.gov/geo/), ArrayExpress (https://www.ebi.ac.uk/arrayexpress/), or by contacting the publication authors (Supplementary Table 3). Microarray data generated by TCGA were downloaded from TCGA data portal (https://tcga-data.nci.nih.gov/tcga/).

Of these 21 studies, eight were excluded as follows: four studies lacked EEC and NEEC subtype information (E-GEOD-36389, E-GEOD-21882, E-GEOD-63678 & refs 38 and 39); one study using a custom platform of which the probe annotations could not be updated^40^; four studies performed by the same research lab (ArrayExpress accession no: E-GEOD-14860, E-MTAB1358, E-MTAB-1007 and E-MTAB-2532) included overlapping sample sets, and, thus, only the largest study (E-MTAB-2532), was selected for inclusion in our analysis.

### Expression Microarray Analysis

Analysis of microarray expression data was performed used the MetaOmics suite of packages in R^41^. Gene probe annotations were updated for each dataset using SOURCE (http://source-search.princeton.edu/) and expression data log transformed (by taking the logarithmic values of the signals to the base of two). Multiple probes mapping to the same gene were summarized using the inter-quartile range method, since this method is considered to be more biologically relevant than averaging probes values^42^. Expression data were filtered to remove the bottom 20% of unexpressed and uninformative genes (i.e. genes with low mean expression intensity values and low variation in expression intensity values) as advised by the authors of the MetaOmics packages.

Quality control measures were generated using the MetaQC package^41^, to identify studies which should be excluded from the meta-analysis, such as outlier studies with gene co-expression profile considered inconsistent using both unsupervised pair-wise comparisons between studies and pathway knowledge provided by curated gene sets from MSigDB (http://software.broadinstitute.org/gsea/msigdb). Other measures generated included those aimed at quantifying the reproducibility of differentially expressed genes.

Genes common across all studies were extracted and datasets merged. Differentially expressed genes were identified for each study using moderated t-tests and p-values combined using Fisher’s combined probability test. Gene expression level differences between EEC and NEEC tissue for each study were expressed as an effect size, a unit-free standardized mean difference, and combined using a random effects model. Adjustment for multiple comparisons on the combined p-values was performed using the false discovery rate procedure of Benjamini and Hochberg. All meta-analyses were performed using the MetaDE package^41^.

### TCGA RNA-Seq data validation

RNA-Seq RSEM gene expression data (level 3 generated for 317 TCGA EEC and NEEC tissues by the Illumina GA platform and 162 TCGA EEC and NEEC by the Illumina HiSeq platform were downloaded from the cancer browser (https://genome-cancer.ucsc.edu/proj/site/hgHeatmap/). RNA-Seq data generated by the two sequencing platforms (GA and HiSeq) were treated as two separate datasets to avoid bias from batch effects. Samples that overlapped with the TCGA microarray dataset were excluded from RNA-Seq analysis. RNA-Seq data was normalized using the voom function from the package limma in R. Unsupervised hierarchical clustering was performed using the ggplot package in R. Class comparison, class prediction and KEGG pathway enrichment were performed using BRB-ArrayTools software (http://brb.nci.nih.gov/BRB-ArrayTools/index.html).

### Function enrichment analysis

Functional enrichment analysis using WebGestalt (https://www.webgestalt.org/) was performed to identify potentially important gene pathways from KEGG and and gene ontology (GO). All 14,673 genes shared across all eight studies for differential expression analysis were used as background in these analyses. P-values were corrected for multiple testing by Benjamini-Hochberg adjustment and only pathways with a corrected P-value < 0.01 for any gene set were considered significant.

### Network Analysis

Recent advances in high-throughput technologies have generated data for protein-protein interaction (PPI). This huge data have stimulated pathway reconstruction for improving the systems-level understanding of specific cellular events. However, most of PPI data derived from mass-spectrum and yeast-two-hybrid technologies are only physical interaction, which may not really exist *in vivo*. Additionally, the physical interaction-based PPI network tends to a highly skewed degree distribution, which may not represent the global interactome involving basic cellular processes. To avoid the inaccuracy, a non-redundant pathway-based human interactome was built based on the PPIs in PathCommons^43^. These PPIs are derived from human-curated pathway databases, including HumanCyc, the NCI signaling pathway database, Reactome, and KEGG pathway. The final human pathway-based interactome contains 3629 genes and 36034 interacting edges. Using a module searching method as previously described^44^, we extracted a subnetwork from all human pathway-based interactomes. This algorithm mapped all interesting input genes to the human interactome, and then it generated a sub-network with the shortest paths between input genes and other genes. Network visualization was performed using Cytoscape 2.8^45^.

### Survival analysis

Validated genes were used in survival prediction analyses of 241 EEC patients from TCGA with Illumina GA RNA-Seq and outcome data available, using the survival package in R. Gene expression was grouped using the auto-cutoff method as described in ref. 46. Briefly, each percentile of expression between the first and third quartiles was computed and best performing threshold was used as the cut-off in the Cox proportional hazards model. Forward selection, likelihood-based modelling to identify the 9-gene prognostic signature from all genes associated with EC outcome was performed using the rbserv package in R. Prognostic indexes using the 9-gene signature were calculated for each patient by subtracting the sum of the normalised expression values of genes with lower expression in EEC compared to NEEC (*PDLIM1, FBP1, NLRC3, ST6GALNAC1, C4BPA*) from the sum of expression values of genes with higher expression (*PPP2R3A, TRIM46, EPH2, PRRG1*). Indexes were grouped into low- and high-risk group using the auto-cutoff method as described above. Kaplan-Meier survival curves were and differences between groups assessed using log-rank test. Multivariate analyses of other clinical features were performed using Cox proportional hazard models. Endpoint for endometrial cancer specific survival (EC-specific survival) was defined as time from diagnosis until death with endometrial tumor present. Results were then tested in 92 EEC patients from TCGA with Illumina HiSeq RNA-Seq and outcome data available.

## Additional Information

**How to cite this article**: O’Mara, T. A. *et al*. Meta-analysis of gene expression studies in endometrial cancer identifies gene expression profiles associated with aggressive disease and patient outcome. *Sci. Rep*. **6**, 36677; doi: 10.1038/srep36677 (2016).

**Publisher’s note:** Springer Nature remains neutral with regard to jurisdictional claims in published maps and institutional affiliations.

## Supplementary Material

Supplementary Information

## Figures and Tables

**Table 1:** Gene expression microarray studies included in meta-analysis.

Study Reference Number	Study Name/Accession Number	NEEC (n)	EEC (n)	Platform	Probes (n)	Reference
1	E-MTAB-2532	39	159	Agilent 4 × 44K	30356	Tangen *et al*. 2014 PLoS One 9(5):e98069
2	E-GEOD-2109	38	162	Affymetrix U133 Plus 2.0	42995	http://www.intgen.org/
3	E-GEOD-56026	12	51	Affymetrix U133 Plus 2.0	42995	Kharma *et al*. 2014 Cancer Res 74(22):6519–30
4	GSE24537	11	22	Illumina HT-12v3.0	35263	Mhawch-Fauceglia *et al*. 2011 PLoS One 6(3):e18066
5	E-GEOD-23518	10	10	Illumina HT-12v3.0	48785	Mhawch-Fauceglia *et al*. 2010 PLoS One 5(11):e15415
6	TCGA	13	41	Agilent G4502A	17814	https://tcga-data.nci.nih.gov/tcga/
7	E-GEOD-17025	12	79	Affymetrix U133 Plus 2.0	42995	Day *et al*. 2011 BMC Bioinformatics 12:213
8	GSE32507	14	24	Agilent 4 × 44 K	40990	Chiyoda *et al*. 2012 Genes Chromosomes Cancer 51(3):229–39
*9*	*Shedden*	*5*	*13*	*Affymetrix Hu6800*	*6245*	*Shedden et al. 2005 Clin Cancer Res 11:2123–2131*
*10*	*Risinger*	*16*	*19*	*Custom*	*7435*	*Risinger et al. 2003 Cancer Research 63:6–11*
*11*	*Moreno-Bueno*	*11*	*24*	*Custom*	*6439*	*Moreno-Bueno et al. 2003 Cancer Research 63:5697–5702*
*12*	*Zorn*	*28*	*7*	*Custom*	*5661*	*Zorn et al. 2005 Clin Cancer Res 11(18):6422–6430*
	**Total included in final analysis**	**149**	**548**			

Studies in italics were regarded as outliers in quality control assessment and excluded from the final analysis.

NEEC: Non-endometrioid endometrial cancer, EEC: Endometrioid endometrial cancer.

**Table 2:** Genes included in 9-gene signature predictive of endometrial cancer specific survival.

Symbol	Gene	Cox proportional regression p-value
*PRRG1*	Proline Rich Gla (G-Carboxyglutamic Acid) 1	2.0 × 10^−3^
*C4BPA*	Complement Component 4 Binding Protein, Alpha	5.4 × 10^−4^
*PDLIM1*	PDZ and LIM Domain 1	7.9 × 10^−4^
*FBP1*	Fructose-Bisphosphatase 1	2.2 × 10^−3^
*PPP2R3A*	Protein Phosphotase 2 Regulatory Subunit B”, Alpha	7.7 × 10^−3^
*NLRC3*	NLR Family, CARD Domain Containing 3	9.2 × 10^−4^
*TRIM46*	Tripartite Motif Containing 46	1.8 × 10^−3^
*ST6GALNAC1*	ST6 N-Acetylgalactosaminide Alpha-2,6-Sialyltransferase 1	3.6 × 10^−3^
*EPHB2*	EPH Receptor B2	2.1 × 10^−3^
