# Supplementary material for: Meta-analysis of gene expression studies in endometrial cancer identifies gene expression profiles associated with aggressive disease and patient outcome
Source: Sci Rep. 2016 Nov 10;6:36677. doi: 10.1038/srep36677 (PMC5103206; doi:10.1038/srep36677)
Supplement: Supplementary Information [file srep36677-s1.pdf]

Supplementary information for:

Meta-analysis of gene expression studies in endometrial cancer identifies gene expression profiles associated with aggressive disease and patient outcome.

Tracy A O'Mara[1*], Min Zhao[2], Amanda B Spurdle[1]

[1]Genetics and Computational Biology Department, QIMR Berghofer Medical Research Institute, Herston, QLD 4006, Australia

[2]School of Engineering, Faculty of Science, Health, Education and Engineering, University of the Sunshine Coast, Queensland, 4558, Australia

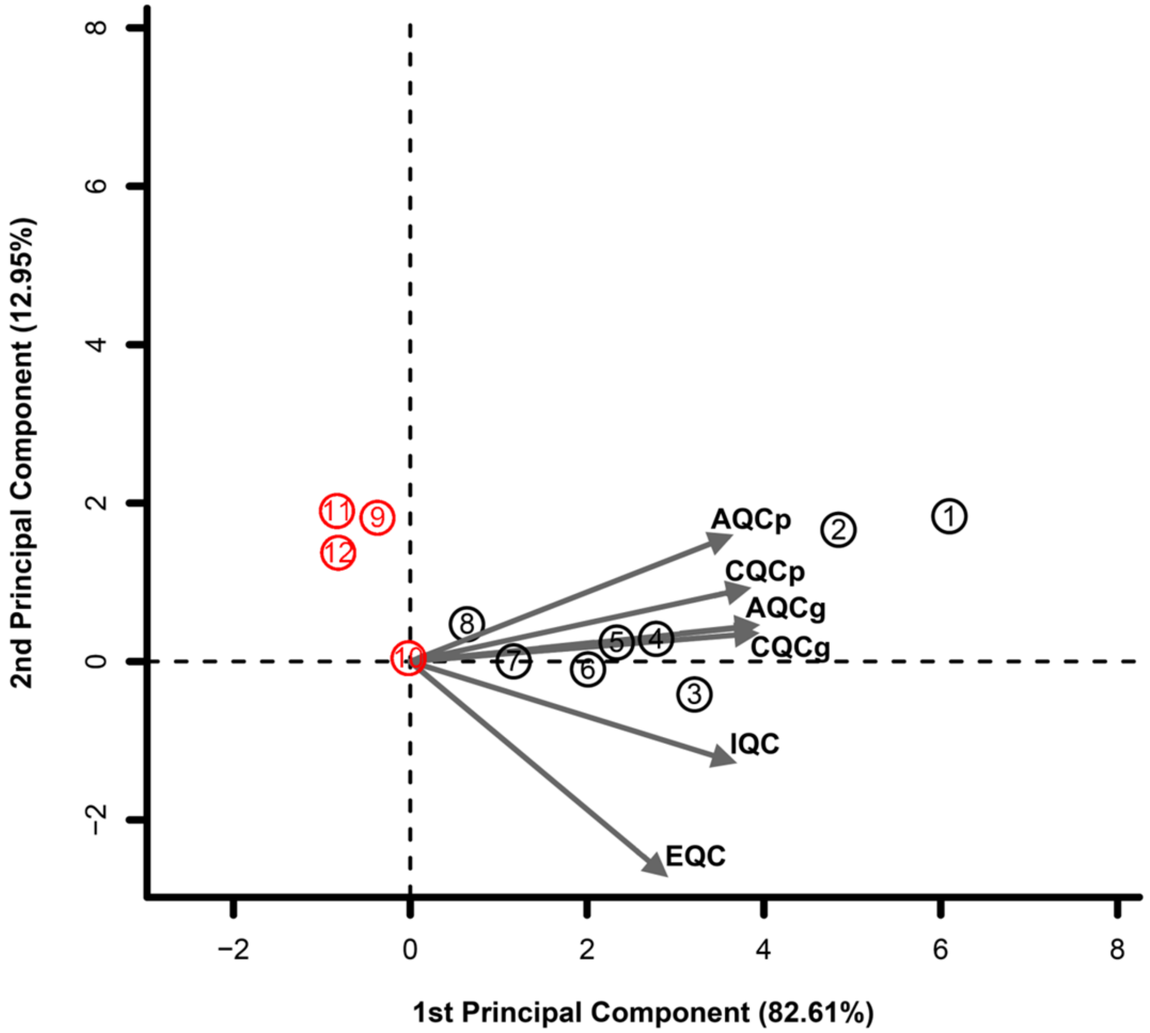

Supplementary Figure 1. Principal components analysis using co-expression and reproducibility estimates from MetaQC quality control analysis of 12 microarray studies. Studies highlighted in red were excluded from the final microarray meta-analysis. Study ID can be found in Table 1.

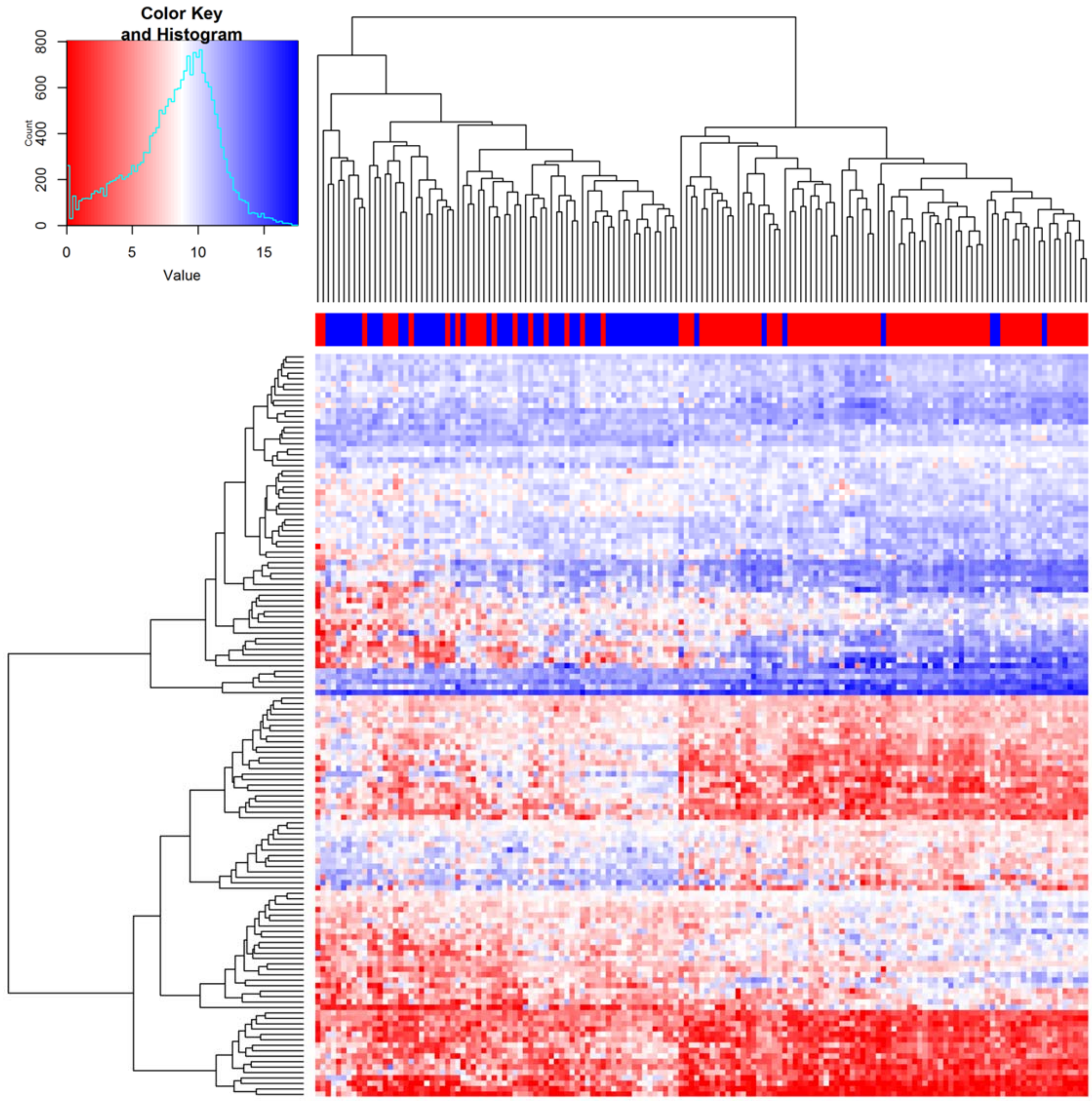

Supplementary Figure 2. Gene expression patterns in endometrial cancer patients using RNA-Seq HiSeq data from The Cancer Genome Atlas. Unsupervised hierarchical clustering and heatmap showing individual expression pattern in 145 most significantly differentially expressed genes identified by microarray meta-analysis. Patient subgroup (NEEC - blue, EEC - red) is depicted by the bar across the top of the heat map. Normalized expression value is displayed by the heatmap, where blue represents upregulated genes and red represents downregulated genes. EEC - endometrioid endometrial cancer; NEEC - non-endometrioid endometrial cancer.

Supplementary Table 1. Quality control measures assessing microarray studies suitability for inclusion in meta-analysis. Italicised studies were excluded from final analysis.

Study No.	Study/Accession No.	Internal Quality Control Score	External Quality Control Score	Consistency Quality Control Score (genes)	Consistency Quality Control Score (pathways)	Accuracy Quality Control Score (genes)	Accuracy Quality Control Score (pathways)	Quality Control Rank
1	E-MTAB-2532	8.09	2.82	142.37	137.48	67.18	69.96	1.67
2	E-GEOD-2109	7	2.69	105.1	136.08	40.36	63.32	2.83
3	E-GEOD-56026	8	3.4	92.17	48.3	31.59	16.73	3.33
4	E-GEOD-24537	6.34	2.9	58.99	56.27	30.23	27.77	3.83
5	E-GEOD-23518	4.52	2.96	68.86	52.77	27.01	16.9	4.5
6	TCGA	4.59	3.22	45.46	50.95	22.49	13.04	5.33
7	E-GEOD-17025	5.39	2.49	17.96	26.47	8.47	14.47	6.83
8	E-GEOD-32507	4.43	1.72	14.54	24.37	6.59	9.24	8.67
9	*Shedden*	*1.05*	*0.07*	*20.21*	*12*	*7.26*	*6.39*	*9.33*
10	*Risinger*	*1.62*	*2.52*	*2.59*	*2.31*	*2.82*	*4.38*	*9.67*
11	*Moreno-Bueno*	*0.03*	*0.08*	*0.46*	*4.25*	*0.22*	*11.23*	*10.83*
12	*Zorn*	*0.27*	*0.6*	*1.24*	*1.37*	*0.45*	*3.37*	*11.17*

Supplementary Table 2. Details for 2,053 genes differentially expressed between EEC and NEEC, identified by microarray meta-analysis.

EEC - endometrioid endometrial cancer, NEEC - non-endometrioid endometrial cancer, SMD - Standardised Mean Difference, FDR - False Discovery Rate, Var - Variance

Gene	145 gene list	1253 gene list	Associated with EEC-specific survival?	Up/Down Regulated in NEEC	average standardised fold change	Microarray Meta FDR	RNASeq FDR	Individual microarray study results																							
								TCGA			E-MTAB-2532			E-GEOD-56026			GSE32507			GSE24537			E-GEOD-23518			E-GEOD-17025			E-GEOD-2109		
								SMD	Var	P-value	SMD	Var	P-value	SMD	Var	P-value	SMD	Var	P-value	SMD	Var	P-value	SMD	Var	P-value	SMD	Var	P-value	SMD	Var	P-value
L1CAM	Yes	Yes	Yes	Up	2.65	1.83E-19	1.5E-19	2.07	0.14	9.09E-07	1.29	0.04	5.91E-06	1.84	0.13	1.00E-20	0.36	0.11	1.28E-01	1.10	0.15	5.82E-03	0.92	0.22	2.47E-02	2.48	0.13	1.00E-20	1.18	0.04	1.00E-20
GPR19	Yes	Yes	No	Up	2.61	1.83E-19	1.5E-19	1.88	0.13	1.75E-05	1.59	0.04	1.00E-20	1.76	0.13	5.66E-05	1.49	0.14	2.86E-05	0.61	0.14	3.27E-01	1.04	0.23	1.11E-01	1.50	0.11	1.16E-03	1.21	0.04	1.00E-20
RYR1	Yes	Yes	No	Up	2.59	1.83E-19	1.5E-19	1.47	0.12	1.29E-04	1.60	0.04	1.00E-20	1.33	0.12	5.27E-04	1.29	0.14	2.45E-05	1.24	0.16	4.82E-03	1.82	0.28	5.97E-04	0.85	0.10	2.54E-02	1.38	0.04	1.00E-20
FAM24B	Yes	Yes	No	Up	2.55	1.83E-19	1.5E-19	1.88	0.13	4.54E-07	1.20	0.04	4.54E-07	1.53	0.12	2.52E-04	0.88	0.12	2.36E-03	1.32	0.16	4.06E-02	1.93	0.29	2.36E-02	1.41	0.11	2.86E-04	0.68	0.03	2.39E-04
USP11	Yes	Yes	No	Up	2.52	1.83E-19	1.5E-19	0.89	0.11	5.43E-03	1.18	0.04	3.18E-06	1.07	0.11	4.38E-03	0.94	0.12	1.54E-02	1.52	0.17	6.90E-04	2.49	0.36	6.57E-05	1.30	0.11	2.64E-04	1.30	0.04	8.63E-06
PLAG1	Yes	Yes	No	Up	2.52	1.83E-19	1.5E-19	2.28	0.15	1.00E-20	1.55	0.04	1.00E-20	1.89	0.13	1.00E-20	0.63	0.12	1.64E-02	0.98	0.15	1.15E-01	1.07	0.23	6.22E-02	1.07	0.10	1.52E-04	1.21	0.04	6.82E-07
UCHL1	Yes	Yes	Yes	Up	2.48	1.83E-19	1.5E-19	1.46	0.12	6.82E-07	1.76	0.05	1.00E-20	1.54	0.12	1.36E-06	0.69	0.12	5.63E-03	0.98	0.15	1.64E-03	1.63	0.27	1.05E-04	0.91	0.10	8.41E-04	1.53	0.04	1.00E-20
EYA4	Yes	Yes	Yes	Up	2.45	1.83E-19	1.5E-19	1.31	0.12	5.47E-05	1.68	0.04	1.00E-20	1.33	0.12	2.32E-05	1.07	0.13	1.22E-04	0.94	0.15	9.38E-02	1.17	0.23	1.34E-02	1.46	0.11	2.04E-06	1.41	0.04	1.00E-20
MX2	Yes	Yes	No	Up	2.44	1.83E-19	1.5E-19	2.06	0.14	4.54E-07	1.11	0.04	4.54E-07	1.12	0.11	3.08E-04	0.27	0.11	3.01E-01	1.98	0.20	1.48E-05	1.38	0.25	1.78E-03	1.18	0.10	1.40E-04	1.19	0.04	1.14E-06
HIF3A	Yes	Yes	No	Up	2.40	1.83E-19	1.5E-19	1.81	0.13	2.61E-05	2.17	0.05	1.00E-20	0.91	0.11	3.66E-03	1.47	0.14	1.36E-06	0.37	0.14	6.12E-01	1.05	0.23	2.17E-01	1.33	0.11	2.39E-03	1.03	0.04	1.00E-20
DLL3	Yes	Yes	No	Up	2.40	1.83E-19	1.5E-19	0.89	0.11	5.60E-03	1.74	0.05	1.00E-20	1.07	0.11	6.55E-03	2.07	0.17	1.00E-20	1.18	0.16	5.03E-03	1.97	0.30	4.35E-04	0.32	0.10	5.16E-01	0.89	0.03	3.18E-06
KIRREL	Yes	Yes	No	Up	2.39	1.83E-19	1.5E-19	1.27	0.12	1.13E-03	1.29	0.04	4.54E-07	1.11	0.11	6.22E-04	0.74	0.12	1.31E-02	1.86	0.19	1.79E-01	2.03	0.30	1.50E-01	1.48	0.11	2.66E-05	0.27	0.03	8.11E-02
WNK3	Yes	Yes	No	Up	2.39	1.83E-19	1.5E-19	1.08	0.11	8.04E-04	1.65	0.04	1.00E-20	1.13	0.11	2.17E-04	1.36	0.14	2.39E-05	1.39	0.17	2.78E-01	1.43	0.25	2.99E-01	1.14	0.10	3.15E-04	0.87	0.03	1.14E-06
ZNF334	Yes	Yes	No	Up	2.38	1.83E-19	1.5E-19	2.24	0.15	1.00E-20	0.88	0.04	2.02E-05	1.86	0.13	3.88E-05	0.94	0.12	6.72E-04	1.03	0.15	9.46E-02	1.06	0.23	2.77E-02	0.90	0.10	5.46E-03	1.07	0.04	1.00E-20
CDC25B	Yes	Yes	Yes	Up	2.36	1.83E-19	1.5E-19	1.93	0.14	2.50E-06	0.93	0.04	3.04E-05	1.33	0.12	3.82E-04	1.10	0.13	3.96E-03	1.71	0.18	7.50E-06	1.20	0.24	1.55E-03	0.61	0.10	3.72E-02	1.08	0.04	2.86E-05
MRGBP	Yes	Yes	No	Up	2.35	1.83E-19	0.00023	1.44	0.12	1.02E-03	1.22	0.04	3.09E-05	1.38	0.12	5.16E-04	0.86	0.12	3.45E-02	1.46	0.17	9.75E-05	1.58	0.26	2.15E-04	0.85	0.10	2.21E-02	1.09	0.04	5.60E-04
FBLN2	Yes	Yes	No	Up	2.35	1.83E-19	5.5E-05	2.14	0.14	1.00E-20	0.87	0.04	5.91E-06	1.02	0.11	2.12E-03	1.22	0.13	2.22E-04	1.47	0.17	9.04E-05	1.08	0.23	3.48E-03	1.22	0.10	2.03E-04	0.84	0.03	9.09E-07
IGF2BP2	Yes	Yes	No	Up	2.34	1.83E-19	1.5E-19	2.11	0.14	1.00E-20	1.38	0.04	1.00E-20	1.29	0.12	7.50E-06	0.35	0.11	1.49E-01	0.83	0.15	7.02E-03	1.07	0.23	3.09E-03	1.56	0.11	4.54E-07	1.23	0.04	1.00E-20
FAM110B	Yes	Yes	Yes	Up	2.33	1.83E-19	1.5E-19	0.84	0.11	2.36E-03	1.10	0.04	6.82E-07	1.61	0.12	5.68E-06	1.35	0.14	2.11E-05	1.07	0.15	1.52E-02	1.40	0.25	7.34E-03	1.33	0.11	6.16E-05	1.07	0.04	1.00E-20
CDKN2A	Yes	Yes	No	Up	2.32	1.83E-19	1.5E-19	1.97	0.14	1.00E-20	1.40	0.04	1.00E-20	1.72	0.13	1.00E-20	0.72	0.12	6.95E-03	1.01	0.15	5.32E-03	0.63	0.21	8.13E-02	1.09	0.10	1.14E-04	1.14	0.04	1.00E-20
COL8A2	Yes	Yes	No	Up	2.31	1.83E-19	1.5E-19	1.23	0.12	4.34E-05	1.08	0.04	4.54E-07	1.62	0.12	6.82E-06	1.43	0.14	2.48E-05	1.26	0.16	9.99E-04	1.24	0.24	3.14E-03	0.73	0.10	1.78E-02	1.07	0.04	1.00E-20
TMEM136	Yes	Yes	No	Up	2.30	1.83E-19	3.1E-05	1.35	0.12	1.74E-04	1.38	0.04	9.09E-07	1.23	0.11	1.59E-03	1.55	0.14	1.15E-03	1.39	0.17	4.43E-03	1.43	0.25	7.32E-03	0.80	0.10	2.23E-02	0.52	0.03	1.71E-02
PKIA	Yes	Yes	No	Up	2.30	1.83E-19	1.5E-19	1.04	0.11	2.55E-04	0.94	0.04	3.18E-06	1.29	0.12	3.95E-05	1.36	0.14	7.63E-05	1.20	0.16	2.95E-03	1.34	0.25	4.63E-03	0.85	0.10	3.66E-03	1.56	0.04	1.00E-20
IL11RA	Yes	Yes	No	Up	2.27	1.83E-19	0.00024	1.45	0.12	3.23E-05	1.05	0.04	3.18E-06	1.03	0.11	1.52E-03	1.71	0.15	2.36E-05	1.23	0.16	2.63E-04	1.48	0.25	2.32E-04	0.56	0.10	7.54E-02	0.98	0.03	1.34E-05
FBXO17	Yes	Yes	No	Up	2.23	1.83E-19	1.5E-19	1.53	0.12	7.04E-06	1.15	0.04	1.36E-06	1.20	0.11	2.44E-04	1.18	0.13	5.72E-05	1.24	0.16	6.00E-03	0.75	0.21	8.03E-02	1.42	0.11	6.91E-05	0.80	0.03	4.09E-06
DNM1	Yes	Yes	No	Up	2.21	1.83E-19	6.7E-06	0.90	0.11	7.17E-03	0.80	0.04	2.26E-04	1.12	0.11	1.32E-03	1.11	0.13	1.19E-03	1.29	0.16	1.64E-01	2.47	0.35	8.89E-02	0.90	0.10	2.00E-03	0.55	0.03	2.51E-03
EPHB1	Yes	Yes	No	Up	2.20	1.83E-19	1.5E-19	1.39	0.12	2.61E-05	0.29	0.04	2.24E-01	1.22	0.11	1.85E-04	1.49	0.14	1.98E-05	1.21	0.16	3.71E-02	1.90	0.29	5.28E-02	0.96	0.10	3.15E-03	0.66	0.03	2.93E-04
ZNF311	Yes	Yes	No	Up	2.20	1.83E-19	1.5E-19	1.33	0.12	1.38E-04	1.28	0.04	1.40E-04	1.37	0.12	2.47E-04	0.66	0.12	1.39E-02	0.30	0.14	6.28E-01	1.61	0.26	7.22E-02	1.74	0.11	1.70E-05	0.81	0.03	3.68E-05
HOXA4	Yes	Yes	No	Up	2.18	1.83E-19	1.5E-19	2.08	0.14	1.00E-20	1.21	0.04	1.00E-20	1.33	0.12	2.68E-04	0.56	0.12	2.80E-02	0.94	0.15	4.37E-02	0.55	0.21	1.92E-01	1.69	0.11	3.32E-04	0.64	0.03	2.79E-04
DDX25	Yes	Yes	No	Up	2.17	1.83E-19	1.5E-19	0.63	0.11	3.15E-02	0.96	0.04	2.36E-04	1.97	0.13	3.18E-06	1.20	0.13	1.41E-04	1.24	0.16	2.99E-01	1.06	0.23	1.79E-01	1.22	0.10	1.52E-02	0.69	0.03	2.03E-04
SYT13	Yes	Yes	No	Up	2.17	1.83E-19	1.5E-19	1.44	0.12	4.54E-07	1.21	0.04	1.00E-20	1.12	0.11	2.20E-04	0.90	0.12	9.80E-04	1.00	0.15	1.42E-02	1.48	0.26	3.26E-03	0.80	0.10	2.84E-03	1.01	0.04	1.00E-20
SLC22A17	Yes	Yes	No	Up	2.17	1.83E-19	2.8E-05	1.06	0.11	1.86E-03	0.95	0.04	3.68E-05	1.23	0.11	1.49E-03	0.50	0.12	1.62E-01	1.57	0.17	6.16E-05	1.70	0.27	1.36E-04	1.02	0.10	1.28E-02	0.93	0.03	4.54E-07
TPX2	Yes	Yes	Yes	Up	2.17	1.83E-19	1.5E-19	1.72	0.13	3.63E-06	1.03	0.04	1.36E-06	0.79	0.11	3.83E-03	1.21	0.13	6.30E-04	1.10	0.15	3.68E-03	1.20	0.24	4.47E-03	0.86	0.10	2.29E-03	1.03	0.04	1.36E-06
TUBB6	Yes	Yes	No	Up	2.17	1.83E-19	4E-05	1.22	0.12	7.98E-04	0.89	0.04	1.34E-05	0.78	0.11	5.85E-03	1.21	0.13	5.13E-04	1.35	0.16	3.56E-04	1.27	0.24	3.14E-03	1.33	0.11	7.72E-05	0.88	0.03	2.16E-05
KIF3C	Yes	Yes	Yes	Up	2.17	1.83E-19	1.3E-05	0.64	0.11	3.66E-02	1.22	0.04	1.36E-06	1.04	0.11	6.00E-03	1.01	0.13	6.69E-03	1.18	0.16	1.65E-01	2.06	0.31	2.86E-02	0.85	0.10	1.79E-02	0.92	0.03	3.01E-04
SCHIP1	Yes	Yes	No	Up	2.16	1.83E-19	1.5E-19	1.90	0.13	4.54E-07	1.04	0.04	2.27E-06	1.31	0.12	4.77E-05	1.33	0.14	1.02E-04	0.46	0.14	1.29E-01	0.17	0.20	4.64E-01	1.60	0.11	2.50E-06	1.07	0.04	1.36E-06
PNMA3	Yes	Yes	No	Up	2.16	1.83E-19	1.5E-19	1.31	0.12	1.18E-05	1.12	0.04	1.00E-20	1.56	0.12	3.43E-04	0.42	0.12	8.96E-02	0.92	0.15	1.49E-02	1.81	0.28	5.82E-04	1.31	0.11	4.01E-04	0.42	0.03	1.47E-02
CPVL	Yes	Yes	No	Up	2.15	1.83E-19	1.5E-19	1.45	0.12	1.18E-05	0.65	0.04	2.26E-04	1.19	0.11	2.02E-04	1.32	0.14	7.07E-05	1.25	0.16	2.44E-04	1.28	0.24	1.17E-03	0.79	0.10	5.52E-03	0.93	0.03	2.75E-05
NTF3	Yes	Yes	No	Up	2.15	1.83E-19	1.5E-19	0.87	0.11	4.24E-03	1.30	0.04	1.00E-20	1.85	0.13	1.23E-05	2.15	0.17	2.27E-07	0.45	0.14	3.24E-01	0.83	0.22	5.34E-02	0.92	0.10	5.03E-03	0.48	0.03	5.36E-03
EPHB2	Yes	Yes	Yes	Up	2.15	1.83E-19	1.5E-19	1.70	0.13	2.50E-06	1.09	0.04	9.09E-07	0.95	0.11	2.84E-03	1.34	0.14	1.68E-04	0.89	0.15	8.23E-02	0.64	0.21	2.39E-01	1.53	0.11	1.12E-04	0.70	0.03	3.27E-05
TRO	Yes	Yes	No	Up	2.15	1.83E-19	1.5E-19	1.04	0.11	9.84E-05	0.86	0.04	4.54E-06	1.16	0.11	5.31E-04	0.79	0.12	2.76E-03	1.48	0.17	2.27E-04	1.88	0.29	1.05E-04	0.78	0.10	3.62E-03	0.84	0.03	1.36E-06
CLIP2	Yes	Yes	No	Up	2.14	1.83E-19	0.00138	1.69	0.13	4.63E-05	0.83	0.04	2.21E-04	0.59	0.11	6.35E-02	1.37	0.14	9.58E-04	1.64	0.18	1.50E-03	1.27	0.24	2.16E-02	0.77	0.10	6.35E-02	0.63	0.03	7.29E-04
CABLES2	Yes	Yes	No	Up	2.13	1.83E-19	0.00018	1.68	0.13	9.72E-05	0.87	0.04	5.24E-04	1.13	0.11	1.60E-03	0.70	0.12	6.16E-02	0.85	0.15	1.32E-01	1.28	0.24	6.76E-02	1.20	0.10	9.21E-04	1.05	0.04	2.99E-04
VASN	Yes	Yes	No	Up	2.13	1.83E-19	1.5E-19	1.41	0.12	1.29E-05	0.79	0.04	7.59E-05	1.39	0.12	1.70E-04	0.42	0.12	2.12E-01	1.43	0.17	9.56E-05	1.39	0.25	3.82E-04	1.04	0.10	3.01E-03	0.83	0.03	1.35E-03
ELOVL3	Yes	Yes	No	Up	2.12	1.83E-19	1.5E-19	1.39	0.12	3.59E-05	1.19	0.04	5.23E-05	1.81	0.13	7.26E-04	0.94	0.12	7.97E-03	1.21	0.16	4.26E-02	0.61	0.21	2.73E-01	0.58	0.10	3.39E-01	0.94	0.03	2.27E-07
LOXL3	Yes	No	No	Up	2.11	1.83E-19	0.01385	0.97	0.11	1.35E-03	1.00	0.04	2.20E-05	1.24	0.12	2.00E-03	1.29	0.14	8.66E-04	1.69	0.18	4.25E-02	1.67	0.27	2.07E-02	0.37	0.10	3.65E-01	0.39	0.03	5.15E-02
SENP5	Yes	No	Yes	Up	2.11	1.83E-19	0.0001	2.46	0.16	6.82E-07	1.16	0.04	4.93E-05	1.18	0.11	1.69E-03	0.60	0.12	2.34E-01	0.69	0.14	1.59E-01	1.05	0.23	6.63E-02	0.62	0.10	4.59E-02	0.87	0.03	2.31E-03
SOX11	Yes	Yes	No	Up	2.11	1.83E-19	1.5E-19	1.20	0.11	3.41E-05	1.12	0.04	1.00E-20	0.92	0.11	7.38E-04	0.78	0.12	2.20E-03	1.24	0.16	1.04E-03	1.51	0.26	7.20E-04	0.40	0.10	1.66E-01	1.42	0.04	1.00E-20
NUDT11	Yes	No	No	Up	2.10	1.83E-19	0.0002	0.51	0.10	4.93E-02	0.73	0.04	5.00E-05	1.73	0.13	1.59E-06	1.40	0.14	1.07E-05	1.22	0.16	4.61E-03	1.34	0.24	8.72E-03	0.86	0.10	1.32E-02	0.77	0.03	1.65E-04
TCFL5	Yes	Yes	No	Up	2.10	1.83E-19	0.0001	1.46	0.12	1.13E-03	0.87	0.04	9.11E-05	1.24	0.12	2.45E-04	1.01	0.13	6.96E-03	1.09	0.15	3.52E-03	0.95	0.22	2.11E-02	1.23	0.10	1.01E-04	0.67	0.03	4.89E-03
FSD1	Yes	Yes	No	Up	2.09	1.83E-19	1.5E-19	0.82	0.11	7.94E-03	2.01	0.05	1.00E-20	0.87	0.11	7.67E-02	1.10	0.13	5.39E-04	0.85	0.15	9.55E-02	1.52	0.26	1.08E-01	0.49	0.10	1.82E-01	0.86	0.03	2.04E-06
CAD	Yes	Yes	No	Up	2.09	1.83E-19	0.00092	1.15	0.11	5.28E-03	1.16	0.04	1.41E-05	1.18	0.11	2.76E-03	0.57	0.12	1.88E-01	1.46	0.17	2.50E-03	1.83	0.28	2.14E-03	0.80	0.10	2.20E-02	0.34	0.03	4.40E-02
PKNOX2	Yes	No	No	Up	2.07	1.83E-19	validated	0.92	0.11	1.94E-03	0.47	0.04	1.03E-02	0.63	0.11	5.65E-02	2.05	0.17	1.00E-20	1.02	0.15	1.10E-01	1.29	0.24	3.86E-02	1.19	0.10	4.01E-03	0.80	0.03	4.09E-06
PDRG1	Yes	No	Yes	Up	2.06	1.83E-19	0.00073	1.67	0.13	2.49E-04	0.69	0.04	6.40E-03	1.17	0.11	4.36E-03	1.27	0.13	2.91E-02	0.58	0.14	8.67E-02	1.07	0.23	1.33E-02	0.51	0.10	1.27E-01	1.39	0.04	1.06E-04
SLC7A10	Yes	Yes	No	Up	2.05	1.83E-19	1.5E-19	1.17	0.11	3.61E-05	1.33	0.04	1.00E-20	0.78	0.11	3.99E-02	1.16	0.13	6.20E-05	0.90	0.15	4.59E-02	1.15	0.23	4.59E-02	1.17	0.10	4.80E-02	0.62	0.03	5.03E-04
CTCFL	Yes	Yes	No	Up	2.05	1.83E-19	1.5E-19	1.38	0.12	4.32E-06	1.49	0.04	1.00E-20	1.25	0.12	3.02E-05	0.81	0.12	3.49E-03	0.51	0.14	6.55E-02	0.88	0.22	1.44E-02	0.87	0.10	3.42E-03	1.10	0.04	1.00E-20
PPARGC1A	Yes	Yes	No	Up	2.05	1.83E-19	1.5E-19	1.32	0.12	4.63E-05	0.98	0.04	5.23E-06	0.96	0.11	1.89E-03	0.61	0.12	1.59E-02	0.96	0.15	5.00E-02	0.94	0.22	1.85E-01	1.79	0.11	4.54E-07	0.72	0.03	2.18E-05
SLC6A12	Yes	Yes	No	Up	2.04	1.83E-19	1.5E-19	2.78	0.17	1.00E-20	1.11	0.04	6.82E-07	0.71	0.11	2.18E-02	0.12	0.11	6.26E-01	0.78	0.15	1.17E-02	0.80	0.22	8.68E-02	1.35	0.11	3.48E-04	0.58	0.03	2.22E-03
KCNIP3	Yes	Yes	No	Up	2.04	1.83E-19	1.5E-19	1.35	0.12	7.04E-05	1.08	0.04	9.09E-07	0.07	0.10	8.63E-01	1.14	0.13	2.94E-04	1.11	0.16	3.15E-02	2.23	0.32	4.46E-03	0.75	0.10	1.16E-01	0.49	0.03	3.93E-03
MTBP	Yes	Yes	No	Up	2.03	1.83E-19	1.5E-19	1.46	0.12	7.70E-05	1.05	0.04	3.68E-05	1.10	0.11	5.59E-03	1.23	0.13	3.11E-03	0.66	0.14	2.51E-01	1.50	0.26	9.10E-02	0.85	0.10	1.05E-01	0.32	0.03	4.96E-02
PDE6B	Yes	Yes	No	Up	2.02	1.83E-19	1.5E-19	1.19	0.11	1.07E-04	1.34	0.04	1.00E-20	1.29	0.12	3.76E-03	0.79	0.12	3.79E-03	0.85	0.15	3.10E-01	1.28	0.24	1.05E-01	0.86	0.10	2.48E-02	0.52	0.03	2.97E-03
TNNT1	Yes	Yes	No	Up	2.00	1.83E-19	1.5E-19	1.33	0.12	2.50E-06	1.54	0.04	1.00E-20	0.46	0.10	4.03E-02	1.49	0.14	4.54E-07	0.59	0.14	1.79E-01	0.39	0.20	3.28E-01	0.95	0.10	1.21E-03	1.28	0.04	1.00E-20
XBP1	Yes	Yes	No	Down	0.50	1.83E-19	2E-05	-1.09	0.11	4.72E-04	-1.05	0.04	4.54E-07	-1.15	0.11	7.95E-04	-1.24	0.13	1.30E-03	-1.14	0.16	2.64E-04	-0.90	0.22	6.52E-03	-0.37	0.10	1.34E-01	-1.06	0.04	2.04E-06
MBOAT1	Yes	No	No	Down	0.50	1.83E-19	validated	-1.06	0.11	7.94E-04	-0.79	0.04	6.50E-05	-1.31	0.12	2.08E-04	-1.16	0.13	1.02E-03	-1.28	0.16	3.40E-03	-1.09	0.23	2.16E-02	-0.47	0.10	8.47E-02	-0.88	0.03	1.75E-04
MFAP3L	Yes	Yes	No	Down	0.50	1.83E-19	6.7E-06	-0.88	0.11	1.65E-03	-1.25	0.04	4.54E-07	-1.15	0.11	1.89E-04	-1.16	0.13	2.25E-04	-1.37	0.16	2.31E-02	-0.77	0.21	2.06E-01	-0.83	0.10	1.56E-03	-0.63	0.03	1.57E-04
EEF2	Yes	No	No	Down	0.50	1.83E-19	1.5E-05	-1.37	0.12	6.08E-04	-0.91	0.04	1.10E-04	-1.09	0.11	1.01E-02	-0.32	0.11	3.84E-01	-1.20	0.16	1.90E-04	-0.63	0.21	3.38E-02	-1.50	0.11	5.68E-06	-1.04	0.04	5.29E-04
CCDC125	Yes	Yes	No	Down	0.50	1.83E-19	0.00011	-1.13	0.11	1.71E-04	-0.83	0.04	2.68E-05	-1.48	0.12	2.14E-04	-1.37	0.14	7.75E-04	-1.00	0.15	1.13E-01	-0.62	0.21	4.04E-01	-0.75	0.10	9.87E-03	-0.88	0.03	2.43E-05
SLFN5	Yes	No	No	Down	0.49	1.83E-19	0.04835	-1.19	0.11	1.41E-04	-0.66	0.04	8.78E-04	-1.17	0.11	7.91E-04	-1.06	0.13	2.57E-03	-1.15	0.16	2.45E-02	-0.88	0.22	1.44E-01	-1.12	0.10	4.24E-04	-0.93	0.03	2.04E-06
TST	Yes	No	No	Down	0.49	1.83E-19	validated	-0.68	0.11	2.72E-02	-1.06	0.04	5.45E-06	-1.08	0.11	2.68E-03	-1.36	0.14	8.23E-04	-1.20	0.16	2.48E-04	-0.85	0.22	1.18E-02	-0.96	0.10	1.84E-03	-1.04	0.04	1.59E-06

Supplementary Table 2. Details for 2,053 genes differentially expressed between EEC and NEEC, identified by microarray meta-analysis.

EEC - endometrioid endometrial cancer, NEEC - non-endometrioid endometrial cancer, SMD - Standardised Mean Difference, FDR - False Discovery Rate, Var - Variance

Gene	145 gene list	1253 gene list	Associated with EEC-specific survival?	Up/Down Regulated in NEEC	average standardised fold change	Microarray Meta FDR	RNASeq FDR	Individual microarray study results: TCGA			E-MTAB-2532			E-GEOD-56026			GSE32507			GSE24537			E-GEOD-23518			E-GEOD-17025			E-GEOD-2109		
								SMD	Var	P-value	SMD	Var	P-value	SMD	Var	P-value	SMD	Var	P-value	SMD	Var	P-value	SMD	Var	P-value	SMD	Var	P-value	SMD	Var	P-value
TMC4	Yes	No	No	Down	0.49	1.83E-19	validated	-0.32	0.10	2.40E-01	-1.03	0.04	2.50E-06	-0.69	0.11	1.40E-02	-1.76	0.15	7.72E-06	-1.48	0.17	7.08E-04	-1.09	0.23	2.72E-02	-0.63	0.10	4.08E-02	-1.24	0.04	1.00E-20
RSPH1	Yes	Yes	No	Down	0.49	1.83E-19	0.00012	-0.83	0.11	1.94E-03	-1.08	0.04	1.00E-20	-0.65	0.11	9.29E-03	-1.59	0.15	8.41E-06	-1.09	0.15	1.07E-03	-0.91	0.22	1.72E-02	-1.25	0.10	1.91E-05	-0.84	0.03	2.27E-07
MPZL2	Yes	Yes	Yes	Down	0.49	1.83E-19	0.00043	-0.81	0.11	9.11E-03	-0.82	0.04	2.75E-05	-0.83	0.11	5.29E-03	-0.96	0.13	3.82E-03	-1.53	0.17	1.77E-05	-1.60	0.26	1.21E-04	-0.70	0.10	7.98E-03	-1.00	0.03	1.00E-20
GCNT3	Yes	Yes	Yes	Down	0.49	1.83E-19	9.7E-05	-0.84	0.11	9.92E-04	-1.44	0.04	1.00E-20	-1.37	0.12	2.00E-05	-2.05	0.17	1.00E-20	-0.72	0.14	1.16E-02	-0.39	0.20	1.73E-01	-0.67	0.10	1.01E-02	-0.78	0.03	6.82E-06
RANGRF	Yes	Yes	No	Down	0.49	1.83E-19	1.5E-19	-1.88	0.13	6.82E-07	-1.11	0.04	2.73E-06	-0.98	0.11	2.04E-03	-0.75	0.12	4.64E-02	-0.58	0.14	5.74E-01	-0.87	0.22	5.37E-01	-1.04	0.10	9.48E-04	-1.05	0.04	5.68E-06
ACSL5	Yes	Yes	No	Down	0.49	1.83E-19	1.5E-19	-1.08	0.11	4.82E-05	-0.96	0.04	9.09E-07	-1.10	0.11	9.25E-05	-1.28	0.13	6.11E-05	-0.87	0.15	2.16E-03	-0.90	0.22	6.76E-03	-1.22	0.10	1.16E-05	-0.90	0.03	4.54E-07
GLYATL2	Yes	Yes	No	Down	0.49	1.83E-19	1.5E-19	-1.70	0.13	4.54E-07	-1.28	0.04	1.00E-20	-1.00	0.11	2.58E-04	-0.37	0.11	1.16E-01	-0.95	0.15	3.59E-03	-1.37	0.25	1.78E-03	-0.92	0.10	1.68E-03	-0.73	0.03	8.86E-06
LRBA	Yes	Yes	No	Down	0.49	1.83E-19	0.00214	-1.14	0.11	6.93E-03	-0.81	0.04	4.05E-04	-1.18	0.11	2.10E-03	-1.31	0.14	2.05E-03	-0.78	0.15	1.12E-02	-1.66	0.27	2.35E-04	-0.85	0.10	6.80E-03	-0.60	0.03	1.82E-02
ADAM28	Yes	Yes	No	Down	0.49	1.83E-19	1.5E-19	-1.03	0.11	8.49E-04	-1.19	0.04	1.00E-20	-0.69	0.11	7.09E-03	-1.00	0.13	8.52E-04	-1.26	0.16	3.50E-02	-1.12	0.23	7.12E-02	-1.10	0.10	8.77E-05	-0.96	0.03	1.00E-20
TRAF3IP2	Yes	Yes	No	Down	0.48	1.83E-19	1.5E-19	-0.87	0.11	2.96E-03	-1.12	0.04	6.82E-07	-0.94	0.11	2.13E-03	-0.70	0.12	2.89E-02	-1.36	0.16	8.66E-05	-1.31	0.24	6.59E-04	-1.08	0.10	6.96E-04	-0.97	0.03	1.61E-05
SLC25A35	Yes	Yes	No	Down	0.48	1.83E-19	1.5E-19	-1.51	0.12	3.63E-06	-0.96	0.04	4.54E-07	-1.10	0.11	5.23E-04	-1.67	0.15	1.59E-06	-0.37	0.14	1.10E-01	-0.56	0.21	9.31E-02	-0.90	0.10	1.06E-03	-1.29	0.04	1.00E-20
NPDC1	Yes	Yes	No	Down	0.48	1.83E-19	1.5E-19	-1.72	0.13	6.82E-07	-1.44	0.04	1.00E-20	-0.93	0.11	2.16E-03	-1.46	0.14	3.01E-04	-0.21	0.14	3.51E-01	-0.22	0.20	3.69E-01	-1.17	0.10	5.95E-04	-1.23	0.04	4.54E-07
SYTL1	Yes	Yes	No	Down	0.48	1.83E-19	1.5E-19	-1.09	0.11	7.68E-04	-0.92	0.04	5.45E-06	-0.79	0.11	7.74E-03	-1.82	0.16	7.50E-06	-1.08	0.15	1.16E-02	-0.92	0.22	3.80E-02	-0.62	0.10	3.08E-02	-1.14	0.04	1.00E-20
EHF	Yes	No	No	Down	0.48	1.83E-19	0.04128	-0.79	0.11	1.43E-03	-1.01	0.04	6.82E-07	-0.58	0.11	2.54E-02	-1.12	0.13	1.28E-04	-1.19	0.16	6.95E-04	-0.82	0.22	3.47E-02	-1.79	0.11	2.27E-07	-1.10	0.04	1.00E-20
LNX1	No	Yes	No	Down	0.50	1.83E-19	6.7E-06	-1.04	0.11	3.37E-03	-0.75	0.04	5.13E-04	-1.18	0.11	2.54E-04	-0.84	0.12	1.40E-02	-1.01	0.15	2.47E-01	-1.06	0.23	2.36E-01	-0.91	0.10	3.01E-03	-1.22	0.04	1.00E-20
SLC40A1	No	Yes	No	Down	0.50	1.83E-19	1.5E-19	-1.17	0.11	6.00E-05	-1.23	0.04	1.00E-20	-1.36	0.12	3.82E-05	-0.37	0.11	1.84E-01	-1.14	0.16	3.10E-04	-0.70	0.21	2.49E-02	-1.32	0.11	8.63E-06	-0.68	0.03	5.43E-04
OVOL2	No	No	No	Down	0.50	1.83E-19	validated	-0.72	0.11	1.34E-02	-0.74	0.04	6.20E-05	-0.71	0.11	1.91E-02	-0.92	0.12	1.13E-03	-1.93	0.19	7.72E-06	-1.55	0.26	8.91E-04	-0.75	0.10	1.73E-02	-0.64	0.03	3.91E-03
PRR15	No	Yes	Yes	Down	0.50	1.83E-19	1.5E-19	-1.18	0.11	2.35E-04	-1.12	0.04	1.00E-20	-1.05	0.11	5.92E-04	-1.68	0.15	1.36E-06	-0.54	0.14	3.65E-01	-0.48	0.21	4.13E-01	-0.98	0.10	1.10E-03	-0.93	0.03	6.82E-07
CRYL1	No	Yes	No	Down	0.50	1.83E-19	1.9E-05	-0.92	0.11	2.16E-03	-1.33	0.04	1.00E-20	-1.19	0.11	6.50E-04	-0.54	0.12	9.91E-02	-0.78	0.15	2.40E-02	-1.25	0.24	4.44E-03	-0.85	0.10	4.47E-03	-1.09	0.04	3.68E-05
NDUFAF1	No	Yes	No	Down	0.50	1.83E-19	0.00722	-0.94	0.11	1.77E-02	-1.26	0.04	1.07E-05	-0.78	0.11	2.65E-02	-0.31	0.11	4.41E-01	-1.34	0.16	9.50E-03	-1.45	0.25	1.66E-02	-0.97	0.10	4.56E-03	-0.88	0.03	4.20E-03
ARPC3	No	No	No	Down	0.50	1.14E-05	validated	-0.48	0.10	2.10E-01	-0.63	0.04	1.51E-02	-1.33	0.12	1.52E-02	-1.80	0.16	9.83E-03	-0.90	0.15	1.99E-03	-1.41	0.25	3.00E-04	-0.59	0.10	5.39E-02	-0.77	0.03	1.92E-02
FOLH1	No	Yes	No	Down	0.50	1.83E-19	1.5E-19	-0.97	0.11	1.03E-03	-0.88	0.04	5.00E-06	-0.77	0.11	3.37E-03	-1.20	0.13	2.91E-04	-0.92	0.15	5.61E-02	-0.93	0.22	8.22E-02	-1.37	0.11	7.27E-06	-0.87	0.03	3.41E-06
SSR4	No	No	No	Down	0.50	1.83E-19	0.00181	-0.51	0.10	1.24E-01	-0.50	0.04	2.18E-02	-1.25	0.12	2.33E-03	-1.32	0.14	3.68E-03	-0.60	0.14	1.86E-02	-1.15	0.23	1.43E-03	-1.67	0.11	1.02E-05	-0.90	0.03	1.43E-03
ARL6IP5	No	Yes	No	Down	0.50	1.83E-19	4.6E-06	-1.40	0.12	2.37E-04	-0.80	0.04	2.86E-04	-0.88	0.11	1.37E-02	-0.41	0.12	2.07E-01	-1.22	0.16	2.31E-04	-1.10	0.23	2.40E-03	-0.93	0.10	1.67E-03	-1.16	0.04	5.34E-05
SH2D4A	No	Yes	No	Down	0.50	1.83E-19	5E-05	-0.88	0.11	4.83E-03	-0.98	0.04	1.73E-05	-1.28	0.12	1.82E-03	-0.81	0.12	1.48E-02	-1.12	0.16	1.45E-02	-1.53	0.26	5.85E-03	-0.70	0.10	6.32E-02	-0.58	0.03	1.52E-02
C9ORF116	No	Yes	No	Down	0.51	1.83E-19	0.00023	-0.90	0.11	3.07E-03	-1.01	0.04	1.36E-06	-0.91	0.11	2.64E-03	-1.63	0.15	8.93E-05	-1.20	0.16	5.48E-04	-0.89	0.22	2.26E-02	-0.59	0.10	5.41E-02	-0.74	0.03	3.18E-06
PPM1H	No	Yes	No	Down	0.51	1.83E-19	0.00296	-1.16	0.11	2.34E-04	-0.71	0.04	2.14E-04	-0.65	0.11	1.66E-02	-1.15	0.13	6.93E-04	-1.05	0.15	2.34E-03	-1.53	0.26	1.16E-03	-1.34	0.11	3.82E-05	-0.27	0.03	9.53E-02
CYP4X1	No	Yes	No	Down	0.51	1.83E-19	1.5E-19	-1.18	0.11	4.18E-05	-0.91	0.04	3.41E-06	-1.02	0.11	3.16E-04	-0.40	0.12	1.15E-01	-0.64	0.14	1.63E-02	-0.68	0.21	2.99E-02	-1.85	0.11	1.00E-20	-1.18	0.04	1.00E-20
FUK	No	Yes	No	Down	0.51	3.58E-06	0.00371	-0.86	0.11	1.85E-02	-0.83	0.04	5.60E-04	-1.39	0.12	2.38E-03	-1.94	0.16	7.30E-04	-0.62	0.14	8.95E-02	-1.05	0.23	3.34E-02	-0.18	0.10	6.06E-01	-0.99	0.03	9.67E-04
ERP29	No	Yes	No	Down	0.51	1.83E-19	0.02039	-0.07	0.10	8.54E-01	-0.71	0.04	4.94E-03	-0.46	0.10	1.58E-01	-1.03	0.13	3.97E-02	-1.11	0.16	4.06E-04	-2.40	0.34	4.54E-07	-1.31	0.11	1.36E-04	-0.73	0.03	1.49E-02
OMA1	No	Yes	No	Down	0.51	1.83E-19	0.02831	-0.86	0.11	1.66E-02	-1.19	0.04	2.95E-05	-1.01	0.11	8.26E-03	-0.82	0.12	9.01E-02	-0.69	0.15	6.06E-02	-1.50	0.26	3.96E-03	-0.68	0.10	5.12E-02	-1.07	0.04	3.68E-04
OSTF1	No	Yes	No	Down	0.51	1.83E-19	1.5E-19	-1.22	0.12	1.03E-03	-1.01	0.04	2.43E-05	-1.05	0.11	6.08E-03	-0.75	0.12	3.20E-02	-0.67	0.14	2.17E-02	-1.06	0.23	6.96E-03	-1.27	0.10	3.65E-04	-0.78	0.03	2.28E-03
RAB25	No	No	No	Down	0.51	1.83E-19	validated	-0.43	0.10	1.10E-01	-0.91	0.04	4.54E-06	-0.60	0.11	2.11E-02	-1.17	0.13	2.64E-04	-1.61	0.18	1.48E-05	-1.15	0.23	1.90E-03	-0.97	0.10	8.14E-04	-0.99	0.03	1.00E-20
TTC12	No	Yes	No	Down	0.51	1.83E-19	0.00178	-0.90	0.11	4.99E-03	-0.97	0.04	2.32E-05	-0.49	0.10	1.04E-01	-0.84	0.12	3.08E-02	-1.30	0.16	7.74E-03	-1.82	0.28	8.79E-03	-0.73	0.10	1.96E-02	-0.75	0.03	7.73E-04
SWAP70	No	Yes	No	Down	0.51	1.83E-19	4.2E-05	-1.29	0.12	8.04E-04	-0.70	0.04	7.29E-04	-0.35	0.10	2.39E-01	-1.57	0.15	2.66E-04	-0.68	0.14	3.43E-02	-1.23	0.24	7.76E-03	-0.91	0.10	1.50E-03	-1.05	0.04	4.52E-04
SEC11C	No	Yes	No	Down	0.51	1.83E-19	0.01717	-1.01	0.11	3.00E-03	-0.93	0.04	7.68E-05	-0.51	0.10	1.14E-01	-0.71	0.12	4.70E-02	-1.26	0.16	1.17E-03	-0.95	0.22	1.55E-02	-1.16	0.10	7.35E-04	-1.25	0.04	1.13E-04
TMED6	No	Yes	No	Down	0.51	1.83E-19	1.5E-19	-0.92	0.11	2.54E-04	-1.13	0.04	1.00E-20	-0.78	0.11	2.33E-03	-1.37	0.14	3.20E-05	-0.88	0.15	1.28E-02	-0.93	0.22	3.17E-02	-1.02	0.10	1.64E-04	-0.74	0.03	2.73E-06
ARFIP1	No	Yes	No	Down	0.51	1.83E-19	0.0176	-1.25	0.12	3.23E-03	-1.27	0.04	1.23E-05	-0.64	0.11	5.18E-02	-0.45	0.12	2.12E-01	-0.99	0.15	8.68E-03	-1.69	0.27	1.26E-03	-0.57	0.10	3.88E-02	-0.89	0.03	1.47E-03
CREB3L4	No	Yes	No	Down	0.51	1.83E-19	1.5E-19	-0.95	0.11	1.35E-03	-1.28	0.04	1.00E-20	-0.64	0.11	2.12E-02	-1.12	0.13	3.29E-03	-0.76	0.15	5.37E-02	-0.74	0.21	5.20E-02	-1.35	0.11	4.48E-05	-0.88	0.03	5.47E-05
ENPP3	No	Yes	No	Down	0.51	1.83E-19	1.5E-19	-1.37	0.12	2.50E-06	-1.16	0.04	1.00E-20	-0.94	0.11	2.24E-04	-1.29	0.13	1.77E-05	-0.35	0.14	1.12E-01	-1.20	0.24	2.34E-02	-0.91	0.10	2.19E-04	-0.50	0.03	9.21E-04
SLC5A1	No	Yes	No	Down	0.51	1.83E-19	1.5E-19	-0.88	0.11	1.08E-03	-1.14	0.04	1.00E-20	-0.90	0.11	3.92E-03	-1.00	0.13	4.79E-04	-1.02	0.15	9.51E-03	-1.08	0.23	2.34E-02	-0.76	0.10	6.42E-03	-0.92	0.03	2.04E-06
C19ORF24	No	Yes	No	Down	0.51	3.97E-05	0.00018	-0.76	0.11	2.85E-02	-0.30	0.04	1.14E-01	-1.39	0.12	5.31E-03	-1.27	0.13	7.55E-03	-0.40	0.14	2.14E-01	-1.58	0.26	5.32E-03	-1.41	0.11	3.95E-03	-0.58	0.03	1.47E-02
C4BPA	No	Yes	Yes	Down	0.51	1.83E-19	1.5E-19	-1.00	0.11	1.15E-04	-1.24	0.04	1.00E-20	-0.86	0.11	5.33E-04	-1.36	0.14	1.00E-05	-0.62	0.14	2.34E-02	-0.97	0.22	7.68E-03	-0.91	0.10	3.86E-04	-0.72	0.03	2.04E-06
PLA2G4A	No	Yes	No	Down	0.52	1.83E-19	1.5E-19	-0.80	0.11	1.33E-03	-0.97	0.04	9.09E-07	-1.09	0.11	1.22E-04	-0.98	0.13	1.24E-03	-0.93	0.15	1.67E-01	-1.13	0.23	2.10E-01	-1.02	0.10	8.34E-05	-0.73	0.03	2.84E-05
PIK3R1	No	Yes	No	Down	0.52	1.83E-19	1.5E-19	-0.92	0.11	2.07E-03	-1.17	0.04	4.54E-07	-1.41	0.12	2.23E-05	-0.62	0.12	3.04E-02	-0.67	0.14	1.33E-02	-0.43	0.20	1.23E-01	-1.54	0.11	1.82E-06	-0.88	0.03	4.77E-06
PPIB	No	No	No	Down	0.52	1.83E-19	4.3E-05	-1.06	0.11	5.20E-03	-0.91	0.04	1.49E-04	-1.51	0.12	7.59E-04	-1.30	0.14	5.82E-03	-0.45	0.14	5.61E-01	-0.39	0.20	6.96E-01	-1.20	0.10	1.44E-04	-0.81	0.03	9.89E-04
TSPAN8	No	Yes	No	Down	0.52	1.83E-19	1.5E-19	-1.07	0.11	3.41E-05	-1.28	0.04	1.00E-20	-1.21	0.11	1.11E-05	-0.43	0.12	6.96E-02	-0.62	0.14	2.14E-02	-1.48	0.25	3.85E-04	-0.93	0.10	1.71E-04	-0.59	0.03	8.06E-05
SLC44A3	No	Yes	No	Down	0.52	1.83E-19	0.03658	-0.68	0.11	9.52E-03	-0.92	0.04	9.31E-06	-1.32	0.12	9.97E-05	-0.74	0.12	1.20E-02	-0.84	0.15	7.50E-03	-1.50	0.26	1.04E-03	-0.90	0.10	1.07E-03	-0.73	0.03	2.43E-04
NUCB2	No	Yes	Yes	Down	0.52	1.83E-19	1.5E-19	-1.04	0.11	1.61E-03	-0.77	0.04	2.19E-04	-0.89	0.11	3.70E-03	-0.70	0.12	3.58E-02	-0.98	0.15	4.65E-03	-0.78	0.22	5.20E-02	-1.27	0.10	7.88E-05	-1.18	0.04	4.09E-06
PODXL	No	Yes	Yes	Down	0.52	1.83E-19	0.00026	-0.47	0.10	6.80E-02	-1.23	0.04	1.00E-20	-0.43	0.10	1.11E-01	-1.53	0.14	9.20E-05	-1.01	0.15	9.74E-04	-0.87	0.22	9.92E-03	-1.04	0.10	2.85E-04	-1.02	0.04	6.82E-07
SLC22A5	No	Yes	Yes	Down	0.52	1.83E-19	0.00128	-1.07	0.11	1.07E-03	-0.81	0.04	4.23E-05	-0.84	0.11	8.93E-03	-1.85	0.16	9.20E-05	-1.12	0.16	1.64E-02	-1.89	0.29	4.12E-03	-0.01	0.10	9.79E-01	-0.02	0.03	9.16E-01
ALCAM	No	Yes	No	Down	0.52	1.83E-19	2.3E-06	-1.08	0.11	5.31E-04	-0.31	0.04	5.35E-02	-1.32	0.12	2.73E-05	-0.86	0.12	1.05E-02	-1.48	0.17	1.70E-04	-1.56	0.26	3.31E-04	-0.16	0.10	4.40E-01	-0.82	0.03	1.61E-05
HDHD3	No	Yes	No	Down	0.52	1.83E-19	0.00208	-0.68	0.11	5.71E-02	-1.14	0.04	1.91E-05	-1.22	0.11	1.44E-03	-0.92	0.12	1.55E-02	-0.89	0.15	2.16E-02	-1.04	0.23	2.64E-02	-0.75	0.10	2.69E-02	-0.92	0.03	1.18E-04
C4ORF34	No	Yes	No	Down	0.52	1.83E-19	0.03277	-0.74	0.11	2.33E-02	-1.13	0.04	5.45E-06	-1.04	0.11	3.52E-03	-0.87	0.12	1.94E-02	-1.08	0.15	7.13E-04	-0.78	0.22	1.99E-02	-0.54	0.10	3.46E-02	-1.38	0.04	9.09E-07
HERPUD1	No	Yes	No	Down	0.52	1.83E-19	1.5E-19	-1.28	0.12	3.04E-04	-1.20	0.04	5.45E-06	-0.87	0.11	1.02E-02	-1.01	0.13	1.27E-02	-1.09	0.15	5.99E-04	-0.36	0.20	1.81E-01	-1.39	0.11	8.36E-05	-0.31	0.03	7.11E-02
PMM1	No	Yes	No	Down	0.52	1.83E-19	1.5E-19	-1.24	0.12	1.41E-04	-0.80	0.04	1.41E-04	-0.86	0.11	7.08E-03	-0.52	0.12	2.01E-01	-1.12	0.16	9.06E-04	-1.22	0.24	2.33E-03	-0.71	0.10	3.11E-02	-1.01	0.04	1.33E-04
SERPINA3	No	Yes	Yes	Down	0.52	1.83E-19	1.5E-19	-1.22	0.12	1.68E-05	-1.06	0.04	1.00E-20	-0.81	0.11	1.06E-03	-0.84	0.12	8.46E-04	-0.71	0.14	7.19E-03	-0.74	0.21	1.72E-02	-0.99	0.10	7.36E-05	-1.10	0.04	1.00E-20
GRHL2	No	Yes	No	Down	0.52	1.83E-19	0.0109	-1.21	0.11	6.30E-04	-0.86	0.04	9.77E-06	-0.51	0.10	7.87E-02	-1.14	0.13	2.76E-04	-1.17	0.16	1.32E-02	-0.46	0.21	1.93E-01	-1.01	0.10	3.79E-03	-1.09	0.04	6.82E-07
STXBP6	No	Yes	No	Down	0.53	1.83E-19	4.5E-05	-0.79	0.11	2.43E-03	-1.03	0.04	4.54E-07	-0.71	0.11	7.81E-03	-0.76	0.12	7.47E-03	-1.11	0.16	6.56E-04	-0.82	0.22	1.47E-02	-1.36	0.11	3.18E-06	-0.85	0.03	9.09E-07
PDIA5	No	Yes	No	Down	0.53	1.83E-19	0.00346	-0.79	0.11	9.92E-03	-1.03	0.04	8.86E-06	-1.04	0.11	2.34E-03	-1.24	0.13	2.80E-03	-0.65	0.14	2.31E-02	-1.01	0.23	7.72E-03	-1.12	0.10	4.26E-04	-0.51	0.03	3.10E-02
STEAP1	No	Yes	Yes	Down	0.53	1.83E-19	1.5E-19	-1.38	0.12	8.18E-06	-0.58	0.04	5.92E-04	-1.83	0.13	1.00E-20	-0.44	0.12	1.02E-01	-0.74	0.14	1.89E-02	-1.00	0.22	1.48E-02	-1.02	0.10	3.15E-04	-0.41	0.03	2.70E-02
CA12	No	Yes	No	Down	0.53	1.83E-19	1.5E-19	-0.75	0.11	2.89E-03	-1.02	0.04	4.54E-07	-1.12	0.11	2.16E-04	-1.23	0.13	1.09E-04	-0.55	0.14	3.35E-02	-1.00	0.22	5.09E-03	-0.83	0.10	1.89E-03	-0.85	0.03	6.82E-07
ANKRD22	No	Yes	No	Down	0.53	1.83E-19	1.5E-19	-1.36	0.12	1.11E-05	-0.89	0.04	9.31E-06	-1.16	0.11	1.80E-04	-0.62	0.12	2.66E-02	-0.72	0.14	5.82E-02	-1.13	0.23	1.82E-02	-0.82	0.10	2.04E-03	-0.66	0.03	4.71E-04
OCIAD2	No	Yes	No	Down	0.53	1.83E-19	0.00114	-0.88	0.11	1.18E-02	-0.95	0.04	3.68E-05	-0.95	0.11	9.01E-03	-1.18	0.13	9.37E-04	-1.09	0.15	4.59E-04	-0.59	0.21	4.89E-02	-0.89	0.10	3.20E-03	-0.82	0.03	2.47E-04
HOMER2	No	Yes	No	Down	0.53	1.83E-19	1.5E-19	-0.48	0.10	6.14E-02	-1.05	0.04	6.82E-07	-0.45	0.10	4.88E-02	-0.94	0.12	4.14E-03	-1.47	0.17	4.32E-05	-0.85	0.22	1.28E-02	-1.19	0.10	2.93E-05	-0.90	0.03	3.18E-06
ITGA6	No	Yes	No	Down	0.53	1.83E-19	0.00266	-1.07	0.11	1.20E-03	-1.50	0.04	1.00E-20	-0.68	0.11	2.64E-02	-1.50	0.14	2.66E-04	-0.42	0.14	2.29E-01	-0.66	0.21	1.25E-01	-0.61	0.10	3.76E-02	-0.88	0.03	1.65E-04
ISOC1	No	Yes	No	Down	0.53	1.83E-19	0.0001	-1.38	0.12	3.02E-04	-1.05	0.04	1.48E-05	-1.08	0.11	4.30E-03	-0.52	0.12	1.35E-01	-0.98	0.15	5.87E-03	-0.49	0.21	1.95E-01	-0.44	0.10	7.53E-02	-1.36	0.04	6.82E-07
MPST	No	Yes	No	Down	0.53	3.58E-06	0.00243	-1.04	0.11	7.33E-03	-0.69	0.04	1.17E-03	-0.93	0.11	1.44E-02	-1.57	0.15	2.43E-03	-1.02	0.15	6.13E-03	-0.42	0.20	1.82E-01	-0.74	0.10	4.32E-02	-0.88	0.03	4.59E-03
TMEM144	No	Yes	No	Down	0.53	1.83E-19	6.7E-06	-0.95	0.11	2.46E-03	-1.14	0.04	5.33E-04	-1.09	0.11	7.54E-04	-1.46	0.14	2.09E-04	-0.89	0.15	3.37E-02	-0.87	0.22	3.95E-02	-0.47	0.10	7.87E-02	-0.39	0.03	1.58E-02

Supplementary Table 2. Details for 2,053 genes differentially expressed between EEC and NEEC, identified by microarray meta-analysis.

EEC - endometrioid endometrial cancer, NEEC - non-endometrioid endometrial cancer, SMD - Standardised Mean Difference, FDR - False Discovery Rate, Var - Variance

Gene	145 gene list	1253 gene list	Associated with EEC-specific survival?	Up/Down Regulated in NEEC	average standardised fold change	Microarray Meta FDR	RNASeq FDR	Individual microarray study results																							
								TCGA			E-MTAB-2532			E-GEOD-56026			GSE32507			GSE24537			E-GEOD-23518			E-GEOD-17025			E-GEOD-2109		
								SMD	Var	P-value	SMD	Var	P-value	SMD	Var	P-value	SMD	Var	P-value	SMD	Var	P-value	SMD	Var	P-value	SMD	Var	P-value	SMD	Var	P-value
GJB1	No	No	No	Down	0.53	1.83E-19	validated	-0.90	0.11	8.08E-04	-1.01	0.04	4.54E-07	-0.96	0.11	1.03E-02	-1.42	0.14	9.31E-06	-0.78	0.15	5.43E-02	-1.26	0.24	1.14E-02	-0.38	0.10	2.14E-01	-0.54	0.03	1.93E-03
RUNDC1	No	No	No	Down	0.53	3.58E-06	0.00782	-1.41	0.12	4.90E-03	-1.10	0.04	3.75E-05	-0.91	0.11	1.75E-02	-0.59	0.12	1.99E-01	-0.79	0.15	4.95E-02	-1.37	0.25	1.98E-02	-0.44	0.10	1.98E-01	-0.63	0.03	6.21E-04
KIAA0247	No	No	Yes	Down	0.54	3.58E-06	validated	-1.18	0.11	4.61E-03	-0.53	0.04	2.18E-02	-0.32	0.10	2.71E-01	-0.71	0.12	9.42E-02	-0.64	0.14	6.18E-02	-1.16	0.23	1.07E-02	-1.85	0.11	6.13E-06	-0.82	0.03	3.62E-03
NUMB	No	No	No	Down	0.54	3.34E-05	validated	-0.74	0.11	5.62E-02	-0.92	0.04	1.23E-03	-0.11	0.10	6.50E-01	-2.27	0.18	3.71E-03	-1.07	0.15	2.58E-03	-1.21	0.24	4.67E-03	-0.27	0.10	2.46E-01	-0.59	0.03	1.50E-02
PLCB1	No	Yes	No	Down	0.54	1.83E-19	0.0018	-1.02	0.11	1.36E-03	-0.62	0.04	5.45E-04	-0.66	0.11	2.05E-02	-1.11	0.13	1.26E-03	-1.47	0.17	7.74E-04	-0.79	0.22	7.21E-02	-0.86	0.10	1.93E-03	-0.61	0.03	2.99E-03
GAS8	No	Yes	No	Down	0.54	1.14E-05	0.00058	-1.26	0.12	1.59E-03	-0.82	0.04	2.59E-04	-0.46	0.10	2.99E-01	-0.73	0.12	2.08E-02	-1.11	0.15	1.57E-02	-1.22	0.24	2.05E-02	-0.96	0.10	2.11E-02	-0.58	0.03	1.94E-02
LRRC8E	No	Yes	No	Down	0.54	1.83E-19	0.00227	-0.42	0.10	1.62E-01	-0.93	0.04	1.29E-05	-0.55	0.11	7.43E-02	-1.00	0.13	2.74E-03	-1.43	0.17	2.67E-02	-1.19	0.24	8.28E-02	-0.62	0.10	5.22E-02	-0.99	0.03	1.36E-06
FAM107B	No	No	No	Down	0.54	1.83E-19	0.03833	-0.69	0.11	2.00E-02	-0.93	0.04	5.45E-05	-0.66	0.11	4.59E-02	-0.26	0.11	4.11E-01	-1.66	0.18	4.66E-05	-1.36	0.25	1.08E-03	-0.88	0.10	4.84E-03	-0.69	0.03	6.77E-03
SEC16A	No	Yes	No	Down	0.54	1.66E-05	0.00122	-1.08	0.11	1.28E-02	-0.95	0.04	4.01E-04	-0.76	0.11	4.30E-02	-1.24	0.13	2.29E-02	-0.79	0.15	1.25E-02	-0.99	0.22	1.41E-02	-0.36	0.10	3.02E-01	-0.95	0.03	9.56E-03
ANAPC4	No	Yes	Yes	Down	0.54	1.83E-19	2E-05	-0.99	0.11	8.13E-04	-0.74	0.04	5.88E-05	-0.48	0.10	6.39E-02	-1.51	0.14	1.60E-04	-0.67	0.14	1.17E-02	-0.87	0.22	1.24E-02	-0.94	0.10	1.17E-03	-0.90	0.03	1.70E-05
SLC1A1	No	Yes	No	Down	0.54	1.83E-19	1.5E-19	-1.80	0.13	3.63E-06	-0.75	0.04	6.36E-05	-0.59	0.11	1.88E-02	-1.16	0.13	7.38E-04	-0.79	0.15	2.26E-01	-0.89	0.22	2.76E-01	-0.62	0.10	1.31E-02	-0.50	0.03	2.95E-03
ZNF562	No	No	No	Down	0.54	8.89E-05	validated	-0.52	0.10	2.10E-01	-0.64	0.04	1.01E-02	-0.93	0.11	1.52E-02	-0.71	0.12	9.15E-02	-1.12	0.16	6.68E-03	-1.58	0.26	2.25E-03	-0.96	0.10	2.17E-02	-0.64	0.03	4.68E-02
FUT2	No	Yes	No	Down	0.54	1.83E-19	0.00011	-0.50	0.10	8.98E-02	-0.90	0.04	1.32E-05	-1.23	0.11	1.89E-04	-0.91	0.12	1.24E-03	-1.08	0.15	1.11E-02	-1.16	0.23	3.34E-02	-0.58	0.10	7.79E-02	-0.74	0.03	9.31E-05
C1ORF168	No	No	No	Down	0.54	1.83E-19	validated	-0.30	0.10	2.14E-01	-0.93	0.04	3.86E-06	-0.93	0.11	7.51E-04	-1.26	0.13	6.29E-05	-0.38	0.14	3.89E-01	-1.64	0.27	3.97E-02	-1.01	0.10	1.67E-04	-0.64	0.03	2.22E-04
CA8	No	Yes	No	Down	0.54	1.83E-19	1.5E-19	-0.45	0.10	4.92E-02	-1.30	0.04	1.00E-20	-0.59	0.11	2.18E-02	-0.53	0.12	3.96E-02	-1.41	0.17	1.06E-03	-1.50	0.26	3.65E-03	-0.39	0.10	1.61E-01	-0.90	0.03	2.04E-06
B4GALT4	No	Yes	No	Down	0.54	3.58E-06	0.0056	-0.71	0.11	4.33E-02	-0.67	0.04	2.84E-03	-0.93	0.11	5.82E-03	-0.38	0.12	2.85E-01	-1.31	0.16	2.81E-03	-1.31	0.24	6.87E-03	-1.02	0.10	2.53E-03	-0.74	0.03	2.33E-03
CCNO	No	Yes	No	Down	0.54	1.83E-19	4.1E-05	-1.26	0.12	1.24E-04	-1.24	0.04	1.00E-20	-0.73	0.11	9.88E-03	-0.44	0.12	1.82E-01	-1.03	0.15	3.65E-03	-0.71	0.21	1.21E-01	-0.78	0.10	6.67E-03	-0.88	0.03	1.39E-05
PDZD8	No	Yes	No	Down	0.54	1.83E-19	0.00404	-1.21	0.11	3.62E-04	-0.53	0.04	5.68E-03	-0.88	0.11	5.10E-03	-1.47	0.14	3.19E-04	-0.32	0.14	1.98E-01	-0.85	0.22	3.99E-02	-0.93	0.10	1.00E-03	-0.87	0.03	3.26E-04
C17ORF28	No	No	No	Down	0.54	1.83E-19	validated	-0.56	0.10	1.28E-01	-0.87	0.04	3.50E-05	-0.28	0.10	4.19E-01	-2.13	0.17	5.23E-06	-1.16	0.16	5.07E-03	-1.29	0.24	6.20E-03	-0.19	0.10	6.00E-01	-0.58	0.03	1.17E-03
REEP5	No	No	No	Down	0.54	6.71E-06	0.04094	-1.10	0.11	1.03E-02	-0.13	0.04	4.91E-01	-1.59	0.12	4.86E-04	-1.48	0.14	3.89E-03	-0.51	0.14	4.37E-02	-0.94	0.22	6.86E-03	-0.06	0.10	8.37E-01	-1.22	0.04	7.09E-05
KCNK6	No	Yes	No	Down	0.54	1.83E-19	1.5E-19	-1.36	0.12	2.43E-04	-1.32	0.04	1.00E-20	-0.94	0.11	1.98E-03	-0.40	0.12	1.64E-01	-0.78	0.15	3.15E-02	-0.68	0.21	8.16E-02	-0.78	0.10	2.31E-02	-0.77	0.03	8.72E-04
LRRC46	No	Yes	No	Down	0.54	1.83E-19	1.5E-19	-1.19	0.11	6.88E-05	-0.89	0.04	2.04E-06	-0.76	0.11	2.80E-03	-1.13	0.13	1.00E-03	-0.83	0.15	2.03E-02	-0.74	0.21	1.68E-01	-0.86	0.10	1.53E-03	-0.62	0.03	1.39E-04
GYLTL1B	No	No	No	Down	0.54	1.83E-19	0.0092	-0.63	0.11	2.93E-02	-0.88	0.04	1.61E-05	-0.64	0.11	3.01E-02	-2.13	0.17	2.50E-06	-1.05	0.15	2.46E-02	-0.92	0.22	4.52E-02	-0.24	0.10	3.90E-01	-0.52	0.03	1.36E-02
SLC11A2	No	Yes	Yes	Down	0.54	1.83E-19	1.3E-05	-0.53	0.10	8.93E-02	-0.78	0.04	2.54E-04	-0.86	0.11	1.01E-02	-0.75	0.12	4.63E-02	-1.10	0.15	4.99E-04	-1.30	0.24	6.78E-04	-0.75	0.10	1.25E-02	-0.93	0.03	1.87E-04
C1ORF64	No	Yes	Yes	Down	0.54	1.83E-19	1.5E-19	-1.54	0.12	4.54E-07	-1.19	0.04	1.00E-20	-0.78	0.11	6.20E-03	-0.89	0.12	5.31E-04	-0.73	0.14	8.47E-03	-0.76	0.21	4.87E-02	-0.49	0.10	1.04E-01	-0.63	0.03	2.42E-04
MSX1	No	Yes	Yes	Down	0.55	1.83E-19	1.5E-19	-0.74	0.11	1.69E-03	-0.86	0.04	1.36E-06	-0.47	0.10	3.02E-02	-0.82	0.12	3.02E-03	-0.98	0.15	8.54E-04	-1.34	0.25	3.21E-04	-0.94	0.10	2.13E-04	-0.84	0.03	1.00E-20
BACE2	No	Yes	Yes	Down	0.55	1.83E-19	6.4E-05	-1.33	0.12	4.63E-05	-0.98	0.04	5.45E-06	-0.54	0.11	5.53E-02	-0.73	0.12	2.82E-02	-0.91	0.15	1.42E-02	-1.20	0.24	7.25E-03	-0.44	0.10	8.46E-02	-0.87	0.03	7.22E-05
WDR78	No	Yes	No	Down	0.55	1.83E-19	0.00242	-0.71	0.11	7.00E-03	-0.80	0.04	2.41E-05	-0.80	0.11	3.80E-03	-1.19	0.13	7.66E-04	-1.06	0.15	9.08E-02	-0.42	0.20	6.04E-01	-1.19	0.10	5.29E-05	-0.84	0.03	2.04E-06
SCGB1D2	No	Yes	No	Down	0.55	1.83E-19	2E-05	-1.04	0.11	5.36E-05	-0.89	0.04	4.54E-07	-1.01	0.11	8.43E-05	-0.93	0.12	4.14E-04	-0.73	0.14	6.64E-03	-0.62	0.21	4.85E-02	-0.78	0.10	1.07E-03	-0.99	0.03	1.00E-20
SDF2L1	No	Yes	No	Down	0.55	3.58E-06	0.00346	-0.61	0.10	5.96E-02	-0.48	0.04	1.75E-02	-0.55	0.11	9.99E-02	-1.61	0.15	7.82E-04	-0.52	0.14	3.83E-02	-1.21	0.24	1.37E-03	-1.60	0.11	6.47E-05	-0.40	0.03	8.71E-02
AGR3	No	Yes	No	Down	0.55	1.83E-19	0.00179	-0.76	0.11	1.50E-03	-0.88	0.04	1.36E-06	-0.66	0.11	4.81E-03	-0.91	0.12	1.30E-03	-0.96	0.15	1.95E-03	-1.09	0.23	8.76E-03	-0.90	0.10	3.36E-04	-0.81	0.03	1.00E-20
F11R	No	No	No	Down	0.55	1.83E-19	validated	-0.11	0.10	7.53E-01	-0.85	0.04	5.43E-05	-0.49	0.10	1.32E-01	-1.48	0.14	1.17E-04	-0.84	0.15	1.68E-02	-1.80	0.28	1.57E-03	-0.75	0.10	1.33E-02	-0.67	0.03	3.43E-04
LAMC2	No	No	No	Down	0.55	1.83E-19	0.03406	-0.57	0.10	3.57E-02	-0.63	0.04	1.89E-04	-0.47	0.10	6.56E-02	-1.26	0.13	7.75E-05	-1.37	0.16	1.73E-04	-1.34	0.24	1.01E-03	-0.45	0.10	7.24E-02	-0.88	0.03	2.04E-06
PDLIM1	No	Yes	Yes	Down	0.55	1.83E-19	0.00033	-0.57	0.10	5.07E-02	-0.98	0.04	5.00E-06	-1.01	0.11	2.64E-03	-0.44	0.12	1.33E-01	-1.14	0.16	4.44E-04	-1.29	0.24	9.52E-04	-0.24	0.10	3.08E-01	-1.28	0.04	1.00E-20
FKBP11	No	Yes	No	Down	0.55	1.83E-19	1.5E-19	-1.40	0.12	2.73E-05	-0.73	0.04	2.38E-04	-0.73	0.11	1.09E-02	-1.10	0.13	2.63E-03	-0.87	0.15	3.24E-03	-0.73	0.21	3.40E-02	-0.73	0.10	1.47E-02	-0.65	0.03	9.83E-03
CPM	No	Yes	Yes	Down	0.55	1.83E-19	0.00011	-0.92	0.11	2.81E-04	-0.68	0.04	3.79E-05	-0.92	0.11	3.65E-04	-0.69	0.12	1.48E-02	-0.84	0.15	2.74E-02	-1.32	0.24	4.58E-03	-0.78	0.10	1.20E-03	-0.76	0.03	2.04E-06
COBL	No	No	No	Down	0.55	1.83E-19	0.02421	-0.68	0.11	6.23E-02	-1.01	0.04	3.18E-06	-0.31	0.10	1.64E-01	-1.41	0.14	6.20E-05	-1.02	0.15	2.57E-03	-0.91	0.22	1.76E-02	-0.92	0.10	1.86E-03	-0.64	0.03	6.27E-05
TMEM101	No	Yes	No	Down	0.55	1.83E-19	6E-05	-0.94	0.11	1.83E-04	-0.92	0.04	6.82E-07	-0.71	0.11	5.84E-03	-1.16	0.13	1.33E-04	-0.80	0.15	4.40E-03	-1.03	0.23	3.24E-03	-0.51	0.10	2.63E-02	-0.83	0.03	3.86E-06
PLCH1	No	Yes	No	Down	0.55	6.71E-06	0.02243	-1.14	0.11	2.67E-03	-0.72	0.04	3.27E-04	-0.71	0.11	1.60E-02	-1.04	0.13	1.72E-03	-0.75	0.14	1.58E-01	-1.48	0.25	1.07E-01	-0.62	0.10	3.78E-02	-0.44	0.03	8.00E-03
SLC15A2	No	Yes	No	Down	0.55	1.83E-19	1.5E-19	-0.18	0.10	4.67E-01	-0.65	0.04	3.76E-03	-1.21	0.11	5.04E-05	-0.47	0.12	1.14E-01	-0.98	0.15	2.20E-02	-1.50	0.26	2.69E-02	-1.26	0.10	7.70E-05	-0.63	0.03	3.73E-04
KCNN4	No	Yes	No	Down	0.55	1.83E-19	2.3E-06	-1.04	0.11	1.05E-04	-0.99	0.04	6.82E-07	-0.40	0.10	9.87E-02	-1.28	0.13	2.25E-04	-0.91	0.15	6.29E-02	-1.10	0.23	8.15E-02	-0.68	0.10	1.84E-02	-0.48	0.03	3.94E-03
NUDT18	No	Yes	No	Down	0.55	1.14E-05	1.5E-19	-1.17	0.11	5.41E-04	-0.79	0.04	3.97E-04	-1.31	0.12	8.54E-03	-0.58	0.12	9.52E-02	-1.00	0.15	1.26E-02	-0.99	0.22	3.37E-02	-0.79	0.10	5.36E-02	-0.25	0.03	2.14E-01
ATG4A	No	Yes	No	Down	0.55	0.000805	0.00424	-0.42	0.10	2.31E-01	-0.99	0.04	1.05E-03	-0.96	0.11	1.90E-02	-0.75	0.12	1.24E-01	-0.77	0.15	8.16E-02	-1.77	0.28	7.87E-03	-0.77	0.10	5.49E-02	-0.44	0.03	2.04E-01
PAM	No	Yes	No	Down	0.55	1.83E-19	0.0003	-0.80	0.11	6.55E-03	-0.59	0.04	8.46E-04	-0.52	0.11	5.56E-02	-0.58	0.12	4.68E-02	-1.53	0.17	1.34E-05	-1.02	0.23	3.19E-03	-1.10	0.10	2.28E-04	-0.71	0.03	1.39E-04
GAD1	No	Yes	No	Down	0.55	1.83E-19	1.5E-19	-1.11	0.11	6.00E-05	-0.67	0.04	4.29E-05	-0.86	0.11	5.90E-04	-0.45	0.12	4.83E-02	-0.89	0.15	4.89E-03	-1.05	0.23	5.58E-03	-1.23	0.10	4.77E-06	-0.59	0.03	8.68E-05
GABRP	No	No	No	Down	0.55	1.83E-19	validated	-0.86	0.11	1.54E-03	-0.78	0.04	4.32E-06	-0.72	0.11	2.55E-03	-1.09	0.13	4.25E-04	-0.61	0.14	1.95E-02	-1.10	0.23	2.51E-03	-0.77	0.10	9.67E-04	-0.90	0.03	1.00E-20
PHKB	No	Yes	No	Down	0.55	1.83E-19	1.5E-19	-1.01	0.11	5.04E-03	-1.43	0.04	3.18E-06	-0.10	0.10	7.27E-01	-0.89	0.12	5.04E-02	-0.96	0.15	4.55E-03	-1.25	0.24	1.16E-02	-0.61	0.10	2.08E-02	-0.60	0.03	4.86E-03
KLHL2	No	Yes	No	Down	0.55	2.40E-05	0.00099	-1.11	0.11	8.80E-04	-0.64	0.04	4.38E-03	-0.65	0.11	5.49E-02	-0.48	0.12	1.20E-01	-1.43	0.17	6.19E-02	-1.02	0.23	1.74E-01	-0.33	0.10	2.46E-01	-1.17	0.04	4.91E-05
MRPL54	No	Yes	No	Down	0.55	6.71E-06	6.7E-06	-1.16	0.11	4.13E-03	-0.98	0.04	4.06E-04	-1.15	0.11	4.01E-03	-0.51	0.12	1.87E-01	-0.10	0.14	5.42E-01	-0.81	0.22	2.70E-02	-1.16	0.10	7.14E-04	-0.96	0.03	6.67E-03
SLC26A2	No	Yes	No	Down	0.55	1.83E-19	1.5E-19	-1.48	0.12	1.00E-05	-0.59	0.04	9.30E-04	-0.71	0.11	6.77E-03	-1.44	0.14	7.84E-05	-0.74	0.14	3.17E-01	-0.11	0.20	8.78E-01	-0.93	0.10	3.94E-04	-0.84	0.03	1.20E-05
MANSC1	No	Yes	No	Down	0.55	1.83E-19	1.5E-19	-0.63	0.11	1.79E-02	-1.09	0.04	6.82E-07	-1.14	0.11	7.49E-04	-0.17	0.11	5.19E-01	-1.18	0.16	9.60E-04	-1.23	0.24	4.40E-03	-0.48	0.10	9.45E-02	-0.91	0.03	7.95E-05
SQRDL	No	Yes	No	Down	0.55	1.83E-19	1.5E-19	-0.89	0.11	3.63E-03	-1.28	0.04	1.00E-20	-1.05	0.11	1.09E-03	-0.66	0.12	4.53E-02	-0.43	0.14	1.80E-01	-0.95	0.22	5.18E-02	-0.67	0.10	1.24E-02	-0.89	0.03	1.57E-04
GALNT7	No	Yes	No	Down	0.55	1.83E-19	0.00028	-1.18	0.11	2.13E-04	-1.23	0.04	6.82E-07	-0.88	0.11	5.30E-03	-1.34	0.14	5.31E-04	-0.05	0.14	9.73E-01	-0.16	0.20	9.26E-01	-0.84	0.10	4.31E-03	-1.12	0.04	2.73E-06
SLC6A14	No	Yes	No	Down	0.55	1.83E-19	1.5E-19	-1.07	0.11	1.07E-04	-1.01	0.04	4.54E-07	-0.71	0.11	3.12E-03	-0.48	0.12	4.89E-02	-0.59	0.14	3.17E-01	-1.21	0.24	1.38E-01	-0.91	0.10	3.44E-04	-0.83	0.03	6.82E-07
FOXJ1	No	No	No	Down	0.56	1.83E-19	validated	-0.72	0.11	1.93E-02	-0.69	0.04	2.41E-05	-0.68	0.11	2.38E-02	-1.95	0.16	2.27E-07	-0.84	0.15	8.07E-03	-0.94	0.22	1.65E-02	-0.12	0.10	6.83E-01	-0.85	0.03	1.14E-06
GOLM1	No	Yes	No	Down	0.56	1.83E-19	2.3E-06	-0.56	0.10	7.56E-02	-0.86	0.04	1.73E-05	-1.12	0.11	1.20E-03	-0.39	0.12	1.65E-01	-1.36	0.16	5.70E-04	-0.74	0.21	5.77E-02	-0.84	0.10	3.35E-03	-0.92	0.03	1.02E-05
CDH1	No	No	No	Down	0.56	1.83E-19	validated	-0.89	0.11	8.25E-03	-0.78	0.04	1.64E-05	-0.48	0.10	1.02E-01	-1.29	0.14	3.79E-05	-1.14	0.16	3.27E-04	-0.84	0.22	1.10E-02	-0.80	0.10	5.61E-03	-0.56	0.03	7.36E-04
PEX11G	No	Yes	No	Down	0.56	0.000108	1.5E-19	-0.60	0.10	3.36E-02	-0.78	0.04	1.70E-03	-0.90	0.11	3.90E-02	-0.78	0.12	4.79E-02	-1.31	0.16	1.64E-01	-0.85	0.22	3.41E-01	-0.79	0.10	8.15E-02	-0.76	0.03	1.52E-04
RPS27L	No	Yes	No	Down	0.56	1.83E-19	1.5E-19	-1.80	0.13	2.20E-05	-1.06	0.04	1.66E-05	-0.46	0.10	8.28E-02	-0.06	0.11	8.50E-01	-0.77	0.15	6.09E-03	-1.16	0.23	1.34E-03	-0.48	0.10	7.84E-02	-0.97	0.03	1.70E-05
SPG11	No	Yes	No	Down	0.56	6.88E-05	0.00223	-0.88	0.11	3.76E-02	-0.72	0.04	4.62E-03	-0.91	0.11	2.23E-02	-1.47	0.14	5.49E-03	-0.98	0.15	5.83E-03	-0.77	0.21	3.78E-02	-0.81	0.10	3.33E-02	-0.23	0.03	1.83E-01
PEX1	No	Yes	No	Down	0.56	0.000148	0.03325	-1.19	0.11	2.04E-03	-0.80	0.04	1.41E-03	-0.34	0.10	3.10E-01	-0.75	0.12	1.75E-01	-1.04	0.15	7.54E-02	-0.95	0.22	1.08E-01	-0.95	0.10	1.12E-02	-0.73	0.03	8.21E-03
TRIM2	No	Yes	No	Down	0.56	1.83E-19	0.00059	-1.76	0.13	9.31E-06	-0.85	0.04	3.04E-05	-0.57	0.11	2.28E-02	-0.37	0.11	1.63E-01	-0.86	0.15	9.68E-03	-0.95	0.22	1.15E-02	-0.84	0.10	2.47E-03	-0.53	0.03	1.13E-03
CDKN1A	No	Yes	No	Down	0.56	1.83E-19	1.5E-19	-1.59	0.12	1.45E-05	-1.03	0.04	2.73E-06	-0.89	0.11	4.84E-03	-0.38	0.11	2.19E-01	-1.06	0.15	6.74E-04	-0.92	0.22	6.43E-03	-0.84	0.10	8.84E-03	-0.03	0.03	8.05E-01
CASC1	No	Yes	No	Down	0.56	1.83E-19	0.01383	-0.76	0.11	3.27E-03	-0.84	0.04	1.11E-05	-0.46	0.10	5.20E-02	-1.18	0.13	4.43E-04	-1.14	0.16	1.47E-03	-0.87	0.22	6.12E-02	-0.79	0.10	2.79E-03	-0.69	0.03	3.04E-05
ADAMTS19	No	Yes	No	Down	0.56	1.83E-19	1.5E-19	-1.37	0.12	4.32E-06	-0.72	0.04	1.68E-04	-0.55	0.11	2.60E-02	-0.59	0.12	2.23E-02	-0.76	0.15	6.70E-02	-1.09	0.23	3.99E-02	-0.77	0.10	2.05E-03	-0.86	0.03	1.00E-20
PLA2G10	No	Yes	No	Down	0.56	1.83E-19	6E-05	-0.79	0.11	4.60E-03	-0.78	0.04	2.36E-05	-1.02	0.11	6.78E-03	-0.77	0.12	7.50E-03	-0.84	0.15	1.30E-02	-1.10	0.23	9.46E-03	-0.95	0.10	8.91E-03	-0.48	0.03	8.56E-03
FZD10	No	Yes	Yes	Down	0.56	1.83E-19	5.2E-05	-1.53	0.12	4.54E-07	-0.55	0.04	4.81E-04	-0.77	0.11	2.95E-03	-0.56	0.12	2.96E-02	-0.97	0.15	2.18E-01	-0.89	0.22	3.53E-01	-0.83	0.10	1.15E-03	-0.62	0.03	1.65E-04
HMGCL	No	Yes	No	Down	0.56	0.000125	0.00064	-0.58	0.10	7.34E-02	-0.68	0.04	2.98E-03	-0.65	0.11	7.42E-02	-0.24	0.11	5.65E-01	-1.31	0.16	1.65E-02	-1.42	0.25	1.57E-02	-0.59	0.10	7.20E-02	-1.24	0.04	5.46E-04
FAM108A1	No	Yes	No	Down	0.56	0.002356	0.0004	-0.79	0.11	2.10E-02	-0.45	0.04	4.03E-02	-1.01	0.11	2.41E-02	-1.37	0.14	2.13E-02	-0.20	0.14	8.10E-01	-0.99	0.22	6.63E-01	-1.48	0.11	1.82E-03	-0.41	0.03	1.05E-01

Supplementary Table 2. Details for 2,053 genes differentially expressed between EEC and NEEC, identified by microarray meta-analysis.

EEC - endometrioid endometrial cancer, NEEC - non-endometrioid endometrial cancer, SMD - Standardised Mean Difference, FDR - False Discovery Rate, Var - Variance

Gene	145 gene list	1253 gene list	Associated with EEC-specific survival?	Up/Down Regulated in NEEC	average standardised fold change	Microarray Meta FDR	RNASeq FDR	Individual microarray study results: TCGA SMD	TCGA Var	TCGA P-value	E-MTAB-2532 SMD	E-MTAB-2532 Var	E-MTAB-2532 P-value	E-GEOD-56026 SMD	E-GEOD-56026 Var	E-GEOD-56026 P-value	GSE32507 SMD	GSE32507 Var	GSE32507 P-value	GSE24537 SMD	GSE24537 Var	GSE24537 P-value	E-GEOD-23518 SMD	E-GEOD-23518 Var	E-GEOD-23518 P-value	E-GEOD-17025 SMD	E-GEOD-17025 Var	E-GEOD-17025 P-value	E-GEOD-2109 SMD	E-GEOD-2109 Var	E-GEOD-2109 P-value
NUDT9	No	No	No	Down	0.56	0.000637	0.03844	-1.24	0.12	5.65E-03	-0.08	0.04	6.79E-01	-0.16	0.10	6.33E-01	-1.17	0.13	2.57E-02	-1.03	0.15	6.02E-03	-2.01	0.30	4.55E-04	-0.29	0.10	3.50E-01	-0.72	0.03	3.73E-02
GSTZ1	No	Yes	No	Down	0.56	2.19E-05	0.00053	-1.00	0.11	3.13E-03	-0.49	0.04	1.49E-02	-0.63	0.11	5.44E-02	-0.78	0.12	7.15E-02	-0.90	0.15	6.16E-02	-1.13	0.23	5.37E-02	-0.83	0.10	3.33E-02	-0.94	0.03	1.55E-04
NQO1	No	Yes	No	Down	0.56	1.83E-19	1.5E-19	-1.21	0.11	2.50E-05	-0.65	0.04	1.59E-04	-1.24	0.12	3.98E-05	-0.49	0.12	4.99E-02	-0.34	0.14	1.27E-01	-0.91	0.22	8.47E-03	-0.78	0.10	1.88E-03	-1.06	0.04	1.00E-20
PKHD1L1	No	Yes	No	Down	0.56	1.83E-19	1.5E-19	-0.67	0.11	6.94E-03	-0.82	0.04	3.18E-06	-0.97	0.11	1.30E-04	-0.46	0.12	4.67E-02	-0.92	0.15	5.64E-03	-0.88	0.22	2.38E-02	-0.98	0.10	8.97E-05	-0.97	0.03	1.00E-20
SLC27A2	No	Yes	No	Down	0.56	1.83E-19	0.00025	-0.63	0.10	2.11E-02	-1.11	0.04	6.82E-07	-0.60	0.11	2.01E-02	-0.91	0.12	2.88E-03	-0.91	0.15	1.69E-02	-0.65	0.21	1.04E-01	-1.04	0.10	5.39E-04	-0.81	0.03	2.25E-05
TMPRSS13	No	Yes	No	Down	0.56	1.83E-19	1.5E-19	-1.04	0.11	1.51E-03	-0.78	0.04	4.93E-05	-0.64	0.11	3.01E-02	-0.56	0.12	3.17E-02	-1.10	0.15	2.99E-02	-1.01	0.23	5.95E-02	-0.99	0.10	5.10E-03	-0.53	0.03	8.36E-04
ELL3	No	Yes	No	Down	0.56	1.83E-19	0.00016	-0.78	0.11	9.52E-03	-1.11	0.04	1.59E-06	-1.06	0.11	2.69E-03	-1.06	0.13	1.98E-03	-0.63	0.14	1.81E-01	-0.71	0.21	1.87E-01	-0.74	0.10	1.53E-02	-0.54	0.03	2.46E-02
C1ORF88	No	Yes	No	Down	0.56	1.83E-19	0.00388	-0.76	0.11	4.65E-03	-0.90	0.04	9.09E-07	-0.55	0.11	2.30E-02	-1.29	0.13	9.20E-05	-0.94	0.15	7.82E-02	-0.54	0.21	3.42E-01	-0.80	0.10	1.75E-03	-0.84	0.03	1.59E-06
CADPS2	No	Yes	No	Down	0.56	1.83E-19	4.1E-05	-0.83	0.11	3.98E-03	-1.22	0.04	1.36E-06	-0.97	0.11	1.12E-03	-0.36	0.11	1.65E-01	-0.21	0.14	2.83E-01	-1.23	0.24	9.30E-03	-1.14	0.10	1.76E-04	-0.68	0.03	1.79E-03
TMEM2	No	Yes	No	Down	0.56	1.83E-19	6.7E-06	-1.33	0.12	8.95E-05	-0.32	0.04	5.37E-02	-0.75	0.11	2.78E-02	-0.82	0.12	2.67E-02	-0.78	0.15	1.26E-02	-1.71	0.27	1.12E-04	-0.29	0.10	2.56E-01	-0.63	0.03	1.24E-03
P2RX4	No	Yes	Yes	Down	0.56	1.83E-19	0.00037	-0.75	0.11	1.68E-02	-0.86	0.04	1.21E-04	-0.66	0.11	3.66E-02	-0.54	0.12	1.35E-01	-1.05	0.15	3.64E-03	-1.01	0.23	1.52E-02	-1.04	0.10	4.25E-03	-0.72	0.03	3.34E-03
SH3RF2	No	Yes	No	Down	0.56	1.83E-19	7.6E-05	-0.84	0.11	6.14E-03	-0.66	0.04	4.48E-03	-1.21	0.11	1.54E-04	-1.04	0.13	3.75E-03	-0.69	0.14	5.50E-02	-0.65	0.21	3.20E-01	-0.77	0.10	3.81E-03	-0.76	0.03	8.86E-06
SFN	No	Yes	No	Down	0.56	1.83E-19	1.5E-19	-1.10	0.11	1.91E-04	-0.71	0.04	6.36E-05	-0.71	0.11	5.78E-03	-1.15	0.13	9.70E-04	-0.47	0.14	5.37E-02	-0.74	0.21	1.96E-02	-0.51	0.10	4.43E-02	-1.20	0.04	1.00E-20
CHST6	No	Yes	No	Down	0.56	1.83E-19	1.5E-19	-0.44	0.10	6.03E-02	-0.77	0.04	1.00E-05	-1.03	0.11	8.03E-04	-1.08	0.13	5.05E-04	-0.91	0.15	2.18E-02	-0.58	0.21	1.90E-01	-1.12	0.10	1.90E-04	-0.68	0.03	5.45E-06
RAB17	No	No	No	Down	0.56	1.83E-19	validated	-0.34	0.10	2.53E-01	-0.75	0.04	2.08E-04	-0.39	0.10	1.91E-01	-0.95	0.13	2.22E-03	-1.40	0.17	1.24E-04	-1.13	0.23	3.59E-03	-0.96	0.10	1.04E-02	-0.68	0.03	6.60E-04
ELF5	No	Yes	No	Down	0.56	1.83E-19	1.5E-19	-1.23	0.12	1.27E-04	-0.83	0.04	2.61E-05	-1.00	0.11	4.10E-04	-0.33	0.11	1.63E-01	-0.90	0.15	3.09E-02	-0.81	0.22	2.21E-02	-0.98	0.10	2.08E-04	-0.50	0.03	2.47E-03
KLK11	No	No	No	Down	0.57	1.83E-19	validated	-1.43	0.12	6.82E-07	-0.99	0.04	1.00E-20	-1.07	0.11	1.72E-04	-1.02	0.13	2.78E-04	-0.75	0.14	5.83E-03	-0.24	0.20	2.80E-01	-0.39	0.10	8.88E-02	-0.69	0.03	1.59E-05
TSPAN13	No	Yes	No	Down	0.57	1.83E-19	0.00024	-1.12	0.11	3.85E-04	-0.73	0.04	8.25E-05	-1.20	0.11	1.40E-03	-0.15	0.11	5.86E-01	-1.15	0.16	4.29E-04	-0.81	0.22	1.98E-02	-0.52	0.10	1.14E-01	-0.90	0.03	4.91E-05
MKNK2	No	Yes	No	Down	0.57	1.83E-19	3.1E-05	-1.01	0.11	8.04E-03	-0.59	0.04	2.52E-03	-1.33	0.12	9.28E-04	-0.58	0.12	9.24E-02	-0.70	0.14	1.34E-02	-0.74	0.21	2.40E-02	-0.26	0.10	3.80E-01	-1.38	0.04	1.36E-06
CHMP2A	No	No	No	Down	0.57	1.14E-05	0.03166	-0.52	0.10	1.58E-01	-0.79	0.04	2.34E-03	-0.78	0.11	3.50E-02	-0.34	0.11	4.52E-01	-0.95	0.15	3.30E-03	-1.23	0.24	3.53E-03	-0.96	0.10	2.09E-03	-0.98	0.03	4.30E-03
MAPK6	No	Yes	No	Down	0.57	1.14E-05	0.00453	-0.55	0.10	1.02E-01	-0.74	0.04	1.52E-03	-1.36	0.12	2.92E-03	-0.14	0.11	7.30E-01	-1.44	0.17	2.91E-04	-0.60	0.21	7.30E-02	-0.89	0.10	8.18E-03	-0.81	0.03	1.49E-02
GCNT1	No	Yes	No	Down	0.57	1.83E-19	1.5E-19	-0.42	0.10	9.07E-02	-1.24	0.04	1.00E-20	-0.58	0.11	3.64E-02	-0.75	0.12	1.21E-02	-0.79	0.15	6.60E-03	-0.94	0.22	7.24E-03	-0.87	0.10	1.92E-03	-0.96	0.03	1.98E-05
NT5E	No	Yes	No	Down	0.57	1.83E-19	1.5E-19	-1.33	0.12	1.68E-05	-1.08	0.04	1.00E-20	-0.66	0.11	7.28E-03	-0.19	0.11	4.16E-01	-0.72	0.14	2.01E-02	-0.87	0.22	2.08E-02	-0.88	0.10	7.97E-04	-0.82	0.03	9.31E-06
LSM7	No	Yes	No	Down	0.57	1.14E-05	1.5E-19	-1.10	0.11	8.41E-03	-0.55	0.04	2.00E-02	-0.76	0.11	3.10E-02	-1.18	0.13	3.15E-02	-0.54	0.14	4.69E-02	-0.42	0.20	1.44E-01	-1.50	0.11	2.27E-05	-0.48	0.03	6.23E-02
ICAM3	No	Yes	No	Down	0.57	6.71E-06	0.0057	-0.38	0.10	2.08E-01	-0.44	0.04	2.04E-02	-1.52	0.12	5.68E-04	-0.75	0.12	7.40E-02	-1.08	0.15	4.90E-03	-0.60	0.21	9.54E-02	-0.90	0.10	6.07E-03	-0.86	0.03	4.17E-04
LRG1	No	No	No	Down	0.57	1.83E-19	validated	-0.59	0.10	1.21E-02	-0.99	0.04	6.82E-07	-0.69	0.11	9.50E-03	-1.29	0.14	9.20E-05	-0.82	0.15	8.34E-03	-0.80	0.22	2.79E-02	-0.60	0.10	3.47E-02	-0.74	0.03	3.98E-05
CYBA	No	No	No	Down	0.57	1.83E-19	validated	-0.38	0.10	1.61E-01	-0.84	0.04	5.86E-05	-1.17	0.11	7.45E-04	-1.66	0.15	1.96E-04	-0.81	0.15	3.61E-03	-0.36	0.20	1.53E-01	-1.25	0.10	1.22E-04	-0.04	0.03	8.08E-01
MOGAT1	No	Yes	No	Down	0.57	1.83E-19	0.04371	-0.93	0.11	5.67E-04	-0.70	0.04	3.43E-05	-0.80	0.11	5.94E-03	-1.71	0.15	4.54E-07	-0.39	0.14	2.62E-01	-0.72	0.21	3.05E-01	-0.49	0.10	3.08E-02	-0.77	0.03	9.09E-06
RBBP7	No	Yes	No	Down	0.57	1.83E-19	5.4E-05	-0.92	0.11	4.04E-03	-0.90	0.04	1.06E-04	-0.79	0.11	2.21E-02	-0.44	0.12	1.78E-01	-1.00	0.15	3.94E-03	-0.86	0.22	2.67E-02	-0.83	0.10	7.35E-03	-0.76	0.03	1.73E-03
PAQR4	No	Yes	No	Down	0.57	1.14E-05	1.5E-19	-0.78	0.11	6.36E-02	-0.61	0.04	1.64E-03	-0.81	0.11	3.78E-02	-0.28	0.11	3.96E-01	-1.44	0.17	2.36E-04	-1.12	0.23	1.34E-02	-0.91	0.10	1.88E-02	-0.54	0.03	2.01E-02
PIAS3	No	Yes	Yes	Down	0.57	8.12E-05	0.00722	-0.78	0.11	3.30E-02	-0.52	0.04	9.98E-03	-0.84	0.11	1.50E-02	-1.05	0.13	1.22E-02	-0.65	0.14	9.26E-02	-1.24	0.24	2.64E-02	-0.59	0.10	6.81E-02	-0.81	0.03	3.43E-03
GALNT10	No	Yes	Yes	Down	0.57	1.83E-19	1.5E-19	-0.87	0.11	1.50E-02	-0.77	0.04	3.36E-05	-1.18	0.11	4.55E-04	-0.13	0.11	6.82E-01	-0.66	0.14	5.33E-02	-0.91	0.22	3.84E-02	-1.31	0.11	2.84E-05	-0.65	0.03	1.18E-03
BBS2	No	Yes	No	Down	0.57	9.46E-05	0.03088	-1.24	0.12	2.39E-03	-0.45	0.04	3.43E-02	-0.50	0.10	1.13E-01	-1.00	0.13	3.28E-02	-1.38	0.17	8.89E-04	-0.70	0.21	9.27E-02	-0.37	0.10	2.46E-01	-0.84	0.03	8.28E-03
CCDC113	No	Yes	No	Down	0.57	1.83E-19	0.00105	-0.88	0.11	1.21E-02	-0.87	0.04	1.45E-05	-0.67	0.11	1.25E-02	-1.14	0.13	1.33E-03	-0.97	0.15	5.38E-01	-0.12	0.20	9.24E-01	-1.14	0.10	6.72E-05	-0.68	0.03	2.63E-04
PLCB4	No	Yes	No	Down	0.57	1.83E-19	1.5E-19	-0.89	0.11	2.83E-03	-0.69	0.04	4.15E-04	-0.63	0.11	1.60E-02	-0.80	0.12	1.19E-02	-1.02	0.15	5.44E-02	-0.96	0.22	8.95E-02	-0.71	0.10	5.38E-03	-0.77	0.03	3.43E-04
TOX3	No	Yes	No	Down	0.57	1.83E-19	0.00023	-0.87	0.11	1.74E-03	-0.48	0.04	1.14E-02	-1.45	0.12	1.82E-06	-0.71	0.12	1.34E-02	-0.81	0.15	7.22E-02	-0.48	0.21	3.88E-01	-1.09	0.10	3.86E-05	-0.58	0.03	1.54E-04
SERPINA6	No	Yes	No	Down	0.57	1.83E-19	1.5E-19	-0.96	0.11	3.37E-04	-0.92	0.04	6.82E-07	-0.61	0.11	2.32E-02	-1.19	0.13	2.44E-04	-0.71	0.14	1.56E-01	-0.80	0.22	3.66E-01	-0.62	0.10	4.24E-02	-0.65	0.03	2.27E-05
TEX264	No	Yes	No	Down	0.57	0.000148	6.8E-05	-0.16	0.10	6.52E-01	-0.53	0.04	1.98E-02	-0.73	0.11	6.65E-02	-0.86	0.12	7.68E-02	-1.13	0.16	1.15E-02	-1.41	0.25	6.25E-03	-0.66	0.10	5.41E-02	-0.96	0.03	4.71E-04
KIF9	No	Yes	No	Down	0.57	1.83E-19	0.0141	-0.58	0.10	7.99E-02	-1.05	0.04	3.18E-06	-0.64	0.11	4.61E-02	-1.48	0.14	2.45E-04	-0.40	0.14	5.25E-01	-0.82	0.22	3.32E-01	-0.99	0.10	1.06E-03	-0.46	0.03	7.12E-03
CDK7	No	No	No	Down	0.57	0.00022	validated	-0.38	0.10	3.64E-01	-0.88	0.04	9.11E-04	-0.76	0.11	3.04E-02	-1.17	0.13	5.97E-02	-0.92	0.15	1.06E-02	-1.43	0.25	5.02E-03	-0.24	0.10	3.33E-01	-0.63	0.03	2.58E-02
C10ORF47	No	No	No	Down	0.57	1.83E-19	validated	-0.54	0.10	7.95E-02	-0.60	0.04	1.30E-03	-0.47	0.10	9.01E-02	-1.02	0.13	1.53E-03	-1.38	0.17	8.48E-04	-1.34	0.25	2.52E-03	-0.55	0.10	3.65E-02	-0.52	0.03	6.90E-03
GALK2	No	Yes	No	Down	0.57	0.000113	0.00029	-0.99	0.11	8.98E-03	-1.00	0.04	2.63E-04	-1.13	0.11	5.70E-03	-0.91	0.12	8.55E-02	-0.69	0.14	1.19E-01	-0.32	0.20	5.02E-01	-0.91	0.10	2.20E-02	-0.47	0.03	5.45E-02
COL4A3BP	No	No	No	Down	0.57	6.71E-06	validated	-0.73	0.11	1.22E-02	-0.38	0.04	3.06E-02	-0.83	0.11	9.68E-03	-1.40	0.14	3.11E-03	-0.67	0.14	2.95E-02	-1.61	0.27	6.18E-04	-0.01	0.10	9.49E-01	-0.78	0.03	7.85E-04
C12ORF45	No	Yes	No	Down	0.57	5.83E-05	0.0082	-0.45	0.10	1.69E-01	-0.93	0.04	1.87E-04	-0.65	0.11	7.03E-02	-1.02	0.13	5.36E-02	-0.64	0.14	5.29E-02	-1.28	0.24	5.06E-03	-0.82	0.10	7.13E-02	-0.62	0.03	9.31E-03
TOM1L1	No	Yes	No	Down	0.57	1.83E-19	0.02792	-0.14	0.10	6.21E-01	-1.00	0.04	8.86E-06	-1.14	0.11	1.52E-03	-0.87	0.12	2.46E-02	-0.47	0.14	1.35E-01	-0.87	0.22	7.09E-02	-0.70	0.10	2.14E-02	-1.23	0.04	1.59E-06
WFDC2	No	No	No	Down	0.57	1.83E-19	validated	-0.63	0.10	1.67E-02	-0.77	0.04	5.91E-06	-0.17	0.10	4.54E-01	-0.98	0.13	3.86E-04	-1.22	0.16	4.94E-03	-0.93	0.22	3.03E-02	-0.98	0.10	5.50E-04	-0.72	0.03	7.72E-06
WDR31	No	Yes	No	Down	0.57	0.000348	0.01344	-1.54	0.12	7.33E-04	-1.07	0.04	1.73E-04	-1.05	0.11	9.68E-03	-0.15	0.11	6.46E-01	-0.55	0.14	4.37E-01	-0.92	0.22	3.21E-01	-0.72	0.10	1.91E-01	-0.40	0.03	2.99E-02
GMDS	No	Yes	No	Down	0.57	1.83E-19	1.5E-19	-0.80	0.11	8.87E-03	-0.86	0.04	2.41E-05	-1.17	0.11	6.50E-04	-0.02	0.11	9.40E-01	-0.80	0.15	4.42E-03	-0.89	0.22	8.50E-03	-0.95	0.10	5.28E-04	-0.90	0.03	2.14E-05
KRT23	No	Yes	No	Down	0.57	1.83E-19	0.00481	-1.35	0.12	2.50E-06	-0.82	0.04	3.41E-06	-0.26	0.10	2.21E-01	-0.82	0.12	2.34E-03	-0.72	0.14	2.28E-02	-0.49	0.21	1.49E-01	-1.05	0.10	1.90E-04	-0.88	0.03	1.00E-20
UQCRQ	No	Yes	No	Down	0.57	8.89E-05	0.00226	-1.20	0.11	3.25E-03	-0.44	0.04	7.46E-02	-1.45	0.12	4.70E-03	-0.62	0.12	1.75E-01	-0.45	0.14	5.93E-02	-1.47	0.25	1.70E-04	-0.23	0.10	3.92E-01	-0.54	0.03	5.63E-02
TESC	No	Yes	Yes	Down	0.57	1.83E-19	1.5E-19	-1.75	0.13	2.27E-07	-0.87	0.04	2.50E-06	-0.46	0.10	4.90E-02	-0.83	0.12	3.45E-03	-0.85	0.15	5.96E-03	-0.35	0.20	1.84E-01	-0.37	0.10	1.16E-01	-0.91	0.03	1.36E-06
USP54	No	Yes	No	Down	0.58	6.71E-06	0.00133	-0.46	0.10	2.18E-01	-0.97	0.04	2.41E-05	-1.07	0.11	2.34E-03	-1.21	0.13	2.56E-03	-0.77	0.15	1.63E-01	-0.41	0.20	4.05E-01	-0.62	0.10	5.12E-02	-0.87	0.03	1.53E-03
MDM2	No	Yes	No	Down	0.58	0.000227	1.5E-19	-1.38	0.12	5.81E-04	-0.36	0.04	7.68E-02	-0.78	0.11	2.62E-02	-1.10	0.13	5.28E-03	-1.24	0.16	1.70E-01	-0.62	0.21	7.01E-01	-0.38	0.10	1.91E-01	-0.52	0.03	2.15E-03
KLHDC9	No	Yes	No	Down	0.58	1.83E-19	0.01817	-0.48	0.10	1.10E-01	-1.17	0.04	6.82E-07	-0.59	0.11	5.71E-02	-1.05	0.13	1.44E-03	-0.85	0.15	9.47E-03	-0.75	0.21	4.67E-02	-0.61	0.10	7.08E-02	-0.89	0.03	2.96E-04
TXK	No	Yes	No	Down	0.58	1.83E-19	0.00096	-0.45	0.10	1.73E-01	-0.50	0.04	5.54E-03	-0.81	0.11	5.26E-03	-1.54	0.14	1.52E-05	-0.70	0.14	4.12E-01	-0.63	0.21	4.93E-01	-0.81	0.10	8.50E-03	-0.93	0.03	2.04E-06
BSG	No	No	No	Down	0.58	1.14E-05	0.00344	-0.27	0.10	4.51E-01	-0.50	0.04	1.50E-02	-0.63	0.11	5.96E-02	-2.36	0.19	2.69E-04	-0.05	0.14	6.93E-01	-0.62	0.21	4.01E-02	-1.17	0.10	5.42E-04	-0.78	0.03	9.64E-04
ERBB2IP	No	No	No	Down	0.58	0.002361	validated	-1.68	0.13	7.12E-04	-0.74	0.04	3.83E-03	-0.63	0.11	7.09E-02	-0.66	0.12	1.16E-01	-1.01	0.15	2.45E-01	-0.85	0.22	4.01E-01	-0.49	0.10	1.17E-01	-0.31	0.03	1.75E-01
DOCK5	No	Yes	Yes	Down	0.58	5.41E-05	1.5E-19	-0.47	0.10	9.46E-02	-0.45	0.04	5.66E-02	-1.11	0.11	1.99E-04	-1.09	0.13	8.98E-04	-0.59	0.14	2.47E-01	-1.78	0.28	1.23E-01	-0.51	0.10	3.08E-02	-0.34	0.03	2.24E-02
SERPINA1	No	Yes	No	Down	0.58	1.83E-19	3.5E-05	-0.82	0.11	2.42E-03	-0.70	0.04	2.43E-05	-0.50	0.10	2.52E-02	-0.79	0.12	4.04E-03	-0.81	0.15	3.64E-03	-0.75	0.21	1.75E-02	-1.05	0.10	5.18E-05	-0.92	0.03	1.00E-20
REPS2	No	Yes	No	Down	0.58	1.83E-19	1.5E-19	-1.04	0.11	9.25E-04	-1.28	0.04	1.00E-20	-0.61	0.11	2.72E-02	-0.10	0.11	6.95E-01	-0.88	0.15	1.66E-02	-0.71	0.21	6.99E-02	-0.85	0.10	2.48E-03	-0.87	0.03	3.18E-06
C16ORF48	No	Yes	No	Down	0.58	1.83E-19	0.00247	-1.01	0.11	3.87E-03	-0.62	0.04	2.56E-03	-0.41	0.10	1.76E-01	-1.23	0.13	2.72E-03	-0.61	0.14	4.17E-02	-0.60	0.21	1.11E-01	-0.75	0.10	1.83E-02	-1.12	0.04	1.36E-06
COASY	No	No	No	Down	0.58	0.000283	0.01255	-0.56	0.10	1.07E-01	-0.34	0.04	1.16E-01	-1.12	0.11	5.81E-03	-1.18	0.13	3.29E-02	-0.55	0.14	5.08E-02	-0.74	0.21	3.98E-02	-0.92	0.10	3.04E-02	-0.93	0.03	2.98E-03
ARHGEF3	No	Yes	No	Down	0.58	1.83E-19	0.00019	-0.76	0.11	7.45E-03	-0.90	0.04	2.54E-05	-1.03	0.11	2.54E-03	-0.71	0.12	4.11E-02	-0.83	0.15	3.99E-03	-0.62	0.21	4.39E-02	-0.58	0.10	4.04E-02	-0.90	0.03	1.24E-04
CCDC96	No	Yes	No	Down	0.58	3.58E-06	0.00015	-0.62	0.10	4.06E-02	-0.81	0.04	2.18E-05	-0.88	0.11	3.74E-03	-1.08	0.13	4.06E-03	-0.95	0.15	1.38E-01	-0.70	0.21	3.73E-01	-0.84	0.10	3.11E-03	-0.45	0.03	1.51E-02
PLEKHH1	No	Yes	No	Down	0.58	3.58E-06	0.0331	-0.13	0.10	7.24E-01	-0.46	0.04	2.70E-02	-0.79	0.11	1.33E-02	-1.57	0.15	7.20E-05	-0.68	0.14	6.39E-02	-1.06	0.23	7.53E-02	-0.63	0.10	2.30E-02	-1.00	0.03	7.27E-06
UBE2D2	No	No	No	Down	0.58	1.14E-05	validated	-0.66	0.11	5.02E-02	-0.80	0.04	4.09E-04	-1.10	0.11	2.49E-03	-0.36	0.11	3.22E-01	-1.01	0.15	1.17E-02	-0.94	0.22	3.77E-02	-0.52	0.10	7.16E-02	-0.93	0.03	7.02E-04
DCBLD1	No	Yes	No	Down	0.58	8.51E-05	0.00034	-0.92	0.11	5.81E-03	-0.64	0.04	3.55E-03	-1.15	0.11	2.47E-03	-0.56	0.12	1.00E-01	-1.06	0.15	6.25E-02	-0.77	0.21	2.32E-01	-0.49	0.10	7.16E-02	-0.73	0.03	7.13E-03
ZFHX3	No	Yes	No	Down	0.58	1.83E-19	0.00061	-1.25	0.12	5.20E-04	-0.29	0.04	7.83E-02	-0.85	0.11	1.54E-02	-0.96	0.13	1.48E-02	-0.59	0.14	3.54E-02	-0.09	0.20	6.43E-01	-1.84	0.11	8.63E-06	-0.46	0.03	6.67E-03
DACT2	No	No	No	Down	0.58	1.83E-19	validated	-0.88	0.11	4.55E-04	-0.99	0.04	4.54E-07	-0.79	0.11	8.49E-03	-0.78	0.12	4.06E-03	-1.18	0.16	7.02E-04	-0.36	0.20	2.02E-01	-0.66	0.10	2.55E-02	-0.66	0.03	1.91E-04

Supplementary Table 2. Details for 2,053 genes differentially expressed between EEC and NEEC, identified by microarray meta-analysis.

EEC - endometrioid endometrial cancer, NEEC - non-endometrioid endometrial cancer, SMD - Standardised Mean Difference, FDR - False Discovery Rate, Var - Variance

Gene	145 gene list	1253 gene list	Associated with EEC-specific survival?	Up/Down Regulated in NEEC	average standardised fold change	Microarray Meta FDR	RNASeq FDR	Individual microarray study results																							
								TCGA			E-MTAB-2532			E-GEOD-56026			GSE32507			GSE24537			E-GEOD-23518			E-GEOD-17025			E-GEOD-2109		
								SMD	Var	P-value	SMD	Var	P-value	SMD	Var	P-value	SMD	Var	P-value	SMD	Var	P-value	SMD	Var	P-value	SMD	Var	P-value	SMD	Var	P-value
SLC6A6	No	Yes	No	Down	0.58	3.97E-05	0.00075	-0.81	0.11	2.87E-02	-0.69	0.04	8.82E-04	-1.12	0.11	2.79E-03	-0.95	0.13	8.24E-03	-0.31	0.14	5.83E-01	-1.44	0.25	2.49E-01	-0.43	0.10	1.24E-01	-0.55	0.03	9.30E-04
SIDT1	No	Yes	No	Down	0.58	1.83E-19	1.5E-19	-0.59	0.10	3.00E-02	-1.42	0.04	1.00E-20	-0.78	0.11	4.10E-03	-0.64	0.12	2.18E-02	-0.67	0.14	1.73E-01	-0.78	0.22	1.72E-01	-0.76	0.10	3.40E-03	-0.66	0.03	6.84E-04
CCDC11	No	Yes	No	Down	0.58	1.83E-19	0.00361	-0.76	0.11	2.94E-03	-0.61	0.04	2.79E-04	-0.62	0.11	1.54E-02	-1.05	0.13	8.18E-04	-1.05	0.15	7.93E-02	-0.80	0.22	3.47E-01	-0.80	0.10	2.45E-03	-0.58	0.03	8.76E-04
ARFIP2	No	Yes	No	Down	0.58	0.00028	0.00016	-0.70	0.11	6.13E-02	-1.25	0.04	3.43E-05	-0.40	0.10	2.41E-01	-1.40	0.14	1.32E-02	-0.15	0.14	7.22E-01	-0.51	0.21	4.08E-01	-1.28	0.10	4.67E-03	-0.58	0.03	4.62E-02
DLX5	No	Yes	No	Down	0.58	1.83E-19	1.5E-19	-1.01	0.11	3.26E-04	-0.51	0.04	2.19E-03	-0.56	0.11	2.13E-02	-0.53	0.12	4.79E-02	-0.79	0.15	4.83E-03	-1.36	0.25	4.01E-04	-1.08	0.10	1.38E-04	-0.44	0.03	2.45E-03
INTS9	No	Yes	No	Down	0.58	8.89E-05	1.5E-19	-1.37	0.12	2.47E-03	-0.89	0.04	5.53E-04	-1.14	0.11	8.65E-03	-0.60	0.12	1.61E-01	-0.65	0.14	3.19E-02	-0.62	0.21	1.18E-01	-0.75	0.10	4.06E-02	-0.25	0.03	1.54E-01
KIAA1244	No	Yes	No	Down	0.58	1.83E-19	1.5E-19	-1.23	0.12	2.59E-04	-0.90	0.04	2.79E-05	-0.82	0.11	5.11E-03	-0.56	0.12	4.36E-02	-0.53	0.14	3.59E-01	-0.99	0.22	2.02E-01	-0.83	0.10	4.16E-03	-0.41	0.03	1.43E-02
SORD	No	Yes	No	Down	0.58	1.83E-19	0.00036	-0.92	0.11	4.20E-03	-1.13	0.04	4.54E-07	-0.91	0.11	2.13E-03	-1.27	0.13	4.85E-04	-0.50	0.14	6.06E-02	-0.05	0.20	8.66E-01	-0.63	0.10	1.53E-02	-0.87	0.03	3.57E-05
PHYHD1	No	Yes	No	Down	0.58	1.83E-19	0.0001	-0.73	0.11	6.51E-03	-0.85	0.04	9.77E-06	-0.96	0.11	2.36E-03	-1.34	0.14	1.22E-04	-0.59	0.14	1.60E-01	-0.77	0.21	1.80E-01	-0.33	0.10	1.92E-01	-0.68	0.03	5.90E-04
CPNE2	No	Yes	No	Down	0.58	3.58E-06	2E-05	-0.87	0.11	5.72E-03	-1.06	0.04	1.05E-05	-0.97	0.11	3.61E-03	-0.87	0.12	2.07E-02	-0.17	0.14	5.65E-01	-1.13	0.23	7.71E-02	-0.39	0.10	1.45E-01	-0.79	0.03	8.31E-04
CTNNBIP1	No	Yes	No	Down	0.58	1.14E-05	0.00014	-1.59	0.12	6.86E-05	-0.52	0.04	1.21E-02	-0.29	0.10	4.09E-01	-0.20	0.11	6.30E-01	-0.96	0.15	9.98E-03	-0.92	0.22	2.19E-02	-0.99	0.10	1.30E-02	-0.77	0.03	2.98E-03
HS3ST1	No	Yes	No	Down	0.58	1.83E-19	1.5E-19	-1.33	0.12	2.68E-05	-1.20	0.04	2.27E-07	-0.64	0.11	1.35E-02	-0.63	0.12	2.82E-02	-0.74	0.14	1.64E-02	-0.85	0.22	2.81E-02	-0.50	0.10	3.85E-02	-0.33	0.03	3.93E-02
RG9MTD2	No	No	No	Down	0.58	5.61E-05	validated	-0.79	0.11	1.63E-02	-0.97	0.04	2.26E-04	-0.81	0.11	1.21E-02	-1.29	0.14	3.95E-03	-0.45	0.14	3.10E-01	-0.87	0.22	1.63E-01	-0.75	0.10	2.19E-02	-0.31	0.03	1.07E-01
MCCC2	No	No	No	Down	0.58	6.71E-06	validated	-0.85	0.11	1.09E-02	-0.70	0.04	1.36E-03	-0.49	0.10	6.34E-02	-1.65	0.15	7.07E-04	-1.08	0.15	7.59E-03	-0.60	0.21	1.11E-01	-0.17	0.10	4.89E-01	-0.69	0.03	8.25E-04
XG	No	Yes	No	Down	0.58	1.83E-19	1.5E-19	-0.62	0.10	7.88E-03	-0.73	0.04	2.86E-05	-1.06	0.11	1.25E-04	-0.57	0.12	1.67E-02	-0.69	0.14	3.93E-01	-0.62	0.21	5.06E-01	-1.31	0.11	3.86E-06	-0.61	0.03	4.89E-04
HLA-DMB	No	No	No	Down	0.58	1.83E-19	validated	-0.10	0.10	7.01E-01	-1.00	0.04	6.82E-07	-0.83	0.11	4.73E-03	-0.97	0.13	3.11E-03	-0.52	0.14	3.35E-02	-0.92	0.22	5.54E-03	-1.13	0.10	1.78E-04	-0.75	0.03	2.26E-04
SPECC1	No	No	No	Down	0.58	1.83E-19	validated	-0.32	0.10	2.10E-01	-1.10	0.04	6.82E-07	-0.98	0.11	1.80E-03	-1.32	0.14	1.22E-04	-0.84	0.15	3.95E-01	-0.29	0.20	7.10E-01	-0.53	0.10	4.48E-02	-0.82	0.03	2.73E-06
GREB1	No	Yes	No	Down	0.58	1.83E-19	1.5E-19	-1.12	0.11	4.45E-04	-0.75	0.04	5.04E-05	-0.49	0.10	3.85E-02	-0.83	0.12	2.92E-03	-0.89	0.15	3.24E-02	-0.53	0.21	1.79E-01	-0.69	0.10	6.78E-03	-0.90	0.03	1.36E-06
ANKMY1	No	No	No	Down	0.58	3.58E-06	validated	-0.55	0.10	6.79E-02	-0.73	0.04	4.91E-04	-0.52	0.11	1.35E-01	-1.44	0.14	4.22E-04	-0.88	0.15	5.54E-03	-0.88	0.22	5.35E-02	-0.63	0.10	9.21E-02	-0.57	0.03	9.10E-04
FAAH	No	Yes	No	Down	0.59	4.80E-05	0.00208	-1.03	0.11	6.24E-03	-0.65	0.04	3.22E-03	-0.44	0.10	1.87E-01	-1.61	0.15	3.71E-04	-0.43	0.14	1.65E-01	-1.09	0.23	2.84E-02	-0.32	0.10	2.61E-01	-0.62	0.03	6.88E-03
ACP6	No	Yes	No	Down	0.59	1.14E-05	0.00651	-0.63	0.10	4.90E-02	-0.68	0.04	1.08E-03	-0.67	0.11	3.48E-02	-0.88	0.12	2.86E-02	-0.75	0.14	5.78E-02	-0.65	0.21	1.32E-01	-1.14	0.10	9.59E-04	-0.77	0.03	2.99E-03
FAM83E	No	No	No	Down	0.59	3.58E-05	0.01527	-0.47	0.10	9.87E-02	-0.95	0.04	1.98E-05	-1.03	0.11	7.54E-03	-0.99	0.13	5.54E-03	-1.15	0.16	4.75E-02	-1.10	0.23	1.07E-01	-0.06	0.10	8.50E-01	-0.41	0.03	2.17E-02
ATPIF1	No	Yes	No	Down	0.59	9.46E-05	0.0002	-0.22	0.10	5.17E-01	-0.56	0.04	1.64E-02	-0.34	0.10	2.64E-01	-1.52	0.14	2.51E-03	-0.71	0.14	1.66E-02	-1.59	0.26	2.22E-04	-0.95	0.10	8.49E-03	-0.26	0.03	2.90E-01
DNAH9	No	Yes	No	Down	0.59	1.83E-19	1.5E-19	-1.07	0.11	4.07E-05	-0.95	0.04	6.82E-07	-0.77	0.11	6.06E-03	-0.98	0.13	8.06E-04	-0.46	0.14	1.11E-01	-0.50	0.21	1.57E-01	-0.79	0.10	2.83E-03	-0.63	0.03	6.02E-05
HEBP2	No	No	No	Down	0.59	2.85E-05	validated	-0.89	0.11	1.25E-02	-0.67	0.04	1.36E-03	-1.03	0.11	1.04E-02	-0.74	0.12	8.25E-02	-0.31	0.14	1.76E-01	-1.47	0.25	1.65E-03	-0.07	0.10	7.73E-01	-0.95	0.03	1.85E-03
C20ORF85	No	Yes	No	Down	0.59	1.83E-19	2.3E-06	-0.68	0.11	2.61E-03	-0.92	0.04	1.00E-20	-0.63	0.11	6.27E-03	-0.76	0.12	2.79E-03	-0.95	0.15	2.12E-03	-0.76	0.21	3.62E-02	-0.72	0.10	2.68E-03	-0.70	0.03	9.09E-07
ETS2	No	Yes	No	Down	0.59	1.83E-19	0.00246	-1.19	0.11	4.70E-04	-0.87	0.04	1.73E-05	-0.84	0.11	1.15E-02	-0.31	0.11	3.21E-01	-0.75	0.14	1.01E-02	-0.75	0.21	2.62E-02	-0.58	0.10	2.82E-02	-0.83	0.03	1.20E-04
AKAP7	No	Yes	No	Down	0.59	1.83E-19	0.04129	-1.10	0.11	3.18E-04	-1.01	0.04	4.32E-06	-0.67	0.11	1.38E-02	-0.46	0.12	1.12E-01	-0.87	0.15	8.31E-03	-0.79	0.22	2.95E-02	-0.54	0.10	3.23E-02	-0.70	0.03	1.99E-03
PSMB10	No	No	No	Down	0.59	3.58E-06	0.02745	-0.49	0.10	8.10E-02	-0.63	0.04	1.70E-03	-0.73	0.11	1.85E-02	-1.29	0.13	2.25E-03	-0.60	0.14	2.62E-02	-0.78	0.22	1.78E-02	-0.88	0.10	4.73E-03	-0.70	0.03	1.68E-03
RIBC2	No	Yes	No	Down	0.59	1.83E-19	0.00071	-1.10	0.11	9.38E-05	-0.48	0.04	2.36E-03	-0.62	0.11	2.49E-02	-0.39	0.12	1.45E-01	-1.23	0.16	2.14E-01	-0.75	0.21	5.21E-01	-0.74	0.10	1.01E-02	-0.79	0.03	9.09E-07
C10ORF57	No	Yes	No	Down	0.59	3.58E-06	0.00206	-0.78	0.11	2.42E-02	-0.99	0.04	2.07E-05	-0.72	0.11	2.31E-02	-0.33	0.11	3.41E-01	-1.27	0.16	6.07E-04	-0.90	0.22	1.87E-02	-0.67	0.10	4.48E-02	-0.44	0.03	3.84E-02
ST6GALNAC2	No	Yes	Yes	Down	0.59	1.83E-19	0.00155	-0.48	0.10	4.27E-02	-0.87	0.04	7.72E-06	-0.19	0.10	4.21E-01	-1.07	0.13	7.47E-04	-0.99	0.15	3.17E-02	-0.50	0.21	3.17E-01	-0.89	0.10	3.28E-03	-1.09	0.04	1.00E-20
TTC19	No	Yes	No	Down	0.59	2.19E-05	0.00122	-0.75	0.11	2.77E-02	-0.99	0.04	4.23E-04	-0.56	0.11	9.28E-02	-0.16	0.11	6.97E-01	-0.90	0.15	6.09E-03	-1.02	0.23	9.19E-03	-0.44	0.10	1.10E-01	-1.26	0.04	6.65E-04
GRAMD3	No	No	No	Down	0.59	3.58E-06	validated	-0.29	0.10	2.95E-01	-0.84	0.04	2.63E-04	-0.64	0.11	2.24E-02	-0.93	0.12	7.79E-03	-1.07	0.15	6.18E-03	-1.04	0.23	2.06E-02	-0.29	0.10	2.03E-01	-0.97	0.03	1.49E-04
KCTD14	No	Yes	No	Down	0.59	1.83E-19	1.5E-19	-0.89	0.11	1.49E-03	-0.86	0.04	1.84E-05	-0.65	0.11	1.26E-02	-0.41	0.12	1.26E-01	-0.57	0.14	3.21E-02	-0.78	0.22	3.01E-02	-1.16	0.10	9.97E-05	-0.76	0.03	1.83E-04
RFK	No	No	No	Down	0.59	0.005239	validated	-0.53	0.10	8.06E-02	-0.54	0.04	5.76E-03	-0.64	0.11	4.46E-02	-1.04	0.13	1.35E-02	-1.21	0.16	2.87E-01	-1.48	0.25	1.91E-01	-0.04	0.10	8.45E-01	-0.59	0.03	2.36E-02
GCNT2	No	Yes	No	Down	0.59	1.83E-19	1.9E-05	-0.91	0.11	2.75E-03	-0.64	0.04	2.56E-03	-0.47	0.10	7.64E-02	-0.64	0.12	2.55E-02	-0.35	0.14	1.92E-01	-0.93	0.22	3.81E-02	-1.46	0.11	8.18E-06	-0.66	0.03	2.74E-04
TSKU	No	Yes	No	Down	0.59	3.58E-06	1.5E-19	-1.28	0.12	1.54E-04	-0.71	0.04	4.68E-04	-0.52	0.11	1.20E-01	-0.55	0.12	5.96E-02	-1.07	0.15	7.64E-04	-1.02	0.23	5.54E-03	-0.43	0.10	1.72E-01	-0.48	0.03	3.51E-02
UPK1B	No	Yes	No	Down	0.59	1.83E-19	3.8E-05	-0.48	0.10	3.73E-02	-1.14	0.04	1.00E-20	-1.58	0.12	1.00E-20	-0.26	0.11	2.43E-01	-0.65	0.14	8.67E-02	-0.50	0.21	3.17E-01	-0.81	0.10	7.66E-04	-0.65	0.03	1.39E-05
AACS	No	Yes	No	Down	0.59	0.000227	0.00311	-0.50	0.10	1.30E-01	-0.79	0.04	9.26E-04	-0.76	0.11	3.04E-02	-0.64	0.12	1.48E-01	-0.70	0.14	5.06E-02	-1.18	0.23	5.56E-02	-0.61	0.10	6.43E-02	-0.88	0.03	3.05E-03
ROPN1L	No	Yes	No	Down	0.59	1.83E-19	3E-05	-0.73	0.11	1.90E-03	-0.94	0.04	4.54E-07	-0.65	0.11	7.24E-03	-0.57	0.12	2.79E-02	-0.99	0.15	1.55E-03	-0.72	0.21	4.25E-02	-0.76	0.10	4.32E-03	-0.69	0.03	2.04E-06
DHRS3	No	No	No	Down	0.59	1.83E-19	validated	-0.53	0.10	4.54E-02	-0.52	0.04	2.64E-03	-0.72	0.11	1.10E-02	-0.29	0.11	3.27E-01	-0.91	0.15	3.30E-03	-1.37	0.25	7.00E-04	-0.99	0.10	8.10E-04	-0.72	0.03	1.15E-03
MTUS1	No	Yes	No	Down	0.59	1.83E-19	1.5E-19	-1.72	0.13	1.52E-05	-1.08	0.04	5.45E-06	-0.77	0.11	1.31E-02	-0.36	0.11	2.37E-01	-0.24	0.14	3.67E-01	-0.14	0.20	5.78E-01	-1.09	0.10	2.21E-04	-0.64	0.03	9.36E-03
RPS6KA1	No	Yes	No	Down	0.59	0.00011	0.00092	-0.73	0.11	5.02E-02	-0.67	0.04	3.38E-03	-0.67	0.11	4.44E-02	-1.34	0.14	5.65E-03	-0.07	0.14	6.36E-01	-0.97	0.22	2.01E-02	-0.90	0.10	2.12E-02	-0.69	0.03	6.68E-03
DYNLRB2	No	Yes	No	Down	0.59	1.83E-19	9.2E-05	-0.99	0.11	1.47E-04	-0.88	0.04	2.27E-06	-0.25	0.10	2.29E-01	-0.24	0.11	3.24E-01	-1.02	0.15	1.91E-03	-0.84	0.22	3.88E-02	-1.04	0.10	1.06E-04	-0.77	0.03	6.82E-07
CTSL2	No	Yes	No	Down	0.59	1.83E-19	1.5E-19	-0.69	0.11	6.00E-03	-0.74	0.04	1.11E-05	-0.49	0.10	4.09E-02	-0.76	0.12	7.12E-03	-1.04	0.15	1.55E-03	-0.97	0.22	1.12E-02	-0.56	0.10	1.68E-02	-0.77	0.03	1.73E-05
LRIG1	No	No	No	Down	0.59	1.83E-19	validated	-0.80	0.11	3.10E-03	-0.67	0.04	2.24E-04	-0.55	0.11	3.46E-02	-0.43	0.12	1.72E-01	-1.53	0.17	2.57E-05	-0.51	0.21	8.33E-02	-0.97	0.10	4.31E-04	-0.56	0.03	2.45E-03
WDR34	No	Yes	No	Down	0.59	4.80E-05	0.00064	-0.79	0.11	2.88E-02	-0.71	0.04	1.97E-03	-0.63	0.11	6.51E-02	-0.51	0.12	2.22E-01	-1.18	0.16	2.29E-03	-1.25	0.24	4.25E-03	-0.23	0.10	4.53E-01	-0.70	0.03	3.91E-03
TBC1D2	No	Yes	No	Down	0.60	3.97E-05	0.00024	-0.80	0.11	1.43E-02	-0.80	0.04	2.41E-04	-0.44	0.10	1.47E-01	-0.49	0.12	1.55E-01	-0.89	0.15	2.57E-02	-1.43	0.25	5.47E-02	-0.33	0.10	2.99E-01	-0.81	0.03	2.34E-04
ZMYND10	No	Yes	No	Down	0.60	1.83E-19	4.6E-06	-0.91	0.11	7.17E-04	-0.88	0.04	1.59E-06	-0.65	0.11	1.91E-02	-0.38	0.11	1.90E-01	-0.94	0.15	5.36E-03	-0.79	0.22	7.60E-02	-0.53	0.10	4.74E-02	-0.91	0.03	4.54E-07
WDR49	No	Yes	No	Down	0.60	1.83E-19	0.01079	-0.42	0.10	1.08E-01	-0.69	0.04	1.90E-04	-0.67	0.11	8.79E-03	-0.53	0.12	4.03E-02	-1.00	0.15	9.56E-02	-1.13	0.23	2.68E-01	-0.81	0.10	1.51E-03	-0.72	0.03	3.63E-06
ACADVL	No	Yes	No	Down	0.60	2.64E-05	0.00072	-0.70	0.11	5.59E-02	-0.54	0.04	1.54E-02	-0.74	0.11	5.82E-02	-1.28	0.13	3.57E-03	-0.35	0.14	1.14E-01	-0.20	0.20	3.64E-01	-1.07	0.10	1.48E-03	-1.09	0.04	4.72E-04
LRP5L	No	Yes	No	Down	0.60	5.41E-05	0.00074	-0.83	0.11	8.80E-03	-0.49	0.04	9.02E-03	-0.06	0.10	8.62E-01	-2.42	0.19	1.98E-05	-0.42	0.14	1.59E-01	-0.73	0.21	1.25E-01	-0.34	0.10	1.99E-01	-0.66	0.03	3.42E-03
EPS8	No	Yes	Yes	Down	0.60	3.58E-06	1.5E-19	-1.00	0.11	2.02E-03	-1.13	0.04	5.23E-06	-0.82	0.11	1.08E-02	-0.10	0.11	7.29E-01	-0.74	0.14	6.87E-02	-0.59	0.21	1.72E-01	-0.87	0.10	3.76E-03	-0.70	0.03	5.63E-03
HMGCR	No	No	No	Down	0.60	1.83E-19	0.01117	-0.67	0.11	6.00E-02	-0.67	0.04	3.65E-04	-0.89	0.11	3.06E-03	-1.00	0.13	1.06E-02	-0.42	0.14	7.70E-02	-1.36	0.25	7.65E-04	-0.21	0.10	3.28E-01	-0.73	0.03	3.17E-04
YIPF1	No	No	No	Down	0.60	0.000526	validated	-0.23	0.10	4.94E-01	-0.66	0.04	1.20E-02	-0.67	0.11	1.06E-01	-0.92	0.12	8.86E-02	-0.94	0.15	6.02E-03	-0.21	0.20	4.05E-01	-1.63	0.11	2.79E-04	-0.68	0.03	3.73E-02
MSX2	No	Yes	No	Down	0.60	1.83E-19	1.5E-19	-1.31	0.12	9.77E-06	-0.68	0.04	7.34E-05	-0.29	0.10	1.75E-01	-0.51	0.12	4.27E-02	-0.55	0.14	1.51E-01	-0.85	0.22	1.38E-01	-1.02	0.10	9.88E-05	-0.72	0.03	3.41E-06
SYK	No	No	No	Down	0.60	1.83E-19	0.01524	-0.78	0.11	1.32E-02	-0.90	0.04	3.54E-05	-0.81	0.11	7.19E-03	-0.50	0.12	1.56E-01	-1.30	0.16	5.24E-04	-0.50	0.21	9.44E-02	-0.67	0.10	1.25E-02	-0.48	0.03	1.39E-02
ROBO2	No	Yes	No	Down	0.60	3.58E-06	1.5E-19	-0.59	0.10	1.96E-02	-0.61	0.04	1.03E-03	-0.84	0.11	1.19E-03	-0.30	0.11	2.34E-01	-0.90	0.15	1.33E-01	-1.09	0.23	1.05E-01	-1.24	0.10	1.00E-05	-0.37	0.03	1.77E-02
CDRT4	No	Yes	No	Down	0.60	6.71E-06	9.2E-05	-0.62	0.10	8.56E-02	-0.53	0.04	6.44E-03	-0.69	0.11	3.94E-02	-0.65	0.12	6.14E-02	-0.44	0.14	9.92E-02	-0.50	0.21	2.33E-01	-1.24	0.10	1.67E-04	-1.25	0.04	2.75E-05
CDC20B	No	Yes	No	Down	0.60	1.83E-19	2.3E-06	-0.64	0.11	2.99E-02	-0.60	0.04	3.31E-03	-0.77	0.11	1.28E-03	-0.42	0.12	9.89E-02	-0.93	0.15	1.33E-02	-0.84	0.22	6.45E-02	-0.87	0.10	1.99E-04	-0.85	0.03	1.00E-20
B4GALNT3	No	Yes	Yes	Down	0.60	6.71E-06	1.5E-19	-0.34	0.10	4.06E-01	-1.14	0.04	4.54E-07	-0.94	0.11	3.36E-03	-1.31	0.14	1.40E-04	-0.84	0.15	4.23E-01	-0.47	0.21	6.30E-01	-0.83	0.10	6.70E-03	-0.05	0.03	7.45E-01
AQP5	No	Yes	No	Down	0.60	1.83E-19	0.00021	-0.91	0.11	2.36E-03	-1.14	0.04	1.00E-20	-0.59	0.11	1.98E-02	-1.04	0.13	2.65E-04	-0.62	0.14	3.39E-02	-0.52	0.21	1.19E-01	-0.49	0.10	7.31E-02	-0.59	0.03	2.45E-04
GOLT1A	No	Yes	No	Down	0.60	1.83E-19	0.00082	-0.45	0.10	7.43E-02	-0.71	0.04	1.39E-04	-0.99	0.11	6.20E-04	-0.78	0.12	8.94E-03	-0.74	0.14	3.04E-02	-1.00	0.23	1.98E-02	-0.70	0.10	2.76E-02	-0.53	0.03	7.45E-04
AK7	No	Yes	No	Down	0.60	1.83E-19	0.03958	-0.97	0.11	6.67E-04	-0.87	0.04	6.59E-06	-0.44	0.10	6.05E-02	-1.14	0.13	5.54E-04	-0.60	0.14	6.89E-01	-0.55	0.21	7.00E-01	-0.58	0.10	3.88E-02	-0.75	0.03	5.23E-06
GFOD2	No	Yes	No	Down	0.60	0.001452	0.0014	-0.86	0.11	3.36E-02	-0.57	0.04	2.62E-02	-0.46	0.10	2.86E-01	-0.90	0.12	6.27E-02	-0.66	0.14	8.36E-02	-0.94	0.22	4.45E-02	-0.99	0.10	1.87E-02	-0.51	0.03	1.37E-02
TMED3	No	Yes	No	Down	0.60	1.83E-19	0.00092	-0.62	0.10	5.24E-02	-0.86	0.04	9.11E-05	-0.76	0.11	2.07E-02	-0.64	0.12	6.83E-02	-0.78	0.15	4.93E-03	-0.48	0.21	8.60E-02	-0.90	0.10	2.00E-03	-0.85	0.03	1.42E-03
ARHGEF18	No	Yes	No	Down	0.60	0.000482	0.00097	-0.17	0.10	6.89E-01	-0.83	0.04	3.75E-03	-0.42	0.10	1.87E-01	-0.67	0.12	1.48E-01	-1.03	0.15	2.25E-03	-0.87	0.22	2.88E-02	-0.93	0.10	2.35E-02	-0.95	0.03	1.05E-02
SCGB1D1	No	Yes	No	Down	0.60	1.83E-19	0.00061	-1.14	0.11	2.25E-05	-0.96	0.04	1.00E-20	-0.70	0.11	4.54E-03	-0.88	0.12	6.03E-04	-0.32	0.14	1.47E-01	-1.15	0.23	4.79E-01	-0.35	0.10	1.22E-01	-0.37	0.03	8.53E-03

Supplementary Table 2. Details for 2,053 genes differentially expressed between EEC and NEEC, identified by microarray meta-analysis.

EEC - endometrioid endometrial cancer, NEEC - non-endometrioid endometrial cancer, SMD - Standardised Mean Difference, FDR - False Discovery Rate, Var - Variance

Gene	145 gene list	1253 gene list	Associated with EEC-specific survival?	Up/Down Regulated in NEEC	average standardised fold change	Microarray Meta FDR	RNASeq FDR	Individual microarray study results																							
								TCGA			E-MTAB-2532			E-GEOD-56026			GSE32507			GSE24537			E-GEOD-23518			E-GEOD-17025			E-GEOD-2109		
								SMD	Var	P-value	SMD	Var	P-value	SMD	Var	P-value	SMD	Var	P-value	SMD	Var	P-value	SMD	Var	P-value	SMD	Var	P-value	SMD	Var	P-value
MYO6	No	No	No	Down	0.60	1.83E-19	validated	-0.49	0.10	8.52E-02	-0.67	0.04	7.00E-04	-0.52	0.11	8.62E-02	-1.24	0.13	4.75E-04	-0.60	0.14	9.38E-02	-1.02	0.23	2.11E-02	-0.24	0.10	3.11E-01	-1.10	0.04	2.27E-07
SCGB2A2	No	Yes	Yes	Down	0.60	1.83E-19	1.5E-19	-1.11	0.11	1.73E-05	-0.73	0.04	9.31E-06	-0.99	0.11	1.20E-04	-0.35	0.11	1.13E-01	-0.35	0.14	1.11E-01	-0.47	0.21	8.87E-02	-0.83	0.10	4.70E-04	-1.03	0.04	1.00E-20
ZNF516	No	Yes	Yes	Down	0.60	1.83E-19	2.3E-06	-1.46	0.12	8.18E-06	-0.75	0.04	2.04E-04	-0.24	0.10	3.04E-01	-0.51	0.12	9.05E-02	-0.50	0.14	9.61E-02	-0.50	0.21	2.96E-01	-1.15	0.10	2.78E-04	-0.75	0.03	4.63E-04
SAT2	No	Yes	No	Down	0.60	0.000211	1.5E-19	-1.38	0.12	1.02E-03	-0.38	0.04	5.63E-02	-0.99	0.11	1.47E-02	-0.67	0.12	7.75E-02	-0.07	0.14	6.96E-01	-0.51	0.21	1.67E-01	-1.32	0.11	4.53E-04	-0.55	0.03	7.71E-02
CISH	No	Yes	No	Down	0.60	1.66E-05	9.2E-05	-1.12	0.11	3.85E-03	-0.54	0.04	3.39E-03	-1.40	0.12	4.08E-04	-0.04	0.11	8.75E-01	-0.45	0.14	2.20E-01	-1.07	0.23	3.92E-02	-0.49	0.10	1.49E-01	-0.75	0.03	4.05E-04
AADAT	No	Yes	No	Down	0.60	1.83E-19	1.5E-19	-0.90	0.11	1.26E-02	-0.95	0.04	1.48E-05	-0.27	0.10	3.09E-01	-1.04	0.13	1.98E-03	-0.77	0.15	4.38E-02	-0.15	0.20	5.91E-01	-1.13	0.10	4.16E-04	-0.63	0.03	2.52E-03
ATP5D	No	Yes	No	Down	0.60	0.000108	0.00173	-0.78	0.11	2.16E-02	-0.36	0.04	3.71E-02	-1.12	0.11	5.55E-03	-1.35	0.14	1.50E-03	-0.52	0.14	4.31E-02	-0.71	0.21	2.99E-02	-0.96	0.10	1.03E-02	-0.04	0.03	8.33E-01
ZDHHC9	No	No	No	Down	0.60	4.44E-05	0.02336	-0.76	0.11	1.34E-02	-0.18	0.04	2.95E-01	-0.33	0.10	2.33E-01	-1.23	0.13	2.71E-03	-0.99	0.15	1.53E-03	-1.46	0.25	5.47E-04	-0.54	0.10	5.59E-02	-0.35	0.03	9.58E-02
NUDT13	No	Yes	No	Down	0.60	0.000266	5.2E-05	-0.85	0.11	1.16E-02	-0.65	0.04	5.26E-03	-1.00	0.11	7.17E-03	-0.87	0.12	1.03E-02	-0.66	0.14	4.51E-01	-0.29	0.20	7.37E-01	-0.93	0.10	1.54E-02	-0.59	0.03	1.68E-02
GSR	No	Yes	No	Down	0.60	3.58E-06	6.4E-05	-0.78	0.11	8.73E-03	-0.85	0.04	9.47E-05	-1.33	0.12	3.47E-04	-0.06	0.11	8.33E-01	-0.41	0.14	1.13E-01	-0.99	0.22	4.37E-02	-0.81	0.10	7.16E-03	-0.61	0.03	4.18E-03
TPPP3	No	Yes	No	Down	0.60	1.83E-19	1.5E-19	-0.94	0.11	3.71E-04	-0.76	0.04	7.95E-06	-0.67	0.11	7.62E-03	-0.32	0.11	2.26E-01	-1.01	0.15	1.84E-03	-0.70	0.21	6.11E-02	-0.86	0.10	9.21E-04	-0.55	0.03	1.20E-04
RSPRY1	No	Yes	No	Down	0.60	0.000321	0.01636	-0.63	0.10	6.05E-02	-0.51	0.04	3.34E-02	-1.38	0.12	6.40E-04	-0.74	0.12	1.31E-01	-0.73	0.14	2.68E-02	-0.59	0.21	8.55E-02	-0.52	0.10	7.70E-02	-0.71	0.03	1.70E-02
IRAK4	No	Yes	No	Down	0.61	0.002764	0.01306	-0.42	0.10	1.93E-01	-0.60	0.04	2.34E-02	-1.05	0.11	1.18E-02	-0.30	0.11	4.25E-01	-0.68	0.14	2.51E-01	-0.66	0.21	3.59E-01	-1.05	0.10	3.59E-02	-1.04	0.04	9.03E-04
ITGB7	No	Yes	No	Down	0.61	7.73E-05	0.00968	-0.43	0.10	1.45E-01	-0.75	0.04	1.63E-04	-1.15	0.11	1.58E-02	-1.17	0.13	1.17E-03	-0.53	0.14	1.30E-01	-0.80	0.22	6.66E-02	-0.61	0.10	1.48E-01	-0.35	0.03	5.70E-02
ANKRD54	No	Yes	No	Down	0.61	0.001093	0.01551	-0.75	0.11	4.16E-02	-0.72	0.04	2.80E-03	-0.35	0.10	3.54E-01	-1.42	0.14	8.81E-03	-0.36	0.14	1.88E-01	-0.68	0.21	1.15E-01	-0.82	0.10	3.70E-02	-0.68	0.03	2.88E-02
ARL15	No	Yes	No	Down	0.61	0.000519	0.00105	-1.05	0.11	3.84E-03	-0.83	0.04	3.58E-03	-1.00	0.11	5.54E-03	-0.21	0.11	5.29E-01	-0.36	0.14	5.11E-01	-0.77	0.21	4.73E-01	-0.68	0.10	3.91E-02	-0.89	0.03	3.46E-03
TMOD3	No	Yes	No	Down	0.61	0.000623	0.01999	-0.58	0.10	7.25E-02	-0.69	0.04	5.36E-03	-1.35	0.12	9.21E-04	-0.75	0.12	1.32E-01	-1.15	0.16	6.04E-02	-0.36	0.20	5.17E-01	-0.33	0.10	2.10E-01	-0.58	0.03	6.89E-03
SERPINA5	No	Yes	No	Down	0.61	1.83E-19	2E-05	-0.88	0.11	3.53E-04	-0.93	0.04	2.27E-07	-0.39	0.10	6.73E-02	-0.89	0.12	5.85E-04	-0.77	0.15	4.98E-03	-0.53	0.21	6.68E-02	-0.66	0.10	6.03E-03	-0.73	0.03	1.14E-06
METAP1	No	Yes	No	Down	0.61	4.19E-05	0.00698	-0.64	0.11	5.02E-02	-0.76	0.04	6.45E-04	-0.62	0.11	7.88E-02	-0.30	0.11	5.06E-01	-0.75	0.14	8.82E-03	-1.48	0.26	3.86E-04	-0.38	0.10	2.30E-01	-0.84	0.03	1.07E-02
C8ORF34	No	Yes	No	Down	0.61	8.51E-05	0.00093	-0.61	0.10	3.81E-02	-0.54	0.04	1.75E-03	-0.49	0.10	5.21E-02	-0.62	0.12	5.27E-02	-1.01	0.15	2.89E-02	-1.49	0.26	2.64E-02	-0.77	0.10	3.77E-03	-0.25	0.03	7.93E-02
NAT1	No	Yes	No	Down	0.61	3.58E-06	0.00066	-0.24	0.10	3.63E-01	-0.74	0.04	2.38E-04	-1.55	0.12	8.18E-06	-0.83	0.12	1.60E-02	-0.84	0.15	4.43E-02	-0.34	0.20	5.34E-01	-0.58	0.10	3.11E-02	-0.66	0.03	3.52E-03
USO1	No	No	No	Down	0.61	0.000125	validated	-1.13	0.11	2.14E-03	-0.54	0.04	1.83E-02	-0.42	0.10	1.83E-01	-0.26	0.11	5.01E-01	-1.35	0.16	8.53E-04	-0.89	0.22	2.36E-02	-0.67	0.10	1.84E-02	-0.52	0.03	7.25E-02
HEXB	No	Yes	No	Down	0.61	6.71E-06	0.00036	-0.12	0.10	6.43E-01	-0.67	0.04	5.44E-04	-0.91	0.11	1.10E-02	-0.03	0.11	9.26E-01	-0.36	0.14	1.10E-01	-1.82	0.28	2.82E-05	-0.95	0.10	1.65E-03	-0.91	0.03	5.53E-03
GNPNAT1	No	Yes	No	Down	0.61	0.00016	0.00214	-1.05	0.11	4.86E-03	-0.23	0.04	2.00E-01	-1.31	0.12	2.20E-03	-0.68	0.12	3.79E-02	-0.51	0.14	4.19E-01	-0.56	0.21	3.66E-01	-0.19	0.10	4.51E-01	-1.25	0.04	2.45E-05
CAPSL	No	Yes	No	Down	0.61	1.83E-19	2.6E-05	-0.33	0.10	1.29E-01	-0.80	0.04	3.41E-06	-0.41	0.10	5.54E-02	-0.77	0.12	6.05E-03	-1.13	0.16	7.81E-04	-0.82	0.22	2.81E-02	-0.82	0.10	1.04E-03	-0.70	0.03	2.04E-06
FECH	No	No	No	Down	0.61	0.000121	validated	-0.61	0.10	5.13E-02	-0.31	0.04	5.95E-02	-0.70	0.11	2.32E-02	-0.94	0.12	1.83E-02	-0.77	0.15	8.36E-02	-1.04	0.23	7.03E-02	-0.42	0.10	1.30E-01	-0.97	0.03	8.70E-05
CA5B	No	Yes	No	Down	0.61	0.001896	0.01405	-0.38	0.10	2.48E-01	-0.93	0.04	1.56E-04	-0.45	0.10	2.67E-01	-1.01	0.13	7.82E-03	-0.89	0.15	1.77E-01	-1.09	0.23	7.87E-02	-0.83	0.10	8.39E-02	-0.17	0.03	2.77E-01
FGL1	No	Yes	Yes	Down	0.61	1.83E-19	1.5E-19	-1.47	0.12	1.00E-05	-0.73	0.04	4.23E-05	-0.74	0.11	3.04E-02	-1.41	0.14	3.18E-05	-0.21	0.14	3.35E-01	-0.23	0.20	7.08E-01	-0.68	0.10	1.18E-02	-0.26	0.03	1.65E-01
KCNRG	No	Yes	No	Down	0.61	1.83E-19	1.5E-19	-0.52	0.10	4.97E-02	-0.84	0.04	3.23E-05	-0.64	0.11	1.59E-02	-0.75	0.12	2.91E-02	-0.82	0.15	1.86E-02	-0.55	0.21	2.96E-01	-0.92	0.10	1.70E-03	-0.70	0.03	6.84E-05
PBLD	No	Yes	No	Down	0.61	0.00034	0.00351	-0.59	0.10	3.86E-02	-0.81	0.04	9.93E-05	-0.67	0.11	1.35E-02	-1.21	0.13	1.99E-03	-0.46	0.14	3.24E-01	-1.64	0.27	1.23E-01	-0.18	0.10	4.45E-01	-0.17	0.03	3.35E-01
CYP2J2	No	Yes	No	Down	0.61	1.83E-19	8.6E-05	-0.91	0.11	6.40E-04	-0.67	0.04	1.10E-04	-0.42	0.10	1.05E-01	-0.65	0.12	2.07E-02	-0.68	0.14	1.80E-02	-0.76	0.21	3.39E-02	-1.17	0.10	3.48E-04	-0.48	0.03	3.21E-03
FAM110C	No	No	No	Down	0.61	1.83E-19	validated	-0.12	0.10	6.06E-01	-0.90	0.04	2.50E-06	-0.76	0.11	4.37E-03	-1.13	0.13	1.51E-04	-0.40	0.14	3.90E-01	-0.75	0.21	2.96E-01	-0.56	0.10	2.45E-02	-1.10	0.04	1.00E-20
MAN2A1	No	Yes	No	Down	0.61	2.85E-05	0.01492	-1.00	0.11	2.36E-03	-0.46	0.04	1.60E-02	-0.95	0.11	2.79E-03	-0.52	0.12	1.98E-01	-0.32	0.14	2.71E-01	-1.01	0.23	1.74E-02	-0.95	0.10	1.41E-03	-0.51	0.03	4.01E-02
CCDC17	No	Yes	No	Down	0.61	3.58E-06	0.00159	-0.71	0.11	5.31E-03	-0.47	0.04	2.56E-03	-0.65	0.11	1.79E-02	-1.10	0.13	6.39E-04	-0.60	0.14	5.60E-02	-1.01	0.23	1.98E-01	-0.61	0.10	1.98E-02	-0.58	0.03	9.08E-04
GREM2	No	Yes	No	Down	0.61	1.83E-19	1.5E-19	-0.97	0.11	1.99E-04	-0.71	0.04	1.23E-05	-0.89	0.11	8.71E-04	-0.55	0.12	2.22E-02	-0.73	0.14	3.36E-02	-0.81	0.22	4.75E-02	-0.79	0.10	1.60E-03	-0.24	0.03	7.82E-02
ARHGAP18	No	Yes	No	Down	0.61	6.71E-06	0.00092	-1.19	0.11	3.64E-04	-0.94	0.04	8.68E-05	-0.63	0.11	3.31E-02	-0.47	0.12	1.98E-01	-0.16	0.14	8.45E-01	-0.58	0.21	6.29E-01	-0.86	0.10	4.64E-03	-0.86	0.03	3.86E-04
STEAP2	No	Yes	No	Down	0.61	1.83E-19	1.3E-05	-1.39	0.12	3.32E-05	-0.76	0.04	7.72E-05	-1.12	0.11	2.67E-04	-0.58	0.12	3.33E-02	-0.22	0.14	4.05E-01	-0.66	0.21	1.26E-01	-0.45	0.10	8.16E-02	-0.51	0.03	8.92E-03
WDR38	No	Yes	No	Down	0.61	1.83E-19	0.00024	-0.41	0.10	9.35E-02	-0.83	0.04	1.64E-05	-0.65	0.11	1.89E-02	-1.03	0.13	3.53E-03	-0.75	0.14	1.59E-02	-0.74	0.21	1.31E-01	-0.69	0.10	1.26E-02	-0.59	0.03	6.02E-05
FXYD3	No	No	No	Down	0.61	1.83E-19	0.00398	-0.51	0.10	3.78E-02	-1.03	0.04	4.54E-07	-1.17	0.11	1.06E-04	-0.61	0.12	1.84E-02	-0.73	0.14	1.99E-01	-0.23	0.20	7.48E-01	-0.63	0.10	1.47E-02	-0.77	0.03	3.18E-06
SORL1	No	No	No	Down	0.61	3.58E-06	validated	-0.93	0.11	3.23E-03	-0.41	0.04	1.81E-02	-0.86	0.11	6.47E-03	-0.95	0.13	4.84E-03	-0.45	0.14	1.02E-01	-0.65	0.21	5.31E-02	-0.81	0.10	3.86E-03	-0.62	0.03	1.61E-03
ATP8B1	No	Yes	No	Down	0.61	1.83E-19	0.01222	-0.26	0.10	3.97E-01	-0.96	0.04	1.59E-04	-0.87	0.11	5.27E-03	-0.78	0.12	2.37E-02	-0.62	0.14	3.71E-01	-0.10	0.20	8.88E-01	-0.66	0.10	1.56E-02	-1.44	0.04	1.00E-20
YSK4	No	Yes	No	Down	0.61	3.58E-06	5.5E-05	-0.57	0.10	1.99E-02	-0.84	0.04	8.86E-06	-0.70	0.11	9.66E-03	-0.31	0.11	2.05E-01	-0.92	0.15	6.66E-02	-1.06	0.23	3.90E-01	-0.81	0.10	3.67E-03	-0.46	0.03	1.00E-03
ZRSR2	No	Yes	No	Down	0.61	0.000829	0.00077	-0.58	0.10	1.23E-01	-0.41	0.04	3.17E-02	-0.94	0.11	4.29E-02	-1.19	0.13	3.01E-02	-0.02	0.14	8.70E-01	-0.48	0.21	1.43E-01	-1.51	0.11	5.79E-05	-0.53	0.03	1.21E-01
ATP6V1C2	No	Yes	No	Down	0.61	1.83E-19	0.00154	-0.10	0.10	6.47E-01	-0.97	0.04	4.54E-07	-0.56	0.11	2.03E-02	-1.05	0.13	2.93E-04	-0.59	0.14	1.21E-01	-0.78	0.22	1.33E-01	-0.79	0.10	1.27E-03	-0.83	0.03	6.82E-07
MAPK13	No	Yes	No	Down	0.61	2.40E-05	0.01365	-0.51	0.10	8.53E-02	-0.65	0.04	7.59E-04	-0.34	0.10	2.27E-01	-0.81	0.12	1.68E-02	-1.10	0.15	1.06E-03	-1.30	0.24	2.70E-03	-0.53	0.10	8.52E-02	-0.43	0.03	5.92E-02
SCGB3A1	No	Yes	No	Down	0.61	1.83E-19	1.5E-05	-1.03	0.11	1.56E-04	-0.73	0.04	5.91E-06	-0.63	0.11	7.58E-03	-0.74	0.12	4.43E-03	-0.30	0.14	1.62E-01	-0.83	0.22	2.06E-02	-0.50	0.10	3.30E-02	-0.89	0.03	1.00E-20
CXORF41	No	Yes	No	Down	0.61	3.58E-06	2E-05	-0.57	0.10	1.94E-02	-0.74	0.04	7.95E-05	-0.84	0.11	4.73E-03	-0.66	0.12	1.79E-02	-0.84	0.15	2.50E-01	-0.69	0.21	6.51E-01	-0.73	0.10	7.29E-03	-0.58	0.03	2.31E-04
SLC43A1	No	Yes	No	Down	0.61	1.83E-19	1.5E-19	-1.16	0.11	5.59E-05	-0.91	0.04	3.18E-06	-0.48	0.10	4.78E-02	-0.79	0.12	6.48E-03	-0.76	0.15	2.93E-01	-0.58	0.21	5.73E-01	-0.54	0.10	3.13E-02	-0.42	0.03	2.99E-02
ENDOG	No	Yes	No	Down	0.61	3.83E-05	0.00487	-0.75	0.11	2.15E-02	-0.70	0.04	1.52E-03	-0.67	0.11	4.58E-02	-0.12	0.11	7.74E-01	-1.02	0.15	1.04E-02	-0.89	0.22	4.17E-02	-0.83	0.10	1.17E-02	-0.67	0.03	1.31E-03
C15ORF26	No	Yes	No	Down	0.61	1.83E-19	6.7E-06	-1.01	0.11	1.71E-04	-0.84	0.04	8.86E-06	-0.57	0.11	3.44E-02	-0.67	0.12	1.57E-02	-0.91	0.15	8.69E-03	-0.59	0.21	1.85E-01	-0.58	0.10	4.62E-02	-0.47	0.03	1.90E-03
ARRDC4	No	Yes	No	Down	0.61	1.14E-05	0.00016	-0.63	0.10	3.22E-02	-1.00	0.04	8.86E-06	-1.01	0.11	8.33E-04	-0.56	0.12	1.30E-01	-0.40	0.14	2.44E-01	-0.96	0.22	5.58E-02	-0.72	0.10	8.94E-03	-0.33	0.03	9.91E-02
FZD3	No	Yes	No	Down	0.62	3.58E-06	0.01014	-1.15	0.11	6.42E-04	-0.62	0.04	2.41E-03	-0.92	0.11	2.87E-03	-0.38	0.12	1.67E-01	-0.87	0.15	1.23E-02	-0.47	0.21	1.65E-01	-0.53	0.10	3.84E-02	-0.68	0.03	1.21E-03
OVGP1	No	Yes	No	Down	0.62	1.83E-19	1.5E-19	-0.87	0.11	5.06E-04	-0.63	0.04	4.45E-05	-0.68	0.11	6.25E-03	-1.24	0.13	3.98E-05	-0.69	0.14	8.61E-03	-0.48	0.21	8.62E-02	-0.48	0.10	3.05E-02	-0.53	0.03	5.68E-04
IDE	No	No	No	Down	0.62	2.40E-05	validated	-0.20	0.10	5.32E-01	-0.89	0.04	1.91E-04	-1.25	0.12	2.06E-03	-0.95	0.12	2.36E-02	-0.31	0.14	3.05E-01	-0.08	0.20	7.26E-01	-1.13	0.10	1.02E-03	-0.78	0.03	3.77E-03
CCNA1	No	Yes	No	Down	0.62	1.83E-19	0.00057	-0.82	0.11	8.44E-04	-0.96	0.04	6.82E-07	-0.48	0.10	3.10E-02	-0.46	0.12	6.64E-02	-0.80	0.15	5.90E-03	-0.95	0.22	7.67E-03	-0.63	0.10	6.50E-03	-0.50	0.03	8.23E-04
DECR2	No	Yes	No	Down	0.62	0.000113	0.00037	-0.73	0.11	4.60E-02	-0.62	0.04	2.13E-03	-0.83	0.11	4.70E-02	-1.61	0.15	3.28E-04	-0.24	0.14	3.16E-01	-0.66	0.21	9.83E-02	-0.21	0.10	4.98E-01	-0.69	0.03	3.43E-03
CCNH	No	Yes	No	Down	0.62	0.022689	0.0253	-0.82	0.11	6.89E-02	-0.44	0.04	5.51E-02	-1.10	0.11	1.15E-02	-0.43	0.12	3.02E-01	-0.64	0.14	1.77E-01	-1.42	0.25	7.74E-02	-0.20	0.10	4.62E-01	-0.54	0.03	1.40E-01
SUDS3	No	No	No	Down	0.62	0.00314	validated	-0.86	0.11	4.32E-02	-0.40	0.04	9.04E-02	-0.59	0.11	9.76E-02	-1.41	0.14	1.16E-02	-0.46	0.14	5.62E-01	-0.42	0.20	5.84E-01	-0.55	0.10	5.30E-02	-0.86	0.03	1.20E-03
TRAPPC5	No	Yes	No	Down	0.62	0.002216	0.00947	-0.61	0.10	5.50E-02	-0.28	0.04	1.42E-01	-0.56	0.11	1.56E-01	-1.10	0.13	4.63E-02	-0.29	0.14	2.08E-01	-1.53	0.26	7.83E-04	-0.56	0.10	1.12E-01	-0.62	0.03	3.77E-02
ENPP4	No	Yes	No	Down	0.62	9.89E-06	0.00073	-0.72	0.11	1.85E-02	-0.89	0.04	4.66E-05	-0.84	0.11	6.59E-03	-0.25	0.11	3.88E-01	-0.87	0.15	1.26E-02	-0.73	0.21	5.53E-02	-0.70	0.10	1.11E-02	-0.55	0.03	1.45E-02
ZNF524	No	Yes	No	Down	0.62	0.000754	0.00405	-1.21	0.11	1.26E-03	-0.58	0.04	6.83E-03	-0.67	0.11	1.74E-01	-1.28	0.13	4.76E-03	-0.30	0.14	2.67E-01	-1.22	0.24	1.13E-02	-0.01	0.10	9.83E-01	-0.27	0.03	1.59E-01
ASCL2	No	No	No	Down	0.62	1.83E-19	validated	-0.57	0.10	2.51E-02	-0.70	0.04	4.86E-05	-0.62	0.11	1.72E-02	-0.39	0.12	1.34E-01	-0.73	0.14	9.59E-03	-1.44	0.25	3.90E-04	-0.70	0.10	6.51E-03	-0.41	0.03	7.98E-03
ALDH18A1	No	Yes	No	Down	0.62	0.000381	0.00133	-0.66	0.11	8.85E-02	-0.60	0.04	6.33E-03	-1.00	0.11	8.89E-03	-1.01	0.13	2.63E-02	-0.34	0.14	1.55E-01	-1.05	0.23	6.90E-03	-0.42	0.10	1.20E-01	-0.46	0.03	4.54E-02
RCAN3	No	No	No	Down	0.62	0.000246	validated	-0.75	0.11	2.43E-02	-0.25	0.04	1.49E-01	-0.63	0.11	5.26E-02	-1.10	0.13	2.38E-03	-0.86	0.15	2.52E-01	-0.70	0.21	3.65E-01	-0.57	0.10	4.32E-02	-0.68	0.03	1.87E-04
C6ORF165	No	No	No	Down	0.62	1.83E-19	validated	-0.26	0.10	2.49E-01	-0.73	0.04	2.61E-05	-0.33	0.10	2.04E-01	-1.06	0.13	1.18E-03	-0.98	0.15	6.08E-03	-0.79	0.22	8.80E-02	-0.74	0.10	1.65E-02	-0.63	0.03	1.68E-04
ANG	No	Yes	No	Down	0.62	1.83E-19	1.5E-19	-1.52	0.12	3.18E-06	-1.00	0.04	2.50E-06	-0.87	0.11	4.28E-03	-0.43	0.12	1.09E-01	-0.30	0.14	1.86E-01	-0.08	0.20	6.40E-01	-0.75	0.10	1.14E-02	-0.57	0.03	1.46E-03
AP1M2	No	No	No	Down	0.62	1.83E-19	validated	-0.39	0.10	2.47E-01	-0.70	0.04	1.75E-04	-0.65	0.11	3.72E-02	-1.14	0.13	7.03E-04	-0.69	0.14	1.85E-01	-0.44	0.20	3.20E-01	-0.43	0.10	1.18E-01	-1.08	0.04	1.00E-20
RPS6KA5	No	Yes	No	Down	0.62	1.83E-19	1.5E-19	-1.19	0.11	1.97E-04	-1.02	0.04	3.18E-06	-0.38	0.10	1.37E-01	-0.08	0.11	7.73E-01	-0.75	0.14	3.64E-02	-0.66	0.21	1.09E-01	-1.40	0.11	3.02E-05	-0.02	0.03	8.87E-01
TCN1	No	Yes	Yes	Down	0.62	1.83E-19	1.5E-19	-0.30	0.10	1.89E-01	-0.80	0.04	9.31E-06	-0.84	0.11	8.12E-04	-0.69	0.12	1.22E-02	-0.84	0.15	1.11E-02	-0.92	0.22	3.72E-02	-0.43	0.10	7.14E-02	-0.69	0.03	1.57E-05

Supplementary Table 2. Details for 2,053 genes differentially expressed between EEC and NEEC, identified by microarray meta-analysis.

EEC - endometrioid endometrial cancer, NEEC - non-endometrioid endometrial cancer, SMD - Standardised Mean Difference, FDR - False Discovery Rate, Var - Variance

Gene	145 gene list	1253 gene list	Associated with EEC-specific survival?	Up/Down Regulated in NEEC	average standardised fold change	Microarray Meta FDR	RNASeq FDR	TCGA SMD	TCGA Var	TCGA P-value	E-MTAB-2532 SMD	E-MTAB-2532 Var	E-MTAB-2532 P-value	E-GEOD-56026 SMD	E-GEOD-56026 Var	E-GEOD-56026 P-value	GSE32507 SMD	GSE32507 Var	GSE32507 P-value	GSE24537 SMD	GSE24537 Var	GSE24537 P-value	E-GEOD-23518 SMD	E-GEOD-23518 Var	E-GEOD-23518 P-value	E-GEOD-17025 SMD	E-GEOD-17025 Var	E-GEOD-17025 P-value	E-GEOD-2109 SMD	E-GEOD-2109 Var	E-GEOD-2109 P-value
APOO	No	Yes	No	Down	0.62	0.000381	0.00904	-0.93	0.11	9.62E-03	-0.67	0.04	2.57E-03	-0.80	0.11	1.67E-02	-0.30	0.11	4.07E-01	-0.78	0.15	2.87E-02	-0.84	0.22	4.93E-02	-0.70	0.10	3.71E-02	-0.48	0.03	8.46E-02
NPAS3	No	Yes	No	Down	0.62	1.83E-19	0.0003	-0.37	0.10	9.35E-02	-0.90	0.04	3.18E-06	-0.49	0.10	2.49E-02	-0.77	0.12	3.69E-03	-1.09	0.15	1.98E-03	-0.32	0.20	2.71E-01	-0.82	0.10	5.65E-04	-0.74	0.03	3.86E-06
ZFYVE19	No	Yes	No	Down	0.62	0.003193	0.00286	-0.19	0.10	6.40E-01	-0.73	0.04	1.46E-03	-0.44	0.10	2.56E-01	-1.14	0.13	2.10E-02	-0.93	0.15	3.22E-02	-0.78	0.22	7.38E-02	-0.95	0.10	3.98E-02	-0.34	0.03	2.04E-01
ART3	No	Yes	Yes	Down	0.62	2.19E-05	1.5E-19	-1.33	0.12	2.89E-05	-0.86	0.04	5.64E-04	-0.82	0.11	5.77E-03	-0.07	0.11	7.74E-01	-0.72	0.14	4.61E-02	-0.86	0.22	4.09E-02	-0.81	0.10	2.50E-02	-0.02	0.03	8.69E-01
CLCN3	No	Yes	No	Down	0.62	0.003014	0.03619	-1.21	0.11	4.47E-03	-0.19	0.04	3.65E-01	-0.40	0.10	2.47E-01	-1.49	0.14	7.86E-03	-0.59	0.14	1.28E-01	-0.67	0.21	1.04E-01	-0.24	0.10	3.40E-01	-0.70	0.03	5.78E-03
DIO2	No	Yes	Yes	Down	0.62	3.58E-06	1.5E-19	-0.94	0.11	7.75E-04	-0.62	0.04	1.47E-04	-0.21	0.10	3.20E-01	-0.80	0.12	5.66E-03	-1.44	0.17	4.62E-04	-1.05	0.23	1.34E-02	-0.06	0.10	7.55E-01	-0.38	0.03	1.46E-02
SPINK4	No	Yes	No	Down	0.62	8.71E-05	1.5E-19	-1.48	0.12	3.18E-06	-0.91	0.04	6.93E-05	-0.32	0.10	3.68E-01	-0.27	0.11	2.67E-01	-1.05	0.15	1.68E-01	-1.06	0.23	7.29E-02	-0.24	0.10	5.15E-01	-0.16	0.03	3.13E-01
SH3RF1	No	No	No	Down	0.62	9.28E-05	validated	-0.48	0.10	1.61E-01	-0.87	0.04	1.63E-04	-0.20	0.10	4.90E-01	-0.67	0.12	6.76E-02	-1.26	0.16	1.28E-02	-0.38	0.20	3.19E-01	-0.80	0.10	2.16E-02	-0.82	0.03	6.29E-04
C9ORF117	No	Yes	No	Down	0.62	1.83E-19	0.00063	-0.81	0.11	8.84E-03	-0.69	0.04	4.77E-05	-0.85	0.11	4.68E-03	-1.27	0.13	5.38E-05	-0.42	0.14	8.71E-02	-0.50	0.21	2.89E-01	-0.49	0.10	4.57E-02	-0.44	0.03	6.26E-03
CYB5D2	No	Yes	No	Down	0.62	5.41E-05	0.00911	-0.54	0.10	8.78E-02	-1.01	0.04	6.41E-05	-0.46	0.10	1.45E-01	-1.07	0.13	2.03E-02	-0.62	0.14	6.05E-02	-0.25	0.20	3.35E-01	-0.69	0.10	5.49E-02	-0.82	0.03	1.06E-03
TRIP4	No	No	No	Down	0.62	0.001779	validated	-0.18	0.10	6.24E-01	-0.92	0.04	9.08E-04	-0.99	0.11	7.60E-03	-0.74	0.12	1.74E-01	-0.98	0.15	1.59E-02	-0.51	0.21	2.03E-01	-0.64	0.10	5.72E-02	-0.52	0.03	1.65E-01
MPND	No	Yes	No	Down	0.62	0.000159	1.5E-19	-0.93	0.11	2.72E-02	-0.37	0.04	6.89E-02	-0.77	0.11	3.07E-02	-0.50	0.12	2.22E-01	-0.28	0.14	5.46E-01	-0.77	0.21	2.77E-01	-0.81	0.10	1.19E-02	-1.04	0.04	5.91E-06
EGLN3	No	Yes	No	Down	0.62	1.83E-19	0.00015	-1.16	0.11	2.02E-04	-0.91	0.04	3.41E-06	-0.47	0.10	6.21E-02	-0.02	0.11	9.34E-01	-0.62	0.14	2.64E-01	-1.29	0.24	1.20E-01	-0.11	0.10	6.08E-01	-0.87	0.03	7.27E-06
PON2	No	No	No	Down	0.62	0.000143	validated	-0.76	0.11	2.03E-02	-0.26	0.04	1.56E-01	-0.37	0.10	1.74E-01	-0.77	0.12	1.25E-01	-0.94	0.15	1.84E-03	-1.84	0.28	2.00E-05	-0.30	0.10	2.72E-01	-0.22	0.03	1.62E-01
KLK12	No	Yes	No	Down	0.62	1.83E-19	1.5E-19	-1.73	0.13	1.00E-20	-0.69	0.04	4.75E-05	-0.55	0.11	8.22E-02	-0.73	0.12	3.26E-03	-0.57	0.14	9.88E-02	-0.81	0.22	1.31E-01	-0.23	0.10	4.40E-01	-0.15	0.03	3.03E-01
TSNAXIP1	No	Yes	No	Down	0.62	6.26E-05	0.00044	-0.86	0.11	1.53E-03	-0.47	0.04	5.19E-03	-0.64	0.11	6.69E-02	-0.97	0.13	4.61E-03	-0.98	0.15	8.74E-02	-0.17	0.20	8.06E-01	-0.97	0.10	2.97E-03	-0.41	0.03	4.71E-02
SLCO2A1	No	Yes	No	Down	0.62	1.83E-19	3.8E-05	-0.73	0.11	3.79E-03	-0.86	0.04	8.86E-06	-0.61	0.11	2.04E-02	-1.14	0.13	3.69E-04	-0.15	0.14	3.79E-01	-0.61	0.21	4.42E-02	-0.63	0.10	1.33E-02	-0.71	0.03	6.04E-04
F3	No	No	No	Down	0.62	1.83E-19	0.0124	-0.30	0.10	2.12E-01	-0.68	0.04	1.81E-04	-0.59	0.11	1.63E-02	-1.06	0.13	1.40E-03	-0.31	0.14	2.32E-01	-1.24	0.24	7.28E-03	-0.47	0.10	4.68E-02	-0.79	0.03	9.31E-06
ZNF19	No	Yes	No	Down	0.62	0.026533	0.03034	-0.13	0.10	6.91E-01	-0.49	0.04	9.97E-02	-0.57	0.11	1.56E-01	-1.71	0.15	6.34E-04	-0.68	0.14	6.06E-01	-0.62	0.21	6.17E-01	-1.07	0.10	2.38E-02	-0.18	0.03	2.94E-01
AHCYL1	No	No	No	Down	0.62	0.000797	validated	-0.67	0.11	8.50E-02	-0.66	0.04	2.24E-03	-0.23	0.10	4.54E-01	-1.91	0.16	1.22E-03	-0.50	0.14	5.48E-02	-0.93	0.22	9.31E-03	-0.44	0.10	1.21E-01	-0.10	0.03	6.13E-01
TPST2	No	Yes	No	Down	0.62	1.14E-05	0.00157	-0.43	0.10	1.16E-01	-0.74	0.04	4.44E-04	-0.25	0.10	3.69E-01	-1.03	0.13	1.59E-02	-0.61	0.14	2.46E-02	-0.62	0.21	5.04E-02	-1.20	0.10	1.19E-04	-0.57	0.03	2.71E-02
EFCAB6	No	No	No	Down	0.62	6.71E-06	validated	-0.43	0.10	9.83E-02	-0.58	0.04	1.72E-03	-0.39	0.10	2.30E-01	-0.92	0.12	2.26E-03	-0.62	0.14	6.83E-02	-0.87	0.22	1.88E-01	-0.89	0.10	7.58E-03	-0.74	0.03	1.48E-05
NHS	No	Yes	No	Down	0.62	6.71E-06	0.00084	-1.13	0.11	2.66E-04	-0.72	0.04	4.55E-04	-0.97	0.11	2.60E-03	-0.26	0.11	3.61E-01	-0.62	0.14	8.38E-02	-0.78	0.22	8.12E-02	-0.35	0.10	1.48E-01	-0.60	0.03	1.85E-03
PIGN	No	No	No	Down	0.62	0.00066	validated	-0.93	0.11	1.80E-02	-0.35	0.04	1.21E-01	-0.54	0.11	8.34E-02	-0.95	0.12	2.17E-02	-0.85	0.15	1.67E-02	-0.41	0.20	2.55E-01	-0.58	0.10	6.75E-02	-0.84	0.03	2.12E-03
DNAL4	No	No	No	Down	0.62	2.85E-05	validated	-0.58	0.10	8.23E-02	-0.73	0.04	9.49E-04	-0.20	0.10	5.22E-01	-0.22	0.11	6.24E-01	-1.42	0.17	1.37E-04	-0.72	0.21	3.05E-02	-0.77	0.10	5.54E-02	-0.79	0.03	1.08E-03
PRKAB1	No	Yes	No	Down	0.62	0.000415	0.02874	-0.15	0.10	5.76E-01	-0.67	0.04	2.23E-03	-0.60	0.11	6.63E-02	-0.66	0.12	9.12E-02	-0.94	0.15	9.40E-03	-1.46	0.25	1.43E-03	-0.67	0.10	5.30E-02	-0.28	0.03	1.62E-01
REEP6	No	Yes	No	Down	0.62	1.14E-05	0.00028	-1.41	0.12	2.93E-05	-0.71	0.04	1.69E-04	-0.42	0.10	1.56E-01	-1.07	0.13	2.94E-03	-0.66	0.14	9.26E-02	-0.32	0.20	3.93E-01	-0.60	0.10	6.85E-02	-0.24	0.03	1.23E-01
IQUB	No	Yes	No	Down	0.63	5.41E-05	0.00024	-0.67	0.11	1.37E-02	-0.79	0.04	2.17E-04	-0.62	0.11	2.47E-02	-0.89	0.12	3.76E-03	-0.98	0.15	4.95E-02	-0.49	0.21	4.38E-01	-0.72	0.10	2.38E-02	-0.27	0.03	1.25E-01
ENOSF1	No	No	No	Down	0.63	0.00011	0.04583	-0.71	0.11	2.38E-02	-0.48	0.04	1.32E-02	-0.49	0.10	8.53E-02	-1.58	0.15	2.24E-04	-0.94	0.15	1.20E-02	-0.32	0.20	3.69E-01	-0.41	0.10	1.01E-01	-0.48	0.03	2.79E-02
GLB1	No	No	No	Down	0.63	3.97E-05	validated	-0.14	0.10	6.39E-01	-0.44	0.04	1.25E-02	-0.54	0.11	1.07E-01	-0.88	0.12	3.82E-02	-0.93	0.15	2.02E-03	-1.35	0.25	5.88E-04	-0.21	0.10	4.03E-01	-0.92	0.03	6.53E-04
CCT6B	No	No	No	Down	0.63	0.002197	validated	-0.41	0.10	1.38E-01	-0.72	0.04	1.00E-03	-0.47	0.10	1.06E-01	-0.31	0.11	3.64E-01	-1.30	0.16	6.29E-02	-0.84	0.22	2.86E-01	-0.62	0.10	3.97E-02	-0.73	0.03	9.80E-03
CCDC104	No	No	No	Down	0.63	0.000345	validated	-0.50	0.10	1.30E-01	-0.65	0.04	2.84E-03	-0.43	0.10	2.14E-01	-0.96	0.13	3.70E-02	-0.61	0.14	6.83E-02	-0.44	0.20	1.85E-01	-1.08	0.10	2.33E-03	-0.74	0.03	7.25E-03
MRPS27	No	Yes	No	Down	0.63	0.001158	0.03341	-1.20	0.11	4.48E-03	-0.52	0.04	2.07E-02	-1.13	0.11	6.53E-03	-0.61	0.12	1.78E-01	-0.55	0.14	5.70E-02	-0.22	0.20	3.78E-01	-0.71	0.10	2.88E-02	-0.45	0.03	1.45E-01
VAMP8	No	No	No	Down	0.63	3.58E-06	validated	-0.08	0.10	8.20E-01	-0.60	0.04	2.71E-03	-0.23	0.10	5.08E-01	-0.52	0.12	1.67E-01	-0.64	0.14	1.59E-02	-1.29	0.24	6.49E-04	-1.13	0.10	7.16E-05	-0.91	0.03	1.80E-04
THOC7	No	No	No	Down	0.63	0.001055	validated	-0.60	0.10	1.99E-01	-0.29	0.04	2.27E-01	-0.38	0.10	3.32E-01	-0.24	0.11	6.35E-01	-1.16	0.16	7.30E-04	-1.35	0.25	7.78E-04	-0.62	0.10	3.44E-02	-0.75	0.03	4.13E-02
TFF1	No	Yes	No	Down	0.63	1.83E-19	1.5E-19	-0.81	0.11	1.37E-02	-0.74	0.04	8.86E-06	-0.66	0.11	7.75E-03	-0.80	0.12	1.92E-03	-0.49	0.14	2.31E-01	-0.87	0.22	1.24E-01	-0.48	0.10	5.45E-02	-0.55	0.03	8.60E-04
LEF1	No	Yes	No	Down	0.63	1.83E-19	1.5E-19	-1.19	0.11	4.13E-05	-0.40	0.04	1.05E-02	-0.70	0.11	7.30E-03	-0.71	0.12	1.00E-02	-0.99	0.15	1.32E-03	-0.58	0.21	5.59E-02	-0.73	0.10	3.93E-03	-0.10	0.03	5.30E-01
RAB11FIP1	No	Yes	No	Down	0.63	1.83E-19	0.00174	-0.43	0.10	1.13E-01	-0.82	0.04	3.52E-05	-0.85	0.11	6.57E-03	-0.70	0.12	3.35E-02	-0.45	0.14	6.34E-02	-0.88	0.22	1.03E-02	-0.37	0.10	1.24E-01	-0.87	0.03	4.20E-05
LIMS3	No	No	No	Down	0.63	3.58E-06	0.01736	-0.62	0.10	6.34E-03	-0.43	0.04	2.70E-03	-0.63	0.11	1.02E-02	-1.27	0.13	1.89E-05	-0.87	0.15	3.40E-01	-0.72	0.21	4.10E-01	-0.26	0.10	1.96E-01	-0.57	0.03	4.32E-04
STRBP	No	No	No	Down	0.63	0.000526	validated	-0.71	0.11	7.43E-02	-0.65	0.04	5.26E-03	-0.08	0.10	7.39E-01	-0.35	0.11	3.81E-01	-1.34	0.16	3.01E-03	-1.09	0.23	1.92E-02	-0.82	0.10	3.85E-03	-0.32	0.03	5.79E-02
PPAPDC1B	No	Yes	No	Down	0.63	6.71E-06	1.5E-19	-0.62	0.10	5.47E-02	-1.09	0.04	2.68E-05	-1.06	0.11	2.78E-03	-0.11	0.11	7.29E-01	-0.90	0.15	2.21E-02	-0.15	0.20	5.66E-01	-0.58	0.10	5.39E-02	-0.87	0.03	1.15E-04
SIRT6	No	Yes	No	Down	0.63	0.049082	0.00015	-1.12	0.11	1.32E-02	-0.42	0.04	7.93E-02	-0.80	0.11	8.76E-02	-1.52	0.14	1.18E-02	-0.28	0.14	6.90E-01	-0.68	0.21	4.90E-01	-0.43	0.10	4.07E-01	-0.12	0.03	6.45E-01
TNFRSF10B	No	Yes	No	Down	0.63	0.000175	1.5E-19	-0.56	0.10	8.10E-02	-0.25	0.04	1.56E-01	-0.94	0.11	5.19E-03	-1.03	0.13	2.16E-03	-0.63	0.14	3.84E-02	-1.41	0.25	1.33E-03	-0.17	0.10	5.18E-01	-0.36	0.03	4.82E-02
C4BPB	No	Yes	No	Down	0.63	6.71E-06	1.5E-19	-0.54	0.10	3.51E-02	-0.98	0.04	3.86E-06	-1.12	0.11	1.11E-03	-0.68	0.12	1.37E-02	-0.23	0.14	4.12E-01	-0.94	0.22	1.00E-01	-0.44	0.10	1.76E-01	-0.42	0.03	1.05E-02
DUSP6	No	Yes	No	Down	0.63	0.000586	1.5E-19	-0.55	0.10	4.31E-02	-0.30	0.04	6.89E-02	-0.44	0.10	8.88E-02	-0.88	0.12	1.26E-02	-0.70	0.14	6.02E-02	-1.83	0.28	3.29E-03	-0.16	0.10	4.78E-01	-0.50	0.03	5.58E-03
SERPINA4	No	Yes	No	Down	0.63	6.71E-06	1.5E-19	-0.96	0.11	1.73E-03	-0.85	0.04	6.45E-05	-0.77	0.11	1.38E-02	-1.31	0.14	1.06E-04	-0.33	0.14	7.34E-01	-0.25	0.20	7.96E-01	-0.47	0.10	1.47E-01	-0.42	0.03	1.49E-02
ALDH3B2	No	No	No	Down	0.63	1.83E-19	0.01519	-0.12	0.10	6.52E-01	-0.94	0.04	9.54E-05	-0.39	0.10	1.04E-01	-0.83	0.12	2.70E-02	-0.80	0.15	6.80E-03	-0.64	0.21	6.52E-02	-1.01	0.10	5.88E-04	-0.61	0.03	8.45E-05
FUCA1	No	Yes	No	Down	0.63	2.85E-05	0.00175	-0.57	0.10	8.43E-02	-0.49	0.04	1.03E-02	-0.65	0.11	4.84E-02	-0.18	0.11	6.51E-01	-0.76	0.15	9.92E-03	-1.40	0.25	8.80E-04	-0.25	0.10	3.20E-01	-1.05	0.04	8.36E-05
ACSM3	No	No	No	Down	0.63	1.83E-19	validated	-0.36	0.10	1.53E-01	-0.80	0.04	8.00E-05	-0.99	0.11	5.18E-04	-1.16	0.13	5.10E-04	-0.32	0.14	1.60E-01	-0.75	0.21	4.49E-02	-0.37	0.10	1.40E-01	-0.58	0.03	3.38E-04
PCBD1	No	No	No	Down	0.63	0.00021	validated	-0.04	0.10	9.14E-01	-0.21	0.04	2.83E-01	-0.79	0.11	2.12E-02	-1.19	0.13	6.59E-03	-0.63	0.14	2.12E-02	-1.41	0.25	6.02E-04	-0.29	0.10	2.55E-01	-0.79	0.03	2.19E-03
SERPINB1	No	No	No	Down	0.63	1.14E-05	0.01955	-0.81	0.11	6.84E-03	-0.59	0.04	1.58E-03	-1.04	0.11	6.80E-04	-0.97	0.13	9.63E-03	-0.46	0.14	6.23E-02	-0.85	0.22	1.50E-02	-0.36	0.10	1.19E-01	-0.25	0.03	1.23E-01
MIA2	No	Yes	No	Down	0.63	0.001424	0.00585	-0.83	0.11	3.24E-03	-0.61	0.04	2.02E-03	-0.48	0.10	1.32E-01	-1.08	0.13	2.07E-04	-0.68	0.14	4.82E-01	-1.53	0.26	4.42E-01	-0.01	0.10	9.78E-01	-0.12	0.03	3.91E-01
AKAP14	No	Yes	No	Down	0.63	1.83E-19	0.00151	-0.46	0.10	4.18E-02	-0.79	0.04	1.41E-05	-0.72	0.11	3.60E-03	-0.56	0.12	3.44E-02	-0.76	0.15	1.51E-02	-0.77	0.21	1.54E-01	-0.80	0.10	1.25E-03	-0.48	0.03	7.05E-04
CDCP1	No	No	No	Down	0.63	1.14E-05	validated	-0.13	0.10	6.68E-01	-0.96	0.04	5.45E-06	-0.58	0.11	4.20E-02	-0.48	0.12	8.81E-02	-0.63	0.14	5.15E-02	-1.91	0.29	1.50E-04	-0.19	0.10	4.78E-01	-0.44	0.03	1.50E-02
ABCA7	No	Yes	No	Down	0.63	0.000623	1.5E-19	-0.97	0.11	2.95E-03	-0.33	0.04	4.58E-02	-1.07	0.11	7.74E-03	-0.87	0.12	1.62E-02	-0.25	0.14	2.89E-01	-0.76	0.21	1.36E-01	-0.66	0.10	2.94E-02	-0.40	0.03	1.09E-01
OXSM	No	Yes	No	Down	0.63	0.008151	0.0296	-0.45	0.10	2.24E-01	-0.83	0.04	1.39E-03	-0.64	0.11	8.06E-02	-1.57	0.15	1.29E-02	-0.32	0.14	4.95E-01	-0.39	0.20	4.46E-01	-0.34	0.10	2.93E-01	-0.74	0.03	4.07E-02
THOP1	No	Yes	No	Down	0.63	0.006951	0.00069	-0.91	0.11	1.55E-02	-0.14	0.04	4.49E-01	-0.92	0.11	3.09E-02	-1.13	0.13	9.20E-03	-0.43	0.14	1.44E-01	-1.07	0.23	2.66E-02	-0.41	0.10	2.82E-01	-0.28	0.03	2.74E-01
C9ORF24	No	Yes	No	Down	0.63	1.83E-19	0.00035	-0.57	0.10	1.02E-02	-0.68	0.04	1.36E-05	-0.55	0.11	1.36E-02	-0.36	0.11	1.39E-01	-0.95	0.15	1.41E-03	-0.79	0.22	2.37E-02	-0.66	0.10	6.35E-03	-0.71	0.03	1.59E-06
WFS1	No	No	No	Down	0.63	3.58E-06	0.01407	-0.93	0.11	2.60E-03	-0.81	0.04	1.68E-04	-0.09	0.10	7.51E-01	-1.04	0.13	8.86E-03	-0.19	0.14	2.99E-01	-0.43	0.20	1.16E-01	-1.11	0.10	8.20E-04	-0.68	0.03	8.33E-04
NEK10	No	Yes	No	Down	0.63	0.000191	0.00013	-0.74	0.11	1.01E-02	-0.66	0.04	4.09E-04	-0.64	0.11	9.03E-02	-0.60	0.12	4.31E-02	-1.22	0.16	4.24E-01	-0.24	0.20	8.51E-01	-0.49	0.10	1.31E-01	-0.69	0.03	3.08E-04
MYO1E	No	Yes	No	Down	0.63	0.003392	0.0005	-0.39	0.10	2.84E-01	-0.92	0.04	6.27E-05	-0.60	0.11	9.25E-02	-0.65	0.12	1.02E-01	-1.08	0.15	3.21E-01	-0.47	0.21	6.01E-01	-0.51	0.10	1.55E-01	-0.65	0.03	2.21E-02
CCDC78	No	Yes	No	Down	0.63	1.83E-19	0.00296	-0.84	0.11	9.17E-04	-0.61	0.04	1.50E-04	-0.39	0.10	1.23E-01	-0.93	0.12	1.74E-03	-0.50	0.14	1.47E-01	-0.78	0.22	3.30E-01	-0.58	0.10	2.83E-02	-0.64	0.03	1.33E-04
NDFIP1	No	No	No	Down	0.63	0.004961	validated	-0.73	0.11	8.55E-02	-0.24	0.04	1.76E-01	-0.71	0.11	7.35E-02	-0.34	0.11	4.00E-01	-1.01	0.15	2.98E-02	-0.70	0.21	1.95E-01	-0.57	0.10	3.48E-02	-0.98	0.03	3.01E-03
AR	No	No	No	Down	0.63	1.83E-19	validated	-0.72	0.11	5.97E-03	-0.84	0.04	5.91E-06	-0.79	0.11	2.54E-03	-1.06	0.13	8.93E-04	-0.23	0.14	3.91E-01	-0.33	0.20	3.36E-01	-0.43	0.10	5.72E-02	-0.86	0.03	4.77E-06
ITGA2	No	Yes	No	Down	0.63	2.19E-05	0.00081	-0.84	0.11	5.28E-03	-0.46	0.04	6.09E-03	-0.12	0.10	5.83E-01	-0.93	0.12	2.97E-03	-0.38	0.14	2.08E-01	-1.40	0.25	1.58E-02	-0.53	0.10	2.56E-02	-0.60	0.03	6.53E-04
MPPED2	No	Yes	No	Down	0.63	1.83E-19	1.5E-19	-0.44	0.10	6.22E-02	-0.88	0.04	5.45E-06	-0.61	0.11	1.71E-02	-0.48	0.12	6.25E-02	-0.92	0.15	6.28E-02	-0.01	0.20	9.85E-01	-1.32	0.11	8.63E-06	-0.60	0.03	4.39E-04
PEBP1	No	No	No	Down	0.63	0.002123	validated	-0.39	0.10	2.55E-01	-0.14	0.04	5.12E-01	-0.66	0.11	8.40E-02	-1.86	0.16	5.83E-04	-0.04	0.14	7.26E-01	-1.43	0.25	5.21E-04	-0.10	0.10	6.81E-01	-0.63	0.03	2.12E-02
ARSD	No	Yes	No	Down	0.63	1.14E-05	0.00182	-0.40	0.10	1.88E-01	-1.03	0.04	1.59E-05	-0.47	0.10	8.50E-02	-0.37	0.11	1.89E-01	-1.03	0.15	1.12E-03	-0.83	0.22	1.32E-02	-1.00	0.10	2.67E-03	-0.12	0.03	4.79E-01
STX6	No	Yes	No	Down	0.64	0.000321	2E-05	-0.81	0.11	2.30E-02	-0.18	0.04	2.36E-01	-0.43	0.10	1.21E-01	-0.59	0.12	5.22E-02	-0.88	0.15	6.66E-03	-1.16	0.23	6.20E-03	-0.91	0.10	3.93E-03	-0.27	0.03	9.18E-02

Supplementary Table 2. Details for 2,053 genes differentially expressed between EEC and NEEC, identified by microarray meta-analysis.

EEC - endometrioid endometrial cancer, NEEC - non-endometrioid endometrial cancer, SMD - Standardised Mean Difference, FDR - False Discovery Rate, Var - Variance

Gene	145 gene list	1253 gene list	Associated with EEC-specific survival?	Up/Down Regulated in NEEC	average standardised fold change	Microarray Meta FDR	RNASeq FDR	Individual microarray study results																							
								TCGA			E-MTAB-2532			E-GEOD-56026			GSE32507			GSE24537			E-GEOD-23518			E-GEOD-17025			E-GEOD-2109		
								SMD	Var	P-value	SMD	Var	P-value	SMD	Var	P-value	SMD	Var	P-value	SMD	Var	P-value	SMD	Var	P-value	SMD	Var	P-value	SMD	Var	P-value
ZMYND12	No	Yes	No	Down	0.64	1.14E-05	0.00112	-0.47	0.10	7.38E-02	-0.64	0.04	1.04E-03	-0.40	0.10	1.07E-01	-0.73	0.12	1.87E-02	-0.96	0.15	1.29E-02	-0.32	0.20	3.67E-01	-0.95	0.10	2.29E-03	-0.76	0.03	9.18E-04
EFHC1	No	No	No	Down	0.64	7.73E-05	validated	-0.22	0.10	4.34E-01	-0.46	0.04	8.55E-03	-0.05	0.10	8.44E-01	-1.13	0.13	7.98E-03	-0.84	0.15	2.69E-02	-0.75	0.21	1.71E-01	-1.05	0.10	3.66E-04	-0.72	0.03	7.34E-04
S100A11	No	No	No	Down	0.64	1.14E-05	0.03543	-0.16	0.10	5.89E-01	-0.57	0.04	2.52E-03	-0.90	0.11	1.19E-02	-0.51	0.12	1.48E-01	-0.43	0.14	7.31E-02	-1.20	0.24	1.23E-03	-0.77	0.10	1.12E-02	-0.70	0.03	6.15E-04
PLEKHA1	No	No	No	Down	0.64	0.00017	validated	-0.35	0.10	2.42E-01	-0.62	0.04	2.27E-03	-0.33	0.10	2.34E-01	-1.40	0.14	1.37E-03	-0.15	0.14	3.92E-01	-1.06	0.23	5.24E-03	-0.77	0.10	1.80E-02	-0.55	0.03	2.53E-02
PITRM1	No	No	No	Down	0.64	0.000405	validated	-0.86	0.11	1.03E-02	-0.51	0.04	9.56E-03	-0.75	0.11	2.22E-02	-0.90	0.12	4.59E-02	-0.51	0.14	7.86E-02	-0.61	0.21	7.07E-02	-0.95	0.10	3.45E-03	-0.13	0.03	4.46E-01
TTC29	No	Yes	No	Down	0.64	3.58E-06	0.00037	-0.79	0.11	1.31E-03	-0.62	0.04	4.28E-04	-0.60	0.11	1.75E-02	-0.33	0.11	2.05E-01	-0.94	0.15	1.76E-02	-0.69	0.21	1.77E-01	-0.70	0.10	1.11E-02	-0.53	0.03	3.51E-04
KIAA0232	No	No	No	Down	0.64	0.014864	validated	-0.31	0.10	4.17E-01	-0.58	0.04	2.50E-02	-0.71	0.11	3.03E-02	-0.99	0.13	2.34E-02	-0.92	0.15	1.41E-01	-0.82	0.22	2.19E-01	-0.50	0.10	9.31E-02	-0.38	0.03	1.85E-01
CRIP1	No	No	No	Down	0.64	1.83E-19	validated	-0.47	0.10	6.43E-02	-0.70	0.04	8.11E-05	-0.03	0.10	8.70E-01	-1.38	0.14	1.04E-04	-0.40	0.14	7.94E-02	-0.34	0.20	1.81E-01	-0.96	0.10	8.72E-04	-0.92	0.03	9.09E-07
FBXL8	No	Yes	No	Down	0.64	0.017726	0.00014	-1.01	0.11	1.50E-03	-0.27	0.04	2.31E-01	-0.36	0.10	3.37E-01	-1.03	0.13	2.04E-02	-1.00	0.15	3.06E-01	-0.65	0.21	3.85E-01	-0.60	0.10	2.77E-01	-0.28	0.03	7.96E-02
ARHGEF16	No	No	No	Down	0.64	0.000148	validated	-1.16	0.11	2.53E-03	-0.55	0.04	2.23E-03	-0.39	0.10	3.05E-01	-1.55	0.14	3.14E-05	-0.73	0.14	2.34E-01	-0.30	0.20	6.03E-01	0.00	0.10	9.96E-01	-0.51	0.03	1.54E-02
C1ORF172	No	No	No	Down	0.64	3.58E-06	validated	-0.24	0.10	5.79E-01	-0.66	0.04	3.10E-04	-0.28	0.10	3.84E-01	-1.21	0.13	1.23E-04	-0.91	0.15	3.19E-02	-0.92	0.22	4.33E-02	-0.03	0.10	9.26E-01	-0.93	0.03	2.04E-06
TMEM63A	No	Yes	No	Down	0.64	6.71E-06	0.00083	-0.47	0.10	1.19E-01	-1.12	0.04	9.09E-07	-0.32	0.10	2.16E-01	-1.49	0.14	2.64E-04	-0.26	0.14	2.60E-01	-0.18	0.20	5.44E-01	-0.80	0.10	6.84E-03	-0.54	0.03	1.98E-02
UGDH	No	No	No	Down	0.64	2.40E-05	validated	-0.66	0.11	2.40E-02	-0.66	0.04	7.25E-04	-0.85	0.11	1.19E-02	-0.41	0.12	1.72E-01	-0.80	0.15	8.83E-03	-0.62	0.21	6.53E-02	-0.34	0.10	2.20E-01	-0.83	0.03	7.17E-04
GFM2	No	Yes	No	Down	0.64	0.001814	0.03136	-0.55	0.10	1.87E-01	-0.63	0.04	3.33E-03	-0.75	0.11	1.52E-02	-0.52	0.12	2.40E-01	-1.05	0.15	2.34E-02	-0.36	0.20	4.57E-01	-0.92	0.10	9.77E-03	-0.40	0.03	1.01E-01
APRT	No	No	No	Down	0.64	0.000781	validated	-0.63	0.11	7.99E-02	-0.63	0.04	8.76E-03	-0.38	0.10	3.12E-01	-1.46	0.14	4.56E-03	-0.46	0.14	8.16E-02	-0.15	0.20	4.52E-01	-0.86	0.10	9.67E-03	-0.59	0.03	1.03E-02
NLRX1	No	Yes	No	Down	0.64	0.000752	0.01003	-0.73	0.11	4.23E-02	-1.14	0.04	2.16E-05	-0.47	0.10	1.75E-01	-1.10	0.13	2.37E-02	-0.81	0.15	3.64E-02	-0.49	0.21	1.69E-01	-0.20	0.10	5.68E-01	-0.24	0.03	2.53E-01
CST1	No	Yes	No	Down	0.64	1.83E-19	2E-05	-0.75	0.11	3.50E-03	-0.42	0.04	5.05E-03	-0.75	0.11	2.87E-03	-0.95	0.12	7.01E-04	-1.05	0.15	2.58E-03	-0.46	0.21	8.90E-02	-0.42	0.10	6.06E-02	-0.36	0.03	4.75E-03
SLC22A4	No	Yes	No	Down	0.64	8.12E-05	0.02185	-0.78	0.11	5.20E-03	-0.86	0.04	5.88E-05	-0.14	0.10	5.48E-01	-0.47	0.12	1.24E-01	-0.91	0.15	7.69E-02	-0.82	0.22	2.03E-01	-0.61	0.10	2.59E-02	-0.55	0.03	4.15E-03
WDR77	No	No	No	Down	0.64	0.001351	validated	-0.59	0.10	7.77E-02	-0.59	0.04	4.09E-03	-0.24	0.10	4.13E-01	-1.24	0.13	8.53E-03	-1.01	0.15	7.84E-03	-0.79	0.22	5.59E-02	-0.45	0.10	1.02E-01	-0.24	0.03	2.67E-01
ANKRD35	No	Yes	No	Down	0.64	1.83E-19	1.5E-19	-0.96	0.11	6.69E-04	-0.81	0.04	6.59E-06	-0.74	0.11	1.24E-02	-0.67	0.12	1.33E-02	-0.03	0.14	8.20E-01	-0.21	0.20	3.88E-01	-0.94	0.10	5.11E-04	-0.77	0.03	9.09E-05
TP53I3	No	Yes	No	Down	0.64	1.41E-05	0.00862	-0.66	0.11	3.02E-02	-0.59	0.04	2.33E-03	-0.48	0.10	8.18E-02	-0.47	0.12	1.90E-01	-0.95	0.15	3.34E-03	-0.08	0.20	6.34E-01	-1.28	0.10	9.31E-05	-0.61	0.03	9.54E-03
RNF126	No	No	No	Down	0.64	0.013848	validated	-0.95	0.11	3.69E-02	-0.03	0.04	8.56E-01	-0.56	0.11	9.00E-02	-1.44	0.14	3.73E-03	-0.72	0.14	8.04E-02	-0.40	0.20	3.04E-01	-0.54	0.10	1.65E-01	-0.49	0.03	7.74E-02
KIAA0141	No	No	No	Down	0.64	0.006294	validated	-1.15	0.11	7.14E-03	-0.51	0.04	2.97E-02	-0.82	0.11	4.27E-02	-1.24	0.13	1.86E-02	-0.18	0.14	4.74E-01	-0.65	0.21	1.34E-01	-0.41	0.10	1.70E-01	-0.17	0.03	2.57E-01
PRRG2	No	No	No	Down	0.64	0.000621	validated	-0.27	0.10	3.82E-01	-0.55	0.04	1.27E-02	-0.39	0.10	3.05E-01	-0.16	0.11	5.48E-01	-1.57	0.17	1.92E-04	-1.29	0.24	6.83E-03	-0.30	0.10	5.21E-01	-0.60	0.03	3.80E-03
PCSK9	No	Yes	No	Down	0.64	1.41E-05	6.4E-05	-1.38	0.12	1.59E-05	-0.67	0.04	4.68E-05	-0.72	0.11	1.37E-02	-0.51	0.12	4.12E-02	-0.48	0.14	4.03E-01	-0.85	0.22	3.17E-01	-0.21	0.10	4.46E-01	-0.31	0.03	8.49E-02
RASEF	No	No	No	Down	0.64	3.97E-05	validated	-0.66	0.11	1.25E-02	-0.47	0.04	4.34E-03	-0.30	0.10	2.09E-01	-1.42	0.14	4.27E-05	-1.11	0.15	8.34E-03	-0.61	0.21	1.04E-01	-0.22	0.10	3.15E-01	-0.33	0.03	7.25E-02
NMRAL1	No	Yes	No	Down	0.64	0.000754	0.00525	-0.76	0.11	6.60E-02	-0.55	0.04	2.67E-02	-0.47	0.10	1.28E-01	-0.28	0.11	5.09E-01	-0.05	0.14	6.67E-01	-0.83	0.22	1.99E-02	-1.40	0.11	2.00E-04	-0.77	0.03	1.10E-02
C11ORF63	No	Yes	No	Down	0.64	0.000602	1.5E-19	-0.73	0.11	1.63E-02	-0.56	0.04	2.83E-03	-0.40	0.10	1.25E-01	-0.87	0.12	4.06E-03	-0.79	0.15	1.02E-01	-0.67	0.21	2.72E-01	-0.96	0.10	6.84E-03	-0.12	0.03	4.29E-01
SLC9A3R1	No	No	No	Down	0.64	1.83E-19	validated	-0.35	0.10	2.12E-01	-0.84	0.04	3.09E-05	-0.76	0.11	1.36E-02	-0.45	0.12	1.34E-01	-0.81	0.15	4.54E-03	-0.42	0.20	1.22E-01	-0.61	0.10	2.65E-02	-0.86	0.03	2.18E-05
KIAA1737	No	No	No	Down	0.64	0.000125	validated	-0.36	0.10	2.86E-01	-0.51	0.04	2.15E-02	-0.05	0.10	8.71E-01	-0.08	0.11	8.10E-01	-1.32	0.16	6.37E-04	-0.95	0.22	2.33E-02	-0.52	0.10	4.56E-02	-1.31	0.04	3.00E-05
SPAG4	No	Yes	Yes	Down	0.64	0.000405	2E-05	-0.37	0.10	1.65E-01	-0.35	0.04	2.75E-02	-0.20	0.10	3.98E-01	-1.35	0.14	5.64E-04	-0.87	0.15	4.11E-02	-0.90	0.22	6.93E-02	-0.60	0.10	3.15E-02	-0.46	0.03	9.49E-03
NXNL2	No	Yes	No	Down	0.64	0.000385	0.01307	-1.01	0.11	8.35E-04	-0.85	0.04	3.05E-04	-0.61	0.11	1.17E-01	-0.55	0.12	6.90E-02	-1.01	0.15	1.96E-02	-0.60	0.21	2.35E-01	-0.17	0.10	7.61E-01	-0.30	0.03	1.08E-01
EDF1	No	No	No	Down	0.64	0.001349	0.00843	-0.08	0.10	8.32E-01	-0.34	0.04	1.74E-01	-0.63	0.11	1.20E-01	-0.92	0.12	1.40E-01	-0.57	0.14	2.64E-02	-0.36	0.20	1.63E-01	-1.56	0.11	2.25E-05	-0.63	0.03	5.62E-02
C1ORF201	No	Yes	No	Down	0.64	0.001648	0.00058	-1.21	0.11	3.33E-03	-0.73	0.04	4.09E-04	-0.48	0.10	1.86E-01	-1.41	0.14	1.86E-03	-0.19	0.14	7.39E-01	-0.51	0.21	5.13E-01	-0.49	0.10	1.72E-01	-0.06	0.03	6.58E-01
THUMPD1	No	No	No	Down	0.64	0.003959	validated	-1.14	0.11	1.48E-02	-0.24	0.04	1.72E-01	-0.87	0.11	3.35E-02	-0.79	0.12	5.73E-02	-0.42	0.14	1.65E-01	-0.13	0.20	6.38E-01	-0.48	0.10	1.43E-01	-1.01	0.04	2.13E-03
BRP44	No	No	No	Down	0.64	2.64E-05	validated	-0.20	0.10	4.84E-01	-0.45	0.04	7.27E-03	-1.42	0.12	6.84E-05	-0.99	0.13	8.99E-03	-0.33	0.14	1.47E-01	-0.77	0.21	1.88E-02	-0.08	0.10	7.37E-01	-0.83	0.03	1.03E-03
AK3	No	No	No	Down	0.65	0.000302	validated	-0.42	0.10	2.10E-01	-0.85	0.04	1.28E-03	-0.02	0.10	9.25E-01	-0.90	0.12	4.39E-02	-0.17	0.14	3.49E-01	-0.77	0.21	2.07E-02	-1.35	0.11	1.80E-04	-0.56	0.03	3.31E-02
C9ORF142	No	Yes	No	Down	0.65	0.000707	0.01695	-0.22	0.10	4.91E-01	-0.23	0.04	1.93E-01	-0.49	0.10	2.17E-01	-0.99	0.13	3.31E-02	-0.53	0.14	4.42E-02	-0.88	0.22	1.39E-02	-0.82	0.10	3.29E-02	-0.88	0.03	2.01E-04
RNF43	No	No	No	Down	0.65	2.85E-05	validated	-0.48	0.10	8.02E-02	-0.46	0.04	3.55E-03	-0.20	0.10	3.96E-01	-1.23	0.13	2.43E-04	-0.73	0.14	1.12E-01	-0.54	0.21	1.75E-01	-0.98	0.10	6.22E-04	-0.42	0.03	1.84E-02
KRTCAP3	No	No	No	Down	0.65	9.89E-06	validated	-0.24	0.10	4.05E-01	-0.60	0.04	6.28E-04	-0.43	0.10	1.24E-01	-1.11	0.13	5.31E-04	-0.76	0.15	6.48E-02	-0.54	0.21	1.58E-01	-0.45	0.10	1.13E-01	-0.91	0.03	1.34E-05
ARMC2	No	No	No	Down	0.65	0.000594	validated	-0.81	0.11	8.30E-03	-0.53	0.04	7.01E-03	-0.47	0.10	9.43E-02	-1.06	0.13	3.64E-03	-0.91	0.15	1.62E-01	-0.27	0.20	7.73E-01	-0.50	0.10	9.06E-02	-0.49	0.03	7.98E-03
PIP5K1B	No	Yes	No	Down	0.65	4.80E-05	1.5E-19	-0.34	0.10	1.93E-01	-1.09	0.04	2.27E-06	-0.30	0.10	1.92E-01	-0.22	0.11	4.18E-01	-0.84	0.15	2.17E-01	-0.72	0.21	3.55E-01	-0.85	0.10	2.79E-03	-0.70	0.03	1.73E-03
TEKT2	No	Yes	No	Down	0.65	1.14E-05	0.01276	-0.32	0.10	1.78E-01	-0.63	0.04	1.19E-04	-0.29	0.10	2.44E-01	-0.86	0.12	4.82E-03	-1.11	0.15	1.60E-02	-0.62	0.21	1.87E-01	-0.64	0.10	2.43E-02	-0.56	0.03	9.98E-04
WDR66	No	Yes	No	Down	0.65	6.71E-06	0.00809	-0.65	0.11	1.26E-02	-0.39	0.04	1.02E-02	-0.54	0.11	2.99E-02	-0.97	0.13	1.92E-03	-0.50	0.14	1.18E-01	-0.40	0.20	4.32E-01	-1.17	0.10	6.91E-05	-0.39	0.03	6.05E-03
SCMH1	No	Yes	No	Down	0.65	0.011045	0.01445	-0.50	0.10	1.84E-01	-0.33	0.04	1.70E-01	-0.48	0.10	1.78E-01	-0.92	0.12	8.34E-02	-0.37	0.14	1.69E-01	-1.21	0.24	7.13E-03	-0.55	0.10	1.41E-01	-0.66	0.03	2.41E-02
MAP7	No	No	No	Down	0.65	1.66E-05	validated	-0.12	0.10	6.69E-01	-0.72	0.04	1.75E-04	-0.35	0.10	2.30E-01	-0.74	0.12	2.54E-02	-1.16	0.16	2.21E-03	-1.06	0.23	7.47E-03	-0.15	0.10	5.86E-01	-0.71	0.03	3.44E-04
GSTA3	No	Yes	No	Down	0.65	0.00011	0.00043	-0.24	0.10	3.00E-01	-0.57	0.04	7.34E-04	-0.61	0.11	2.51E-02	-0.58	0.12	3.43E-02	-0.88	0.15	8.94E-02	-0.68	0.21	2.86E-01	-0.96	0.10	7.50E-03	-0.49	0.03	2.13E-03
AQP3	No	Yes	No	Down	0.65	1.83E-19	0.01136	-0.86	0.11	1.74E-03	-0.93	0.04	1.59E-06	-0.63	0.11	1.03E-02	-0.21	0.11	4.33E-01	-0.48	0.14	5.88E-01	-0.01	0.20	9.88E-01	-1.23	0.10	1.75E-05	-0.67	0.03	2.62E-04
IRF6	No	No	No	Down	0.65	1.14E-05	validated	-0.04	0.10	8.92E-01	-0.98	0.04	1.00E-05	-0.18	0.10	4.82E-01	-1.00	0.13	1.70E-03	-1.17	0.16	2.75E-03	-0.31	0.20	3.07E-01	-0.93	0.10	2.38E-03	-0.41	0.03	2.44E-02
SGK3	No	Yes	No	Down	0.65	1.14E-05	0.00383	-0.10	0.10	7.69E-01	-0.92	0.04	8.79E-05	-0.73	0.11	3.20E-02	-0.73	0.12	5.64E-02	-0.45	0.14	1.84E-01	-0.31	0.20	3.96E-01	-0.53	0.10	7.30E-02	-1.24	0.04	2.04E-06
DUT	No	No	No	Down	0.65	0.012496	validated	-0.77	0.11	3.43E-02	-0.56	0.04	1.56E-02	-0.73	0.11	3.88E-02	-0.90	0.12	1.17E-01	-1.48	0.17	8.03E-03	-0.44	0.20	2.11E-01	0.00	0.10	9.97E-01	-0.13	0.03	6.23E-01
CXCL14	No	Yes	Yes	Down	0.65	1.83E-19	1.5E-19	-1.32	0.12	2.27E-06	-0.54	0.04	2.61E-04	-0.69	0.11	3.23E-03	-0.16	0.11	4.66E-01	-0.46	0.14	5.98E-02	-0.65	0.21	4.27E-02	-0.78	0.10	7.26E-04	-0.41	0.03	2.57E-03
CCL20	No	Yes	No	Down	0.65	1.83E-19	0.00552	-0.56	0.10	1.55E-02	-0.65	0.04	6.25E-05	-0.64	0.11	5.68E-03	-0.70	0.12	7.59E-03	-0.73	0.14	1.20E-02	-0.87	0.22	1.49E-02	-0.47	0.10	3.41E-02	-0.39	0.03	3.50E-03
LRGUK	No	Yes	No	Down	0.65	0.004219	0.00068	-0.32	0.10	2.36E-01	-0.42	0.04	4.28E-02	-0.55	0.11	7.36E-02	-1.19	0.13	7.64E-04	-1.09	0.15	2.79E-01	-0.47	0.21	6.53E-01	-0.71	0.10	8.26E-03	-0.25	0.03	2.16E-01
EN2	No	Yes	No	Down	0.65	0.002864	1.5E-19	-0.78	0.11	1.04E-02	-0.05	0.04	7.30E-01	-0.82	0.11	2.59E-03	-0.99	0.13	1.23E-02	-0.72	0.14	1.74E-01	-0.93	0.22	9.85E-02	-0.53	0.10	6.55E-02	-0.16	0.03	2.67E-01
ACAD8	No	Yes	No	Down	0.65	0.001333	0.01682	-0.33	0.10	3.01E-01	-0.69	0.04	4.43E-03	-0.07	0.10	8.01E-01	-0.15	0.11	7.41E-01	-0.94	0.15	3.25E-02	-0.85	0.22	6.33E-02	-1.21	0.10	6.92E-04	-0.74	0.03	1.14E-02
COG3	No	No	No	Down	0.65	0.004082	validated	-0.82	0.11	6.43E-02	-0.67	0.04	4.38E-03	-0.54	0.11	1.66E-01	-0.51	0.12	1.98E-01	-0.83	0.15	8.99E-02	-0.03	0.20	9.42E-01	-1.32	0.11	8.95E-04	-0.27	0.03	2.45E-01
TSPO	No	Yes	No	Down	0.65	5.41E-05	0.00593	-0.92	0.11	8.42E-03	-0.81	0.04	1.75E-04	-0.68	0.11	4.33E-02	-0.50	0.12	2.07E-01	-0.32	0.14	1.36E-01	-0.76	0.21	1.78E-02	-0.15	0.10	5.60E-01	-0.83	0.03	1.10E-03
SPATS1	No	Yes	No	Down	0.65	0.000345	1.5E-19	-0.74	0.11	4.52E-03	-0.68	0.04	9.00E-04	-0.66	0.11	2.28E-02	-0.63	0.12	2.87E-02	-0.90	0.15	2.96E-02	-0.70	0.21	2.60E-01	-0.46	0.10	1.33E-01	-0.21	0.03	2.28E-01
AMD1	No	No	No	Down	0.65	0.002764	validated	-1.17	0.11	5.61E-03	-0.16	0.04	3.50E-01	-0.31	0.10	3.34E-01	-0.35	0.11	3.52E-01	-0.99	0.15	3.03E-03	-1.11	0.23	8.99E-03	-0.47	0.10	8.10E-02	-0.40	0.03	1.30E-01
DUS2L	No	Yes	Yes	Down	0.65	0.031909	0.00068	-0.85	0.11	3.33E-02	-0.39	0.04	8.02E-02	-0.56	0.11	2.58E-01	-0.06	0.11	8.84E-01	-1.32	0.16	1.96E-02	-0.66	0.21	1.66E-01	-0.92	0.10	4.70E-02	-0.20	0.03	3.11E-01
VPS37A	No	Yes	No	Down	0.65	0.003735	0.0011	-0.68	0.11	5.82E-02	-0.21	0.04	3.45E-01	-1.17	0.11	1.62E-03	-0.08	0.11	8.25E-01	-0.54	0.14	1.17E-01	-0.96	0.22	6.08E-02	-0.55	0.10	5.43E-02	-0.77	0.03	1.33E-02
HPGD	No	Yes	No	Down	0.65	1.83E-19	6.8E-05	-0.86	0.11	1.61E-03	-0.51	0.04	8.63E-04	-0.53	0.11	1.50E-02	-0.42	0.12	7.59E-02	-0.69	0.14	9.86E-03	-0.44	0.20	1.07E-01	-0.66	0.10	4.01E-03	-0.84	0.03	1.00E-20
KCNE1	No	Yes	No	Down	0.65	1.83E-19	0.00011	-0.60	0.10	2.33E-02	-0.81	0.04	6.36E-06	-0.53	0.11	2.95E-02	-0.12	0.11	6.21E-01	-0.90	0.15	5.05E-02	-0.39	0.20	4.09E-01	-0.85	0.10	1.48E-03	-0.93	0.03	2.04E-06
NDUFS7	No	Yes	No	Down	0.65	0.003833	0.00272	-0.50	0.10	1.23E-01	-0.21	0.04	3.19E-01	-1.08	0.11	5.23E-03	-1.14	0.13	2.62E-02	-0.25	0.14	2.34E-01	-0.93	0.22	7.38E-03	-0.61	0.10	9.82E-02	-0.22	0.03	1.61E-01
NFIC	No	Yes	No	Down	0.65	0.000229	6.7E-06	-1.14	0.11	2.91E-03	-0.63	0.04	2.58E-03	-1.19	0.11	7.23E-04	-1.02	0.13	2.19E-02	-0.09	0.14	5.38E-01	-0.34	0.20	2.20E-01	-0.13	0.10	6.07E-01	-0.41	0.03	3.55E-02
COX7C	No	No	No	Down	0.65	0.005522	validated	-0.57	0.10	1.27E-01	-0.44	0.04	6.27E-02	-0.81	0.11	5.58E-02	-0.90	0.12	1.13E-01	-0.45	0.14	5.52E-02	-0.99	0.22	3.83E-03	-0.28	0.10	3.25E-01	-0.51	0.03	9.93E-02
WDR45L	No	No	No	Down	0.65	0.002624	validated	-0.64	0.11	5.93E-02	-0.44	0.04	3.28E-02	-0.66	0.11	8.51E-02	-1.07	0.13	2.00E-02	-0.53	0.14	4.73E-02	-0.19	0.20	4.00E-01	-0.79	0.10	2.96E-02	-0.62	0.03	3.16E-02
RGS7BP	No	Yes	Yes	Down	0.65	0.004062	0.00269	-0.43	0.10	1.59E-01	-0.61	0.04	3.85E-02	-0.92	0.11	1.23E-03	-0.42	0.12	1.56E-01	-0.78	0.15	1.47E-01	-0.98	0.22	2.63E-01	-0.51	0.10	5.17E-02	-0.28	0.03	7.20E-02

Supplementary Table 2. Details for 2,053 genes differentially expressed between EEC and NEEC, identified by microarray meta-analysis.

EEC - endometrioid endometrial cancer, NEEC - non-endometrioid endometrial cancer, SMD - Standardised Mean Difference, FDR - False Discovery Rate, Var - Variance

Gene	145 gene list	1253 gene list	Associated with EEC-specific survival?	Up/Down Regulated in NEEC	average standardised fold change	Microarray Meta FDR	RNASeq FDR	Individual microarray study results: TCGA			E-MTAB-2532			E-GEOD-56026			GSE32507			GSE24537			E-GEOD-23518			E-GEOD-17025			E-GEOD-2109		
								SMD	Var	P-value	SMD	Var	P-value	SMD	Var	P-value	SMD	Var	P-value	SMD	Var	P-value	SMD	Var	P-value	SMD	Var	P-value	SMD	Var	P-value
YTHDC2	No	Yes	No	Down	0.65	0.001604	0.01491	-0.90	0.11	4.73E-03	-0.32	0.04	1.04E-01	-0.69	0.11	2.75E-02	-0.84	0.12	3.59E-02	-0.59	0.14	3.47E-01	-0.34	0.20	5.74E-01	-0.96	0.10	1.31E-03	-0.29	0.03	1.50E-01
TMEM30B	No	No	No	Down	0.65	8.89E-05	validated	-1.04	0.11	2.32E-02	-0.19	0.04	1.80E-01	-0.35	0.10	1.55E-01	-0.55	0.12	3.42E-02	-1.23	0.16	4.38E-04	-0.33	0.20	2.10E-01	-0.56	0.10	2.19E-02	-0.67	0.03	9.83E-04
TCTN1	No	No	No	Down	0.65	0.000248	validated	-0.54	0.10	4.79E-02	-0.64	0.04	1.55E-03	-0.21	0.10	4.42E-01	-0.46	0.12	1.55E-01	-1.21	0.16	6.46E-04	-0.85	0.22	2.47E-02	-0.66	0.10	6.55E-02	-0.35	0.03	6.58E-02
WDR63	No	Yes	No	Down	0.65	6.71E-06	0.00705	-0.57	0.10	2.00E-02	-0.71	0.04	5.00E-05	-0.61	0.11	1.43E-02	-0.91	0.12	2.10E-03	-0.71	0.14	1.21E-01	-0.28	0.20	6.82E-01	-0.70	0.10	9.30E-03	-0.41	0.03	5.42E-03
UFM1	No	No	No	Down	0.65	0.000674	validated	-0.68	0.11	6.36E-02	-0.60	0.04	1.25E-02	-0.03	0.10	9.04E-01	-0.65	0.12	1.40E-01	-1.08	0.15	3.04E-03	-0.25	0.20	3.40E-01	-1.16	0.10	4.44E-04	-0.46	0.03	5.43E-02
GMPR	No	Yes	No	Down	0.65	9.98E-05	0.00075	-0.76	0.11	5.59E-03	-0.74	0.04	1.54E-04	-0.50	0.10	5.70E-02	-0.38	0.11	1.93E-01	-0.73	0.14	3.45E-02	-1.05	0.23	1.95E-02	-0.55	0.10	3.48E-02	-0.20	0.03	2.61E-01
PAPSS1	No	Yes	No	Down	0.65	0.000125	0.00031	-0.90	0.11	5.74E-03	-0.52	0.04	1.01E-02	-0.69	0.11	2.81E-02	-0.62	0.12	1.04E-01	-0.09	0.14	5.33E-01	-0.40	0.20	1.39E-01	-0.97	0.10	1.65E-03	-0.71	0.03	4.22E-03
LIMS1	No	No	No	Down	0.65	0.000258	validated	-0.81	0.11	1.21E-02	-0.35	0.04	7.12E-02	-0.31	0.10	2.37E-01	-0.53	0.12	2.70E-01	-1.06	0.15	1.12E-03	-0.89	0.22	1.48E-02	-0.11	0.10	5.79E-01	-0.84	0.03	6.89E-04
ELMO3	No	No	No	Down	0.65	0.000676	validated	-0.32	0.10	2.33E-01	-0.21	0.04	1.82E-01	-0.22	0.10	4.28E-01	-1.98	0.16	1.52E-05	-0.76	0.15	8.23E-02	-0.44	0.20	2.46E-01	-0.32	0.10	2.99E-01	-0.66	0.03	1.50E-03
C1ORF186	No	No	No	Down	0.65	1.83E-19	validated	-0.10	0.10	6.29E-01	-0.62	0.04	4.38E-05	-0.61	0.11	1.50E-02	-1.22	0.13	1.98E-05	-0.63	0.14	2.03E-02	-0.55	0.21	8.13E-02	-0.57	0.10	1.53E-02	-0.60	0.03	2.17E-04
PAFAH2	No	Yes	No	Down	0.65	0.004725	0.00354	-0.38	0.10	2.76E-01	-0.65	0.04	3.55E-03	-0.88	0.11	1.88E-02	-0.08	0.11	8.26E-01	-0.62	0.14	1.39E-01	-1.04	0.23	4.71E-02	-0.74	0.10	8.40E-02	-0.51	0.03	2.88E-02
OTUB2	No	Yes	No	Down	0.65	0.003189	0.00067	-0.63	0.11	5.00E-02	-0.72	0.04	2.21E-03	-0.17	0.10	5.44E-01	-1.13	0.13	1.76E-03	-0.86	0.15	5.81E-02	-0.76	0.21	2.36E-01	-0.57	0.10	8.51E-02	-0.04	0.03	7.77E-01
ANXA1	No	Yes	No	Down	0.65	1.83E-19	4.6E-06	-0.75	0.11	8.72E-03	-0.89	0.04	5.45E-06	-0.51	0.11	2.79E-02	-0.33	0.11	2.48E-01	-0.46	0.14	4.94E-02	-0.84	0.22	1.03E-02	-0.02	0.10	9.00E-01	-1.08	0.04	1.00E-20
LRRC6	No	No	No	Down	0.65	0.000707	validated	-0.41	0.10	9.79E-02	-0.35	0.04	3.03E-02	-0.40	0.10	1.27E-01	-0.59	0.12	4.04E-02	-1.08	0.15	1.95E-02	-0.74	0.21	1.83E-01	-0.88	0.10	1.96E-03	-0.43	0.03	2.61E-02
MS4A8B	No	No	No	Down	0.66	1.83E-19	0.01333	-0.67	0.11	4.58E-03	-0.70	0.04	1.61E-05	-0.51	0.10	2.85E-02	-0.76	0.12	2.66E-03	-0.60	0.14	2.51E-02	-0.56	0.21	1.53E-01	-0.39	0.10	7.82E-02	-0.68	0.03	3.18E-06
FHIT	No	Yes	No	Down	0.66	0.019962	0.02267	-0.87	0.11	1.70E-02	-0.31	0.04	9.01E-02	-0.54	0.11	1.12E-01	-0.25	0.11	4.48E-01	-1.10	0.15	9.85E-02	-0.74	0.21	1.99E-01	-0.92	0.10	1.14E-02	-0.15	0.03	5.03E-01
PKP3	No	No	No	Down	0.66	3.58E-06	validated	-0.46	0.10	9.82E-02	-0.82	0.04	5.91E-06	-0.24	0.10	3.94E-01	-1.25	0.13	8.45E-05	-0.80	0.15	8.73E-02	-0.39	0.20	3.09E-01	-0.28	0.10	4.17E-01	-0.64	0.03	3.61E-04
SUCLG2	No	Yes	No	Down	0.66	0.002976	0.02471	-0.42	0.10	1.92E-01	-0.77	0.04	4.84E-04	-0.58	0.11	1.08E-01	-0.72	0.12	1.67E-01	-0.90	0.15	1.85E-02	-1.29	0.24	8.30E-03	-0.11	0.10	6.07E-01	-0.09	0.03	5.08E-01
ANKRA2	No	Yes	No	Down	0.66	0.0025	0.00563	-0.95	0.11	1.20E-02	-0.61	0.04	3.52E-03	-0.76	0.11	4.06E-02	-0.72	0.12	9.67E-02	-0.62	0.14	8.91E-02	-0.44	0.20	2.41E-01	-0.02	0.10	9.42E-01	-0.75	0.03	1.55E-02
FHL2	No	Yes	No	Down	0.66	1.14E-05	0.00564	-0.27	0.10	2.84E-01	-0.74	0.04	1.44E-04	-0.39	0.10	1.40E-01	-0.19	0.11	4.65E-01	-1.13	0.16	3.48E-04	-1.14	0.23	1.58E-03	-0.56	0.10	2.65E-02	-0.45	0.03	1.85E-02
MMP26	No	Yes	No	Down	0.66	1.83E-19	1.5E-19	-0.98	0.11	1.31E-04	-0.70	0.04	1.45E-05	-0.58	0.11	1.59E-02	-1.15	0.13	3.95E-05	-0.48	0.14	7.33E-02	-0.49	0.21	1.28E-01	-0.39	0.10	8.66E-02	-0.08	0.03	5.55E-01
STXBP2	No	Yes	No	Down	0.66	0.002079	0.0017	-0.02	0.10	9.54E-01	-0.79	0.04	1.48E-03	-0.56	0.11	1.71E-01	-0.96	0.13	1.11E-02	-0.97	0.15	6.29E-03	-0.94	0.22	1.85E-02	-0.49	0.10	2.14E-01	-0.12	0.03	4.94E-01
TGM3	No	Yes	No	Down	0.66	0.001123	0.00132	-0.23	0.10	3.82E-01	-0.68	0.04	1.93E-03	-0.76	0.11	2.09E-02	-0.96	0.13	5.80E-03	-0.37	0.14	1.22E-01	-1.16	0.23	6.72E-02	-0.41	0.10	1.88E-01	-0.28	0.03	6.59E-02
DHX32	No	No	No	Down	0.66	0.002764	validated	-0.39	0.10	2.15E-01	-0.77	0.04	1.90E-03	-0.47	0.10	2.33E-01	-0.60	0.12	1.38E-01	-0.74	0.14	1.93E-02	-0.64	0.21	7.44E-02	-0.45	0.10	2.54E-01	-0.78	0.03	1.39E-02
UNC13B	No	Yes	No	Down	0.66	0.006512	0.00159	-0.71	0.11	5.46E-02	-0.57	0.04	1.24E-02	-0.07	0.10	8.26E-01	-0.63	0.12	1.49E-01	-0.29	0.14	4.74E-01	-0.52	0.21	3.21E-01	-1.35	0.11	1.21E-03	-0.70	0.03	3.31E-02
CLDN7	No	No	No	Down	0.66	3.58E-06	validated	-0.50	0.10	1.16E-01	-0.63	0.04	2.82E-04	-0.30	0.10	2.38E-01	-1.08	0.13	7.01E-04	-0.91	0.15	2.36E-03	-0.39	0.20	1.44E-01	-0.29	0.10	2.71E-01	-0.73	0.03	3.16E-05
LCN2	No	No	No	Down	0.66	1.83E-19	validated	0.00	0.10	9.82E-01	-0.61	0.04	8.11E-05	-0.32	0.10	1.14E-01	-1.47	0.14	7.27E-06	-0.51	0.14	3.33E-02	-0.95	0.22	4.50E-03	-0.25	0.10	2.20E-01	-0.71	0.03	2.04E-06
ALDH6A1	No	Yes	No	Down	0.66	9.84E-05	0.00459	-0.89	0.11	4.52E-03	-0.69	0.04	9.54E-04	-0.57	0.11	4.56E-02	-0.59	0.12	9.10E-02	-0.96	0.15	2.54E-03	-0.57	0.21	8.92E-02	-0.23	0.10	3.07E-01	-0.32	0.03	4.30E-02
PPP2R2C	No	Yes	No	Down	0.66	1.83E-19	0.00548	-0.88	0.11	9.32E-04	-0.95	0.04	1.36E-06	-0.59	0.11	1.18E-02	-0.55	0.12	2.45E-02	-0.52	0.14	4.08E-02	-0.88	0.22	1.57E-02	-0.15	0.10	4.46E-01	-0.30	0.03	5.09E-02
PPP1R7	No	No	No	Down	0.66	0.0022	0.00744	-0.28	0.10	4.68E-01	-0.74	0.04	4.02E-03	-0.22	0.10	4.89E-01	-0.01	0.11	9.84E-01	-1.43	0.17	1.57E-03	-1.06	0.23	2.29E-02	-0.66	0.10	2.57E-02	-0.42	0.03	4.52E-02
TTC21A	No	Yes	No	Down	0.66	0.000125	0.0003	-0.70	0.11	1.05E-02	-0.51	0.04	2.60E-03	-0.62	0.11	4.44E-02	-0.84	0.12	1.56E-02	-0.38	0.14	1.38E-01	-0.49	0.21	4.10E-01	-1.17	0.10	1.74E-04	-0.10	0.03	5.21E-01
DNAI1	No	Yes	No	Down	0.66	3.58E-06	0.00261	-0.49	0.10	7.89E-02	-0.71	0.04	1.21E-04	-0.51	0.11	6.20E-02	-0.84	0.12	3.65E-03	-0.60	0.14	4.03E-02	-0.49	0.21	3.42E-01	-0.58	0.10	2.29E-02	-0.59	0.03	9.16E-05
TRIM13	No	Yes	No	Down	0.66	0.000925	0.04471	-0.84	0.11	5.98E-02	-0.62	0.04	4.14E-03	-0.04	0.10	8.53E-01	-0.57	0.12	7.50E-02	-0.78	0.15	2.25E-02	-0.57	0.21	1.33E-01	-1.16	0.10	5.24E-04	-0.22	0.03	2.27E-01
TC2N	No	No	No	Down	0.66	2.40E-05	validated	-0.46	0.10	1.04E-01	-1.32	0.04	4.54E-07	-0.29	0.10	2.63E-01	-1.02	0.13	1.99E-03	-0.33	0.14	3.59E-01	-0.66	0.21	2.17E-01	-0.31	0.10	1.92E-01	-0.43	0.03	1.18E-02
DNAJC15	No	No	No	Down	0.66	9.84E-05	validated	-0.33	0.10	1.69E-01	-0.02	0.04	8.61E-01	-1.37	0.12	3.00E-05	-0.81	0.12	1.22E-02	-0.50	0.14	6.58E-02	-0.79	0.22	3.07E-02	-0.62	0.10	1.79E-02	-0.36	0.03	2.74E-02
ZNF480	No	No	No	Down	0.66	0.010856	validated	-1.02	0.11	1.64E-03	-0.26	0.04	1.47E-01	-0.70	0.11	9.59E-02	-1.18	0.13	1.05E-02	-0.49	0.14	1.47E-01	-0.63	0.21	1.48E-01	-0.43	0.10	4.87E-01	-0.08	0.03	7.02E-01
CCDC41	No	Yes	No	Down	0.66	0.001536	0.02733	-0.30	0.10	3.09E-01	-0.53	0.04	8.13E-03	-0.06	0.10	8.34E-01	-0.95	0.13	2.41E-02	-0.67	0.14	7.27E-02	-0.87	0.22	9.52E-02	-1.22	0.10	2.30E-04	-0.19	0.03	2.16E-01
SOX5	No	Yes	No	Down	0.66	0.008176	0.03803	-0.83	0.11	5.41E-03	-0.01	0.04	9.32E-01	-0.23	0.10	3.26E-01	-0.87	0.12	2.72E-03	-1.11	0.15	4.51E-01	-0.78	0.22	6.33E-01	-0.72	0.10	6.28E-03	-0.24	0.03	1.08E-01
SPATA2L	No	Yes	No	Down	0.66	0.015649	0.00283	-0.57	0.10	9.07E-02	-0.16	0.04	4.06E-01	-0.19	0.10	6.10E-01	-0.98	0.13	1.94E-02	-0.92	0.15	3.83E-02	-0.62	0.21	2.05E-01	-0.75	0.10	3.34E-02	-0.59	0.03	3.95E-02
AFTPH	No	No	No	Down	0.66	0.025383	validated	-0.41	0.10	4.08E-01	-0.34	0.04	1.23E-01	-0.22	0.10	5.63E-01	-1.22	0.13	2.95E-02	-0.52	0.14	9.82E-02	-0.86	0.22	3.91E-02	-0.38	0.10	2.49E-01	-0.83	0.03	2.00E-02
SLC37A3	No	Yes	No	Down	0.66	0.001121	0.00414	-0.47	0.10	1.98E-01	-0.60	0.04	1.67E-03	-0.64	0.11	8.37E-02	-0.09	0.11	7.69E-01	-0.43	0.14	1.23E-01	-0.49	0.21	1.21E-01	-1.25	0.10	5.22E-03	-0.81	0.03	5.45E-03
TMEM181	No	Yes	No	Down	0.66	0.002715	0.01361	-1.31	0.12	7.69E-04	-0.08	0.04	6.77E-01	-0.57	0.11	9.18E-02	-0.54	0.12	2.19E-01	-0.34	0.14	1.34E-01	-0.69	0.21	3.41E-02	-0.49	0.10	9.95E-02	-0.75	0.03	1.33E-02
RASGRF1	No	Yes	Yes	Down	0.66	0.00072	1.5E-19	-0.72	0.11	7.09E-03	-0.60	0.04	5.02E-03	-0.59	0.11	8.92E-02	-0.48	0.12	7.68E-02	-0.87	0.15	1.97E-01	-0.56	0.21	3.91E-01	-0.54	0.10	4.02E-02	-0.41	0.03	4.00E-03
TEKT1	No	Yes	No	Down	0.66	1.83E-19	0.00012	-0.45	0.10	5.18E-02	-0.70	0.04	2.23E-04	-0.65	0.11	1.27E-02	-0.13	0.11	6.16E-01	-0.82	0.15	5.86E-03	-0.58	0.21	1.22E-01	-0.81	0.10	1.71E-03	-0.64	0.03	1.98E-05
C10ORF118	No	No	No	Down	0.66	0.001032	validated	-0.52	0.10	1.32E-01	-0.44	0.04	1.57E-02	-0.06	0.10	8.16E-01	-1.50	0.14	3.01E-03	-0.48	0.14	4.27E-01	-0.48	0.21	4.00E-01	-0.10	0.10	6.10E-01	-1.18	0.04	1.39E-05
GPR37	No	Yes	No	Down	0.66	0.00017	0.04975	-0.73	0.11	1.06E-02	-0.55	0.04	5.05E-03	-0.33	0.10	1.26E-01	-0.44	0.12	1.45E-01	-0.82	0.15	3.04E-02	-0.80	0.22	3.40E-02	-0.83	0.10	2.10E-03	-0.27	0.03	7.67E-02
MUCL1	No	Yes	No	Down	0.66	1.83E-19	1.5E-19	-1.20	0.11	4.09E-06	-0.52	0.04	4.17E-04	-0.59	0.11	1.32E-02	-0.08	0.11	6.90E-01	-0.76	0.15	5.98E-02	-0.45	0.20	9.96E-02	-0.53	0.10	1.84E-02	-0.64	0.03	5.11E-05
PARVG	No	Yes	No	Down	0.66	0.000398	0.00453	-0.88	0.11	3.92E-03	-0.62	0.04	1.19E-03	-0.67	0.11	3.11E-02	-0.72	0.12	2.67E-02	-0.28	0.14	2.69E-01	-0.82	0.22	7.48E-02	-0.58	0.10	4.46E-02	-0.18	0.03	2.41E-01
GSTO2	No	No	No	Down	0.66	1.66E-05	validated	-0.10	0.10	7.07E-01	-0.72	0.04	2.54E-04	-0.39	0.10	1.04E-01	-1.18	0.13	4.95E-04	-0.43	0.14	8.41E-02	-0.76	0.21	2.51E-02	-0.59	0.10	3.89E-02	-0.58	0.03	3.04E-03
RNPEP	No	No	No	Down	0.66	0.001216	validated	-0.35	0.10	2.79E-01	-0.50	0.04	6.66E-03	-0.27	0.10	3.88E-01	-0.86	0.12	6.94E-02	-0.24	0.14	3.90E-01	-0.55	0.21	1.42E-01	-1.22	0.10	5.74E-04	-0.74	0.03	6.81E-03
SULT2B1	No	No	No	Down	0.66	0.00011	0.01505	-0.73	0.11	1.05E-02	-0.75	0.04	8.45E-05	-0.95	0.11	3.73E-03	-0.76	0.12	1.34E-02	-0.70	0.14	4.76E-02	-0.44	0.20	3.06E-01	-0.20	0.10	5.24E-01	-0.22	0.03	2.26E-01
MEGF9	No	Yes	No	Down	0.66	0.000125	0.00023	-0.70	0.11	2.44E-02	-0.97	0.04	3.09E-05	-1.08	0.11	2.15E-03	0.00	0.11	9.88E-01	-0.52	0.14	4.73E-01	-0.49	0.21	5.42E-01	-0.39	0.10	1.09E-01	-0.57	0.03	2.16E-03
DKK4	No	Yes	No	Down	0.66	1.83E-19	0.00146	-0.93	0.11	1.24E-04	-0.45	0.04	9.55E-04	-0.65	0.11	3.79E-03	-0.54	0.12	1.67E-02	-0.59	0.14	2.02E-02	-0.65	0.21	3.11E-02	-0.59	0.10	6.94E-03	-0.34	0.03	6.54E-03
GPX4	No	Yes	No	Down	0.66	0.005728	0.00053	-0.61	0.10	9.26E-02	-0.45	0.04	3.61E-02	-0.79	0.11	4.62E-02	-0.87	0.12	5.42E-02	-0.01	0.14	9.01E-01	-0.23	0.20	3.01E-01	-1.19	0.10	2.60E-03	-0.58	0.03	6.37E-02
CAMK2N1	No	Yes	No	Down	0.66	9.89E-06	0.00113	-0.75	0.11	1.21E-02	-0.57	0.04	1.02E-03	-0.30	0.10	1.82E-01	-0.78	0.12	1.68E-02	-0.35	0.14	1.14E-01	-0.97	0.22	5.13E-03	-0.34	0.10	1.24E-01	-0.65	0.03	8.66E-05
ZNF596	No	Yes	No	Down	0.66	0.043906	0.03247	-0.51	0.10	1.45E-01	-0.45	0.04	5.89E-02	-0.52	0.11	1.14E-01	-1.04	0.13	1.04E-02	-0.83	0.15	5.34E-01	-0.44	0.20	6.92E-01	-0.70	0.10	7.05E-02	-0.23	0.03	2.64E-01
TSPAN1	No	No	No	Down	0.66	1.83E-19	validated	-0.16	0.10	4.87E-01	-0.92	0.04	9.09E-07	-0.51	0.10	3.40E-02	-0.93	0.12	1.94E-03	-0.54	0.14	4.38E-02	-0.29	0.20	2.62E-01	-0.41	0.10	8.08E-02	-0.95	0.03	1.00E-20
CST2	No	Yes	No	Down	0.66	0.000925	3.7E-05	-0.79	0.11	5.73E-03	-0.38	0.04	1.43E-02	-0.61	0.11	4.70E-02	-0.75	0.12	7.71E-03	-0.63	0.14	4.15E-01	-0.81	0.22	3.33E-01	-0.36	0.10	1.80E-01	-0.39	0.03	7.63E-03
SPATA17	No	No	No	Down	0.67	4.19E-05	validated	-0.26	0.10	3.77E-01	-0.74	0.04	7.59E-05	-0.31	0.10	1.94E-01	-0.79	0.12	2.16E-02	-0.90	0.15	1.87E-01	-0.18	0.20	7.99E-01	-0.94	0.10	6.11E-04	-0.57	0.03	9.80E-04
CUEDC1	No	No	No	Down	0.67	4.80E-05	0.03796	-0.33	0.10	2.48E-01	-0.55	0.04	4.79E-03	-0.91	0.11	6.59E-03	-0.55	0.12	1.43E-01	-0.60	0.14	2.49E-02	-0.50	0.21	1.12E-01	-0.34	0.10	2.27E-01	-0.92	0.03	2.16E-05
ZBTB45	No	No	No	Down	0.67	0.012892	validated	-1.38	0.12	1.30E-03	-0.63	0.04	2.84E-02	-0.61	0.11	2.30E-01	-0.40	0.12	3.62E-01	-0.68	0.14	1.52E-01	-0.60	0.21	3.36E-01	-0.03	0.10	9.45E-01	-0.37	0.03	1.87E-02
PACRG	No	No	No	Down	0.67	0.000102	validated	-0.29	0.10	1.94E-01	-0.62	0.04	6.71E-04	-0.33	0.10	1.51E-01	-1.00	0.13	7.03E-03	-0.87	0.15	4.03E-02	-0.45	0.20	3.07E-01	-0.57	0.10	3.78E-02	-0.56	0.03	9.29E-04
FGF20	No	Yes	No	Down	0.67	1.14E-05	1.5E-19	-0.79	0.11	9.71E-04	-0.31	0.04	2.93E-02	-0.50	0.10	2.65E-02	-0.63	0.12	1.04E-02	-0.76	0.15	3.80E-02	-0.61	0.21	5.65E-02	-0.67	0.10	5.72E-03	-0.42	0.03	3.05E-03
CPD	No	No	No	Down	0.67	9.84E-05	0.02762	-0.38	0.10	1.54E-01	-0.65	0.04	3.21E-04	-0.76	0.11	1.41E-02	-0.25	0.11	3.71E-01	-0.73	0.14	1.11E-02	-1.37	0.25	7.02E-04	-0.35	0.10	1.41E-01	-0.20	0.03	1.94E-01
CADM1	No	Yes	No	Down	0.67	0.000112	0.0009	-0.92	0.11	7.57E-04	-0.30	0.04	5.88E-02	-0.12	0.10	6.13E-01	-0.77	0.12	1.64E-02	-0.81	0.15	5.51E-03	-1.05	0.23	4.25E-03	-0.59	0.10	2.03E-02	-0.13	0.03	3.70E-01
STX17	No	No	No	Down	0.67	0.009414	validated	-0.40	0.10	2.58E-01	-0.54	0.04	2.84E-02	-0.82	0.11	2.35E-02	-1.06	0.13	4.53E-02	-0.38	0.14	5.54E-01	-0.10	0.20	8.60E-01	-1.06	0.10	3.07E-03	-0.31	0.03	1.09E-01
HEATR3	No	No	No	Down	0.67	0.027406	validated	-0.16	0.10	6.28E-01	-0.50	0.04	4.18E-02	-0.62	0.11	6.32E-02	-0.68	0.12	1.02E-01	-1.04	0.15	2.97E-02	-1.38	0.25	1.05E-02	-0.19	0.10	6.01E-01	-0.10	0.03	6.05E-01
GALNT12	No	No	No	Down	0.67	2.40E-05	validated	-0.69	0.11	7.82E-03	-0.76	0.04	1.44E-04	-0.44	0.10	7.94E-02	-0.43	0.12	1.22E-01	-1.00	0.15	2.23E-02	-0.38	0.20	3.11E-01	-0.30	0.10	1.83E-01	-0.67	0.03	2.29E-04
TTC18	No	No	No	Down	0.67	3.58E-06	validated	-0.44	0.10	5.92E-02	-0.63	0.04	2.11E-04	-0.26	0.10	2.52E-01	-1.56	0.14	2.41E-05	-0.76	0.15	8.68E-02	-0.02	0.20	9.67E-01	-0.38	0.10	8.76E-02	-0.64	0.03	1.31E-04

Supplementary Table 2. Details for 2,053 genes differentially expressed between EEC and NEEC, identified by microarray meta-analysis.

EEC - endometrioid endometrial cancer, NEEC - non-endometrioid endometrial cancer, SMD - Standardised Mean Difference, FDR - False Discovery Rate, Var - Variance

Gene	145 gene list	1253 gene list	Associated with EEC-specific survival?	Up/Down Regulated in NEEC	average standardised fold change	Microarray Meta FDR	RNASeq FDR	Individual microarray study results																							
								TCGA			E-MTAB-2532			E-GEOD-56026			GSE32507			GSE24537			E-GEOD-23518			E-GEOD-17025			E-GEOD-2109		
								SMD	Var	P-value	SMD	Var	P-value	SMD	Var	P-value	SMD	Var	P-value	SMD	Var	P-value	SMD	Var	P-value	SMD	Var	P-value	SMD	Var	P-value
DNAH5	No	No	No	Down	0.67	1.14E-05	validated	-0.11	0.10	7.24E-01	-0.79	0.04	1.02E-05	-0.54	0.11	6.19E-02	-1.07	0.13	3.43E-04	-0.53	0.14	4.42E-01	-0.40	0.20	6.43E-01	-0.78	0.10	2.39E-03	-0.47	0.03	1.24E-02
SPAG8	No	Yes	No	Down	0.67	3.97E-05	0.00154	-0.61	0.10	1.39E-02	-0.61	0.04	1.99E-04	-0.43	0.10	1.37E-01	-0.79	0.12	7.77E-03	-0.90	0.15	9.31E-02	-0.34	0.20	5.59E-01	-0.50	0.10	4.99E-02	-0.50	0.03	1.05E-03
IL7	No	No	No	Down	0.67	0.000343	validated	-0.17	0.10	4.76E-01	-0.78	0.04	2.28E-04	-0.54	0.11	3.23E-02	-0.54	0.12	6.33E-02	-0.32	0.14	4.36E-01	-0.97	0.22	2.02E-01	-0.81	0.10	1.42E-02	-0.56	0.03	2.21E-03
STEAP3	No	Yes	No	Down	0.67	0.000349	2E-05	-0.73	0.11	1.36E-02	-0.75	0.04	4.93E-05	-0.17	0.10	4.83E-01	-0.62	0.12	4.87E-02	-0.90	0.15	2.04E-02	-0.92	0.22	4.05E-02	-0.40	0.10	1.59E-01	-0.16	0.03	3.10E-01
CASP7	No	No	No	Down	0.67	0.00072	validated	-0.49	0.10	1.07E-01	-0.90	0.04	1.34E-04	-0.46	0.10	1.33E-01	-0.61	0.12	1.05E-01	-0.11	0.14	5.67E-01	-0.76	0.21	5.44E-02	-0.77	0.10	1.36E-02	-0.57	0.03	3.51E-02
DNAH11	No	Yes	No	Down	0.67	0.000584	1.5E-19	-0.87	0.11	3.30E-03	-0.71	0.04	6.45E-05	-0.37	0.10	1.90E-01	-0.32	0.11	1.77E-01	-0.73	0.14	2.34E-01	-0.67	0.21	5.44E-01	-0.73	0.10	2.39E-02	-0.25	0.03	7.98E-02
ATF4	No	No	No	Down	0.67	0.005154	validated	-0.06	0.10	8.76E-01	-0.51	0.04	6.15E-02	-0.10	0.10	7.95E-01	-1.27	0.13	2.11E-02	-0.59	0.14	2.17E-02	-0.15	0.20	4.32E-01	-1.66	0.11	1.06E-04	-0.31	0.03	3.24E-01
IL19	No	Yes	Yes	Down	0.67	6.71E-06	1.5E-19	-0.49	0.10	3.39E-02	-0.77	0.04	1.34E-05	-0.79	0.11	4.94E-03	-0.84	0.12	2.05E-03	-0.38	0.14	1.18E-01	-0.65	0.21	4.17E-02	-0.46	0.10	7.45E-02	-0.26	0.03	6.98E-02
GTF3C5	No	Yes	No	Down	0.67	0.001724	0.02139	-0.66	0.11	6.01E-02	-0.55	0.04	8.54E-03	-0.34	0.10	2.94E-01	-0.47	0.12	2.84E-01	-0.48	0.14	8.29E-02	-0.61	0.21	6.85E-02	-1.01	0.10	8.72E-03	-0.52	0.03	1.03E-02
CHIA	No	No	No	Down	0.67	0.001095	validated	-0.51	0.10	3.75E-02	-0.51	0.04	1.62E-03	-0.36	0.10	2.17E-01	-1.18	0.13	1.62E-04	-0.37	0.14	6.17E-01	-1.03	0.23	3.49E-01	-0.40	0.10	2.89E-01	-0.30	0.03	6.38E-02
UQCRH	No	Yes	No	Down	0.67	6.71E-06	1.5E-19	-0.09	0.10	7.27E-01	-0.41	0.04	1.29E-02	-0.89	0.11	2.90E-03	-0.13	0.11	6.01E-01	-0.06	0.14	6.10E-01	-0.49	0.21	8.15E-02	-1.85	0.11	2.27E-07	-0.73	0.03	1.16E-03
SCAMP4	No	Yes	No	Down	0.67	0.008518	3.5E-05	-0.82	0.11	6.65E-02	-0.27	0.04	1.05E-01	-0.77	0.11	3.84E-02	-1.31	0.14	4.91E-04	-0.11	0.14	7.31E-01	-0.49	0.21	3.58E-01	-0.68	0.10	1.29E-01	-0.21	0.03	2.17E-01
ZNF18	No	Yes	No	Down	0.67	0.038925	0.0036	-0.71	0.11	5.09E-02	-0.23	0.04	2.91E-01	-0.45	0.10	2.45E-01	-0.33	0.11	5.38E-01	-0.47	0.14	2.21E-01	-0.85	0.22	1.62E-01	-1.07	0.10	1.06E-02	-0.53	0.03	6.77E-02
C14ORF166	No	No	No	Down	0.67	0.045805	validated	-1.02	0.11	5.05E-02	-0.17	0.04	4.80E-01	-0.07	0.10	8.46E-01	-0.62	0.12	3.36E-01	-1.06	0.15	5.00E-03	-1.11	0.23	1.94E-02	-0.27	0.10	2.98E-01	-0.31	0.03	3.97E-01
ZNF282	No	No	No	Down	0.67	0.01678	0.00211	-0.45	0.10	2.92E-01	-0.62	0.04	8.54E-03	-0.36	0.10	3.34E-01	-0.10	0.11	7.76E-01	-0.64	0.14	6.65E-02	-1.32	0.24	1.69E-02	-0.74	0.10	5.22E-02	-0.40	0.03	1.41E-01
PDIA4	No	No	No	Down	0.67	0.004463	validated	-0.10	0.10	7.53E-01	-0.09	0.04	6.02E-01	-0.65	0.11	4.93E-02	-0.49	0.12	2.26E-01	-0.97	0.15	9.79E-03	-0.96	0.22	2.75E-02	-1.00	0.10	1.66E-03	-0.36	0.03	9.34E-02
PIR	No	Yes	No	Down	0.67	2.40E-05	1.5E-19	-0.92	0.11	1.40E-03	-0.65	0.04	4.38E-04	-0.71	0.11	8.63E-03	-0.55	0.12	5.85E-02	-0.19	0.14	3.87E-01	-0.61	0.21	9.57E-02	-0.45	0.10	5.84E-02	-0.53	0.03	5.08E-03
FAM81B	No	Yes	No	Down	0.67	1.83E-19	0.00868	-0.74	0.11	1.80E-03	-0.67	0.04	3.61E-05	-0.48	0.10	2.76E-02	-0.65	0.12	1.19E-02	-0.22	0.14	7.91E-01	-0.53	0.21	6.25E-01	-0.66	0.10	3.73E-03	-0.67	0.03	9.54E-06
RORC	No	No	No	Down	0.67	9.09E-05	validated	-0.01	0.10	9.82E-01	-0.80	0.04	6.18E-05	-0.54	0.11	4.04E-02	-0.93	0.12	2.73E-03	-0.51	0.14	1.43E-01	-0.97	0.22	1.42E-01	-0.42	0.10	1.08E-01	-0.46	0.03	3.11E-03
C4ORF19	No	Yes	No	Down	0.67	0.000173	0.03979	-0.41	0.10	1.01E-01	-0.87	0.04	9.31E-06	-0.01	0.10	9.70E-01	-0.88	0.12	5.30E-03	-0.50	0.14	1.53E-01	-1.35	0.25	3.22E-02	-0.22	0.10	3.29E-01	-0.38	0.03	2.24E-02
LRRC43	No	No	No	Down	0.67	0.000704	validated	-0.49	0.10	5.07E-02	-0.67	0.04	3.60E-04	-0.62	0.11	4.43E-02	-0.54	0.12	5.19E-02	-0.68	0.14	1.37E-01	-0.65	0.21	3.71E-01	-0.64	0.10	3.33E-02	-0.33	0.03	3.83E-02
HLA-DMA	No	No	No	Down	0.67	6.71E-06	validated	-0.05	0.10	8.44E-01	-0.81	0.04	2.41E-05	-0.93	0.11	1.34E-03	-0.37	0.11	1.84E-01	-0.22	0.14	2.53E-01	-0.97	0.22	4.90E-03	-0.56	0.10	2.40E-02	-0.71	0.03	8.31E-04
LGALS3	No	No	No	Down	0.67	0.00072	validated	-0.55	0.10	5.06E-02	-0.72	0.04	3.37E-04	-0.70	0.11	2.71E-02	-0.53	0.12	1.15E-01	-0.69	0.14	6.81E-02	-0.75	0.21	1.20E-01	-0.27	0.10	2.24E-01	-0.40	0.03	3.12E-02
CCDC65	No	No	No	Down	0.67	8.12E-05	validated	-0.62	0.10	1.34E-02	-0.70	0.04	3.35E-04	-0.54	0.11	2.79E-02	-0.66	0.12	2.51E-02	-0.81	0.15	5.73E-02	-0.21	0.20	6.45E-01	-0.75	0.10	1.26E-02	-0.32	0.03	2.48E-02
ZSWIM6	No	Yes	No	Down	0.67	0.017941	0.04556	-0.62	0.10	1.10E-01	-0.38	0.04	7.75E-02	-0.39	0.10	2.62E-01	-0.45	0.12	3.16E-01	-0.48	0.14	1.07E-01	-0.65	0.21	1.60E-01	-0.94	0.10	1.19E-02	-0.69	0.03	4.46E-02
VAV3	No	No	No	Down	0.67	1.14E-05	validated	-0.35	0.10	1.38E-01	-0.48	0.04	2.68E-03	-0.37	0.10	1.29E-01	-0.62	0.12	2.56E-02	-0.62	0.14	2.17E-02	-0.79	0.22	1.73E-02	-0.85	0.10	2.09E-03	-0.50	0.03	1.29E-03
ABCC2	No	No	No	Down	0.67	0.047149	validated	-0.93	0.11	5.70E-03	-0.25	0.04	1.72E-01	-0.91	0.11	7.22E-02	-0.84	0.12	1.38E-02	-0.50	0.14	5.85E-01	-0.77	0.21	5.57E-01	-0.32	0.10	4.53E-01	-0.08	0.03	5.93E-01
SLITRK6	No	Yes	No	Down	0.67	0.001462	1.5E-19	-0.97	0.11	9.47E-05	-0.38	0.04	6.61E-03	-0.39	0.10	7.52E-02	-0.28	0.11	2.12E-01	-0.78	0.15	5.69E-01	-1.18	0.23	4.50E-01	-0.26	0.10	2.00E-01	-0.33	0.03	3.02E-02
KIAA1407	No	No	No	Down	0.67	0.002404	validated	-0.66	0.11	2.16E-02	-0.39	0.04	1.66E-02	-0.25	0.10	4.56E-01	-0.46	0.12	1.32E-01	-1.02	0.15	4.23E-02	-0.56	0.21	3.35E-01	-0.45	0.10	1.45E-01	-0.78	0.03	1.06E-03
CCDC114	No	No	No	Down	0.67	0.001029	0.0279	-0.37	0.10	3.76E-01	-0.08	0.04	7.90E-01	-0.48	0.10	9.39E-02	-0.91	0.12	1.92E-02	-0.68	0.14	1.67E-01	-0.69	0.21	3.35E-01	-0.76	0.10	2.12E-03	-0.60	0.03	1.15E-04
DTNB	No	Yes	No	Down	0.67	0.009523	0.00167	-0.85	0.11	2.85E-02	-0.56	0.04	4.49E-03	-0.29	0.10	4.46E-01	-0.14	0.11	6.83E-01	-0.33	0.14	3.28E-01	-1.23	0.24	8.75E-02	-0.75	0.10	1.02E-01	-0.44	0.03	1.13E-02
PLEKHG1	No	No	No	Down	0.67	0.000538	validated	-0.31	0.10	2.44E-01	-0.61	0.04	2.33E-03	-0.25	0.10	3.38E-01	-0.33	0.11	2.21E-01	-0.84	0.15	2.83E-02	-0.73	0.21	8.76E-02	-0.86	0.10	2.53E-03	-0.63	0.03	5.68E-03
TMED7	No	No	No	Down	0.67	0.005864	validated	-0.76	0.11	4.65E-02	-0.33	0.04	1.48E-01	-0.91	0.11	2.12E-02	-0.45	0.12	3.24E-01	-0.96	0.15	4.40E-03	-0.28	0.20	3.19E-01	-0.03	0.10	8.93E-01	-0.85	0.03	6.70E-03
SCNN1B	No	Yes	No	Down	0.67	1.83E-19	1.5E-19	-0.55	0.10	4.78E-02	-0.97	0.04	5.91E-06	-0.68	0.11	1.02E-02	-0.06	0.11	8.27E-01	-0.23	0.14	3.03E-01	-0.50	0.21	1.22E-01	-0.74	0.10	1.49E-02	-0.85	0.03	1.14E-06
FMO5	No	Yes	No	Down	0.67	0.000138	1.5E-19	-0.80	0.11	5.34E-03	-0.77	0.04	2.20E-04	-0.87	0.11	2.07E-03	-0.52	0.12	4.94E-02	-0.40	0.14	2.08E-01	-0.69	0.21	1.48E-01	-0.26	0.10	2.82E-01	-0.25	0.03	1.02E-01
GALNT6	No	No	No	Down	0.67	1.66E-05	validated	-0.16	0.10	5.62E-01	-0.56	0.04	1.50E-03	-0.13	0.10	5.56E-01	-1.45	0.14	5.11E-05	-0.38	0.14	1.79E-01	-0.50	0.21	1.37E-01	-0.87	0.10	4.02E-03	-0.52	0.03	9.98E-04
ENTPD7	No	No	No	Down	0.67	0.006556	validated	-0.36	0.10	2.04E-01	-0.66	0.04	2.83E-03	-1.15	0.11	3.55E-03	-0.99	0.13	7.12E-03	-0.32	0.14	3.91E-01	-0.77	0.21	2.97E-01	-0.23	0.10	4.32E-01	-0.07	0.03	7.06E-01
ADAMTS8	No	Yes	No	Down	0.67	1.83E-19	1.5E-19	-0.75	0.11	4.59E-03	-0.80	0.04	2.18E-05	-0.50	0.10	3.57E-02	-0.30	0.11	2.26E-01	-0.41	0.14	1.46E-01	-0.17	0.20	7.69E-01	-1.11	0.10	2.25E-05	-0.52	0.03	1.03E-03
DTX2	No	Yes	No	Down	0.67	0.008811	0.00415	-0.11	0.10	6.97E-01	-0.41	0.04	9.85E-02	-0.87	0.11	2.79E-02	-0.54	0.12	1.48E-01	-0.63	0.14	3.51E-02	-1.26	0.24	7.91E-03	-0.29	0.10	5.23E-01	-0.43	0.03	2.55E-02
SLC7A11	No	Yes	No	Down	0.67	0.000148	0.00832	-1.49	0.12	3.95E-05	-0.35	0.04	3.09E-02	-0.44	0.10	6.16E-02	-0.62	0.12	1.77E-02	-0.29	0.14	6.59E-01	-0.62	0.21	2.60E-01	-0.19	0.10	3.65E-01	-0.54	0.03	1.42E-03
CDK10	No	Yes	No	Down	0.67	0.00224	0.00868	-0.62	0.10	1.57E-01	-0.38	0.04	7.21E-02	-0.20	0.10	5.47E-01	-1.44	0.14	3.02E-03	-0.23	0.14	2.60E-01	-0.52	0.21	9.93E-02	-0.61	0.10	3.76E-02	-0.53	0.03	2.20E-03
MVD	No	Yes	No	Down	0.67	0.005655	2E-05	-0.91	0.11	1.52E-02	-0.75	0.04	2.73E-03	-0.66	0.11	1.24E-01	-0.16	0.11	6.91E-01	-0.43	0.14	2.47E-01	-1.12	0.23	9.38E-02	-0.01	0.10	9.75E-01	-0.50	0.03	4.54E-03
ODF2L	No	Yes	No	Down	0.68	0.004701	0.02861	-0.47	0.10	1.86E-01	-0.39	0.04	4.77E-02	-0.17	0.10	5.23E-01	-0.89	0.12	1.45E-02	-0.92	0.15	1.75E-02	-0.74	0.21	8.39E-02	-0.93	0.10	2.65E-03	-0.02	0.03	9.08E-01
AGBL2	No	Yes	No	Down	0.68	0.00096	0.01984	-0.47	0.10	4.78E-02	-0.50	0.04	2.05E-03	-0.39	0.10	1.04E-01	-0.36	0.11	1.70E-01	-1.15	0.16	3.30E-01	-0.44	0.20	7.19E-01	-0.65	0.10	6.44E-03	-0.58	0.03	2.39E-03
NRIP1	No	No	No	Down	0.68	9.98E-05	0.02526	-0.01	0.10	9.66E-01	-0.72	0.04	4.37E-04	-0.40	0.10	1.50E-01	-0.46	0.12	1.13E-01	-0.75	0.14	1.43E-02	-0.53	0.21	8.86E-02	-1.03	0.10	1.81E-03	-0.62	0.03	3.42E-03
SLC35A4	No	No	No	Down	0.68	0.020037	validated	-0.46	0.10	3.09E-01	-0.16	0.04	4.83E-01	-1.13	0.11	1.38E-02	-0.17	0.11	7.38E-01	-0.03	0.14	8.25E-01	-1.25	0.24	6.39E-03	-0.66	0.10	8.02E-02	-0.65	0.03	1.35E-02
CHGA	No	No	No	Down	0.68	1.83E-19	0.00119	-0.74	0.11	2.62E-03	-0.55	0.04	3.69E-04	-0.91	0.11	1.47E-03	-0.76	0.12	7.34E-03	-0.51	0.14	4.44E-02	-0.12	0.20	6.32E-01	-0.38	0.10	8.21E-02	-0.55	0.03	1.77E-04
RPS19BP1	No	No	No	Down	0.68	0.01809	validated	-0.54	0.10	1.74E-01	-0.45	0.04	6.21E-02	-0.70	0.11	9.77E-02	-0.36	0.11	3.82E-01	-0.49	0.14	1.11E-01	-0.21	0.20	4.09E-01	-1.39	0.11	1.42E-03	-0.37	0.03	2.53E-01
LY6G5C	No	Yes	No	Down	0.68	0.047329	1.5E-19	-0.20	0.10	6.65E-01	-0.42	0.04	1.01E-01	-1.37	0.12	1.39E-03	-0.69	0.12	8.23E-02	-1.07	0.15	1.47E-01	-0.19	0.20	7.81E-01	-0.31	0.10	3.25E-01	-0.26	0.03	3.02E-01
DENND2D	No	No	No	Down	0.68	0.00169	validated	-0.12	0.10	6.61E-01	-0.69	0.04	7.00E-04	-0.28	0.10	3.25E-01	-0.93	0.12	7.52E-03	-0.96	0.15	9.73E-03	-1.17	0.23	8.98E-03	-0.25	0.10	3.77E-01	-0.11	0.03	5.68E-01
ARHGAP12	No	No	No	Down	0.68	0.037911	validated	-0.27	0.10	4.26E-01	-0.53	0.04	3.17E-02	-0.31	0.10	3.53E-01	-1.19	0.13	3.51E-02	-0.57	0.14	2.12E-01	-0.74	0.21	1.54E-01	-0.10	0.10	7.41E-01	-0.80	0.03	1.15E-02
C10ORF93	No	No	No	Down	0.68	0.031273	validated	-0.47	0.10	1.17E-01	-0.52	0.04	2.21E-02	-0.53	0.11	3.56E-02	-0.61	0.12	3.21E-02	-1.27	0.16	2.91E-01	-0.60	0.21	5.85E-01	-0.38	0.10	1.30E-01	-0.12	0.03	4.22E-01
UBB	No	Yes	No	Down	0.68	6.46E-05	1.5E-19	-1.44	0.12	5.13E-05	-0.56	0.04	8.53E-03	-0.25	0.10	3.07E-01	-0.07	0.11	8.04E-01	-1.00	0.15	7.29E-04	-0.04	0.20	7.59E-01	-0.83	0.10	1.80E-03	-0.28	0.03	2.46E-01
RIBC1	No	Yes	No	Down	0.68	0.000372	0.00019	-0.60	0.10	6.06E-02	-0.91	0.04	5.45E-06	-0.57	0.11	4.62E-02	-0.53	0.12	9.75E-02	-0.22	0.14	7.58E-01	-0.70	0.21	5.94E-01	-0.60	0.10	3.72E-02	-0.36	0.03	2.86E-02
P2RY14	No	Yes	No	Down	0.68	9.28E-05	1.5E-19	-0.72	0.11	1.42E-02	-0.68	0.04	1.47E-04	-0.52	0.11	3.92E-02	-0.15	0.11	6.09E-01	-0.38	0.14	3.50E-01	-0.66	0.21	6.79E-01	-0.64	0.10	1.07E-02	-0.75	0.03	3.80E-04
PEX7	No	No	No	Down	0.68	0.000611	validated	-0.58	0.10	5.94E-02	-0.81	0.04	3.88E-04	-0.50	0.10	9.50E-02	-0.17	0.11	6.55E-01	-0.66	0.14	5.90E-02	-0.59	0.21	1.30E-01	-0.39	0.10	1.36E-01	-0.79	0.03	1.35E-03
FBXW4	No	No	No	Down	0.68	0.00536	validated	-0.35	0.10	2.48E-01	-0.74	0.04	1.07E-03	-0.44	0.10	1.88E-01	-0.07	0.11	8.69E-01	-0.80	0.15	3.03E-02	-1.04	0.23	3.48E-02	-0.14	0.10	6.63E-01	-0.90	0.03	1.05E-02
MLKL	No	Yes	No	Down	0.68	0.000173	0.00043	-0.50	0.10	7.75E-02	-1.04	0.04	6.59E-06	-0.80	0.11	1.04E-02	-0.78	0.12	1.80E-02	-0.19	0.14	3.93E-01	-0.45	0.21	1.81E-01	-0.34	0.10	2.30E-01	-0.36	0.03	1.12E-01
ATP6V0A4	No	Yes	No	Down	0.68	6.71E-06	1.5E-19	-0.56	0.10	1.60E-02	-0.88	0.04	2.50E-06	-0.50	0.10	5.23E-02	-0.23	0.11	3.32E-01	-0.26	0.14	3.22E-01	-0.83	0.22	1.49E-01	-0.55	0.10	5.87E-02	-0.66	0.03	7.41E-05
DAPK2	No	Yes	No	Down	0.68	0.009253	0.00146	-0.56	0.10	5.27E-02	-0.59	0.04	1.82E-03	-0.36	0.10	2.99E-01	-0.38	0.11	2.15E-01	-0.48	0.14	3.27E-01	-1.25	0.24	9.14E-02	-0.51	0.10	8.14E-02	-0.31	0.03	7.96E-02
NFKB1	No	No	No	Down	0.68	0.003047	validated	-0.20	0.10	6.10E-01	-1.04	0.04	1.26E-04	-0.31	0.10	3.70E-01	-0.62	0.12	1.65E-01	-0.41	0.14	9.52E-02	-1.21	0.24	1.94E-03	-0.37	0.10	2.85E-01	-0.28	0.03	3.46E-01
UBLCP1	No	No	No	Down	0.68	0.013636	validated	-1.34	0.12	2.92E-03	-0.07	0.04	7.17E-01	-0.46	0.10	1.85E-01	-0.03	0.11	9.39E-01	-0.90	0.15	5.46E-02	-0.36	0.20	3.89E-01	-1.06	0.10	1.09E-03	-0.23	0.03	3.80E-01
MTERFD3	No	Yes	No	Down	0.68	0.040869	0.0364	-0.99	0.11	1.04E-02	-0.56	0.04	1.22E-02	-0.59	0.11	5.25E-02	-0.41	0.12	3.15E-01	-0.44	0.14	7.37E-01	-0.78	0.22	6.48E-01	-0.38	0.10	1.98E-01	-0.31	0.03	2.88E-01
MSMB	No	Yes	No	Down	0.68	0.000158	4.1E-05	-0.75	0.11	6.71E-03	-0.58	0.04	8.47E-04	-0.53	0.11	8.49E-02	-0.68	0.12	1.05E-02	-0.48	0.14	1.57E-01	-0.68	0.21	8.70E-02	-0.37	0.10	1.86E-01	-0.37	0.03	9.72E-03
EFHB	No	No	No	Down	0.68	0.000246	validated	-0.26	0.10	2.60E-01	-0.65	0.04	2.26E-04	-0.39	0.10	8.70E-02	-0.71	0.12	1.45E-02	-0.83	0.15	4.80E-02	-0.77	0.21	2.80E-01	-0.42	0.10	7.94E-02	-0.41	0.03	4.21E-03
FUT3	No	Yes	No	Down	0.68	2.19E-05	0.00061	-0.17	0.10	4.46E-01	-0.95	0.04	1.36E-06	-0.90	0.11	6.31E-03	-0.83	0.12	4.08E-03	-0.53	0.14	1.05E-01	-0.57	0.21	1.00E-01	-0.11	0.10	6.87E-01	-0.37	0.03	2.40E-02
SCYL3	No	No	No	Down	0.68	0.008715	validated	-0.43	0.10	1.92E-01	-0.83	0.04	1.03E-03	-0.38	0.10	2.30E-01	-0.31	0.11	4.34E-01	-0.71	0.14	9.14E-02	-0.64	0.21	2.47E-01	-0.52	0.10	1.06E-01	-0.60	0.03	2.17E-02
LRP11	No	Yes	No	Down	0.68	4.80E-05	0.0024	-0.90	0.11	3.75E-03	-0.78	0.04	7.25E-05	-0.71	0.11	2.76E-02	-0.05	0.11	8.37E-01	-0.58	0.14	7.06E-02	-0.37	0.20	2.46E-01	-0.18	0.10	4.45E-01	-0.85	0.03	2.48E-04
POU5F1P3	No	No	No	Down	0.68	0.000611	validated	-0.03	0.10	9.21E-01	-0.17	0.04	2.46E-01	-0.69	0.11	2.83E-02	-1.11	0.13	7.50E-04	-0.56	0.14	9.69E-02	-0.63	0.21	1.10E-01	-0.59	0.10	4.88E-02	-0.63	0.03	8.04E-04

Supplementary Table 2. Details for 2,053 genes differentially expressed between EEC and NEEC, identified by microarray meta-analysis.

EEC - endometrioid endometrial cancer, NEEC - non-endometrioid endometrial cancer, SMD - Standardised Mean Difference, FDR - False Discovery Rate, Var - Variance

Gene	145 gene list	1253 gene list	Associated with EEC-specific survival?	Up/Down Regulated in NEEC	average standardised fold change	Microarray Meta FDR	RNASeq FDR	Individual microarray study results																							
								TCGA			E-MTAB-2532			E-GEOD-56026			GSE32507			GSE24537			E-GEOD-23518			E-GEOD-17025			E-GEOD-2109		
								SMD	Var	P-value	SMD	Var	P-value	SMD	Var	P-value	SMD	Var	P-value	SMD	Var	P-value	SMD	Var	P-value	SMD	Var	P-value	SMD	Var	P-value
CD59	No	No	No	Down	0.68	0.000546	0.01249	-0.35	0.10	2.79E-01	-0.63	0.04	1.08E-03	-0.14	0.10	5.91E-01	-0.36	0.11	2.73E-01	-0.50	0.14	1.73E-01	-0.89	0.22	7.39E-02	-0.93	0.10	1.85E-03	-0.60	0.03	1.36E-03
ZBTB7A	No	Yes	No	Down	0.68	0.008616	0.00226	-0.86	0.11	9.00E-03	-0.31	0.04	6.54E-02	-0.75	0.11	5.38E-02	-1.54	0.14	5.64E-04	-0.32	0.14	6.96E-01	-0.26	0.20	7.44E-01	-0.16	0.10	5.04E-01	-0.20	0.03	2.13E-01
TLR3	No	No	No	Down	0.68	6.66E-05	validated	-0.69	0.11	9.02E-03	-0.96	0.04	6.59E-06	-0.67	0.11	1.01E-02	-0.28	0.11	3.02E-01	-0.34	0.14	3.68E-01	-0.75	0.21	2.39E-01	-0.19	0.10	4.00E-01	-0.52	0.03	4.38E-03
DNAJC17	No	Yes	No	Down	0.68	0.018482	0.00951	-0.25	0.10	4.75E-01	-1.03	0.04	9.75E-04	-0.39	0.10	3.06E-01	-0.14	0.11	7.85E-01	-0.35	0.14	2.58E-01	-0.98	0.22	1.10E-01	-0.77	0.10	6.50E-02	-0.50	0.03	3.41E-02
ORM1	No	Yes	No	Down	0.68	6.26E-05	1.5E-19	-0.88	0.11	9.55E-04	-0.71	0.04	2.43E-05	-0.55	0.11	2.44E-02	-0.68	0.12	1.47E-02	-0.26	0.14	2.50E-01	-0.91	0.22	1.19E-01	-0.35	0.10	1.56E-01	-0.07	0.03	5.94E-01
PPAPDC2	No	No	No	Down	0.68	0.004537	validated	-0.86	0.11	1.32E-02	-0.68	0.04	4.27E-03	-0.05	0.10	8.65E-01	-0.33	0.11	2.91E-01	-0.91	0.15	7.35E-02	-0.17	0.20	5.57E-01	-0.63	0.10	3.03E-02	-0.74	0.03	1.25E-02
PZP	No	Yes	Yes	Down	0.68	0.000497	1.5E-19	-0.46	0.10	8.47E-02	-0.83	0.04	3.61E-05	-0.70	0.11	1.56E-02	-0.42	0.12	1.01E-01	-0.67	0.14	6.08E-02	-0.58	0.21	4.75E-01	-0.42	0.10	9.99E-02	-0.28	0.03	8.82E-02
STOML3	No	Yes	No	Down	0.68	0.001032	1.9E-05	-0.52	0.10	3.59E-02	-0.72	0.04	2.55E-04	-0.53	0.11	5.60E-02	-0.33	0.11	1.94E-01	-0.69	0.14	8.75E-02	-0.55	0.21	4.84E-01	-0.74	0.10	2.84E-02	-0.30	0.03	6.18E-02
DUSP10	No	Yes	No	Down	0.68	0.005005	0.04506	-0.45	0.10	1.37E-01	-0.49	0.04	9.19E-03	-0.74	0.11	1.70E-02	-0.61	0.12	7.92E-02	-0.57	0.14	1.16E-01	-0.68	0.21	1.55E-01	-0.56	0.10	4.05E-02	-0.27	0.03	2.21E-01
SMPD2	No	No	No	Down	0.69	0.00766	validated	-0.77	0.11	2.43E-02	-0.74	0.04	1.08E-03	-0.46	0.10	2.00E-01	-0.64	0.12	8.82E-02	-0.52	0.14	2.10E-01	-0.51	0.21	2.49E-01	-0.51	0.10	1.28E-01	-0.21	0.03	2.39E-01
RBKS	No	No	No	Down	0.69	0.001899	validated	-0.32	0.10	2.45E-01	-0.79	0.04	2.36E-04	-0.24	0.10	4.26E-01	-0.60	0.12	1.23E-01	-0.70	0.14	5.94E-02	-0.79	0.22	9.91E-02	-0.27	0.10	3.02E-01	-0.65	0.03	4.94E-03
SYNJ2BP	No	No	No	Down	0.69	0.010856	validated	-0.44	0.10	2.24E-01	-0.60	0.04	1.40E-02	-0.08	0.10	7.67E-01	-1.33	0.14	2.29E-02	-0.35	0.14	1.44E-01	-0.72	0.21	3.89E-02	-0.39	0.10	1.53E-01	-0.45	0.03	3.84E-02
SPEF2	No	No	No	Down	0.69	2.85E-05	validated	-0.42	0.10	1.08E-01	-0.67	0.04	2.26E-04	-0.45	0.10	6.49E-02	-0.85	0.12	4.51E-03	-0.43	0.14	3.88E-01	-0.27	0.20	5.99E-01	-0.77	0.10	2.17E-03	-0.49	0.03	1.54E-03
SSFA2	No	Yes	No	Down	0.69	0.000936	0.00037	-0.65	0.11	4.06E-02	-0.81	0.04	3.91E-04	-0.42	0.10	1.11E-01	-1.20	0.13	1.43E-03	-0.42	0.14	2.50E-01	-0.33	0.20	4.38E-01	-0.15	0.10	5.12E-01	-0.37	0.03	4.12E-02
C16ORF55	No	Yes	No	Down	0.69	0.000482	0.00504	-0.99	0.11	9.71E-03	-0.56	0.04	4.26E-03	-0.14	0.10	6.21E-01	-0.51	0.12	1.02E-01	-0.57	0.14	6.90E-01	-0.33	0.20	7.43E-01	-0.22	0.10	4.19E-01	-1.03	0.04	2.04E-06
RIPK2	No	No	No	Down	0.69	0.001465	validated	-0.39	0.10	1.77E-01	-0.64	0.04	1.51E-03	-0.26	0.10	3.43E-01	-0.91	0.12	2.14E-02	-0.25	0.14	2.42E-01	-1.03	0.23	6.93E-03	-0.45	0.10	1.34E-01	-0.42	0.03	3.50E-02
ARRDC3	No	No	No	Down	0.69	0.003016	validated	-0.81	0.11	2.10E-02	-0.47	0.04	1.95E-02	-0.08	0.10	7.97E-01	-0.18	0.11	5.87E-01	-0.91	0.15	6.84E-02	-0.25	0.20	5.02E-01	-0.97	0.10	1.54E-03	-0.69	0.03	8.18E-03
HSDL1	No	Yes	No	Down	0.69	0.012552	0.00015	-0.58	0.10	6.16E-02	-0.55	0.04	1.16E-02	-0.26	0.10	3.95E-01	-0.22	0.11	5.16E-01	-0.62	0.14	1.54E-01	-0.60	0.21	2.08E-01	-1.00	0.10	5.76E-03	-0.53	0.03	9.62E-02
TNFRSF14	No	No	No	Down	0.69	0.000225	validated	-0.73	0.11	1.05E-02	-0.83	0.04	4.73E-05	-0.67	0.11	3.64E-02	-0.78	0.12	2.08E-02	-0.24	0.14	2.22E-01	-0.49	0.21	1.03E-01	-0.12	0.10	6.46E-01	-0.47	0.03	5.23E-02
TALDO1	No	No	No	Down	0.69	0.000527	validated	-0.44	0.10	1.80E-01	-0.81	0.04	3.11E-04	-0.41	0.10	2.02E-01	-0.24	0.11	4.96E-01	-0.37	0.14	1.01E-01	-0.76	0.21	1.97E-02	-0.67	0.10	1.76E-02	-0.64	0.03	7.38E-03
RPL36	No	No	No	Down	0.69	0.011354	0.00277	-0.60	0.10	1.44E-01	-0.34	0.04	1.40E-01	-0.20	0.10	5.90E-01	-0.71	0.12	1.73E-01	-0.13	0.14	4.13E-01	-0.93	0.22	5.73E-03	-1.07	0.10	2.45E-03	-0.35	0.03	1.70E-01
SDSL	No	No	No	Down	0.69	0.001923	validated	-0.05	0.10	8.63E-01	-0.52	0.04	8.68E-03	-0.74	0.11	2.06E-02	-0.31	0.11	4.07E-01	-0.99	0.15	7.99E-03	-0.90	0.22	2.30E-02	-0.39	0.10	1.56E-01	-0.40	0.03	1.52E-02
SND1	No	No	No	Down	0.69	0.03172	0.01377	-1.08	0.11	2.87E-02	-0.63	0.04	1.27E-02	-0.36	0.10	3.11E-01	-0.63	0.12	2.33E-01	-0.19	0.14	3.31E-01	-0.37	0.20	1.93E-01	-0.81	0.10	4.27E-02	-0.23	0.03	3.97E-01
OAZ1	No	No	No	Down	0.69	0.003047	0.00086	-0.54	0.10	1.83E-01	-0.69	0.04	2.18E-02	-0.49	0.10	1.70E-01	-0.50	0.12	3.71E-01	-0.21	0.14	2.49E-01	-0.33	0.20	1.83E-01	-1.09	0.10	7.54E-05	-0.43	0.03	9.92E-02
DNAH7	No	No	No	Down	0.69	0.001976	validated	-0.40	0.10	1.32E-01	-0.87	0.04	9.31E-06	-0.26	0.10	3.05E-01	-0.67	0.12	4.25E-02	-0.85	0.15	1.63E-01	-0.62	0.21	4.08E-01	-0.45	0.10	8.21E-02	-0.15	0.03	3.36E-01
CFTR	No	No	No	Down	0.69	0.000432	validated	-0.47	0.10	2.64E-02	-0.40	0.04	3.19E-03	-0.33	0.10	1.09E-01	-0.53	0.12	2.53E-02	-1.02	0.15	1.23E-02	-0.99	0.22	4.31E-02	-0.29	0.10	1.49E-01	-0.24	0.03	5.35E-02
MYRIP	No	Yes	No	Down	0.69	8.71E-05	0.01067	-0.79	0.11	2.50E-03	-0.52	0.04	1.96E-03	-0.54	0.11	2.57E-02	-0.98	0.13	1.03E-03	-0.21	0.14	4.19E-01	-0.60	0.21	1.64E-01	-0.22	0.10	3.28E-01	-0.41	0.03	1.47E-02
DEFB1	No	No	No	Down	0.69	1.83E-19	validated	-0.35	0.10	9.38E-02	-0.61	0.04	6.36E-05	-0.44	0.10	4.36E-02	-1.05	0.13	2.33E-04	-0.18	0.14	2.96E-01	-0.62	0.21	3.59E-02	-0.34	0.10	9.60E-02	-0.66	0.03	1.27E-05
CEACAM5	No	Yes	Yes	Down	0.69	5.83E-05	3.1E-05	-0.56	0.10	2.97E-02	-0.69	0.04	1.39E-04	-0.88	0.11	6.84E-04	-0.14	0.11	5.75E-01	-0.53	0.14	8.68E-02	-0.55	0.21	3.88E-01	-0.56	0.10	2.00E-02	-0.35	0.03	2.02E-02
GTF3C6	No	No	No	Down	0.69	0.019714	validated	-0.66	0.11	7.63E-02	-0.38	0.04	6.81E-02	-1.23	0.12	7.88E-03	-0.25	0.11	5.59E-01	-0.59	0.14	5.64E-02	-0.71	0.21	5.08E-02	-0.02	0.10	9.49E-01	-0.42	0.03	1.33E-01
TNFRSF10A	No	Yes	No	Down	0.69	0.002479	5.6E-05	-0.37	0.10	2.20E-01	-0.74	0.04	7.63E-04	-0.41	0.10	1.94E-01	-0.90	0.12	6.55E-03	-0.45	0.14	1.81E-01	-0.87	0.22	5.05E-02	-0.14	0.10	5.80E-01	-0.38	0.03	4.51E-02
ADRA2A	No	Yes	Yes	Down	0.69	9.89E-06	1.5E-19	-1.23	0.12	2.07E-05	-0.68	0.04	9.00E-05	-0.19	0.10	3.75E-01	-0.28	0.11	2.43E-01	-0.33	0.14	2.45E-01	-0.45	0.21	1.84E-01	-0.57	0.10	1.79E-02	-0.52	0.03	1.98E-03
CTNNA2	No	No	No	Down	0.69	7.73E-05	0.00486	-0.47	0.10	3.48E-02	-0.49	0.04	1.69E-03	-0.70	0.11	3.79E-03	-0.13	0.11	5.70E-01	-0.95	0.15	2.22E-02	-0.42	0.20	3.22E-01	-0.62	0.10	1.07E-02	-0.47	0.03	3.33E-03
LRRC36	No	Yes	Yes	Down	0.69	0.000556	1.5E-19	-0.73	0.11	8.47E-03	-0.65	0.04	3.04E-04	-0.22	0.10	5.20E-01	-1.02	0.13	1.41E-03	-0.55	0.14	5.39E-01	-0.26	0.20	7.79E-01	-0.41	0.10	1.60E-01	-0.43	0.03	1.27E-02
POLR1D	No	No	No	Down	0.69	0.007556	validated	-0.74	0.11	6.93E-02	-0.19	0.04	2.93E-01	-0.30	0.10	3.33E-01	-0.61	0.12	1.04E-01	-0.82	0.15	4.21E-03	-0.45	0.21	1.02E-01	-0.59	0.10	5.12E-01	-0.53	0.03	4.69E-02
CDCA7	No	Yes	No	Down	0.69	0.000528	0.0061	-0.75	0.11	8.18E-03	-0.42	0.04	1.14E-02	-0.08	0.10	7.44E-01	-0.09	0.11	7.40E-01	-0.94	0.15	2.10E-03	-1.17	0.23	2.21E-03	-0.68	0.10	1.08E-02	-0.10	0.03	5.67E-01
PKP2	No	No	No	Down	0.69	0.003664	validated	-0.30	0.10	2.93E-01	-0.19	0.04	2.39E-01	-0.27	0.10	2.73E-01	-0.55	0.12	1.07E-01	-1.23	0.16	1.77E-03	-1.18	0.23	9.30E-03	-0.03	0.10	8.84E-01	-0.49	0.03	4.35E-03
AMFR	No	No	No	Down	0.69	0.000362	0.0486	-0.77	0.11	1.63E-02	-0.54	0.04	2.56E-03	-1.23	0.12	7.34E-04	-0.39	0.12	2.01E-01	-0.02	0.14	8.74E-01	-0.40	0.20	1.69E-01	-0.37	0.10	1.22E-01	-0.49	0.03	6.27E-03
HIF1A	No	No	No	Down	0.69	0.010699	0.04094	-1.04	0.11	4.44E-03	-0.19	0.04	3.48E-01	-0.19	0.10	5.97E-01	-0.58	0.12	1.43E-01	-0.70	0.14	3.10E-02	-0.18	0.20	5.11E-01	-0.54	0.10	1.15E-01	-0.78	0.03	7.17E-03
TRDMT1	No	Yes	No	Down	0.69	0.017566	0.02012	-0.37	0.10	1.83E-01	-0.15	0.04	4.39E-01	-0.54	0.11	5.74E-02	-0.16	0.11	6.11E-01	-1.29	0.16	5.12E-02	-0.70	0.21	2.67E-01	-0.42	0.10	1.12E-01	-0.58	0.03	1.40E-03
C19ORF25	No	Yes	No	Down	0.69	0.026462	0.00162	-1.09	0.11	6.52E-03	-0.80	0.04	2.47E-03	-0.65	0.11	8.11E-02	-0.25	0.11	5.93E-01	-0.28	0.14	4.76E-01	-0.69	0.21	2.85E-01	-0.25	0.10	4.62E-01	-0.19	0.03	3.64E-01
ALPPL2	No	No	No	Down	0.70	1.14E-05	validated	-0.10	0.10	6.30E-01	-0.71	0.04	1.23E-05	-0.79	0.11	2.52E-03	-0.95	0.13	1.59E-03	-0.58	0.14	6.93E-02	-0.74	0.21	4.24E-02	-0.02	0.10	9.29E-01	-0.32	0.03	1.61E-02
LRRC18	No	Yes	No	Down	0.70	0.008578	1.5E-19	-0.67	0.11	2.95E-02	-0.85	0.04	1.40E-03	-0.67	0.11	3.51E-02	-0.03	0.11	9.16E-01	-0.53	0.14	3.17E-01	-0.42	0.20	7.00E-01	-0.70	0.10	6.61E-02	-0.33	0.03	5.04E-02
RFX3	No	Yes	No	Down	0.70	0.003332	0.0375	-0.05	0.10	8.65E-01	-0.58	0.04	1.25E-02	-0.24	0.10	3.46E-01	-0.85	0.12	5.43E-02	-0.30	0.14	5.76E-01	-0.52	0.21	4.17E-01	-1.02	0.10	6.32E-04	-0.62	0.03	3.47E-03
SCOC	No	No	No	Down	0.70	0.004346	validated	-0.74	0.11	1.59E-02	-0.27	0.04	1.12E-01	-0.44	0.10	1.17E-01	-0.01	0.11	9.80E-01	-0.75	0.14	2.49E-02	-1.29	0.24	5.78E-03	-0.11	0.10	6.25E-01	-0.60	0.03	1.07E-02
TTLL7	No	Yes	No	Down	0.70	0.000815	1.5E-19	-0.70	0.11	5.90E-02	-0.61	0.04	1.30E-03	-0.58	0.11	2.69E-02	-0.01	0.11	9.57E-01	-0.39	0.14	3.87E-01	-0.62	0.21	2.34E-01	-1.11	0.10	8.68E-05	-0.16	0.03	2.73E-01
C1ORF87	No	No	No	Down	0.70	0.006103	0.04484	-0.29	0.10	1.88E-01	-0.43	0.04	6.17E-03	-0.67	0.11	1.89E-02	-0.23	0.11	3.70E-01	-0.78	0.15	6.37E-02	-0.89	0.22	4.18E-01	-0.58	0.10	6.28E-02	-0.31	0.03	3.20E-02
IL17RB	No	Yes	No	Down	0.70	0.001351	1.5E-05	-0.79	0.11	5.49E-03	-0.56	0.04	1.15E-02	-0.40	0.10	1.55E-01	-0.83	0.12	8.48E-03	-0.41	0.14	1.74E-01	-0.26	0.20	4.72E-01	-0.65	0.10	1.95E-02	-0.26	0.03	1.01E-01
HSD17B12	No	No	No	Down	0.70	0.00305	validated	-0.87	0.11	1.63E-02	-0.39	0.04	5.14E-02	-0.50	0.10	1.11E-01	-0.11	0.11	7.81E-01	-1.18	0.16	4.23E-04	-1.01	0.23	4.98E-03	-0.08	0.10	7.53E-01	-0.04	0.03	7.49E-01
C22ORF32	No	Yes	No	Down	0.70	0.000173	8.9E-06	-0.62	0.10	1.01E-01	-1.06	0.04	2.04E-05	-0.60	0.11	7.20E-02	-0.21	0.11	5.45E-01	-0.25	0.14	3.82E-01	-0.10	0.20	6.57E-01	-0.79	0.10	4.12E-03	-0.52	0.03	2.11E-03
PAK1	No	No	No	Down	0.70	0.006559	0.00411	-0.06	0.10	8.55E-01	-0.61	0.04	4.52E-03	-0.47	0.10	1.12E-01	-0.01	0.11	9.81E-01	-1.07	0.15	1.42E-02	-0.96	0.22	4.16E-02	-0.61	0.10	2.98E-02	-0.37	0.03	6.92E-02
P4HB	No	No	No	Down	0.70	0.0117	0.02158	-0.42	0.10	2.41E-01	-0.30	0.04	1.11E-01	-0.69	0.11	4.01E-02	-1.22	0.13	1.85E-02	-0.27	0.14	1.85E-01	-0.25	0.20	2.67E-01	-0.70	0.10	1.97E-02	-0.30	0.03	1.13E-01
CCDC37	No	Yes	No	Down	0.70	2.64E-05	0.00224	-0.39	0.10	8.21E-02	-0.62	0.04	8.68E-05	-0.47	0.10	1.12E-01	-0.65	0.12	1.01E-02	-0.42	0.14	3.18E-01	-0.34	0.20	6.55E-01	-0.66	0.10	2.31E-02	-0.59	0.03	1.40E-04
CCDC40	No	Yes	No	Down	0.70	0.0122	0.00207	-0.59	0.10	2.43E-02	-0.43	0.04	9.34E-03	-0.86	0.11	1.82E-02	-0.72	0.12	1.63E-02	-0.25	0.14	8.34E-01	-1.03	0.23	2.86E-01	-0.03	0.10	8.93E-01	-0.23	0.03	1.68E-01
FBXL5	No	Yes	No	Down	0.70	0.004963	0.01751	-0.66	0.11	6.53E-02	-0.68	0.04	3.47E-03	-0.14	0.10	6.48E-01	-0.19	0.11	6.35E-01	-0.69	0.14	2.75E-02	-0.25	0.20	3.94E-01	-0.49	0.10	7.81E-02	-1.06	0.04	3.39E-03
TRIM5	No	No	No	Down	0.70	0.005655	validated	-0.36	0.10	2.79E-01	-0.58	0.04	1.26E-02	-0.42	0.10	1.94E-01	-0.76	0.12	4.30E-02	-0.85	0.15	1.43E-02	-0.23	0.20	3.92E-01	-0.63	0.10	5.41E-02	-0.33	0.03	4.11E-02
CALN1	No	Yes	No	Down	0.70	0.0264	1.5E-19	-0.64	0.11	2.02E-02	-0.35	0.04	3.00E-02	-0.20	0.10	4.25E-01	-0.03	0.11	9.09E-01	-0.97	0.15	4.36E-02	-1.45	0.25	4.49E-02	-0.48	0.10	4.20E-02	-0.02	0.03	9.06E-01
BEX5	No	Yes	No	Down	0.70	6.71E-06	1.5E-19	-0.75	0.11	1.54E-03	-0.65	0.04	3.54E-05	-0.11	0.10	5.70E-01	-0.63	0.12	1.62E-02	-0.38	0.14	9.46E-02	-0.57	0.21	5.77E-02	-0.76	0.10	1.24E-03	-0.28	0.03	4.78E-02
HP	No	Yes	No	Down	0.70	3.58E-06	1.5E-19	-0.53	0.10	4.13E-02	-0.83	0.04	1.36E-06	-0.42	0.10	5.20E-02	-0.75	0.12	2.37E-03	-0.03	0.14	7.95E-01	-0.68	0.21	3.60E-02	-0.63	0.10	6.69E-03	-0.27	0.03	4.73E-02
DNALI1	No	No	No	Down	0.70	4.80E-05	validated	-0.05	0.10	8.18E-01	-0.30	0.04	2.90E-02	-0.55	0.11	3.08E-02	-0.76	0.12	3.31E-03	-1.00	0.15	3.13E-03	-0.01	0.20	9.64E-01	-1.14	0.10	5.91E-05	-0.32	0.03	3.03E-02
PTGS2	No	Yes	No	Down	0.70	0.000287	1.5E-19	-0.45	0.10	5.80E-02	-0.22	0.04	1.19E-01	-0.50	0.10	2.24E-02	-0.36	0.11	1.60E-01	-0.57	0.14	2.95E-02	-0.97	0.22	6.06E-03	-0.90	0.10	3.43E-04	-0.15	0.03	2.90E-01
TMED2	No	No	No	Down	0.70	0.003047	validated	-0.63	0.11	6.11E-02	-0.37	0.04	3.95E-02	-0.44	0.10	1.28E-01	-0.65	0.12	1.63E-01	-0.73	0.14	1.03E-02	-0.55	0.21	7.27E-02	-0.16	0.10	5.17E-01	-0.59	0.03	4.39E-03
ADD3	No	No	No	Down	0.70	0.000748	validated	-0.35	0.10	1.90E-01	-0.38	0.04	2.51E-02	-0.81	0.11	8.39E-03	-0.03	0.11	8.99E-01	-0.70	0.14	1.54E-02	-0.03	0.20	8.57E-01	-1.22	0.10	4.90E-04	-0.58	0.03	1.41E-02
P2RY1	No	Yes	No	Down	0.70	0.035507	0.00381	-0.85	0.11	9.77E-03	-0.61	0.04	3.12E-03	-0.24	0.10	4.98E-01	-0.06	0.11	8.40E-01	-1.14	0.16	3.36E-01	-0.13	0.20	9.01E-01	-0.81	0.10	7.78E-02	-0.28	0.03	1.30E-01
GCHFR	No	Yes	No	Down	0.70	0.000159	1.5E-19	-0.27	0.10	3.90E-01	-0.81	0.04	5.18E-05	-0.41	0.10	1.31E-01	-0.18	0.11	5.18E-01	-0.39	0.14	1.07E-01	-0.80	0.22	2.26E-02	-0.42	0.10	1.78E-01	-0.83	0.03	2.29E-04
ZNF589	No	Yes	No	Down	0.70	0.010124	0.01352	-0.38	0.10	2.29E-01	-0.53	0.04	5.46E-03	-0.22	0.10	4.46E-01	-1.11	0.13	4.42E-03	-0.30	0.14	2.73E-01	-1.01	0.23	4.13E-02	-0.27	0.10	2.97E-01	-0.28	0.03	1.82E-01
C22ORF15	No	Yes	No	Down	0.70	0.000477	4.3E-05	-0.78	0.11	3.50E-03	-0.73	0.04	3.91E-05	-0.70	0.11	7.82E-02	-0.68	0.12	2.28E-02	-0.54	0.14	6.23E-02	-0.20	0.20	5.84E-01	-0.28	0.10	4.59E-01	-0.16	0.03	2.78E-01
ZNF264	No	No	No	Down	0.70	0.011041	validated	-0.58	0.10	1.05E-01	-0.63	0.04	7.01E-03	-0.46	0.10	1.32E-01	-0.62	0.12	1.11E-01	-0.52	0.14	2.44E-01	-0.16	0.20	6.36E-01	-1.10	0.10	1.20E-03	-0.01	0.03	9.37E-01
PEMT	No	Yes	No	Down	0.70	0.000784	0.00117	-0.39	0.10	2.96E-01	-0.59	0.04	2.99E-03	-0.17	0.10	5.80E-01	-0.02	0.11	9.63E-01	-1.15	0.16	7.66E-04	-0.47	0.21	1.19E-01	-0.33	0.10	2.87E-01	-0.97	0.03	2.91E-04

Supplementary Table 2. Details for 2,053 genes differentially expressed between EEC and NEEC, identified by microarray meta-analysis.

EEC - endometrioid endometrial cancer, NEEC - non-endometrioid endometrial cancer, SMD - Standardised Mean Difference, FDR - False Discovery Rate, Var - Variance

Gene	145 gene list	1253 gene list	Associated with EEC-specific survival?	Up/Down Regulated in NEEC	average standardised fold change	Microarray Meta FDR	RNASeq FDR	Individual microarray study results																							
								TCGA			E-MTAB-2532			E-GEOD-56026			GSE32507			GSE24537			E-GEOD-23518			E-GEOD-17025			E-GEOD-2109		
								SMD	Var	P-value	SMD	Var	P-value	SMD	Var	P-value	SMD	Var	P-value	SMD	Var	P-value	SMD	Var	P-value	SMD	Var	P-value	SMD	Var	P-value
CCDC53	No	Yes	No	Down	0.70	0.014014	0.04104	-0.95	0.11	1.45E-02	-0.42	0.04	7.79E-02	-0.52	0.11	1.23E-01	-0.16	0.11	6.91E-01	-0.30	0.14	2.03E-01	-1.05	0.23	6.75E-03	-0.13	0.10	6.35E-01	-0.55	0.03	4.09E-02
ATXN7L1	No	Yes	No	Down	0.70	0.007501	0.0002	-0.24	0.10	5.55E-01	-0.50	0.04	1.18E-02	-1.32	0.12	1.87E-03	-0.74	0.12	5.48E-02	-0.34	0.14	7.80E-01	-0.10	0.20	9.41E-01	-0.41	0.10	1.55E-01	-0.42	0.03	9.34E-03
FGF3	No	Yes	No	Down	0.70	0.003909	0.00133	-0.85	0.11	2.44E-03	-0.10	0.04	5.72E-01	-0.25	0.10	2.89E-01	-1.01	0.13	1.56E-02	-0.56	0.14	1.43E-01	-0.54	0.21	8.99E-02	-0.46	0.10	5.44E-02	-0.30	0.03	3.49E-02
TAX1BP1	No	No	No	Down	0.70	0.005033	validated	-0.75	0.11	3.45E-02	-0.23	0.04	2.83E-01	-0.68	0.11	5.24E-02	-0.23	0.11	6.55E-01	-0.64	0.14	2.25E-02	-0.25	0.20	3.02E-01	-0.27	0.10	2.65E-01	-1.01	0.04	4.59E-04
RNF135	No	Yes	No	Down	0.70	0.010474	0.02336	-0.58	0.10	1.00E-01	-0.44	0.04	5.51E-02	-0.03	0.10	9.05E-01	-0.07	0.11	8.68E-01	-0.04	0.14	8.27E-01	-0.85	0.22	5.83E-02	-0.67	0.10	4.98E-02	-1.38	0.04	1.57E-04
RPL22L1	No	Yes	No	Down	0.70	0.007038	0.00347	-0.66	0.11	2.33E-02	-0.53	0.04	5.03E-03	-0.47	0.10	8.51E-02	-0.43	0.12	1.80E-01	-0.77	0.15	1.47E-01	-0.48	0.21	3.05E-01	-0.27	0.10	2.77E-01	-0.45	0.03	2.70E-02
KLRB1	No	Yes	No	Down	0.70	0.000167	0.00144	-0.53	0.10	3.78E-02	-0.83	0.04	2.70E-05	-0.74	0.11	1.40E-02	-0.53	0.12	7.36E-02	-0.20	0.14	4.22E-01	-0.50	0.21	2.55E-01	-0.21	0.10	4.82E-01	-0.52	0.03	2.80E-03
PITPNC1	No	Yes	No	Down	0.70	0.000639	0.00028	-0.70	0.11	1.41E-02	-0.59	0.04	2.23E-03	-0.93	0.11	2.61E-03	-0.02	0.11	9.50E-01	-0.21	0.14	3.49E-01	-0.52	0.21	1.17E-01	-1.05	0.10	8.09E-04	-0.02	0.03	8.80E-01
ZDHHC6	No	No	No	Down	0.70	0.041742	validated	-0.07	0.10	8.53E-01	-0.57	0.04	2.34E-02	-0.43	0.10	2.48E-01	-1.34	0.14	3.18E-02	-0.77	0.15	1.33E-02	-0.30	0.20	2.72E-01	-0.06	0.10	8.42E-01	-0.51	0.03	1.26E-01
IFT88	No	Yes	No	Down	0.70	0.024645	0.01373	-0.48	0.10	1.12E-01	-0.46	0.04	3.03E-02	-0.26	0.10	4.08E-01	-0.47	0.12	2.68E-01	-0.49	0.14	2.11E-01	-0.46	0.21	3.14E-01	-0.78	0.10	2.04E-02	-0.65	0.03	2.91E-02
DLEC1	No	Yes	No	Down	0.70	1.14E-05	0.00046	-0.34	0.10	1.70E-01	-0.61	0.04	1.44E-04	-0.37	0.10	2.15E-01	-0.73	0.12	6.03E-03	-0.43	0.14	1.30E-01	-0.27	0.20	6.07E-01	-0.60	0.10	3.68E-02	-0.69	0.03	2.02E-05
PLXNB1	No	No	No	Down	0.71	0.016015	validated	-0.09	0.10	7.43E-01	-0.22	0.04	1.88E-01	-0.06	0.10	8.64E-01	-1.24	0.13	2.85E-03	-0.30	0.14	1.69E-01	-1.57	0.26	1.54E-03	-0.55	0.10	6.06E-02	-0.02	0.03	8.87E-01
SUMF1	No	No	No	Down	0.71	0.005051	validated	-0.05	0.10	8.54E-01	-0.42	0.04	3.74E-02	-0.15	0.10	6.30E-01	-0.83	0.12	4.44E-02	-0.92	0.15	5.12E-03	-0.79	0.22	2.61E-02	-0.02	0.10	9.48E-01	-0.85	0.03	2.48E-03
SAA4	No	No	No	Down	0.71	0.001049	validated	-0.34	0.10	1.34E-01	-0.69	0.04	8.66E-05	-0.41	0.10	1.11E-01	-0.57	0.12	2.18E-02	-0.53	0.14	1.21E-01	-0.96	0.22	4.18E-02	-0.38	0.10	1.36E-01	-0.15	0.03	3.92E-01
TOM1	No	Yes	No	Down	0.71	0.014097	0.00015	-0.32	0.10	3.31E-01	-0.69	0.04	5.74E-03	-0.35	0.10	3.13E-01	-0.21	0.11	5.89E-01	-0.68	0.14	2.02E-02	-0.58	0.21	1.01E-01	-0.74	0.10	6.56E-02	-0.45	0.03	7.31E-02
TCF7	No	Yes	No	Down	0.71	0.0264	7.8E-05	-0.83	0.11	3.27E-03	-0.46	0.04	8.91E-03	-0.11	0.10	7.00E-01	-0.20	0.11	5.01E-01	-1.19	0.16	4.06E-01	-0.41	0.20	5.84E-01	-0.54	0.10	8.21E-02	-0.29	0.03	8.80E-02
CXCL3	No	Yes	No	Down	0.71	6.66E-05	0.00014	-0.93	0.11	7.26E-04	-0.61	0.04	3.05E-04	-0.64	0.11	8.49E-03	-0.76	0.12	5.04E-03	-0.13	0.14	7.07E-01	-0.34	0.20	4.63E-01	-0.29	0.10	1.97E-01	-0.32	0.03	4.41E-02
PTER	No	No	No	Down	0.71	0.018215	validated	-0.39	0.10	1.56E-01	-0.16	0.04	3.84E-01	-0.58	0.11	3.80E-02	-0.91	0.12	1.50E-02	-0.39	0.14	3.06E-01	-0.94	0.22	1.16E-01	-0.05	0.10	8.36E-01	-0.59	0.03	6.63E-03
SLC27A5	No	Yes	No	Down	0.71	0.013872	0.02162	-1.22	0.12	1.18E-03	-0.44	0.04	1.35E-02	-0.25	0.10	4.61E-01	-0.51	0.12	1.54E-01	-0.57	0.14	2.54E-01	-0.32	0.20	3.71E-01	-0.52	0.10	1.28E-01	-0.18	0.03	2.45E-01
GNPAT	No	Yes	No	Down	0.71	0.021683	0.04162	-0.59	0.10	9.86E-02	-0.65	0.04	7.33E-03	-0.31	0.10	3.94E-01	-0.23	0.11	5.92E-01	-0.30	0.14	2.17E-01	-0.66	0.21	5.15E-02	-1.08	0.10	1.07E-02	-0.18	0.03	5.17E-01
TPH1	No	Yes	No	Down	0.71	0.013492	0.0356	-0.67	0.11	1.79E-02	-0.38	0.04	1.63E-01	-0.61	0.11	7.26E-03	-0.49	0.12	1.26E-01	-1.10	0.15	3.50E-01	-0.10	0.20	9.39E-01	-0.45	0.10	4.01E-02	-0.22	0.03	8.77E-02
MFSD4	No	Yes	No	Down	0.71	0.000754	0.03187	-0.07	0.10	8.09E-01	-0.87	0.04	1.48E-05	-0.70	0.11	7.13E-03	-0.22	0.11	3.79E-01	-0.71	0.14	4.50E-01	-0.84	0.22	5.94E-01	-0.01	0.10	9.55E-01	-0.58	0.03	4.08E-04
MAP2K2	No	Yes	No	Down	0.71	0.047732	0.01135	-1.13	0.11	1.81E-02	-0.42	0.04	9.97E-02	-0.23	0.10	4.70E-01	-0.47	0.12	3.22E-01	-0.01	0.14	9.42E-01	-0.21	0.20	3.54E-01	-1.23	0.10	8.18E-03	-0.31	0.03	1.19E-01
USP3	No	No	No	Down	0.71	0.016324	validated	-0.28	0.10	3.86E-01	-0.61	0.04	7.24E-03	-0.92	0.11	1.99E-02	-0.06	0.11	8.84E-01	-0.70	0.14	3.44E-02	-0.40	0.20	2.09E-01	-0.66	0.10	6.40E-02	-0.36	0.03	2.21E-01
IL18R1	No	Yes	No	Down	0.71	0.012343	0.00409	-0.43	0.10	1.52E-01	-0.68	0.04	1.40E-03	-0.37	0.10	1.67E-01	-0.42	0.12	1.83E-01	-0.62	0.14	2.27E-01	-0.61	0.21	2.65E-01	-0.74	0.10	1.51E-02	-0.13	0.03	4.22E-01
CYB5D1	No	Yes	No	Down	0.71	0.007095	0.00163	-0.27	0.10	3.80E-01	-0.31	0.04	2.05E-01	-0.77	0.11	2.12E-02	-0.08	0.11	8.25E-01	-0.70	0.14	3.34E-02	-0.32	0.20	2.88E-01	-0.69	0.10	2.32E-02	-0.85	0.03	2.03E-03
ALAD	No	Yes	No	Down	0.71	0.033949	0.00149	-0.48	0.10	2.04E-01	-0.46	0.04	1.69E-02	-0.58	0.11	9.22E-02	-0.28	0.11	4.40E-01	-0.45	0.14	1.48E-01	-1.23	0.24	1.06E-02	-0.43	0.10	2.22E-01	-0.08	0.03	7.09E-01
GADD45G	No	Yes	No	Down	0.71	0.002726	6.4E-05	-0.80	0.11	1.13E-02	-0.55	0.04	2.63E-03	-0.47	0.10	1.12E-01	-0.09	0.11	7.57E-01	-0.42	0.14	1.28E-01	-0.70	0.21	6.31E-02	-0.59	0.10	4.56E-02	-0.36	0.03	6.93E-02
SSR3	No	No	No	Down	0.71	0.030443	validated	-0.41	0.10	2.81E-01	-0.34	0.04	7.61E-02	-0.54	0.11	8.03E-02	-0.13	0.11	7.39E-01	-1.16	0.16	1.51E-01	-0.08	0.20	8.82E-01	-0.82	0.10	4.46E-03	-0.51	0.03	3.41E-02
ERO1L	No	No	No	Down	0.71	0.001923	validated	-0.64	0.11	2.35E-02	-0.33	0.04	3.37E-02	-0.15	0.10	5.05E-01	-0.38	0.11	2.02E-01	-0.48	0.14	6.97E-02	-1.21	0.24	2.42E-03	-0.31	0.10	1.72E-01	-0.48	0.03	1.17E-02
ANXA3	No	No	No	Down	0.71	0.000559	validated	-0.06	0.10	8.04E-01	-0.69	0.04	1.83E-04	-0.03	0.10	8.91E-01	-0.32	0.11	2.25E-01	-1.09	0.15	9.88E-02	-0.27	0.20	6.10E-01	-0.75	0.10	6.43E-03	-0.75	0.03	1.41E-04
PGRMC2	No	No	No	Down	0.71	0.005905	validated	-0.13	0.10	7.00E-01	-0.34	0.04	7.31E-02	-0.61	0.11	8.43E-02	-0.42	0.12	3.66E-01	-0.31	0.14	1.66E-01	-0.89	0.22	1.02E-02	-0.37	0.10	1.74E-01	-0.89	0.03	8.60E-04
HOXB6	No	No	No	Down	0.71	0.000613	validated	-0.09	0.10	6.42E-01	-0.24	0.04	8.02E-02	-0.50	0.10	2.65E-02	-0.75	0.12	4.86E-03	-0.72	0.14	2.37E-02	-0.78	0.22	3.52E-02	-0.56	0.10	1.49E-02	-0.32	0.03	2.47E-02
TMEM190	No	No	No	Down	0.71	0.002352	validated	-0.32	0.10	1.22E-01	-0.40	0.04	4.31E-03	-0.56	0.11	4.80E-02	-0.54	0.12	4.21E-02	-0.66	0.14	3.90E-02	-0.85	0.22	1.25E-01	-0.41	0.10	7.48E-02	-0.21	0.03	1.14E-01
SPPL2B	No	Yes	No	Down	0.71	0.010774	0.0001	-0.87	0.11	6.35E-03	-0.04	0.04	7.76E-01	-0.82	0.11	2.57E-02	-0.89	0.12	1.96E-02	-0.37	0.14	3.36E-01	-0.35	0.20	5.32E-01	-0.15	0.10	5.93E-01	-0.47	0.03	6.72E-03
DLX6	No	Yes	No	Down	0.71	0.003175	2E-05	-0.57	0.10	2.04E-02	-0.41	0.04	2.62E-02	-0.14	0.10	5.09E-01	-0.51	0.12	6.16E-02	-0.20	0.14	6.04E-01	-0.95	0.22	3.27E-01	-0.91	0.10	6.25E-04	-0.28	0.03	4.56E-02
ATP6V1G1	No	No	No	Down	0.71	0.006351	validated	-0.09	0.10	8.20E-01	-0.44	0.04	4.69E-02	-0.28	0.10	4.31E-01	-0.16	0.11	6.87E-01	-1.46	0.17	6.52E-05	-1.16	0.23	2.06E-03	-0.13	0.10	6.60E-01	-0.23	0.03	4.75E-01
SPRYD3	No	Yes	No	Down	0.71	0.023702	0.01494	-0.24	0.10	5.25E-01	-0.82	0.04	1.42E-03	-0.58	0.11	2.09E-01	-0.48	0.12	3.07E-01	-0.47	0.14	1.15E-01	-0.16	0.20	5.20E-01	-0.46	0.10	3.44E-01	-0.75	0.03	1.33E-02
PCDH19	No	Yes	No	Down	0.71	0.000853	6.7E-06	-0.59	0.10	2.55E-02	-0.07	0.04	6.14E-01	-0.41	0.10	7.32E-02	-0.07	0.11	7.83E-01	-0.91	0.15	3.98E-03	-0.71	0.21	3.27E-02	-1.03	0.10	2.19E-04	-0.18	0.03	1.82E-01
APCDD1	No	No	No	Down	0.71	5.41E-05	validated	-0.98	0.11	2.47E-04	-0.25	0.04	6.44E-02	-0.22	0.10	3.05E-01	-0.24	0.11	3.18E-01	-0.53	0.14	3.58E-02	-0.61	0.21	4.33E-02	-0.51	0.10	2.54E-02	-0.60	0.03	3.07E-04
PPP2R1B	No	Yes	No	Down	0.71	0.020285	0.00058	-0.80	0.11	4.22E-02	-0.65	0.04	1.65E-03	-0.78	0.11	2.15E-02	-0.64	0.12	1.00E-01	-0.04	0.14	8.08E-01	-0.80	0.22	2.11E-01	-0.21	0.10	3.68E-01	-0.01	0.03	9.49E-01
SPPL2A	No	Yes	No	Down	0.71	0.006645	0.02132	-0.01	0.10	9.87E-01	-0.60	0.04	3.71E-03	-1.10	0.11	5.84E-03	-0.41	0.12	3.32E-01	-0.41	0.14	1.20E-01	-0.18	0.20	4.58E-01	-0.41	0.10	1.75E-01	-0.82	0.03	7.73E-03
PTPN6	No	No	No	Down	0.71	0.025572	validated	-0.01	0.10	9.75E-01	-0.30	0.04	1.13E-01	-0.68	0.11	1.06E-01	-1.25	0.13	4.34E-03	-0.10	0.14	5.76E-01	-0.56	0.21	9.76E-02	-0.33	0.10	3.42E-01	-0.69	0.03	1.66E-02
HSPB1	No	No	No	Down	0.71	0.000229	0.00173	-0.18	0.10	4.88E-01	-0.49	0.04	1.07E-02	-0.72	0.11	1.53E-02	-0.11	0.11	7.64E-01	-0.12	0.14	4.25E-01	-0.42	0.20	1.16E-01	-1.12	0.10	1.57E-04	-0.75	0.03	6.47E-04
VPS35	No	No	No	Down	0.71	0.036329	validated	-0.64	0.11	1.57E-01	-0.53	0.04	2.47E-02	-0.24	0.10	4.12E-01	-1.07	0.13	5.62E-02	-0.43	0.14	7.87E-02	-0.31	0.20	2.39E-01	-0.65	0.10	2.97E-02	-0.03	0.03	8.26E-01
MESP1	No	Yes	No	Down	0.71	7.73E-05	1.5E-19	-0.77	0.11	3.19E-03	-0.69	0.04	4.38E-05	-0.29	0.10	2.15E-01	-0.36	0.11	1.52E-01	-0.37	0.14	1.53E-01	-0.46	0.21	1.30E-01	-0.52	0.10	6.32E-02	-0.44	0.03	5.64E-03
SDCBP2	No	Yes	No	Down	0.71	0.003391	0.02044	-0.03	0.10	8.98E-01	-0.67	0.04	2.37E-04	-0.92	0.11	7.20E-03	-0.37	0.11	2.26E-01	-0.63	0.14	1.11E-01	-0.45	0.21	3.35E-01	-0.44	0.10	1.80E-01	-0.38	0.03	4.79E-02
WDR16	No	Yes	No	Down	0.71	0.002448	0.00297	-0.52	0.10	2.56E-02	-0.40	0.04	7.32E-03	-0.36	0.10	1.39E-01	-0.42	0.12	1.22E-01	-0.79	0.15	6.08E-02	-0.53	0.21	3.11E-01	-0.54	0.10	2.46E-02	-0.33	0.03	3.34E-02
BMX	No	Yes	No	Down	0.71	0.004676	1.5E-19	-0.96	0.11	1.08E-03	-0.65	0.04	5.60E-04	-0.50	0.10	8.79E-02	-0.08	0.11	7.39E-01	-0.36	0.14	3.10E-01	-0.82	0.22	5.60E-01	-0.23	0.10	4.95E-01	-0.27	0.03	6.94E-02
TEKT3	No	Yes	No	Down	0.71	0.008939	9.4E-05	-0.18	0.10	4.44E-01	-0.55	0.04	9.05E-03	-0.56	0.11	1.39E-01	-0.69	0.12	2.27E-02	-0.80	0.15	2.75E-01	0.00	0.20	9.98E-01	-0.75	0.10	1.65E-02	-0.34	0.03	1.90E-02
MOSPD2	No	No	No	Down	0.71	0.01306	validated	-0.19	0.10	5.38E-01	-0.68	0.04	2.97E-03	-0.66	0.11	5.75E-02	-0.57	0.12	1.04E-01	-0.06	0.14	7.50E-01	-0.38	0.20	2.46E-01	-1.28	0.10	1.88E-03	-0.05	0.03	8.56E-01
DENND1B	No	No	No	Down	0.72	0.002283	validated	-0.22	0.10	4.24E-01	-0.50	0.04	1.31E-02	-0.49	0.10	7.81E-02	-0.61	0.12	8.48E-02	-0.13	0.14	9.15E-01	-0.38	0.20	7.50E-01	-1.52	0.11	1.82E-06	-0.02	0.03	9.16E-01
NDUFA8	No	No	No	Down	0.72	0.015248	validated	-0.14	0.10	7.10E-01	-0.45	0.04	5.14E-02	-0.64	0.11	8.95E-02	-0.21	0.11	6.26E-01	-0.99	0.15	2.09E-03	-0.98	0.22	6.13E-03	-0.11	0.10	6.54E-01	-0.34	0.03	2.47E-01
PIGV	No	No	No	Down	0.72	0.037819	validated	-0.81	0.11	1.84E-02	-0.46	0.04	5.15E-02	-0.50	0.10	1.23E-01	-1.26	0.13	1.14E-02	-0.60	0.14	1.05E-01	-0.06	0.20	7.78E-01	-0.09	0.10	7.46E-01	-0.08	0.03	5.70E-01
DNAI2	No	Yes	No	Down	0.72	0.000334	0.001	-0.47	0.10	3.66E-02	-0.74	0.04	1.79E-05	-0.45	0.10	7.55E-02	-0.30	0.11	2.13E-01	-0.62	0.14	1.29E-01	-0.49	0.21	3.56E-01	-0.53	0.10	2.21E-02	-0.26	0.03	5.35E-02
C17ORF61	No	Yes	No	Down	0.72	0.015296	0.00399	-1.30	0.12	1.25E-03	-0.10	0.04	5.75E-01	-0.46	0.10	1.41E-01	-0.04	0.11	9.31E-01	-0.11	0.14	4.77E-01	-0.61	0.21	5.15E-02	-0.51	0.10	1.57E-01	-0.72	0.03	1.18E-02
AXIN2	No	Yes	No	Down	0.72	0.000599	0.00096	-1.00	0.11	5.83E-04	-0.30	0.04	4.29E-02	-0.42	0.10	8.10E-02	-0.27	0.11	3.06E-01	-0.42	0.14	8.11E-02	-0.50	0.21	7.53E-02	-0.82	0.10	1.23E-03	-0.13	0.03	3.98E-01
C1GALT1C1	No	No	No	Down	0.72	0.022692	validated	-0.13	0.10	6.50E-01	-0.51	0.04	1.29E-02	-0.62	0.11	6.99E-02	-0.02	0.11	9.56E-01	-0.68	0.14	7.66E-02	-1.12	0.23	2.21E-02	-0.60	0.10	2.35E-02	-0.15	0.03	5.24E-01
FBLN1	No	Yes	No	Down	0.72	9.98E-05	1.5E-19	-1.11	0.11	1.93E-03	-0.39	0.04	8.88E-03	0.00	0.10	9.89E-01	-0.06	0.11	8.11E-01	-0.85	0.15	2.36E-03	-0.20	0.20	3.49E-01	-0.80	0.10	1.24E-03	-0.43	0.03	4.14E-03
MCEE	No	Yes	No	Down	0.72	0.015068	0.03108	-0.28	0.10	3.93E-01	-0.90	0.04	8.32E-04	-0.59	0.11	8.00E-02	-0.01	0.11	9.83E-01	-0.50	0.14	1.11E-01	-0.36	0.20	2.43E-01	-0.66	0.10	5.59E-02	-0.53	0.03	1.06E-01
CEL	No	Yes	No	Down	0.72	0.003472	5.4E-05	-0.34	0.10	3.62E-01	-0.04	0.04	8.51E-01	-0.68	0.11	5.79E-03	-0.43	0.12	1.68E-01	-0.91	0.15	6.70E-03	-0.68	0.21	7.58E-02	-0.32	0.10	1.46E-01	-0.41	0.03	5.27E-03
SKAP1	No	No	No	Down	0.72	0.003321	validated	-0.17	0.10	4.64E-01	-0.26	0.04	8.33E-02	-0.48	0.10	6.12E-02	-1.08	0.13	6.43E-04	-0.71	0.14	1.96E-02	-0.35	0.20	2.62E-01	-0.49	0.10	1.02E-01	-0.29	0.03	1.34E-01
ECHS1	No	No	No	Down	0.72	0.017805	validated	-0.47	0.10	1.99E-01	-0.55	0.04	1.31E-02	-0.51	0.11	1.45E-01	-0.95	0.13	2.06E-02	-0.18	0.14	3.21E-01	-0.14	0.20	4.73E-01	-0.66	0.10	3.23E-02	-0.36	0.03	1.64E-01
LRRC4	No	Yes	No	Down	0.72	0.001662	0.00769	-0.58	0.10	2.14E-02	-0.51	0.04	4.55E-03	-0.63	0.11	4.14E-02	-0.32	0.11	2.56E-01	-0.36	0.14	6.05E-01	-0.49	0.21	5.35E-01	-0.24	0.10	5.41E-01	-0.69	0.03	1.14E-04
LPPR4	No	No	No	Down	0.72	0.002111	validated	-1.02	0.11	2.54E-04	-0.26	0.04	1.12E-01	-0.76	0.11	4.94E-03	-0.63	0.12	3.66E-02	-0.35	0.14	3.46E-01	-0.17	0.20	7.53E-01	-0.35	0.10	1.43E-01	-0.27	0.03	1.80E-01
SMS	No	No	No	Down	0.72	0.008539	validated	-0.95	0.11	8.77E-03	-0.01	0.04	9.54E-01	-0.53	0.11	8.05E-02	-0.04	0.11	8.98E-01	-0.71	0.14	8.99E-03	-0.89	0.22	8.59E-03	-0.45	0.10	6.94E-02	-0.22	0.03	2.99E-01
VPS13C	No	No	No	Down	0.72	0.005604	0.02525	-0.78	0.11	1.33E-02	-1.03	0.04	2.84E-05	-0.30	0.10	2.65E-01	-0.34	0.11	3.30E-01	-0.27	0.14	3.27E-01	-0.86	0.22	1.30E-01	-0.03	0.10	8.80E-01	-0.19	0.03	2.88E-01
CAPN13	No	No	No	Down	0.72	5.41E-05	validated	-0.54	0.10	1.62E-02	-0.54	0.04	2.28E-04	-0.10	0.10	6.12E-01	-0.65	0.12	1.27E-02	-0.20	0.14	2.79E-01	-0.93	0.22	6.85E-03	-0.43	0.10	3.94E-02	-0.40	0.03	8.38E-03
PIP	No	Yes	No	Down	0.72	0.00222	8.9E-06	-0.57	0.10	1.44E-02	-0.57	0.04	1.14E-03	-0.23	0.10	4.10E-01	-0.44	0.12	5.91E-02	-0.71	0.14	1.10E-01	-0.73	0.21	6.58E-02	-0.24	0.10	2.94E-01	-0.31	0.03	4.60E-02

Supplementary Table 2. Details for 2,053 genes differentially expressed between EEC and NEEC, identified by microarray meta-analysis.

EEC - endometrioid endometrial cancer, NEEC - non-endometrioid endometrial cancer, SMD - Standardised Mean Difference, FDR - False Discovery Rate, Var - Variance

Gene	145 gene list	1253 gene list	Associated with EEC-specific survival?	Up/Down Regulated in NEEC	average standardised fold change	Microarray Meta FDR	RNASeq FDR	Individual microarray study results																							
								TCGA			E-MTAB-2532			E-GEOD-56026			GSE32507			GSE24537			E-GEOD-23518			E-GEOD-17025			E-GEOD-2109		
								SMD	Var	P-value	SMD	Var	P-value	SMD	Var	P-value	SMD	Var	P-value	SMD	Var	P-value	SMD	Var	P-value	SMD	Var	P-value	SMD	Var	P-value
AP3B1	No	No	No	Down	0.72	0.030683	validated	-0.37	0.10	3.50E-01	-0.46	0.04	4.19E-02	-0.71	0.11	5.53E-02	-0.11	0.11	7.92E-01	-0.52	0.14	5.65E-02	-0.88	0.22	1.75E-02	-0.44	0.10	1.38E-01	-0.30	0.03	2.99E-01
HYDIN	No	No	No	Down	0.72	0.017495	validated	-0.27	0.10	3.64E-01	-0.23	0.04	9.38E-02	-0.37	0.10	1.49E-01	-0.74	0.12	6.22E-03	-1.00	0.15	3.87E-02	-0.54	0.21	3.13E-01	-0.47	0.10	6.29E-02	-0.15	0.03	2.48E-01
HAGH	No	No	No	Down	0.72	0.043013	validated	-0.26	0.10	4.83E-01	-0.26	0.04	1.79E-01	-0.50	0.10	1.85E-01	-0.23	0.11	6.83E-01	-0.25	0.14	2.86E-01	-0.57	0.21	9.11E-02	-0.98	0.10	1.48E-02	-0.71	0.03	1.55E-02
ADAMTS6	No	Yes	No	Down	0.72	0.001976	0.00123	-0.49	0.10	5.88E-02	-0.43	0.04	8.42E-03	-0.81	0.11	2.63E-03	-0.47	0.12	7.22E-02	-0.59	0.14	9.36E-02	-0.42	0.20	2.68E-01	-0.28	0.10	1.66E-01	-0.27	0.03	7.48E-02
CMTM6	No	No	No	Down	0.72	0.005626	validated	-0.70	0.11	2.54E-02	-0.55	0.04	4.78E-03	-0.52	0.11	1.20E-01	-0.35	0.11	2.49E-01	-0.46	0.14	5.78E-02	-0.82	0.22	1.39E-02	-0.33	0.10	1.54E-01	-0.03	0.03	8.06E-01
TIGD2	No	Yes	No	Down	0.72	0.024532	0.0007	-0.72	0.11	2.96E-02	-0.73	0.04	2.21E-03	-0.38	0.10	1.75E-01	-0.07	0.11	8.43E-01	-0.57	0.14	1.12E-01	-0.77	0.21	1.26E-01	-0.25	0.10	3.51E-01	-0.26	0.03	3.01E-01
CLCA4	No	Yes	No	Down	0.72	0.006681	1.5E-19	-0.36	0.10	2.10E-01	-0.61	0.04	3.48E-03	-0.49	0.10	4.49E-02	-0.36	0.11	1.86E-01	-0.39	0.14	1.72E-01	-0.78	0.22	3.85E-01	-0.34	0.10	2.10E-01	-0.41	0.03	6.30E-03
KIAA1199	No	No	No	Down	0.72	0.000429	0.01142	-0.74	0.11	3.51E-03	-0.38	0.04	8.13E-03	-0.19	0.10	3.75E-01	-0.98	0.13	1.03E-03	-0.42	0.14	1.28E-01	-0.25	0.20	3.22E-01	-0.53	0.10	2.04E-02	-0.24	0.03	1.05E-01
OSTALPHA	No	Yes	No	Down	0.72	0.001025	0.01538	-0.49	0.10	4.96E-02	-0.62	0.04	4.82E-04	-0.28	0.10	2.57E-01	-0.16	0.11	5.18E-01	-0.75	0.14	8.50E-02	-0.73	0.21	1.56E-01	-0.13	0.10	6.17E-01	-0.58	0.03	2.77E-04
RAB36	No	No	No	Down	0.72	0.002625	validated	-0.42	0.10	9.76E-02	-0.65	0.04	4.35E-04	-0.31	0.10	2.45E-01	-0.83	0.12	1.42E-02	-0.79	0.15	4.27E-02	-0.03	0.20	9.41E-01	-0.20	0.10	4.36E-01	-0.50	0.03	2.27E-02
DMBT1	No	Yes	No	Down	0.72	1.14E-05	2E-05	-0.76	0.11	2.42E-03	-0.83	0.04	1.36E-06	-0.15	0.10	5.20E-01	-1.02	0.13	2.44E-04	-0.20	0.14	2.94E-01	-0.03	0.20	8.65E-01	-0.37	0.10	9.73E-02	-0.37	0.03	3.06E-02
TMEM141	No	No	No	Down	0.72	0.017303	validated	-0.06	0.10	8.55E-01	-0.63	0.04	4.99E-03	-0.50	0.10	1.52E-01	-0.92	0.12	3.99E-02	-0.42	0.14	1.38E-01	-0.45	0.21	1.59E-01	-0.29	0.10	3.77E-01	-0.47	0.03	2.75E-02
SGMS2	No	No	No	Down	0.72	0.009108	validated	-0.14	0.10	6.36E-01	-0.59	0.04	1.08E-02	-0.62	0.11	2.09E-02	-0.60	0.12	6.09E-02	-0.44	0.14	4.63E-01	-0.57	0.21	3.44E-01	-0.04	0.10	8.54E-01	-0.72	0.03	9.60E-04
SLC26A9	No	No	No	Down	0.72	0.006163	validated	-0.38	0.10	1.16E-01	-0.36	0.04	2.48E-02	-0.72	0.11	1.58E-02	-0.95	0.13	1.04E-03	-0.52	0.14	4.33E-01	-0.11	0.20	8.32E-01	-0.49	0.10	1.20E-01	-0.18	0.03	2.18E-01
PLA1A	No	No	No	Down	0.72	0.000974	validated	-0.63	0.10	1.44E-02	-0.26	0.04	7.73E-02	-0.73	0.11	3.85E-03	-0.53	0.12	4.40E-02	-0.29	0.14	2.62E-01	-0.43	0.20	1.66E-01	-0.42	0.10	8.82E-02	-0.44	0.03	8.99E-03
CLDN10	No	No	No	Down	0.73	0.000404	validated	-0.45	0.10	4.28E-02	-0.28	0.04	3.02E-02	-0.24	0.10	2.27E-01	-0.42	0.12	7.66E-02	-0.90	0.15	2.56E-03	-0.89	0.22	1.03E-02	-0.07	0.10	6.89E-01	-0.46	0.03	2.09E-03
SLC23A1	No	Yes	No	Down	0.73	0.000227	0.0044	-0.42	0.10	8.23E-02	-0.81	0.04	1.34E-04	-0.70	0.11	9.83E-03	-0.18	0.11	4.51E-01	-0.29	0.14	4.19E-01	-0.31	0.20	7.32E-01	-0.35	0.10	1.54E-01	-0.66	0.03	1.29E-04
SLC39A11	No	No	No	Down	0.73	0.015565	validated	-0.14	0.10	6.28E-01	-0.47	0.04	1.58E-02	-0.67	0.11	3.34E-02	-0.04	0.11	9.07E-01	-0.69	0.14	3.32E-02	-1.03	0.23	1.01E-02	-0.33	0.10	2.24E-01	-0.32	0.03	1.98E-01
IFNGR1	No	No	No	Down	0.73	0.002726	validated	-0.41	0.10	2.26E-01	-0.29	0.04	1.87E-01	-0.27	0.10	3.61E-01	-0.14	0.11	7.67E-01	-0.24	0.14	2.43E-01	-0.57	0.21	6.40E-02	-1.39	0.11	6.36E-06	-0.39	0.03	5.55E-02
MCAT	No	No	No	Down	0.73	0.025417	validated	-0.84	0.11	4.92E-02	-0.19	0.04	1.48E-01	-0.21	0.10	5.26E-01	-0.28	0.11	2.88E-01	-0.64	0.14	7.04E-02	0.00	0.20	9.79E-01	-0.87	0.10	8.22E-03	-0.65	0.03	2.55E-02
SLC24A1	No	No	No	Down	0.73	0.025215	validated	-0.06	0.10	8.33E-01	-0.84	0.04	7.60E-04	-0.63	0.11	6.32E-02	-0.74	0.12	4.81E-02	-0.77	0.15	2.80E-01	-0.08	0.20	8.82E-01	-0.29	0.10	3.36E-01	-0.27	0.03	9.70E-02
UGT8	No	No	No	Down	0.73	0.002661	validated	-0.12	0.10	6.31E-01	-0.45	0.04	1.11E-02	-0.39	0.10	6.71E-02	-0.49	0.12	6.69E-02	-0.59	0.14	1.13E-01	-0.61	0.21	1.96E-01	-0.60	0.10	1.53E-02	-0.42	0.03	5.71E-03
SPCS2	No	No	No	Down	0.73	0.029114	validated	-0.58	0.10	1.27E-01	-0.22	0.04	2.82E-01	-0.36	0.10	2.71E-01	-0.52	0.12	1.53E-01	-0.90	0.15	2.76E-03	-0.70	0.21	3.95E-02	-0.33	0.10	1.65E-01	-0.04	0.03	8.52E-01
IQGAP2	No	Yes	No	Down	0.73	0.001925	0.00573	-0.42	0.10	9.02E-02	-0.56	0.04	2.01E-03	-0.17	0.10	4.66E-01	-0.31	0.11	2.96E-01	-0.56	0.14	8.73E-02	-0.36	0.20	2.45E-01	-0.66	0.10	1.08E-02	-0.59	0.03	4.76E-03
GFRA3	No	Yes	No	Down	0.73	0.009161	7.1E-05	-0.52	0.10	7.11E-02	-0.13	0.04	3.42E-01	-0.39	0.10	8.94E-02	-0.56	0.12	5.45E-02	-0.71	0.14	1.34E-02	-0.75	0.21	2.68E-02	-0.48	0.10	4.56E-02	-0.07	0.03	6.00E-01
CYB561D2	No	Yes	No	Down	0.73	0.042575	0.01547	-0.54	0.10	1.35E-01	-0.45	0.04	3.61E-02	-0.87	0.11	2.81E-02	-0.22	0.11	5.56E-01	-0.59	0.14	7.47E-02	-0.64	0.21	9.16E-02	-0.07	0.10	8.30E-01	-0.23	0.03	1.48E-01
CTSO	No	No	No	Down	0.73	0.012478	validated	-0.36	0.10	1.83E-01	-0.55	0.04	1.01E-02	-0.26	0.10	3.24E-01	-0.23	0.11	4.45E-01	-0.66	0.14	6.29E-02	-0.58	0.21	2.15E-01	-0.09	0.10	6.83E-01	-0.86	0.03	1.04E-03
SPINK5	No	Yes	No	Down	0.73	0.002774	0.00047	-0.77	0.11	1.58E-03	-0.38	0.04	7.49E-03	-0.34	0.10	1.63E-01	-0.18	0.11	4.51E-01	-0.55	0.14	2.24E-01	-0.64	0.21	2.52E-01	-0.32	0.10	1.45E-01	-0.42	0.03	9.40E-03
CD96	No	Yes	No	Down	0.73	0.022942	0.00946	-0.59	0.10	3.01E-02	-0.27	0.04	1.02E-01	-0.75	0.11	1.58E-02	-0.68	0.12	2.02E-02	-0.27	0.14	4.86E-01	-0.43	0.20	4.54E-01	-0.40	0.10	2.54E-01	-0.19	0.03	2.19E-01
TFPI2	No	Yes	No	Down	0.73	0.000307	0.00237	-0.14	0.10	5.08E-01	-0.72	0.04	2.59E-05	-0.46	0.10	3.68E-02	-0.57	0.12	3.21E-02	-0.32	0.14	2.93E-01	-0.56	0.21	2.17E-01	-0.44	0.10	4.27E-02	-0.37	0.03	1.15E-02
TMEM123	No	No	No	Down	0.73	0.007002	validated	-0.51	0.10	2.08E-01	-0.41	0.04	3.18E-02	-0.09	0.10	8.10E-01	-0.13	0.11	7.44E-01	-0.86	0.15	3.07E-03	-0.30	0.20	2.25E-01	-0.57	0.10	2.69E-02	-0.71	0.03	8.08E-03
CXCL2	No	Yes	No	Down	0.73	0.000125	0.00173	-0.93	0.11	4.15E-04	-0.58	0.04	2.34E-04	-0.30	0.10	1.49E-01	-0.28	0.11	2.26E-01	-0.23	0.14	3.89E-01	-0.21	0.20	4.60E-01	-0.90	0.10	4.26E-04	-0.12	0.03	3.50E-01
KISS1R	No	No	No	Down	0.73	0.015255	validated	-0.57	0.10	5.59E-02	-0.07	0.04	6.65E-01	-0.39	0.10	7.46E-02	-0.80	0.12	4.62E-02	-0.46	0.14	1.62E-01	-0.79	0.22	3.50E-02	-0.07	0.10	7.42E-01	-0.42	0.03	7.88E-03
RNF39	No	No	No	Down	0.74	0.042046	validated	-0.41	0.10	1.71E-01	-0.51	0.04	1.69E-02	-0.33	0.10	2.74E-01	-0.15	0.11	5.95E-01	-1.00	0.15	6.08E-02	-0.17	0.20	7.69E-01	-0.59	0.10	9.07E-02	-0.40	0.03	3.08E-02
GRM8	No	Yes	No	Down	0.74	0.011227	0.04188	-0.97	0.11	4.11E-04	0.00	0.04	9.85E-01	-0.55	0.11	3.57E-02	-0.55	0.12	3.64E-02	-0.74	0.14	3.27E-01	-0.45	0.21	1.25E-01	-0.06	0.10	8.53E-01	-0.23	0.03	1.08E-01
ITIH2	No	Yes	No	Down	0.74	0.005508	0.00024	-0.96	0.11	1.64E-03	-0.32	0.04	7.81E-02	-0.68	0.11	1.22E-02	-0.82	0.12	5.00E-03	-0.35	0.14	2.66E-01	-0.19	0.20	7.66E-01	-0.06	0.10	7.90E-01	-0.17	0.03	2.69E-01
RAMP3	No	Yes	No	Down	0.74	0.002525	1.7E-05	-0.93	0.11	2.26E-03	-0.66	0.04	1.31E-03	-0.73	0.11	9.42E-03	-0.22	0.11	4.26E-01	-0.06	0.14	6.61E-01	-0.13	0.20	5.18E-01	-0.60	0.10	4.92E-02	-0.19	0.03	2.68E-01
OR51E1	No	Yes	No	Down	0.74	0.016392	1.5E-19	-0.83	0.11	1.19E-02	-0.63	0.04	1.69E-02	-1.22	0.11	4.46E-04	-0.11	0.11	6.95E-01	-0.08	0.14	9.09E-01	-0.16	0.20	8.59E-01	-0.23	0.10	3.96E-01	-0.25	0.03	2.64E-01
IVNS1ABP	No	No	No	Down	0.74	0.011472	validated	-0.45	0.10	1.35E-01	-0.61	0.04	2.30E-03	-0.47	0.10	9.03E-02	-0.52	0.12	1.75E-01	-0.38	0.14	1.14E-01	-0.83	0.22	1.48E-02	-0.09	0.10	6.84E-01	-0.15	0.03	3.67E-01
IQCD	No	No	No	Down	0.74	0.038612	validated	-0.40	0.10	1.43E-01	-0.28	0.04	6.76E-02	-0.84	0.11	3.00E-02	-0.80	0.12	2.48E-02	-0.28	0.14	4.22E-01	-0.36	0.20	4.26E-01	-0.26	0.10	3.22E-01	-0.28	0.03	1.17E-01
HAMP	No	No	No	Down	0.74	0.019113	validated	-0.70	0.11	1.82E-02	-0.49	0.04	5.83E-03	-0.51	0.11	7.92E-02	-0.50	0.12	1.20E-01	-0.27	0.14	2.99E-01	-0.75	0.21	9.81E-02	-0.07	0.10	8.43E-01	-0.22	0.03	3.03E-01
SDC1	No	No	No	Down	0.74	0.008939	0.00904	-0.76	0.11	1.13E-02	-0.05	0.04	7.01E-01	-0.10	0.10	6.85E-01	-0.14	0.11	6.45E-01	-0.87	0.15	5.84E-03	-0.55	0.21	9.02E-02	-0.61	0.10	2.07E-02	-0.42	0.03	2.84E-02
EDAR	No	No	No	Down	0.74	0.033627	validated	-0.51	0.10	3.47E-02	-0.26	0.04	1.30E-01	-0.41	0.10	1.24E-01	-0.72	0.12	1.54E-02	-0.63	0.14	1.68E-01	-0.58	0.21	1.95E-01	-0.24	0.10	2.80E-01	-0.15	0.03	4.24E-01
SLC1A2	No	Yes	No	Down	0.74	0.014182	1.5E-19	-0.89	0.11	1.95E-02	-0.72	0.04	9.38E-04	-0.43	0.10	6.78E-02	-0.18	0.11	4.64E-01	-0.23	0.14	7.95E-01	-0.28	0.20	8.47E-01	-0.73	0.10	9.93E-03	-0.01	0.03	9.12E-01
C1QTNF6	No	No	No	Down	0.74	0.013297	0.01726	-0.53	0.10	5.76E-02	-0.25	0.04	1.12E-01	-0.64	0.11	5.85E-02	-0.61	0.12	5.47E-02	-0.48	0.14	1.05E-01	-0.24	0.20	3.83E-01	-0.35	0.10	2.88E-01	-0.39	0.03	1.26E-02
SLC6A20	No	No	No	Down	0.74	0.002826	validated	-0.25	0.10	3.36E-01	-0.72	0.04	4.43E-05	-0.72	0.11	6.83E-03	-0.37	0.11	1.54E-01	-0.52	0.14	4.10E-01	-0.48	0.21	4.46E-01	-0.15	0.10	4.61E-01	-0.29	0.03	5.26E-02
NFX1	No	No	No	Down	0.74	0.038392	validated	-0.17	0.10	7.06E-01	-0.83	0.04	1.25E-03	-0.13	0.10	6.75E-01	-1.14	0.13	2.17E-02	-0.46	0.14	1.75E-01	-0.27	0.20	4.14E-01	-0.04	0.10	9.00E-01	-0.43	0.03	5.71E-02
SLC44A4	No	No	No	Down	0.74	1.66E-05	validated	-0.14	0.10	5.29E-01	-0.61	0.04	2.36E-04	-0.66	0.11	8.80E-03	-0.83	0.12	3.73E-03	-0.08	0.14	5.36E-01	-0.02	0.20	8.82E-01	-0.44	0.10	8.23E-02	-0.70	0.03	1.48E-05
OBFC1	No	No	No	Down	0.74	0.049509	validated	-0.02	0.10	9.47E-01	-0.30	0.04	1.27E-01	-0.45	0.10	1.91E-01	-0.45	0.12	2.29E-01	-0.48	0.14	1.34E-01	-0.38	0.20	2.59E-01	-1.17	0.10	2.12E-03	-0.23	0.03	2.54E-01
CYP3A7	No	No	No	Down	0.74	0.016995	validated	-0.77	0.11	7.46E-03	-0.31	0.04	2.94E-02	-0.68	0.11	5.11E-02	-0.61	0.12	1.56E-02	-0.15	0.14	8.73E-01	-0.40	0.20	4.87E-01	-0.35	0.10	4.01E-01	-0.19	0.03	1.87E-01
L3MBTL3	No	No	No	Down	0.74	0.011507	validated	-1.03	0.11	1.16E-03	-0.60	0.04	4.05E-03	-0.36	0.10	1.99E-01	-0.08	0.11	7.65E-01	-0.47	0.14	1.76E-01	-0.41	0.20	2.66E-01	-0.44	0.10	8.20E-02	-0.07	0.03	7.62E-01
SDAD1	No	No	No	Down	0.74	0.047345	validated	-0.62	0.10	1.08E-01	-0.16	0.04	4.06E-01	-0.61	0.11	7.17E-02	-0.39	0.12	2.49E-01	-0.50	0.14	5.91E-02	-0.72	0.21	2.95E-02	-0.16	0.10	5.42E-01	-0.31	0.03	1.17E-01
PVRL4	No	No	No	Down	0.74	0.014296	validated	-0.15	0.10	5.51E-01	-0.65	0.04	4.90E-04	-0.15	0.10	5.44E-01	-0.93	0.12	1.75E-03	-0.59	0.14	3.38E-01	-0.61	0.21	2.65E-01	-0.29	0.10	2.77E-01	-0.09	0.03	5.61E-01
AP1G2	No	No	No	Down	0.74	0.046519	validated	-0.55	0.10	1.24E-01	-0.03	0.04	8.84E-01	-0.01	0.10	9.89E-01	-0.91	0.12	2.10E-02	-0.60	0.14	7.39E-02	-0.59	0.21	1.73E-01	-0.08	0.10	7.49E-01	-0.69	0.03	3.78E-03
RUNDC3B	No	Yes	No	Down	0.74	0.000907	0.00039	-0.34	0.10	1.70E-01	-0.75	0.04	2.37E-04	-0.46	0.10	4.37E-02	-0.23	0.11	3.71E-01	-0.31	0.14	2.35E-01	-0.32	0.20	2.63E-01	-0.53	0.10	2.68E-02	-0.50	0.03	4.86E-03
CEACAM7	No	No	No	Down	0.74	0.003939	validated	-0.02	0.10	9.44E-01	-0.55	0.04	7.70E-04	-0.65	0.11	6.40E-03	-0.49	0.12	5.81E-02	-0.54	0.14	1.59E-01	-0.58	0.21	3.70E-01	-0.53	0.10	1.84E-02	-0.07	0.03	5.30E-01
FAM3D	No	Yes	No	Down	0.74	0.001506	1.5E-19	-0.55	0.10	2.33E-02	-0.77	0.04	5.91E-06	-0.21	0.10	3.52E-01	-0.43	0.12	8.60E-02	-0.55	0.14	2.66E-01	-0.35	0.20	4.46E-01	-0.28	0.10	3.63E-01	-0.26	0.03	9.35E-02
CANX	No	No	No	Down	0.75	0.028966	validated	-0.67	0.11	4.98E-02	-0.45	0.04	2.41E-02	-0.92	0.11	1.19E-02	-0.02	0.11	9.56E-01	-0.18	0.14	3.86E-01	-0.61	0.21	9.32E-02	-0.18	0.10	4.01E-01	-0.35	0.03	1.13E-01
C11ORF70	No	No	No	Down	0.75	0.000588	validated	-0.38	0.10	9.78E-02	-0.43	0.04	7.49E-03	-0.09	0.10	6.50E-01	-0.15	0.11	5.57E-01	-0.82	0.15	7.94E-03	-0.24	0.20	3.54E-01	-0.79	0.10	1.83E-03	-0.49	0.03	1.29E-03
PMCH	No	Yes	No	Down	0.75	0.008189	0.00767	-0.04	0.10	8.81E-01	-0.35	0.04	2.65E-02	-0.58	0.11	1.69E-02	-0.34	0.11	1.49E-01	-0.70	0.14	4.64E-01	-0.52	0.21	6.14E-01	-0.32	0.10	1.58E-01	-0.54	0.03	3.29E-04
ACSL4	No	No	No	Down	0.75	0.005902	0.02521	-0.36	0.10	1.51E-01	-0.46	0.04	8.21E-03	-0.11	0.10	6.10E-01	-0.05	0.11	8.69E-01	-0.91	0.15	4.13E-03	-0.74	0.21	2.82E-02	-0.65	0.10	8.48E-03	-0.08	0.03	6.16E-01
HNMT	No	No	Yes	Down	0.75	0.002576	validated	-0.47	0.10	8.82E-02	-0.73	0.04	1.46E-04	-0.43	0.10	8.69E-02	-0.11	0.11	7.09E-01	-0.38	0.14	1.54E-01	-0.29	0.20	3.56E-01	-0.73	0.10	6.85E-03	-0.22	0.03	1.91E-01
HSD17B3	No	No	No	Down	0.75	0.041408	validated	-0.35	0.10	3.57E-01	-0.25	0.04	2.64E-01	-0.66	0.11	6.75E-02	-0.82	0.12	7.17E-03	-0.29	0.14	4.63E-01	-0.16	0.20	9.24E-01	-0.52	0.10	4.98E-02	-0.30	0.03	6.10E-02
ZNF750	No	Yes	No	Down	0.75	0.000103	0.0007	-0.12	0.10	5.75E-01	-0.92	0.04	1.41E-05	-0.44	0.10	4.87E-02	-0.32	0.11	2.00E-01	-0.55	0.14	1.35E-01	-0.15	0.20	6.64E-01	-0.16	0.10	5.04E-01	-0.68	0.03	1.70E-05
FGF19	No	Yes	No	Down	0.75	0.01272	9.4E-05	-0.58	0.10	1.81E-02	-0.08	0.04	5.90E-01	-0.49	0.10	5.69E-02	-0.52	0.12	5.16E-02	-0.54	0.14	2.50E-01	-0.44	0.20	1.27E-01	-0.32	0.10	2.17E-01	-0.36	0.03	1.24E-02
PHYHIPL	No	Yes	No	Down	0.75	0.003694	0.00345	-1.27	0.12	1.33E-04	-0.35	0.04	2.19E-02	-0.73	0.11	5.74E-03	-0.06	0.11	7.97E-01	-0.42	0.14	1.28E-01	-0.08	0.20	7.24E-01	-0.34	0.10	1.87E-01	-0.09	0.03	5.78E-01
NUS1	No	No	No	Down	0.75	0.023125	validated	-0.46	0.10	1.09E-01	-0.22	0.04	1.23E-01	-0.26	0.10	3.29E-01	-0.91	0.12	2.47E-03	-0.61	0.14	6.11E-01	-0.20	0.20	8.51E-01	-0.10	0.10	6.62E-01	-0.56	0.03	3.26E-03
CPT2	No	No	No	Down	0.75	0.043874	validated	-0.56	0.10	1.00E-01	-0.47	0.04	2.07E-02	-0.76	0.11	3.90E-02	-0.28	0.11	4.24E-01	-0.67	0.14	3.60E-02	-0.31	0.20	2.64E-01	-0.02	0.10	9.29E-01	-0.23	0.03	2.29E-01
GPR157	No	Yes	No	Down	0.75	0.048102	0.00123	-1.13	0.11	1.35E-03	-0.37	0.04	2.75E-02	-0.15	0.10	5.64E-01	-0.69	0.12	1.95E-02	-0.21	0.14	8.47E-01	-0.50	0.21	7.71E-01	-0.24	0.10	3.57E-01	-0.01	0.03	9.55E-01

Supplementary Table 2. Details for 2,053 genes differentially expressed between EEC and NEEC, identified by microarray meta-analysis.

EEC - endometrioid endometrial cancer, NEEC - non-endometrioid endometrial cancer, SMD - Standardised Mean Difference, FDR - False Discovery Rate, Var - Variance

Gene	145 gene list	1253 gene list	Associated with EEC-specific survival?	Up/Down Regulated in NEEC	average standardised fold change	Microarray Meta FDR	RNASeq FDR	Individual microarray study results: TCGA			E-MTAB-2532			E-GEOD-56026			GSE32507			GSE24537			E-GEOD-23518			E-GEOD-17025			E-GEOD-2109		
								SMD	Var	P-value	SMD	Var	P-value	SMD	Var	P-value	SMD	Var	P-value	SMD	Var	P-value	SMD	Var	P-value	SMD	Var	P-value	SMD	Var	P-value
DPEP1	No	No	No	Down	0.75	0.025326	validated	-0.51	0.10	2.56E-02	-0.41	0.04	1.19E-02	-0.36	0.10	1.73E-01	-0.36	0.11	1.83E-01	-0.63	0.14	2.96E-01	-0.52	0.21	1.86E-01	-0.31	0.10	1.89E-01	-0.20	0.03	1.57E-01
HEY2	No	No	No	Down	0.75	0.005801	0.01742	-0.56	0.10	2.31E-02	-0.20	0.04	1.53E-01	-0.26	0.10	2.27E-01	-0.23	0.11	3.53E-01	-0.70	0.14	1.32E-02	-0.43	0.20	1.43E-01	-0.64	0.10	9.85E-03	-0.27	0.03	7.32E-02
EPHA7	No	Yes	No	Down	0.75	0.001768	1.5E-19	-0.35	0.10	1.75E-01	-0.47	0.04	5.47E-03	-0.82	0.11	1.31E-03	-0.42	0.12	8.20E-02	-0.20	0.14	9.00E-01	-0.03	0.20	9.85E-01	-0.86	0.10	6.55E-04	-0.12	0.03	3.77E-01
PLAC8	No	Yes	No	Down	0.75	0.010927	0.02931	-0.27	0.10	2.52E-01	-0.52	0.04	1.77E-03	-0.53	0.11	2.95E-02	-0.05	0.11	8.55E-01	-0.52	0.14	9.19E-02	-0.80	0.22	1.06E-01	-0.22	0.10	3.39E-01	-0.33	0.03	4.96E-02
LYN	No	No	No	Down	0.76	0.008182	validated	-0.26	0.10	4.29E-01	-0.54	0.04	6.28E-03	-0.44	0.10	1.04E-01	-0.15	0.11	6.56E-01	-0.26	0.14	2.25E-01	-0.70	0.21	3.43E-02	-0.12	0.10	6.10E-01	-0.77	0.03	1.00E-03
DYDC2	No	No	No	Down	0.76	0.027615	validated	-0.05	0.10	8.26E-01	-0.59	0.04	2.67E-04	-0.24	0.10	2.87E-01	-0.11	0.11	6.34E-01	-0.86	0.15	1.19E-01	-0.69	0.21	3.47E-01	-0.53	0.10	4.99E-02	-0.16	0.03	2.41E-01
NLRC3	No	Yes	Yes	Down	0.76	0.033818	0.0257	-0.61	0.10	7.15E-02	-0.48	0.04	7.34E-03	-0.52	0.11	6.36E-02	-0.48	0.12	9.70E-02	-0.12	0.14	7.87E-01	-0.45	0.21	4.74E-01	-0.29	0.10	2.21E-01	-0.24	0.03	1.28E-01
HOXB5	No	No	No	Down	0.76	0.010931	validated	-0.30	0.10	1.84E-01	-0.23	0.04	7.59E-02	-0.48	0.10	5.76E-02	-0.55	0.12	2.67E-02	-0.66	0.14	1.29E-02	-0.57	0.21	6.00E-02	-0.26	0.10	2.74E-01	-0.14	0.03	4.05E-01
CD2	No	Yes	Yes	Down	0.76	0.009629	0.03784	-0.64	0.11	9.80E-03	-0.34	0.04	2.34E-02	-0.59	0.11	3.72E-02	-0.62	0.12	2.75E-02	-0.37	0.14	2.33E-01	-0.21	0.20	4.44E-01	-0.05	0.10	8.30E-01	-0.33	0.03	6.59E-02
ELP2	No	No	No	Down	0.76	0.024031	validated	-0.01	0.10	9.63E-01	-0.31	0.04	1.40E-01	-0.85	0.11	1.41E-02	-0.01	0.11	9.87E-01	-0.37	0.14	1.81E-01	-0.14	0.20	5.38E-01	-1.30	0.11	2.11E-04	-0.15	0.03	3.50E-01
RARRES3	No	No	No	Down	0.76	0.002071	validated	-0.11	0.10	6.61E-01	-0.36	0.04	1.76E-02	-0.40	0.10	8.74E-02	-0.55	0.12	4.93E-02	-0.08	0.14	5.45E-01	-0.40	0.20	1.31E-01	-0.57	0.10	2.42E-02	-0.65	0.03	3.75E-04
ABCG1	No	No	No	Down	0.76	0.018561	0.0496	-0.52	0.10	6.36E-02	-0.64	0.04	8.09E-04	-0.16	0.10	5.32E-01	-0.53	0.12	1.05E-01	-0.42	0.14	1.43E-01	-0.42	0.20	2.40E-01	-0.07	0.10	7.52E-01	-0.33	0.03	9.52E-02
NKD1	No	Yes	No	Down	0.77	0.019892	0.00101	-0.68	0.11	5.79E-03	-0.17	0.04	2.15E-01	-0.39	0.10	1.13E-01	-0.20	0.11	3.84E-01	-0.47	0.14	1.15E-01	-0.61	0.21	5.13E-02	-0.51	0.10	2.80E-02	-0.01	0.03	9.51E-01
SLC28A3	No	Yes	No	Down	0.77	0.008311	0.00885	-0.03	0.10	9.06E-01	-0.59	0.04	4.39E-03	-0.27	0.10	2.22E-01	-0.33	0.11	2.10E-01	-0.25	0.14	6.47E-01	-0.57	0.21	4.38E-01	-0.25	0.10	2.25E-01	-0.73	0.03	7.66E-05
CD3D	No	Yes	Yes	Down	0.77	0.017044	0.00715	-0.65	0.11	2.09E-02	-0.44	0.04	7.17E-03	-0.66	0.11	3.55E-02	-0.32	0.11	2.00E-01	-0.20	0.14	4.45E-01	-0.15	0.20	5.36E-01	-0.27	0.10	3.16E-01	-0.31	0.03	6.98E-02
TPD52L1	No	No	No	Down	0.77	0.013996	validated	-0.03	0.10	9.07E-01	-0.46	0.04	5.92E-03	-0.70	0.11	1.06E-02	-0.10	0.11	7.23E-01	-0.70	0.14	1.45E-02	-0.77	0.21	2.36E-02	-0.05	0.10	8.43E-01	-0.19	0.03	2.87E-01
NOTUM	No	No	No	Down	0.77	0.026361	validated	-0.42	0.10	6.00E-02	-0.10	0.04	4.21E-01	-0.29	0.10	2.18E-01	-0.36	0.11	1.36E-01	-0.47	0.14	6.69E-02	-0.80	0.22	1.60E-02	-0.35	0.10	1.73E-01	-0.20	0.03	1.26E-01
DDIT4L	No	Yes	Yes	Down	0.77	0.005375	1.5E-19	-0.68	0.11	6.29E-03	-0.27	0.04	5.06E-02	-0.57	0.11	1.98E-02	-0.13	0.11	5.81E-01	-0.04	0.14	7.32E-01	-0.40	0.20	1.43E-01	-0.82	0.10	1.39E-03	-0.07	0.03	5.96E-01
BCAS1	No	No	No	Down	0.77	0.043449	validated	-0.53	0.10	6.22E-02	-0.55	0.04	2.33E-03	-0.10	0.10	7.79E-01	-0.51	0.12	8.18E-02	-0.52	0.14	2.83E-01	-0.32	0.20	5.58E-01	-0.16	0.10	6.19E-01	-0.28	0.03	7.45E-02
UHMK1	No	No	No	Down	0.78	0.048752	validated	-0.49	0.10	1.01E-01	-0.37	0.04	6.25E-02	-0.12	0.10	6.41E-01	-0.04	0.11	9.20E-01	-0.16	0.14	5.64E-01	-0.27	0.20	6.13E-01	-1.02	0.10	1.60E-03	-0.41	0.03	4.57E-02
CCL21	No	No	No	Down	0.78	0.038456	0.03038	-0.31	0.10	3.45E-01	-0.54	0.04	2.76E-03	-0.10	0.10	6.71E-01	-0.67	0.12	6.07E-02	-0.04	0.14	7.61E-01	-0.80	0.22	3.36E-02	-0.13	0.10	7.85E-01	-0.28	0.03	6.22E-02
C1ORF116	No	No	No	Down	0.78	0.00225	validated	-0.02	0.10	9.19E-01	-0.47	0.04	3.49E-03	-0.48	0.10	4.70E-02	-0.57	0.12	3.72E-02	-0.16	0.14	4.33E-01	-0.42	0.20	2.54E-01	-0.13	0.10	5.28E-01	-0.61	0.03	1.24E-04
TRIM22	No	No	Yes	Down	0.78	0.006067	validated	-0.44	0.10	9.18E-02	-0.73	0.04	2.61E-04	-0.05	0.10	8.27E-01	-0.25	0.11	3.55E-01	-0.01	0.14	9.63E-01	-0.40	0.20	2.29E-01	-0.30	0.10	1.73E-01	-0.69	0.03	1.60E-03
SEC16B	No	Yes	No	Down	0.78	0.014126	0.03499	-0.20	0.10	6.10E-01	-0.72	0.04	2.94E-04	-0.67	0.11	1.46E-02	-0.77	0.12	1.01E-02	-0.08	0.14	9.45E-01	-0.03	0.20	9.81E-01	-0.25	0.10	2.62E-01	-0.10	0.03	5.40E-01
RFX2	No	Yes	No	Down	0.79	0.024042	0.00383	-0.77	0.11	1.60E-02	-0.53	0.04	1.66E-03	-0.20	0.10	4.41E-01	-0.22	0.11	4.24E-01	-0.36	0.14	2.37E-01	-0.31	0.20	3.28E-01	-0.04	0.10	8.54E-01	-0.32	0.03	4.12E-02
TRIM31	No	No	No	Down	0.79	0.028534	validated	-0.28	0.10	3.06E-01	-0.60	0.04	4.28E-03	-0.60	0.11	1.93E-02	-0.71	0.12	8.08E-03	-0.41	0.14	1.73E-01	-0.02	0.20	9.60E-01	-0.11	0.10	6.50E-01	0.00	0.03	9.83E-01
CD1A	No	No	No	Down	0.79	0.040911	validated	-0.23	0.10	3.52E-01	-0.70	0.04	1.32E-04	-0.17	0.10	5.24E-01	-0.30	0.11	2.50E-01	-0.36	0.14	3.20E-01	-0.28	0.20	3.99E-01	-0.59	0.10	1.06E-01	-0.07	0.03	6.94E-01
CXCL5	No	No	No	Down	0.80	0.015848	validated	-0.25	0.10	3.91E-01	-0.27	0.04	4.33E-02	-0.16	0.10	3.80E-01	-0.25	0.11	2.76E-01	-0.69	0.14	2.41E-02	-0.02	0.20	8.96E-01	-0.77	0.10	1.12E-03	-0.21	0.03	1.10E-01
EPDR1	No	No	No	Down	0.80	0.013475	0.03379	-0.44	0.10	7.69E-02	-0.51	0.04	1.92E-03	-0.36	0.10	1.07E-01	-0.28	0.11	2.35E-01	-0.30	0.14	1.66E-01	-0.16	0.20	4.19E-01	-0.15	0.10	4.53E-01	-0.40	0.03	2.65E-02
RBP1	No	No	No	Down	0.81	0.035912	validated	-0.04	0.10	8.70E-01	-0.11	0.04	4.18E-01	-0.05	0.10	7.97E-01	-0.12	0.11	6.55E-01	-0.86	0.15	2.49E-03	-0.31	0.20	2.03E-01	-0.76	0.10	3.19E-03	-0.23	0.03	1.31E-01
DPYSL4	No	No	No	Up	1.22	0.034747	validated	0.05	0.10	8.23E-01	0.43	0.04	8.51E-03	0.06	0.10	8.21E-01	1.30	0.14	6.11E-05	0.30	0.14	2.09E-01	0.12	0.20	5.75E-01	0.00	0.10	9.94E-01	0.02	0.03	8.73E-01
GALNT14	No	Yes	No	Up	1.25	0.004032	1.5E-19	0.57	0.10	9.38E-03	0.51	0.04	5.77E-04	0.28	0.10	2.10E-01	0.25	0.11	2.64E-01	0.33	0.14	5.37E-01	0.11	0.20	8.50E-01	0.09	0.10	6.85E-01	0.47	0.03	1.76E-03
CLIC4	No	No	No	Up	1.27	0.04916	validated	0.15	0.10	6.24E-01	0.05	0.04	7.37E-01	0.37	0.10	1.93E-01	1.32	0.14	1.48E-04	0.19	0.14	3.30E-01	0.35	0.20	1.96E-01	0.02	0.10	9.26E-01	0.30	0.03	1.23E-01
C3ORF14	No	No	No	Up	1.28	0.012991	0.00249	0.32	0.10	1.49E-01	0.46	0.04	5.40E-03	0.35	0.10	1.26E-01	0.33	0.11	1.91E-01	0.26	0.14	2.66E-01	0.23	0.20	3.83E-01	0.53	0.10	3.06E-02	0.33	0.03	4.67E-02
IFITM2	No	No	No	Up	1.28	0.021189	validated	0.78	0.11	4.98E-03	0.30	0.04	9.49E-02	0.28	0.10	3.30E-01	0.17	0.11	5.59E-01	1.01	0.15	6.77E-04	0.30	0.20	2.14E-01	0.01	0.10	9.79E-01	0.05	0.03	8.02E-01
CSPG5	No	No	No	Up	1.29	0.03981	0.04384	0.79	0.11	1.73E-02	0.31	0.04	9.53E-02	0.44	0.10	6.73E-02	0.17	0.11	6.21E-01	0.24	0.14	3.80E-01	0.34	0.20	2.95E-01	0.21	0.10	3.59E-01	0.45	0.03	1.91E-02
PRRT3	No	No	No	Up	1.29	0.024577	validated	0.30	0.10	2.73E-01	0.97	0.04	1.86E-05	0.41	0.10	1.63E-01	0.26	0.11	4.75E-01	0.04	0.14	8.49E-01	0.58	0.21	2.86E-01	0.26	0.10	3.85E-01	0.15	0.03	3.91E-01
IRF9	No	Yes	No	Up	1.30	0.037015	0.0022	0.57	0.10	4.71E-02	0.42	0.04	2.99E-02	0.71	0.11	2.21E-02	0.03	0.11	9.21E-01	0.44	0.14	7.88E-02	0.66	0.21	4.03E-02	0.18	0.10	4.95E-01	0.01	0.03	9.65E-01
BMP2	No	No	No	Up	1.30	0.018217	validated	0.09	0.10	6.93E-01	0.21	0.04	1.87E-01	0.18	0.10	3.85E-01	0.21	0.11	3.73E-01	0.81	0.15	2.57E-02	0.59	0.21	6.95E-02	0.47	0.10	3.81E-02	0.45	0.03	5.31E-03
TEX14	No	No	No	Up	1.30	0.037019	validated	0.59	0.10	7.78E-02	0.71	0.04	3.67E-03	0.16	0.10	7.50E-01	0.63	0.12	2.29E-02	0.22	0.14	8.67E-01	0.23	0.20	8.44E-01	0.13	0.10	8.16E-01	0.38	0.03	1.49E-02
CCNJL	No	No	Yes	Up	1.30	0.039465	0.00748	0.29	0.10	2.64E-01	0.78	0.04	1.48E-04	0.30	0.10	3.34E-01	0.27	0.11	3.02E-01	0.43	0.14	5.55E-01	0.33	0.20	5.89E-01	0.34	0.10	3.73E-01	0.31	0.03	1.08E-01
ZNF462	No	No	No	Up	1.30	0.015276	0.00903	0.48	0.10	5.17E-02	0.31	0.04	5.01E-02	0.61	0.11	2.81E-02	0.25	0.11	3.39E-01	0.40	0.14	1.17E-01	0.15	0.20	4.78E-01	0.55	0.10	3.97E-02	0.32	0.03	7.75E-02
OAF	No	No	No	Up	1.31	0.040572	validated	0.17	0.10	5.68E-01	0.14	0.04	4.10E-01	0.07	0.10	8.42E-01	0.62	0.12	4.59E-02	0.92	0.15	2.21E-03	0.81	0.22	1.38E-02	0.17	0.10	6.36E-01	0.19	0.03	3.21E-01
PPAT	No	No	Yes	Up	1.31	0.017915	0.01053	0.03	0.10	9.08E-01	0.73	0.04	4.60E-04	0.27	0.10	3.36E-01	0.41	0.12	2.52E-01	0.27	0.14	3.33E-01	0.45	0.20	2.02E-01	0.27	0.10	2.68E-01	0.66	0.03	1.01E-02
C16ORF59	No	No	No	Up	1.31	0.020096	0.01938	0.38	0.10	2.26E-01	0.79	0.04	1.05E-04	0.15	0.10	6.74E-01	0.47	0.12	1.83E-01	0.28	0.14	3.46E-01	0.41	0.20	3.16E-01	0.37	0.10	2.54E-01	0.25	0.03	1.08E-01
XKR6	No	Yes	No	Up	1.31	0.001122	1.5E-19	0.71	0.11	6.53E-03	0.97	0.04	1.32E-05	0.21	0.10	4.44E-01	0.01	0.11	9.46E-01	0.10	0.14	7.39E-01	0.35	0.20	5.04E-01	0.17	0.10	5.64E-01	0.57	0.03	1.19E-03
WNK1	No	No	No	Up	1.31	0.01396	validated	0.10	0.10	7.85E-01	0.72	0.04	4.67E-04	0.14	0.10	5.89E-01	0.76	0.12	1.62E-02	0.73	0.14	3.73E-02	0.16	0.20	5.10E-01	0.38	0.10	1.27E-01	0.13	0.03	4.00E-01
KIAA1324L	No	No	No	Up	1.31	0.008323	0.02081	0.15	0.10	4.67E-01	0.56	0.04	8.92E-04	0.61	0.11	2.28E-02	0.40	0.12	9.90E-02	0.81	0.15	3.34E-02	0.04	0.20	8.60E-01	0.35	0.10	1.51E-01	0.20	0.03	2.23E-01
SLC11A1	No	No	No	Up	1.31	0.030014	validated	0.57	0.10	1.62E-01	0.17	0.04	3.02E-01	0.04	0.10	9.14E-01	0.48	0.12	1.27E-01	0.42	0.14	2.05E-01	0.07	0.20	7.47E-01	0.76	0.10	8.34E-02	0.62	0.03	3.37E-04
MAD2L1	No	Yes	No	Up	1.31	0.011795	0.0007	0.16	0.10	5.31E-01	0.21	0.04	1.40E-01	0.12	0.10	5.92E-01	1.01	0.13	2.37E-03	0.35	0.14	1.52E-01	0.38	0.20	1.74E-01	0.48	0.10	3.77E-02	0.45	0.03	2.14E-02
PKMYT1	No	Yes	No	Up	1.31	0.035855	0.00046	0.49	0.10	1.43E-01	0.66	0.04	5.47E-04	0.03	0.10	9.41E-01	0.00	0.11	9.97E-01	0.42	0.14	1.96E-01	0.51	0.21	1.99E-01	0.80	0.10	5.67E-02	0.25	0.03	2.46E-01
C20ORF160	No	No	No	Up	1.31	0.048812	validated	0.58	0.10	4.97E-02	0.11	0.04	5.57E-01	0.30	0.10	2.88E-01	0.79	0.12	1.44E-02	0.58	0.14	4.57E-02	0.03	0.20	8.70E-01	0.47	0.10	1.61E-01	0.29	0.03	1.29E-01
RGMA	No	No	No	Up	1.32	0.035146	validated	0.13	0.10	5.98E-01	0.03	0.04	8.08E-01	0.09	0.10	7.37E-01	0.76	0.12	5.67E-03	0.32	0.14	1.77E-01	1.05	0.23	8.74E-03	0.59	0.10	4.76E-02	0.18	0.03	2.55E-01
EZH2	No	No	No	Up	1.32	0.014895	validated	0.47	0.10	1.65E-01	0.56	0.04	2.80E-03	0.22	0.10	3.65E-01	0.69	0.12	6.23E-02	0.05	0.14	7.94E-01	0.03	0.20	9.10E-01	0.58	0.10	4.11E-02	0.57	0.03	1.28E-02
NAALAD2	No	Yes	No	Up	1.32	0.021713	1.5E-19	0.36	0.10	2.83E-01	0.50	0.04	3.57E-02	0.69	0.11	1.56E-02	0.59	0.12	4.71E-02	0.40	0.14	3.45E-01	0.16	0.20	7.01E-01	0.11	0.10	7.71E-01	0.41	0.03	7.58E-03
SLC16A10	No	Yes	No	Up	1.32	0.00815	2.6E-05	0.51	0.10	8.09E-02	0.48	0.04	1.55E-02	0.12	0.10	5.94E-01	0.65	0.12	2.42E-02	0.44	0.14	1.59E-01	0.39	0.20	1.98E-01	0.23	0.10	2.91E-01	0.41	0.03	5.15E-03
NUSAP1	No	No	No	Up	1.33	0.014651	0.03724	0.48	0.10	1.01E-01	0.28	0.04	1.02E-01	0.03	0.10	8.81E-01	1.08	0.13	2.96E-03	0.13	0.14	4.51E-01	0.29	0.20	2.61E-01	0.82	0.10	3.46E-03	0.15	0.03	3.47E-01
SLC35F5	No	Yes	No	Up	1.33	0.012802	1.5E-19	1.00	0.11	1.63E-03	0.38	0.04	4.19E-02	0.61	0.11	2.25E-02	0.22	0.11	6.17E-01	0.25	0.14	3.15E-01	0.02	0.20	9.29E-01	0.68	0.10	1.99E-02	0.10	0.03	4.89E-01
NCAPG2	No	No	No	Up	1.33	0.01861	0.01416	0.24	0.10	4.02E-01	0.76	0.04	5.49E-04	0.22	0.10	4.19E-01	0.42	0.12	2.39E-01	0.13	0.14	5.79E-01	0.53	0.21	2.21E-01	0.39	0.10	1.60E-01	0.61	0.03	1.56E-02
GSTM2	No	No	No	Up	1.33	0.006141	0.01079	0.92	0.11	4.03E-03	0.63	0.04	1.84E-03	0.71	0.11	2.44E-02	0.17	0.11	4.86E-01	0.30	0.14	1.52E-01	0.28	0.20	2.35E-01	0.02	0.10	9.14E-01	0.28	0.03	1.55E-01
ZSCAN20	No	No	No	Up	1.33	0.028802	validated	1.05	0.11	4.85E-03	0.88	0.04	9.95E-04	0.07	0.10	8.47E-01	0.48	0.12	2.40E-01	0.35	0.14	6.64E-01	0.09	0.20	8.88E-01	0.01	0.10	9.80E-01	0.39	0.03	3.91E-02
TEX15	No	Yes	No	Up	1.33	0.012903	1.5E-19	0.30	0.10	2.94E-01	0.17	0.04	4.09E-01	0.49	0.10	5.28E-02	0.61	0.12	3.16E-02	0.45	0.14	2.01E-01	0.58	0.21	1.71E-01	0.20	0.10	7.03E-01	0.54	0.03	5.73E-04
SNAI1	No	No	No	Up	1.34	0.007291	validated	0.52	0.10	6.66E-02	0.55	0.04	2.17E-03	0.45	0.10	2.91E-01	1.00	0.13	3.93E-03	0.15	0.14	8.89E-01	0.04	0.20	9.71E-01	0.13	0.10	7.50E-01	0.48	0.03	6.16E-03
FANCD2	No	Yes	No	Up	1.34	0.007522	0.00266	0.34	0.10	3.52E-01	0.59	0.04	1.69E-03	0.03	0.10	9.11E-01	0.76	0.12	2.54E-02	0.14	0.14	5.62E-01	0.19	0.20	4.65E-01	1.03	0.10	1.14E-03	0.28	0.03	1.74E-01
THBS2	No	No	No	Up	1.34	0.002006	validated	0.14	0.10	5.45E-01	0.21	0.04	1.10E-01	0.07	0.10	7.35E-01	0.98	0.13	1.49E-03	0.94	0.15	2.07E-03	0.48	0.21	9.15E-02	0.04	0.10	8.63E-01	0.50	0.03	2.81E-03
NPHP3	No	No	No	Up	1.34	0.026979	validated	0.02	0.10	9.43E-01	0.25	0.04	1.82E-01	0.29	0.10	2.47E-01	0.03	0.11	9.33E-01	0.83	0.15	2.85E-02	0.67	0.21	1.24E-01	1.06	0.10	7.45E-04	0.23	0.03	1.78E-01
TIMELESS	No	Yes	No	Up	1.34	0.01685	0.0088	0.73	0.11	4.11E-02	0.77	0.04	5.10E-04	0.46	0.10	1.04E-01	0.15	0.11	6.81E-01	0.28	0.14	2.45E-01	0.53	0.21	1.18E-01	0.38	0.10	1.91E-01	0.08	0.03	6.59E-01
PPP3R1	No	Yes	No	Up	1.34	0.029828	0.0413	0.76	0.11	3.40E-02	0.56	0.04	1.30E-02	0.47	0.10	1.15E-01	0.17	0.11	6.56E-01	0.30	0.14	1.98E-01	0.69	0.21	4.12E-02	0.14	0.10	5.94E-01	0.30	0.03	1.49E-01
ANKS1A	No	Yes	No	Up	1.34	0.042339	0.0103	0.82	0.11	2.24E-02	0.00	0.04	9.84E-01	0.41	0.10	1.96E-01	0.13	0.11	7.80E-01	0.47	0.14	6.04E-02	0.10	0.20	5.90E-01	0.81	0.10	2.36E-02	0.66	0.03	2.23E-02

Supplementary Table 2. Details for 2,053 genes differentially expressed between EEC and NEEC, identified by microarray meta-analysis.

EEC - endometrioid endometrial cancer, NEEC - non-endometrioid endometrial cancer, SMD - Standardised Mean Difference, FDR - False Discovery Rate, Var - Variance

Gene	145 gene list	1253 gene list	Associated with EEC-specific survival?	Up/Down Regulated in NEEC	average standardised fold change	Microarray Meta FDR	RNASeq FDR	Individual microarray study results: TCGA			E-MTAB-2532			E-GEOD-56026			GSE32507			GSE24537			E-GEOD-23518			E-GEOD-17025			E-GEOD-2109		
								SMD	Var	P-value	SMD	Var	P-value	SMD	Var	P-value	SMD	Var	P-value	SMD	Var	P-value	SMD	Var	P-value	SMD	Var	P-value	SMD	Var	P-value
BAALC	No	No	No	Up	1.34	0.02326	validated	0.31	0.10	2.43E-01	0.11	0.04	5.58E-01	0.42	0.10	1.42E-01	1.18	0.13	1.52E-04	0.06	0.14	7.49E-01	0.85	0.22	1.94E-01	0.16	0.10	5.93E-01	0.30	0.03	4.93E-02
TNRC6C	No	Yes	No	Up	1.34	0.042508	0.00649	0.12	0.10	7.74E-01	0.40	0.04	2.22E-02	0.36	0.10	2.01E-01	0.63	0.12	9.36E-02	0.71	0.14	5.02E-01	0.37	0.20	6.22E-01	0.22	0.10	4.56E-01	0.60	0.03	1.38E-03
MKX	No	No	No	Up	1.34	0.001465	validated	0.32	0.10	1.80E-01	0.31	0.04	5.51E-02	0.38	0.10	7.89E-02	0.55	0.12	3.28E-02	0.57	0.14	3.16E-02	0.44	0.20	1.14E-01	0.33	0.10	1.27E-01	0.50	0.03	1.31E-03
PIWIL4	No	No	No	Up	1.35	0.048442	0.01117	0.77	0.11	1.11E-02	0.27	0.04	1.22E-01	0.26	0.10	3.26E-01	0.30	0.11	2.92E-01	0.69	0.14	3.90E-01	0.03	0.20	9.63E-01	0.90	0.10	7.09E-03	0.21	0.03	2.69E-01
LOX	No	No	No	Up	1.35	0.001052	validated	0.09	0.10	7.01E-01	0.35	0.04	2.39E-02	0.24	0.10	2.51E-01	0.87	0.12	2.24E-03	0.53	0.14	5.49E-02	0.53	0.21	9.42E-02	0.27	0.10	2.13E-01	0.54	0.03	7.43E-04
HOMER3	No	No	No	Up	1.35	0.031737	validated	0.52	0.10	9.24E-02	0.49	0.04	1.32E-02	0.51	0.10	1.15E-01	0.50	0.12	1.70E-01	0.44	0.14	1.72E-01	0.63	0.21	1.55E-01	0.01	0.10	9.64E-01	0.33	0.03	4.66E-02
PSIP1	No	No	No	Up	1.35	0.016929	validated	0.11	0.10	7.40E-01	0.62	0.04	3.25E-03	0.61	0.11	7.00E-02	0.06	0.11	8.48E-01	0.56	0.14	1.40E-01	0.58	0.21	1.80E-01	0.33	0.10	1.71E-01	0.57	0.03	8.99E-03
ANGPTL7	No	No	No	Up	1.35	0.035025	0.00068	0.64	0.11	1.66E-02	0.45	0.04	3.79E-02	0.09	0.10	8.66E-01	0.49	0.12	5.18E-02	0.74	0.14	3.49E-01	0.15	0.20	9.04E-01	0.49	0.10	3.05E-01	0.41	0.03	1.38E-02
LMNB2	No	No	No	Up	1.35	0.021968	validated	0.16	0.10	6.29E-01	0.41	0.04	3.28E-02	0.26	0.10	3.27E-01	0.37	0.11	3.38E-01	0.77	0.15	1.52E-02	0.70	0.21	4.13E-02	0.41	0.10	1.20E-01	0.37	0.03	6.24E-02
CSDA	No	Yes	No	Up	1.35	0.005735	0.00211	0.22	0.10	4.67E-01	0.63	0.04	1.66E-03	0.85	0.11	1.27E-02	0.03	0.11	9.00E-01	0.27	0.14	1.95E-01	0.53	0.21	6.89E-02	0.80	0.10	7.67E-03	0.14	0.03	4.15E-01
CHRNA5	No	No	No	Up	1.35	0.004549	validated	0.12	0.10	6.17E-01	0.43	0.04	1.37E-02	0.84	0.11	4.83E-03	0.71	0.12	1.25E-02	0.43	0.14	8.09E-02	0.22	0.20	3.43E-01	0.30	0.10	4.37E-01	0.43	0.03	3.57E-02
KIF14	No	No	No	Up	1.35	0.005485	0.00394	0.24	0.10	3.97E-01	0.49	0.04	2.05E-02	0.10	0.10	6.56E-01	0.78	0.12	1.24E-02	0.40	0.14	2.64E-01	0.24	0.20	4.28E-01	0.60	0.10	1.64E-02	0.62	0.03	2.74E-03
USP42	No	No	No	Up	1.35	0.030408	validated	0.08	0.10	8.31E-01	0.07	0.04	6.56E-01	0.17	0.10	6.33E-01	0.73	0.12	8.65E-02	0.41	0.14	1.94E-01	0.70	0.21	9.00E-02	0.90	0.10	2.81E-03	0.41	0.03	1.74E-02
BRSK1	No	No	No	Up	1.35	0.029988	validated	0.09	0.10	8.08E-01	0.70	0.04	1.73E-02	0.05	0.10	8.94E-01	0.15	0.11	5.81E-01	0.26	0.14	2.64E-01	0.75	0.21	5.47E-02	1.07	0.10	1.87E-02	0.44	0.03	1.25E-02
KCNC1	No	No	No	Up	1.35	0.000235	0.01499	0.43	0.10	7.39E-02	0.81	0.04	5.57E-04	0.04	0.10	9.09E-01	1.13	0.13	1.08E-04	0.26	0.14	8.31E-01	0.10	0.20	9.36E-01	0.09	0.10	8.26E-01	0.65	0.03	1.23E-04
MCM6	No	No	No	Up	1.35	0.004078	0.02799	0.25	0.10	3.82E-01	0.63	0.04	1.33E-03	0.43	0.10	1.44E-01	0.99	0.13	1.00E-02	0.48	0.14	5.64E-02	0.04	0.20	7.69E-01	0.22	0.10	3.78E-01	0.45	0.03	1.43E-02
C16ORF45	No	No	No	Up	1.36	0.03159	validated	0.10	0.10	7.04E-01	0.06	0.04	6.56E-01	0.37	0.10	2.20E-01	0.82	0.12	7.55E-03	0.89	0.15	3.52E-02	0.25	0.20	4.30E-01	0.59	0.10	6.12E-02	0.43	0.03	3.97E-02
ADAMTS1	No	Yes	No	Up	1.36	0.002576	0.00013	0.63	0.10	1.56E-02	0.57	0.04	1.31E-03	0.25	0.10	2.49E-01	0.25	0.11	3.54E-01	0.61	0.14	4.71E-02	0.59	0.21	8.62E-02	0.22	0.10	3.10E-01	0.40	0.03	2.49E-02
CD47	No	Yes	No	Up	1.36	0.002704	1.5E-19	1.33	0.12	1.69E-04	0.32	0.04	3.38E-02	0.22	0.10	3.55E-01	0.55	0.12	9.40E-02	0.14	0.14	4.72E-01	0.11	0.20	5.64E-01	0.53	0.10	2.42E-02	0.33	0.03	5.09E-02
GABARAPL1	No	No	No	Up	1.36	0.020271	0.01843	0.53	0.10	5.68E-02	0.02	0.04	9.14E-01	0.55	0.11	6.44E-02	0.42	0.12	1.68E-01	0.82	0.15	9.78E-03	0.07	0.20	6.98E-01	0.97	0.10	6.08E-03	0.16	0.03	3.82E-01
TMEM176A	No	Yes	No	Up	1.36	0.000982	1.5E-19	0.65	0.11	2.22E-02	0.54	0.04	1.98E-03	0.15	0.10	5.26E-01	0.27	0.11	3.69E-01	0.65	0.14	3.97E-02	0.25	0.20	4.52E-01	0.35	0.10	1.42E-01	0.69	0.03	3.03E-04
PDZD2	No	No	No	Up	1.36	0.013297	validated	0.64	0.11	2.98E-02	0.30	0.04	7.71E-02	0.13	0.10	5.67E-01	0.25	0.11	3.69E-01	1.15	0.16	2.74E-02	0.25	0.20	5.08E-01	0.28	0.10	2.01E-01	0.56	0.03	2.23E-03
PROCR	No	No	No	Up	1.36	0.037911	validated	0.11	0.10	6.57E-01	0.45	0.04	1.12E-02	0.08	0.10	7.32E-01	0.25	0.11	4.06E-01	0.88	0.15	3.67E-02	0.68	0.21	1.53E-01	0.84	0.10	1.99E-02	0.27	0.03	1.89E-01
MAP3K12	No	Yes	No	Up	1.36	0.010383	0.00081	1.11	0.11	2.14E-03	0.66	0.04	2.81E-03	0.37	0.10	2.94E-01	0.44	0.12	1.70E-01	0.07	0.14	8.32E-01	0.05	0.20	8.95E-01	0.46	0.10	2.16E-01	0.40	0.03	3.32E-02
KREMEN2	No	Yes	No	Up	1.36	0.002715	1.5E-19	0.61	0.10	1.71E-02	0.71	0.04	5.86E-05	0.65	0.11	1.73E-01	0.15	0.11	5.33E-01	0.04	0.14	8.10E-01	0.15	0.20	5.41E-01	0.90	0.10	5.60E-02	0.36	0.03	2.81E-02
HRC	No	No	No	Up	1.36	0.002476	validated	0.09	0.10	7.67E-01	0.57	0.04	1.42E-03	0.37	0.10	2.83E-01	0.45	0.12	1.01E-01	0.51	0.14	2.28E-01	0.67	0.21	6.58E-02	0.25	0.10	6.56E-01	0.66	0.03	1.66E-04
RRAS	No	Yes	No	Up	1.36	0.016477	0.00128	0.80	0.11	1.93E-02	0.43	0.04	2.17E-02	0.41	0.10	1.77E-01	0.30	0.11	3.72E-01	0.46	0.14	6.34E-02	0.77	0.21	2.23E-02	0.21	0.10	4.83E-01	0.20	0.03	2.71E-01
SLITRK4	No	No	No	Up	1.36	0.000796	0.04189	0.94	0.11	1.47E-04	0.18	0.04	1.73E-01	0.28	0.10	2.12E-01	0.29	0.11	2.26E-01	0.72	0.14	3.80E-02	0.42	0.20	2.29E-01	0.24	0.10	3.61E-01	0.51	0.03	1.03E-03
CRMP1	No	No	Yes	Up	1.37	0.012249	0.00867	0.10	0.10	7.03E-01	0.22	0.04	1.32E-01	0.17	0.10	6.52E-01	1.20	0.13	6.79E-04	0.14	0.14	4.55E-01	0.66	0.21	4.71E-02	0.68	0.10	8.66E-02	0.42	0.03	3.17E-02
KLF12	No	Yes	No	Up	1.37	0.015448	0.00039	0.45	0.10	7.99E-02	0.63	0.04	1.94E-03	0.56	0.11	3.02E-02	0.47	0.12	9.61E-02	0.28	0.14	4.47E-01	0.96	0.22	1.11E-01	0.07	0.10	7.35E-01	0.16	0.03	2.68E-01
UBE2I	No	No	No	Up	1.37	0.008994	validated	0.15	0.10	6.77E-01	0.36	0.04	1.21E-01	0.12	0.10	6.51E-01	0.14	0.11	7.79E-01	0.75	0.14	8.80E-03	0.93	0.22	9.14E-03	1.04	0.10	7.54E-04	0.12	0.03	4.40E-01
CDC25C	No	Yes	No	Up	1.37	0.00224	5.6E-05	0.80	0.11	9.78E-03	0.87	0.04	3.00E-05	0.04	0.10	8.70E-01	0.86	0.12	1.23E-02	0.04	0.14	8.86E-01	0.45	0.20	3.96E-01	0.32	0.10	2.11E-01	0.22	0.03	1.72E-01
DIP2C	No	No	No	Up	1.37	0.018146	validated	0.12	0.10	7.06E-01	0.23	0.04	2.07E-01	0.54	0.11	7.48E-02	0.32	0.11	2.88E-01	0.88	0.15	2.54E-02	0.28	0.20	3.43E-01	1.04	0.10	1.12E-03	0.20	0.03	2.16E-01
FLNA	No	Yes	No	Up	1.37	0.03528	0.01552	0.96	0.11	4.19E-03	0.04	0.04	8.03E-01	0.38	0.10	1.66E-01	1.07	0.13	2.75E-03	0.63	0.14	1.06E-01	0.36	0.20	4.28E-01	0.14	0.10	6.30E-01	0.03	0.03	8.69E-01
ST8SIA1	No	No	No	Up	1.37	0.002337	validated	0.53	0.10	4.94E-02	0.37	0.04	3.90E-02	0.27	0.10	3.14E-01	0.86	0.12	6.67E-03	0.10	0.14	8.31E-01	0.25	0.20	6.34E-01	0.57	0.10	2.07E-01	0.66	0.03	1.00E-04
MATN3	No	No	No	Up	1.37	0.004642	validated	0.14	0.10	5.68E-01	0.77	0.04	4.16E-04	0.14	0.10	5.52E-01	0.93	0.12	2.86E-03	0.50	0.14	5.40E-01	0.15	0.20	7.00E-01	0.56	0.10	1.24E-01	0.42	0.03	1.31E-02
C1ORF112	No	No	No	Up	1.37	0.028306	0.00435	0.39	0.10	2.44E-01	0.61	0.04	4.46E-03	0.21	0.10	4.31E-01	0.86	0.12	6.22E-02	0.33	0.14	2.88E-01	0.48	0.21	2.56E-01	0.21	0.10	5.76E-01	0.53	0.03	1.71E-02
PRELID2	No	No	No	Up	1.37	0.004419	0.01584	0.37	0.10	1.58E-01	0.95	0.04	2.43E-05	0.34	0.10	3.10E-01	0.63	0.12	6.64E-02	0.08	0.14	9.11E-01	0.03	0.20	9.77E-01	0.61	0.10	1.47E-01	0.61	0.03	1.98E-02
CREB1	No	Yes	No	Up	1.37	0.030435	0.04112	0.64	0.11	4.48E-02	0.26	0.04	1.86E-01	1.03	0.11	1.12E-02	0.89	0.12	1.95E-02	0.30	0.14	1.51E-01	0.07	0.20	6.33E-01	0.17	0.10	4.87E-01	0.27	0.03	3.00E-01
BUB1B	No	Yes	No	Up	1.37	0.009446	0.00023	0.86	0.11	4.73E-03	0.33	0.04	4.47E-02	0.12	0.10	5.83E-01	0.68	0.12	3.71E-02	0.30	0.14	6.15E-01	0.39	0.20	5.70E-01	0.52	0.10	4.20E-02	0.45	0.03	1.87E-02
MMP13	No	No	No	Up	1.37	0.011814	validated	0.40	0.10	1.06E-01	0.20	0.04	1.99E-01	0.07	0.10	8.22E-01	0.96	0.13	1.56E-03	0.62	0.14	2.64E-01	0.54	0.21	3.06E-01	0.39	0.10	3.41E-01	0.47	0.03	2.98E-03
CCNA2	No	No	No	Up	1.37	0.002965	0.00778	0.58	0.10	5.11E-02	0.38	0.04	3.19E-02	0.19	0.10	4.07E-01	0.57	0.12	5.92E-02	0.35	0.14	1.60E-01	0.14	0.20	5.08E-01	0.95	0.10	2.56E-03	0.51	0.03	9.72E-03
FLVCR2	No	No	No	Up	1.37	0.028566	validated	0.12	0.10	6.72E-01	0.01	0.04	9.55E-01	0.08	0.10	7.73E-01	1.42	0.14	3.31E-04	0.38	0.14	4.11E-01	0.25	0.20	5.89E-01	1.01	0.10	2.51E-02	0.39	0.03	2.17E-02
USP44	No	Yes	No	Up	1.37	0.044069	1.5E-19	0.53	0.10	4.99E-02	0.34	0.04	1.10E-01	0.39	0.10	1.12E-01	0.08	0.11	7.40E-01	0.14	0.14	8.51E-01	0.59	0.21	5.41E-01	1.12	0.10	3.97E-02	0.46	0.03	8.45E-03
BRD8	No	Yes	No	Up	1.37	0.022034	0.01174	0.40	0.10	2.63E-01	0.36	0.04	7.59E-02	0.34	0.10	3.51E-01	0.06	0.11	9.04E-01	0.49	0.14	1.53E-01	0.76	0.21	7.61E-02	0.89	0.10	5.60E-03	0.37	0.03	2.63E-02
PRICKLE1	No	No	No	Up	1.37	0.000931	validated	0.63	0.10	1.88E-02	0.14	0.04	3.33E-01	0.42	0.10	7.47E-02	0.89	0.12	3.05E-03	0.43	0.14	8.94E-02	0.19	0.20	3.87E-01	0.20	0.10	3.69E-01	0.77	0.03	3.19E-04
SLC16A1	No	Yes	No	Up	1.37	0.002257	0.00293	0.36	0.10	1.60E-01	0.57	0.04	1.75E-03	0.66	0.11	9.19E-03	0.44	0.12	2.52E-01	0.03	0.14	9.80E-01	0.63	0.21	6.97E-01	0.31	0.10	1.47E-01	0.67	0.03	6.26E-04
BCHE	No	No	No	Up	1.37	0.000803	0.00975	0.43	0.10	9.88E-02	0.18	0.04	1.75E-01	0.01	0.10	9.52E-01	0.45	0.12	7.56E-02	0.69	0.14	4.33E-02	0.44	0.20	1.83E-01	0.82	0.10	6.29E-03	0.65	0.03	6.61E-05
EXO1	No	No	No	Up	1.38	0.002697	0.00858	0.25	0.10	3.71E-01	0.43	0.04	1.23E-02	0.27	0.10	2.82E-01	0.74	0.12	2.34E-02	0.66	0.14	7.53E-02	0.31	0.20	3.28E-01	0.25	0.10	5.18E-01	0.76	0.03	1.62E-04
UBR4	No	No	No	Up	1.38	0.033634	validated	0.23	0.10	6.20E-01	0.48	0.04	7.69E-02	0.34	0.10	3.33E-01	1.14	0.13	2.08E-03	0.50	0.14	9.14E-02	0.11	0.20	6.07E-01	0.74	0.10	5.07E-02	0.14	0.03	3.60E-01
PXDNL	No	No	No	Up	1.38	0.010206	0.00035	0.82	0.11	2.23E-02	0.88	0.04	6.32E-04	0.03	0.10	9.61E-01	0.95	0.13	1.00E-03	0.67	0.14	5.81E-01	0.08	0.20	9.52E-01	0.10	0.10	7.92E-01	0.17	0.03	2.58E-01
INCENP	No	Yes	No	Up	1.38	0.029186	0.00646	0.65	0.11	4.18E-02	0.68	0.04	1.22E-03	0.18	0.10	6.19E-01	0.25	0.11	4.53E-01	0.64	0.14	1.33E-01	0.41	0.20	3.52E-01	0.71	0.10	1.23E-01	0.18	0.03	2.80E-01
FLRT3	No	No	No	Up	1.38	0.001016	5.4E-05	0.67	0.11	5.13E-03	0.22	0.04	1.37E-01	0.14	0.10	5.03E-01	0.80	0.12	7.16E-03	0.29	0.14	2.61E-01	0.52	0.21	9.09E-02	0.66	0.10	6.32E-03	0.42	0.03	1.87E-02
NTRK1	No	No	No	Up	1.38	0.005287	0.00226	0.33	0.10	2.73E-01	1.10	0.04	2.32E-05	0.07	0.10	9.00E-01	0.82	0.12	3.26E-03	0.39	0.14	6.58E-01	0.24	0.20	8.03E-01	0.48	0.10	3.51E-01	0.28	0.03	8.97E-02
TK1	No	Yes	No	Up	1.38	0.003542	0.00068	0.60	0.10	4.02E-02	0.53	0.04	4.37E-03	0.02	0.10	9.36E-01	0.17	0.11	5.89E-01	0.35	0.14	1.62E-01	0.66	0.21	6.63E-02	0.95	0.10	5.23E-03	0.43	0.03	2.22E-02
ASF1B	No	Yes	No	Up	1.38	0.010549	0.00081	0.75	0.11	2.40E-02	0.49	0.04	9.11E-03	0.05	0.10	8.43E-01	0.23	0.11	5.25E-01	0.73	0.14	9.35E-02	0.21	0.20	4.87E-01	0.71	0.10	3.35E-02	0.56	0.03	1.13E-02
TSHR	No	Yes	No	Up	1.38	0.026634	1.5E-19	0.20	0.10	5.21E-01	0.31	0.04	2.17E-01	0.22	0.10	5.69E-01	0.05	0.11	8.85E-01	0.99	0.15	5.13E-01	0.52	0.21	1.75E-01	0.74	0.10	6.71E-02	0.70	0.03	5.25E-05
FBXO5	No	No	No	Up	1.38	0.008025	0.0064	0.30	0.10	2.86E-01	0.36	0.04	5.51E-02	0.24	0.10	3.55E-01	1.19	0.13	1.40E-03	0.31	0.14	2.30E-01	0.43	0.20	1.78E-01	0.43	0.10	9.03E-02	0.46	0.03	2.83E-02
PDZRN4	No	No	No	Up	1.38	0.001481	0.01375	0.49	0.10	1.50E-01	0.45	0.04	2.76E-02	0.22	0.10	4.49E-01	0.74	0.12	9.42E-03	0.47	0.14	4.44E-01	0.25	0.20	6.47E-01	0.29	0.10	6.09E-01	0.83	0.03	5.23E-06
SLC25A43	No	No	No	Up	1.38	0.008062	0.00281	0.68	0.11	3.70E-02	0.57	0.04	6.26E-03	0.05	0.10	8.31E-01	0.60	0.12	6.21E-02	0.59	0.14	5.59E-02	0.42	0.20	1.74E-01	0.52	0.10	4.99E-02	0.30	0.03	1.43E-01
SUPT3H	No	No	No	Up	1.39	0.040993	validated	0.34	0.10	2.53E-01	0.62	0.04	3.14E-02	0.78	0.11	2.95E-02	0.50	0.12	1.13E-01	0.03	0.14	9.01E-01	0.70	0.21	1.97E-01	0.44	0.10	1.34E-01	0.35	0.03	9.19E-02
MCL1	No	No	No	Up	1.39	0.03166	validated	1.06	0.11	4.82E-03	0.10	0.04	5.86E-01	0.42	0.10	1.07E-01	0.41	0.12	2.68E-01	1.12	0.16	8.21E-03	0.41	0.20	1.65E-01	0.20	0.10	4.08E-01	0.08	0.03	6.33E-01
PAPOLG	No	No	No	Up	1.39	0.017222	0.01974	0.61	0.10	5.84E-02	0.84	0.04	2.77E-03	0.57	0.11	7.19E-02	0.12	0.11	7.31E-01	0.07	0.14	9.07E-01	0.35	0.20	6.00E-01	0.93	0.10	1.08E-02	0.29	0.03	1.16E-01
TMEM38B	No	No	No	Up	1.39	0.005154	validated	0.40	0.10	1.18E-01	0.31	0.04	6.76E-02	0.65	0.11	1.89E-02	0.88	0.12	4.88E-03	0.46	0.14	1.82E-01	0.34	0.20	4.02E-01	0.26	0.10	3.01E-01	0.49	0.03	1.81E-02
KCTD17	No	No	No	Up	1.39	0.005657	0.00488	0.37	0.10	2.44E-01	0.54	0.04	1.79E-02	0.74	0.11	1.23E-01	0.49	0.12	1.88E-01	0.07	0.14	8.90E-01	0.15	0.20	7.73E-01	0.63	0.10	1.73E-01	0.80	0.03	3.04E-05
CDKN1C	No	Yes	No	Up	1.39	0.005495	0.00666	0.22	0.10	3.47E-01	0.35	0.04	2.48E-02	0.50	0.10	5.06E-02	0.65	0.12	2.64E-02	0.78	0.15	2.33E-02	0.85	0.22	3.36E-02	0.10	0.10	6.13E-01	0.33	0.03	6.15E-02
ZNF529	No	No	No	Up	1.39	0.024031	0.01603	0.87	0.11	1.43E-02	0.35	0.04	1.21E-01	0.16	0.10	5.30E-01	0.64	0.12	7.96E-02	0.38	0.14	1.95E-01	0.62	0.21	1.27E-01	0.56	0.10	4.03E-02	0.22	0.03	1.86E-01
NFIL3	No	Yes	No	Up	1.39	0.009745	0.00207	0.83	0.11	1.23E-02	0.31	0.04	7.11E-02	0.60	0.11	3.73E-02	1.07	0.13	4.96E-03	0.06	0.14	7.10E-01	0.32	0.20	4.40E-01	0.54	0.10	3.70E-02	0.07	0.03	7.31E-01
STK17B	No	No	No	Up	1.39	0.035507	0.04816	0.59	0.10	5.72E-02	0.18	0.04	3.10E-01	0.22	0.10	3.60E-01	0.47	0.12	1.59E-01	0.46	0.14	3.09E-01	0.56	0.21	3.07E-01	1.14	0.10	1.94E-03	0.18	0.03	2.09E-01

Supplementary Table 2. Details for 2,053 genes differentially expressed between EEC and NEEC, identified by microarray meta-analysis.

EEC - endometrioid endometrial cancer, NEEC - non-endometrioid endometrial cancer, SMD - Standardised Mean Difference, FDR - False Discovery Rate, Var - Variance

Gene	145 gene list	1253 gene list	Associated with EEC-specific survival?	Up/Down Regulated in NEEC	average standardised fold change	Microarray Meta FDR	RNASeq FDR	Individual microarray study results																							
								TCGA			E-MTAB-2532			E-GEOD-56026			GSE32507			GSE24537			E-GEOD-23518			E-GEOD-17025			E-GEOD-2109		
								SMD	Var	P-value	SMD	Var	P-value	SMD	Var	P-value	SMD	Var	P-value	SMD	Var	P-value	SMD	Var	P-value	SMD	Var	P-value	SMD	Var	P-value
RTP3	No	No	No	Up	1.39	0.027491	validated	0.94	0.11	1.52E-03	0.01	0.04	9.46E-01	0.28	0.10	5.75E-01	0.36	0.11	1.75E-01	0.58	0.14	1.03E-01	0.96	0.22	4.87E-01	0.22	0.10	6.45E-01	0.47	0.03	4.19E-03
FKRP	No	No	No	Up	1.39	0.046232	validated	0.22	0.10	5.26E-01	0.82	0.04	3.20E-03	0.30	0.10	4.28E-01	0.28	0.11	5.54E-01	0.44	0.14	2.33E-01	0.63	0.21	1.24E-01	0.92	0.10	3.56E-02	0.21	0.03	1.97E-01
CHST8	No	No	No	Up	1.39	0.046229	0.00019	0.08	0.10	7.72E-01	0.46	0.04	7.12E-03	0.09	0.10	8.40E-01	0.93	0.12	2.44E-03	0.51	0.14	6.97E-01	0.72	0.21	6.81E-01	0.83	0.10	7.09E-02	0.19	0.03	2.14E-01
KCNK2	No	Yes	Yes	Up	1.39	0.025123	1.5E-19	0.74	0.11	2.07E-02	0.26	0.04	3.50E-01	0.60	0.11	2.06E-01	0.67	0.12	4.92E-02	0.23	0.14	7.25E-01	0.67	0.21	2.64E-01	0.10	0.10	8.72E-01	0.54	0.03	1.25E-03
KIF18A	No	No	No	Up	1.39	0.007853	0.00071	0.87	0.11	4.67E-03	0.44	0.04	1.75E-02	0.30	0.10	2.11E-01	0.74	0.12	2.20E-02	0.29	0.14	5.25E-01	0.49	0.21	3.37E-01	0.24	0.10	3.59E-01	0.46	0.03	3.20E-02
RAB3IL1	No	No	No	Up	1.39	0.019674	0.0086	0.47	0.10	1.21E-01	0.01	0.04	9.52E-01	0.19	0.10	6.07E-01	0.62	0.12	6.02E-02	1.14	0.16	7.75E-04	0.97	0.22	9.21E-03	0.34	0.10	4.08E-01	0.09	0.03	6.74E-01
CLSTN3	No	Yes	No	Up	1.39	0.028034	0.00329	0.70	0.11	1.80E-02	0.43	0.04	4.83E-02	0.89	0.11	2.56E-02	0.09	0.11	8.20E-01	0.27	0.14	4.18E-01	0.23	0.20	4.81E-01	0.80	0.10	6.42E-02	0.42	0.03	8.74E-02
PTP4A2	No	Yes	No	Up	1.39	0.018032	0.00509	1.41	0.12	1.41E-03	0.03	0.04	8.31E-01	0.27	0.10	4.22E-01	0.82	0.12	3.36E-02	0.85	0.15	3.04E-03	0.17	0.20	4.09E-01	0.21	0.10	4.15E-01	0.07	0.03	7.60E-01
TMEM147	No	Yes	No	Up	1.39	0.022841	0.01813	1.26	0.12	2.91E-03	0.39	0.04	8.15E-02	0.21	0.10	5.44E-01	0.22	0.11	6.04E-01	0.39	0.14	9.30E-02	0.63	0.21	3.95E-02	0.47	0.10	1.20E-01	0.28	0.03	3.44E-01
TBC1D13	No	No	No	Up	1.40	0.03159	validated	0.72	0.11	4.39E-02	0.45	0.04	6.12E-02	0.16	0.10	6.61E-01	0.45	0.12	3.37E-01	0.59	0.14	1.18E-01	0.28	0.20	3.91E-01	0.73	0.10	1.09E-01	0.45	0.03	9.38E-03
RGS9	No	Yes	No	Up	1.40	0.033048	1.5E-19	0.78	0.11	6.74E-03	0.49	0.04	1.54E-02	0.55	0.11	2.99E-01	0.35	0.11	1.54E-01	0.29	0.14	8.58E-01	0.40	0.20	7.45E-01	0.68	0.10	3.07E-01	0.33	0.03	3.38E-02
PRUNE	No	Yes	No	Up	1.40	0.046377	0.02585	0.63	0.10	6.05E-02	0.12	0.04	5.55E-01	0.29	0.10	3.63E-01	0.52	0.12	1.60E-01	1.07	0.15	2.05E-02	0.46	0.21	1.33E-01	0.56	0.10	6.29E-02	0.22	0.03	2.43E-01
TNNI3	No	Yes	No	Up	1.40	3.97E-05	1.5E-19	0.81	0.11	2.94E-03	0.95	0.04	6.82E-07	0.39	0.10	1.10E-01	0.30	0.11	2.24E-01	0.39	0.14	3.30E-01	0.20	0.20	5.50E-01	0.25	0.10	3.73E-01	0.57	0.03	2.98E-03
EPHA4	No	No	No	Up	1.40	0.00168	0.00558	0.68	0.11	6.75E-03	0.08	0.04	5.63E-01	0.43	0.10	6.10E-02	1.01	0.13	1.50E-03	0.86	0.15	4.06E-02	0.40	0.20	2.64E-01	0.01	0.10	9.67E-01	0.41	0.03	5.84E-03
CYFIP2	No	Yes	Yes	Up	1.40	0.000754	0.00021	0.51	0.10	6.44E-02	0.75	0.04	1.89E-04	0.52	0.11	5.16E-02	0.33	0.11	2.95E-01	0.79	0.15	1.15E-02	0.06	0.20	7.39E-01	0.54	0.10	4.11E-02	0.38	0.03	5.24E-02
TLE4	No	No	No	Up	1.40	0.003843	0.00092	0.37	0.10	1.90E-01	0.38	0.04	1.78E-02	0.44	0.10	7.95E-02	0.82	0.12	1.04E-02	0.85	0.15	1.46E-02	0.46	0.21	2.04E-01	0.26	0.10	2.55E-01	0.30	0.03	6.94E-02
PROK2	No	No	No	Up	1.40	0.000148	0.00138	0.48	0.10	5.58E-02	0.91	0.04	2.50E-06	0.26	0.10	2.56E-01	0.36	0.11	1.19E-01	0.04	0.14	7.24E-01	0.61	0.21	6.00E-02	0.98	0.10	8.09E-03	0.24	0.03	7.86E-02
WDR5	No	No	No	Up	1.40	0.008703	validated	0.86	0.11	1.37E-02	0.65	0.04	6.25E-03	0.52	0.11	9.75E-02	0.71	0.12	9.19E-02	0.13	0.14	5.09E-01	0.09	0.20	6.96E-01	0.44	0.10	1.76E-01	0.52	0.03	2.12E-02
SERPING1	No	Yes	No	Up	1.40	0.002944	0.00103	0.57	0.10	4.70E-02	0.18	0.04	2.18E-01	0.15	0.10	5.34E-01	0.20	0.11	4.43E-01	1.45	0.17	7.22E-05	0.70	0.21	3.04E-02	0.47	0.10	5.47E-02	0.20	0.03	2.66E-01
GALT	No	Yes	No	Up	1.41	0.037595	0.01065	0.51	0.10	1.67E-01	0.36	0.04	9.57E-02	0.23	0.10	5.35E-01	0.20	0.11	6.60E-01	1.55	0.17	1.13E-03	0.42	0.20	2.13E-01	0.43	0.10	2.30E-01	0.24	0.03	1.45E-01
PLSCR4	No	No	No	Up	1.41	0.003764	0.00482	0.39	0.10	1.46E-01	0.12	0.04	4.05E-01	0.11	0.10	6.09E-01	0.92	0.12	2.32E-03	1.14	0.16	2.08E-03	0.49	0.21	1.28E-01	0.30	0.10	2.15E-01	0.46	0.03	2.94E-02
NMT2	No	Yes	No	Up	1.41	0.009399	0.00189	0.46	0.10	1.20E-01	0.29	0.04	1.16E-01	0.67	0.11	2.19E-02	1.10	0.13	3.24E-03	0.39	0.14	1.97E-01	0.30	0.20	3.63E-01	0.43	0.10	1.02E-01	0.31	0.03	1.73E-01
ACVR2A	No	No	No	Up	1.41	0.008008	0.04867	0.77	0.11	8.35E-03	0.42	0.04	3.46E-02	0.26	0.10	3.70E-01	0.84	0.12	1.55E-02	0.18	0.14	6.22E-01	0.41	0.20	4.42E-01	0.37	0.10	2.43E-01	0.69	0.03	7.35E-03
DISP2	No	No	No	Up	1.41	0.003756	validated	0.08	0.10	7.60E-01	0.75	0.04	1.79E-04	0.48	0.10	2.07E-01	0.84	0.12	6.56E-03	0.61	0.14	4.46E-01	0.65	0.21	4.40E-01	0.07	0.10	8.80E-01	0.47	0.03	4.38E-03
SYT12	No	Yes	No	Up	1.41	0.017235	6.7E-06	1.14	0.11	4.77E-03	0.46	0.04	6.39E-03	0.65	0.11	6.46E-02	0.43	0.12	8.14E-02	0.46	0.14	4.57E-01	0.33	0.20	5.65E-01	0.35	0.10	3.55E-01	0.14	0.03	3.95E-01
MXRA5	No	Yes	No	Up	1.41	0.000415	0.00107	0.67	0.11	9.00E-03	0.43	0.04	1.08E-02	0.02	0.10	9.36E-01	1.25	0.13	2.84E-04	0.33	0.14	1.43E-01	0.65	0.21	3.58E-02	0.14	0.10	5.06E-01	0.46	0.03	1.34E-02
HOXD13	No	No	No	Up	1.41	0.001088	validated	0.20	0.10	4.31E-01	0.79	0.04	4.47E-03	0.18	0.10	6.99E-01	0.30	0.11	2.90E-01	0.24	0.14	5.83E-01	0.65	0.21	2.24E-01	0.79	0.10	5.09E-02	0.81	0.03	3.18E-06
SIVA1	No	No	No	Up	1.41	0.037667	validated	0.34	0.10	3.77E-01	0.33	0.04	1.51E-01	0.77	0.11	3.53E-02	0.66	0.12	1.34E-01	0.52	0.14	6.85E-02	0.73	0.21	3.51E-02	0.50	0.10	1.16E-01	0.10	0.03	6.11E-01
NIPBL	No	Yes	No	Up	1.41	0.002278	0.01959	0.94	0.11	1.69E-02	0.63	0.04	5.76E-04	0.49	0.10	1.13E-01	0.07	0.11	8.21E-01	0.35	0.14	2.15E-01	0.67	0.21	8.16E-02	0.65	0.10	5.79E-03	0.16	0.03	4.58E-01
RPA2	No	No	No	Up	1.41	0.040057	validated	0.04	0.10	9.11E-01	0.41	0.04	6.66E-02	0.56	0.11	1.13E-01	1.03	0.13	4.68E-02	0.42	0.14	1.16E-01	0.38	0.20	1.83E-01	0.31	0.10	2.49E-01	0.81	0.03	3.19E-02
ACACB	No	Yes	No	Up	1.41	0.002071	0.00012	0.96	0.11	4.04E-03	0.48	0.04	3.84E-02	0.32	0.10	2.05E-01	0.69	0.12	2.50E-02	0.70	0.14	1.56E-02	0.09	0.20	6.10E-01	0.35	0.10	1.32E-01	0.36	0.03	3.25E-02
RHCE	No	No	No	Up	1.41	0.011716	0.03619	0.72	0.11	1.67E-02	0.19	0.04	3.17E-01	0.03	0.10	9.42E-01	0.67	0.12	2.42E-02	0.18	0.14	8.29E-01	0.46	0.21	6.66E-01	1.15	0.10	2.34E-02	0.57	0.03	1.41E-03
LYAR	No	No	No	Up	1.41	0.004836	0.00527	0.69	0.11	7.18E-02	0.82	0.04	5.80E-04	0.62	0.11	4.83E-02	0.20	0.11	6.30E-01	0.62	0.14	6.56E-02	0.00	0.20	9.80E-01	0.37	0.10	1.86E-01	0.64	0.03	1.66E-02
PER3	No	Yes	No	Up	1.41	0.011403	9.5E-05	0.29	0.10	3.36E-01	0.05	0.04	7.23E-01	1.32	0.12	1.57E-04	0.27	0.11	3.25E-01	0.80	0.15	4.90E-02	0.60	0.21	1.66E-01	0.56	0.10	3.53E-02	0.08	0.03	5.77E-01
TIMM8A	No	No	Yes	Up	1.41	0.008244	0.00552	1.31	0.12	1.36E-03	0.96	0.04	1.45E-04	0.45	0.10	1.70E-01	0.24	0.11	5.43E-01	0.09	0.14	7.92E-01	0.53	0.21	4.26E-01	0.26	0.10	2.88E-01	0.13	0.03	5.03E-01
SLC45A4	No	Yes	No	Up	1.41	0.004775	2.6E-05	1.33	0.12	1.11E-04	0.05	0.04	7.25E-01	0.43	0.10	1.66E-01	0.41	0.12	7.73E-02	0.43	0.14	2.04E-01	0.24	0.20	4.60E-01	1.04	0.10	2.90E-03	0.04	0.03	8.69E-01
STAMBP	No	No	No	Up	1.41	0.035594	0.03249	0.52	0.10	1.87E-01	0.33	0.04	1.61E-01	0.59	0.11	8.60E-02	0.76	0.12	1.17E-01	0.16	0.14	4.13E-01	0.70	0.21	5.06E-02	0.31	0.10	3.09E-01	0.60	0.03	2.00E-02
TCEB1	No	No	No	Up	1.41	0.014023	validated	1.28	0.12	4.63E-03	0.31	0.04	1.04E-01	0.41	0.10	2.27E-01	0.92	0.12	6.57E-03	0.15	0.14	3.87E-01	0.35	0.20	1.89E-01	0.31	0.10	2.20E-01	0.26	0.03	2.96E-01
SLC23A2	No	No	No	Up	1.41	0.027401	0.0314	0.32	0.10	5.30E-01	0.36	0.04	6.21E-02	0.96	0.11	5.63E-03	0.28	0.11	3.50E-01	0.86	0.15	5.69E-02	0.69	0.21	1.51E-01	0.17	0.10	5.75E-01	0.34	0.03	6.04E-02
PARL	No	Yes	Yes	Up	1.41	0.016848	0.00076	0.80	0.11	1.90E-02	0.21	0.04	1.98E-01	1.16	0.11	1.16E-02	0.31	0.11	3.22E-01	0.69	0.14	1.74E-02	0.22	0.20	3.35E-01	0.35	0.10	3.19E-01	0.24	0.03	2.07E-01
SUPT16H	No	No	No	Up	1.41	0.009565	validated	0.23	0.10	6.01E-01	0.28	0.04	9.44E-02	0.66	0.11	2.39E-02	0.50	0.12	1.95E-01	0.88	0.15	7.28E-03	1.21	0.24	2.84E-03	0.09	0.10	6.42E-01	0.14	0.03	3.73E-01
LAMC3	No	No	No	Up	1.41	0.001921	0.00201	0.44	0.10	8.08E-02	0.56	0.04	1.05E-03	0.87	0.11	8.25E-03	0.39	0.12	1.94E-01	0.05	0.14	7.27E-01	0.87	0.22	2.60E-02	0.67	0.10	2.64E-02	0.14	0.03	4.01E-01
CCDC42	No	No	No	Up	1.41	0.032079	1.5E-19	1.41	0.12	7.77E-04	0.60	0.04	2.89E-02	0.13	0.10	8.01E-01	0.83	0.12	1.16E-02	0.32	0.14	8.48E-01	0.23	0.20	8.88E-01	0.35	0.10	4.89E-01	0.13	0.03	3.85E-01
UBE2L6	No	Yes	No	Up	1.41	0.0029	0.00046	1.09	0.11	1.10E-03	0.12	0.04	4.28E-01	0.61	0.11	2.92E-02	0.71	0.12	3.15E-02	0.63	0.14	1.73E-02	0.46	0.21	9.90E-02	0.03	0.10	8.74E-01	0.35	0.03	1.14E-01
ANGPT1	No	No	No	Up	1.42	0.000875	validated	0.19	0.10	5.28E-01	0.47	0.04	1.29E-02	0.16	0.10	4.80E-01	1.12	0.13	1.73E-04	0.54	0.14	1.04E-01	0.43	0.20	1.94E-01	0.79	0.10	8.89E-03	0.31	0.03	4.82E-02
CAMK1D	No	No	No	Up	1.42	0.00034	validated	0.37	0.10	1.53E-01	0.69	0.04	5.01E-04	0.96	0.11	2.40E-03	0.31	0.11	2.64E-01	0.61	0.14	1.31E-01	0.40	0.20	2.92E-01	0.04	0.10	8.81E-01	0.65	0.03	3.71E-04
GTSE1	No	No	No	Up	1.42	0.00388	0.01926	0.49	0.10	9.63E-02	0.35	0.04	3.92E-02	0.33	0.10	1.66E-01	0.59	0.12	5.78E-02	0.80	0.15	6.43E-02	0.12	0.20	6.31E-01	1.14	0.10	5.13E-04	0.21	0.03	2.00E-01
SNX8	No	No	No	Up	1.42	0.04441	validated	0.32	0.10	2.83E-01	0.31	0.04	1.27E-01	0.52	0.11	1.35E-01	0.43	0.12	2.81E-01	1.40	0.17	3.98E-03	0.56	0.21	1.61E-01	0.33	0.10	2.94E-01	0.16	0.03	2.82E-01
NR0B1	No	Yes	No	Up	1.42	0.005098	1.5E-19	1.43	0.12	2.95E-06	0.30	0.04	7.66E-02	0.50	0.10	2.65E-01	0.07	0.11	7.48E-01	0.14	0.14	7.39E-01	1.01	0.23	2.85E-01	0.47	0.10	7.60E-02	0.12	0.03	3.98E-01
CACNA2D3	No	Yes	No	Up	1.42	0.000304	1.5E-19	0.50	0.10	5.09E-02	0.40	0.04	2.43E-02	0.36	0.10	1.53E-01	0.52	0.12	5.56E-02	0.14	0.14	6.58E-01	0.50	0.21	2.90E-01	0.94	0.10	2.14E-03	0.65	0.03	1.10E-04
ALS2CR11	No	Yes	No	Up	1.42	0.004885	1.5E-19	0.94	0.11	2.16E-03	0.90	0.04	2.74E-03	0.49	0.10	2.06E-01	0.18	0.11	4.55E-01	0.42	0.14	7.56E-01	0.24	0.20	8.53E-01	0.37	0.10	4.59E-01	0.50	0.03	1.59E-03
PAPLN	No	Yes	No	Up	1.42	0.002784	1.5E-19	0.99	0.11	8.31E-03	0.28	0.04	6.51E-02	0.58	0.11	2.09E-02	0.67	0.12	3.84E-02	0.44	0.14	1.77E-01	0.18	0.20	6.42E-01	0.74	0.10	4.47E-03	0.16	0.03	3.06E-01
CDH15	No	No	No	Up	1.42	0.005314	validated	0.12	0.10	6.40E-01	0.51	0.04	2.42E-03	0.10	0.10	8.15E-01	0.65	0.12	9.93E-03	0.26	0.14	5.08E-01	1.14	0.23	1.41E-01	0.77	0.10	7.49E-02	0.50	0.03	4.65E-03
BCL2L12	No	Yes	No	Up	1.42	0.006007	0.00511	0.46	0.10	1.95E-01	1.03	0.04	1.33E-04	0.24	0.10	4.04E-01	0.69	0.12	1.24E-01	0.14	0.14	4.77E-01	0.41	0.20	1.88E-01	0.49	0.10	1.68E-01	0.59	0.03	2.15E-02
HESX1	No	No	No	Up	1.42	0.04583	validated	0.42	0.10	1.41E-01	0.56	0.04	4.23E-02	0.90	0.11	4.29E-02	0.87	0.12	5.07E-02	0.48	0.14	4.99E-01	0.42	0.20	5.67E-01	0.01	0.10	9.78E-01	0.39	0.03	2.21E-02
ZCCHC11	No	No	No	Up	1.42	0.015707	validated	0.32	0.10	2.92E-01	0.55	0.04	2.04E-02	0.78	0.11	1.54E-02	0.10	0.11	8.24E-01	0.73	0.14	6.10E-02	0.87	0.22	8.88E-02	0.33	0.10	2.56E-01	0.36	0.03	4.38E-02
CCDC59	No	No	No	Up	1.42	0.010314	0.03803	0.56	0.10	1.47E-01	0.71	0.04	1.92E-03	0.37	0.10	3.18E-01	0.28	0.11	5.15E-01	0.75	0.14	1.74E-02	0.39	0.20	1.81E-01	0.75	0.10	2.83E-02	0.24	0.03	3.82E-01
CA11	No	No	No	Up	1.42	0.007002	0.03828	0.75	0.11	1.91E-02	0.20	0.04	2.05E-01	0.26	0.10	3.18E-01	0.68	0.12	3.82E-02	0.93	0.15	6.19E-03	0.63	0.21	6.75E-02	0.27	0.10	4.19E-01	0.34	0.03	7.19E-02
SCARA5	No	No	No	Up	1.42	0.001094	validated	0.23	0.10	5.24E-01	0.49	0.04	5.07E-03	0.21	0.10	4.25E-01	0.61	0.12	1.59E-02	0.64	0.14	6.93E-02	0.99	0.22	1.71E-01	0.28	0.10	4.85E-01	0.62	0.03	8.13E-05
IFI44	No	Yes	No	Up	1.42	1.66E-05	1.9E-05	0.82	0.11	1.64E-03	0.80	0.04	2.43E-05	0.81	0.11	2.83E-03	0.16	0.11	5.20E-01	0.68	0.14	1.51E-02	0.22	0.20	3.31E-01	0.12	0.10	5.46E-01	0.46	0.03	1.35E-02
ENO3	No	No	No	Up	1.42	0.00801	validated	0.25	0.10	4.47E-01	0.46	0.04	7.14E-03	0.80	0.11	5.56E-02	0.57	0.12	5.19E-02	0.17	0.14	4.60E-01	0.93	0.22	3.96E-02	0.56	0.10	9.27E-02	0.32	0.03	5.25E-02
STK35	No	Yes	No	Up	1.42	0.043669	0.00199	1.09	0.11	2.50E-02	0.09	0.04	6.35E-01	1.32	0.12	2.14E-03	0.84	0.12	2.88E-02	0.17	0.14	5.14E-01	0.53	0.21	1.65E-01	0.01	0.10	9.68E-01	0.03	0.03	8.53E-01
ZNF251	No	Yes	No	Up	1.42	0.004823	0.00345	1.00	0.11	3.65E-03	0.75	0.04	7.46E-04	0.58	0.11	8.87E-02	0.63	0.12	1.36E-01	0.32	0.14	4.74E-01	0.04	0.20	9.27E-01	0.16	0.10	5.67E-01	0.60	0.03	3.08E-02
C11ORF41	No	No	No	Up	1.42	0.001474	3.7E-05	0.33	0.10	1.77E-01	0.64	0.04	2.61E-04	0.41	0.10	1.56E-01	0.77	0.12	3.95E-03	0.13	0.14	6.72E-01	0.79	0.22	2.00E-01	0.66	0.10	1.08E-01	0.35	0.03	3.83E-02
ADAM33	No	Yes	No	Up	1.42	0.033736	1.5E-19	0.39	0.10	3.07E-01	0.14	0.04	3.12E-01	0.46	0.10	1.06E-01	0.42	0.12	1.77E-01	0.97	0.15	2.39E-01	0.81	0.22	3.87E-01	0.37	0.10	2.18E-01	0.51	0.03	9.30E-04
ZNF643	No	No	No	Up	1.42	0.010671	validated	0.85	0.11	1.39E-02	0.58	0.04	1.07E-02	0.95	0.11	3.71E-03	0.06	0.11	8.58E-01	0.15	0.14	8.02E-01	0.96	0.22	3.27E-01	0.03	0.10	9.29E-01	0.50	0.03	1.49E-02
PDGFRL	No	No	No	Up	1.42	6.88E-05	validated	0.37	0.10	1.33E-01	0.27	0.04	6.77E-02	0.19	0.10	4.02E-01	0.30	0.11	2.34E-01	0.97	0.15	2.14E-03	0.81	0.22	1.70E-02	0.34	0.10	1.77E-01	0.82	0.03	5.23E-06
PRIM1	No	No	No	Up	1.42	0.005597	validated	0.40	0.10	1.97E-01	0.39	0.04	3.56E-02	0.49	0.10	8.76E-02	1.14	0.13	4.38E-03	0.26	0.14	3.05E-01	0.69	0.21	8.81E-02	0.02	0.10	9.42E-01	0.70	0.03	5.21E-03
SOCS3	No	Yes	No	Up	1.42	0.00017	2E-05	0.80	0.11	9.19E-03	0.59	0.04	7.92E-04	0.63	0.11	1.51E-02	0.04	0.11	8.88E-01	0.59	0.14	9.25E-02	0.15	0.20	5.22E-01	1.18	0.10	6.57E-05	0.09	0.03	5.38E-01

Supplementary Table 2. Details for 2,053 genes differentially expressed between EEC and NEEC, identified by microarray meta-analysis.

EEC - endometrioid endometrial cancer, NEEC - non-endometrioid endometrial cancer, SMD - Standardised Mean Difference, FDR - False Discovery Rate, Var - Variance

Gene	145 gene list	1253 gene list	Associated with EEC-specific survival?	Up/Down Regulated in NEEC	average standardised fold change	Microarray Meta FDR	RNASeq FDR	Individual microarray study results																							
								TCGA			E-MTAB-2532			E-GEOD-56026			GSE32507			GSE24537			E-GEOD-23518			E-GEOD-17025			E-GEOD-2109		
								SMD	Var	P-value	SMD	Var	P-value	SMD	Var	P-value	SMD	Var	P-value	SMD	Var	P-value	SMD	Var	P-value	SMD	Var	P-value	SMD	Var	P-value
CCK	No	Yes	No	Up	1.42	0.001209	1.5E-19	0.79	0.11	2.51E-03	0.80	0.04	2.66E-04	0.56	0.11	2.79E-01	0.69	0.12	6.85E-03	0.25	0.14	5.65E-01	0.40	0.20	7.32E-01	0.26	0.10	6.43E-01	0.34	0.03	3.14E-02
SOHLH1	No	Yes	No	Up	1.42	0.00072	1.5E-19	0.76	0.11	7.19E-03	0.78	0.04	1.11E-04	0.50	0.10	3.43E-01	0.22	0.11	3.42E-01	0.03	0.14	9.79E-01	0.27	0.20	8.43E-01	0.95	0.10	2.76E-02	0.57	0.03	1.41E-03
C21ORF91	No	No	No	Up	1.42	0.020762	validated	1.46	0.12	3.67E-04	0.20	0.04	3.62E-01	0.59	0.11	4.65E-02	0.95	0.12	3.01E-02	0.23	0.14	5.97E-01	0.17	0.20	7.12E-01	0.36	0.10	2.05E-01	0.11	0.03	5.82E-01
NEIL3	No	No	No	Up	1.42	0.008869	0.00062	0.06	0.10	8.32E-01	0.67	0.04	4.96E-03	0.25	0.10	3.29E-01	0.68	0.12	3.12E-02	0.71	0.14	1.87E-01	0.47	0.21	3.57E-01	0.61	0.10	4.52E-02	0.65	0.03	8.34E-03
AQP11	No	No	No	Up	1.43	0.047412	0.02418	0.62	0.10	5.78E-02	0.38	0.04	1.72E-01	0.54	0.11	1.70E-01	0.84	0.12	1.68E-02	0.40	0.14	3.59E-01	0.60	0.21	2.95E-01	0.49	0.10	1.58E-01	0.23	0.03	1.85E-01
CDK2	No	Yes	No	Up	1.43	0.018986	0.01499	0.82	0.11	1.47E-02	0.44	0.04	5.02E-02	0.71	0.11	2.82E-02	1.20	0.13	1.84E-02	0.15	0.14	4.33E-01	0.60	0.21	8.83E-02	0.14	0.10	5.83E-01	0.02	0.03	8.73E-01
ATP1B2	No	No	No	Up	1.43	0.021107	validated	0.72	0.11	9.19E-02	0.37	0.04	1.95E-02	0.29	0.10	3.73E-01	0.58	0.12	3.73E-02	0.95	0.15	3.66E-02	0.23	0.20	4.94E-01	0.75	0.10	8.35E-02	0.20	0.03	2.62E-01
SLIT3	No	No	No	Up	1.43	0.001712	validated	0.30	0.10	2.23E-01	0.19	0.04	1.82E-01	0.10	0.10	6.94E-01	0.27	0.11	2.73E-01	1.51	0.17	2.35E-04	0.74	0.21	3.59E-02	0.41	0.10	2.97E-01	0.58	0.03	1.12E-03
F7	No	No	No	Up	1.43	0.008568	validated	0.79	0.11	2.66E-02	0.81	0.04	1.39E-04	0.37	0.10	3.68E-01	0.83	0.12	8.50E-03	0.04	0.14	9.72E-01	0.74	0.21	5.59E-01	0.45	0.10	2.58E-01	0.08	0.03	6.03E-01
EPYC	No	No	No	Up	1.43	0.001953	validated	0.86	0.11	1.52E-02	0.64	0.04	1.88E-02	0.57	0.11	1.33E-02	0.24	0.11	4.55E-01	0.72	0.14	1.86E-01	0.41	0.20	1.62E-01	0.17	0.10	6.55E-01	0.49	0.03	8.22E-04
SRM	No	No	No	Up	1.43	0.006602	validated	0.33	0.10	3.67E-01	0.52	0.04	1.60E-02	0.26	0.10	4.10E-01	0.92	0.12	2.61E-02	0.70	0.14	1.79E-02	0.04	0.20	8.17E-01	0.70	0.10	3.16E-02	0.64	0.03	1.80E-02
ECH1	No	No	No	Up	1.43	0.011776	validated	0.42	0.10	3.16E-01	0.06	0.04	7.04E-01	0.05	0.10	8.68E-01	1.24	0.13	4.63E-03	1.10	0.15	6.93E-04	0.66	0.21	3.72E-02	0.30	0.10	2.93E-01	0.28	0.03	3.26E-01
ST3GAL5	No	No	No	Up	1.43	0.010936	validated	0.10	0.10	6.98E-01	0.23	0.04	1.99E-01	0.62	0.11	2.79E-02	0.42	0.12	1.58E-01	0.92	0.15	9.51E-03	1.36	0.25	4.94E-03	0.14	0.10	5.44E-01	0.32	0.03	1.19E-01
DBN1	No	Yes	No	Up	1.43	0.004883	0.0278	0.10	0.10	7.53E-01	0.79	0.04	5.48E-04	0.45	0.10	1.82E-01	1.13	0.13	2.02E-02	0.47	0.14	5.86E-02	0.59	0.21	5.94E-02	0.28	0.10	3.49E-01	0.32	0.03	1.40E-01
LPIN2	No	Yes	No	Up	1.43	0.016965	0.00063	0.83	0.11	4.87E-02	0.56	0.04	1.53E-02	0.63	0.11	3.36E-02	0.06	0.11	8.44E-01	0.55	0.14	1.21E-01	0.42	0.20	2.88E-01	0.49	0.10	1.25E-01	0.57	0.03	6.04E-02
DGUOK	No	No	No	Up	1.43	0.009012	validated	1.04	0.11	2.88E-02	0.68	0.04	6.38E-03	0.37	0.10	2.63E-01	0.57	0.12	3.12E-01	0.01	0.14	9.04E-01	0.87	0.22	1.06E-02	0.10	0.10	7.04E-01	0.49	0.03	1.09E-02
TACC3	No	Yes	No	Up	1.43	0.000889	3E-05	0.78	0.11	8.64E-03	0.91	0.04	2.20E-05	0.40	0.10	1.54E-01	0.44	0.12	1.90E-01	0.63	0.14	1.52E-01	0.54	0.21	2.07E-01	0.07	0.10	7.88E-01	0.36	0.03	3.72E-02
EHD3	No	Yes	No	Up	1.43	0.028751	0.00182	0.91	0.11	9.05E-03	0.26	0.04	1.66E-01	0.34	0.10	2.13E-01	0.19	0.11	5.36E-01	0.38	0.14	6.73E-01	1.00	0.22	4.13E-01	0.66	0.10	2.96E-02	0.40	0.03	1.56E-02
OLFM2	No	Yes	No	Up	1.43	0.008773	1.5E-19	0.16	0.10	6.59E-01	0.93	0.04	1.31E-04	0.13	0.10	7.78E-01	0.45	0.12	1.52E-01	0.43	0.14	2.11E-01	0.40	0.20	2.58E-01	1.26	0.10	5.94E-02	0.38	0.03	3.16E-02
STX16	No	Yes	No	Up	1.43	0.00632	0.04171	0.84	0.11	4.50E-02	0.46	0.04	2.03E-02	0.99	0.11	3.50E-03	0.03	0.11	9.19E-01	0.40	0.14	1.10E-01	0.88	0.22	1.45E-02	0.50	0.10	1.20E-01	0.03	0.03	8.49E-01
DDIT3	No	No	No	Up	1.43	0.008351	validated	0.72	0.11	1.42E-02	0.17	0.04	3.42E-01	0.68	0.11	3.40E-02	0.36	0.11	2.47E-01	0.78	0.15	1.77E-02	0.43	0.20	2.04E-01	0.45	0.10	1.74E-01	0.55	0.03	3.60E-02
TCEAL2	No	No	No	Up	1.43	0.000125	validated	0.53	0.10	2.04E-02	0.17	0.04	1.91E-01	0.49	0.10	3.24E-02	0.58	0.12	4.63E-02	1.13	0.16	4.21E-04	0.64	0.21	3.98E-02	0.12	0.10	5.43E-01	0.48	0.03	1.82E-03
TPM4	No	No	No	Up	1.43	0.000121	validated	0.69	0.11	3.54E-02	1.07	0.04	1.14E-06	0.59	0.11	4.40E-02	0.40	0.12	1.65E-01	0.20	0.14	3.45E-01	0.48	0.21	1.28E-01	0.61	0.10	1.15E-02	0.11	0.03	5.76E-01
PHF19	No	No	No	Up	1.43	0.003812	0.04551	0.52	0.10	1.35E-01	0.64	0.04	1.37E-03	0.48	0.10	9.64E-02	0.63	0.12	5.66E-02	0.31	0.14	4.62E-01	0.53	0.21	2.59E-01	0.61	0.10	5.27E-02	0.43	0.03	2.28E-02
EID2	No	No	No	Up	1.43	0.031414	0.01724	0.14	0.10	6.56E-01	0.44	0.04	4.10E-02	0.42	0.10	2.71E-01	0.81	0.12	5.48E-02	0.49	0.14	1.33E-01	0.89	0.22	3.78E-02	0.44	0.10	1.37E-01	0.52	0.03	1.01E-01
FAM127B	No	No	No	Up	1.43	0.011932	validated	1.03	0.11	7.11E-03	0.30	0.04	1.64E-01	0.46	0.10	2.02E-01	0.24	0.11	5.57E-01	0.80	0.15	9.09E-03	0.18	0.20	4.17E-01	0.95	0.10	1.22E-02	0.18	0.03	3.75E-01
ASB1	No	No	No	Up	1.43	0.01236	0.01496	1.12	0.11	9.08E-03	0.61	0.04	6.50E-03	0.56	0.11	8.49E-02	0.81	0.12	4.19E-02	0.28	0.14	3.53E-01	0.35	0.20	3.59E-01	0.20	0.10	5.69E-01	0.23	0.03	1.65E-01
CHSY1	No	No	No	Up	1.43	0.008311	validated	0.04	0.10	8.94E-01	0.63	0.04	7.05E-03	0.72	0.11	7.13E-02	0.98	0.13	2.91E-02	0.08	0.14	5.79E-01	0.56	0.21	7.23E-02	0.48	0.10	1.47E-01	0.67	0.03	1.13E-02
PIGX	No	No	Yes	Up	1.43	0.002421	0.01481	0.57	0.10	8.37E-02	0.37	0.04	4.41E-02	0.81	0.11	7.65E-03	1.06	0.13	8.01E-03	0.10	0.14	7.27E-01	0.13	0.20	7.21E-01	0.85	0.10	6.92E-03	0.29	0.03	5.87E-02
CFL2	No	No	No	Up	1.44	0.015804	validated	0.27	0.10	3.88E-01	0.27	0.04	1.13E-01	0.47	0.10	7.17E-02	0.93	0.12	3.95E-03	0.39	0.14	5.69E-01	0.98	0.22	3.78E-01	0.28	0.10	2.64E-01	0.58	0.03	6.58E-03
PSTK	No	Yes	No	Up	1.44	0.003269	4.2E-05	0.98	0.11	4.51E-03	0.42	0.04	6.22E-02	1.00	0.11	4.30E-03	0.18	0.11	6.36E-01	0.25	0.14	5.51E-01	0.23	0.20	6.79E-01	0.61	0.10	6.80E-02	0.50	0.03	5.24E-03
RECQL4	No	Yes	No	Up	1.44	1.14E-05	0.00411	0.81	0.11	8.35E-03	0.96	0.04	2.50E-06	0.00	0.10	9.96E-01	0.38	0.12	2.34E-01	0.45	0.14	1.84E-01	0.19	0.20	5.70E-01	0.68	0.10	3.10E-02	0.69	0.03	3.98E-05
STAU1	No	No	No	Up	1.44	0.025215	0.01379	0.59	0.10	1.12E-01	0.44	0.04	5.88E-02	1.36	0.12	2.49E-03	0.85	0.12	1.20E-01	0.00	0.14	9.95E-01	0.29	0.20	2.41E-01	0.58	0.10	4.14E-02	0.08	0.03	7.93E-01
DYNLRB1	No	No	No	Up	1.44	0.012223	validated	1.20	0.11	1.43E-02	0.58	0.04	2.26E-02	0.57	0.11	1.21E-01	0.07	0.11	8.34E-01	0.30	0.14	1.83E-01	0.51	0.21	8.32E-02	0.64	0.10	8.32E-02	0.31	0.03	5.80E-02
ENOX1	No	No	No	Up	1.44	0.000177	0.01891	0.09	0.10	7.37E-01	0.64	0.04	1.88E-03	0.24	0.10	3.71E-01	1.48	0.14	1.61E-05	0.18	0.14	8.60E-01	0.00	0.20	9.99E-01	0.62	0.10	1.29E-01	0.93	0.03	2.08E-04
PTGS1	No	Yes	No	Up	1.44	9.98E-05	1.5E-19	0.90	0.11	4.31E-04	0.27	0.04	5.65E-02	0.34	0.10	1.28E-01	0.26	0.11	3.29E-01	0.59	0.14	2.76E-02	0.60	0.21	7.61E-02	0.92	0.10	6.68E-04	0.32	0.03	3.86E-02
MELK	No	Yes	No	Up	1.44	0.000127	0.00371	0.47	0.10	1.09E-01	0.62	0.04	6.70E-04	0.27	0.10	2.55E-01	0.43	0.12	1.29E-01	0.67	0.14	2.14E-02	0.31	0.20	2.45E-01	0.95	0.10	8.25E-04	0.47	0.03	9.59E-03
STXBP1	No	No	No	Up	1.44	0.006571	0.00618	0.20	0.10	5.65E-01	0.31	0.04	1.10E-01	0.45	0.10	8.09E-02	1.06	0.13	1.70E-03	0.96	0.15	4.09E-02	0.40	0.20	3.15E-01	0.51	0.10	4.47E-02	0.31	0.03	1.06E-01
TBC1D24	No	No	No	Up	1.44	0.019512	0.01821	0.98	0.11	3.43E-03	0.62	0.04	9.03E-03	0.59	0.11	4.59E-02	0.30	0.11	4.13E-01	1.03	0.15	9.86E-02	0.47	0.21	3.98E-01	0.02	0.10	9.39E-01	0.18	0.03	3.70E-01
ISYNA1	No	Yes	No	Up	1.44	0.000339	4.1E-05	0.63	0.11	2.75E-02	1.02	0.04	9.09E-06	0.88	0.11	5.72E-03	0.33	0.11	2.94E-01	0.45	0.14	1.04E-01	0.44	0.20	1.60E-01	0.41	0.10	1.09E-01	0.04	0.03	7.75E-01
AATK	No	Yes	No	Up	1.44	0.001148	1.5E-19	0.20	0.10	5.72E-01	0.89	0.04	1.05E-05	0.15	0.10	6.99E-01	0.19	0.11	4.45E-01	0.24	0.14	8.45E-01	0.57	0.21	6.55E-01	1.08	0.10	2.61E-02	0.88	0.03	3.56E-04
YPEL3	No	Yes	No	Up	1.44	0.004981	0.00802	1.21	0.11	2.65E-03	0.47	0.04	3.22E-02	0.29	0.10	3.80E-01	0.47	0.12	2.52E-01	0.76	0.15	1.69E-02	0.48	0.21	1.32E-01	0.12	0.10	7.07E-01	0.40	0.03	2.11E-02
DGKD	No	No	No	Up	1.44	0.033252	validated	0.28	0.10	3.62E-01	0.56	0.04	5.07E-03	0.78	0.11	8.25E-02	0.93	0.12	4.04E-02	0.37	0.14	5.59E-01	0.60	0.21	4.41E-01	0.04	0.10	8.87E-01	0.64	0.03	2.42E-02
KNTC1	No	No	No	Up	1.44	0.002525	validated	0.59	0.10	6.78E-02	0.72	0.04	6.51E-04	0.18	0.10	5.10E-01	0.55	0.12	1.66E-01	0.73	0.14	4.64E-02	0.25	0.20	4.11E-01	0.69	0.10	2.23E-02	0.50	0.03	3.40E-02
KIAA0355	No	No	No	Up	1.44	0.015951	validated	0.66	0.11	9.70E-02	0.07	0.04	6.97E-01	0.14	0.10	6.48E-01	0.16	0.11	7.43E-01	0.85	0.15	6.90E-03	0.80	0.22	2.48E-02	1.03	0.10	6.04E-03	0.51	0.03	1.42E-01
FBXO11	No	No	No	Up	1.44	0.002939	0.01887	0.90	0.11	2.02E-02	0.78	0.04	6.56E-03	0.65	0.11	3.84E-02	0.58	0.12	1.30E-01	0.07	0.14	6.32E-01	0.19	0.20	4.04E-01	0.61	0.10	2.67E-02	0.44	0.03	1.72E-02
CBX6	No	Yes	No	Up	1.44	0.002171	0.00015	0.35	0.10	2.46E-01	0.45	0.04	1.94E-02	0.70	0.11	1.82E-02	0.30	0.11	3.58E-01	0.70	0.14	1.59E-02	0.46	0.21	1.27E-01	0.93	0.10	7.76E-03	0.31	0.03	7.47E-02
DSEL	No	No	No	Up	1.44	0.000235	0.00019	0.11	0.10	6.56E-01	0.95	0.04	2.92E-04	0.21	0.10	3.17E-01	0.92	0.12	2.71E-03	0.38	0.14	4.72E-01	0.29	0.20	5.66E-01	0.95	0.10	6.92E-04	0.42	0.03	1.06E-02
COL24A1	No	No	No	Up	1.44	0.009534	validated	0.40	0.10	1.86E-01	0.68	0.04	1.57E-03	0.42	0.10	1.17E-01	0.25	0.11	3.60E-01	0.75	0.14	2.10E-01	0.72	0.21	3.12E-01	0.70	0.10	3.03E-02	0.30	0.03	5.28E-02
FXYD5	No	No	No	Up	1.44	0.00342	validated	0.40	0.10	1.42E-01	0.27	0.04	1.19E-01	0.06	0.10	8.34E-01	0.97	0.13	9.75E-03	0.78	0.15	7.33E-03	0.91	0.22	9.62E-03	0.43	0.10	2.35E-01	0.42	0.03	4.95E-02
GPR62	No	No	No	Up	1.44	0.01962	0.00016	1.17	0.11	3.08E-03	0.39	0.04	8.88E-02	0.22	0.10	6.26E-01	0.14	0.11	6.73E-01	0.07	0.14	9.58E-01	0.64	0.21	5.76E-01	1.08	0.10	5.10E-02	0.55	0.03	2.53E-03
NUDT3	No	No	No	Up	1.44	0.006141	validated	0.74	0.11	5.26E-02	0.74	0.04	4.11E-03	0.92	0.11	2.69E-02	0.09	0.11	8.57E-01	0.50	0.14	6.70E-02	0.35	0.20	2.26E-01	0.57	0.10	1.46E-01	0.34	0.03	4.02E-02
RIF1	No	No	No	Up	1.44	0.005314	0.00366	0.82	0.11	2.42E-02	0.86	0.04	2.57E-04	0.71	0.11	3.83E-02	0.33	0.11	3.37E-01	0.54	0.14	2.73E-01	0.64	0.21	2.79E-01	0.00	0.10	9.93E-01	0.34	0.03	5.13E-02
COL6A3	No	No	No	Up	1.44	1.14E-05	validated	0.16	0.10	5.34E-01	0.66	0.04	2.26E-04	0.11	0.10	6.16E-01	0.81	0.12	7.10E-03	1.12	0.16	3.23E-04	0.63	0.21	3.50E-02	0.06	0.10	7.73E-01	0.68	0.03	2.62E-04
COL11A1	No	No	No	Up	1.44	6.71E-06	validated	0.06	0.10	7.63E-01	0.81	0.04	8.86E-06	0.79	0.11	2.11E-03	0.52	0.12	4.03E-02	0.62	0.14	2.30E-02	0.80	0.22	1.54E-02	0.23	0.10	2.92E-01	0.42	0.03	3.81E-03
ESM1	No	No	No	Up	1.44	0.000186	validated	0.13	0.10	6.23E-01	0.65	0.04	7.08E-04	0.21	0.10	3.80E-01	1.27	0.13	1.46E-04	0.55	0.14	7.35E-02	0.49	0.21	1.14E-01	0.30	0.10	2.23E-01	0.65	0.03	4.73E-03
NRBP1	No	No	No	Up	1.45	0.043564	validated	0.87	0.11	3.99E-02	0.13	0.04	5.08E-01	0.64	0.11	1.11E-01	1.07	0.13	3.89E-02	0.88	0.15	1.31E-02	0.16	0.20	5.71E-01	0.21	0.10	5.12E-01	0.30	0.03	2.03E-01
FAM113A	No	Yes	No	Up	1.45	0.025878	0.00639	0.15	0.10	6.30E-01	0.29	0.04	2.64E-01	1.66	0.12	2.77E-04	0.14	0.11	7.24E-01	0.59	0.14	1.96E-01	1.00	0.22	4.73E-02	0.11	0.10	6.78E-01	0.31	0.03	7.96E-02
ZFPM2	No	No	No	Up	1.45	0.000632	0.00309	0.50	0.10	6.32E-02	0.09	0.04	5.34E-01	0.28	0.10	1.98E-01	0.65	0.12	1.06E-02	0.73	0.14	1.52E-01	0.55	0.21	2.72E-01	0.81	0.10	4.43E-03	0.65	0.03	1.69E-04
RAB32	No	Yes	No	Up	1.45	0.001494	0.00345	0.28	0.10	3.07E-01	0.11	0.04	4.73E-01	0.49	0.10	1.10E-01	1.21	0.13	4.69E-04	0.74	0.14	1.75E-02	0.76	0.21	3.97E-02	0.06	0.10	8.39E-01	0.61	0.03	3.79E-03
EXTL1	No	No	No	Up	1.45	0.008343	0.00045	0.78	0.11	2.17E-02	0.92	0.04	1.03E-04	0.16	0.10	7.14E-01	0.87	0.12	7.75E-03	0.36	0.14	5.51E-01	0.63	0.21	6.51E-01	0.50	0.10	2.55E-01	0.03	0.03	8.07E-01
SPOCK3	No	No	No	Up	1.45	0.006222	6.7E-06	0.40	0.10	9.35E-02	0.78	0.04	1.60E-03	1.03	0.11	4.48E-04	0.11	0.11	6.77E-01	0.75	0.14	5.94E-01	0.65	0.21	6.44E-01	0.39	0.10	9.59E-02	0.15	0.03	2.76E-01
SPAG6	No	No	No	Up	1.45	0.002455	0.00177	0.53	0.10	3.93E-02	0.42	0.04	1.01E-01	0.14	0.10	5.68E-01	0.42	0.12	1.32E-01	0.38	0.14	5.01E-01	0.28	0.20	4.26E-01	1.63	0.11	2.30E-04	0.47	0.03	3.43E-03
GPC5	No	Yes	No	Up	1.45	0.001995	1.5E-19	0.86	0.11	5.88E-03	0.57	0.04	6.06E-03	0.04	0.10	9.40E-01	0.88	0.12	1.04E-02	0.63	0.14	2.84E-01	0.65	0.21	3.68E-01	0.11	0.10	7.97E-01	0.54	0.03	1.03E-03
C16ORF74	No	No	No	Up	1.45	0.021416	2.6E-05	0.87	0.11	1.73E-03	0.21	0.04	1.69E-01	0.89	0.11	4.23E-02	0.50	0.12	9.44E-02	0.22	0.14	7.43E-01	0.66	0.21	5.22E-01	0.82	0.10	4.65E-02	0.11	0.03	4.81E-01
PRPS1	No	No	No	Up	1.45	0.010911	0.04485	0.56	0.10	7.77E-02	0.11	0.04	4.62E-01	0.30	0.10	2.95E-01	1.57	0.15	7.56E-04	0.34	0.14	2.02E-01	0.65	0.21	9.38E-02	0.07	0.10	7.80E-01	0.68	0.03	1.54E-02
RUNDC3A	No	Yes	No	Up	1.45	0.011524	1.5E-19	0.51	0.10	1.58E-01	0.83	0.04	1.55E-03	0.17	0.10	6.24E-01	0.20	0.11	5.13E-01	0.34	0.14	4.92E-01	0.68	0.21	5.54E-01	1.01	0.10	3.35E-02	0.54	0.03	2.95E-03
NPR2	No	No	No	Up	1.45	0.020564	validated	0.44	0.10	2.18E-01	0.23	0.04	1.76E-01	0.43	0.10	1.29E-01	0.24	0.11	4.61E-01	1.18	0.16	4.02E-03	1.11	0.23	1.67E-02	0.48	0.10	1.79E-01	0.19	0.03	3.35E-01
SPAG5	No	Yes	No	Up	1.45	0.001473	0.00142	0.82	0.11	8.53E-03	0.65	0.04	5.79E-04	0.40	0.10	1.24E-01	0.44	0.12	2.15E-01	0.69	0.14	2.78E-01	0.28	0.20	6.18E-01	0.46	0.10	9.69E-02	0.56	0.03	7.17E-03

Supplementary Table 2. Details for 2,053 genes differentially expressed between EEC and NEEC, identified by microarray meta-analysis.

EEC - endometrioid endometrial cancer, NEEC - non-endometrioid endometrial cancer, SMD - Standardised Mean Difference, FDR - False Discovery Rate, Var - Variance

Individual microarray study results

Gene	145 gene list	1253 gene list	Associated with EEC-specific survival?	Up/Down Regulated in NEEC	average standardised fold change	Microarray Meta FDR	RNASeq FDR	TCGA SMD	TCGA Var	TCGA P-value	E-MTAB-2532 SMD	E-MTAB-2532 Var	E-MTAB-2532 P-value	E-GEOD-56026 SMD	E-GEOD-56026 Var	E-GEOD-56026 P-value	GSE32507 SMD	GSE32507 Var	GSE32507 P-value	GSE24537 SMD	GSE24537 Var	GSE24537 P-value	E-GEOD-23518 SMD	E-GEOD-23518 Var	E-GEOD-23518 P-value	E-GEOD-17025 SMD	E-GEOD-17025 Var	E-GEOD-17025 P-value	E-GEOD-2109 SMD	E-GEOD-2109 Var	E-GEOD-2109 P-value
ERAL1	No	No	No	Up	1.45	0.033704	validated	0.98	0.11	2.83E-02	0.63	0.04	1.79E-02	0.42	0.10	2.16E-01	0.38	0.11	5.09E-01	0.35	0.14	1.60E-01	0.46	0.21	1.32E-01	0.67	0.10	1.36E-01	0.40	0.03	2.12E-01
REST	No	No	No	Up	1.45	0.033247	0.02367	0.48	0.10	1.57E-01	0.56	0.04	1.03E-02	0.56	0.11	1.35E-01	0.05	0.11	8.73E-01	0.05	0.14	8.99E-01	1.42	0.25	2.31E-01	0.84	0.10	1.30E-02	0.33	0.03	6.33E-02
PPIF	No	Yes	Yes	Up	1.45	0.029105	0.0102	0.73	0.11	9.48E-02	0.32	0.04	8.86E-02	0.12	0.10	6.12E-01	0.61	0.12	7.83E-02	0.83	0.15	4.02E-01	0.35	0.20	6.37E-01	0.85	0.10	8.41E-03	0.50	0.03	2.62E-02
SRGAP1	No	Yes	No	Up	1.45	0.002514	7.7E-05	1.07	0.11	6.65E-04	0.46	0.04	7.52E-03	0.39	0.10	1.42E-01	0.11	0.11	7.14E-01	0.98	0.15	2.00E-02	0.75	0.21	1.33E-01	0.38	0.10	1.43E-01	0.17	0.03	2.75E-01
ME1	No	Yes	No	Up	1.45	0.00416	4E-05	0.36	0.10	1.80E-01	0.32	0.04	5.23E-02	0.53	0.11	2.50E-02	0.23	0.11	3.57E-01	1.43	0.17	4.39E-02	0.52	0.21	3.60E-01	0.32	0.10	1.38E-01	0.61	0.03	9.72E-04
CRB2	No	Yes	No	Up	1.45	0.007351	1.5E-19	1.42	0.12	3.89E-04	0.47	0.04	4.96E-02	0.43	0.10	2.64E-01	0.53	0.12	5.54E-02	0.18	0.14	8.69E-01	0.22	0.20	8.43E-01	0.66	0.10	2.51E-01	0.42	0.03	1.30E-02
TTL	No	No	No	Up	1.45	0.009672	validated	0.45	0.10	1.62E-01	0.68	0.04	1.37E-02	1.05	0.11	3.83E-03	0.69	0.12	7.62E-02	0.78	0.15	6.99E-02	0.15	0.20	5.57E-01	0.36	0.10	2.05E-01	0.18	0.03	2.61E-01
CHEK1	No	No	No	Up	1.45	0.000745	validated	0.89	0.11	2.84E-03	0.58	0.04	1.36E-03	0.46	0.10	6.10E-02	0.83	0.12	1.07E-02	0.44	0.14	1.54E-01	0.74	0.21	8.48E-02	0.39	0.10	9.88E-02	0.00	0.03	9.86E-01
ABCC5	No	Yes	No	Up	1.45	0.001319	0.00925	0.66	0.11	8.15E-02	0.62	0.04	1.36E-03	1.27	0.12	6.89E-04	0.12	0.11	7.59E-01	0.47	0.14	1.34E-01	0.35	0.20	2.94E-01	0.35	0.10	1.76E-01	0.49	0.03	3.15E-02
MASTL	No	Yes	No	Up	1.45	0.006853	0.00178	0.70	0.11	2.20E-02	0.56	0.04	1.14E-02	0.47	0.10	7.78E-02	0.59	0.12	7.36E-02	0.63	0.14	1.58E-01	0.51	0.21	2.81E-01	0.66	0.10	2.38E-02	0.19	0.03	3.77E-01
NAT10	No	No	No	Up	1.45	0.039473	validated	0.76	0.11	1.24E-01	0.19	0.04	4.38E-01	0.68	0.11	6.96E-02	0.26	0.11	6.60E-01	0.67	0.14	2.65E-02	0.67	0.21	5.73E-02	0.49	0.10	1.91E-01	0.60	0.03	7.21E-02
STAG1	No	No	No	Up	1.45	0.018272	validated	0.71	0.11	1.98E-02	0.40	0.04	9.90E-02	0.20	0.10	5.14E-01	0.77	0.12	5.19E-02	0.70	0.14	4.29E-02	0.83	0.22	7.45E-02	0.61	0.10	6.49E-02	0.09	0.03	6.27E-01
FHL3	No	No	No	Up	1.45	0.005672	validated	0.50	0.10	1.58E-01	0.43	0.04	3.75E-02	0.28	0.10	4.97E-01	0.83	0.12	3.46E-02	0.51	0.14	9.29E-02	1.06	0.23	1.38E-02	0.08	0.10	8.67E-01	0.65	0.03	3.26E-03
MRPS18B	No	Yes	No	Up	1.45	0.0128	0.03134	0.99	0.11	2.14E-02	0.75	0.04	1.12E-02	0.43	0.10	2.27E-01	0.05	0.11	9.17E-01	0.25	0.14	3.08E-01	0.89	0.22	1.62E-02	0.82	0.10	1.99E-02	0.16	0.03	5.51E-01
TTYH3	No	No	No	Up	1.46	0.003119	validated	0.71	0.11	3.23E-02	0.53	0.04	1.12E-02	0.00	0.10	9.93E-01	0.72	0.12	7.46E-02	1.00	0.15	2.93E-03	0.43	0.20	1.48E-01	0.40	0.10	3.10E-01	0.54	0.03	2.51E-02
FSTL5	No	No	No	Up	1.46	0.00022	validated	0.34	0.10	1.95E-01	0.73	0.04	3.55E-04	0.17	0.10	5.95E-01	0.95	0.12	2.05E-03	0.61	0.14	5.80E-02	0.67	0.21	6.00E-02	0.50	0.10	7.79E-02	0.35	0.03	1.20E-02
ZSWIM1	No	No	No	Up	1.46	0.017065	0.00479	0.84	0.11	3.42E-02	0.69	0.04	4.62E-03	0.35	0.10	3.10E-01	0.57	0.12	3.08E-01	0.08	0.14	6.48E-01	0.34	0.20	2.90E-01	1.02	0.10	3.47E-02	0.45	0.03	5.75E-02
PCSK1	No	No	No	Up	1.46	0.000642	0.04772	0.61	0.10	1.74E-02	0.48	0.04	5.11E-03	0.21	0.10	3.81E-01	0.09	0.11	6.78E-01	1.14	0.16	6.67E-03	0.95	0.22	2.94E-02	0.46	0.10	1.02E-01	0.41	0.03	4.97E-03
MX1	No	Yes	No	Up	1.46	1.14E-05	1.5E-19	0.66	0.11	1.31E-02	0.61	0.04	2.41E-04	0.90	0.11	1.05E-03	0.09	0.11	7.01E-01	0.92	0.15	1.50E-03	0.43	0.20	1.11E-01	0.41	0.10	7.43E-02	0.32	0.03	5.67E-02
RBM38	No	Yes	No	Up	1.46	0.003331	1.5E-19	0.89	0.11	1.64E-02	0.49	0.04	1.61E-02	0.90	0.11	6.13E-03	0.35	0.11	3.48E-01	0.22	0.14	2.96E-01	0.62	0.21	6.04E-02	0.31	0.10	3.34E-01	0.56	0.03	3.20E-02
C10ORF125	No	Yes	No	Up	1.46	0.001029	3.8E-05	0.84	0.11	7.97E-03	0.80	0.04	1.24E-04	0.55	0.11	7.01E-02	0.09	0.11	7.75E-01	0.84	0.15	9.19E-02	0.36	0.20	4.13E-01	0.20	0.10	5.44E-01	0.67	0.03	6.54E-03
PPFIBP1	No	Yes	No	Up	1.46	0.000334	1.5E-19	1.13	0.11	1.38E-03	0.78	0.04	1.95E-04	0.94	0.11	2.23E-03	0.26	0.11	3.68E-01	0.44	0.14	1.46E-01	0.35	0.20	3.19E-01	0.16	0.10	4.25E-01	0.28	0.03	1.32E-01
PNO1	No	Yes	Yes	Up	1.46	0.001282	0.00104	0.82	0.11	1.51E-02	0.44	0.04	1.60E-02	0.10	0.10	7.29E-01	1.04	0.13	2.73E-03	0.16	0.14	5.38E-01	0.48	0.21	2.21E-01	0.55	0.10	7.52E-02	0.77	0.03	2.77E-03
KLHL29	No	No	No	Up	1.46	0.000213	0.00132	0.09	0.10	6.98E-01	0.53	0.04	3.17E-03	0.99	0.11	8.95E-04	1.01	0.13	1.29E-03	0.74	0.14	1.11E-01	0.21	0.20	5.26E-01	0.41	0.10	8.91E-02	0.37	0.03	2.02E-02
KIAA0196	No	Yes	No	Up	1.46	0.011487	0.008	0.66	0.11	2.88E-02	0.32	0.04	1.01E-01	0.83	0.11	5.40E-02	1.25	0.13	4.06E-03	0.40	0.14	1.34E-01	0.53	0.21	9.46E-02	0.28	0.10	3.16E-01	0.08	0.03	8.17E-01
SLMO2	No	Yes	No	Up	1.46	0.001007	0.03242	0.47	0.10	1.97E-01	0.92	0.04	1.90E-04	1.17	0.11	2.88E-03	0.30	0.11	3.50E-01	0.16	0.14	4.24E-01	0.60	0.21	8.82E-02	0.06	0.10	7.85E-01	0.67	0.03	6.23E-03
GPC3	No	No	No	Up	1.46	2.85E-05	validated	0.24	0.10	2.85E-01	0.62	0.04	2.07E-04	0.18	0.10	4.41E-01	1.47	0.14	6.13E-06	0.73	0.14	1.29E-02	0.58	0.21	1.04E-01	0.29	0.10	2.31E-01	0.25	0.03	1.32E-01
CSE1L	No	Yes	Yes	Up	1.46	0.000209	0.00028	0.28	0.10	4.77E-01	0.88	0.04	3.34E-04	0.82	0.11	1.61E-02	0.37	0.11	4.07E-01	0.51	0.14	4.23E-02	0.46	0.21	1.08E-01	0.15	0.10	5.06E-01	0.89	0.03	1.01E-04
CENPQ	No	No	No	Up	1.46	0.025552	validated	0.17	0.10	5.68E-01	0.86	0.04	6.63E-04	0.15	0.10	5.83E-01	0.70	0.12	6.05E-02	0.60	0.14	4.64E-01	0.82	0.22	4.57E-01	0.62	0.10	5.39E-02	0.44	0.03	1.07E-01
LSG1	No	Yes	Yes	Up	1.46	0.004537	0.00168	1.71	0.13	9.63E-04	0.84	0.04	1.02E-03	1.03	0.11	5.01E-03	0.21	0.11	5.40E-01	0.10	0.14	6.51E-01	0.11	0.20	6.36E-01	0.07	0.10	8.23E-01	0.29	0.03	2.42E-01
PCDH8	No	No	No	Up	1.46	3.97E-05	validated	0.44	0.10	6.50E-02	0.42	0.04	1.87E-02	0.71	0.11	1.01E-02	1.42	0.14	7.27E-06	0.45	0.14	7.30E-01	0.30	0.20	6.07E-01	0.06	0.10	8.95E-01	0.56	0.03	2.92E-04
SLC1A6	No	No	No	Up	1.46	0.021107	0.00042	0.62	0.10	5.69E-02	0.43	0.04	6.98E-02	0.38	0.10	1.76E-01	0.50	0.12	6.39E-02	0.73	0.14	5.85E-01	0.68	0.21	6.06E-01	0.58	0.10	1.97E-01	0.45	0.03	3.15E-03
RAD54L	No	Yes	No	Up	1.46	0.000294	6E-05	0.73	0.11	1.65E-02	0.89	0.04	1.00E-05	0.38	0.10	1.90E-01	0.31	0.11	3.09E-01	0.70	0.14	2.10E-01	0.68	0.21	2.20E-01	0.11	0.10	6.66E-01	0.58	0.03	1.50E-03
MSN	No	Yes	No	Up	1.46	0.000602	0.00151	0.69	0.11	3.75E-02	0.60	0.04	3.10E-03	0.09	0.10	7.17E-01	0.21	0.11	4.44E-01	1.63	0.18	1.41E-05	0.89	0.22	1.06E-02	0.13	0.10	5.62E-01	0.11	0.03	6.11E-01
RAB9B	No	No	No	Up	1.46	0.003985	0.01523	0.75	0.11	2.63E-02	0.22	0.04	2.73E-01	0.19	0.10	5.26E-01	1.13	0.13	2.34E-04	1.00	0.15	1.08E-01	0.29	0.20	5.25E-01	0.31	0.10	3.22E-01	0.47	0.03	9.95E-03
SLC38A2	No	Yes	No	Up	1.46	0.000956	3.1E-05	1.58	0.12	3.11E-04	0.98	0.04	6.41E-05	0.81	0.11	2.20E-02	0.10	0.11	7.84E-01	0.26	0.14	2.64E-01	0.32	0.20	2.67E-01	0.28	0.10	2.95E-01	0.04	0.03	8.70E-01
APOBEC2	No	No	No	Up	1.46	0.00058	validated	0.03	0.10	9.32E-01	0.41	0.04	3.89E-02	0.08	0.10	8.48E-01	0.89	0.12	7.37E-04	0.46	0.14	5.77E-01	0.64	0.21	4.75E-01	0.84	0.10	7.15E-02	1.02	0.04	3.86E-06
PIP5KL1	No	Yes	No	Up	1.46	0.006966	1.5E-19	0.71	0.11	4.93E-02	0.88	0.04	1.37E-03	0.43	0.10	2.73E-01	0.31	0.11	2.65E-01	0.45	0.14	6.25E-01	0.03	0.20	9.61E-01	1.13	0.10	1.18E-02	0.43	0.03	1.70E-02
PCK1	No	Yes	No	Up	1.46	1.14E-05	1.5E-19	0.78	0.11	1.01E-02	0.79	0.04	6.70E-05	0.71	0.11	3.35E-03	0.43	0.12	7.39E-02	0.51	0.14	2.83E-01	0.02	0.20	9.70E-01	0.63	0.10	1.28E-02	0.52	0.03	6.50E-04
ZFP42	No	Yes	No	Up	1.46	0.013593	1.5E-19	0.04	0.10	8.75E-01	0.60	0.04	1.79E-02	0.59	0.11	2.23E-02	0.74	0.12	1.21E-02	0.62	0.14	3.24E-01	0.85	0.22	2.29E-01	0.64	0.10	2.06E-01	0.31	0.03	4.90E-02
DMRT2	No	No	No	Up	1.46	0.000227	0.00938	0.06	0.10	7.99E-01	0.93	0.04	3.63E-05	0.02	0.10	9.27E-01	1.23	0.13	3.82E-05	0.62	0.14	1.15E-01	0.69	0.21	1.02E-01	0.63	0.10	1.12E-01	0.18	0.03	2.20E-01
PRKACA	No	Yes	No	Up	1.46	0.03801	0.00967	0.67	0.11	1.40E-01	0.57	0.04	3.44E-02	0.05	0.10	8.95E-01	0.21	0.11	5.83E-01	0.43	0.14	5.45E-01	0.56	0.21	4.18E-01	1.46	0.11	5.12E-03	0.44	0.03	1.60E-02
KCNIP1	No	Yes	No	Up	1.46	0.031923	1.5E-19	0.49	0.10	1.21E-01	0.15	0.04	4.75E-01	0.37	0.10	4.25E-01	0.57	0.12	2.42E-02	1.09	0.15	5.34E-02	0.76	0.21	1.62E-01	0.54	0.10	3.09E-01	0.43	0.03	1.84E-02
ATG9A	No	No	No	Up	1.46	0.027903	0.04413	1.11	0.11	1.34E-02	0.41	0.04	1.42E-01	0.09	0.10	8.08E-01	0.28	0.11	6.27E-01	1.22	0.16	3.52E-03	0.23	0.20	4.55E-01	0.68	0.10	7.30E-02	0.38	0.03	1.82E-01
BLM	No	Yes	No	Up	1.46	0.003218	0.00162	0.55	0.10	7.07E-02	0.61	0.04	2.15E-03	0.01	0.10	9.76E-01	0.32	0.11	3.56E-01	0.42	0.14	2.92E-01	0.81	0.22	1.09E-01	0.81	0.10	1.86E-02	0.87	0.03	3.14E-03
IFI44L	No	Yes	No	Up	1.46	1.83E-19	1.5E-19	1.08	0.11	9.79E-05	0.58	0.04	3.92E-04	0.82	0.11	1.25E-03	0.04	0.11	8.46E-01	0.75	0.14	8.55E-03	0.17	0.20	4.06E-01	0.44	0.10	4.43E-02	0.51	0.03	1.32E-03
TWIST1	No	No	No	Up	1.46	1.14E-05	validated	0.11	0.10	6.40E-01	0.68	0.04	9.41E-05	0.36	0.10	9.52E-02	1.90	0.16	4.54E-07	0.68	0.14	2.00E-02	0.26	0.20	2.78E-01	0.12	0.10	5.41E-01	0.28	0.03	9.73E-02
CALCA	No	No	No	Up	1.46	7.73E-05	0.0003	1.48	0.12	3.18E-06	0.43	0.04	6.24E-03	0.37	0.10	9.33E-02	0.94	0.12	8.90E-04	0.31	0.14	1.74E-01	0.60	0.21	2.33E-01	0.27	0.10	5.34E-01	0.01	0.03	9.29E-01
MAZ	No	No	No	Up	1.46	0.015614	validated	0.64	0.11	1.49E-01	0.55	0.04	1.89E-02	0.35	0.10	3.12E-01	0.10	0.11	8.00E-01	0.62	0.14	1.13E-01	0.64	0.21	1.15E-01	1.26	0.10	2.94E-03	0.24	0.03	1.68E-01
MATN4	No	No	No	Up	1.46	0.000876	2.2E-05	0.51	0.10	5.37E-02	1.19	0.04	4.54E-07	0.17	0.10	7.33E-01	0.69	0.12	2.12E-02	0.26	0.14	8.30E-01	0.94	0.22	5.17E-01	0.31	0.10	4.65E-01	0.35	0.03	6.49E-02
EID3	No	No	No	Up	1.47	0.005987	0.00311	0.55	0.10	4.69E-02	0.41	0.04	4.11E-02	0.42	0.10	1.48E-01	0.84	0.12	1.43E-02	0.36	0.14	6.26E-01	0.62	0.21	4.71E-01	0.60	0.10	3.69E-02	0.62	0.03	9.37E-03
ZDHHC15	No	No	No	Up	1.47	0.001209	0.00543	0.78	0.11	7.48E-03	0.59	0.04	4.01E-02	0.56	0.11	5.17E-02	1.02	0.13	2.27E-03	0.07	0.14	8.94E-01	0.44	0.20	5.85E-01	0.24	0.10	3.89E-01	0.71	0.03	1.49E-03
ELOF1	No	No	No	Up	1.47	0.046575	validated	1.24	0.12	2.93E-03	0.36	0.04	1.05E-01	0.63	0.11	1.73E-01	0.56	0.12	2.45E-01	1.06	0.15	4.40E-02	0.37	0.20	2.95E-01	0.09	0.10	7.09E-01	0.10	0.03	6.89E-01
ZMYM1	No	No	No	Up	1.47	0.009216	0.01055	0.59	0.10	8.26E-02	0.67	0.04	4.94E-03	0.64	0.11	9.40E-02	0.65	0.12	1.46E-01	0.46	0.14	1.58E-01	0.32	0.20	3.37E-01	0.94	0.10	7.25E-03	0.15	0.03	5.47E-01
RIMS4	No	No	No	Up	1.47	0.000215	0.00075	1.03	0.11	1.14E-03	0.67	0.04	6.45E-05	0.77	0.11	2.94E-02	0.82	0.12	3.53E-03	0.43	0.14	3.46E-01	0.31	0.20	4.63E-01	0.26	0.10	5.48E-01	0.13	0.03	4.07E-01
MAGEA11	No	Yes	No	Up	1.47	1.83E-19	1.5E-19	0.80	0.11	4.50E-03	0.54	0.04	5.60E-03	0.33	0.10	1.42E-01	0.18	0.11	4.57E-01	0.38	0.14	4.53E-01	0.43	0.20	3.96E-01	0.81	0.10	2.32E-03	0.96	0.03	1.00E-20
PLK1	No	Yes	No	Up	1.47	0.001104	0.00014	0.84	0.11	1.59E-02	0.71	0.04	2.04E-04	0.54	0.11	1.07E-01	0.81	0.12	2.00E-02	0.44	0.14	3.25E-01	0.27	0.20	5.47E-01	0.35	0.10	3.60E-01	0.45	0.03	1.95E-02
MTERFD1	No	No	No	Up	1.47	0.019355	validated	1.09	0.11	6.53E-03	0.40	0.04	7.91E-02	0.82	0.11	4.18E-02	0.97	0.13	5.11E-02	0.14	0.14	4.53E-01	0.44	0.20	1.48E-01	0.19	0.10	5.70E-01	0.38	0.03	1.85E-01
SETBP1	No	No	No	Up	1.47	0.000601	0.02772	1.04	0.11	5.04E-04	0.17	0.04	2.65E-01	0.54	0.11	5.08E-02	0.78	0.12	1.54E-02	0.63	0.14	3.31E-02	0.39	0.20	1.89E-01	0.49	0.10	5.25E-02	0.38	0.03	5.37E-02
SNX10	No	No	No	Up	1.47	0.000569	0.00236	0.51	0.10	5.01E-02	0.55	0.04	1.86E-03	0.25	0.10	3.04E-01	0.22	0.11	4.51E-01	0.63	0.14	1.94E-01	0.64	0.21	4.09E-01	1.09	0.10	2.33E-04	0.55	0.03	7.06E-03
RAB33A	No	No	No	Up	1.47	0.021446	validated	0.53	0.10	6.99E-02	0.45	0.04	1.25E-02	0.41	0.10	2.60E-01	0.98	0.13	4.83E-03	0.49	0.14	5.74E-01	1.11	0.23	3.25E-01	0.06	0.10	8.94E-01	0.41	0.03	5.59E-02
NRBP2	No	Yes	No	Up	1.47	0.00074	0.00123	1.16	0.11	1.92E-03	0.78	0.04	1.44E-04	0.75	0.11	2.57E-02	0.53	0.12	1.19E-01	0.29	0.14	2.33E-01	0.26	0.20	3.48E-01	0.45	0.10	1.25E-01	0.22	0.03	3.67E-01
SGOL2	No	No	No	Up	1.47	0.002272	0.00296	0.61	0.10	4.72E-02	0.46	0.04	1.46E-02	0.33	0.10	2.01E-01	0.90	0.12	9.36E-03	0.68	0.14	1.06E-01	0.52	0.21	1.88E-01	0.42	0.10	8.86E-02	0.54	0.03	1.80E-02
NBEA	No	No	No	Up	1.47	0.000125	0.03998	0.18	0.10	4.49E-01	0.17	0.04	2.38E-01	0.71	0.11	5.29E-03	0.91	0.12	2.75E-03	0.30	0.14	4.27E-01	0.45	0.21	3.72E-01	1.19	0.10	4.98E-05	0.55	0.03	7.04E-03
AHNAK2	No	Yes	No	Up	1.47	1.14E-05	1.5E-19	0.95	0.11	8.42E-04	0.31	0.04	3.89E-02	0.69	0.11	7.32E-03	0.07	0.11	7.38E-01	0.62	0.14	2.03E-02	0.40	0.20	1.39E-01	1.36	0.11	4.77E-06	0.05	0.03	7.42E-01
CEP72	No	No	No	Up	1.47	0.002082	0.00048	0.55	0.10	8.08E-02	0.58	0.04	2.85E-03	0.43	0.10	1.25E-01	0.48	0.12	2.04E-01	0.23	0.14	6.96E-01	1.01	0.23	2.60E-01	0.49	0.10	6.31E-02	0.70	0.03	4.92E-04
MYBL2	No	Yes	No	Up	1.47	0.000361	4.7E-05	0.59	0.10	5.29E-02	0.81	0.04	3.88E-05	0.55	0.11	6.12E-02	0.69	0.12	5.14E-02	0.25	0.14	7.72E-01	0.31	0.20	8.01E-01	0.83	0.10	1.52E-02	0.43	0.03	1.11E-02
PRPH	No	No	No	Up	1.47	2.40E-05	0.01582	1.03	0.11	6.34E-04	0.61	0.04	2.09E-04	0.12	0.10	6.51E-01	0.61	0.12	2.26E-02	1.00	0.15	8.73E-04	0.68	0.21	4.22E-02	0.15	0.10	6.11E-01	0.28	0.03	7.56E-02
RACGAP1	No	Yes	No	Up	1.47	0.000399	0.00129	0.50	0.10	9.65E-02	0.53	0.04	3.81E-03	0.30	0.10	2.23E-01	1.03	0.13	5.36E-03	0.48	0.14	1.06E-01	0.39	0.20	2.10E-01	0.64	0.10	1.61E-02	0.61	0.03	5.38E-03

Supplementary Table 2. Details for 2,053 genes differentially expressed between EEC and NEEC, identified by microarray meta-analysis.

EEC - endometrioid endometrial cancer, NEEC - non-endometrioid endometrial cancer, SMD - Standardised Mean Difference, FDR - False Discovery Rate, Var - Variance

Gene	145 gene list	1253 gene list	Associated with EEC-specific survival?	Up/Down Regulated in NEEC	average standardised fold change	Microarray Meta FDR	RNASeq FDR	Individual microarray study results																							
								TCGA			E-MTAB-2532			E-GEOD-56026			GSE32507			GSE24537			E-GEOD-23518			E-GEOD-17025			E-GEOD-2109		
								SMD	Var	P-value	SMD	Var	P-value	SMD	Var	P-value	SMD	Var	P-value	SMD	Var	P-value	SMD	Var	P-value	SMD	Var	P-value	SMD	Var	P-value
SYDE1	No	No	No	Up	1.47	0.009902	validated	0.15	0.10	6.84E-01	0.38	0.04	7.95E-02	0.54	0.11	1.37E-01	0.50	0.12	1.53E-01	1.08	0.15	5.38E-03	0.55	0.21	1.20E-01	1.01	0.10	2.43E-02	0.27	0.03	7.93E-02
POLQ	No	Yes	No	Up	1.48	0.000257	1.5E-19	0.96	0.11	5.40E-03	0.74	0.04	2.09E-04	0.18	0.10	4.94E-01	0.60	0.12	5.67E-02	0.59	0.14	1.62E-01	0.46	0.21	2.50E-01	0.29	0.10	2.51E-01	0.68	0.03	1.12E-03
MAP4K4	No	No	No	Up	1.48	0.00167	validated	0.54	0.10	1.56E-01	0.76	0.04	1.78E-03	0.49	0.10	7.38E-02	0.92	0.12	1.34E-02	0.28	0.14	3.99E-01	0.56	0.21	1.63E-01	0.20	0.10	3.92E-01	0.74	0.03	3.00E-03
BHLHB9	No	No	No	Up	1.48	0.009565	validated	0.61	0.10	3.84E-02	0.57	0.04	1.14E-02	0.61	0.11	6.46E-02	0.56	0.12	7.47E-02	0.56	0.14	1.81E-01	1.20	0.24	5.91E-02	0.12	0.10	6.85E-01	0.27	0.03	8.48E-02
NOL11	No	Yes	No	Up	1.48	0.007545	0.00394	0.32	0.10	2.35E-01	0.39	0.04	3.82E-02	0.37	0.10	2.95E-01	0.67	0.12	7.34E-02	0.79	0.15	2.01E-02	0.87	0.22	2.62E-02	0.24	0.10	4.38E-01	0.84	0.03	1.60E-02
SAMD5	No	Yes	No	Up	1.48	0.001604	1.3E-05	0.64	0.11	2.13E-02	0.20	0.04	2.01E-01	0.77	0.11	5.18E-03	0.74	0.12	1.06E-02	0.88	0.15	1.45E-01	0.31	0.20	5.30E-01	0.62	0.10	3.45E-02	0.34	0.03	3.05E-02
RBM28	No	No	No	Up	1.48	0.013167	validated	0.75	0.11	5.83E-02	0.49	0.04	4.74E-02	0.85	0.11	1.17E-02	0.56	0.12	2.21E-01	0.44	0.14	2.85E-01	0.21	0.20	5.76E-01	0.24	0.10	4.54E-01	0.97	0.03	5.45E-03
SMARCD3	No	Yes	No	Up	1.48	0.000343	0.00158	0.50	0.10	5.22E-02	0.56	0.04	2.28E-03	0.94	0.11	2.20E-03	0.99	0.13	2.01E-03	0.56	0.14	9.55E-02	0.39	0.20	2.77E-01	0.39	0.10	1.48E-01	0.18	0.03	2.99E-01
PIF1	No	Yes	No	Up	1.48	0.007771	0.00025	0.43	0.10	1.74E-01	0.70	0.04	2.41E-04	0.31	0.10	2.87E-01	0.50	0.12	2.39E-01	0.59	0.14	3.40E-01	1.01	0.23	1.63E-01	0.88	0.10	8.39E-03	0.08	0.03	5.63E-01
GRIN2C	No	No	No	Up	1.48	0.001872	0.00053	0.28	0.10	4.08E-01	0.89	0.04	1.68E-05	0.02	0.10	9.59E-01	1.33	0.14	2.32E-04	0.73	0.14	4.86E-01	0.63	0.21	5.25E-01	0.38	0.10	4.50E-01	0.26	0.03	1.46E-01
JAKMIP2	No	No	No	Up	1.48	0.004599	0.01584	0.46	0.10	9.24E-02	0.80	0.04	3.01E-04	0.33	0.10	2.83E-01	0.66	0.12	1.07E-02	0.25	0.14	8.08E-01	1.10	0.23	5.47E-01	0.55	0.10	2.51E-01	0.38	0.03	2.44E-02
ONECUT2	No	No	No	Up	1.48	1.83E-19	6.7E-05	0.41	0.10	1.42E-01	1.24	0.04	1.00E-20	0.22	0.10	3.72E-01	0.48	0.12	8.36E-02	0.52	0.14	3.81E-01	0.58	0.21	2.78E-01	0.38	0.10	1.54E-01	0.71	0.03	1.01E-04
ATOH8	No	No	No	Up	1.48	0.001055	0.01228	0.56	0.10	4.85E-02	0.08	0.04	5.97E-01	0.92	0.11	1.69E-02	0.63	0.12	2.35E-02	0.79	0.15	1.89E-02	0.42	0.20	2.10E-01	0.44	0.10	2.16E-01	0.71	0.03	7.87E-04
RAB2B	No	No	No	Up	1.48	0.027429	validated	0.67	0.11	1.01E-01	0.05	0.04	8.11E-01	0.94	0.11	2.84E-02	0.98	0.13	5.82E-02	0.23	0.14	2.98E-01	0.37	0.20	1.88E-01	0.96	0.10	8.69E-03	0.34	0.03	2.96E-01
TREML2	No	Yes	No	Up	1.48	0.003957	1.5E-19	0.99	0.11	8.41E-04	0.35	0.04	4.78E-02	0.08	0.10	8.38E-01	0.01	0.11	9.85E-01	0.21	0.14	7.71E-01	1.56	0.26	1.95E-01	0.58	0.10	2.66E-01	0.79	0.03	1.18E-04
PRRG1	No	Yes	Yes	Up	1.48	0.000745	1.5E-19	1.42	0.12	6.35E-04	0.34	0.04	9.51E-02	0.72	0.11	9.87E-03	0.10	0.11	7.32E-01	0.68	0.14	6.16E-02	0.02	0.20	9.29E-01	0.58	0.10	3.62E-02	0.70	0.03	3.56E-03
KIRREL2	No	Yes	Yes	Up	1.48	8.71E-05	1.5E-19	0.30	0.10	2.02E-01	1.15	0.04	4.54E-07	0.46	0.10	1.83E-01	1.13	0.13	1.37E-04	0.25	0.14	8.01E-01	0.75	0.21	5.06E-01	0.25	0.10	5.17E-01	0.28	0.03	9.04E-02
KIF15	No	No	Yes	Up	1.48	0.00011	0.00761	0.53	0.10	6.12E-02	0.60	0.04	9.80E-04	0.31	0.10	1.92E-01	0.97	0.13	3.49E-03	0.20	0.14	4.55E-01	0.55	0.21	2.44E-01	0.75	0.10	4.11E-03	0.64	0.03	4.24E-03
PHF20L1	No	No	No	Up	1.48	0.003975	validated	0.70	0.11	8.48E-02	0.33	0.04	5.87E-02	0.43	0.10	1.26E-01	1.14	0.13	2.99E-03	0.50	0.14	1.19E-01	0.73	0.21	5.81E-02	0.65	0.10	2.14E-02	0.10	0.03	5.70E-01
FXYD1	No	No	No	Up	1.49	0.000381	0.00305	1.06	0.11	1.27E-03	0.03	0.04	8.24E-01	0.25	0.10	5.06E-01	0.81	0.12	5.59E-03	1.01	0.15	1.03E-02	0.70	0.21	6.26E-02	0.14	0.10	7.58E-01	0.56	0.03	5.28E-04
LECT1	No	No	No	Up	1.49	0.001066	0.00107	1.10	0.11	2.43E-04	0.64	0.04	6.24E-03	0.41	0.10	4.96E-01	0.29	0.11	3.45E-01	0.64	0.14	4.75E-01	0.44	0.20	2.66E-01	0.45	0.10	2.97E-01	0.60	0.03	8.14E-04
RPS6KA6	No	Yes	No	Up	1.49	0.00349	1.5E-19	1.00	0.11	8.23E-04	0.72	0.04	1.44E-02	0.66	0.11	2.66E-02	0.14	0.11	5.86E-01	0.96	0.15	3.16E-01	0.25	0.20	7.60E-01	0.47	0.10	1.23E-01	0.36	0.03	2.16E-02
HEATR6	No	No	No	Up	1.49	0.033435	0.01638	0.25	0.10	5.41E-01	0.54	0.04	3.88E-02	0.36	0.10	3.67E-01	0.89	0.12	5.66E-02	0.55	0.14	1.72E-01	0.82	0.22	1.11E-01	0.79	0.10	4.31E-02	0.37	0.03	9.13E-02
SPON1	No	Yes	No	Up	1.49	3.58E-06	1.5E-19	1.51	0.12	4.54E-07	0.26	0.04	4.90E-02	0.56	0.11	1.25E-02	0.17	0.11	4.22E-01	0.28	0.14	1.73E-01	0.77	0.21	1.54E-02	0.66	0.10	4.06E-03	0.38	0.03	1.03E-02
PHF1	No	No	No	Up	1.49	0.003011	validated	0.82	0.11	4.50E-02	0.69	0.04	1.34E-03	0.39	0.10	2.89E-01	0.21	0.11	6.45E-01	0.88	0.15	6.22E-03	0.52	0.21	1.27E-01	1.02	0.10	1.22E-02	0.05	0.03	7.60E-01
TROAP	No	Yes	No	Up	1.49	0.000167	5.1E-05	0.66	0.11	4.75E-02	0.84	0.04	2.20E-05	0.29	0.10	3.02E-01	0.17	0.11	5.71E-01	0.41	0.14	1.95E-01	0.76	0.21	6.76E-02	0.99	0.10	3.34E-03	0.47	0.03	1.97E-02
FOXM1	No	Yes	No	Up	1.49	4.80E-05	0.00075	0.68	0.11	2.23E-02	0.78	0.04	6.75E-05	0.31	0.10	1.98E-01	0.78	0.12	2.85E-02	0.57	0.14	1.05E-01	0.50	0.21	1.79E-01	0.36	0.10	1.47E-01	0.62	0.03	6.19E-04
FIGN	No	Yes	No	Up	1.49	4.80E-05	1.5E-19	1.20	0.11	6.88E-04	0.58	0.04	1.50E-03	0.76	0.11	4.67E-03	0.27	0.11	2.80E-01	0.53	0.14	6.00E-01	0.38	0.20	6.37E-01	0.14	0.10	4.92E-01	0.74	0.03	4.93E-05
C1QTNF4	No	No	No	Up	1.49	0.005476	0.02715	0.71	0.11	2.14E-02	0.31	0.04	7.24E-02	0.42	0.10	1.56E-01	0.77	0.12	1.54E-02	0.69	0.14	1.77E-01	0.63	0.21	3.12E-01	0.64	0.10	2.45E-01	0.45	0.03	6.64E-03
ST8SIA2	No	No	No	Up	1.49	8.89E-05	0.00086	0.21	0.10	6.11E-01	0.48	0.04	6.32E-02	1.22	0.11	1.20E-04	0.33	0.11	2.49E-01	0.24	0.14	6.39E-01	0.80	0.22	1.69E-01	0.69	0.10	5.58E-03	0.64	0.03	6.45E-05
ODC1	No	Yes	No	Up	1.49	0.000327	0.00023	1.05	0.11	2.00E-03	0.39	0.04	3.51E-02	0.96	0.11	4.01E-03	0.37	0.11	2.52E-01	0.55	0.14	3.13E-02	0.29	0.20	2.17E-01	0.36	0.10	1.20E-01	0.64	0.03	9.59E-03
WWC3	No	No	No	Up	1.49	0.005065	validated	0.38	0.10	1.76E-01	0.66	0.04	1.87E-03	0.82	0.11	1.63E-02	0.48	0.12	1.74E-01	1.21	0.16	8.65E-03	0.80	0.22	1.12E-01	0.10	0.10	7.18E-01	0.17	0.03	4.35E-01
MAPK3	No	Yes	No	Up	1.49	0.029354	0.0041	1.19	0.11	8.88E-03	0.19	0.04	3.81E-01	0.24	0.10	6.25E-01	0.77	0.12	1.22E-01	0.58	0.14	3.45E-02	0.61	0.21	5.59E-02	0.79	0.10	1.33E-01	0.25	0.03	3.51E-01
ITGB1BP1	No	Yes	No	Up	1.49	0.00063	0.00859	0.50	0.10	1.09E-01	0.36	0.04	6.17E-02	0.51	0.11	6.05E-02	1.13	0.13	2.95E-03	0.78	0.15	2.06E-02	0.27	0.20	2.77E-01	0.55	0.10	6.02E-02	0.51	0.03	5.25E-03
TYRO3	No	No	No	Up	1.49	0.020325	validated	0.21	0.10	4.54E-01	0.09	0.04	5.80E-01	0.47	0.10	1.73E-01	0.60	0.12	7.21E-02	1.09	0.15	5.38E-03	1.38	0.25	1.21E-02	0.54	0.10	2.10E-01	0.23	0.03	2.01E-01
CENPE	No	No	No	Up	1.49	0.000138	0.00268	0.68	0.11	2.59E-02	0.73	0.04	2.91E-04	0.37	0.10	1.37E-01	0.39	0.12	1.95E-01	0.84	0.15	3.44E-02	0.25	0.20	3.99E-01	0.69	0.10	2.40E-02	0.67	0.03	1.62E-03
ZNF404	No	No	No	Up	1.49	0.00016	validated	0.60	0.10	2.66E-02	0.56	0.04	4.34E-03	0.88	0.11	2.66E-03	0.55	0.12	3.36E-02	0.19	0.14	7.09E-01	1.09	0.23	3.10E-01	0.08	0.10	7.36E-01	0.67	0.03	8.25E-05
SERTAD2	No	No	No	Up	1.49	0.001735	validated	1.01	0.11	1.10E-02	0.68	0.04	2.52E-03	0.64	0.11	2.68E-02	0.48	0.12	1.51E-01	0.16	0.14	3.91E-01	1.20	0.24	4.62E-03	0.06	0.10	8.15E-01	0.39	0.03	1.33E-01
SLC9A9	No	No	No	Up	1.49	0.002443	validated	0.14	0.10	6.44E-01	0.29	0.04	9.46E-02	0.07	0.10	8.37E-01	0.68	0.12	2.94E-02	1.42	0.17	4.80E-03	0.99	0.22	5.29E-02	0.40	0.10	3.60E-01	0.64	0.03	3.58E-04
SHC4	No	No	No	Up	1.49	0.001648	validated	0.74	0.11	1.65E-02	0.59	0.04	1.10E-02	0.65	0.11	3.85E-02	1.36	0.14	3.08E-04	0.23	0.14	7.96E-01	0.40	0.20	7.38E-01	0.31	0.10	5.48E-01	0.36	0.03	2.91E-02
RIOK1	No	No	No	Up	1.49	0.01228	validated	1.17	0.11	2.85E-03	0.07	0.04	7.35E-01	0.75	0.11	3.57E-02	0.74	0.12	7.23E-02	0.24	0.14	3.75E-01	0.36	0.20	2.97E-01	0.72	0.10	4.71E-02	0.59	0.03	8.62E-02
NXPH4	No	Yes	No	Up	1.50	9.84E-05	1.5E-19	0.26	0.10	4.64E-01	0.73	0.04	1.38E-03	0.14	0.10	7.06E-01	1.45	0.14	3.57E-05	0.19	0.14	3.93E-01	0.36	0.20	2.23E-01	0.68	0.10	1.01E-01	0.83	0.03	3.80E-04
MGC16121	No	No	No	Up	1.50	0.000875	0.03473	0.52	0.10	4.82E-02	0.28	0.04	4.90E-02	0.38	0.10	2.08E-01	0.86	0.12	3.49E-03	0.29	0.14	2.91E-01	1.24	0.24	7.82E-02	0.51	0.10	9.41E-02	0.56	0.03	1.32E-03
PRC1	No	Yes	No	Up	1.50	9.98E-05	0.00905	0.50	0.10	9.72E-02	0.48	0.04	9.31E-03	0.10	0.10	6.72E-01	0.38	0.11	2.46E-01	0.86	0.15	5.98E-03	0.74	0.21	2.56E-02	1.04	0.10	4.75E-04	0.56	0.03	5.24E-03
SLC35B4	No	No	No	Up	1.50	0.005622	validated	0.96	0.11	3.96E-02	0.70	0.04	4.13E-03	0.38	0.10	2.75E-01	0.29	0.11	4.52E-01	0.43	0.14	3.70E-01	0.49	0.21	3.52E-01	0.87	0.10	1.70E-02	0.53	0.03	7.99E-03
C19ORF52	No	Yes	No	Up	1.50	0.004836	1.5E-05	1.45	0.12	1.24E-03	0.71	0.04	4.64E-03	0.14	0.10	6.62E-01	0.98	0.13	5.82E-02	0.34	0.14	2.04E-01	0.34	0.20	2.12E-01	0.27	0.10	3.89E-01	0.42	0.03	6.80E-02
CDKN1B	No	No	No	Up	1.50	0.000321	validated	0.38	0.10	1.46E-01	0.34	0.04	3.97E-02	0.71	0.11	1.13E-02	0.60	0.12	5.49E-02	1.19	0.16	8.58E-04	0.49	0.21	1.21E-01	0.53	0.10	2.95E-02	0.41	0.03	4.62E-02
NRXN2	No	No	No	Up	1.50	0.000929	0.03212	0.10	0.10	7.61E-01	0.18	0.04	2.08E-01	0.21	0.10	3.72E-01	0.57	0.12	4.10E-02	0.88	0.15	1.27E-02	1.75	0.28	9.40E-04	0.44	0.10	1.67E-01	0.54	0.03	1.20E-03
TRPC1	No	No	No	Up	1.50	1.14E-05	0.00875	0.89	0.11	3.39E-03	0.55	0.04	1.98E-03	0.84	0.11	1.91E-03	1.00	0.13	1.05E-03	0.39	0.14	1.50E-01	0.07	0.20	7.18E-01	0.58	0.10	2.64E-02	0.35	0.03	3.33E-02
SPNS1	No	No	No	Up	1.50	0.014747	0.00343	0.66	0.11	9.81E-02	0.52	0.04	2.43E-02	0.40	0.10	3.61E-01	1.05	0.13	4.56E-02	0.38	0.14	1.45E-01	0.58	0.21	8.94E-02	0.58	0.10	1.55E-01	0.51	0.03	4.87E-02
RIN2	No	Yes	No	Up	1.50	0.004166	0.00033	0.79	0.11	1.58E-02	0.01	0.04	9.58E-01	1.14	0.11	5.40E-03	0.56	0.12	1.09E-01	0.91	0.15	5.67E-03	0.99	0.22	1.11E-02	0.20	0.10	4.63E-01	0.06	0.03	6.88E-01
SDC3	No	Yes	No	Up	1.50	0.004282	7.4E-05	0.62	0.10	3.46E-02	0.47	0.04	8.54E-03	0.76	0.11	1.27E-02	0.76	0.12	3.37E-02	0.50	0.14	5.13E-01	0.63	0.21	5.78E-01	0.80	0.10	1.24E-02	0.13	0.03	4.06E-01
MCM4	No	Yes	No	Up	1.50	0.000176	0.00451	1.02	0.11	3.79E-03	0.66	0.04	7.33E-04	0.53	0.11	6.43E-02	0.92	0.12	8.41E-03	0.29	0.14	1.83E-01	0.55	0.21	7.12E-02	0.37	0.10	1.40E-01	0.33	0.03	6.76E-02
BEST1	No	No	No	Up	1.50	0.015315	0.00546	0.39	0.10	2.36E-01	0.40	0.04	1.25E-01	0.04	0.10	9.21E-01	0.70	0.12	2.73E-02	1.04	0.15	1.95E-01	0.72	0.21	3.54E-01	0.84	0.10	4.74E-02	0.54	0.03	1.76E-03
DDX18	No	Yes	No	Up	1.50	0.005728	0.01308	0.75	0.11	3.78E-02	0.49	0.04	2.69E-02	0.46	0.10	1.86E-01	0.84	0.12	7.58E-02	0.82	0.15	1.23E-02	0.41	0.20	1.96E-01	0.24	0.10	3.05E-01	0.67	0.03	3.60E-02
C18ORF54	No	No	No	Up	1.50	0.00128	0.00034	0.05	0.10	8.50E-01	1.12	0.04	1.23E-05	0.68	0.11	2.50E-02	0.79	0.12	4.76E-02	0.60	0.14	3.39E-01	0.73	0.21	2.64E-01	0.49	0.10	7.10E-02	0.22	0.03	1.50E-01
SAMD14	No	No	No	Up	1.50	0.043073	validated	0.13	0.10	7.04E-01	0.62	0.04	1.14E-02	0.14	0.10	6.75E-01	0.45	0.12	1.24E-01	0.86	0.15	2.66E-01	1.05	0.23	2.09E-01	1.15	0.10	2.28E-02	0.29	0.03	7.68E-02
SRD5A1	No	Yes	No	Up	1.50	0.000918	4E-05	0.88	0.11	5.67E-03	0.46	0.04	2.70E-02	0.45	0.10	1.07E-01	1.34	0.14	4.75E-03	0.17	0.14	5.57E-01	0.24	0.20	5.07E-01	0.70	0.10	1.02E-02	0.45	0.03	2.47E-02
LPHN2	No	Yes	No	Up	1.50	4.80E-05	1.5E-19	0.89	0.11	1.72E-03	0.71	0.04	4.84E-04	0.60	0.11	2.43E-02	0.53	0.12	1.09E-01	0.61	0.14	4.77E-02	0.40	0.20	2.15E-01	0.76	0.10	3.32E-03	0.18	0.03	2.07E-01
FZD4	No	No	No	Up	1.50	0.000989	validated	0.27	0.10	4.07E-01	0.20	0.04	2.37E-01	0.45	0.10	1.53E-01	0.64	0.12	3.46E-02	1.15	0.16	5.72E-04	0.55	0.21	8.74E-02	1.06	0.10	9.80E-03	0.38	0.03	2.67E-02
SLC6A8	No	No	No	Up	1.50	0.002422	validated	1.12	0.11	4.95E-03	0.37	0.04	4.08E-02	0.54	0.11	6.32E-02	0.50	0.12	1.13E-01	0.86	0.15	1.18E-02	0.73	0.21	7.64E-02	0.42	0.10	9.57E-02	0.15	0.03	3.88E-01
EIF4A3	No	Yes	No	Up	1.50	0.003183	0.00185	0.43	0.10	1.50E-01	0.30	0.04	1.16E-01	0.65	0.11	7.69E-02	1.03	0.13	2.30E-02	0.76	0.15	7.72E-03	0.62	0.21	4.72E-02	0.08	0.10	7.42E-01	0.84	0.03	1.15E-02
TRIB2	No	Yes	No	Up	1.50	4.80E-05	9.4E-05	0.25	0.10	3.21E-01	0.76	0.04	1.69E-04	0.50	0.10	6.69E-02	0.75	0.12	1.30E-02	0.79	0.15	1.57E-02	0.93	0.22	1.25E-02	0.14	0.10	5.10E-01	0.59	0.03	2.68E-03
YPEL1	No	No	No	Up	1.50	0.001797	validated	0.32	0.10	2.37E-01	0.59	0.04	3.00E-03	1.02	0.11	3.46E-03	1.04	0.13	7.33E-03	0.19	0.14	5.54E-01	0.72	0.21	1.83E-01	0.61	0.10	5.18E-02	0.22	0.03	2.46E-01
VPS72	No	Yes	No	Up	1.50	0.011401	0.03937	1.11	0.11	1.41E-02	0.37	0.04	9.91E-02	0.39	0.10	2.29E-01	0.32	0.11	5.21E-01	0.92	0.15	1.32E-02	0.76	0.21	2.85E-02	0.32	0.10	3.05E-01	0.54	0.03	1.07E-01
RAD23A	No	Yes	No	Up	1.51	0.008309	0.00691	0.88	0.11	6.76E-02	0.60	0.04	1.17E-02	0.40	0.10	2.67E-01	0.98	0.13	6.90E-02	0.69	0.14	2.33E-02	0.09	0.20	6.01E-01	0.81	0.10	1.26E-02	0.26	0.03	3.52E-01
ITSN1	No	No	No	Up	1.51	0.000502	0.03542	0.77	0.11	5.35E-02	0.70	0.04	1.14E-03	0.36	0.10	1.84E-01	0.59	0.12	7.30E-02	0.64	0.14	3.28E-02	0.12	0.20	5.63E-01	1.35	0.11	4.28E-04	0.20	0.03	1.93E-01
MBD5	No	Yes	No	Up	1.51	0.01957	0.00296	0.98	0.11	4.77E-03	0.45	0.04	8.42E-02	0.85	0.11	2.48E-02	1.04	0.13	5.14E-03	0.72	0.14	4.52E-01	0.61	0.21	5.04E-01	0.03	0.10	9.02E-01	0.05	0.03	7.70E-01
RNASEH2A	No	Yes	No	Up	1.51	0.000732	0.00693	0.96	0.11	1.01E-02	0.79	0.04	3.64E-04	0.34	0.10	1.91E-01	0.36	0.11	3.27E-01	0.60	0.14	7.77E-02	0.90	0.22	3.77E-02	0.16	0.10	5.26E-01	0.62	0.03	8.65E-03

Supplementary Table 2. Details for 2,053 genes differentially expressed between EEC and NEEC, identified by microarray meta-analysis.

EEC - endometrioid endometrial cancer, NEEC - non-endometrioid endometrial cancer, SMD - Standardised Mean Difference, FDR - False Discovery Rate, Var - Variance

Gene	145 gene list	1253 gene list	Associated with EEC-specific survival?	Up/Down Regulated in NEEC	average standardised fold change	Microarray Meta FDR	RNASeq FDR	Individual microarray study results: TCGA			E-MTAB-2532			E-GEOD-56026			GSE32507			GSE24537			E-GEOD-23518			E-GEOD-17025			E-GEOD-2109		
								SMD	Var	P-value	SMD	Var	P-value	SMD	Var	P-value	SMD	Var	P-value	SMD	Var	P-value	SMD	Var	P-value	SMD	Var	P-value	SMD	Var	P-value
ZNF187	No	No	No	Up	1.51	0.014866	validated	0.60	0.10	6.17E-02	0.22	0.04	2.57E-01	0.79	0.11	3.88E-02	1.10	0.13	2.08E-02	0.30	0.14	3.21E-01	0.71	0.21	1.06E-01	0.34	0.10	3.12E-01	0.68	0.03	2.92E-02
XPO5	No	Yes	Yes	Up	1.51	0.002177	0.00023	1.18	0.11	4.89E-03	0.70	0.04	4.04E-03	1.06	0.11	4.56E-03	0.50	0.12	2.31E-01	0.30	0.14	3.12E-01	0.38	0.20	3.32E-01	0.14	0.10	6.48E-01	0.48	0.03	2.63E-02
MAGEE1	No	Yes	No	Up	1.51	0.001149	9.7E-05	0.68	0.11	5.48E-02	0.29	0.04	1.52E-01	0.85	0.11	9.97E-03	0.70	0.12	9.37E-02	0.83	0.15	2.42E-02	0.48	0.21	1.63E-01	0.36	0.10	1.22E-01	0.56	0.03	2.56E-03
EVC2	No	No	No	Up	1.51	0.002852	0.00603	0.41	0.10	1.17E-01	0.23	0.04	2.56E-01	0.88	0.11	1.20E-02	0.75	0.12	1.19E-02	0.66	0.14	5.19E-01	0.84	0.22	4.59E-01	0.36	0.10	2.43E-01	0.62	0.03	2.89E-04
NES	No	No	No	Up	1.51	3.34E-05	validated	0.09	0.10	7.31E-01	0.53	0.04	3.07E-03	0.46	0.10	9.78E-02	1.05	0.13	9.38E-04	1.28	0.16	3.53E-04	0.76	0.21	2.57E-02	0.10	0.10	7.12E-01	0.49	0.03	5.35E-03
HSPA12B	No	No	No	Up	1.51	0.000113	validated	0.43	0.10	2.11E-01	0.19	0.04	2.12E-01	0.41	0.10	2.21E-01	1.26	0.13	1.31E-04	1.02	0.15	3.45E-03	0.64	0.21	5.81E-02	0.21	0.10	5.40E-01	0.59	0.03	5.76E-04
HECW2	No	No	No	Up	1.51	0.008591	validated	0.47	0.10	1.78E-01	0.53	0.04	3.05E-02	0.28	0.10	2.90E-01	0.77	0.12	1.72E-02	1.34	0.16	4.73E-02	0.30	0.20	4.68E-01	0.89	0.10	7.66E-03	0.18	0.03	2.14E-01
ASB6	No	No	No	Up	1.51	0.014669	validated	1.27	0.12	4.67E-03	0.57	0.04	2.03E-02	0.52	0.11	1.95E-01	0.62	0.12	2.19E-01	0.12	0.14	5.73E-01	0.40	0.20	3.95E-01	0.96	0.10	2.23E-02	0.29	0.03	1.86E-01
ZNF570	No	No	No	Up	1.51	0.014322	validated	0.95	0.11	6.25E-03	0.50	0.04	4.64E-02	0.26	0.10	4.47E-01	0.61	0.12	1.21E-01	0.35	0.14	5.84E-01	0.91	0.22	3.24E-01	0.53	0.10	1.51E-01	0.67	0.03	7.98E-03
LRP12	No	No	No	Up	1.51	0.000384	0.04269	0.38	0.10	1.90E-01	1.01	0.04	2.43E-05	0.72	0.11	9.19E-03	1.02	0.13	7.43E-03	0.49	0.14	4.84E-01	0.52	0.21	4.80E-01	0.30	0.10	2.52E-01	0.34	0.03	4.20E-02
PELI2	No	Yes	No	Up	1.51	0.000108	1.5E-19	0.99	0.11	9.86E-04	0.42	0.04	1.63E-02	0.49	0.10	4.98E-02	1.07	0.13	2.45E-03	0.21	0.14	4.55E-01	0.75	0.21	7.37E-02	0.38	0.10	1.17E-01	0.45	0.03	8.93E-03
DDB1	No	No	No	Up	1.51	0.004798	validated	0.85	0.11	4.82E-02	0.67	0.04	1.19E-02	0.72	0.11	1.18E-01	0.20	0.11	6.76E-01	0.63	0.14	1.90E-02	0.82	0.22	1.20E-02	0.27	0.10	3.93E-01	0.61	0.03	6.09E-02
EMP1	No	Yes	No	Up	1.51	1.14E-05	0.00297	1.07	0.11	1.94E-04	0.69	0.04	1.96E-04	0.62	0.11	1.62E-02	0.36	0.11	1.99E-01	0.43	0.14	7.45E-02	0.83	0.22	1.13E-02	0.40	0.10	7.43E-02	0.37	0.03	3.97E-02
PDE3B	No	No	No	Up	1.51	0.015715	0.04499	0.05	0.10	8.68E-01	0.52	0.04	2.34E-02	0.71	0.11	2.56E-02	0.44	0.12	1.06E-01	0.86	0.15	1.07E-01	1.30	0.24	2.40E-01	0.54	0.10	1.00E-01	0.35	0.03	3.27E-02
MFN2	No	No	No	Up	1.51	0.004237	validated	0.18	0.10	6.48E-01	0.68	0.04	7.64E-03	0.53	0.11	1.71E-01	0.14	0.11	7.91E-01	0.80	0.15	4.54E-02	0.66	0.21	1.10E-01	1.67	0.11	1.19E-04	0.11	0.03	4.68E-01
LY6E	No	Yes	No	Up	1.51	9.89E-06	1.5E-19	1.12	0.11	2.60E-04	0.66	0.04	5.67E-04	0.91	0.11	1.35E-03	0.54	0.12	5.52E-02	0.81	0.15	3.69E-03	0.22	0.20	3.12E-01	0.22	0.10	3.40E-01	0.30	0.03	6.12E-02
FBXL16	No	Yes	No	Up	1.51	1.41E-05	1.5E-19	1.21	0.11	2.79E-05	0.61	0.04	3.29E-04	0.47	0.10	5.85E-02	0.04	0.11	8.79E-01	0.63	0.14	1.88E-01	0.44	0.20	4.19E-01	0.89	0.10	5.89E-03	0.49	0.03	9.73E-03
HOXA13	No	No	No	Up	1.51	4.80E-05	validated	0.15	0.10	5.33E-01	0.46	0.04	9.45E-03	0.09	0.10	7.48E-01	1.15	0.13	7.41E-05	0.99	0.15	1.25E-02	0.82	0.22	4.90E-02	0.64	0.10	6.29E-02	0.50	0.03	1.16E-03
ZNF568	No	Yes	No	Up	1.51	0.000847	0.00024	0.48	0.10	1.13E-01	0.27	0.04	1.86E-01	0.75	0.11	3.73E-02	1.97	0.16	3.63E-06	0.41	0.14	6.99E-01	0.07	0.20	9.50E-01	0.50	0.10	1.01E-01	0.36	0.03	2.40E-02
C12ORF53	No	No	No	Up	1.51	0.000642	validated	0.54	0.10	9.42E-02	0.06	0.04	6.36E-01	0.04	0.10	9.12E-01	1.38	0.14	2.27E-05	1.02	0.15	5.00E-02	0.85	0.22	2.03E-01	0.31	0.10	5.18E-01	0.59	0.03	3.52E-04
GPX7	No	No	No	Up	1.52	9.84E-05	validated	0.05	0.10	8.47E-01	0.78	0.04	3.25E-04	0.91	0.11	4.15E-03	0.85	0.12	9.50E-03	0.55	0.14	4.40E-02	0.95	0.22	9.37E-03	0.13	0.10	5.73E-01	0.58	0.03	2.06E-02
RPL39L	No	Yes	No	Up	1.52	1.14E-05	3.5E-05	1.01	0.11	4.10E-04	0.47	0.04	4.33E-03	0.73	0.11	1.05E-02	0.70	0.12	1.12E-02	0.44	0.14	8.40E-02	0.83	0.22	1.98E-02	0.11	0.10	6.49E-01	0.51	0.03	6.02E-03
CHRNB1	No	No	No	Up	1.52	0.001524	0.01137	0.79	0.11	2.69E-02	0.85	0.04	4.34E-04	0.63	0.11	9.92E-02	0.40	0.12	1.62E-01	0.53	0.14	2.00E-01	0.54	0.21	1.98E-01	0.58	0.10	3.04E-01	0.49	0.03	7.67E-03
WWTR1	No	No	No	Up	1.52	0.002815	validated	1.15	0.11	9.88E-04	0.37	0.04	4.44E-02	0.38	0.10	1.89E-01	0.95	0.13	3.42E-03	0.66	0.14	1.94E-01	0.77	0.21	2.35E-01	0.33	0.10	2.18E-01	0.20	0.03	2.45E-01
FYN	No	No	No	Up	1.52	0.000235	0.02417	0.34	0.10	1.98E-01	0.39	0.04	3.13E-02	0.58	0.11	2.97E-02	0.77	0.12	1.99E-02	1.40	0.17	4.78E-04	0.58	0.21	8.64E-02	0.21	0.10	3.69E-01	0.55	0.03	5.77E-03
CRABP1	No	No	No	Up	1.52	1.83E-19	0.00024	0.41	0.10	8.33E-02	1.03	0.04	4.54E-07	0.58	0.11	3.62E-02	0.99	0.13	1.11E-03	0.25	0.14	2.16E-01	0.74	0.21	2.37E-02	0.33	0.10	3.26E-01	0.48	0.03	1.05E-03
EVC	No	Yes	No	Up	1.52	0.003764	0.00098	0.65	0.11	3.61E-02	0.51	0.04	8.77E-03	0.51	0.11	8.81E-02	0.46	0.12	1.10E-01	1.22	0.16	1.44E-02	0.78	0.22	1.15E-01	0.61	0.10	4.23E-02	0.08	0.03	6.54E-01
ETNK2	No	No	No	Up	1.52	0.0071	0.02377	0.69	0.11	5.17E-02	0.24	0.04	2.53E-01	0.55	0.11	1.40E-01	1.19	0.13	9.10E-04	0.41	0.14	3.50E-01	0.72	0.21	1.62E-01	0.63	0.10	1.11E-01	0.39	0.03	5.92E-02
SLC4A1AP	No	Yes	No	Up	1.52	0.023395	0.00255	1.22	0.12	1.29E-02	0.75	0.04	8.73E-03	0.85	0.11	3.05E-02	0.77	0.12	1.30E-01	0.64	0.14	2.91E-01	0.22	0.20	5.49E-01	0.07	0.10	8.17E-01	0.30	0.03	2.16E-01
CACNA1G	No	No	No	Up	1.52	0.000475	validated	0.15	0.10	7.28E-01	0.76	0.04	1.19E-04	0.87	0.11	1.80E-02	1.09	0.13	5.01E-04	0.57	0.14	2.51E-01	0.75	0.21	1.91E-01	0.33	0.10	4.52E-01	0.31	0.03	6.58E-02
KPNA1	No	No	No	Up	1.52	0.00449	0.04772	0.79	0.11	6.37E-02	0.77	0.04	1.06E-02	0.63	0.11	6.21E-02	0.17	0.11	7.00E-01	0.38	0.14	2.34E-01	0.43	0.20	2.95E-01	1.25	0.10	8.22E-04	0.41	0.03	1.28E-01
PDPN	No	Yes	No	Up	1.52	8.12E-05	0.00064	0.60	0.10	2.60E-02	0.59	0.04	9.09E-04	0.58	0.11	3.49E-02	0.46	0.12	7.83E-02	0.61	0.14	3.56E-02	0.74	0.21	3.89E-02	1.15	0.10	7.49E-04	0.09	0.03	5.46E-01
ATP11B	No	Yes	Yes	Up	1.52	0.000524	1.5E-05	0.32	0.10	2.83E-01	0.24	0.04	1.46E-01	0.76	0.11	7.87E-03	0.80	0.12	2.35E-02	0.86	0.15	1.61E-01	0.67	0.21	2.52E-01	0.40	0.10	9.95E-02	0.78	0.03	4.45E-05
RAB42	No	No	No	Up	1.52	0.000734	0.0065	0.54	0.10	5.43E-02	0.34	0.04	3.80E-02	0.23	0.10	4.48E-01	1.01	0.13	5.12E-03	0.54	0.14	5.54E-01	0.16	0.20	8.29E-01	1.39	0.11	2.49E-04	0.62	0.03	5.68E-03
POLR3A	No	No	No	Up	1.52	0.005495	validated	1.00	0.11	3.65E-02	0.53	0.04	1.88E-02	0.65	0.11	6.80E-02	0.50	0.12	2.22E-01	0.55	0.14	1.05E-01	0.32	0.20	2.80E-01	1.00	0.10	1.36E-02	0.29	0.03	8.16E-02
CCNF	No	Yes	No	Up	1.52	0.000302	0.00053	0.85	0.11	1.45E-02	0.74	0.04	1.43E-03	0.35	0.10	2.50E-01	1.06	0.13	4.93E-03	0.78	0.15	2.03E-02	0.23	0.20	4.07E-01	0.39	0.10	2.45E-01	0.45	0.03	9.10E-03
EPM2AIP1	No	Yes	No	Up	1.52	5.41E-05	1.5E-19	0.39	0.10	1.07E-01	0.56	0.04	1.34E-03	0.61	0.11	2.76E-02	0.64	0.12	2.32E-02	0.51	0.14	7.01E-02	1.25	0.24	2.96E-03	0.48	0.10	3.59E-02	0.41	0.03	2.52E-02
KCNE2	No	Yes	No	Up	1.52	0.000482	1.5E-19	1.43	0.12	6.95E-05	0.56	0.04	3.31E-02	0.41	0.10	3.92E-01	0.85	0.12	7.76E-03	0.14	0.14	9.13E-01	0.29	0.20	8.16E-01	0.53	0.10	2.70E-01	0.64	0.03	8.22E-04
RARG	No	Yes	No	Up	1.52	0.009543	1.5E-19	0.91	0.11	1.58E-02	0.83	0.04	5.12E-04	0.23	0.10	5.39E-01	0.58	0.12	1.11E-01	0.46	0.14	7.12E-01	0.53	0.21	6.96E-01	1.08	0.10	3.93E-02	0.23	0.03	1.38E-01
CDC20	No	Yes	No	Up	1.52	3.34E-05	2E-05	0.86	0.11	6.11E-03	0.57	0.04	1.29E-03	0.30	0.10	1.97E-01	0.88	0.12	2.01E-02	0.89	0.15	2.69E-03	0.42	0.20	1.27E-01	0.48	0.10	5.90E-02	0.46	0.03	1.15E-02
MCM3	No	Yes	No	Up	1.52	0.000639	0.00709	0.90	0.11	1.63E-02	0.82	0.04	8.36E-04	0.22	0.10	4.48E-01	0.80	0.12	9.08E-02	0.60	0.14	2.32E-02	0.43	0.20	1.33E-01	0.38	0.10	1.73E-01	0.72	0.03	7.68E-03
TAF1A	No	No	No	Up	1.52	0.000915	0.01644	0.92	0.11	7.89E-03	0.34	0.04	7.82E-02	0.87	0.11	4.03E-03	0.71	0.12	6.79E-02	0.33	0.14	4.85E-01	0.76	0.21	2.12E-01	0.08	0.10	7.29E-01	0.87	0.03	4.31E-04
BRPF1	No	No	No	Up	1.52	0.010551	validated	0.47	0.10	2.70E-01	0.78	0.04	5.21E-03	0.20	0.10	6.16E-01	0.68	0.12	2.34E-01	0.49	0.14	6.31E-02	0.60	0.21	7.13E-02	1.43	0.11	3.79E-03	0.22	0.03	4.83E-01
ZNF689	No	No	No	Up	1.53	0.00466	0.00585	0.06	0.10	8.24E-01	0.21	0.04	2.38E-01	0.83	0.11	3.67E-02	1.53	0.14	5.35E-04	0.28	0.14	3.16E-01	1.11	0.23	1.55E-02	0.46	0.10	1.52E-01	0.39	0.03	8.15E-02
SLC7A8	No	No	No	Up	1.53	0.000274	validated	0.51	0.10	3.96E-02	0.58	0.04	8.80E-04	0.26	0.10	3.16E-01	0.40	0.12	1.39E-01	1.23	0.16	4.02E-03	0.85	0.22	4.23E-02	0.78	0.10	1.83E-02	0.25	0.03	8.53E-02
SPC25	No	Yes	No	Up	1.53	5.41E-05	6.5E-05	0.50	0.10	6.77E-02	0.63	0.04	4.50E-04	0.31	0.10	1.80E-01	0.75	0.12	1.60E-02	0.82	0.15	8.89E-02	0.45	0.20	3.29E-01	0.69	0.10	1.52E-02	0.73	0.03	5.04E-04
PRX	No	No	No	Up	1.53	0.017804	0.00034	0.33	0.10	4.03E-01	0.83	0.04	1.05E-04	0.16	0.10	7.59E-01	0.75	0.12	4.46E-02	0.97	0.15	2.19E-01	0.87	0.22	3.82E-01	0.93	0.10	5.88E-02	0.02	0.03	8.99E-01
CDCA3	No	No	No	Up	1.53	0.000235	0.02611	0.28	0.10	3.82E-01	0.69	0.04	5.17E-04	0.36	0.10	1.46E-01	0.69	0.12	6.21E-02	0.56	0.14	1.46E-01	0.78	0.22	1.06E-01	0.84	0.10	4.01E-03	0.68	0.03	2.87E-03
PBX4	No	No	No	Up	1.53	0.001124	0.00075	0.94	0.11	1.93E-03	0.63	0.04	8.50E-04	0.68	0.11	4.38E-02	0.16	0.11	6.01E-01	0.35	0.14	5.22E-01	1.11	0.23	2.05E-01	0.68	0.10	3.29E-02	0.33	0.03	5.97E-02
BSN	No	Yes	No	Up	1.53	0.001084	1.5E-19	0.85	0.11	5.49E-03	1.16	0.04	1.41E-05	0.27	0.10	4.32E-01	0.65	0.12	3.69E-02	0.47	0.14	3.59E-01	0.63	0.21	3.54E-01	0.75	0.10	1.05E-01	0.11	0.03	5.03E-01
ANAPC1	No	Yes	No	Up	1.53	0.004219	0.01159	0.73	0.11	9.93E-02	0.62	0.04	1.54E-02	1.11	0.11	2.04E-03	0.66	0.12	9.47E-02	0.53	0.14	1.04E-01	0.61	0.21	1.20E-01	0.00	0.10	9.90E-01	0.64	0.03	5.04E-02
ADARB1	No	Yes	No	Up	1.53	0.000105	0.00033	0.49	0.10	1.13E-01	0.60	0.04	1.62E-03	0.89	0.11	4.33E-03	0.75	0.12	3.59E-02	0.71	0.14	1.56E-02	0.51	0.21	9.93E-02	0.62	0.10	3.51E-02	0.32	0.03	4.34E-02
MYO9B	No	No	No	Up	1.53	0.003204	validated	0.85	0.11	5.57E-02	0.97	0.04	3.53E-04	0.57	0.11	9.22E-02	0.70	0.12	5.83E-02	0.57	0.14	9.54E-02	0.22	0.20	4.74E-01	0.77	0.10	8.91E-02	0.24	0.03	2.27E-01
SMAD3	No	Yes	No	Up	1.53	0.001208	0.00012	1.36	0.12	4.69E-04	0.50	0.04	1.19E-02	0.33	0.10	2.87E-01	0.42	0.12	2.00E-01	1.26	0.16	1.65E-03	0.45	0.21	2.11E-01	0.53	0.10	1.24E-01	0.05	0.03	7.62E-01
RSRC1	No	Yes	Yes	Up	1.53	6.46E-05	0.00021	0.66	0.11	1.40E-02	0.70	0.04	5.02E-04	0.03	0.10	8.98E-01	0.52	0.12	6.63E-02	0.83	0.15	2.26E-02	1.21	0.24	4.39E-03	0.67	0.10	1.13E-02	0.29	0.03	5.60E-02
PTTG1	No	No	No	Up	1.53	1.14E-05	0.00682	0.59	0.10	4.07E-02	0.83	0.04	2.84E-05	0.35	0.10	1.73E-01	0.58	0.12	5.88E-02	0.91	0.15	2.34E-03	0.78	0.22	1.83E-02	0.32	0.10	2.13E-01	0.56	0.03	6.95E-03
TMEM55A	No	No	No	Up	1.53	0.000294	validated	0.57	0.10	5.39E-02	0.21	0.04	1.92E-01	0.60	0.11	3.25E-02	1.04	0.13	1.91E-03	0.81	0.15	2.52E-02	0.38	0.20	2.52E-01	0.63	0.10	4.06E-02	0.67	0.03	2.72E-03
PIM1	No	No	No	Up	1.53	0.000718	0.0323	0.78	0.11	2.54E-02	0.45	0.04	1.11E-02	0.45	0.10	1.39E-01	0.38	0.11	2.54E-01	0.29	0.14	2.09E-01	1.05	0.23	1.07E-02	0.92	0.10	5.86E-03	0.59	0.03	2.26E-02
PTGES	No	Yes	No	Up	1.53	0.000415	1.5E-19	0.95	0.11	4.52E-03	0.28	0.04	2.27E-01	0.82	0.11	7.28E-03	0.61	0.12	3.47E-02	0.25	0.14	3.44E-01	0.32	0.20	2.87E-01	1.42	0.11	3.36E-04	0.27	0.03	1.04E-01
BRMS1L	No	No	No	Up	1.53	0.003726	0.00855	0.57	0.10	6.35E-02	0.32	0.04	7.94E-02	0.79	0.11	1.52E-02	0.85	0.12	9.24E-03	0.61	0.14	1.42E-01	1.24	0.24	3.45E-02	0.10	0.10	6.92E-01	0.45	0.03	5.67E-02
C1ORF135	No	Yes	No	Up	1.53	4.80E-05	1.5E-19	0.97	0.11	1.86E-03	0.89	0.04	1.29E-05	0.66	0.11	3.89E-02	0.33	0.11	2.89E-01	0.66	0.14	8.72E-02	0.32	0.20	3.95E-01	0.85	0.10	1.11E-02	0.24	0.03	1.18E-01
MAGEA10	No	Yes	No	Up	1.53	3.58E-06	1.5E-19	0.08	0.10	7.59E-01	0.72	0.04	3.00E-04	0.62	0.11	4.86E-02	0.65	0.12	3.71E-02	0.62	0.14	2.91E-02	0.65	0.21	4.23E-02	0.66	0.10	7.03E-02	0.92	0.03	1.14E-06
MCM8	No	Yes	No	Up	1.53	8.51E-05	9.1E-05	0.99	0.11	1.17E-02	1.16	0.04	2.73E-06	0.70	0.11	1.92E-02	0.85	0.12	3.92E-02	0.16	0.14	3.67E-01	0.11	0.20	5.67E-01	0.85	0.10	1.70E-02	0.12	0.03	4.56E-01
VCL	No	Yes	No	Up	1.53	0.000809	0.00717	1.33	0.12	2.54E-03	0.00	0.04	9.82E-01	0.61	0.11	4.72E-02	0.54	0.12	1.24E-01	0.39	0.14	8.86E-02	0.26	0.20	2.65E-01	1.60	0.11	6.84E-05	0.21	0.03	1.77E-01
MYLPF	No	Yes	No	Up	1.53	1.83E-19	1.5E-19	0.45	0.10	2.42E-01	0.45	0.04	1.39E-02	0.24	0.10	6.23E-01	0.82	0.12	1.57E-03	0.74	0.14	6.13E-02	0.64	0.21	1.09E-01	0.46	0.10	2.57E-01	1.13	0.04	1.00E-20
SEMA6D	No	Yes	No	Up	1.53	1.83E-19	1.5E-19	0.17	0.10	5.38E-01	0.70	0.04	2.64E-04	0.31	0.10	1.72E-01	1.35	0.14	2.84E-05	0.12	0.14	9.15E-01	0.35	0.20	7.78E-01	1.30	0.11	3.82E-05	0.65	0.03	4.29E-04
TSC22D3	No	Yes	No	Up	1.53	0.000235	3.8E-05	0.99	0.11	2.26E-03	0.12	0.04	3.97E-01	0.41	0.10	1.35E-01	1.27	0.13	7.19E-04	0.82	0.15	9.33E-03	0.78	0.22	2.48E-02	0.17	0.10	5.41E-01	0.39	0.03	2.91E-02
RNMT	No	Yes	No	Up	1.53	0.003061	0.00182	0.99	0.11	1.17E-02	0.68	0.04	1.11E-02	0.92	0.11	1.03E-02	1.06	0.13	3.95E-02	0.16	0.14	4.62E-01	0.26	0.20	3.51E-01	0.49	0.10	1.39E-01	0.39	0.03	7.37E-02
COL5A1	No	No	No	Up	1.54	9.89E-06	validated	0.33	0.10	2.55E-01	0.48	0.04	3.92E-03	0.41	0.10	9.41E-02	1.16	0.13	1.94E-04	0.59	0.14	2.13E-02	0.82	0.22	1.18E-02	0.52	0.10	5.18E-02	0.63	0.03	1.05E-03

Supplementary Table 2. Details for 2,053 genes differentially expressed between EEC and NEEC, identified by microarray meta-analysis.

EEC - endometrioid endometrial cancer, NEEC - non-endometrioid endometrial cancer, SMD - Standardised Mean Difference, FDR - False Discovery Rate, Var - Variance

Gene	145 gene list	1253 gene list	Associated with EEC-specific survival?	Up/Down Regulated in NEEC	average standardised fold change	Microarray Meta FDR	RNASeq FDR	Individual microarray study results: TCGA			E-MTAB-2532			E-GEOD-56026			GSE32507			GSE24537			E-GEOD-23518			E-GEOD-17025			E-GEOD-2109		
								SMD	Var	P-value	SMD	Var	P-value	SMD	Var	P-value	SMD	Var	P-value	SMD	Var	P-value	SMD	Var	P-value	SMD	Var	P-value	SMD	Var	P-value
SMG5	No	Yes	No	Up	1.54	0.001804	0.00116	1.45	0.12	8.74E-04	0.85	0.04	8.05E-04	1.02	0.11	1.26E-02	0.51	0.12	2.93E-01	0.50	0.14	1.04E-01	0.26	0.20	4.52E-01	0.20	0.10	5.33E-01	0.16	0.03	3.62E-01
TCP11L1	No	No	No	Up	1.54	0.001636	validated	0.41	0.10	1.90E-01	0.66	0.04	4.57E-03	0.66	0.11	4.31E-02	1.01	0.13	1.66E-02	0.78	0.15	1.92E-01	0.28	0.20	5.27E-01	0.64	0.10	5.05E-02	0.54	0.03	6.23E-03
MYL3	No	No	No	Up	1.54	0.00142	validated	0.66	0.11	1.06E-01	0.77	0.04	7.95E-04	0.13	0.10	8.06E-01	0.45	0.12	8.12E-02	0.92	0.15	3.65E-01	0.07	0.20	8.75E-01	1.25	0.10	4.00E-02	0.71	0.03	2.05E-04
EIF2AK2	No	Yes	No	Up	1.54	0.000594	2.6E-05	0.73	0.11	1.26E-02	0.20	0.04	2.24E-01	1.21	0.11	2.31E-03	1.15	0.13	2.03E-03	0.46	0.14	7.00E-02	0.55	0.21	8.13E-02	0.44	0.10	1.00E-01	0.23	0.03	2.38E-01
RUBCN	No	Yes	No	Up	1.54	0.009325	0.00086	0.50	0.10	2.31E-01	0.79	0.04	2.47E-03	1.07	0.11	7.57E-03	0.20	0.11	5.94E-01	0.47	0.14	5.70E-01	0.63	0.21	5.33E-01	1.15	0.10	5.18E-03	0.15	0.03	3.00E-01
MYLK2	No	No	No	Up	1.54	0.000754	0.00538	1.17	0.11	3.57E-04	0.72	0.04	1.64E-03	0.03	0.10	9.40E-01	0.82	0.12	1.04E-02	0.54	0.14	6.13E-01	0.71	0.21	5.88E-01	0.44	0.10	3.61E-01	0.54	0.03	4.48E-03
CLDN19	No	Yes	Yes	Up	1.54	1.14E-05	1.5E-19	1.98	0.14	4.54E-07	1.18	0.04	1.59E-05	0.29	0.10	4.12E-01	0.51	0.12	5.15E-02	0.29	0.14	4.16E-01	0.28	0.20	4.71E-01	0.06	0.10	9.07E-01	0.39	0.03	2.48E-02
YIPF4	No	Yes	No	Up	1.54	0.001811	0.00022	0.81	0.11	1.87E-02	0.43	0.04	2.79E-02	1.33	0.12	2.25E-03	0.82	0.12	2.18E-02	0.11	0.14	5.64E-01	0.96	0.22	2.45E-02	0.48	0.10	8.09E-02	0.03	0.03	8.29E-01
DGAT2	No	Yes	No	Up	1.54	0.000379	4.5E-05	0.63	0.10	2.79E-02	0.46	0.04	1.47E-02	0.88	0.11	9.47E-03	0.60	0.12	3.67E-02	0.56	0.14	2.84E-01	0.72	0.21	6.22E-01	0.44	0.10	1.93E-01	0.69	0.03	1.51E-04
POLD1	No	No	No	Up	1.54	0.000925	0.0289	0.29	0.10	3.83E-01	1.06	0.04	1.68E-05	0.29	0.10	3.68E-01	0.38	0.12	3.10E-01	0.92	0.15	4.03E-02	1.00	0.23	9.46E-02	0.33	0.10	3.21E-01	0.72	0.03	6.27E-03
KDELC1	No	No	No	Up	1.54	0.001833	0.02547	0.31	0.10	2.52E-01	0.45	0.04	2.67E-02	0.61	0.11	3.27E-02	1.30	0.14	1.12E-03	0.70	0.14	2.04E-01	0.83	0.22	1.74E-01	0.07	0.10	8.10E-01	0.71	0.03	3.46E-03
SOCS5	No	Yes	No	Up	1.54	0.012483	0.01312	0.30	0.10	3.11E-01	0.40	0.04	6.63E-02	1.15	0.11	2.87E-03	1.02	0.13	1.06E-02	0.10	0.14	7.57E-01	1.41	0.25	7.84E-02	0.31	0.10	2.40E-01	0.28	0.03	2.86E-01
UQCRFS1	No	Yes	No	Up	1.54	0.000159	1.5E-19	0.71	0.11	1.51E-02	0.21	0.04	1.72E-01	0.58	0.11	6.71E-02	1.24	0.13	6.31E-04	0.58	0.14	2.10E-02	0.78	0.22	1.45E-02	0.56	0.10	2.29E-02	0.32	0.03	1.77E-01
KCTD20	No	No	No	Up	1.54	0.001839	validated	0.15	0.10	6.88E-01	0.20	0.04	3.68E-01	0.10	0.10	7.13E-01	1.51	0.14	2.50E-03	1.18	0.16	1.06E-03	1.04	0.23	6.00E-03	0.50	0.10	4.96E-02	0.32	0.03	1.74E-01
IGSF1	No	Yes	No	Up	1.54	0.000125	1.5E-19	0.69	0.11	3.27E-02	0.64	0.04	5.26E-03	0.22	0.10	4.85E-01	0.84	0.12	7.64E-03	0.88	0.15	3.88E-02	0.89	0.22	4.57E-02	0.14	0.10	6.44E-01	0.69	0.03	1.33E-04
MYOZ3	No	No	No	Up	1.54	0.000188	0.02254	0.44	0.10	2.51E-01	0.63	0.04	4.88E-04	0.05	0.10	8.93E-01	1.87	0.16	1.59E-06	1.15	0.16	4.53E-02	0.64	0.21	1.48E-01	0.02	0.10	9.54E-01	0.19	0.03	2.04E-01
FAM20A	No	Yes	No	Up	1.54	4.80E-05	1.5E-19	1.10	0.11	1.30E-04	0.42	0.04	7.84E-03	0.52	0.11	2.30E-02	0.28	0.11	3.09E-01	1.01	0.15	9.03E-02	0.57	0.21	2.94E-01	0.85	0.10	7.13E-04	0.24	0.03	9.36E-02
BEX1	No	No	No	Up	1.54	1.83E-19	0.02357	0.13	0.10	5.54E-01	0.47	0.04	1.33E-03	0.89	0.11	8.30E-04	0.78	0.12	2.25E-03	0.56	0.14	3.22E-02	1.48	0.25	2.31E-04	0.02	0.10	9.29E-01	0.69	0.03	5.95E-05
MYBPH	No	No	No	Up	1.54	1.83E-19	validated	0.05	0.10	8.60E-01	0.59	0.04	9.18E-04	0.06	0.10	8.94E-01	1.38	0.14	8.86E-06	0.68	0.14	2.67E-01	0.43	0.20	4.00E-01	0.69	0.10	1.78E-01	1.13	0.04	1.00E-20
PCDHB6	No	Yes	No	Up	1.54	4.80E-05	5.1E-05	0.34	0.10	1.96E-01	0.42	0.04	7.59E-02	1.43	0.12	8.86E-06	0.63	0.12	3.82E-02	0.22	0.14	7.90E-01	0.88	0.22	1.27E-01	0.34	0.10	1.75E-01	0.76	0.03	1.71E-04
THY1	No	No	No	Up	1.54	3.11E-05	0.04072	0.59	0.10	4.39E-02	0.55	0.04	4.26E-03	0.43	0.10	1.05E-01	0.12	0.11	7.34E-01	1.06	0.15	8.62E-04	1.01	0.23	4.20E-03	0.61	0.10	3.87E-02	0.64	0.03	3.30E-03
PDLIM7	No	Yes	No	Up	1.54	0.000321	0.00143	0.67	0.11	6.10E-02	0.48	0.04	9.35E-03	0.52	0.11	6.94E-02	0.67	0.12	6.15E-02	0.42	0.14	7.45E-02	0.77	0.21	2.02E-02	1.07	0.10	8.80E-03	0.41	0.03	1.55E-02
STARD7	No	Yes	No	Up	1.54	0.006559	0.00031	1.06	0.11	1.56E-02	0.42	0.04	3.78E-02	0.89	0.11	5.94E-02	0.81	0.12	9.82E-02	0.02	0.14	8.31E-01	0.56	0.21	6.13E-02	1.00	0.10	6.80E-03	0.26	0.03	4.34E-01
KIF20A	No	Yes	No	Up	1.54	2.40E-05	0.00116	0.72	0.11	1.55E-02	0.67	0.04	5.42E-04	0.13	0.10	5.78E-01	0.88	0.12	7.64E-03	0.85	0.15	1.09E-02	0.65	0.21	6.54E-02	0.53	0.10	4.57E-02	0.59	0.03	3.52E-03
COL4A4	No	Yes	No	Up	1.54	0.00011	1.5E-19	0.29	0.10	2.01E-01	0.18	0.04	3.62E-01	0.89	0.11	2.37E-03	0.57	0.12	3.03E-02	0.44	0.14	6.24E-01	0.43	0.20	7.66E-01	1.92	0.12	4.54E-07	0.30	0.03	6.84E-02
DEPDC1	No	Yes	No	Up	1.55	4.80E-05	6.4E-05	0.58	0.10	3.61E-02	0.59	0.04	2.23E-03	0.58	0.11	2.18E-02	0.84	0.12	1.11E-02	0.61	0.14	1.93E-01	0.40	0.20	3.55E-01	0.86	0.10	2.08E-03	0.57	0.03	5.32E-03
RGS4	No	No	No	Up	1.55	1.83E-19	validated	0.59	0.10	5.19E-02	0.86	0.04	2.84E-05	0.77	0.11	4.48E-03	0.35	0.11	2.22E-01	0.72	0.14	2.64E-02	0.75	0.21	3.32E-02	0.23	0.10	2.64E-01	0.75	0.03	4.32E-06
ANLN	No	Yes	No	Up	1.55	6.46E-05	0.00191	0.73	0.11	1.85E-02	0.61	0.04	1.25E-03	0.22	0.10	3.39E-01	0.75	0.12	1.34E-02	0.73	0.14	9.87E-02	0.72	0.21	1.35E-01	0.74	0.10	3.75E-03	0.54	0.03	4.89E-03
SMC3	No	Yes	No	Up	1.55	0.001923	0.03333	0.88	0.11	2.05E-02	0.25	0.04	2.06E-01	0.39	0.10	1.72E-01	0.90	0.12	2.24E-02	1.34	0.16	5.90E-04	1.03	0.23	1.06E-02	0.20	0.10	3.47E-01	0.05	0.03	7.77E-01
BAP1	No	No	No	Up	1.55	0.017621	validated	0.82	0.11	3.10E-02	0.50	0.04	3.56E-02	0.09	0.10	7.88E-01	1.41	0.14	5.29E-03	0.91	0.15	5.48E-02	0.61	0.21	1.91E-01	0.49	0.10	2.71E-01	0.20	0.03	4.71E-01
NCAM1	No	No	No	Up	1.55	0.000191	0.00904	0.51	0.10	2.99E-01	0.22	0.04	4.36E-01	0.60	0.11	1.16E-02	0.70	0.12	2.79E-02	1.22	0.16	1.63E-03	1.12	0.23	7.24E-03	0.11	0.10	5.83E-01	0.57	0.03	7.93E-04
C8ORF76	No	No	No	Up	1.55	0.002356	0.01558	1.09	0.11	5.25E-03	0.62	0.04	8.59E-03	0.68	0.11	6.23E-02	1.53	0.14	5.71E-03	0.02	0.14	8.44E-01	0.03	0.20	8.54E-01	0.78	0.10	3.72E-02	0.30	0.03	1.05E-01
ZNFX1	No	Yes	No	Up	1.55	0.005471	0.0209	1.01	0.11	1.74E-02	0.17	0.04	3.79E-01	0.35	0.10	2.73E-01	0.79	0.12	3.73E-02	1.20	0.16	3.12E-03	0.40	0.20	2.24E-01	0.96	0.10	1.31E-02	0.17	0.03	5.41E-01
ABCF3	No	No	No	Up	1.55	0.016172	0.03279	1.49	0.12	5.01E-03	0.55	0.04	3.27E-02	0.40	0.10	3.20E-01	0.27	0.11	5.90E-01	0.86	0.15	1.01E-01	0.54	0.21	2.59E-01	0.08	0.10	8.01E-01	0.85	0.03	7.57E-03
KCTD12	No	Yes	No	Up	1.55	2.40E-05	2.6E-05	1.06	0.11	6.02E-04	0.12	0.04	4.52E-01	0.62	0.11	1.75E-02	1.12	0.13	1.03E-03	0.87	0.15	3.27E-03	0.78	0.22	1.88E-02	0.04	0.10	8.30E-01	0.45	0.03	1.72E-02
SIX2	No	No	No	Up	1.55	2.40E-05	validated	0.13	0.10	6.39E-01	0.65	0.04	2.78E-03	0.22	0.10	4.28E-01	1.37	0.14	7.43E-05	0.87	0.15	1.08E-01	0.83	0.22	1.23E-01	0.19	0.10	4.62E-01	0.80	0.03	1.07E-05
NR3C1	No	Yes	No	Up	1.55	4.80E-05	1.5E-19	0.23	0.10	3.95E-01	0.70	0.04	2.59E-04	0.37	0.10	1.16E-01	1.71	0.15	1.98E-05	0.49	0.14	4.68E-01	0.60	0.21	4.63E-01	0.41	0.10	9.46E-02	0.56	0.03	3.23E-03
ZNF384	No	No	No	Up	1.55	0.024343	validated	0.83	0.11	7.77E-02	0.41	0.04	9.12E-02	1.22	0.11	2.23E-02	0.38	0.11	4.55E-01	0.57	0.14	2.16E-01	0.19	0.20	5.82E-01	1.14	0.10	1.86E-02	0.32	0.03	8.38E-02
GRB2	No	No	No	Up	1.55	0.005033	0.02439	0.93	0.11	3.30E-02	0.10	0.04	5.67E-01	0.23	0.10	4.84E-01	1.14	0.13	1.15E-02	0.28	0.14	2.78E-01	0.59	0.21	1.21E-01	1.19	0.10	2.08E-03	0.60	0.03	3.80E-02
U2AF1L4	No	No	No	Up	1.55	0.025895	0.0147	0.64	0.11	4.98E-02	0.52	0.04	1.59E-02	0.24	0.10	4.79E-01	1.33	0.14	3.86E-03	1.24	0.16	1.39E-01	0.88	0.22	1.59E-01	0.19	0.10	6.09E-01	0.03	0.03	8.45E-01
GALNT8	No	Yes	No	Up	1.55	0.009893	1.5E-19	0.75	0.11	3.59E-02	0.76	0.04	6.76E-03	0.38	0.10	4.10E-01	0.89	0.12	5.21E-03	0.18	0.14	9.11E-01	0.90	0.22	7.22E-01	0.79	0.10	1.52E-01	0.43	0.03	2.76E-02
PTPRA	No	No	No	Up	1.55	0.001797	validated	0.96	0.11	3.11E-02	0.66	0.04	1.57E-02	1.13	0.11	2.11E-03	0.45	0.12	3.90E-01	0.14	0.14	4.57E-01	1.24	0.24	3.05E-03	0.18	0.10	5.28E-01	0.31	0.03	7.89E-02
TRIP13	No	Yes	No	Up	1.55	3.97E-05	1.5E-19	0.98	0.11	5.23E-03	0.51	0.04	1.09E-02	0.32	0.10	2.16E-01	0.55	0.12	9.54E-02	0.32	0.14	2.07E-01	0.96	0.22	1.75E-02	0.37	0.10	1.40E-01	1.05	0.04	1.61E-05
SCUBE3	No	No	No	Up	1.55	0.002356	0.00259	1.13	0.11	9.19E-04	0.35	0.04	1.07E-01	0.84	0.11	2.28E-03	0.81	0.12	1.02E-02	0.50	0.14	5.74E-01	1.05	0.23	3.28E-01	0.20	0.10	4.68E-01	0.19	0.03	2.21E-01
APLP1	No	Yes	Yes	Up	1.55	1.66E-05	1.5E-19	0.76	0.11	1.47E-02	1.03	0.04	8.97E-05	0.66	0.11	3.97E-02	0.05	0.11	8.72E-01	0.50	0.14	9.18E-02	0.63	0.21	6.49E-02	0.91	0.10	1.96E-02	0.55	0.03	4.75E-04
RMND5A	No	No	No	Up	1.55	0.002455	0.02792	1.02	0.11	4.42E-03	0.30	0.04	1.69E-01	0.59	0.11	8.71E-02	0.51	0.12	1.48E-01	0.43	0.14	4.25E-01	0.78	0.22	2.59E-01	1.13	0.10	7.49E-04	0.32	0.03	6.29E-02
COPZ2	No	No	No	Up	1.55	0.00079	validated	0.68	0.11	3.33E-02	0.25	0.04	1.10E-01	0.18	0.10	5.32E-01	1.39	0.14	4.04E-05	0.67	0.14	2.06E-01	0.87	0.22	1.92E-01	0.54	0.10	2.55E-01	0.51	0.03	4.75E-03
KLK7	No	Yes	No	Up	1.55	3.58E-06	1.5E-19	0.62	0.10	1.49E-02	0.69	0.04	7.95E-05	1.28	0.12	1.95E-04	0.16	0.11	4.81E-01	0.29	0.14	2.83E-01	0.27	0.20	3.31E-01	1.30	0.11	4.10E-03	0.48	0.03	1.90E-03
CPXM2	No	Yes	No	Up	1.56	1.83E-19	1.5E-19	1.01	0.11	1.23E-04	0.65	0.04	1.03E-04	0.76	0.11	6.59E-03	0.93	0.12	1.36E-03	0.93	0.15	3.38E-03	0.74	0.21	2.58E-02	0.01	0.10	9.82E-01	0.07	0.03	6.48E-01
HMGB2	No	No	No	Up	1.56	0.000108	0.03467	0.48	0.10	1.19E-01	0.64	0.04	1.56E-03	0.69	0.11	2.51E-02	0.76	0.12	2.96E-02	0.85	0.15	8.60E-03	0.54	0.21	8.87E-02	1.05	0.10	1.37E-03	0.11	0.03	4.92E-01
HDAC5	No	Yes	No	Up	1.56	0.002046	0.00719	0.79	0.11	3.57E-02	0.08	0.04	5.91E-01	0.55	0.11	1.22E-01	2.37	0.19	1.34E-05	0.55	0.14	2.79E-01	0.09	0.20	8.15E-01	0.14	0.10	6.83E-01	0.54	0.03	5.86E-03
C2ORF43	No	Yes	No	Up	1.56	2.85E-05	1.5E-19	1.05	0.11	2.89E-04	0.61	0.04	1.11E-03	0.79	0.11	4.10E-03	0.36	0.11	1.55E-01	0.69	0.14	1.63E-01	0.91	0.22	6.62E-02	0.28	0.10	2.01E-01	0.44	0.03	1.51E-02
SLC26A11	No	Yes	No	Up	1.56	0.00304	0.00018	0.61	0.10	5.66E-02	0.50	0.04	2.22E-02	0.92	0.11	1.19E-02	0.01	0.11	9.85E-01	1.22	0.16	2.38E-02	0.42	0.20	3.67E-01	0.73	0.10	4.15E-02	0.71	0.03	1.59E-02
ZNF135	No	Yes	No	Up	1.56	4.80E-05	2.3E-06	0.76	0.11	7.34E-03	0.38	0.04	1.84E-02	1.37	0.12	1.53E-04	0.23	0.11	3.11E-01	0.44	0.14	1.77E-01	1.12	0.23	1.92E-02	0.32	0.10	2.30E-01	0.49	0.03	2.34E-03
SIN3B	No	Yes	No	Up	1.56	0.000349	0.0205	1.15	0.11	4.02E-03	0.85	0.04	1.22E-04	0.39	0.10	3.15E-01	0.76	0.12	5.40E-02	0.48	0.14	1.29E-01	0.20	0.20	4.94E-01	1.17	0.10	2.21E-03	0.13	0.03	5.46E-01
ANKRD46	No	No	No	Up	1.56	0.001049	validated	0.67	0.11	4.25E-02	0.68	0.04	4.03E-03	0.69	0.11	4.29E-02	0.68	0.12	4.61E-02	0.84	0.15	2.24E-02	0.86	0.22	3.71E-02	0.30	0.10	2.48E-01	0.40	0.03	1.09E-01
CENPO	No	Yes	No	Up	1.56	0.000167	6E-05	0.37	0.10	2.21E-01	0.92	0.04	5.63E-05	0.47	0.10	1.21E-01	0.63	0.12	7.67E-02	0.53	0.14	4.12E-01	0.75	0.21	2.93E-01	0.72	0.10	3.19E-02	0.75	0.03	3.61E-04
MYO15A	No	No	No	Up	1.56	0.0024	0.00377	1.01	0.11	3.44E-03	0.71	0.04	5.77E-04	0.42	0.10	3.15E-01	0.53	0.12	5.61E-02	0.20	0.14	8.48E-01	0.86	0.22	5.67E-01	0.89	0.10	1.05E-01	0.51	0.03	2.67E-02
TUSC1	No	No	No	Up	1.56	2.40E-05	0.01977	0.87	0.11	1.27E-03	0.27	0.04	9.07E-02	0.84	0.11	2.75E-03	0.38	0.12	1.29E-01	1.14	0.16	8.54E-03	0.30	0.20	3.37E-01	0.77	0.10	4.16E-03	0.56	0.03	7.61E-03
MCM10	No	Yes	No	Up	1.56	1.14E-05	4.3E-05	0.96	0.11	2.56E-03	0.75	0.04	1.22E-04	0.42	0.10	7.58E-02	0.72	0.12	2.07E-02	0.62	0.14	1.22E-01	0.49	0.21	2.32E-01	0.63	0.10	1.68E-02	0.56	0.03	1.41E-03
LSS	No	No	No	Up	1.56	0.001768	validated	0.21	0.10	5.21E-01	0.35	0.04	8.56E-02	0.50	0.10	1.36E-01	0.37	0.11	2.72E-01	1.32	0.16	2.85E-03	1.07	0.23	2.21E-02	0.99	0.10	3.43E-03	0.34	0.03	6.35E-02
BNC2	No	No	No	Up	1.56	3.58E-06	0.00534	0.35	0.10	1.79E-01	0.02	0.04	8.41E-01	0.23	0.10	3.25E-01	1.83	0.16	4.54E-07	0.92	0.15	4.57E-03	0.59	0.21	6.70E-02	0.52	0.10	4.18E-02	0.70	0.03	2.32E-04
MDC1	No	No	No	Up	1.56	0.001452	0.03428	0.58	0.10	1.17E-01	0.83	0.04	7.01E-04	0.82	0.11	1.53E-02	0.64	0.12	1.34E-01	0.68	0.14	7.93E-02	0.91	0.22	2.84E-02	0.61	0.10	6.67E-02	0.09	0.03	5.98E-01
TMEM169	No	Yes	No	Up	1.57	0.002832	1.5E-19	0.29	0.10	3.00E-01	0.98	0.04	5.34E-04	0.57	0.11	1.05E-01	0.26	0.11	3.05E-01	0.77	0.15	2.66E-01	0.80	0.22	2.33E-01	1.27	0.10	1.30E-03	0.22	0.03	1.69E-01
RALYL	No	Yes	No	Up	1.57	0.001196	1.5E-19	0.56	0.10	5.91E-02	0.61	0.04	6.45E-03	0.66	0.11	4.97E-02	0.72	0.12	1.27E-02	0.87	0.15	1.06E-01	1.06	0.23	6.37E-02	0.36	0.10	2.14E-01	0.33	0.03	2.95E-02
GIT1	No	Yes	No	Up	1.57	0.01328	0.00758	0.56	0.10	1.50E-01	0.54	0.04	5.26E-02	0.31	0.10	5.24E-01	0.12	0.11	7.75E-01	1.33	0.16	7.11E-03	0.72	0.21	1.14E-01	1.22	0.10	1.63E-02	0.38	0.03	7.12E-02
SLC8A1	No	Yes	No	Up	1.57	4.80E-05	1.5E-19	0.71	0.11	1.28E-02	0.34	0.04	3.78E-02	0.70	0.11	9.19E-03	0.30	0.11	2.63E-01	0.95	0.15	1.70E-01	0.59	0.21	3.13E-01	1.07	0.10	2.10E-04	0.52	0.03	1.19E-03
BUB1	No	Yes	No	Up	1.57	2.19E-05	1.5E-19	1.07	0.11	1.21E-03	0.79	0.04	7.04E-05	0.46	0.10	6.04E-02	0.63	0.12	4.85E-02	0.73	0.14	4.23E-02	0.76	0.21	5.71E-02	0.73	0.10	5.48E-03	0.01	0.03	9.35E-01
ASPM	No	Yes	No	Up	1.57	3.58E-06	0.00012	0.41	0.10	1.75E-01	0.81	0.04	3.09E-05	0.55	0.11	2.54E-02	0.56	0.12	6.71E-02	0.90	0.15	1.48E-02	0.64	0.21	8.28E-02	0.62	0.10	1.57E-02	0.69	0.03	3.84E-04

Supplementary Table 2. Details for 2,053 genes differentially expressed between EEC and NEEC, identified by microarray meta-analysis.

EEC - endometrioid endometrial cancer, NEEC - non-endometrioid endometrial cancer, SMD - Standardised Mean Difference, FDR - False Discovery Rate, Var - Variance

Gene	145 gene list	1253 gene list	Associated with EEC-specific survival?	Up/Down Regulated in NEEC	average standardised fold change	Microarray Meta FDR	RNASeq FDR	Individual microarray study results																							
								TCGA			E-MTAB-2532			E-GEOD-56026			GSE32507			GSE24537			E-GEOD-23518			E-GEOD-17025			E-GEOD-2109		
								SMD	Var	P-value	SMD	Var	P-value	SMD	Var	P-value	SMD	Var	P-value	SMD	Var	P-value	SMD	Var	P-value	SMD	Var	P-value	SMD	Var	P-value
TRIM58	No	Yes	No	Up	1.57	0.013774	1.5E-19	0.44	0.10	1.16E-01	0.29	0.04	1.52E-01	0.82	0.11	1.23E-02	0.31	0.11	2.81E-01	0.35	0.14	7.26E-01	1.36	0.25	4.18E-01	1.18	0.10	4.47E-02	0.43	0.03	4.00E-03
GAB2	No	No	No	Up	1.57	0.000398	0.01969	0.55	0.10	1.35E-01	0.60	0.04	2.25E-03	0.63	0.11	5.26E-02	0.42	0.12	2.56E-01	0.98	0.15	1.93E-02	0.81	0.22	1.11E-01	0.50	0.10	1.11E-01	0.69	0.03	8.55E-04
AURKB	No	No	No	Up	1.57	0.000176	validated	0.70	0.11	2.79E-02	0.42	0.04	2.65E-02	0.13	0.10	5.77E-01	1.02	0.13	2.95E-03	0.76	0.15	1.84E-02	0.96	0.22	1.73E-02	0.84	0.10	2.27E-02	0.36	0.03	2.02E-02
SUPT5H	No	No	No	Up	1.57	0.000206	0.01708	0.70	0.11	7.02E-02	0.94	0.04	1.54E-04	0.20	0.10	6.42E-01	0.18	0.11	6.87E-01	1.31	0.16	2.44E-04	1.08	0.23	4.82E-03	0.07	0.10	8.01E-01	0.71	0.03	5.41E-02
COX4I2	No	No	No	Up	1.57	0.001131	0.01204	1.70	0.13	2.82E-05	0.49	0.04	2.77E-02	0.28	0.10	5.52E-01	0.63	0.12	3.72E-02	0.81	0.15	4.16E-02	0.30	0.20	3.82E-01	0.65	0.10	1.90E-01	0.34	0.03	1.90E-01
N4BP3	No	Yes	No	Up	1.57	0.004452	1.5E-19	0.63	0.11	3.62E-02	0.87	0.04	2.52E-05	0.91	0.11	5.55E-02	0.50	0.12	9.81E-02	0.61	0.14	6.03E-01	0.86	0.22	5.39E-01	0.71	0.10	2.27E-01	0.10	0.03	5.69E-01
DAGLA	No	Yes	No	Up	1.57	0.001348	0.00024	0.97	0.11	1.66E-03	0.86	0.04	1.88E-03	0.18	0.10	7.02E-01	0.41	0.12	1.67E-01	0.87	0.15	1.90E-01	0.76	0.21	2.52E-01	0.64	0.10	2.04E-01	0.51	0.03	3.69E-03
ADD2	No	Yes	No	Up	1.57	1.41E-05	1.5E-19	0.84	0.11	5.87E-03	0.81	0.04	1.44E-03	1.19	0.11	8.99E-04	0.76	0.12	1.24E-02	0.12	0.14	9.08E-01	0.29	0.20	8.33E-01	0.56	0.10	1.64E-01	0.63	0.03	1.81E-04
KRI1	No	Yes	No	Up	1.57	0.000951	0.00471	0.97	0.11	1.61E-02	0.87	0.04	1.32E-04	0.92	0.11	2.61E-02	0.17	0.11	6.77E-01	0.59	0.14	1.81E-01	0.65	0.21	1.14E-01	0.79	0.10	3.66E-02	0.24	0.03	2.20E-01
EPB41L1	No	Yes	No	Up	1.57	0.000107	1.5E-19	0.90	0.11	3.92E-03	0.46	0.04	1.17E-02	0.54	0.11	5.83E-02	0.13	0.11	7.02E-01	1.07	0.15	1.01E-01	0.20	0.20	5.99E-01	1.66	0.11	5.91E-06	0.26	0.03	1.01E-01
DSCR8	No	Yes	No	Up	1.57	1.83E-19	1.5E-19	0.53	0.10	1.76E-02	0.69	0.04	3.34E-05	0.12	0.10	5.25E-01	1.37	0.14	4.09E-06	0.62	0.14	4.07E-02	0.99	0.22	1.14E-02	0.12	0.10	5.50E-01	0.76	0.03	1.59E-06
EPHB6	No	Yes	No	Up	1.57	0.001163	1.5E-19	1.07	0.11	2.94E-03	0.30	0.04	6.84E-02	0.98	0.11	5.96E-03	0.52	0.12	1.02E-01	1.01	0.15	8.75E-03	0.81	0.22	9.46E-02	0.27	0.10	5.06E-01	0.25	0.03	1.93E-01
COL9A1	No	Yes	No	Up	1.57	1.83E-19	1.5E-19	0.72	0.11	2.11E-03	1.00	0.04	4.54E-07	0.86	0.11	1.11E-03	0.39	0.12	1.06E-01	0.44	0.14	1.10E-01	0.94	0.22	1.20E-02	0.65	0.10	1.31E-02	0.21	0.03	1.77E-01
FOXP4	No	Yes	No	Up	1.57	0.002519	1.5E-19	1.59	0.12	1.18E-03	0.61	0.04	3.27E-03	0.33	0.10	3.30E-01	0.41	0.12	2.20E-01	0.20	0.14	5.25E-01	1.12	0.23	1.64E-02	0.77	0.10	7.09E-02	0.19	0.03	3.10E-01
PRKDC	No	Yes	No	Up	1.57	0.000108	0.00132	0.66	0.11	6.89E-02	0.85	0.04	1.16E-04	0.96	0.11	4.55E-03	1.33	0.14	2.04E-03	0.44	0.14	1.19E-01	0.60	0.21	1.07E-01	0.07	0.10	7.40E-01	0.30	0.03	1.06E-01
USE1	No	No	No	Up	1.57	0.01219	0.02831	1.43	0.12	4.57E-03	0.56	0.04	1.26E-02	0.93	0.11	1.23E-02	0.44	0.12	2.46E-01	0.94	0.15	1.63E-01	0.63	0.21	3.32E-01	0.02	0.10	9.57E-01	0.27	0.03	2.67E-01
FXR1	No	Yes	Yes	Up	1.57	0.000613	0.00711	0.95	0.11	2.58E-02	0.08	0.04	6.80E-01	0.74	0.11	2.37E-02	0.86	0.12	4.02E-02	0.49	0.14	7.06E-02	0.79	0.22	2.19E-02	0.95	0.10	2.89E-03	0.36	0.03	2.74E-02
TOP2A	No	Yes	No	Up	1.57	6.71E-06	0.00056	0.23	0.10	3.97E-01	0.39	0.04	1.20E-02	0.35	0.10	1.29E-01	1.09	0.13	6.33E-04	1.16	0.16	4.48E-04	0.90	0.22	9.20E-03	0.71	0.10	6.01E-03	0.40	0.03	1.71E-02
DCLK2	No	No	No	Up	1.57	0.005239	validated	0.16	0.10	6.70E-01	0.78	0.04	5.84E-04	0.51	0.10	1.63E-01	0.71	0.12	2.31E-02	0.61	0.14	4.30E-01	1.24	0.24	3.03E-01	0.92	0.10	2.78E-02	0.30	0.03	5.67E-02
ACOT7	No	Yes	Yes	Up	1.57	1.14E-05	0.00086	0.81	0.11	1.42E-02	0.89	0.04	2.95E-05	0.67	0.11	2.74E-02	0.64	0.12	5.01E-02	0.94	0.15	3.69E-03	0.53	0.21	8.30E-02	0.06	0.10	8.17E-01	0.69	0.03	2.39E-03
ZNF167	No	Yes	No	Up	1.57	0.000159	0.00015	0.69	0.11	3.58E-02	0.75	0.04	4.04E-04	0.76	0.11	1.07E-02	0.20	0.11	4.54E-01	0.02	0.14	9.57E-01	1.68	0.27	3.63E-02	0.68	0.10	7.32E-03	0.47	0.03	7.47E-03
TRAIP	No	Yes	No	Up	1.57	5.41E-05	4E-05	1.05	0.11	6.43E-03	0.98	0.04	1.41E-05	0.43	0.10	2.02E-01	0.57	0.12	1.29E-01	0.05	0.14	9.09E-01	0.83	0.22	3.40E-01	0.66	0.10	9.32E-02	0.66	0.03	2.63E-04
RBL1	No	No	No	Up	1.57	0.002726	0.01236	0.54	0.10	1.66E-01	0.58	0.04	2.63E-02	0.40	0.10	1.46E-01	0.66	0.12	5.60E-02	0.60	0.14	3.81E-01	1.02	0.23	3.13E-01	1.20	0.10	1.11E-04	0.25	0.03	1.37E-01
RAB6B	No	No	No	Up	1.58	1.14E-05	0.00411	0.21	0.10	4.84E-01	0.82	0.04	7.25E-05	0.58	0.11	3.32E-02	0.99	0.13	1.49E-03	0.33	0.14	4.03E-01	0.66	0.21	1.87E-01	1.15	0.10	2.68E-03	0.50	0.03	3.72E-03
NUP107	No	Yes	No	Up	1.58	0.000334	0.00529	0.65	0.11	9.64E-02	0.98	0.04	1.02E-04	0.54	0.11	1.30E-01	0.06	0.11	9.03E-01	0.59	0.14	7.67E-02	0.87	0.22	3.90E-02	0.61	0.10	5.70E-02	0.95	0.03	2.94E-03
NPY	No	Yes	No	Up	1.58	3.58E-06	1.5E-19	0.82	0.11	1.45E-03	0.88	0.04	2.95E-05	0.03	0.10	9.48E-01	0.52	0.12	6.17E-02	0.79	0.15	4.18E-03	0.65	0.21	2.96E-02	1.04	0.10	5.42E-02	0.52	0.03	3.07E-03
DEPDC1B	No	Yes	No	Up	1.58	3.58E-06	2.3E-06	0.92	0.11	2.57E-03	0.81	0.04	5.50E-05	0.50	0.10	4.16E-02	0.35	0.11	1.85E-01	0.76	0.15	1.26E-01	0.27	0.20	6.07E-01	1.15	0.10	7.61E-05	0.49	0.03	6.95E-03
NAP1L3	No	No	No	Up	1.58	9.84E-05	0.00175	0.50	0.10	5.86E-02	0.49	0.04	6.41E-03	0.67	0.11	7.61E-03	0.72	0.12	1.09E-02	0.69	0.14	1.50E-01	1.12	0.23	1.51E-01	0.35	0.10	1.50E-01	0.72	0.03	5.02E-04
ZBTB10	No	Yes	No	Up	1.58	0.000531	0.00658	0.59	0.10	1.68E-01	0.37	0.04	4.89E-02	1.08	0.11	1.74E-03	0.90	0.12	1.59E-02	0.67	0.14	2.72E-01	0.22	0.20	7.93E-01	1.38	0.11	4.13E-05	0.05	0.03	7.33E-01
NT5M	No	No	No	Up	1.58	0.020009	validated	0.21	0.10	4.65E-01	0.44	0.04	2.60E-02	1.03	0.11	2.17E-02	0.56	0.12	8.22E-02	0.83	0.15	3.28E-01	0.81	0.22	2.98E-01	1.20	0.10	1.56E-02	0.17	0.03	2.62E-01
BARX1	No	No	Yes	Up	1.58	1.14E-05	0.0003	0.59	0.10	1.59E-02	0.72	0.04	8.84E-05	0.08	0.10	7.61E-01	0.84	0.12	2.38E-03	0.78	0.15	2.79E-01	0.74	0.21	1.19E-01	0.92	0.10	3.21E-02	0.60	0.03	2.62E-04
HSPB2	No	No	No	Up	1.58	1.83E-19	5.5E-05	1.09	0.11	2.39E-04	0.57	0.04	6.94E-04	0.48	0.10	1.12E-01	0.65	0.12	1.15E-02	0.72	0.14	3.15E-02	0.24	0.20	3.71E-01	0.77	0.10	9.86E-02	0.73	0.03	1.16E-05
RAD51AP1	No	Yes	No	Up	1.58	3.58E-06	4.6E-06	0.82	0.11	6.43E-03	0.40	0.04	1.58E-02	0.36	0.10	1.44E-01	1.02	0.13	1.19E-03	0.31	0.14	2.28E-01	0.70	0.21	5.36E-02	0.84	0.10	2.09E-03	0.80	0.03	7.93E-05
GNAL	No	No	No	Up	1.58	0.000935	0.00041	1.08	0.11	5.86E-03	0.54	0.04	1.68E-02	0.08	0.10	7.16E-01	1.34	0.14	1.49E-04	0.71	0.14	4.58E-01	0.86	0.22	4.11E-01	0.19	0.10	5.13E-01	0.45	0.03	5.77E-03
NDRG4	No	Yes	No	Up	1.58	6.71E-06	4.3E-05	0.58	0.10	2.44E-02	1.14	0.04	6.82E-07	0.84	0.11	2.58E-02	0.27	0.11	3.48E-01	0.77	0.15	1.95E-02	0.89	0.22	1.85E-02	0.27	0.10	3.56E-01	0.49	0.03	4.66E-03
CRKL	No	No	No	Up	1.58	0.000754	0.0007	0.70	0.11	5.87E-02	0.28	0.04	1.89E-01	0.58	0.11	7.76E-02	0.39	0.12	2.80E-01	1.20	0.16	1.03E-03	1.11	0.23	5.93E-03	0.40	0.10	1.59E-01	0.59	0.03	1.44E-02
ATP8B2	No	Yes	No	Up	1.58	0.000349	0.0101	1.00	0.11	5.12E-03	0.70	0.04	5.35E-04	0.49	0.10	6.65E-02	0.67	0.12	5.24E-02	0.71	0.14	8.12E-02	1.20	0.24	5.99E-02	0.03	0.10	8.92E-01	0.47	0.03	1.57E-02
ASTE1	No	No	No	Up	1.58	0.024629	validated	1.11	0.11	1.20E-02	0.04	0.04	8.28E-01	0.58	0.11	1.47E-01	0.97	0.13	5.41E-02	0.02	0.14	9.50E-01	1.14	0.23	1.14E-01	1.14	0.10	6.02E-03	0.27	0.03	2.81E-01
FBN3	No	Yes	No	Up	1.58	5.41E-05	1.5E-19	0.54	0.10	1.10E-01	1.12	0.04	1.00E-05	0.92	0.11	1.16E-02	0.12	0.11	7.23E-01	0.12	0.14	7.26E-01	1.31	0.24	2.43E-02	0.59	0.10	8.61E-02	0.55	0.03	1.43E-03
CENPL	No	No	No	Up	1.58	0.000349	0.00752	0.84	0.11	1.30E-02	0.61	0.04	8.31E-03	0.18	0.10	4.83E-01	1.31	0.14	2.83E-03	0.46	0.14	1.90E-01	0.47	0.21	2.21E-01	0.77	0.10	1.78E-02	0.64	0.03	5.93E-03
BYSL	No	No	No	Up	1.58	0.001099	0.00213	1.06	0.11	2.33E-02	0.95	0.04	4.57E-04	0.88	0.11	1.96E-02	0.62	0.12	1.86E-01	0.11	0.14	5.45E-01	0.69	0.21	6.45E-02	0.41	0.10	2.21E-01	0.55	0.03	2.86E-02
DMRT1	No	Yes	No	Up	1.58	1.83E-19	1.5E-19	0.71	0.11	4.59E-03	0.70	0.04	2.22E-03	1.11	0.11	2.46E-04	1.08	0.13	7.72E-04	0.21	0.14	6.44E-01	0.25	0.20	6.00E-01	0.63	0.10	1.45E-02	0.60	0.03	2.78E-04
CHODL	No	Yes	Yes	Up	1.58	3.58E-06	4.3E-05	1.05	0.11	3.80E-04	0.65	0.04	2.29E-04	0.36	0.10	1.47E-01	0.87	0.12	3.31E-03	0.74	0.14	2.03E-02	0.69	0.21	4.54E-02	0.44	0.10	1.18E-01	0.48	0.03	3.03E-03
GDPD5	No	Yes	No	Up	1.58	0.000175	1.5E-19	1.53	0.12	1.84E-04	0.61	0.04	2.37E-03	0.83	0.11	1.49E-02	0.34	0.11	2.41E-01	0.82	0.15	4.07E-02	0.37	0.20	2.83E-01	0.48	0.10	1.47E-01	0.31	0.03	6.77E-02
ZNF585A	No	No	No	Up	1.58	0.000709	0.01386	0.71	0.11	7.49E-02	0.34	0.04	1.11E-01	0.56	0.11	1.03E-01	1.07	0.13	2.75E-03	0.24	0.14	4.76E-01	0.53	0.21	2.57E-01	1.17	0.10	1.21E-03	0.67	0.03	8.06E-03
UNKL	No	No	No	Up	1.58	0.008941	0.00899	0.38	0.10	2.60E-01	0.42	0.04	3.36E-02	0.63	0.11	5.50E-02	0.54	0.12	1.31E-01	1.30	0.16	3.70E-02	1.09	0.23	8.90E-02	0.68	0.10	4.88E-02	0.25	0.03	1.08E-01
CXORF57	No	No	No	Up	1.58	5.83E-05	0.00289	0.34	0.10	1.70E-01	0.19	0.04	2.27E-01	0.46	0.10	1.20E-01	0.75	0.12	7.19E-03	0.98	0.15	7.21E-02	0.86	0.22	1.14E-01	0.96	0.10	1.35E-02	0.75	0.03	5.91E-06
DNAJC18	No	No	No	Up	1.58	0.000661	0.00482	0.41	0.10	1.39E-01	0.73	0.04	1.14E-03	0.42	0.10	2.01E-01	1.20	0.13	2.79E-03	0.48	0.14	4.04E-01	0.70	0.21	3.33E-01	0.63	0.10	9.21E-02	0.74	0.03	2.23E-03
YBX2	No	Yes	No	Up	1.58	1.83E-19	1.5E-19	1.24	0.12	6.88E-05	1.32	0.04	1.00E-20	0.55	0.11	9.58E-02	0.81	0.12	6.31E-03	0.40	0.14	2.95E-01	0.07	0.20	7.79E-01	0.26	0.10	3.74E-01	0.66	0.03	1.90E-04
FOXRED2	No	No	No	Up	1.59	9.46E-05	validated	1.28	0.12	2.02E-03	1.02	0.04	1.77E-05	0.55	0.11	7.43E-02	0.96	0.13	6.70E-03	0.82	0.15	4.91E-02	0.35	0.20	2.53E-01	0.25	0.10	3.87E-01	0.08	0.03	5.96E-01
WDR67	No	No	No	Up	1.59	0.000526	0.0011	0.93	0.11	8.65E-03	0.42	0.04	2.27E-02	0.68	0.11	2.67E-02	0.45	0.12	8.52E-02	0.72	0.14	1.26E-01	1.20	0.24	2.74E-02	0.50	0.10	7.74E-02	0.41	0.03	1.19E-02
SF3B2	No	No	No	Up	1.59	0.007217	validated	0.82	0.11	1.85E-01	0.54	0.04	6.11E-02	0.55	0.11	1.48E-01	0.58	0.12	3.51E-01	1.04	0.15	2.61E-03	1.41	0.25	1.80E-03	0.21	0.10	4.98E-01	0.17	0.03	4.69E-01
KIF1B	No	Yes	No	Up	1.59	2.19E-05	0.00072	0.60	0.10	7.08E-02	0.49	0.04	1.12E-02	1.02	0.11	2.65E-03	1.56	0.14	3.20E-05	0.39	0.14	1.11E-01	0.00	0.20	9.86E-01	0.91	0.10	7.26E-03	0.36	0.03	4.99E-02
FAM131A	No	Yes	No	Up	1.59	0.002982	0.0026	1.35	0.12	4.79E-03	0.50	0.04	2.34E-02	0.64	0.11	5.30E-02	1.07	0.13	1.99E-02	0.41	0.14	1.87E-01	0.59	0.21	1.29E-01	0.61	0.10	9.27E-02	0.16	0.03	3.06E-01
C15ORF42	No	Yes	Yes	Up	1.59	2.40E-05	0.00104	0.68	0.11	1.87E-02	0.79	0.04	3.61E-05	0.08	0.10	8.03E-01	0.38	0.11	1.81E-01	0.72	0.14	2.76E-01	0.84	0.22	2.18E-01	1.22	0.10	6.33E-04	0.63	0.03	8.91E-04
CENPF	No	Yes	No	Up	1.59	3.58E-06	0.00048	0.84	0.11	4.78E-03	0.70	0.04	1.53E-04	0.42	0.10	9.58E-02	0.69	0.12	3.24E-02	0.67	0.14	2.65E-02	0.78	0.22	2.69E-02	0.47	0.10	6.08E-02	0.77	0.03	2.46E-04
KIF4A	No	Yes	No	Up	1.59	8.12E-05	0.00042	1.21	0.11	6.17E-04	0.39	0.04	2.01E-02	0.17	0.10	4.60E-01	0.76	0.12	1.76E-02	0.98	0.15	2.45E-02	0.72	0.21	9.81E-02	0.46	0.10	7.17E-02	0.65	0.03	2.14E-03
ADORA1	No	No	No	Up	1.59	0.000107	9.4E-05	0.96	0.11	3.12E-03	0.32	0.04	8.58E-02	1.30	0.12	2.74E-04	0.38	0.11	1.73E-01	1.15	0.16	9.67E-03	0.32	0.20	3.42E-01	0.29	0.10	5.13E-01	0.63	0.03	3.19E-03
MEA1	No	No	No	Up	1.59	0.006192	validated	1.09	0.11	3.00E-02	0.71	0.04	1.16E-02	0.83	0.11	5.22E-02	0.65	0.12	1.83E-01	0.99	0.15	6.89E-03	0.54	0.21	1.32E-01	0.41	0.10	2.21E-01	0.12	0.03	6.74E-01
HRASLS	No	Yes	No	Up	1.59	1.83E-19	1.5E-19	0.79	0.11	1.30E-03	0.71	0.04	7.75E-05	1.00	0.11	2.56E-04	0.75	0.12	5.77E-03	0.35	0.14	3.33E-01	0.62	0.21	1.31E-01	0.43	0.10	6.20E-02	0.70	0.03	1.12E-04
CLIP3	No	Yes	No	Up	1.59	9.89E-06	0.00068	1.33	0.12	3.40E-04	0.33	0.04	3.63E-02	0.50	0.10	9.49E-02	0.70	0.12	2.09E-02	0.67	0.14	1.26E-02	0.67	0.21	3.10E-02	0.50	0.10	1.58E-01	0.64	0.03	1.67E-04
VSTM2L	No	Yes	No	Up	1.59	0.00011	6.7E-06	0.06	0.10	8.32E-01	0.86	0.04	1.44E-04	0.17	0.10	6.27E-01	1.22	0.13	8.41E-05	0.68	0.14	4.11E-01	0.83	0.22	3.53E-01	0.96	0.10	5.46E-02	0.58	0.03	1.51E-03
CCDC88A	No	No	No	Up	1.59	3.58E-06	validated	0.52	0.10	7.41E-02	0.90	0.04	1.05E-05	0.50	0.10	4.90E-02	2.11	0.17	1.36E-06	0.31	0.14	5.25E-01	0.23	0.20	5.95E-01	0.45	0.10	5.81E-02	0.33	0.03	3.59E-02
GINS4	No	Yes	No	Up	1.59	8.89E-05	4E-05	1.06	0.11	2.45E-03	0.74	0.04	6.67E-04	0.17	0.10	5.16E-01	0.96	0.13	5.66E-03	0.27	0.14	3.99E-01	0.79	0.22	1.79E-01	0.92	0.10	2.56E-02	0.47	0.03	5.02E-03
OSER1	No	No	No	Up	1.59	0.001163	0.00053	0.94	0.11	1.39E-02	0.75	0.04	2.41E-03	0.86	0.11	2.79E-02	0.84	0.12	4.69E-02	0.25	0.14	2.76E-01	0.68	0.21	7.40E-02	0.58	0.10	9.06E-02	0.48	0.03	1.21E-01
ALPK2	No	No	No	Up	1.59	1.83E-19	0.0089	1.09	0.11	2.96E-04	0.74	0.04	4.20E-04	0.34	0.10	2.33E-01	1.00	0.13	6.95E-04	0.77	0.15	3.32E-02	0.20	0.20	5.40E-01	0.27	0.10	3.82E-01	0.97	0.03	6.82E-07
WBP5	No	No	No	Up	1.59	4.19E-05	0.04867	0.73	0.11	2.49E-02	0.68	0.04	1.50E-03	0.64	0.11	4.33E-02	0.70	0.12	4.10E-02	0.92	0.15	2.94E-03	0.58	0.21	5.79E-02	0.37	0.10	1.28E-01	0.76	0.03	7.47E-03
SLC22A15	No	No	No	Up	1.59	1.66E-05	0.00033	1.07	0.11	2.86E-03	0.82	0.04	1.14E-04	0.92	0.11	3.94E-03	0.37	0.11	1.87E-01	0.38	0.14	3.49E-01	0.51	0.21	4.36E-01	1.01	0.10	7.23E-04	0.31	0.03	8.69E-02
INA	No	No	No	Up	1.59	1.83E-19	0.00017	0.32	0.10	1.74E-01	1.10	0.04	4.54E-07	0.56	0.11	5.78E-02	0.78	0.12	1.18E-02	0.16	0.14	4.02E-01	0.80	0.22	5.38E-02	0.83	0.10	3.21E-03	0.85	0.03	3.86E-06

Supplementary Table 2. Details for 2,053 genes differentially expressed between EEC and NEEC, identified by microarray meta-analysis.

EEC - endometrioid endometrial cancer, NEEC - non-endometrioid endometrial cancer, SMD - Standardised Mean Difference, FDR - False Discovery Rate, Var - Variance

Gene	145 gene list	1253 gene list	Associated with EEC-specific survival?	Up/Down Regulated in NEEC	average standardised fold change	Microarray Meta FDR	RNASeq FDR	Individual microarray study results: TCGA			E-MTAB-2532			E-GEOD-56026			GSE32507			GSE24537			E-GEOD-23518			E-GEOD-17025			E-GEOD-2109		
								SMD	Var	P-value	SMD	Var	P-value	SMD	Var	P-value	SMD	Var	P-value	SMD	Var	P-value	SMD	Var	P-value	SMD	Var	P-value	SMD	Var	P-value
CDCA8	No	Yes	No	Up	1.59	1.14E-05	2.8E-05	0.98	0.11	2.61E-03	0.78	0.04	6.20E-05	0.43	0.10	1.03E-01	0.60	0.12	7.52E-02	0.58	0.14	6.85E-02	0.68	0.21	6.22E-02	0.92	0.10	4.31E-03	0.43	0.03	1.41E-02
PURB	No	No	No	Up	1.60	0.000185	validated	0.85	0.11	5.93E-02	0.76	0.04	1.80E-03	1.04	0.11	3.34E-03	0.72	0.12	7.62E-02	0.73	0.14	1.54E-02	0.51	0.21	9.79E-02	0.43	0.10	1.06E-01	0.34	0.03	4.89E-02
CHTF18	No	Yes	No	Up	1.60	0.000362	8.5E-05	0.47	0.10	1.20E-01	0.65	0.04	6.99E-04	0.51	0.11	1.32E-01	0.07	0.11	8.24E-01	0.77	0.15	2.44E-02	1.44	0.25	1.96E-02	0.95	0.10	3.33E-03	0.51	0.03	4.75E-02
SYCP2	No	Yes	No	Up	1.60	8.51E-05	1.5E-19	0.34	0.10	1.88E-01	0.81	0.04	2.08E-04	1.13	0.11	6.97E-04	0.99	0.13	1.48E-03	0.49	0.14	5.83E-01	0.55	0.21	6.08E-01	0.82	0.10	4.20E-02	0.27	0.03	6.32E-02
ZBTB34	No	No	No	Up	1.60	0.014778	0.01133	0.78	0.11	6.99E-02	0.56	0.04	2.35E-02	0.46	0.10	2.01E-01	0.75	0.12	7.29E-02	0.87	0.15	1.06E-01	1.20	0.24	5.55E-02	0.15	0.10	6.16E-01	0.64	0.03	4.47E-02
SLN	No	No	No	Up	1.60	1.83E-19	0.00061	0.51	0.10	4.55E-02	0.76	0.04	1.36E-04	0.47	0.10	2.03E-01	1.48	0.14	2.27E-06	1.02	0.15	4.71E-02	0.08	0.20	7.01E-01	0.44	0.10	2.72E-01	0.65	0.03	9.16E-05
TSPAN7	No	Yes	No	Up	1.60	3.58E-06	1.5E-19	0.75	0.11	3.63E-03	0.03	0.04	8.31E-01	0.43	0.10	5.76E-02	0.32	0.11	2.04E-01	1.38	0.17	7.56E-05	1.60	0.26	8.81E-05	0.42	0.10	6.70E-02	0.49	0.03	5.08E-03
LRRC33	No	No	No	Up	1.60	0.003014	0.00842	0.43	0.10	2.27E-01	0.13	0.04	6.39E-01	0.61	0.11	7.17E-02	0.67	0.12	5.30E-02	1.34	0.16	5.40E-03	0.95	0.22	5.71E-02	0.89	0.10	3.77E-02	0.41	0.03	1.29E-02
SPEG	No	Yes	No	Up	1.60	1.83E-19	1.5E-19	1.46	0.12	2.77E-05	0.71	0.04	2.48E-05	0.71	0.11	3.17E-02	0.96	0.13	7.28E-04	0.45	0.14	2.30E-01	0.17	0.20	6.02E-01	0.50	0.10	1.66E-01	0.46	0.03	9.76E-03
RECK	No	No	No	Up	1.60	9.98E-05	validated	0.61	0.10	5.98E-02	0.48	0.04	5.66E-03	0.35	0.10	1.52E-01	1.42	0.14	7.07E-05	1.02	0.15	2.40E-02	0.71	0.21	1.16E-01	0.75	0.10	1.21E-02	0.10	0.03	4.88E-01
SUSD4	No	Yes	No	Up	1.60	6.71E-06	2.3E-06	1.76	0.13	6.82E-07	0.36	0.04	3.50E-02	0.59	0.11	2.53E-02	0.56	0.12	3.67E-02	0.53	0.14	1.75E-01	0.63	0.21	1.64E-01	0.57	0.10	2.93E-02	0.44	0.03	7.40E-03
KCNG1	No	Yes	No	Up	1.60	1.14E-05	2E-05	1.07	0.11	3.29E-04	0.19	0.04	1.44E-01	0.67	0.11	1.87E-02	1.02	0.13	2.64E-04	0.15	0.14	4.07E-01	1.75	0.28	4.56E-03	0.09	0.10	7.30E-01	0.49	0.03	2.79E-03
RSAD2	No	Yes	No	Up	1.60	1.83E-19	1.5E-19	1.25	0.12	4.20E-05	0.87	0.04	7.27E-06	0.95	0.11	7.33E-04	0.16	0.11	4.99E-01	0.65	0.14	4.21E-02	0.12	0.20	6.30E-01	0.71	0.10	3.88E-03	0.72	0.03	1.36E-04
LDOC1	No	No	No	Up	1.60	3.58E-06	0.00354	1.02	0.11	8.72E-04	0.73	0.04	1.28E-03	0.91	0.11	1.04E-03	0.28	0.11	3.32E-01	1.00	0.15	2.62E-03	1.04	0.23	3.73E-03	0.00	0.10	9.99E-01	0.46	0.03	6.76E-03
OBSL1	No	Yes	No	Up	1.60	1.66E-05	0.00711	0.63	0.10	1.95E-02	0.41	0.04	1.33E-02	0.80	0.11	6.39E-03	0.38	0.12	1.91E-01	0.94	0.15	2.38E-01	0.65	0.21	4.53E-01	0.78	0.10	9.80E-03	0.85	0.03	7.04E-06
LOC440356	No	No	No	Up	1.60	0.000175	0.00024	0.68	0.11	9.31E-03	0.99	0.04	2.21E-04	1.04	0.11	1.12E-02	0.96	0.13	6.40E-03	0.66	0.14	5.44E-01	0.27	0.20	6.36E-01	0.40	0.10	3.23E-01	0.44	0.03	1.11E-02
C7ORF31	No	Yes	No	Up	1.60	0.000125	4.6E-06	0.55	0.10	8.11E-02	0.78	0.04	5.63E-04	1.06	0.11	5.06E-03	0.64	0.12	3.72E-02	0.42	0.14	7.54E-01	0.59	0.21	7.06E-01	0.57	0.10	7.72E-02	0.84	0.03	2.62E-04
PTP4A3	No	Yes	No	Up	1.60	6.71E-06	6.7E-06	1.34	0.12	3.02E-04	0.87	0.04	5.68E-05	0.52	0.11	5.58E-02	0.56	0.12	6.18E-02	0.79	0.15	1.84E-02	0.60	0.21	7.78E-02	0.20	0.10	4.39E-01	0.56	0.03	4.39E-03
SGCE	No	Yes	No	Up	1.60	1.83E-19	0.00076	0.80	0.11	3.90E-03	0.31	0.04	4.14E-02	0.34	0.10	1.41E-01	0.94	0.12	1.40E-03	0.90	0.15	3.06E-03	0.68	0.21	3.32E-02	0.59	0.10	1.65E-02	0.90	0.03	3.41E-05
RTN2	No	Yes	No	Up	1.60	3.58E-06	6E-05	0.84	0.11	1.05E-02	1.09	0.04	9.31E-06	0.13	0.10	6.58E-01	1.14	0.13	1.12E-03	0.32	0.14	3.39E-01	0.34	0.20	3.85E-01	0.89	0.10	2.86E-02	0.68	0.03	8.06E-05
PRNP	No	Yes	Yes	Up	1.60	3.58E-06	0.00015	0.53	0.10	1.12E-01	0.34	0.04	3.50E-02	1.35	0.12	4.36E-05	0.92	0.12	4.09E-03	0.68	0.14	1.03E-02	0.45	0.21	9.90E-02	0.58	0.10	1.73E-02	0.59	0.03	2.78E-03
HDX	No	Yes	No	Up	1.60	2.64E-05	1.5E-19	1.11	0.11	5.09E-04	0.86	0.04	8.05E-04	0.71	0.11	1.87E-02	0.71	0.12	9.08E-03	0.31	0.14	7.47E-01	0.52	0.21	6.93E-01	0.70	0.10	7.50E-02	0.53	0.03	1.95E-03
NEFH	No	Yes	No	Up	1.60	1.83E-19	6.4E-05	0.65	0.11	5.69E-03	0.70	0.04	1.98E-05	0.37	0.10	8.50E-02	0.52	0.12	3.03E-02	0.79	0.15	8.07E-03	0.82	0.22	2.97E-02	0.83	0.10	1.88E-03	0.77	0.03	2.04E-06
ALKBH3	No	Yes	No	Up	1.60	3.97E-05	6.7E-06	1.08	0.11	3.61E-04	0.55	0.04	5.21E-03	0.93	0.11	3.53E-03	0.32	0.11	2.16E-01	0.66	0.14	8.39E-02	1.43	0.25	5.40E-03	0.14	0.10	5.43E-01	0.35	0.03	2.38E-02
ERF	No	Yes	No	Up	1.61	0.002356	1.5E-19	0.73	0.11	4.54E-02	1.00	0.04	4.73E-04	0.40	0.10	2.23E-01	0.22	0.11	5.20E-01	1.01	0.15	2.73E-02	1.17	0.23	3.83E-02	0.82	0.10	3.86E-02	0.12	0.03	4.46E-01
GPR20	No	No	No	Up	1.61	0.000125	1.5E-19	0.84	0.11	1.74E-02	0.64	0.04	4.20E-04	0.52	0.11	1.92E-01	0.53	0.12	4.76E-02	1.18	0.16	3.41E-01	0.72	0.21	2.56E-01	0.28	0.10	4.09E-01	0.77	0.03	3.79E-05
CEBPB	No	Yes	No	Up	1.61	3.97E-05	1.9E-05	1.23	0.12	1.57E-03	0.27	0.04	1.25E-01	1.08	0.11	1.73E-03	0.44	0.12	2.08E-01	0.86	0.15	2.52E-03	0.30	0.20	2.15E-01	1.13	0.10	6.05E-04	0.16	0.03	4.14E-01
PLEKHM2	No	No	No	Up	1.61	0.003975	validated	0.52	0.10	2.02E-01	0.88	0.04	2.28E-03	0.42	0.10	2.70E-01	0.98	0.13	8.38E-02	0.77	0.15	1.15E-02	0.58	0.21	9.97E-02	1.08	0.10	3.56E-02	0.24	0.03	3.74E-01
BIN1	No	Yes	No	Up	1.61	1.14E-05	7.4E-05	0.92	0.11	7.10E-03	0.73	0.04	7.61E-04	0.31	0.10	3.34E-01	0.16	0.11	5.58E-01	1.57	0.17	5.07E-05	0.73	0.21	2.99E-02	0.48	0.10	1.49E-01	0.59	0.03	3.11E-03
C1ORF85	No	Yes	Yes	Up	1.61	0.000121	0.00108	1.29	0.12	9.60E-04	0.52	0.04	1.34E-02	0.64	0.11	3.82E-02	0.86	0.12	4.97E-02	0.99	0.15	1.77E-03	0.57	0.21	7.24E-02	0.13	0.10	6.09E-01	0.49	0.03	4.47E-02
PLEKHG4	No	Yes	No	Up	1.61	1.83E-19	1.5E-19	0.59	0.10	1.80E-02	0.94	0.04	3.86E-06	0.24	0.10	3.60E-01	0.20	0.11	4.39E-01	0.49	0.14	1.75E-01	1.37	0.25	6.57E-03	0.75	0.10	9.35E-03	0.91	0.03	9.63E-05
ZNF250	No	No	No	Up	1.61	0.000334	0.01237	2.09	0.14	1.64E-05	1.03	0.04	1.16E-04	0.49	0.10	1.36E-01	0.53	0.12	2.68E-01	0.71	0.14	1.09E-01	0.00	0.20	1.00E+00	0.44	0.10	2.03E-01	0.19	0.03	3.74E-01
SLC25A32	No	No	No	Up	1.61	0.006384	validated	0.99	0.11	9.34E-03	0.66	0.04	9.21E-03	0.57	0.11	9.61E-02	1.07	0.13	2.56E-02	0.64	0.14	6.25E-01	0.49	0.21	7.57E-01	0.69	0.10	2.28E-02	0.38	0.03	2.13E-01
TSPAN18	No	Yes	No	Up	1.61	6.71E-06	4.3E-05	0.80	0.11	5.11E-02	0.32	0.04	6.41E-02	0.23	0.10	3.72E-01	1.59	0.15	6.36E-06	0.37	0.14	1.43E-01	0.47	0.21	1.48E-01	0.99	0.10	2.70E-02	0.73	0.03	3.54E-05
RNF40	No	No	No	Up	1.61	0.006333	validated	0.83	0.11	7.56E-02	0.49	0.04	5.40E-02	0.26	0.10	5.40E-01	1.22	0.13	3.27E-02	1.27	0.16	1.50E-03	0.97	0.22	2.89E-02	0.35	0.10	3.07E-01	0.12	0.03	4.98E-01
KIF5C	No	No	No	Up	1.61	1.83E-19	0.00182	0.22	0.10	4.67E-01	0.87	0.04	5.91E-06	1.07	0.11	2.91E-04	0.77	0.12	5.37E-03	1.01	0.15	9.00E-03	0.90	0.22	2.33E-02	0.13	0.10	5.34E-01	0.52	0.03	7.70E-04
ZNF343	No	No	Yes	Up	1.61	0.006516	0.011	0.75	0.11	5.33E-02	0.56	0.04	2.73E-02	1.57	0.12	8.81E-04	0.39	0.12	3.07E-01	0.46	0.14	5.77E-01	0.65	0.21	4.26E-01	0.89	0.10	3.07E-02	0.22	0.03	1.72E-01
MCAM	No	No	No	Up	1.62	0.000178	validated	0.56	0.10	8.53E-02	0.50	0.04	7.36E-03	0.68	0.11	1.80E-02	1.42	0.14	1.06E-04	0.66	0.14	5.22E-01	0.54	0.21	6.25E-01	0.72	0.10	1.64E-02	0.45	0.03	3.02E-02
FAM49A	No	No	No	Up	1.62	0.001425	validated	0.78	0.11	6.73E-03	0.41	0.04	4.17E-02	0.26	0.10	2.90E-01	1.01	0.13	6.08E-03	0.72	0.14	4.52E-01	0.88	0.22	3.89E-01	0.98	0.10	2.48E-02	0.49	0.03	6.78E-03
PLEKHG2	No	Yes	No	Up	1.62	7.73E-05	0.00113	0.44	0.10	8.59E-02	0.88	0.04	5.18E-05	0.69	0.11	5.14E-02	0.48	0.12	1.17E-01	1.19	0.16	8.76E-03	1.16	0.23	1.71E-02	0.22	0.10	4.98E-01	0.48	0.03	1.58E-02
RHOBTB3	No	No	No	Up	1.62	1.83E-19	validated	0.44	0.10	9.81E-02	0.85	0.04	2.95E-05	0.54	0.11	2.71E-02	0.52	0.12	4.87E-02	0.95	0.15	2.79E-03	0.87	0.22	1.29E-02	0.41	0.10	6.09E-02	0.96	0.03	1.14E-06
KLK5	No	Yes	No	Up	1.62	0.001258	1.5E-19	0.76	0.11	7.43E-03	0.49	0.04	8.44E-03	1.35	0.12	6.89E-04	0.04	0.11	8.80E-01	0.78	0.15	1.16E-01	0.96	0.22	1.46E-01	0.96	0.10	9.00E-02	0.21	0.03	1.97E-01
ZNF114	No	No	No	Up	1.62	3.58E-06	0.00115	1.40	0.12	2.79E-05	0.74	0.04	2.84E-04	1.08	0.11	4.37E-04	0.63	0.12	1.82E-02	0.51	0.14	2.57E-01	0.29	0.20	6.78E-01	0.55	0.10	5.42E-02	0.34	0.03	3.96E-02
NOVA1	No	Yes	No	Up	1.62	0.002218	2.3E-06	0.97	0.11	5.16E-03	0.72	0.04	3.25E-03	0.70	0.11	9.46E-03	0.73	0.12	2.09E-02	1.11	0.16	3.91E-01	1.04	0.23	3.63E-01	0.03	0.10	8.93E-01	0.25	0.03	9.22E-02
CPA4	No	No	No	Up	1.62	0.002072	validated	0.41	0.10	2.22E-01	0.43	0.04	8.73E-02	0.17	0.10	7.22E-01	1.32	0.14	5.69E-04	1.32	0.16	4.94E-03	0.72	0.21	8.99E-02	0.96	0.10	4.00E-02	0.23	0.03	2.30E-01
SLC2A4RG	No	Yes	No	Up	1.62	5.61E-05	0.02352	1.03	0.11	5.55E-03	0.86	0.04	1.02E-04	0.90	0.11	1.11E-02	0.05	0.11	8.88E-01	0.96	0.15	2.04E-02	0.44	0.20	3.32E-01	1.28	0.11	6.62E-04	0.03	0.03	8.14E-01
LMO1	No	Yes	No	Up	1.62	4.80E-05	1.5E-19	1.09	0.11	5.00E-05	0.85	0.04	2.41E-05	0.28	0.10	4.73E-01	0.57	0.12	3.19E-02	0.69	0.14	1.26E-01	1.06	0.23	3.62E-01	0.80	0.10	1.21E-01	0.23	0.03	1.51E-01
GHR	No	No	No	Up	1.62	1.14E-05	validated	0.50	0.10	1.22E-01	0.29	0.04	8.58E-02	0.22	0.10	3.28E-01	1.33	0.14	2.48E-05	0.83	0.15	1.59E-02	0.92	0.22	7.16E-02	0.93	0.10	4.46E-03	0.57	0.03	9.59E-04
LAYN	No	No	No	Up	1.62	6.71E-06	0.00052	1.09	0.11	1.37E-03	0.55	0.04	2.41E-03	0.90	0.11	3.33E-03	1.60	0.15	3.43E-05	0.64	0.14	3.91E-02	0.29	0.20	2.77E-01	0.19	0.10	4.66E-01	0.31	0.03	5.26E-02
C1QL1	No	No	No	Up	1.62	0.001594	0.00043	0.76	0.11	9.13E-03	0.65	0.04	2.04E-03	0.46	0.10	2.20E-01	0.46	0.12	7.88E-02	1.45	0.17	5.06E-03	0.97	0.22	1.60E-01	0.83	0.10	7.30E-02	0.00	0.03	9.79E-01
LPHN1	No	Yes	No	Up	1.62	3.58E-06	0.00025	1.18	0.11	4.56E-04	0.90	0.04	1.98E-05	0.45	0.10	1.10E-01	0.47	0.12	1.33E-01	0.97	0.15	4.36E-03	0.50	0.21	1.10E-01	0.50	0.10	8.77E-02	0.62	0.03	5.33E-03
CYBRD1	No	Yes	No	Up	1.62	1.83E-19	0.00211	1.33	0.12	1.93E-04	0.26	0.04	7.70E-02	0.69	0.11	1.25E-02	1.58	0.15	2.23E-05	0.83	0.15	3.52E-03	0.35	0.20	1.70E-01	0.04	0.10	8.48E-01	0.50	0.03	1.81E-03
RB1CC1	No	Yes	No	Up	1.62	0.000359	0.00713	0.72	0.11	5.93E-02	0.87	0.04	1.28E-03	0.92	0.11	1.47E-02	0.79	0.12	6.15E-02	0.42	0.14	1.13E-01	0.59	0.21	9.19E-02	1.17	0.10	1.47E-03	0.12	0.03	6.40E-01
PALM	No	Yes	No	Up	1.63	1.83E-19	1.5E-19	0.84	0.11	1.49E-03	0.49	0.04	2.72E-03	0.14	0.10	5.73E-01	0.71	0.12	1.52E-02	1.07	0.15	1.27E-03	0.67	0.21	4.28E-02	0.87	0.10	7.12E-02	0.81	0.03	4.79E-05
NUF2	No	Yes	No	Up	1.63	9.89E-06	0.00031	0.72	0.11	1.34E-02	0.83	0.04	2.52E-05	0.32	0.10	1.73E-01	0.76	0.12	1.78E-02	0.44	0.14	4.57E-01	1.08	0.23	2.97E-01	0.85	0.10	1.70E-03	0.63	0.03	1.01E-03
SPHK1	No	No	No	Up	1.63	1.14E-05	validated	0.85	0.11	6.00E-03	0.78	0.04	5.47E-05	0.46	0.10	2.15E-01	1.20	0.13	5.75E-04	1.10	0.15	1.42E-02	0.25	0.20	4.20E-01	0.63	0.10	1.37E-01	0.36	0.03	4.84E-02
NTNG1	No	Yes	No	Up	1.63	0.000185	1.5E-19	0.57	0.10	4.81E-02	0.84	0.04	9.91E-04	1.31	0.12	2.97E-04	0.42	0.12	1.01E-01	0.89	0.15	2.23E-01	0.99	0.22	1.96E-01	0.14	0.10	6.53E-01	0.47	0.03	5.14E-03
MYT1	No	Yes	No	Up	1.63	0.000432	1.5E-19	1.06	0.11	6.30E-04	0.93	0.04	1.10E-04	0.54	0.11	2.29E-01	0.38	0.12	1.16E-01	1.49	0.17	7.38E-02	0.79	0.22	7.46E-02	0.24	0.10	5.71E-01	0.20	0.03	1.67E-01
ZSWIM3	No	No	No	Up	1.63	0.006507	0.00109	0.91	0.11	1.64E-02	0.41	0.04	8.69E-02	0.78	0.11	5.90E-02	0.88	0.12	6.56E-02	0.65	0.14	2.68E-01	0.47	0.21	4.01E-01	0.98	0.10	3.93E-02	0.54	0.03	2.17E-02
CRABP2	No	Yes	No	Up	1.63	1.83E-19	1.5E-19	0.53	0.10	2.48E-02	0.87	0.04	3.18E-06	0.79	0.11	6.41E-03	1.07	0.13	5.10E-04	0.48	0.14	4.91E-02	1.17	0.23	1.32E-03	0.05	0.10	7.92E-01	0.67	0.03	1.21E-04
OSBPL11	No	Yes	No	Up	1.63	0.000176	2.3E-06	0.82	0.11	2.64E-02	0.90	0.04	2.26E-04	0.91	0.11	7.47E-03	0.10	0.11	7.92E-01	0.42	0.14	3.86E-01	0.86	0.22	3.77E-01	1.32	0.11	4.97E-04	0.30	0.03	7.19E-02
TDRD5	No	Yes	No	Up	1.63	0.002433	1.5E-19	0.04	0.10	8.91E-01	0.66	0.04	1.08E-02	0.73	0.11	2.21E-02	0.65	0.12	9.88E-02	1.10	0.15	2.72E-01	1.08	0.23	2.12E-01	0.84	0.10	3.78E-02	0.54	0.03	1.07E-03
FKBP10	No	No	No	Up	1.63	6.71E-06	validated	0.59	0.10	3.10E-02	0.92	0.04	6.59E-06	0.45	0.10	9.43E-02	0.91	0.12	3.38E-03	1.12	0.16	1.14E-02	0.92	0.22	5.28E-02	0.33	0.10	4.33E-01	0.40	0.03	7.18E-03
PRAF2	No	Yes	No	Up	1.63	0.00033	0.00585	0.56	0.10	7.79E-02	1.04	0.04	2.43E-05	0.65	0.11	8.01E-02	0.08	0.11	8.31E-01	1.00	0.15	9.29E-02	0.85	0.22	1.32E-01	0.97	0.10	1.47E-02	0.51	0.03	2.45E-02
ZNF300	No	Yes	No	Up	1.63	1.83E-19	1.5E-19	1.21	0.11	5.91E-05	0.53	0.04	1.81E-03	0.98	0.11	6.13E-04	0.76	0.12	5.51E-03	0.38	0.14	5.63E-01	0.20	0.20	7.23E-01	1.01	0.10	4.64E-04	0.58	0.03	8.24E-04
CDIPT	No	No	No	Up	1.63	0.003959	0.00416	0.79	0.11	2.11E-02	0.17	0.04	3.15E-01	0.81	0.11	7.69E-02	1.38	0.14	5.89E-03	0.79	0.15	1.07E-01	0.07	0.20	8.05E-01	1.38	0.11	1.72E-03	0.26	0.03	3.52E-01
APCDD1L	No	No	No	Up	1.63	3.58E-06	0.01225	0.89	0.11	2.30E-03	0.65	0.04	9.54E-04	0.34	0.10	2.82E-01	0.88	0.12	1.73E-03	1.05	0.15	8.14E-03	1.00	0.22	1.23E-02	0.31	0.10	4.12E-01	0.55	0.03	1.52E-03
PRRG3	No	Yes	No	Up	1.63	0.003676	1.3E-05	1.07	0.11	1.12E-02	0.59	0.04	1.52E-02	1.02	0.11	2.41E-03	0.23	0.11	4.20E-01	0.80	0.15	4.17E-01	1.20	0.24	2.90E-01	0.36	0.10	2.70E-01	0.39	0.03	2.31E-02
GID8	No	No	No	Up	1.63	0.000379	0.00026	1.29	0.12	4.22E-03	0.59	0.04	9.52E-03	1.23	0.11	1.50E-03	0.86	0.12	6.40E-02	0.54	0.14	4.30E-02	0.87	0.22	1.30E-02	0.18	0.10	5.10E-01	0.11	0.03	6.69E-01

Supplementary Table 2. Details for 2,053 genes differentially expressed between EEC and NEEC, identified by microarray meta-analysis.

EEC - endometrioid endometrial cancer, NEEC - non-endometrioid endometrial cancer, SMD - Standardised Mean Difference, FDR - False Discovery Rate, Var - Variance

Gene	145 gene list	1253 gene list	Associated with EEC-specific survival?	Up/Down Regulated in NEEC	average standardised fold change	Microarray Meta FDR	RNASeq FDR	Individual microarray study results: TCGA			E-MTAB-2532			E-GEOD-56026			GSE32507			GSE24537			E-GEOD-23518			E-GEOD-17025			E-GEOD-2109		
								SMD	Var	P-value	SMD	Var	P-value	SMD	Var	P-value	SMD	Var	P-value	SMD	Var	P-value	SMD	Var	P-value	SMD	Var	P-value	SMD	Var	P-value
PCOLCE	No	No	No	Up	1.63	1.83E-19	validated	0.39	0.10	1.16E-01	0.59	0.04	7.55E-04	0.36	0.10	1.33E-01	1.92	0.16	1.36E-06	0.81	0.15	4.10E-03	0.76	0.21	1.92E-02	0.12	0.10	6.12E-01	0.69	0.03	2.19E-04
ST8SIA5	No	Yes	No	Up	1.64	0.000125	1.5E-19	0.94	0.11	8.64E-03	1.25	0.04	6.82E-07	0.57	0.11	2.55E-01	0.32	0.11	2.66E-01	0.66	0.14	9.99E-02	0.94	0.22	1.84E-01	0.73	0.10	1.12E-01	0.27	0.03	1.07E-01
ZNF512B	No	Yes	No	Up	1.64	2.85E-05	0.00253	0.97	0.11	1.40E-02	0.97	0.04	8.59E-05	0.79	0.11	7.64E-03	0.07	0.11	8.26E-01	0.69	0.14	1.37E-01	0.61	0.21	2.33E-01	1.40	0.11	6.47E-05	0.19	0.03	3.80E-01
POLR2D	No	No	No	Up	1.64	0.000188	validated	0.74	0.11	6.46E-02	0.85	0.04	5.60E-04	0.88	0.11	1.17E-02	1.11	0.13	1.94E-02	0.35	0.14	2.11E-01	0.56	0.21	1.19E-01	0.33	0.10	2.16E-01	0.87	0.03	5.04E-03
TMEM100	No	No	No	Up	1.64	1.83E-19	0.00114	0.54	0.10	3.33E-02	0.72	0.04	5.95E-05	0.59	0.11	1.27E-02	0.79	0.12	3.80E-03	0.71	0.14	3.65E-02	1.32	0.24	3.40E-03	0.25	0.10	2.39E-01	0.77	0.03	2.48E-05
PSMD2	No	Yes	No	Up	1.64	0.000712	0.00424	1.43	0.12	9.50E-04	0.23	0.04	1.68E-01	0.75	0.11	4.38E-02	1.57	0.15	2.37E-03	0.37	0.14	1.29E-01	0.54	0.21	7.43E-02	0.04	0.10	8.90E-01	0.77	0.03	2.02E-02
MCOLN3	No	Yes	No	Up	1.64	5.03E-05	1.5E-19	1.12	0.11	1.48E-03	0.27	0.04	1.88E-01	0.96	0.11	5.04E-04	0.34	0.11	2.20E-01	0.67	0.14	6.46E-02	1.33	0.24	4.24E-02	0.60	0.10	1.60E-02	0.40	0.03	1.19E-02
MYH6	No	Yes	No	Up	1.64	1.83E-19	1.5E-19	0.78	0.11	4.39E-02	1.17	0.04	1.36E-06	0.06	0.10	8.96E-01	0.89	0.12	1.73E-03	0.91	0.15	2.88E-01	0.80	0.22	1.15E-01	0.26	0.10	4.76E-01	0.83	0.03	7.72E-06
CEBPG	No	Yes	No	Up	1.64	0.000108	7.8E-05	0.76	0.11	1.98E-02	0.58	0.04	4.46E-03	0.68	0.11	1.78E-02	0.83	0.12	5.40E-02	1.13	0.16	6.11E-03	0.68	0.21	5.00E-02	0.54	0.10	6.01E-02	0.50	0.03	4.81E-02
CTSF	No	Yes	No	Up	1.64	1.14E-05	0.00133	0.61	0.10	2.39E-02	0.42	0.04	1.09E-02	1.02	0.11	3.58E-03	1.01	0.13	3.12E-03	1.05	0.15	2.39E-03	0.86	0.22	2.04E-02	0.32	0.10	2.06E-01	0.41	0.03	5.59E-02
TIGD5	No	No	Yes	Up	1.64	0.000209	0.00726	0.97	0.11	9.06E-03	1.13	0.04	1.86E-05	0.52	0.11	1.82E-01	0.41	0.12	2.87E-01	0.59	0.14	6.37E-02	0.64	0.21	1.09E-01	1.43	0.11	4.34E-03	0.01	0.03	9.38E-01
FADS3	No	Yes	No	Up	1.64	0.001301	7.7E-05	0.96	0.11	6.61E-03	0.69	0.04	1.98E-03	0.96	0.11	1.40E-02	0.67	0.12	1.21E-01	0.74	0.14	5.38E-01	0.51	0.21	5.80E-01	0.79	0.10	4.81E-02	0.39	0.03	3.69E-02
LIN37	No	No	No	Up	1.64	0.018189	validated	1.23	0.12	1.89E-03	0.58	0.04	1.06E-02	0.46	0.10	1.68E-01	0.18	0.11	6.79E-01	1.41	0.17	8.33E-02	1.60	0.26	9.61E-02	0.12	0.10	6.95E-01	0.11	0.03	5.28E-01
LOXL1	No	Yes	No	Up	1.64	2.64E-05	0.0005	0.64	0.11	1.33E-02	0.59	0.04	1.75E-03	0.50	0.10	5.79E-02	0.87	0.12	9.50E-03	1.05	0.15	2.33E-02	1.28	0.24	1.71E-02	0.09	0.10	7.14E-01	0.71	0.03	1.35E-03
FOXK1	No	Yes	No	Up	1.64	0.000188	9.3E-05	0.75	0.11	8.12E-02	0.31	0.04	5.78E-02	0.73	0.11	1.98E-02	0.55	0.12	1.52E-01	1.51	0.17	1.69E-04	1.27	0.24	2.24E-03	0.57	0.10	5.29E-02	0.04	0.03	7.93E-01
SALL4	No	Yes	No	Up	1.64	1.83E-19	1.5E-19	0.57	0.10	2.55E-02	1.22	0.04	1.00E-20	0.56	0.11	7.01E-02	0.42	0.12	8.75E-02	0.80	0.15	1.86E-01	1.13	0.23	8.01E-02	0.10	0.10	6.84E-01	0.92	0.03	2.27E-07
ZNF34	No	No	No	Up	1.64	0.004289	validated	0.90	0.11	1.34E-02	0.56	0.04	2.83E-02	0.84	0.11	6.64E-02	0.91	0.12	7.11E-02	0.72	0.14	1.11E-01	0.56	0.21	2.20E-01	0.90	0.10	2.28E-02	0.34	0.03	1.91E-01
PDCD5	No	Yes	Yes	Up	1.64	1.14E-05	5.6E-05	1.07	0.11	4.47E-03	0.91	0.04	8.34E-05	0.90	0.11	5.29E-03	0.44	0.12	2.55E-01	0.88	0.15	5.24E-03	0.50	0.21	9.15E-02	0.61	0.10	1.22E-01	0.42	0.03	6.44E-02
GPX3	No	Yes	No	Up	1.64	1.83E-19	0.0001	1.39	0.12	3.00E-05	0.56	0.04	6.05E-04	0.66	0.11	7.28E-03	0.81	0.12	8.07E-03	1.25	0.16	1.41E-04	0.28	0.20	2.64E-01	0.42	0.10	5.40E-02	0.37	0.03	2.02E-02
HPSE	No	Yes	No	Up	1.64	5.26E-05	1.5E-19	0.84	0.11	4.41E-03	0.38	0.04	4.27E-02	0.53	0.11	4.76E-02	1.33	0.14	1.87E-04	1.16	0.16	5.06E-02	0.34	0.20	6.93E-01	0.72	0.10	1.58E-02	0.45	0.03	1.78E-02
ALX1	No	Yes	No	Up	1.64	0.000707	1.5E-19	0.26	0.10	3.34E-01	0.72	0.04	1.65E-02	0.57	0.11	1.49E-01	0.34	0.11	1.90E-01	1.11	0.16	2.51E-01	0.97	0.22	2.92E-01	0.99	0.10	2.15E-02	0.78	0.03	1.14E-05
VEGFB	No	No	No	Up	1.64	0.000502	validated	1.69	0.13	2.69E-04	0.26	0.04	2.35E-01	0.72	0.11	5.74E-02	0.61	0.12	1.65E-01	1.10	0.15	5.91E-04	0.51	0.21	8.20E-02	0.74	0.10	6.97E-02	0.11	0.03	6.17E-01
SNTA1	No	Yes	No	Up	1.64	9.98E-05	0.00587	0.61	0.10	3.17E-02	0.86	0.04	6.16E-05	0.95	0.11	1.42E-02	0.64	0.12	4.08E-02	0.98	0.15	6.09E-02	0.40	0.20	3.46E-01	0.71	0.10	1.25E-01	0.59	0.03	1.94E-02
DPF1	No	No	No	Up	1.65	0.000888	0.00634	0.56	0.10	1.08E-01	0.65	0.04	2.08E-02	0.20	0.10	6.70E-01	1.50	0.14	3.24E-04	0.38	0.14	4.75E-01	1.02	0.23	2.07E-01	0.76	0.10	1.07E-01	0.69	0.03	9.96E-04
ADIPOR2	No	No	No	Up	1.65	0.001612	validated	1.20	0.11	3.09E-02	0.12	0.04	5.81E-01	0.32	0.10	3.85E-01	1.22	0.13	9.10E-04	0.77	0.15	2.48E-02	0.79	0.22	4.87E-02	0.46	0.10	1.44E-01	0.88	0.03	1.77E-02
PGS1	No	Yes	No	Up	1.65	0.003128	0.00079	1.00	0.11	6.96E-03	0.25	0.04	2.13E-01	1.25	0.12	5.19E-03	0.51	0.12	2.16E-01	1.21	0.16	2.57E-02	0.61	0.21	1.68E-01	0.66	0.10	9.94E-02	0.27	0.03	1.28E-01
SOX12	No	Yes	Yes	Up	1.65	3.34E-05	0.00011	1.12	0.11	2.67E-03	1.18	0.04	1.23E-05	0.81	0.11	1.10E-02	0.37	0.11	2.65E-01	0.16	0.14	7.62E-01	0.92	0.22	3.60E-01	0.53	0.10	1.51E-01	0.68	0.03	1.81E-03
ANKRD27	No	Yes	No	Up	1.65	0.000586	1.5E-19	1.21	0.11	1.14E-03	0.87	0.04	6.84E-04	0.66	0.11	4.80E-02	0.97	0.13	3.61E-02	0.33	0.14	6.43E-01	0.60	0.21	5.03E-01	0.42	0.10	1.96E-01	0.69	0.03	2.05E-02
PTPRT	No	Yes	No	Up	1.65	1.14E-05	1.5E-19	1.25	0.12	3.09E-04	0.43	0.04	6.02E-03	0.49	0.10	1.58E-01	0.80	0.12	1.92E-03	0.44	0.14	4.78E-01	0.72	0.21	4.97E-01	0.94	0.10	4.59E-02	0.71	0.03	5.29E-05
GTF2H4	No	No	No	Up	1.65	0.001594	0.00951	1.00	0.11	2.11E-02	0.37	0.04	6.85E-02	0.21	0.10	5.37E-01	0.72	0.12	6.39E-02	0.71	0.14	3.01E-02	2.08	0.31	2.41E-04	0.35	0.10	2.40E-01	0.31	0.03	2.18E-01
TRIM46	No	Yes	Yes	Up	1.65	0.002182	1.5E-19	0.56	0.10	1.51E-01	0.76	0.04	1.63E-03	0.57	0.11	1.44E-01	0.49	0.12	1.07E-01	0.89	0.15	1.26E-01	1.24	0.24	9.52E-02	0.94	0.10	1.55E-02	0.31	0.03	5.23E-02
SYNGR3	No	Yes	No	Up	1.65	1.66E-05	1.5E-19	0.78	0.11	2.09E-02	0.70	0.04	1.59E-03	0.77	0.11	8.09E-03	0.40	0.12	2.82E-01	0.94	0.15	3.23E-01	0.52	0.21	6.22E-01	1.09	0.10	3.22E-04	0.57	0.03	4.73E-04
JRK	No	No	No	Up	1.65	0.024274	0.01055	0.77	0.11	1.07E-01	0.47	0.04	5.48E-02	1.28	0.12	9.35E-03	0.42	0.12	2.45E-01	0.97	0.15	3.85E-01	0.95	0.22	3.58E-01	0.57	0.10	1.80E-01	0.35	0.03	4.21E-02
CDC7	No	Yes	No	Up	1.65	3.58E-06	0.00018	0.41	0.10	1.48E-01	1.03	0.04	5.68E-06	0.56	0.11	3.83E-02	0.69	0.12	4.86E-02	0.64	0.14	1.64E-01	0.50	0.21	3.08E-01	1.15	0.10	2.14E-04	0.80	0.03	7.75E-04
SLC38A1	No	Yes	No	Up	1.65	1.83E-19	1.5E-19	1.39	0.12	3.61E-05	0.84	0.04	2.29E-05	1.01	0.11	9.16E-04	0.67	0.12	3.76E-02	0.67	0.14	2.26E-02	0.59	0.21	5.95E-02	0.47	0.10	5.27E-02	0.12	0.03	3.89E-01
CDCA5	No	Yes	No	Up	1.65	1.83E-19	6.7E-06	1.01	0.11	1.89E-03	0.83	0.04	3.16E-05	0.73	0.11	1.57E-02	0.70	0.12	3.39E-02	0.38	0.14	1.22E-01	0.94	0.22	1.04E-02	0.59	0.10	5.23E-02	0.60	0.03	1.32E-03
WDR70	No	No	No	Up	1.65	0.001737	0.00472	1.00	0.11	3.39E-02	0.87	0.04	1.05E-03	0.60	0.11	1.58E-01	0.36	0.11	5.43E-01	0.88	0.15	2.04E-02	1.14	0.23	1.31E-02	0.49	0.10	1.49E-01	0.43	0.03	1.81E-01
ADNP	No	Yes	No	Up	1.65	0.000781	0.00212	0.87	0.11	1.34E-02	0.41	0.04	2.55E-02	0.76	0.11	4.32E-02	0.87	0.12	4.44E-02	0.40	0.14	1.98E-01	1.06	0.23	1.90E-02	0.46	0.10	1.27E-01	0.95	0.03	1.18E-02
IGFBP6	No	Yes	No	Up	1.65	3.58E-06	1.5E-19	0.81	0.11	1.10E-02	0.45	0.04	5.70E-03	0.83	0.11	3.75E-03	0.78	0.12	1.02E-02	0.98	0.15	1.77E-03	0.98	0.22	5.98E-03	0.51	0.10	8.00E-02	0.45	0.03	2.40E-02
OCA2	No	Yes	No	Up	1.65	1.14E-05	1.5E-19	1.36	0.12	8.18E-06	0.30	0.04	4.86E-02	0.88	0.11	4.35E-03	0.36	0.11	1.49E-01	0.83	0.15	1.54E-01	0.73	0.21	1.76E-01	0.74	0.10	3.80E-02	0.59	0.03	1.58E-03
KCNH2	No	Yes	No	Up	1.65	1.83E-19	1.5E-19	0.77	0.11	1.50E-02	0.91	0.04	7.72E-06	0.53	0.11	4.27E-02	0.91	0.12	6.59E-04	0.48	0.14	1.76E-01	0.81	0.22	1.28E-01	0.78	0.10	3.65E-02	0.61	0.03	1.77E-04
C15ORF27	No	No	Yes	Up	1.65	0.00028	0.00012	1.27	0.12	7.29E-05	0.95	0.04	2.54E-05	0.35	0.10	4.59E-01	0.21	0.11	3.64E-01	1.02	0.15	4.09E-01	0.85	0.22	3.51E-01	0.75	0.10	1.38E-01	0.39	0.03	6.84E-02
C6ORF114	No	Yes	No	Up	1.65	2.64E-05	1.5E-19	0.56	0.10	5.81E-02	1.16	0.04	5.50E-05	0.67	0.11	2.30E-02	1.40	0.14	3.50E-04	0.11	0.14	9.03E-01	0.65	0.21	5.89E-01	0.79	0.10	2.45E-02	0.46	0.03	1.63E-02
WASF1	No	Yes	No	Up	1.65	5.61E-05	1.5E-19	0.77	0.11	5.84E-03	0.81	0.04	5.36E-05	0.24	0.10	2.82E-01	0.61	0.12	4.01E-02	0.91	0.15	2.48E-01	1.34	0.25	1.40E-01	0.55	0.10	3.23E-02	0.56	0.03	5.12E-03
STON1	No	Yes	No	Up	1.65	1.83E-19	6.7E-06	1.29	0.12	1.89E-05	0.49	0.04	3.58E-03	1.03	0.11	3.87E-04	1.00	0.13	1.45E-03	0.21	0.14	4.08E-01	0.75	0.21	2.22E-01	0.58	0.10	2.89E-02	0.47	0.03	8.67E-03
LEPREL2	No	No	No	Up	1.65	1.83E-19	validated	0.41	0.10	1.00E-01	0.73	0.04	1.01E-04	0.43	0.10	1.51E-01	1.03	0.13	1.43E-03	1.19	0.16	2.51E-03	0.98	0.22	1.53E-02	0.30	0.10	3.85E-01	0.74	0.03	2.95E-05
MAGEA4	No	Yes	No	Up	1.65	1.83E-19	1.5E-19	1.14	0.11	2.47E-04	0.60	0.04	8.15E-04	0.93	0.11	1.07E-03	0.88	0.12	1.55E-03	0.36	0.14	2.81E-01	0.49	0.21	1.38E-01	0.74	0.10	1.37E-02	0.68	0.03	2.34E-05
ARHGEF4	No	Yes	No	Up	1.65	2.85E-05	0.00014	0.38	0.10	1.91E-01	0.55	0.04	2.00E-03	0.98	0.11	6.17E-03	0.16	0.11	5.26E-01	0.40	0.14	2.81E-01	1.34	0.25	4.32E-02	1.55	0.11	3.02E-05	0.46	0.03	1.33E-02
CIC	No	Yes	No	Up	1.66	0.000594	1.5E-19	0.39	0.10	2.73E-01	0.85	0.04	8.54E-04	0.57	0.11	1.74E-01	0.07	0.11	8.56E-01	1.25	0.16	4.14E-03	0.97	0.22	3.10E-02	1.30	0.11	1.03E-02	0.44	0.03	3.93E-02
SFMBT1	No	Yes	No	Up	1.66	1.83E-19	5.5E-05	1.43	0.12	8.00E-05	1.26	0.04	4.54E-07	0.36	0.10	1.93E-01	0.44	0.12	2.31E-01	0.27	0.14	3.60E-01	0.43	0.20	2.70E-01	0.89	0.10	1.36E-02	0.75	0.03	3.40E-03
ZNF536	No	Yes	No	Up	1.66	1.83E-19	1.5E-19	0.34	0.10	2.44E-01	0.51	0.04	7.24E-02	0.93	0.11	2.95E-03	1.36	0.14	4.32E-05	0.13	0.14	8.15E-01	0.63	0.21	3.33E-01	1.01	0.10	1.06E-02	0.93	0.03	9.09E-07
NACAD	No	Yes	No	Up	1.66	1.83E-19	0.0005	0.64	0.11	2.36E-02	0.73	0.04	3.34E-04	0.66	0.11	9.55E-02	1.10	0.13	6.20E-04	0.80	0.15	1.46E-02	0.69	0.21	5.41E-02	0.47	0.10	3.05E-01	0.74	0.03	2.91E-05
ARFRP1	No	No	No	Up	1.66	0.009194	validated	1.08	0.11	4.96E-03	0.70	0.04	4.86E-03	0.49	0.10	2.01E-01	1.10	0.13	2.58E-02	1.01	0.15	1.91E-01	0.97	0.22	2.14E-01	0.37	0.10	4.12E-01	0.12	0.03	5.59E-01
CAND2	No	No	No	Up	1.66	1.83E-19	0.02257	0.94	0.11	6.12E-04	0.75	0.04	7.11E-05	0.65	0.11	2.13E-02	1.21	0.13	2.11E-04	0.75	0.14	4.32E-02	0.37	0.20	2.72E-01	0.30	0.10	2.03E-01	0.87	0.03	4.61E-05
CXCR7	No	Yes	No	Up	1.66	1.83E-19	0.00011	0.20	0.10	4.08E-01	0.78	0.04	9.43E-05	0.78	0.11	4.44E-03	1.06	0.13	1.87E-03	0.75	0.14	1.05E-02	1.44	0.25	3.77E-04	0.77	0.10	4.21E-03	0.05	0.03	7.30E-01
PHF16	No	Yes	No	Up	1.66	0.000186	0.00082	0.68	0.11	9.71E-02	0.52	0.04	1.74E-02	1.09	0.11	4.56E-03	0.55	0.12	1.03E-01	0.59	0.14	9.01E-02	0.75	0.21	6.83E-02	1.17	0.10	7.20E-04	0.50	0.03	6.11E-02
GPC1	No	Yes	No	Up	1.66	2.85E-05	0.01365	0.34	0.10	2.49E-01	0.81	0.04	3.57E-04	0.71	0.11	2.67E-02	0.98	0.13	8.25E-03	0.95	0.15	1.89E-02	0.99	0.22	2.70E-02	0.65	0.10	4.54E-02	0.43	0.03	1.27E-02
TRERF1	No	No	No	Up	1.66	0.000227	0.01759	0.90	0.11	3.87E-03	0.74	0.04	3.10E-04	0.23	0.10	3.29E-01	0.93	0.12	8.20E-03	1.04	0.15	1.01E-01	1.39	0.25	6.51E-02	0.28	0.10	2.85E-01	0.34	0.03	5.00E-02
CCDC77	No	Yes	No	Up	1.66	0.000108	0.00178	1.10	0.11	6.52E-03	0.62	0.04	4.46E-03	0.73	0.11	2.27E-02	0.34	0.11	4.27E-01	0.54	0.14	1.45E-01	0.86	0.22	3.91E-02	0.66	0.10	2.81E-02	1.01	0.04	1.66E-03
SLC7A9	No	Yes	No	Up	1.66	0.000283	1.5E-19	1.57	0.12	9.61E-05	0.36	0.04	1.11E-01	0.45	0.10	2.38E-01	0.37	0.11	2.10E-01	0.66	0.14	4.00E-01	0.54	0.21	3.32E-01	1.48	0.11	1.36E-04	0.44	0.03	4.47E-02
SMO	No	No	No	Up	1.66	0.000623	0.04993	0.84	0.11	5.84E-03	0.55	0.04	3.82E-03	1.11	0.11	2.31E-02	0.33	0.11	2.47E-01	1.12	0.16	1.33E-02	1.21	0.24	2.14E-02	0.36	0.10	4.03E-01	0.34	0.03	1.47E-01
GABRG3	No	Yes	No	Up	1.66	0.005988	1.5E-19	0.89	0.11	2.16E-03	0.30	0.04	2.30E-01	0.90	0.11	4.31E-02	0.56	0.12	4.20E-02	1.05	0.15	4.84E-01	1.00	0.23	4.29E-01	0.79	0.10	1.52E-01	0.38	0.03	1.46E-02
KIF1A	No	Yes	No	Up	1.66	1.83E-19	1.5E-19	0.71	0.11	6.74E-03	0.90	0.04	2.27E-06	1.25	0.12	3.98E-05	0.10	0.11	7.08E-01	0.70	0.14	1.50E-02	0.83	0.22	1.26E-02	0.29	0.10	2.24E-01	1.09	0.04	1.00E-20
CASKIN2	No	Yes	No	Up	1.66	0.02674	0.00025	2.00	0.14	1.81E-04	0.30	0.04	1.52E-01	0.67	0.11	1.05E-01	0.27	0.11	2.77E-01	0.88	0.15	5.47E-01	0.74	0.21	5.99E-01	0.95	0.10	1.05E-01	0.08	0.03	6.60E-01
CSNK2B	No	No	No	Up	1.66	0.000524	validated	1.38	0.12	9.35E-03	0.40	0.04	9.01E-02	0.66	0.11	9.82E-02	0.81	0.12	1.32E-01	0.63	0.14	1.74E-02	1.25	0.24	9.78E-04	0.11	0.10	6.44E-01	0.64	0.03	1.15E-02
SKI	No	Yes	No	Up	1.67	0.000781	0.00331	0.19	0.10	5.65E-01	0.76	0.04	1.50E-05	0.63	0.11	4.73E-02	0.16	0.11	5.04E-01	1.51	0.17	8.23E-02	1.41	0.25	1.27E-01	0.66	0.10	4.77E-02	0.56	0.03	3.64E-02
CCDC99	No	Yes	No	Up	1.67	1.14E-05	2.6E-05	0.64	0.11	4.87E-02	0.88	0.04	1.44E-04	0.37	0.10	1.65E-01	0.95	0.12	1.90E-02	0.88	0.15	2.47E-02	0.66	0.21	9.95E-02	0.87	0.10	3.38E-03	0.64	0.03	8.45E-03
CENPA	No	Yes	No	Up	1.67	1.83E-19	1.5E-19	1.20	0.11	3.24E-04	0.82	0.04	3.95E-05	0.52	0.11	3.75E-02	0.56	0.12	5.46E-02	0.57	0.14	7.43E-02	0.87	0.22	2.40E-02	0.59	0.10	1.78E-02	0.77	0.03	1.56E-04
ACTN2	No	No	No	Up	1.67	1.83E-19	0.00053	0.52	0.10	3.67E-02	0.80	0.04	3.34E-04	0.61	0.11	1.62E-01	1.00	0.13	2.71E-04	0.74	0.14	5.12E-01	0.64	0.21	4.85E-01	0.63	0.10	1.49E-01	0.96	0.03	1.00E-20

Supplementary Table 2. Details for 2,053 genes differentially expressed between EEC and NEEC, identified by microarray meta-analysis.

EEC - endometrioid endometrial cancer, NEEC - non-endometrioid endometrial cancer, SMD - Standardised Mean Difference, FDR - False Discovery Rate, Var - Variance

Gene	145 gene list	1253 gene list	Associated with EEC-specific survival?	Up/Down Regulated in NEEC	average standardised fold change	Microarray Meta FDR	RNASeq FDR	Individual microarray study results																							
								TCGA			E-MTAB-2532			E-GEOD-56026			GSE32507			GSE24537			E-GEOD-23518			E-GEOD-17025			E-GEOD-2109		
								SMD	Var	P-value	SMD	Var	P-value	SMD	Var	P-value	SMD	Var	P-value	SMD	Var	P-value	SMD	Var	P-value	SMD	Var	P-value	SMD	Var	P-value
FAM83D	No	Yes	No	Up	1.67	1.83E-19	1.5E-19	0.84	0.11	2.56E-03	0.53	0.04	1.54E-03	0.59	0.11	1.82E-02	1.07	0.13	7.54E-04	0.47	0.14	1.11E-01	0.77	0.21	4.41E-02	0.79	0.10	2.43E-03	0.83	0.03	3.68E-05
SASH1	No	Yes	No	Up	1.67	6.06E-05	3.5E-05	1.48	0.12	4.63E-04	0.14	0.04	4.09E-01	0.67	0.11	3.92E-02	0.97	0.13	9.46E-03	0.89	0.15	4.99E-03	0.30	0.20	2.85E-01	0.83	0.10	1.07E-02	0.61	0.03	2.14E-02
NDC80	No	Yes	No	Up	1.67	1.83E-19	1.5E-19	1.11	0.11	8.16E-04	1.03	0.04	5.45E-06	0.54	0.11	3.45E-02	0.92	0.12	3.82E-03	0.52	0.14	2.92E-01	0.48	0.21	3.25E-01	0.68	0.10	1.03E-02	0.63	0.03	2.02E-03
TRAM1L1	No	Yes	No	Up	1.67	9.46E-05	1.5E-19	0.81	0.11	6.13E-03	0.67	0.04	3.25E-04	0.67	0.11	2.04E-02	0.30	0.11	2.38E-01	0.68	0.14	5.07E-01	1.46	0.25	3.46E-01	0.64	0.10	1.71E-02	0.67	0.03	1.80E-03
DSE	No	No	No	Up	1.67	3.58E-06	validated	0.04	0.10	8.82E-01	0.75	0.04	3.81E-04	0.49	0.10	9.05E-02	1.45	0.14	5.53E-04	0.88	0.15	5.74E-03	0.83	0.22	1.83E-02	1.19	0.10	1.74E-04	0.28	0.03	5.54E-02
ZYG11A	No	Yes	No	Up	1.67	3.97E-05	1.5E-19	0.48	0.10	4.48E-02	0.74	0.04	1.47E-04	0.64	0.11	2.26E-02	0.36	0.11	1.58E-01	1.43	0.17	5.45E-01	0.68	0.21	7.67E-01	0.99	0.10	1.88E-03	0.58	0.03	4.92E-04
PLEKHA4	No	Yes	No	Up	1.67	2.40E-05	1.5E-05	0.60	0.10	2.29E-02	0.63	0.04	1.07E-03	0.34	0.10	2.30E-01	0.33	0.11	2.26E-01	1.49	0.17	5.68E-04	1.50	0.26	1.38E-03	0.61	0.10	1.33E-01	0.41	0.03	2.64E-02
EGFL6	No	Yes	No	Up	1.67	1.83E-19	1.5E-19	1.36	0.12	1.00E-05	1.06	0.04	1.36E-06	0.64	0.11	1.39E-02	0.53	0.12	5.09E-02	0.57	0.14	3.13E-02	0.80	0.22	1.74E-02	0.28	0.10	1.92E-01	0.68	0.03	2.93E-04
USP51	No	No	No	Up	1.67	1.14E-05	4.3E-05	0.93	0.11	2.70E-03	0.65	0.04	1.22E-03	1.04	0.11	2.59E-03	1.28	0.13	3.73E-04	0.18	0.14	8.65E-01	0.99	0.22	5.46E-01	0.20	0.10	4.48E-01	0.66	0.03	5.50E-04
GSPT2	No	No	No	Up	1.67	1.83E-19	0.00097	0.67	0.11	2.19E-02	0.15	0.04	3.14E-01	0.54	0.11	3.06E-02	0.85	0.12	4.25E-03	1.12	0.16	2.55E-03	1.13	0.23	9.20E-03	0.78	0.10	4.52E-03	0.70	0.03	2.50E-05
MAPRE1	No	Yes	No	Up	1.67	3.97E-05	0.00273	1.66	0.13	3.26E-04	0.65	0.04	3.37E-03	0.52	0.11	1.21E-01	0.93	0.12	3.56E-02	0.28	0.14	1.94E-01	0.81	0.22	1.53E-02	0.08	0.10	7.06E-01	1.01	0.04	9.03E-04
KCNMB3	No	No	No	Up	1.67	0.00022	0.0012	1.13	0.11	8.12E-03	1.04	0.04	1.37E-04	0.96	0.11	3.21E-02	0.56	0.12	9.40E-02	0.39	0.14	4.74E-01	0.76	0.21	1.70E-01	0.68	0.10	4.67E-02	0.42	0.03	2.18E-02
PRR3	No	No	No	Up	1.67	0.001649	0.01497	0.72	0.11	1.37E-01	0.44	0.04	8.69E-02	0.77	0.11	3.32E-02	0.56	0.12	1.55E-01	0.76	0.15	1.70E-01	1.28	0.24	5.52E-02	0.74	0.10	3.59E-02	0.66	0.03	9.71E-04
MN1	No	No	No	Up	1.67	1.14E-05	validated	0.66	0.11	5.33E-02	0.28	0.04	2.04E-01	0.71	0.11	7.43E-03	0.99	0.13	3.27E-03	1.13	0.16	2.28E-02	1.20	0.24	2.69E-02	0.13	0.10	6.68E-01	0.85	0.03	7.04E-06
CCDC3	No	Yes	No	Up	1.67	1.83E-19	5.1E-05	0.49	0.10	5.73E-02	0.48	0.04	5.09E-03	0.66	0.11	2.73E-02	0.56	0.12	4.82E-02	1.26	0.16	3.51E-04	1.52	0.26	5.92E-04	0.23	0.10	3.61E-01	0.75	0.03	1.11E-04
GTF2IRD1	No	Yes	No	Up	1.67	6.26E-05	0.0003	1.24	0.12	1.91E-03	0.83	0.04	8.92E-04	0.62	0.11	8.58E-02	0.56	0.12	1.27E-01	0.47	0.14	1.68E-01	0.00	0.20	9.83E-01	1.47	0.11	4.19E-04	0.75	0.03	1.93E-02
ILVBL	No	Yes	No	Up	1.68	2.85E-05	2.2E-05	1.47	0.12	2.86E-04	0.98	0.04	9.11E-05	1.06	0.11	1.26E-02	0.64	0.12	1.38E-01	0.49	0.14	7.66E-02	0.95	0.22	1.21E-02	0.21	0.10	3.75E-01	0.17	0.03	4.04E-01
TPST1	No	Yes	No	Up	1.68	6.46E-05	6.7E-06	0.78	0.11	7.75E-03	0.39	0.04	2.47E-02	0.67	0.11	3.56E-02	1.11	0.13	6.07E-03	0.78	0.15	2.35E-02	1.13	0.23	1.11E-02	0.45	0.10	1.44E-01	0.67	0.03	1.64E-02
TRIO	No	Yes	No	Up	1.68	0.000178	0.00164	1.13	0.11	7.84E-03	0.81	0.04	1.39E-04	0.89	0.11	1.78E-02	0.25	0.11	4.63E-01	0.92	0.15	1.30E-01	0.94	0.22	1.82E-01	0.71	0.10	2.22E-02	0.33	0.03	4.22E-02
ARL6IP6	No	Yes	No	Up	1.68	3.58E-06	0.00015	0.56	0.10	3.64E-02	0.91	0.04	1.68E-04	0.90	0.11	6.24E-03	0.86	0.12	1.36E-02	0.10	0.14	5.60E-01	0.81	0.22	2.81E-02	1.45	0.11	1.22E-04	0.40	0.03	6.25E-02
SNRPB2	No	Yes	No	Up	1.68	1.14E-05	0.00137	1.02	0.11	2.18E-02	0.92	0.04	3.64E-04	0.44	0.10	1.74E-01	0.86	0.12	3.51E-02	1.05	0.15	9.67E-04	1.38	0.25	3.90E-04	0.01	0.10	9.68E-01	0.31	0.03	6.87E-02
EIF1B	No	Yes	No	Up	1.68	0.00016	0.00028	0.95	0.11	5.07E-03	0.62	0.04	4.48E-03	1.04	0.11	1.29E-02	1.48	0.14	1.89E-03	0.03	0.14	8.54E-01	1.24	0.24	1.43E-02	0.46	0.10	8.24E-02	0.18	0.03	2.47E-01
UVRAG	No	Yes	No	Up	1.68	0.000526	4.6E-06	2.08	0.14	1.29E-05	0.50	0.04	2.63E-02	0.16	0.10	6.57E-01	0.67	0.12	1.11E-01	0.59	0.14	1.75E-01	0.93	0.22	7.46E-02	0.69	0.10	3.17E-02	0.39	0.03	1.39E-01
CDK5RAP1	No	No	Yes	Up	1.68	0.004065	0.01025	0.95	0.11	1.37E-02	0.13	0.04	4.99E-01	1.50	0.12	4.91E-03	0.59	0.12	1.79E-01	0.65	0.14	7.98E-02	1.02	0.23	4.11E-02	0.31	0.10	4.67E-01	0.85	0.03	1.84E-02
MPP6	No	Yes	Yes	Up	1.68	3.58E-06	6.7E-06	1.22	0.12	2.89E-04	0.60	0.04	3.00E-03	0.89	0.11	2.71E-03	0.21	0.11	4.37E-01	1.18	0.16	1.23E-02	0.99	0.22	2.93E-02	0.21	0.10	3.98E-01	0.69	0.03	3.06E-04
CCDC28B	No	No	No	Up	1.68	0.000125	validated	0.61	0.10	2.53E-02	0.71	0.04	8.14E-04	0.79	0.11	3.10E-02	1.28	0.13	6.09E-03	1.32	0.16	1.69E-02	0.71	0.21	1.39E-01	0.17	0.10	6.84E-01	0.42	0.03	1.50E-02
RGS9BP	No	Yes	No	Up	1.68	0.000133	1.5E-19	1.43	0.12	1.59E-05	0.80	0.04	9.79E-03	0.34	0.10	3.74E-01	0.94	0.12	2.58E-03	0.65	0.14	6.05E-01	0.61	0.21	6.47E-01	0.86	0.10	9.40E-02	0.39	0.03	1.82E-02
LSM14A	No	No	No	Up	1.68	5.61E-05	0.00199	1.11	0.11	7.81E-03	0.77	0.04	3.10E-03	0.56	0.11	1.55E-01	0.03	0.11	9.49E-01	1.09	0.15	1.05E-03	0.87	0.22	1.08E-02	0.88	0.10	9.89E-03	0.71	0.03	5.07E-02
RBM42	No	Yes	No	Up	1.68	0.000148	0.00184	1.09	0.11	5.38E-03	0.58	0.04	1.40E-02	0.30	0.10	4.23E-01	0.93	0.12	9.55E-02	1.17	0.16	4.72E-04	1.09	0.23	3.39E-03	0.16	0.10	6.43E-01	0.69	0.03	3.73E-02
PRRX2	No	Yes	No	Up	1.68	1.83E-19	1.5E-19	1.00	0.11	4.06E-04	0.80	0.04	1.32E-05	0.12	0.10	6.66E-01	0.96	0.13	7.11E-04	1.11	0.16	3.91E-03	0.35	0.20	2.72E-01	0.99	0.10	2.14E-02	0.69	0.03	1.38E-04
LY6K	No	Yes	No	Up	1.68	1.83E-19	1.5E-19	0.93	0.11	2.45E-03	0.60	0.04	1.47E-04	1.09	0.11	1.58E-04	0.55	0.12	2.48E-02	0.68	0.14	4.09E-01	1.29	0.24	2.12E-01	0.08	0.10	7.24E-01	0.79	0.03	1.57E-05
CKS1B	No	Yes	No	Up	1.69	1.83E-19	1.5E-19	1.31	0.12	4.18E-04	0.47	0.04	6.01E-03	0.77	0.11	1.47E-02	1.12	0.13	2.07E-03	0.74	0.14	7.50E-03	0.66	0.21	3.15E-02	0.24	0.10	2.80E-01	0.74	0.03	1.91E-03
PCDH10	No	Yes	No	Up	1.69	5.41E-05	1.5E-19	0.40	0.10	2.54E-01	1.27	0.04	5.91E-06	0.86	0.11	2.70E-03	0.22	0.11	4.06E-01	0.80	0.15	1.73E-01	1.17	0.23	1.43E-01	1.01	0.10	1.04E-02	0.32	0.03	4.64E-02
DSCAML1	No	Yes	No	Up	1.69	0.000127	1.5E-19	2.04	0.14	4.54E-07	0.34	0.04	7.19E-02	0.52	0.11	2.11E-01	0.57	0.12	3.21E-02	0.30	0.14	7.66E-01	1.16	0.23	3.71E-01	0.66	0.10	1.81E-01	0.46	0.03	8.62E-03
PLCD4	No	No	No	Up	1.69	3.58E-06	0.01087	1.02	0.11	3.37E-03	0.76	0.04	5.67E-04	0.09	0.10	7.69E-01	1.21	0.13	2.03E-04	0.66	0.14	3.24E-01	0.57	0.21	3.74E-01	1.11	0.10	4.22E-03	0.64	0.03	2.65E-04
MEOX1	No	Yes	No	Up	1.69	1.83E-19	1.5E-19	1.08	0.11	1.89E-04	0.74	0.04	7.17E-04	0.62	0.11	2.82E-02	0.38	0.11	1.20E-01	0.28	0.14	2.57E-01	1.21	0.24	9.40E-03	0.64	0.10	2.71E-02	1.13	0.04	1.00E-20
KIAA1586	No	No	No	Up	1.69	0.003095	validated	0.43	0.10	2.08E-01	0.39	0.04	1.18E-01	0.62	0.11	4.62E-02	1.39	0.14	4.24E-04	0.37	0.14	7.39E-01	1.81	0.28	4.26E-01	0.33	0.10	2.71E-01	0.73	0.03	2.18E-03
GRASP	No	No	No	Up	1.69	9.46E-05	0.00896	0.45	0.10	1.08E-01	0.86	0.04	4.38E-05	0.47	0.10	2.87E-01	0.60	0.12	8.24E-02	1.10	0.15	1.67E-02	1.16	0.23	6.35E-02	0.90	0.10	7.12E-02	0.52	0.03	5.86E-03
TGIF2	No	Yes	No	Up	1.69	6.71E-06	1.5E-19	0.97	0.11	1.81E-03	0.56	0.04	5.85E-03	1.18	0.11	3.43E-04	1.06	0.13	6.47E-03	0.07	0.14	6.54E-01	0.92	0.22	4.05E-02	0.56	0.10	4.80E-02	0.73	0.03	5.19E-03
NRIP3	No	Yes	No	Up	1.69	2.40E-05	1.5E-19	0.25	0.10	5.68E-01	1.12	0.04	1.79E-04	1.76	0.13	2.91E-05	0.82	0.12	2.23E-02	0.63	0.14	2.08E-01	0.57	0.21	2.10E-01	0.34	0.10	2.40E-01	0.58	0.03	5.21E-03
PBX3	No	No	No	Up	1.69	1.83E-19	0.00021	0.77	0.11	5.42E-03	0.57	0.04	1.41E-03	0.97	0.11	1.66E-03	1.31	0.14	1.16E-04	0.76	0.15	2.31E-02	0.21	0.20	4.02E-01	1.46	0.11	7.72E-06	0.02	0.03	8.74E-01
GLO1	No	Yes	No	Up	1.69	2.85E-05	0.0107	0.66	0.11	6.78E-02	0.55	0.04	5.88E-03	1.36	0.12	2.82E-03	0.36	0.11	2.94E-01	0.14	0.14	4.01E-01	1.76	0.28	5.32E-05	0.54	0.10	4.34E-02	0.70	0.03	2.08E-02
CKAP2L	No	No	No	Up	1.69	1.83E-19	0.00023	0.73	0.11	1.92E-02	0.84	0.04	5.23E-05	0.71	0.11	1.42E-02	1.15	0.13	7.68E-04	0.86	0.15	2.54E-02	0.58	0.21	1.26E-01	0.38	0.10	1.77E-01	0.83	0.03	7.34E-05
TTC9C	No	No	No	Up	1.69	0.013108	0.00454	1.52	0.12	1.28E-03	0.64	0.04	2.81E-02	0.17	0.10	5.90E-01	1.04	0.13	8.56E-02	0.67	0.14	2.05E-01	1.61	0.26	5.36E-02	0.22	0.10	4.61E-01	0.19	0.03	3.14E-01
MOCS3	No	Yes	No	Up	1.69	0.002621	7.6E-05	1.06	0.11	5.91E-03	0.62	0.04	1.37E-02	1.02	0.11	1.71E-02	1.04	0.13	4.63E-02	0.23	0.14	6.14E-01	0.77	0.21	3.58E-01	0.79	0.10	1.12E-01	0.56	0.03	3.67E-02
CCDC8	No	Yes	No	Up	1.69	1.83E-19	1.5E-19	0.65	0.11	1.12E-02	0.83	0.04	1.77E-05	0.89	0.11	3.02E-03	0.50	0.12	5.50E-02	0.66	0.14	1.85E-01	1.09	0.23	6.62E-02	0.74	0.10	2.02E-02	0.74	0.03	1.64E-04
NLGN1	No	Yes	No	Up	1.70	6.71E-06	1.5E-19	0.84	0.11	1.89E-03	0.70	0.04	5.62E-04	0.58	0.11	5.22E-02	0.67	0.12	1.21E-02	1.10	0.15	3.95E-02	0.72	0.21	1.48E-01	0.88	0.10	1.08E-01	0.60	0.03	4.55E-04
MLF1	No	Yes	No	Up	1.70	1.83E-19	1.5E-19	1.10	0.11	4.27E-05	0.57	0.04	3.43E-04	0.92	0.11	5.82E-04	0.83	0.12	2.05E-03	0.00	0.14	9.97E-01	0.86	0.22	4.65E-01	0.95	0.10	3.89E-04	0.86	0.03	1.36E-06
ABCG4	No	Yes	No	Up	1.70	0.000143	1.5E-19	1.15	0.11	2.65E-03	0.88	0.04	9.55E-04	0.10	0.10	7.84E-01	1.39	0.14	4.32E-05	0.77	0.15	2.82E-01	0.66	0.21	3.19E-01	0.82	0.10	1.17E-01	0.32	0.03	1.26E-01
BIRC5	No	Yes	No	Up	1.70	1.83E-19	1.5E-05	0.55	0.10	4.90E-02	0.73	0.04	7.77E-05	0.43	0.10	7.57E-02	0.78	0.12	1.47E-02	1.08	0.15	1.83E-03	1.36	0.25	1.25E-03	0.69	0.10	1.65E-02	0.47	0.03	5.36E-03
TMEM108	No	Yes	No	Up	1.70	1.83E-19	1.5E-19	0.70	0.11	6.28E-03	0.94	0.04	3.86E-06	1.09	0.11	4.64E-04	0.72	0.12	6.43E-03	0.14	0.14	5.81E-01	1.18	0.23	4.18E-02	0.50	0.10	4.90E-02	0.83	0.03	1.14E-06
CPNE8	No	Yes	No	Up	1.70	6.71E-06	0.00011	0.89	0.11	4.30E-03	0.57	0.04	1.91E-03	0.11	0.10	6.19E-01	0.56	0.12	7.22E-02	1.40	0.17	1.67E-03	1.14	0.23	3.68E-02	1.14	0.10	9.95E-05	0.32	0.03	3.77E-02
KCNJ12	No	Yes	No	Up	1.70	1.83E-19	1.5E-19	1.15	0.11	6.00E-05	0.91	0.04	2.95E-06	0.47	0.10	7.36E-02	0.71	0.12	5.08E-03	1.07	0.15	3.73E-01	0.54	0.21	3.16E-01	0.65	0.10	3.66E-02	0.62	0.03	3.51E-04
SLC26A10	No	No	No	Up	1.70	6.66E-05	validated	0.18	0.10	5.18E-01	0.78	0.04	3.36E-05	0.67	0.11	6.47E-02	0.89	0.12	2.90E-03	1.02	0.15	3.14E-01	1.34	0.24	2.98E-01	0.52	0.10	1.28E-01	0.73	0.03	7.11E-04
KIF23	No	Yes	No	Up	1.70	3.58E-06	5.4E-05	1.00	0.11	1.84E-03	0.80	0.04	1.37E-04	0.49	0.10	4.85E-02	0.76	0.12	3.78E-02	0.79	0.15	8.92E-02	0.83	0.22	8.86E-02	0.88	0.10	1.65E-03	0.58	0.03	2.11E-03
MAPT	No	No	No	Up	1.70	0.000225	0.03458	0.05	0.10	8.87E-01	0.58	0.04	2.47E-03	0.26	0.10	5.06E-01	1.72	0.15	1.36E-06	1.28	0.16	1.35E-01	1.15	0.23	1.90E-01	0.93	0.10	2.98E-02	0.17	0.03	2.57E-01
PFDN4	No	Yes	No	Up	1.70	1.14E-05	0.00038	0.92	0.11	1.87E-02	1.19	0.04	2.84E-05	1.27	0.12	5.95E-04	0.80	0.12	5.60E-02	0.31	0.14	6.00E-01	0.42	0.20	5.69E-01	0.90	0.10	5.83E-03	0.33	0.03	5.01E-02
KIAA1524	No	Yes	No	Up	1.70	1.83E-19	6.7E-06	0.85	0.11	2.22E-02	0.76	0.04	1.04E-03	0.36	0.10	1.58E-01	1.23	0.13	4.77E-04	0.52	0.14	2.06E-01	0.38	0.20	3.49E-01	1.10	0.10	4.16E-04	0.94	0.03	1.74E-04
MCM2	No	Yes	No	Up	1.71	1.83E-19	0.00024	0.97	0.11	4.79E-03	0.65	0.04	6.37E-04	0.46	0.10	8.65E-02	1.40	0.14	5.47E-04	0.49	0.14	6.98E-02	0.99	0.22	7.25E-03	0.31	0.10	1.89E-01	0.90	0.03	5.97E-05
EFEMP2	No	Yes	No	Up	1.71	1.83E-19	0.00097	0.93	0.11	1.44E-03	0.39	0.04	2.18E-02	0.91	0.11	2.96E-03	0.64	0.12	3.08E-02	0.79	0.15	7.96E-03	1.48	0.25	5.00E-04	0.43	0.10	1.06E-01	0.59	0.03	4.10E-03
ZNF512	No	Yes	No	Up	1.71	3.58E-06	1.5E-19	1.02	0.11	7.94E-04	0.54	0.04	5.46E-03	1.16	0.11	1.07E-03	0.86	0.12	8.30E-03	0.64	0.14	4.33E-02	1.33	0.24	2.28E-03	0.00	0.10	9.95E-01	0.61	0.03	7.40E-03
NRAS	No	Yes	No	Up	1.71	3.58E-06	0.00353	1.18	0.11	1.24E-03	0.97	0.04	4.91E-05	0.68	0.11	4.02E-02	0.70	0.12	3.52E-02	0.25	0.14	3.08E-01	1.02	0.23	2.64E-02	0.57	0.10	3.03E-02	0.80	0.03	2.74E-03
CEBPZ	No	Yes	No	Up	1.71	0.000334	0.00251	0.82	0.11	3.61E-02	0.66	0.04	1.32E-02	0.93	0.11	2.63E-02	0.77	0.12	1.79E-01	0.15	0.14	4.17E-01	1.12	0.23	6.06E-03	1.19	0.10	1.55E-03	0.55	0.03	6.54E-02
CDH18	No	Yes	No	Up	1.71	1.83E-19	1.5E-19	1.29	0.12	3.86E-06	0.88	0.04	1.36E-06	0.21	0.10	3.68E-01	0.90	0.12	9.44E-04	0.71	0.14	5.77E-02	0.91	0.22	4.47E-02	0.73	0.10	1.27E-02	0.55	0.03	1.14E-03
PHACTR3	No	Yes	No	Up	1.71	1.83E-19	1.5E-19	0.40	0.10	1.33E-01	1.01	0.04	2.73E-06	0.41	0.10	3.40E-01	1.40	0.14	1.07E-05	0.96	0.15	7.27E-03	0.66	0.21	6.69E-02	0.71	0.10	8.44E-02	0.64	0.03	2.84E-04
HIC2	No	Yes	No	Up	1.71	1.83E-19	1.5E-19	1.20	0.11	5.21E-04	1.27	0.04	1.36E-06	1.02	0.11	2.84E-03	0.16	0.11	6.32E-01	0.72	0.14	8.19E-02	0.18	0.20	5.52E-01	1.53	0.11	1.79E-05	0.12	0.03	4.94E-01
PGBD1	No	No	No	Up	1.71	0.00016	0.02607	0.29	0.10	3.02E-01	0.20	0.04	4.20E-01	0.84	0.11	8.74E-03	1.94	0.16	8.95E-05	0.44	0.14	3.37E-01	1.47	0.25	3.76E-02	0.06	0.10	8.36E-01	0.95	0.03	1.31E-04
C2ORF56	No	Yes	No	Up	1.71	0.000108	0.00159	1.41	0.12	8.25E-04	1.37	0.04	2.54E-05	0.58	0.11	9.42E-02	0.50	0.12	3.04E-01	0.40	0.14	2.10E-01	1.18	0.23	1.65E-02	0.57	0.10	7.71E-02	0.18	0.03	4.66E-01
ZBTB39	No	Yes	No	Up	1.71	0.00146	0.00122	1.15	0.11	2.33E-03	0.51	0.04	3.82E-02	1.13	0.11	1.16E-02	0.63	0.12	1.00E-01	0.44	0.14	5.08E-01	0.74	0.21	2.56E-01	1.04	0.10	2.94E-02	0.57	0.03	3.82E-02

Supplementary Table 2. Details for 2,053 genes differentially expressed between EEC and NEEC, identified by microarray meta-analysis.

EEC - endometrioid endometrial cancer, NEEC - non-endometrioid endometrial cancer, SMD - Standardised Mean Difference, FDR - False Discovery Rate, Var - Variance

Gene	145 gene list	1253 gene list	Associated with EEC-specific survival?	Up/Down Regulated in NEEC	average standardised fold change	Microarray Meta FDR	RNASeq FDR	Individual microarray study results: TCGA			E-MTAB-2532			E-GEOD-56026			GSE32507			GSE24537			E-GEOD-23518			E-GEOD-17025			E-GEOD-2109		
								SMD	Var	P-value	SMD	Var	P-value	SMD	Var	P-value	SMD	Var	P-value	SMD	Var	P-value	SMD	Var	P-value	SMD	Var	P-value	SMD	Var	P-value
ARL10	No	No	No	Up	1.71	0.000108	0.00174	0.02	0.10	9.58E-01	0.25	0.04	3.77E-01	1.36	0.12	2.36E-04	1.26	0.13	5.54E-04	1.10	0.15	4.38E-01	1.11	0.23	4.35E-01	0.36	0.10	1.58E-01	0.76	0.03	4.91E-05
LATS2	No	Yes	No	Up	1.71	6.71E-06	0.00023	1.34	0.12	6.94E-04	0.56	0.04	7.57E-03	0.95	0.11	4.39E-03	0.54	0.12	1.32E-01	0.68	0.14	1.07E-01	0.14	0.20	5.96E-01	1.65	0.11	2.59E-05	0.37	0.03	3.68E-02
SEMA6C	No	Yes	No	Up	1.72	1.14E-05	1.9E-05	0.68	0.11	1.14E-02	0.77	0.04	1.55E-04	0.23	0.10	5.76E-01	0.81	0.12	1.64E-02	1.55	0.17	1.35E-02	0.76	0.21	5.37E-02	0.88	0.10	4.45E-02	0.55	0.03	2.73E-03
ITGA7	No	Yes	No	Up	1.72	1.83E-19	1.5E-19	1.80	0.13	2.50E-06	0.85	0.04	2.23E-05	0.36	0.10	1.95E-01	0.83	0.12	8.62E-03	1.04	0.15	2.92E-01	0.41	0.20	6.30E-01	0.12	0.10	6.51E-01	0.82	0.03	6.36E-06
SLC43A3	No	Yes	No	Up	1.72	3.58E-06	1.5E-19	0.98	0.11	9.77E-04	0.44	0.04	1.17E-02	0.66	0.11	1.68E-02	0.69	0.12	1.95E-02	1.44	0.17	2.45E-03	0.75	0.21	7.45E-02	0.57	0.10	6.49E-02	0.71	0.03	1.33E-03
EGFLAM	No	No	No	Up	1.72	1.83E-19	validated	1.13	0.11	1.26E-03	0.83	0.04	3.11E-05	0.06	0.10	8.55E-01	1.25	0.13	1.42E-04	0.92	0.15	2.45E-03	0.69	0.21	3.04E-02	0.38	0.10	2.08E-01	0.99	0.03	2.04E-06
PTPN13	No	Yes	No	Up	1.72	3.58E-06	1.5E-19	1.06	0.11	1.16E-04	0.43	0.04	1.09E-02	0.72	0.11	6.96E-03	0.52	0.12	6.71E-02	0.89	0.15	4.65E-02	1.22	0.24	5.05E-02	0.85	0.10	3.12E-03	0.55	0.03	6.16E-03
SLC29A1	No	No	No	Up	1.72	0.000167	validated	0.81	0.11	3.09E-02	0.38	0.04	5.16E-02	0.18	0.10	5.50E-01	0.47	0.12	1.93E-01	1.65	0.18	2.55E-04	1.29	0.24	5.20E-03	1.14	0.10	5.92E-03	0.33	0.03	1.17E-01
IFIT1	No	Yes	No	Up	1.72	1.83E-19	1.5E-19	1.22	0.12	1.73E-05	0.91	0.04	2.27E-06	1.10	0.11	1.00E-04	0.40	0.12	1.17E-01	0.99	0.15	1.06E-03	0.49	0.21	8.60E-02	0.51	0.10	2.47E-02	0.62	0.03	3.14E-04
FAM64A	No	No	No	Up	1.72	1.83E-19	0.00103	0.34	0.10	2.22E-01	0.78	0.04	4.84E-05	0.73	0.11	2.07E-02	1.10	0.13	2.22E-03	0.62	0.14	6.67E-02	1.06	0.23	1.90E-02	0.87	0.10	9.47E-03	0.73	0.03	8.59E-04
CRYAB	No	Yes	No	Up	1.72	1.83E-19	1.5E-19	1.47	0.12	7.04E-06	0.55	0.04	1.01E-03	0.79	0.11	2.48E-03	0.49	0.12	5.55E-02	1.30	0.16	1.13E-04	0.60	0.21	4.51E-02	0.36	0.10	2.34E-01	0.69	0.03	1.38E-04
MSH6	No	Yes	No	Up	1.72	1.14E-05	0.00055	0.83	0.11	1.64E-02	0.91	0.04	3.10E-04	0.74	0.11	1.28E-02	1.37	0.14	5.42E-03	1.13	0.16	1.12E-03	0.73	0.21	3.06E-02	0.48	0.10	7.00E-02	0.08	0.03	5.79E-01
PLAGL1	No	Yes	No	Up	1.72	1.83E-19	1.5E-19	0.91	0.11	1.05E-02	0.77	0.04	6.84E-05	0.33	0.10	1.68E-01	1.00	0.13	1.38E-03	0.93	0.15	5.85E-02	0.80	0.22	5.42E-02	1.13	0.10	9.79E-05	0.39	0.03	3.12E-02
CCNE1	No	Yes	No	Up	1.72	1.83E-19	1.5E-19	1.66	0.13	6.82E-07	0.93	0.04	2.73E-06	0.83	0.11	2.39E-03	0.60	0.12	6.34E-02	0.56	0.14	3.25E-02	0.48	0.21	1.04E-01	0.86	0.10	1.47E-03	0.35	0.03	3.75E-02
BCAS4	No	No	No	Up	1.72	2.40E-05	validated	1.63	0.13	2.29E-04	0.33	0.04	4.89E-02	0.62	0.11	6.52E-02	0.98	0.13	3.30E-03	0.90	0.15	1.35E-02	0.88	0.22	2.09E-02	0.58	0.10	1.55E-01	0.35	0.03	3.02E-02
COL23A1	No	Yes	No	Up	1.72	1.83E-19	1.5E-19	0.86	0.11	1.09E-03	0.89	0.04	2.73E-06	0.35	0.10	1.60E-01	0.65	0.12	1.52E-02	0.90	0.15	2.09E-02	0.77	0.21	5.47E-02	0.84	0.10	1.03E-02	1.03	0.04	6.82E-07
FAM43A	No	No	No	Up	1.72	1.83E-19	0.04	0.64	0.11	4.49E-02	0.93	0.04	1.23E-04	0.99	0.11	1.51E-03	1.59	0.15	5.02E-05	0.61	0.14	3.64E-02	0.02	0.20	8.76E-01	0.87	0.10	3.74E-03	0.64	0.03	3.33E-04
GABBR1	No	Yes	No	Up	1.72	1.14E-05	2.3E-06	0.74	0.11	7.63E-02	0.72	0.04	3.77E-04	1.12	0.11	1.53E-03	0.40	0.12	1.75E-01	1.24	0.16	8.74E-03	1.05	0.23	3.46E-02	0.27	0.10	2.88E-01	0.75	0.03	5.24E-04
SNAP25	No	Yes	No	Up	1.73	1.83E-19	1.5E-19	0.74	0.11	1.34E-02	0.97	0.04	1.68E-04	0.80	0.11	4.31E-03	0.81	0.12	7.75E-03	0.41	0.14	1.31E-01	0.57	0.21	9.87E-02	1.21	0.10	4.48E-04	0.79	0.03	5.45E-06
ABCA3	No	Yes	No	Up	1.73	1.83E-19	1.5E-19	1.17	0.11	1.12E-04	1.18	0.04	1.00E-20	0.65	0.11	4.69E-02	0.87	0.12	2.32E-03	0.81	0.15	8.67E-02	0.10	0.20	6.99E-01	1.01	0.10	7.90E-03	0.51	0.03	6.14E-03
CPNE1	No	Yes	No	Up	1.73	1.41E-05	0.00013	1.06	0.11	3.83E-03	0.60	0.04	2.85E-03	0.86	0.11	1.99E-02	0.85	0.12	3.21E-02	0.80	0.15	1.01E-02	0.65	0.21	3.99E-02	1.17	0.10	4.48E-03	0.31	0.03	1.71E-01
SALL2	No	Yes	No	Up	1.73	1.14E-05	0.00038	0.30	0.10	2.03E-01	0.49	0.04	4.45E-03	0.81	0.11	1.23E-02	0.83	0.12	1.82E-02	0.93	0.15	1.05E-02	2.13	0.31	1.04E-04	0.17	0.10	4.98E-01	0.65	0.03	3.72E-03
GFOD1	No	Yes	No	Up	1.73	1.83E-19	1.5E-19	0.61	0.10	1.85E-02	1.19	0.04	4.54E-07	1.21	0.11	4.83E-04	0.53	0.12	7.73E-02	1.00	0.15	5.11E-03	0.62	0.21	9.62E-02	0.38	0.10	1.64E-01	0.76	0.03	3.67E-03
FAM133A	No	Yes	No	Up	1.73	1.83E-19	1.5E-19	1.55	0.12	5.00E-06	0.74	0.04	2.17E-04	0.47	0.10	9.25E-02	0.79	0.12	3.01E-03	0.59	0.14	1.50E-01	0.62	0.21	1.51E-01	0.96	0.10	6.54E-03	0.59	0.03	4.03E-04
REEP2	No	No	No	Up	1.73	1.83E-19	0.00178	0.92	0.11	1.91E-03	0.99	0.04	1.59E-06	0.36	0.10	4.05E-01	1.35	0.14	2.05E-04	0.51	0.14	5.59E-01	0.46	0.21	6.70E-01	1.04	0.10	8.04E-02	0.69	0.03	4.27E-04
CLCN4	No	Yes	No	Up	1.73	1.66E-05	1.5E-19	1.17	0.11	3.78E-04	0.77	0.04	9.09E-03	0.66	0.11	1.63E-02	1.08	0.13	2.00E-03	0.90	0.15	5.25E-01	0.87	0.22	4.83E-01	0.33	0.10	1.70E-01	0.55	0.03	4.83E-04
CCND3	No	Yes	No	Up	1.73	2.40E-05	0.0125	1.02	0.11	2.97E-02	0.65	0.04	3.23E-03	0.91	0.11	2.04E-02	1.45	0.14	8.93E-04	0.58	0.14	3.00E-02	0.41	0.20	1.41E-01	1.06	0.10	4.61E-03	0.24	0.03	1.23E-01
KCTD15	No	No	No	Up	1.73	1.14E-05	0.00311	0.25	0.10	3.60E-01	0.36	0.04	3.36E-02	0.74	0.11	5.08E-03	1.57	0.15	8.63E-06	0.76	0.15	9.19E-02	1.83	0.28	1.13E-02	0.64	0.10	1.20E-02	0.17	0.03	2.40E-01
TP53BP2	No	Yes	No	Up	1.73	2.85E-05	0.0051	1.00	0.11	3.82E-03	0.54	0.04	1.09E-02	0.91	0.11	1.85E-02	0.60	0.12	1.45E-01	1.40	0.17	7.35E-04	0.96	0.22	1.44E-02	0.09	0.10	7.10E-01	0.84	0.03	6.63E-03
KIAA1429	No	No	No	Up	1.73	0.000269	0.04454	1.42	0.12	1.54E-03	0.10	0.04	6.19E-01	0.54	0.11	1.36E-01	1.62	0.15	1.38E-03	0.74	0.14	3.98E-02	0.70	0.21	7.33E-02	0.50	0.10	1.45E-01	0.70	0.03	5.26E-03
MFAP4	No	No	No	Up	1.73	1.83E-19	0.01951	1.33	0.12	1.66E-05	0.49	0.04	1.15E-03	0.68	0.11	1.58E-02	1.05	0.13	3.44E-04	0.85	0.15	3.01E-03	0.99	0.22	3.93E-03	0.06	0.10	8.41E-01	0.89	0.03	1.00E-20
RIMS3	No	Yes	No	Up	1.73	1.83E-19	1.5E-19	0.59	0.10	4.32E-02	0.97	0.04	1.20E-05	1.05	0.11	2.56E-03	0.92	0.12	1.39E-03	1.04	0.15	2.08E-02	0.43	0.20	2.53E-01	0.74	0.10	3.13E-02	0.60	0.03	1.65E-03
ATP6V1G2	No	No	No	Up	1.73	0.000103	1.5E-19	1.15	0.11	4.93E-03	0.83	0.04	3.35E-04	0.40	0.10	3.58E-01	1.33	0.14	1.27E-03	0.71	0.14	2.06E-01	0.95	0.22	1.96E-01	0.33	0.10	4.46E-01	0.65	0.03	4.74E-03
RNPS1	No	Yes	No	Up	1.74	3.97E-05	0.04521	0.71	0.11	9.94E-02	0.63	0.04	3.70E-03	0.91	0.11	7.05E-02	0.26	0.11	3.97E-01	1.27	0.16	2.59E-04	1.42	0.25	5.29E-04	0.98	0.10	1.46E-02	0.17	0.03	4.50E-01
AP3B2	No	Yes	No	Up	1.74	1.14E-05	1.5E-19	1.06	0.11	2.75E-03	1.25	0.04	7.50E-06	0.60	0.11	1.72E-01	1.00	0.13	1.31E-03	0.22	0.14	6.29E-01	1.25	0.24	1.12E-01	0.56	0.10	2.77E-01	0.42	0.03	7.36E-03
ZFHX4	No	No	No	Up	1.74	1.83E-19	0.00012	0.48	0.10	4.59E-02	1.38	0.04	1.00E-20	0.56	0.11	6.67E-02	1.70	0.15	2.27E-07	0.80	0.15	3.47E-02	0.76	0.21	5.85E-02	0.38	0.10	4.37E-01	0.30	0.03	4.84E-02
SLC4A3	No	No	No	Up	1.74	1.14E-05	0.0249	0.82	0.11	2.92E-03	0.69	0.04	1.53E-04	1.01	0.11	9.59E-03	0.31	0.11	2.27E-01	0.88	0.15	1.30E-01	1.36	0.25	1.89E-01	0.93	0.10	3.80E-03	0.38	0.03	1.79E-02
DZIP1	No	No	No	Up	1.74	1.83E-19	0.0029	0.72	0.11	8.56E-03	0.77	0.04	1.19E-04	0.86	0.11	1.78E-03	1.00	0.13	1.36E-03	0.31	0.14	4.68E-01	1.22	0.24	1.44E-01	0.66	0.10	1.16E-02	0.84	0.03	1.26E-04
TAF1B	No	Yes	No	Up	1.74	0.000265	0.00012	1.21	0.11	4.09E-03	0.34	0.04	1.01E-01	1.27	0.12	1.82E-03	0.74	0.12	1.22E-01	0.84	0.15	7.73E-02	0.55	0.21	1.27E-01	1.22	0.10	1.35E-03	0.20	0.03	3.10E-01
CHRAC1	No	Yes	No	Up	1.74	4.80E-05	0.00071	1.52	0.12	2.33E-04	0.91	0.04	4.38E-04	1.22	0.11	1.35E-02	0.90	0.12	2.00E-02	0.51	0.14	2.43E-01	0.01	0.20	9.73E-01	0.50	0.10	8.72E-02	0.83	0.03	2.21E-02
MYOZ1	No	Yes	No	Up	1.74	1.83E-19	1.5E-19	1.35	0.12	8.61E-05	0.98	0.04	7.66E-05	0.34	0.10	2.33E-01	0.79	0.12	5.93E-03	0.42	0.14	3.08E-01	1.30	0.24	2.61E-02	0.20	0.10	6.13E-01	1.01	0.04	1.14E-06
CHCHD7	No	Yes	No	Up	1.74	1.83E-19	3.1E-05	2.45	0.16	4.54E-07	0.93	0.04	3.27E-05	1.00	0.11	2.44E-03	0.94	0.12	1.37E-02	0.06	0.14	7.31E-01	0.13	0.20	5.74E-01	0.44	0.10	9.82E-02	0.46	0.03	4.19E-02
DUSP9	No	Yes	No	Up	1.74	2.85E-05	1.5E-19	0.52	0.10	1.41E-01	0.92	0.04	2.07E-04	1.54	0.12	1.56E-03	0.46	0.12	2.60E-01	0.49	0.14	5.16E-01	0.44	0.20	5.39E-01	1.39	0.11	5.53E-03	0.64	0.03	3.48E-04
PLAC1	No	Yes	No	Up	1.74	1.83E-19	1.5E-19	1.07	0.11	2.14E-04	1.18	0.04	4.54E-06	0.29	0.10	4.99E-01	0.93	0.12	1.53E-03	0.50	0.14	4.60E-01	1.01	0.23	1.90E-01	0.84	0.10	6.28E-02	0.59	0.03	4.74E-04
PDCL2	No	Yes	No	Up	1.74	1.83E-19	1.5E-19	1.69	0.13	4.54E-07	0.78	0.04	8.15E-04	0.57	0.11	9.03E-02	0.60	0.12	3.58E-02	0.46	0.14	7.01E-01	0.27	0.20	8.84E-01	1.49	0.11	1.67E-03	0.56	0.03	3.36E-04
BACH2	No	Yes	No	Up	1.74	1.83E-19	1.9E-05	1.19	0.11	2.73E-04	0.88	0.04	5.88E-05	0.94	0.11	2.21E-03	1.33	0.14	1.27E-04	0.52	0.14	3.18E-01	0.36	0.20	4.34E-01	0.52	0.10	7.94E-02	0.68	0.03	1.77E-03
FKBP1B	No	Yes	No	Up	1.75	1.83E-19	1.5E-19	0.78	0.11	3.14E-03	0.95	0.04	6.13E-06	0.97	0.11	9.25E-04	1.09	0.13	1.37E-03	0.64	0.14	4.89E-01	1.14	0.23	2.44E-01	0.50	0.10	4.34E-02	0.37	0.03	4.79E-02
NRM	No	No	No	Up	1.75	0.000143	validated	0.49	0.10	1.05E-01	0.93	0.04	2.61E-04	0.40	0.10	2.44E-01	1.30	0.14	3.97E-03	1.04	0.15	4.08E-02	1.05	0.23	5.67E-02	0.50	0.10	1.30E-01	0.73	0.03	1.42E-02
KIFC1	No	Yes	No	Up	1.75	1.83E-19	6.4E-05	1.16	0.11	4.38E-04	0.99	0.04	3.18E-06	0.54	0.11	7.67E-02	0.63	0.12	4.14E-02	0.99	0.15	2.25E-03	1.13	0.23	3.78E-03	0.81	0.10	1.96E-02	0.20	0.03	1.80E-01
ADCY3	No	Yes	No	Up	1.75	1.14E-05	0.0059	0.34	0.10	2.30E-01	0.44	0.04	2.44E-02	0.97	0.11	4.92E-03	1.05	0.13	1.15E-02	1.09	0.15	5.90E-04	0.65	0.21	3.91E-02	1.17	0.10	9.56E-03	0.73	0.03	5.51E-03
NUAK1	No	Yes	No	Up	1.75	1.83E-19	0.0008	0.89	0.11	1.40E-02	0.60	0.04	1.24E-03	0.93	0.11	4.03E-03	1.02	0.13	5.41E-03	1.11	0.16	8.25E-04	0.18	0.20	4.12E-01	0.72	0.10	1.57E-02	1.01	0.04	5.47E-05
SHKBP1	No	Yes	No	Up	1.75	0.000418	1.5E-19	0.89	0.11	2.23E-02	0.69	0.04	2.82E-03	0.69	0.11	4.48E-02	1.48	0.14	4.29E-03	0.98	0.15	2.70E-02	1.04	0.23	3.63E-02	0.49	0.10	2.34E-01	0.19	0.03	3.30E-01
DDX27	No	Yes	Yes	Up	1.75	1.14E-05	2E-05	1.40	0.12	5.90E-04	1.28	0.04	1.20E-05	1.63	0.12	5.77E-04	0.21	0.11	6.97E-01	0.40	0.14	2.13E-01	0.48	0.21	2.56E-01	0.17	0.10	5.84E-01	0.90	0.03	3.51E-03
SKP2	No	No	No	Up	1.75	1.83E-19	validated	0.86	0.11	1.38E-02	0.83	0.04	3.10E-04	0.69	0.11	2.25E-02	1.78	0.15	8.63E-05	0.46	0.14	8.52E-02	0.72	0.21	2.98E-02	0.65	0.10	4.04E-02	0.48	0.03	2.90E-03
GLT8D2	No	No	No	Up	1.75	1.83E-19	validated	1.16	0.11	2.76E-04	0.38	0.04	2.32E-02	0.27	0.10	2.25E-01	1.34	0.14	6.20E-05	0.92	0.15	6.54E-03	1.17	0.23	4.88E-03	0.66	0.10	1.18E-02	0.57	0.03	1.79E-03
MGAT4A	No	Yes	No	Up	1.75	1.83E-19	1.5E-19	1.69	0.13	3.41E-06	0.42	0.04	1.77E-02	0.60	0.11	3.43E-02	0.44	0.12	2.21E-01	1.42	0.17	7.39E-04	0.43	0.20	1.76E-01	0.81	0.10	6.06E-03	0.68	0.03	9.24E-04
DSN1	No	Yes	No	Up	1.75	1.14E-05	0.00181	0.70	0.11	2.82E-02	0.97	0.04	1.10E-04	0.76	0.11	1.48E-02	1.26	0.13	5.16E-03	0.51	0.14	1.54E-01	0.44	0.20	2.97E-01	1.15	0.10	4.93E-03	0.68	0.03	8.52E-03
TNFAIP6	No	No	No	Up	1.75	1.83E-19	0.01692	0.79	0.11	5.41E-03	0.80	0.04	3.79E-05	0.29	0.10	1.89E-01	1.11	0.13	6.36E-04	0.68	0.14	2.68E-02	0.66	0.21	4.93E-02	1.23	0.10	2.71E-04	0.90	0.03	3.86E-06
DCUN1D2	No	Yes	No	Up	1.75	0.000125	0.0011	1.28	0.12	1.81E-03	0.61	0.04	3.14E-03	1.44	0.12	3.24E-03	1.25	0.13	2.33E-03	0.27	0.14	5.34E-01	0.87	0.22	1.59E-01	0.32	0.10	2.69E-01	0.44	0.03	9.51E-02
CUZD1	No	Yes	No	Up	1.75	3.97E-05	1.5E-19	1.41	0.12	4.18E-05	0.95	0.04	1.40E-04	0.14	0.10	6.64E-01	0.55	0.12	3.59E-02	0.27	0.14	5.58E-01	2.03	0.30	3.33E-01	0.54	0.10	1.79E-01	0.59	0.03	2.00E-03
SNRPC	No	No	No	Up	1.75	0.000269	0.00993	1.23	0.12	1.80E-02	0.66	0.04	1.34E-02	0.88	0.11	3.95E-02	1.07	0.13	7.97E-03	0.58	0.14	3.98E-02	0.97	0.22	9.64E-03	0.44	0.10	2.18E-01	0.66	0.03	6.28E-02
PSRC1	No	Yes	No	Up	1.75	1.83E-19	0.00226	0.82	0.11	1.14E-02	0.73	0.04	5.02E-04	0.83	0.11	4.90E-03	0.96	0.13	1.84E-02	0.77	0.15	3.93E-02	0.69	0.21	1.00E-01	0.94	0.10	2.46E-03	0.75	0.03	1.92E-03
CORO6	No	Yes	No	Up	1.76	2.40E-05	1.5E-19	0.86	0.11	8.58E-03	0.96	0.04	6.59E-06	0.31	0.10	4.12E-01	0.61	0.12	3.53E-02	1.22	0.16	1.98E-01	0.90	0.22	3.27E-01	1.01	0.10	5.45E-03	0.63	0.03	1.21E-02
LRP1	No	No	No	Up	1.76	2.85E-05	validated	0.84	0.11	2.24E-02	0.42	0.04	3.58E-02	0.43	0.10	1.77E-01	1.53	0.14	2.44E-04	1.39	0.17	8.85E-03	1.10	0.23	5.33E-02	0.19	0.10	6.23E-01	0.61	0.03	5.60E-04
KIF2C	No	Yes	No	Up	1.76	1.83E-19	1.5E-19	1.08	0.11	7.02E-04	1.04	0.04	1.59E-06	0.47	0.10	6.18E-02	0.64	0.12	4.91E-02	0.98	0.15	1.02E-02	0.87	0.22	4.50E-02	0.58	0.10	3.04E-02	0.84	0.03	1.20E-04
CDV3	No	Yes	No	Up	1.76	1.83E-19	0.00295	1.61	0.13	1.58E-03	0.65	0.04	1.21E-04	0.66	0.11	2.88E-02	1.05	0.13	2.78E-03	0.27	0.14	2.46E-01	0.64	0.21	5.86E-02	1.28	0.11	8.79E-05	0.36	0.03	5.95E-02
PKN1	No	Yes	No	Up	1.76	3.58E-06	1.5E-19	1.89	0.13	2.41E-05	1.09	0.04	1.20E-05	0.86	0.11	1.62E-02	0.63	0.12	9.58E-02	0.86	0.15	1.79E-02	0.57	0.21	1.44E-01	0.41	0.10	1.80E-01	0.20	0.03	3.54E-01
CDKL2	No	Yes	No	Up	1.76	1.83E-19	1.5E-19	1.57	0.12	3.50E-05	0.78	0.04	1.72E-04	0.94	0.11	2.53E-03	0.24	0.11	2.99E-01	0.57	0.14	5.02E-01	0.83	0.22	2.90E-01	1.33	0.11	2.77E-05	0.27	0.03	1.15E-01
AURKA	No	Yes	No	Up	1.76	1.83E-19	1.5E-19	1.17	0.11	1.05E-03	0.93	0.04	6.59E-06	0.67	0.11	1.21E-02	0.88	0.12	1.15E-02	0.82	0.15	8.22E-03	0.94	0.22	1.42E-02	0.62	0.10	2.04E-02	0.49	0.03	5.35E-03

Supplementary Table 2. Details for 2,053 genes differentially expressed between EEC and NEEC, identified by microarray meta-analysis.

EEC - endometrioid endometrial cancer, NEEC - non-endometrioid endometrial cancer, SMD - Standardised Mean Difference, FDR - False Discovery Rate, Var - Variance

Gene	145 gene list	1253 gene list	Associated with EEC-specific survival?	Up/Down Regulated in NEEC	average standardised fold change	Microarray Meta FDR	RNASeq FDR	Individual microarray study results																							
								TCGA			E-MTAB-2532			E-GEOD-56026			GSE32507			GSE24537			E-GEOD-23518			E-GEOD-17025			E-GEOD-2109		
								SMD	Var	P-value	SMD	Var	P-value	SMD	Var	P-value	SMD	Var	P-value	SMD	Var	P-value	SMD	Var	P-value	SMD	Var	P-value	SMD	Var	P-value
RAE1	No	Yes	No	Up	1.76	1.14E-05	4.6E-06	1.41	0.12	2.30E-03	0.73	0.04	1.96E-03	1.19	0.11	1.64E-03	0.65	0.12	8.26E-02	0.29	0.14	1.88E-01	1.82	0.28	1.05E-04	0.11	0.10	6.71E-01	0.33	0.03	5.05E-02
TRAM2	No	Yes	No	Up	1.76	6.46E-05	0.00036	1.23	0.12	1.41E-02	0.68	0.04	2.80E-03	1.02	0.11	6.02E-03	0.45	0.12	1.79E-01	0.96	0.15	2.97E-02	1.36	0.25	2.21E-02	0.28	0.10	3.28E-01	0.56	0.03	2.77E-03
CSNK2A1	No	Yes	No	Up	1.76	8.51E-05	0.00024	1.95	0.14	6.26E-04	0.85	0.04	1.05E-03	0.99	0.11	7.20E-03	0.80	0.12	1.07E-01	0.31	0.14	2.42E-01	1.00	0.22	9.74E-03	0.51	0.10	7.14E-02	0.12	0.03	4.57E-01
GLDC	No	Yes	No	Up	1.76	1.83E-19	1.5E-19	1.24	0.12	3.68E-05	0.63	0.04	5.95E-05	0.97	0.11	3.66E-04	0.09	0.11	7.05E-01	1.26	0.16	2.54E-04	0.45	0.21	1.07E-01	1.43	0.11	4.77E-06	0.47	0.03	2.40E-03
TRIB3	No	Yes	No	Up	1.76	1.83E-19	8.5E-05	0.60	0.10	2.45E-02	1.02	0.04	1.36E-06	1.17	0.11	5.09E-04	0.70	0.12	1.99E-02	0.94	0.15	7.17E-03	1.28	0.24	6.39E-03	0.53	0.10	5.61E-02	0.29	0.03	4.94E-02
BHMT2	No	No	No	Up	1.76	3.58E-06	0.00024	1.66	0.13	6.82E-07	0.51	0.04	4.35E-03	0.70	0.11	1.54E-02	0.15	0.11	5.10E-01	1.51	0.17	7.27E-02	0.68	0.21	3.43E-01	1.04	0.10	1.53E-03	0.30	0.03	5.09E-02
PLXNA1	No	Yes	No	Up	1.76	6.71E-06	0.00155	0.92	0.11	1.81E-02	1.00	0.04	4.13E-05	0.95	0.11	7.85E-03	1.22	0.13	6.89E-03	0.46	0.14	1.35E-01	0.78	0.22	7.87E-02	0.89	0.10	7.82E-03	0.33	0.03	4.70E-02
MXRA8	No	Yes	No	Up	1.77	1.83E-19	0.00148	1.26	0.12	1.45E-04	0.56	0.04	8.93E-04	0.64	0.11	3.12E-02	1.36	0.14	1.48E-04	0.28	0.14	3.72E-01	1.41	0.25	9.59E-03	0.55	0.10	5.62E-02	0.51	0.03	3.49E-03
MED30	No	Yes	No	Up	1.77	6.71E-06	0.00016	1.06	0.11	3.22E-03	0.47	0.04	2.99E-02	1.32	0.12	4.06E-04	1.48	0.14	7.28E-04	0.42	0.14	1.09E-01	1.37	0.25	1.20E-03	0.23	0.10	3.31E-01	0.22	0.03	1.68E-01
PLAGL2	No	Yes	No	Up	1.77	1.83E-19	1.5E-19	1.48	0.12	2.79E-05	0.85	0.04	2.77E-04	0.92	0.11	7.61E-03	0.30	0.11	4.23E-01	0.63	0.14	5.07E-02	1.13	0.23	4.03E-03	0.46	0.10	1.17E-01	0.80	0.03	1.60E-03
TKTL1	No	Yes	Yes	Up	1.77	1.83E-19	1.5E-19	0.98	0.11	1.13E-03	0.71	0.04	1.17E-04	0.56	0.11	2.95E-02	0.43	0.12	1.88E-01	0.94	0.15	2.82E-02	0.74	0.21	1.46E-01	1.51	0.11	6.00E-05	0.72	0.03	4.16E-05
EMILIN3	No	No	No	Up	1.77	0.000634	validated	0.28	0.10	5.20E-01	0.77	0.04	6.81E-04	1.00	0.11	1.08E-02	1.02	0.13	8.36E-03	0.82	0.15	2.28E-01	1.55	0.26	1.41E-01	0.78	0.10	3.49E-02	0.35	0.03	6.20E-02
ADAMTS3	No	No	No	Up	1.77	1.83E-19	validated	1.03	0.11	4.80E-03	0.92	0.04	4.11E-05	0.81	0.11	5.91E-03	0.77	0.12	6.69E-03	0.15	0.14	8.88E-01	0.72	0.21	5.86E-01	1.22	0.10	1.03E-03	0.97	0.03	1.27E-05
LGI2	No	Yes	No	Up	1.77	1.83E-19	1.5E-19	1.08	0.11	1.20E-04	0.41	0.04	1.78E-02	0.50	0.10	1.59E-01	0.80	0.12	3.76E-03	1.08	0.15	5.94E-02	0.89	0.22	1.64E-01	0.84	0.10	6.21E-02	0.99	0.03	1.14E-06
TSC22D2	No	Yes	No	Up	1.77	4.80E-05	0.00015	0.81	0.11	2.86E-02	0.96	0.04	6.15E-04	0.88	0.11	1.84E-02	0.39	0.12	2.19E-01	1.05	0.15	1.53E-02	1.72	0.27	1.06E-03	0.51	0.10	1.25E-01	0.28	0.03	9.53E-02
RNF26	No	Yes	No	Up	1.77	0.000252	0.00124	1.36	0.12	2.69E-03	0.97	0.04	1.41E-03	1.00	0.11	1.14E-02	0.69	0.12	1.76E-01	0.95	0.15	3.52E-02	1.10	0.23	2.66E-02	0.09	0.10	7.85E-01	0.45	0.03	6.40E-02
CMSS1	No	Yes	Yes	Up	1.77	9.89E-06	0.00046	1.06	0.11	5.56E-03	0.85	0.04	4.97E-04	1.15	0.11	3.19E-03	0.87	0.12	3.44E-02	0.31	0.14	2.07E-01	0.82	0.22	2.84E-02	0.63	0.10	6.30E-02	0.91	0.03	2.41E-03
C20ORF4	No	No	No	Up	1.77	0.001284	0.00585	1.35	0.12	2.08E-02	0.90	0.04	7.01E-03	0.78	0.11	6.76E-02	0.70	0.12	3.08E-01	0.37	0.14	1.64E-01	1.03	0.23	1.20E-02	0.62	0.10	1.03E-01	0.85	0.03	1.93E-02
C10ORF114	No	No	No	Up	1.77	1.83E-19	5.1E-05	1.73	0.13	4.54E-07	1.35	0.04	1.00E-20	0.43	0.10	3.15E-01	0.59	0.12	2.20E-02	0.89	0.15	1.40E-01	0.55	0.21	4.03E-01	0.52	0.10	2.40E-01	0.54	0.03	5.72E-04
SEMA4F	No	No	No	Up	1.77	0.000402	validated	0.82	0.11	1.32E-02	0.43	0.04	4.16E-02	1.05	0.11	5.23E-03	0.77	0.12	3.62E-02	1.23	0.16	5.24E-02	1.33	0.24	2.25E-02	0.46	0.10	1.55E-01	0.52	0.03	4.48E-02
CDKN2C	No	No	No	Up	1.78	1.83E-19	validated	0.48	0.10	6.03E-02	0.16	0.04	2.95E-01	0.66	0.11	1.61E-02	1.18	0.13	1.01E-03	1.08	0.15	4.35E-03	0.93	0.22	6.43E-02	1.71	0.11	1.14E-06	0.43	0.03	1.57E-02
ICE1	No	Yes	No	Up	1.78	0.000593	0.0003	1.13	0.11	6.18E-03	0.82	0.04	2.48E-03	1.05	0.11	1.41E-02	0.56	0.12	2.22E-01	0.66	0.14	3.14E-01	0.92	0.22	1.88E-01	0.26	0.10	4.10E-01	1.23	0.04	1.54E-03
GAS1	No	No	No	Up	1.78	1.83E-19	0.0125	1.35	0.12	6.36E-06	0.07	0.04	5.76E-01	0.64	0.11	7.38E-03	1.35	0.14	2.00E-05	1.03	0.15	7.12E-04	0.88	0.22	8.14E-03	0.59	0.10	1.09E-02	0.73	0.03	5.45E-06
CHERP	No	Yes	No	Up	1.78	0.000391	0.00053	1.46	0.12	2.06E-03	0.51	0.04	2.11E-02	1.08	0.11	9.88E-03	0.78	0.12	5.51E-02	1.05	0.15	3.49E-02	0.24	0.20	4.57E-01	1.40	0.11	4.89E-03	0.12	0.03	4.38E-01
SLC6A13	No	Yes	No	Up	1.78	1.83E-19	1.5E-19	1.69	0.13	2.18E-05	1.34	0.04	1.00E-20	0.74	0.11	4.40E-03	0.45	0.12	1.10E-01	0.01	0.14	9.74E-01	0.19	0.20	7.29E-01	1.30	0.11	4.77E-06	0.91	0.03	2.04E-06
PHC3	No	Yes	No	Up	1.78	8.51E-05	0.00383	0.85	0.11	3.40E-02	0.78	0.04	2.79E-03	0.89	0.11	6.38E-03	0.71	0.12	3.01E-02	0.79	0.15	2.25E-01	1.02	0.23	1.25E-01	1.21	0.10	3.60E-03	0.40	0.03	2.03E-02
FZD7	No	Yes	No	Up	1.78	1.83E-19	0.00023	0.64	0.11	7.67E-03	0.75	0.04	2.18E-05	0.78	0.11	1.75E-03	1.54	0.14	7.50E-06	0.98	0.15	8.85E-03	0.86	0.22	1.95E-02	0.39	0.10	6.38E-02	0.70	0.03	9.68E-05
PBX2	No	Yes	No	Up	1.78	9.89E-06	8.9E-06	1.46	0.12	2.14E-04	0.77	0.04	8.56E-04	0.68	0.11	9.35E-02	0.88	0.12	2.74E-02	0.16	0.14	4.59E-01	0.60	0.21	1.10E-01	1.67	0.11	1.67E-04	0.45	0.03	5.99E-02
CYTL1	No	No	No	Up	1.78	1.83E-19	0.00024	0.46	0.10	4.29E-02	0.76	0.04	7.29E-05	0.91	0.11	1.54E-03	1.66	0.15	9.09E-07	0.84	0.15	8.70E-03	1.04	0.23	6.59E-03	0.03	0.10	9.19E-01	0.96	0.03	1.00E-20
C20ORF27	No	Yes	Yes	Up	1.78	5.41E-05	0.00011	1.13	0.11	2.06E-03	0.38	0.04	4.60E-02	1.70	0.13	6.05E-04	1.29	0.14	2.84E-03	0.24	0.14	3.11E-01	1.31	0.24	9.87E-03	0.29	0.10	4.27E-01	0.32	0.03	8.28E-02
PPT1	No	Yes	No	Up	1.78	2.19E-05	0.00123	1.44	0.12	2.49E-04	0.19	0.04	2.10E-01	1.37	0.12	4.15E-03	1.44	0.14	1.80E-03	0.76	0.15	9.10E-03	0.75	0.21	2.63E-02	0.16	0.10	4.79E-01	0.54	0.03	6.81E-02
C16ORF5	No	No	No	Up	1.78	6.71E-06	0.04148	1.23	0.12	9.50E-04	0.58	0.04	6.19E-03	0.87	0.11	1.61E-02	1.89	0.16	1.23E-05	0.81	0.15	9.54E-02	0.26	0.20	4.85E-01	0.58	0.10	5.68E-02	0.44	0.03	3.14E-02
CPT1C	No	No	No	Up	1.78	1.83E-19	0.03326	1.31	0.12	1.52E-05	1.04	0.04	1.36E-06	0.28	0.10	3.00E-01	1.05	0.13	8.35E-04	0.92	0.15	1.46E-02	1.00	0.23	2.39E-02	0.27	0.10	3.60E-01	0.80	0.03	1.77E-05
RBM11	No	Yes	No	Up	1.78	5.03E-05	1.5E-19	0.86	0.11	2.94E-03	0.72	0.04	1.24E-03	1.38	0.12	9.42E-04	0.15	0.11	5.35E-01	0.65	0.14	4.99E-01	1.04	0.23	3.19E-01	1.37	0.11	6.43E-03	0.51	0.03	8.83E-03
CUL7	No	Yes	No	Up	1.79	0.00021	0.00423	1.51	0.12	2.62E-04	0.99	0.04	6.64E-04	0.53	0.11	1.93E-01	0.20	0.11	5.92E-01	0.27	0.14	6.82E-01	1.07	0.23	2.23E-01	1.86	0.11	5.71E-04	0.27	0.03	1.45E-01
PEPD	No	Yes	No	Up	1.79	1.14E-05	2.6E-05	1.36	0.12	1.05E-03	0.43	0.04	3.66E-02	0.55	0.11	1.40E-01	1.16	0.13	1.35E-02	0.93	0.15	3.40E-03	1.33	0.24	9.02E-04	0.21	0.10	4.50E-01	0.74	0.03	6.94E-03
SRCRB4D	No	Yes	No	Up	1.79	1.83E-19	1.5E-19	1.76	0.13	6.82E-07	1.35	0.04	1.00E-20	0.41	0.10	2.57E-01	0.55	0.12	8.45E-02	0.40	0.14	6.69E-01	0.93	0.22	5.98E-01	0.88	0.10	4.82E-02	0.43	0.03	2.43E-02
ALK	No	Yes	No	Up	1.79	1.14E-05	1.5E-19	1.20	0.11	6.09E-05	0.84	0.04	3.03E-04	1.33	0.12	2.78E-03	1.05	0.13	4.25E-04	1.03	0.15	4.20E-01	0.89	0.22	3.44E-01	0.29	0.10	2.13E-01	0.08	0.03	6.16E-01
NCAPD2	No	Yes	No	Up	1.79	1.83E-19	0.00018	1.09	0.11	5.62E-03	0.91	0.04	1.32E-04	1.06	0.11	1.75E-03	0.65	0.12	9.65E-02	0.23	0.14	3.55E-01	1.20	0.24	5.51E-03	0.79	0.10	8.04E-03	0.79	0.03	3.16E-03
CACNA2D2	No	Yes	No	Up	1.79	1.83E-19	1.5E-19	1.05	0.11	1.26E-03	0.71	0.04	9.38E-05	0.77	0.11	7.33E-03	0.71	0.12	1.09E-02	0.64	0.14	8.65E-02	1.72	0.27	1.63E-02	0.60	0.10	2.98E-02	0.52	0.03	3.34E-03
UBE2E2	No	Yes	No	Up	1.79	1.83E-19	3.3E-05	1.48	0.12	1.20E-05	0.70	0.04	4.16E-04	0.87	0.11	3.09E-03	0.49	0.12	1.03E-01	0.93	0.15	5.42E-03	0.59	0.21	6.57E-02	1.08	0.10	2.30E-04	0.57	0.03	9.16E-03
NFYA	No	No	No	Up	1.79	1.14E-05	validated	1.11	0.11	1.74E-03	0.86	0.04	7.59E-05	0.89	0.11	4.17E-03	1.13	0.13	1.40E-02	0.90	0.15	6.85E-02	1.15	0.23	4.38E-02	0.50	0.10	1.09E-01	0.17	0.03	2.71E-01
NUP153	No	Yes	No	Up	1.79	6.71E-06	0.00111	1.20	0.11	3.61E-03	0.92	0.04	1.12E-03	0.74	0.11	1.69E-02	0.38	0.11	3.59E-01	0.67	0.14	5.75E-02	1.29	0.24	3.50E-03	1.02	0.10	4.06E-03	0.50	0.03	9.57E-03
CPEB1	No	Yes	No	Up	1.79	1.14E-05	1.5E-19	0.61	0.10	3.29E-02	0.61	0.04	1.77E-03	0.68	0.11	4.75E-02	0.65	0.12	1.71E-02	1.07	0.15	1.68E-01	1.12	0.23	2.40E-01	1.23	0.10	5.41E-03	0.74	0.03	7.68E-05
GLI3	No	No	No	Up	1.79	1.83E-19	0.00077	1.06	0.11	1.64E-04	0.48	0.04	3.14E-03	0.55	0.11	4.71E-02	1.36	0.14	3.48E-05	1.32	0.16	2.36E-03	1.21	0.24	8.51E-03	0.16	0.10	4.69E-01	0.60	0.03	3.41E-04
CYP26B1	No	No	No	Up	1.79	1.83E-19	0.00752	0.64	0.11	1.92E-02	0.63	0.04	2.99E-04	0.78	0.11	1.16E-02	1.51	0.14	1.05E-05	1.22	0.16	7.55E-04	0.86	0.22	1.76E-02	0.34	0.10	1.57E-01	0.76	0.03	5.45E-06
LEPREL1	No	Yes	No	Up	1.79	1.83E-19	1.5E-19	1.45	0.12	1.00E-05	0.40	0.04	1.29E-02	1.18	0.11	1.87E-04	0.86	0.12	4.60E-03	0.69	0.14	1.30E-02	0.20	0.20	3.73E-01	1.35	0.11	6.27E-05	0.62	0.03	2.19E-04
CD40	No	Yes	No	Up	1.80	7.95E-05	1.5E-19	1.12	0.11	4.08E-04	0.36	0.04	3.84E-02	0.54	0.11	7.00E-02	0.24	0.11	4.23E-01	2.41	0.22	1.10E-03	1.32	0.24	1.32E-02	0.38	0.10	2.42E-01	0.38	0.03	2.06E-02
C1ORF38	No	Yes	No	Up	1.80	1.83E-19	1.5E-19	2.44	0.16	1.00E-20	0.56	0.04	2.16E-03	0.61	0.11	2.30E-02	0.33	0.11	2.99E-01	0.87	0.15	4.98E-01	0.77	0.21	4.97E-01	0.55	0.10	1.23E-01	0.63	0.03	5.29E-03
FAM89A	No	No	No	Up	1.80	1.83E-19	0.00783	0.35	0.10	2.14E-01	0.72	0.04	2.79E-04	0.60	0.11	3.67E-02	0.62	0.12	4.31E-02	1.58	0.17	1.54E-04	0.82	0.22	3.84E-02	1.50	0.11	1.79E-05	0.59	0.03	1.54E-02
ART5	No	No	No	Up	1.80	0.000177	4.1E-05	0.68	0.11	9.99E-03	0.41	0.04	2.22E-02	0.39	0.10	3.51E-01	1.75	0.15	7.50E-06	1.49	0.17	8.85E-02	1.33	0.24	1.28E-01	0.36	0.10	4.48E-01	0.35	0.03	6.40E-02
AGO2	No	No	No	Up	1.80	1.83E-19	0.00475	1.53	0.12	1.07E-04	1.01	0.04	8.72E-05	1.23	0.11	7.56E-04	0.54	0.12	1.91E-01	0.72	0.14	1.27E-02	0.36	0.20	1.87E-01	0.91	0.10	4.60E-03	0.48	0.03	5.91E-02
SV2A	No	No	No	Up	1.80	0.000177	0.03622	0.40	0.10	2.09E-01	0.67	0.04	1.61E-02	0.57	0.11	4.42E-02	0.82	0.12	6.57E-03	1.23	0.16	7.53E-02	1.91	0.29	9.67E-02	0.60	0.10	3.27E-02	0.59	0.03	8.12E-04
KCTD2	No	Yes	No	Up	1.80	0.000258	0.00317	1.05	0.11	1.48E-02	0.73	0.04	1.29E-02	0.67	0.11	1.91E-01	1.14	0.13	3.34E-03	1.32	0.16	9.31E-03	1.04	0.23	6.14E-02	0.34	0.10	4.12E-01	0.48	0.03	1.29E-02
SLMO1	No	Yes	Yes	Up	1.80	1.83E-19	1.5E-19	0.82	0.11	3.48E-03	1.20	0.04	2.27E-07	1.16	0.11	4.70E-03	0.63	0.12	2.68E-02	0.73	0.14	1.13E-01	1.23	0.24	4.20E-02	0.48	0.10	1.62E-01	0.54	0.03	2.09E-03
METRNL	No	Yes	No	Up	1.80	1.83E-19	1.5E-19	0.93	0.11	1.32E-03	0.82	0.04	9.72E-05	1.00	0.11	2.49E-03	0.72	0.12	3.20E-02	1.25	0.16	8.77E-04	1.05	0.23	7.03E-03	0.22	0.10	4.01E-01	0.82	0.03	6.28E-04
FN1	No	No	No	Up	1.80	1.83E-19	validated	0.96	0.11	2.09E-03	0.39	0.04	8.89E-03	0.32	0.10	1.99E-01	1.96	0.16	2.27E-07	0.82	0.15	3.08E-01	1.29	0.24	1.88E-01	0.30	0.10	1.86E-01	0.76	0.03	5.23E-05
MRAS	No	No	No	Up	1.80	3.58E-06	0.00563	0.94	0.11	2.65E-03	0.60	0.04	1.61E-03	0.73	0.11	2.20E-02	0.85	0.12	7.28E-03	1.52	0.17	3.56E-02	0.84	0.22	1.85E-01	0.56	0.10	6.78E-02	0.76	0.03	5.59E-05
CCNYL1	No	Yes	No	Up	1.80	1.83E-19	0.00028	1.59	0.12	1.43E-05	0.90	0.04	1.01E-04	0.68	0.11	2.41E-02	0.89	0.12	1.72E-02	0.76	0.15	1.03E-01	0.80	0.22	1.81E-01	0.81	0.10	6.37E-03	0.38	0.03	1.18E-01
C7ORF10	No	Yes	No	Up	1.80	1.83E-19	1.5E-19	0.88	0.11	2.37E-02	0.52	0.04	2.53E-02	0.88	0.11	5.77E-03	0.61	0.12	4.74E-02	0.91	0.15	2.35E-02	0.72	0.21	7.16E-02	1.55	0.11	4.10E-04	0.73	0.03	3.04E-05
BEX2	No	Yes	No	Up	1.80	1.83E-19	1.5E-19	0.76	0.11	2.89E-03	0.77	0.04	1.05E-05	1.20	0.11	2.57E-05	0.75	0.12	3.73E-03	0.99	0.15	2.35E-03	1.36	0.25	1.03E-03	0.41	0.10	5.76E-02	0.58	0.03	1.25E-03
C9ORF66	No	Yes	No	Up	1.81	6.46E-05	1.5E-19	0.38	0.10	1.83E-01	0.11	0.04	6.68E-01	1.13	0.11	7.97E-04	1.24	0.13	1.48E-03	0.54	0.14	3.32E-01	1.31	0.24	1.03E-01	1.73	0.11	2.76E-04	0.37	0.03	1.85E-02
DLX4	No	Yes	No	Up	1.81	3.58E-06	1.5E-19	1.19	0.11	2.15E-04	0.77	0.04	1.62E-04	1.04	0.11	1.78E-03	0.91	0.12	2.44E-03	0.59	0.14	4.93E-01	1.08	0.23	2.92E-01	1.02	0.10	1.51E-02	0.21	0.03	1.84E-01
CTDSP2	No	Yes	No	Up	1.81	3.58E-06	0.0001	1.48	0.12	4.26E-04	0.87	0.04	2.14E-04	0.86	0.11	2.08E-02	0.74	0.12	4.99E-02	0.70	0.14	1.62E-02	1.01	0.23	9.17E-03	0.07	0.10	7.99E-01	1.10	0.04	1.46E-03
CD22	No	Yes	No	Up	1.81	0.000103	1.5E-19	1.26	0.12	3.10E-04	0.46	0.04	4.60E-02	0.78	0.11	6.36E-02	0.74	0.12	1.46E-02	0.66	0.14	4.82E-01	1.31	0.24	2.53E-01	1.09	0.10	2.76E-02	0.53	0.03	1.37E-03
ELOVL2	No	Yes	No	Up	1.81	0.000744	1.5E-19	0.28	0.10	3.25E-01	0.78	0.04	3.94E-02	0.98	0.11	6.04E-03	1.03	0.13	4.39E-03	0.83	0.15	4.35E-01	1.38	0.25	3.03E-01	1.07	0.10	1.06E-02	0.47	0.03	6.73E-03
GLI2	No	No	No	Up	1.81	3.58E-06	validated	0.15	0.10	5.69E-01	0.69	0.04	2.62E-04	0.98	0.11	3.19E-03	1.56	0.15	7.72E-06	1.30	0.16	1.66E-02	1.28	0.24	1.24E-02	0.71	0.10	1.01E-01	0.16	0.03	2.73E-01
LRFN5	No	No	No	Up	1.81	1.83E-19	0.00028	1.64	0.13	5.18E-05	0.66	0.04	2.61E-03	0.78	0.11	4.32E-03	0.43	0.12	1.40E-01	1.00	0.15	4.79E-02	1.10	0.23	4.43E-02	0.63	0.10	2.39E-02	0.59	0.03	1.61E-03
KCNA5	No	No	No	Up	1.81	4.80E-05	0.0293	1.17	0.11	3.71E-04	0.17	0.04	3.13E-01	0.44	0.10	3.09E-01	1.00	0.13	1.84E-03	1.13	0.16	2.33E-02	1.81	0.28	1.56E-02	0.66	0.10	1.17E-01	0.46	0.03	4.59E-03

Supplementary Table 2. Details for 2,053 genes differentially expressed between EEC and NEEC, identified by microarray meta-analysis.

EEC - endometrioid endometrial cancer, NEEC - non-endometrioid endometrial cancer, SMD - Standardised Mean Difference, FDR - False Discovery Rate, Var - Variance

Gene	145 gene list	1253 gene list	Associated with EEC-specific survival?	Up/Down Regulated in NEEC	average standardised fold change	Microarray Meta FDR	RNASeq FDR	Individual microarray study results																								
								TCGA			E-MTAB-2532			E-GEOD-56026			GSE32507			GSE24537			E-GEOD-23518			E-GEOD-17025			E-GEOD-2109		
								SMD	Var	P-value	SMD	Var	P-value	SMD	Var	P-value	SMD	Var	P-value	SMD	Var	P-value	SMD	Var	P-value	SMD	Var	P-value	SMD	Var	P-value
STX1A	No	No	No	Up	1.81	1.14E-05	0.00308	1.25	0.12	5.14E-03	1.04	0.04	5.68E-06	0.77	0.11	9.15E-02	0.02	0.11	9.37E-01	1.18	0.16	4.42E-02	0.92	0.22	1.20E-01	1.16	0.10	9.26E-03	0.50	0.03	1.28E-02
PFKM	No	Yes	Yes	Up	1.81	1.83E-19	8.9E-05	0.56	0.10	3.86E-02	0.93	0.04	2.41E-05	0.74	0.11	1.20E-02	1.23	0.13	2.36E-03	0.94	0.15	1.45E-02	1.50	0.26	2.64E-03	0.08	0.10	7.14E-01	0.90	0.03	4.15E-04
UST	No	Yes	No	Up	1.82	1.83E-19	1.5E-19	0.98	0.11	6.78E-04	0.46	0.04	1.60E-02	1.35	0.12	4.20E-05	1.06	0.13	6.15E-04	0.93	0.15	2.53E-02	0.67	0.21	9.05E-02	1.02	0.10	3.54E-03	0.41	0.03	2.99E-02
NUP155	No	Yes	No	Up	1.82	1.83E-19	0.00158	0.97	0.11	6.79E-03	0.88	0.04	1.54E-04	0.72	0.11	3.39E-02	1.32	0.14	2.27E-03	0.99	0.15	8.53E-03	0.80	0.22	3.75E-02	0.24	0.10	3.02E-01	0.96	0.03	2.95E-04
CAMTA1	No	Yes	No	Up	1.82	1.83E-19	0.00347	1.02	0.11	3.98E-03	1.21	0.04	4.54E-06	1.50	0.12	6.82E-06	0.84	0.12	2.57E-03	0.91	0.15	7.33E-02	0.50	0.21	2.47E-01	0.08	0.10	7.34E-01	0.83	0.03	5.45E-06
PTBP2	No	Yes	No	Up	1.82	1.83E-19	0.00019	0.75	0.11	1.65E-02	0.92	0.04	4.02E-05	1.59	0.12	7.00E-05	0.50	0.12	2.37E-01	0.67	0.14	3.54E-02	0.99	0.22	1.50E-02	0.95	0.10	8.18E-03	0.52	0.03	1.44E-02
RNF144A	No	Yes	No	Up	1.82	1.83E-19	7.4E-05	1.00	0.11	6.44E-03	0.35	0.04	6.60E-02	0.90	0.11	4.35E-03	0.97	0.13	7.27E-03	1.53	0.17	1.81E-04	0.89	0.22	1.36E-02	0.88	0.10	1.14E-02	0.38	0.03	2.30E-02
TTK	No	Yes	Yes	Up	1.82	1.83E-19	1.5E-19	1.19	0.11	2.80E-04	0.89	0.04	2.54E-05	0.41	0.10	8.72E-02	0.92	0.12	3.43E-03	0.63	0.14	4.72E-02	1.07	0.23	1.69E-02	0.99	0.10	5.05E-04	0.81	0.03	9.02E-05
RTN4R	No	Yes	No	Up	1.82	0.000331	0.0005	0.85	0.11	4.79E-03	0.27	0.04	1.18E-01	1.23	0.11	2.55E-03	1.14	0.13	2.59E-03	0.58	0.14	2.28E-01	1.56	0.26	5.89E-02	1.11	0.10	3.29E-02	0.18	0.03	3.46E-01
UBE2C	No	Yes	No	Up	1.82	1.83E-19	1.5E-19	1.03	0.11	1.36E-03	1.11	0.04	4.54E-07	0.72	0.11	7.79E-03	0.59	0.12	5.89E-02	0.61	0.14	1.97E-02	1.29	0.24	7.46E-04	0.80	0.10	7.46E-03	0.78	0.03	2.05E-04
E2F6	No	No	No	Up	1.82	4.80E-05	0.01552	1.14	0.11	2.35E-03	0.27	0.04	1.42E-01	1.02	0.11	1.25E-02	1.21	0.13	5.22E-03	0.97	0.15	2.64E-02	0.98	0.22	1.28E-02	0.44	0.10	1.63E-01	0.91	0.03	1.01E-02
CDON	No	Yes	No	Up	1.83	1.83E-19	4.1E-05	1.09	0.11	1.16E-03	0.80	0.04	1.39E-04	0.64	0.11	1.87E-02	1.99	0.17	4.54E-07	0.48	0.14	5.25E-01	0.52	0.21	7.19E-01	0.72	0.10	1.13E-02	0.72	0.03	2.79E-05
DEGS1	No	Yes	No	Up	1.83	1.83E-19	1.5E-19	1.43	0.12	1.39E-04	0.38	0.04	2.20E-02	0.93	0.11	4.22E-03	1.02	0.13	5.39E-03	0.80	0.15	4.16E-03	0.48	0.21	1.06E-01	1.17	0.10	3.14E-04	0.75	0.03	9.56E-04
C19ORF40	No	Yes	Yes	Up	1.83	0.00016	1.5E-19	0.84	0.11	1.24E-02	0.65	0.04	2.79E-02	0.68	0.11	9.01E-02	1.46	0.14	2.93E-04	0.44	0.14	3.62E-01	1.34	0.24	7.07E-02	1.01	0.10	1.11E-02	0.54	0.03	5.38E-02
COL6A6	No	No	No	Up	1.83	1.83E-19	0.0003	1.28	0.12	1.57E-04	0.28	0.04	9.75E-02	0.35	0.10	1.47E-01	1.86	0.16	2.27E-07	0.58	0.14	3.45E-01	0.96	0.22	3.90E-01	0.94	0.10	3.25E-02	0.71	0.03	3.41E-05
GABRE	No	Yes	No	Up	1.83	1.83E-19	1.5E-19	1.83	0.13	6.82E-07	0.84	0.04	5.45E-06	0.94	0.11	2.05E-03	0.79	0.12	6.32E-03	0.52	0.14	2.73E-01	0.17	0.20	6.98E-01	1.13	0.10	2.08E-04	0.76	0.03	4.16E-05
DMRT3	No	No	No	Up	1.83	1.83E-19	0.00013	0.56	0.10	2.86E-02	0.29	0.04	4.83E-02	1.24	0.12	6.00E-05	2.62	0.20	1.00E-20	0.08	0.14	8.62E-01	0.89	0.22	3.00E-01	0.68	0.10	2.58E-01	0.64	0.03	5.02E-05
SLC6A15	No	Yes	No	Up	1.84	1.83E-19	1.5E-19	0.98	0.11	2.33E-03	1.00	0.04	5.43E-04	0.97	0.11	4.94E-03	0.72	0.12	1.74E-02	0.81	0.15	3.74E-01	1.13	0.23	3.13E-01	0.47	0.10	1.82E-01	0.93	0.03	1.00E-20
C7ORF13	No	Yes	No	Up	1.84	1.83E-19	1.5E-19	0.76	0.11	4.35E-03	1.16	0.04	1.20E-05	0.81	0.11	2.76E-02	1.34	0.14	7.77E-05	0.38	0.14	3.70E-01	0.81	0.22	1.31E-01	0.74	0.10	5.42E-02	1.05	0.04	3.86E-06
ARHGAP28	No	Yes	No	Up	1.84	1.83E-19	0.00015	1.04	0.11	2.15E-03	1.24	0.04	4.54E-07	0.89	0.11	1.80E-03	1.42	0.14	2.79E-05	1.03	0.15	6.60E-02	0.63	0.21	1.91E-01	0.42	0.10	1.26E-01	0.40	0.03	9.41E-03
TIMP2	No	Yes	No	Up	1.85	1.83E-19	0.00529	1.15	0.11	1.26E-04	1.27	0.04	4.54E-07	0.41	0.10	1.04E-01	0.68	0.12	2.08E-02	1.18	0.16	2.17E-04	1.08	0.23	2.15E-03	0.64	0.10	1.60E-02	0.66	0.03	5.89E-04
FXYD6	No	Yes	No	Up	1.85	1.83E-19	1.5E-19	1.55	0.12	5.91E-06	0.32	0.04	2.80E-02	1.12	0.11	2.76E-04	0.93	0.12	2.69E-03	0.66	0.14	1.71E-02	1.01	0.23	6.27E-03	0.56	0.10	3.20E-02	0.93	0.03	3.41E-06
ZBTB46	No	Yes	No	Up	1.85	1.83E-19	1.5E-19	0.78	0.11	3.14E-02	0.45	0.04	1.32E-02	1.59	0.12	2.16E-05	1.10	0.13	2.01E-03	1.22	0.16	4.07E-03	0.91	0.22	2.81E-02	0.77	0.10	6.58E-03	0.29	0.03	8.95E-02
RCBTB2	No	Yes	No	Up	1.85	3.58E-06	0.00028	0.95	0.11	7.85E-03	0.61	0.04	6.93E-03	1.17	0.11	7.67E-04	0.62	0.12	7.00E-02	1.19	0.16	7.13E-03	1.33	0.24	1.08E-02	1.03	0.10	1.50E-03	0.21	0.03	1.77E-01
RNF212	No	Yes	No	Up	1.85	1.83E-19	1.5E-19	1.08	0.11	4.96E-04	1.00	0.04	1.24E-04	0.39	0.10	2.82E-01	0.40	0.12	1.20E-01	1.05	0.15	4.38E-02	1.45	0.25	7.54E-03	0.86	0.10	7.41E-02	0.87	0.03	3.63E-06
CCDC90A	No	No	No	Up	1.85	3.58E-06	0.0074	1.48	0.12	3.62E-04	0.63	0.04	5.53E-03	0.60	0.11	5.80E-02	1.02	0.13	2.40E-02	1.01	0.15	9.05E-03	0.76	0.21	4.85E-02	0.82	0.10	4.75E-03	0.78	0.03	1.32E-02
MAGEL2	No	Yes	Yes	Up	1.85	1.83E-19	1.5E-19	0.97	0.11	3.66E-04	0.82	0.04	1.41E-05	0.95	0.11	5.31E-03	0.74	0.12	5.47E-03	0.96	0.15	8.34E-02	0.72	0.21	1.80E-01	0.96	0.10	1.06E-02	0.98	0.03	1.00E-20
HAPLN4	No	Yes	No	Up	1.85	1.83E-19	1.5E-19	0.97	0.11	6.41E-02	0.99	0.04	1.35E-04	1.16	0.11	4.14E-03	0.82	0.12	7.14E-03	0.66	0.14	2.44E-01	0.65	0.21	2.86E-01	0.95	0.10	3.94E-02	0.91	0.03	9.09E-07
BLCAP	No	Yes	No	Up	1.85	3.58E-06	0.00021	1.37	0.12	2.57E-03	0.61	0.04	1.06E-02	1.06	0.11	6.81E-03	0.86	0.12	2.70E-02	0.72	0.14	2.31E-02	0.73	0.21	5.15E-02	0.78	0.10	1.82E-02	0.99	0.03	4.27E-04
C5ORF34	No	Yes	No	Up	1.85	3.58E-06	4.6E-06	1.25	0.12	3.08E-04	0.95	0.04	2.92E-04	0.18	0.10	5.17E-01	1.35	0.14	2.33E-03	0.74	0.14	1.31E-01	1.12	0.23	5.23E-02	0.68	0.10	3.51E-02	0.86	0.03	1.34E-03
DNMT3A	No	Yes	No	Up	1.85	1.14E-05	0.00051	1.56	0.12	6.52E-05	1.16	0.04	6.36E-06	1.25	0.12	5.64E-04	0.05	0.11	8.78E-01	1.11	0.15	3.60E-01	1.54	0.26	2.78E-01	0.31	0.10	1.92E-01	0.16	0.03	3.91E-01
CCDC23	No	Yes	No	Up	1.86	1.83E-19	0.00043	0.40	0.10	2.17E-01	0.83	0.04	5.47E-04	1.06	0.11	7.94E-03	0.95	0.12	5.75E-02	1.61	0.18	8.91E-05	1.37	0.25	1.21E-03	0.41	0.10	1.36E-01	0.50	0.03	5.20E-03
KLRG2	No	Yes	Yes	Up	1.86	1.83E-19	1.5E-19	1.44	0.12	2.61E-05	1.22	0.04	6.82E-07	1.53	0.12	4.48E-05	0.55	0.12	3.28E-02	0.57	0.14	5.52E-01	0.72	0.21	3.32E-01	0.66	0.10	8.05E-02	0.47	0.03	5.81E-03
GFPT2	No	No	No	Up	1.86	1.83E-19	validated	0.50	0.10	7.18E-02	0.24	0.04	1.03E-01	0.21	0.10	4.26E-01	1.26	0.13	1.81E-04	1.92	0.19	3.58E-04	1.37	0.25	1.50E-02	0.80	0.10	7.98E-03	0.87	0.03	1.73E-05
ABCF1	No	Yes	No	Up	1.86	1.14E-05	0.01271	1.31	0.12	7.20E-03	0.73	0.04	1.94E-03	0.72	0.11	8.38E-02	1.05	0.13	3.32E-02	1.15	0.16	8.51E-04	1.10	0.23	3.20E-03	0.17	0.10	5.26E-01	0.96	0.03	1.15E-02
KIF7	No	No	No	Up	1.86	1.83E-19	0.00406	0.59	0.10	4.56E-02	0.83	0.04	3.13E-04	0.80	0.11	6.36E-02	1.39	0.14	2.87E-04	0.65	0.14	1.36E-01	1.00	0.23	3.81E-02	1.11	0.10	8.53E-03	0.81	0.03	4.45E-05
ARHGAP23	No	Yes	No	Up	1.86	1.83E-19	1.5E-19	0.89	0.11	6.96E-03	0.60	0.04	2.03E-03	0.76	0.11	3.21E-02	0.95	0.12	8.39E-03	0.92	0.15	4.16E-03	0.90	0.22	1.27E-02	1.76	0.11	1.28E-04	0.40	0.03	2.02E-02
TUB	No	Yes	No	Up	1.86	1.83E-19	1.5E-19	0.71	0.11	2.57E-02	0.49	0.04	3.98E-03	1.32	0.12	6.93E-05	1.00	0.13	8.56E-04	0.75	0.14	8.72E-02	1.86	0.29	1.11E-02	0.86	0.10	7.03E-03	0.20	0.03	2.18E-01
FNDC5	No	No	No	Up	1.87	1.83E-19	0.00269	0.69	0.11	1.73E-02	0.60	0.04	4.94E-04	0.60	0.11	5.70E-02	1.78	0.15	2.27E-07	0.93	0.15	5.25E-02	0.72	0.21	7.77E-02	0.90	0.10	4.82E-02	0.98	0.03	1.00E-20
TBCB	No	Yes	No	Up	1.87	6.71E-06	0.00656	1.37	0.12	1.20E-03	0.93	0.04	1.64E-03	0.86	0.11	1.53E-02	1.32	0.14	1.52E-02	1.06	0.15	1.15E-03	0.70	0.21	3.26E-02	0.43	0.10	1.68E-01	0.54	0.03	3.95E-02
S100A1	No	Yes	No	Up	1.87	1.83E-19	1.5E-19	1.93	0.14	2.95E-06	0.84	0.04	2.52E-05	0.45	0.10	1.18E-01	0.21	0.11	4.07E-01	1.59	0.17	1.83E-03	0.63	0.21	9.26E-02	1.11	0.10	1.24E-02	0.45	0.03	7.31E-03
FAM127A	No	Yes	No	Up	1.87	3.58E-06	0.0267	1.53	0.12	4.86E-04	0.89	0.04	1.20E-03	0.51	0.10	1.31E-01	1.23	0.13	1.96E-02	0.82	0.15	5.22E-03	1.38	0.25	5.44E-04	0.17	0.10	6.14E-01	0.68	0.03	1.55E-02
ELMO2	No	No	No	Up	1.87	9.98E-05	0.0018	1.01	0.11	3.13E-02	0.33	0.04	1.23E-01	0.98	0.11	9.93E-03	1.78	0.15	1.35E-03	1.14	0.16	4.71E-03	1.35	0.25	3.35E-03	0.53	0.10	1.30E-01	0.09	0.03	5.57E-01
TCF15	No	Yes	No	Up	1.87	4.80E-05	1.5E-19	0.31	0.10	1.81E-01	1.02	0.04	6.82E-07	0.47	0.10	2.98E-01	0.86	0.12	3.70E-03	1.14	0.16	3.52E-01	2.26	0.33	1.60E-01	0.79	0.10	5.76E-02	0.38	0.03	2.95E-02
EFNB1	No	Yes	No	Up	1.87	1.83E-19	1.5E-19	0.61	0.10	2.09E-02	0.98	0.04	5.68E-06	0.42	0.10	3.36E-01	0.66	0.12	3.58E-02	1.66	0.18	3.88E-04	1.50	0.26	3.48E-03	0.95	0.10	8.98E-02	0.46	0.03	1.80E-02
UCKL1	No	Yes	No	Up	1.87	3.58E-06	0.00471	1.07	0.11	1.86E-02	0.56	0.04	1.33E-02	0.23	0.10	5.04E-01	0.82	0.12	1.16E-01	1.59	0.17	8.52E-05	1.55	0.26	3.14E-04	1.07	0.10	1.62E-03	0.35	0.03	3.31E-02
PNOC	No	Yes	No	Up	1.87	1.83E-19	1.5E-19	2.53	0.16	1.00E-20	0.92	0.04	6.82E-07	0.89	0.11	1.36E-03	0.83	0.12	2.40E-03	0.06	0.14	7.07E-01	0.57	0.21	5.83E-02	0.80	0.10	9.78E-03	0.64	0.03	5.63E-05
LRRN1	No	Yes	No	Up	1.87	1.83E-19	1.5E-19	1.03	0.11	9.22E-05	1.32	0.04	1.00E-20	0.84	0.11	9.05E-04	1.10	0.13	7.84E-05	0.45	0.14	1.71E-01	0.68	0.21	7.18E-02	0.59	0.10	1.90E-02	1.24	0.04	1.00E-20
ESPL1	No	Yes	No	Up	1.88	1.83E-19	1.5E-19	1.58	0.12	1.09E-03	0.93	0.04	8.86E-06	0.64	0.11	2.43E-02	0.86	0.12	2.11E-02	0.95	0.15	1.05E-01	0.17	0.20	6.63E-01	1.26	0.10	1.94E-04	0.88	0.03	4.29E-05
TOPBP1	No	Yes	Yes	Up	1.88	3.58E-06	0.0002	1.13	0.11	2.29E-03	0.86	0.04	3.85E-04	0.80	0.11	3.53E-02	1.09	0.13	2.30E-02	0.37	0.14	2.96E-01	0.90	0.22	6.14E-02	1.08	0.10	3.28E-03	1.02	0.04	1.52E-03
TTYH2	No	No	No	Up	1.88	3.58E-06	0.00012	0.70	0.11	6.20E-02	0.64	0.04	7.18E-03	0.54	0.11	7.16E-02	1.57	0.15	1.07E-05	1.64	0.18	1.51E-02	1.15	0.23	2.47E-02	0.39	0.10	2.72E-01	0.65	0.03	5.20E-04
SAMD4A	No	Yes	No	Up	1.88	1.83E-19	1.3E-05	1.23	0.12	7.14E-04	1.01	0.04	1.66E-05	0.84	0.11	3.52E-03	1.00	0.13	1.87E-03	1.62	0.18	3.59E-02	0.88	0.22	9.38E-02	0.19	0.10	4.64E-01	0.51	0.03	3.95E-03
CHN1	No	No	No	Up	1.88	1.83E-19	0.04038	0.80	0.11	1.16E-02	1.03	0.04	4.54E-06	0.76	0.11	9.98E-03	1.24	0.13	1.30E-03	0.59	0.14	4.44E-02	1.40	0.25	1.41E-03	0.58	0.10	3.54E-02	0.90	0.03	7.72E-05
ZNF519	No	Yes	No	Up	1.88	1.14E-05	1.5E-19	0.81	0.11	1.09E-02	0.54	0.04	2.59E-02	0.88	0.11	9.37E-03	0.94	0.12	8.78E-03	0.50	0.14	1.73E-01	1.96	0.30	1.45E-02	0.99	0.10	1.93E-03	0.68	0.03	2.60E-03
EFS	No	No	No	Up	1.89	1.83E-19	0.00175	0.89	0.11	6.91E-04	0.63	0.04	1.89E-04	0.96	0.11	6.15E-04	0.99	0.13	8.75E-04	1.34	0.16	1.91E-03	1.31	0.24	2.79E-03	0.63	0.10	2.07E-02	0.57	0.03	9.53E-04
COL9A3	No	Yes	No	Up	1.89	1.83E-19	1.5E-19	0.72	0.11	3.01E-03	0.89	0.04	2.27E-06	1.82	0.13	1.00E-20	0.90	0.12	9.79E-04	0.92	0.15	4.22E-01	0.82	0.22	1.64E-01	0.50	0.10	4.86E-02	0.73	0.03	3.86E-06
LRRN2	No	Yes	No	Up	1.89	1.83E-19	1.5E-19	1.36	0.12	3.63E-06	0.75	0.04	2.59E-05	1.13	0.11	9.20E-04	0.26	0.11	3.24E-01	1.17	0.16	3.27E-04	0.80	0.22	1.40E-02	1.27	0.10	9.31E-04	0.59	0.03	4.98E-04
WNT3	No	No	Yes	Up	1.89	1.83E-19	2E-05	0.43	0.10	1.05E-01	0.95	0.04	2.66E-05	0.64	0.11	4.84E-02	1.37	0.14	3.57E-05	0.91	0.15	3.36E-01	1.28	0.24	1.95E-01	1.07	0.10	2.26E-03	0.67	0.03	8.70E-05
DCHS1	No	No	No	Up	1.89	1.83E-19	validated	0.38	0.10	1.56E-01	0.60	0.04	1.23E-03	0.84	0.11	3.46E-03	1.82	0.16	7.50E-06	1.24	0.16	2.43E-03	1.17	0.23	2.19E-02	0.83	0.10	5.48E-03	0.47	0.03	6.57E-03
LRCH2	No	Yes	No	Up	1.89	1.83E-19	1.5E-19	2.41	0.16	1.00E-20	0.58	0.04	2.07E-03	0.80	0.11	3.33E-03	0.45	0.12	5.87E-02	1.03	0.15	7.69E-03	0.72	0.21	6.15E-02	0.79	0.10	4.57E-03	0.57	0.03	1.72E-03
PCOLCE2	No	No	No	Up	1.89	1.83E-19	0.0032	0.62	0.10	1.10E-02	0.83	0.04	1.20E-05	0.99	0.11	1.39E-03	1.12	0.13	1.41E-04	1.19	0.16	5.42E-04	0.95	0.22	1.12E-02	0.71	0.10	1.59E-02	0.94	0.03	2.04E-06
TMEM121	No	Yes	No	Up	1.89	0.000136	6E-05	0.67	0.11	1.03E-02	0.30	0.04	6.69E-02	1.25	0.12	3.54E-03	1.24	0.13	8.13E-04	0.91	0.15	4.15E-01	1.10	0.23	2.84E-01	1.54	0.11	1.28E-02	0.34	0.03	3.46E-02
C5ORF25	No	Yes	No	Up	1.89	3.58E-06	2E-05	0.29	0.10	2.58E-01	0.73	0.04	9.43E-04	0.68	0.11	3.31E-02	1.09	0.13	1.12E-03	1.13	0.16	2.90E-02	1.51	0.26	3.81E-02	1.18	0.10	1.22E-03	0.75	0.03	6.58E-03
SCRN1	No	Yes	No	Up	1.89	1.83E-19	1.5E-19	1.22	0.12	4.09E-04	0.89	0.04	2.59E-05	0.75	0.11	1.27E-02	0.94	0.12	9.89E-03	1.10	0.15	1.66E-03	1.21	0.24	3.50E-03	0.59	0.10	2.50E-02	0.65	0.03	3.21E-03
PRKCI	No	Yes	Yes	Up	1.89	1.83E-19	1.5E-19	1.28	0.12	6.60E-04	0.89	0.04	1.03E-04	1.07	0.11	1.35E-03	0.25	0.11	6.02E-01	0.28	0.14	4.71E-01	0.96	0.22	1.47E-01	1.96	0.12	2.50E-06	0.68	0.03	6.44E-03
IGF2BP3	No	Yes	No	Up	1.90	1.83E-19	1.5E-19	1.38	0.12	1.36E-05	0.94	0.04	6.82E-07	0.70	0.11	3.27E-03	0.58	0.12	2.48E-02	0.93	0.15	4.55E-03	1.57	0.26	3.77E-04	0.56	0.10	1.20E-02	0.72	0.03	2.39E-05
PPP2R3A	No	Yes	Yes	Up	1.90	3.58E-06	7.7E-05	1.35	0.12	7.20E-04	0.82	0.04	1.01E-03	1.06	0.11	8.77E-03	1.22	0.13	5.48E-03	0.57	0.14	2.37E-01	0.63	0.21	2.69E-01	1.15	0.10	1.19E-03	0.60	0.03	1.65E-02
CBS	No	Yes	Yes	Up	1.90	1.83E-19	1.5E-19	1.32	0.12	5.27E-05	1.13	0.04	1.00E-20	1.82	0.13	1.00E-20	0.81	0.12	4.18E-03	0.50	0.14	6.47E-02	0.28	0.20	2.99E-01	1.08	0.10	6.61E-04	0.46	0.03	3.71E-03
ADCY9	No	Yes	No	Up	1.90	1.83E-19	1.5E-19	1.57	0.12	3.20E-05	0.75	0.04	1.04E-04	0.63	0.11	1.50E-02	0.66	0.12	2.04E-02	1.05	0.15	5.08E-02	0.84	0.22	1.16E-01	1.29	0.11	2.25E-05	0.61	0.03	1.15E-03

Supplementary Table 2. Details for 2,053 genes differentially expressed between EEC and NEEC, identified by microarray meta-analysis.

EEC - endometrioid endometrial cancer, NEEC - non-endometrioid endometrial cancer, SMD - Standardised Mean Difference, FDR - False Discovery Rate, Var - Variance

Gene	145 gene list	1253 gene list	Associated with EEC-specific survival?	Up/Down Regulated in NEEC	average standardised fold change	Microarray Meta FDR	RNASeq FDR	Individual microarray study results: TCGA			E-MTAB-2532			E-GEOD-56026			GSE32507			GSE24537			E-GEOD-23518			E-GEOD-17025			E-GEOD-2109		
								SMD	Var	P-value	SMD	Var	P-value	SMD	Var	P-value	SMD	Var	P-value	SMD	Var	P-value	SMD	Var	P-value	SMD	Var	P-value	SMD	Var	P-value
PXDN	No	Yes	No	Up	1.90	1.83E-19	2.6E-05	0.88	0.11	2.84E-03	1.08	0.04	4.54E-07	0.92	0.11	9.22E-04	1.02	0.13	8.83E-04	1.04	0.15	3.46E-03	0.69	0.21	5.16E-02	0.67	0.10	1.08E-02	1.10	0.04	6.82E-07
HPDL	No	Yes	No	Up	1.90	1.83E-19	1.5E-19	1.33	0.12	8.27E-05	1.24	0.04	1.00E-20	0.77	0.11	3.93E-02	0.21	0.11	3.98E-01	0.41	0.14	3.99E-01	1.14	0.23	1.36E-01	1.25	0.10	3.69E-03	1.07	0.04	1.00E-20
HOXA2	No	Yes	No	Up	1.90	1.83E-19	1.5E-19	1.61	0.13	4.54E-06	0.88	0.04	1.23E-05	1.39	0.12	2.09E-04	0.21	0.11	3.64E-01	0.99	0.15	1.36E-01	0.68	0.21	3.66E-01	1.16	0.10	1.26E-03	0.50	0.03	5.26E-03
ZFP64	No	Yes	No	Up	1.90	0.000159	0.00266	1.01	0.11	6.21E-02	0.59	0.04	1.03E-02	1.09	0.11	1.18E-02	1.36	0.14	2.68E-03	1.31	0.16	1.19E-01	0.98	0.22	1.58E-01	0.55	0.10	7.38E-02	0.52	0.03	4.86E-03
LMO3	No	Yes	No	Up	1.90	1.83E-19	1.5E-19	1.33	0.12	3.41E-06	0.29	0.04	4.05E-02	1.04	0.11	2.09E-04	0.57	0.12	1.80E-02	1.19	0.16	8.30E-04	1.13	0.23	3.25E-03	1.43	0.11	3.86E-06	0.43	0.03	6.60E-03
YTHDF1	No	No	No	Up	1.90	9.89E-06	0.01919	1.51	0.12	3.75E-03	1.32	0.04	1.29E-05	0.75	0.11	7.09E-02	0.45	0.12	2.96E-01	1.32	0.16	9.56E-04	1.15	0.23	5.99E-03	0.25	0.10	4.61E-01	0.68	0.03	8.79E-02
TMTC1	No	Yes	No	Up	1.90	1.83E-19	1.5E-19	0.72	0.11	5.47E-03	0.92	0.04	5.23E-06	0.29	0.10	2.02E-01	1.07	0.13	6.21E-04	1.14	0.16	3.75E-03	1.70	0.27	7.40E-04	0.65	0.10	1.30E-02	0.95	0.03	2.27E-07
DHX35	No	No	No	Up	1.91	3.97E-05	0.00044	1.54	0.12	2.29E-04	0.73	0.04	3.74E-03	1.20	0.11	1.58E-03	0.52	0.12	3.02E-01	1.03	0.15	8.67E-02	1.52	0.26	3.54E-02	0.65	0.10	7.53E-02	0.25	0.03	1.10E-01
KCNS1	No	Yes	No	Up	1.91	1.83E-19	1.5E-19	0.97	0.11	2.04E-03	1.04	0.04	2.20E-05	0.70	0.11	6.01E-02	0.98	0.13	2.79E-03	1.31	0.16	3.26E-03	1.03	0.23	8.59E-03	0.75	0.10	4.62E-02	0.67	0.03	1.18E-03
DVL3	No	Yes	No	Up	1.91	1.14E-05	0.0045	1.83	0.13	9.09E-04	0.67	0.04	4.24E-03	0.80	0.11	3.89E-02	0.39	0.12	4.42E-01	0.71	0.14	6.13E-02	1.06	0.23	2.15E-02	1.70	0.11	7.97E-05	0.32	0.03	6.76E-02
ZNF302	No	Yes	No	Up	1.91	1.83E-19	4E-05	1.13	0.11	7.06E-04	0.55	0.04	3.17E-03	1.34	0.12	2.03E-04	1.76	0.15	2.36E-05	0.59	0.14	3.69E-02	0.94	0.22	1.19E-02	0.01	0.10	9.47E-01	1.15	0.04	1.36E-06
RBMS1	No	Yes	No	Up	1.92	1.83E-19	6E-05	1.07	0.11	1.75E-03	0.46	0.04	1.71E-02	1.08	0.11	1.34E-03	1.46	0.14	3.87E-04	1.03	0.15	1.83E-03	1.12	0.23	4.31E-03	0.80	0.10	1.04E-02	0.48	0.03	3.13E-02
TMEFF2	No	Yes	No	Up	1.92	1.83E-19	1.5E-19	1.44	0.12	4.57E-05	0.48	0.04	3.53E-03	0.36	0.10	1.04E-01	1.61	0.15	2.04E-06	0.47	0.14	4.97E-01	1.20	0.24	2.09E-01	1.51	0.11	2.04E-06	0.46	0.03	2.54E-03
PIK3CA	No	Yes	No	Up	1.92	1.83E-19	1.5E-19	1.40	0.12	3.52E-04	0.85	0.04	6.82E-04	1.30	0.12	8.11E-04	0.96	0.13	4.71E-02	0.33	0.14	6.12E-01	0.81	0.22	3.74E-01	1.54	0.11	9.77E-06	0.33	0.03	1.20E-01
RFC4	No	Yes	Yes	Up	1.92	1.83E-19	2.3E-06	1.37	0.12	1.39E-04	0.99	0.04	4.43E-05	1.03	0.11	2.89E-03	1.21	0.13	7.51E-03	0.38	0.14	1.33E-01	1.13	0.23	4.00E-03	0.35	0.10	1.76E-01	1.06	0.04	5.29E-05
SORCS2	No	Yes	No	Up	1.92	1.83E-19	1.5E-19	1.46	0.12	4.32E-06	0.72	0.04	2.88E-04	0.46	0.10	1.97E-01	0.92	0.12	1.53E-03	1.00	0.15	1.12E-01	1.29	0.24	4.54E-02	0.80	0.10	6.64E-03	0.88	0.03	1.36E-06
NMU	No	Yes	No	Up	1.92	1.83E-19	1.5E-19	0.56	0.10	2.09E-02	1.11	0.04	1.00E-20	1.15	0.11	1.52E-04	0.65	0.12	1.43E-02	1.22	0.16	1.08E-03	0.67	0.21	6.78E-02	1.39	0.11	2.34E-05	0.80	0.03	3.86E-06
FNDC4	No	No	No	Up	1.93	1.83E-19	0.01553	0.57	0.10	3.57E-02	0.88	0.04	1.06E-04	0.49	0.10	1.33E-01	1.22	0.13	2.46E-04	1.22	0.16	4.66E-02	1.11	0.23	7.62E-02	1.30	0.11	1.45E-02	0.77	0.03	4.75E-04
FANCE	No	No	No	Up	1.93	1.83E-19	0.00142	0.94	0.11	4.05E-03	0.82	0.04	2.10E-04	0.55	0.11	1.26E-01	1.39	0.14	1.59E-03	0.63	0.14	1.29E-01	1.24	0.24	2.34E-02	1.10	0.10	4.10E-03	0.88	0.03	3.58E-04
AP1M1	No	No	No	Up	1.93	1.14E-05	validated	1.42	0.12	4.56E-03	0.84	0.04	3.18E-03	0.68	0.11	3.66E-02	1.50	0.14	1.23E-02	0.61	0.14	3.86E-02	0.56	0.21	1.03E-01	1.54	0.11	1.42E-04	0.41	0.03	8.88E-02
TSPAN4	No	Yes	No	Up	1.93	1.83E-19	0.00032	1.56	0.12	8.45E-05	0.56	0.04	4.87E-03	0.68	0.11	2.27E-02	0.97	0.13	1.23E-02	1.49	0.17	1.73E-04	1.10	0.23	7.47E-03	0.78	0.10	5.84E-02	0.44	0.03	2.95E-02
IRS2	No	Yes	No	Up	1.93	1.83E-19	1.5E-19	1.15	0.11	2.13E-04	0.31	0.04	4.08E-02	1.13	0.11	1.85E-04	0.74	0.12	8.76E-03	1.19	0.16	2.08E-03	1.21	0.24	6.76E-03	1.28	0.10	1.91E-05	0.59	0.03	9.94E-04
MBOAT2	No	Yes	No	Up	1.93	1.83E-19	1.5E-19	1.10	0.11	1.68E-04	1.08	0.04	9.09E-07	1.34	0.12	2.98E-05	1.43	0.14	2.45E-05	1.03	0.15	2.10E-01	0.26	0.20	4.16E-01	0.66	0.10	1.98E-02	0.71	0.03	6.33E-04
ADA	No	Yes	Yes	Up	1.93	1.83E-19	1.5E-19	0.45	0.10	1.62E-01	1.23	0.04	4.54E-07	0.85	0.11	8.97E-03	0.72	0.12	3.61E-02	1.37	0.16	2.70E-03	0.78	0.22	5.06E-02	1.25	0.10	4.58E-04	0.94	0.03	1.14E-06
PNMA2	No	Yes	No	Up	1.94	1.83E-19	2.3E-06	0.60	0.10	8.13E-02	1.03	0.04	5.91E-06	1.32	0.12	2.57E-05	2.02	0.17	1.00E-20	0.58	0.14	7.48E-02	0.56	0.21	3.07E-01	1.34	0.11	5.43E-05	0.18	0.03	2.49E-01
FAM55C	No	Yes	No	Up	1.94	3.58E-06	0.00131	1.32	0.12	1.21E-04	0.78	0.04	6.67E-04	0.51	0.11	8.89E-02	1.50	0.14	1.07E-04	0.76	0.15	4.45E-01	1.21	0.24	4.67E-01	1.31	0.11	2.10E-03	0.24	0.03	1.38E-01
ZDBF2	No	Yes	No	Up	1.94	1.83E-19	1.5E-19	0.71	0.11	4.74E-03	0.74	0.04	1.32E-04	0.95	0.11	7.84E-04	0.61	0.12	2.03E-02	0.51	0.14	6.72E-01	1.96	0.30	4.29E-01	1.34	0.11	7.66E-05	0.83	0.03	4.50E-05
NPTX2	No	No	No	Up	1.94	1.83E-19	0.01994	1.06	0.11	8.56E-05	0.95	0.04	1.59E-06	0.47	0.10	5.13E-02	1.40	0.14	2.14E-05	0.82	0.15	4.12E-03	0.82	0.22	1.29E-02	1.16	0.10	1.54E-04	0.97	0.03	1.00E-20
MTCH1	No	Yes	No	Up	1.94	1.83E-19	0.00191	1.35	0.12	5.54E-04	0.11	0.04	4.40E-01	1.24	0.12	1.59E-02	1.49	0.14	3.24E-04	0.52	0.14	3.56E-02	1.76	0.28	2.11E-05	0.43	0.10	1.88E-01	0.74	0.03	4.26E-02
CRTAC1	No	Yes	No	Up	1.94	1.83E-19	1.5E-19	1.29	0.12	3.66E-05	0.96	0.04	8.86E-06	0.99	0.11	8.51E-03	1.33	0.14	9.54E-05	1.06	0.15	3.87E-03	0.95	0.22	8.25E-03	0.44	0.10	2.96E-01	0.65	0.03	6.07E-04
MLLT11	No	No	No	Up	1.95	1.83E-19	0.02831	0.60	0.10	1.48E-02	0.96	0.04	3.18E-06	0.79	0.11	5.79E-03	1.90	0.16	2.04E-06	0.78	0.15	6.67E-03	1.42	0.25	3.37E-04	0.31	0.10	1.72E-01	0.91	0.03	5.45E-06
ATAD2	No	Yes	No	Up	1.95	1.83E-19	4E-05	1.75	0.13	1.16E-05	0.91	0.04	1.23E-05	0.88	0.11	2.95E-03	1.12	0.13	1.93E-03	0.62	0.14	5.99E-02	0.93	0.22	2.71E-02	0.87	0.10	1.42E-03	0.61	0.03	1.68E-03
TMEFF1	No	Yes	No	Up	1.95	1.83E-19	1.5E-19	0.81	0.11	2.91E-03	1.11	0.04	5.45E-06	1.22	0.11	5.84E-05	1.60	0.15	1.27E-05	0.38	0.14	7.42E-01	0.70	0.21	6.02E-01	0.87	0.10	8.36E-04	1.02	0.04	1.36E-06
SRPX	No	Yes	No	Up	1.95	1.83E-19	6.8E-05	1.03	0.11	3.80E-04	0.61	0.04	3.44E-04	0.80	0.11	2.48E-03	1.36	0.14	2.95E-05	1.08	0.15	7.57E-04	1.27	0.24	1.61E-03	0.70	0.10	1.11E-02	0.86	0.03	2.04E-06
MAGEH1	No	Yes	No	Up	1.95	1.83E-19	1.5E-19	1.44	0.12	1.29E-05	0.45	0.04	5.24E-03	0.79	0.11	6.16E-03	0.91	0.12	4.27E-03	0.89	0.15	2.13E-01	1.81	0.28	5.54E-02	0.49	0.10	6.08E-02	0.94	0.03	2.11E-05
C19ORF12	No	No	No	Up	1.96	1.83E-19	1.5E-19	1.84	0.13	1.68E-05	0.83	0.04	1.55E-03	1.00	0.11	2.68E-03	0.96	0.13	1.47E-02	1.02	0.15	2.63E-03	1.04	0.23	4.22E-03	0.83	0.10	1.25E-02	0.23	0.03	1.42E-01
EFNB3	No	No	No	Up	1.96	1.83E-19	0.00141	0.77	0.11	5.21E-02	0.92	0.04	5.45E-06	1.03	0.11	8.40E-04	1.54	0.14	5.23E-06	0.52	0.14	7.96E-02	1.40	0.25	3.60E-03	0.94	0.10	1.91E-03	0.64	0.03	1.50E-04
DOCK3	No	Yes	No	Up	1.96	1.83E-19	1.5E-19	1.53	0.12	7.04E-06	1.52	0.04	1.00E-20	0.72	0.11	3.69E-02	0.39	0.12	1.14E-01	0.72	0.14	4.54E-01	0.52	0.21	6.98E-01	1.68	0.11	5.05E-04	0.69	0.03	1.57E-04
ASXL1	No	No	No	Up	1.96	1.83E-19	4E-05	1.81	0.13	9.47E-05	1.04	0.04	8.66E-05	0.79	0.11	1.11E-02	0.48	0.12	2.44E-01	1.10	0.15	7.35E-03	1.45	0.25	5.89E-03	0.13	0.10	5.77E-01	0.96	0.03	1.13E-03
INPP4A	No	Yes	No	Up	1.96	1.14E-05	0.00154	2.36	0.15	4.32E-06	0.67	0.04	7.21E-03	1.26	0.12	1.32E-03	0.63	0.12	1.31E-01	1.23	0.16	8.80E-02	0.91	0.22	1.74E-01	0.43	0.10	1.77E-01	0.28	0.03	1.10E-01
GPRIN2	No	Yes	No	Up	1.96	3.58E-06	1.5E-19	1.06	0.11	1.18E-03	0.81	0.04	3.00E-05	0.89	0.11	4.05E-02	0.38	0.12	1.48E-01	0.86	0.15	1.96E-01	1.58	0.26	6.75E-02	1.78	0.11	1.40E-03	0.43	0.03	8.44E-03
ARMCX1	No	Yes	No	Up	1.96	1.83E-19	1.3E-05	0.85	0.11	1.29E-03	0.50	0.04	2.61E-03	1.02	0.11	4.58E-04	0.80	0.12	5.69E-03	1.64	0.18	9.00E-05	1.75	0.28	2.65E-04	0.21	0.10	3.05E-01	1.03	0.04	9.09E-07
EHMT2	No	Yes	No	Up	1.97	1.14E-05	0.01705	1.36	0.12	1.79E-03	1.12	0.04	9.54E-06	0.97	0.11	1.43E-02	0.40	0.12	3.39E-01	1.07	0.15	6.40E-02	2.09	0.31	1.88E-02	0.55	0.10	1.00E-01	0.23	0.03	1.90E-01
PGAM2	No	Yes	No	Up	1.97	1.83E-19	1.5E-19	1.43	0.12	2.57E-05	1.15	0.04	6.82E-07	0.36	0.10	3.33E-01	0.73	0.12	9.53E-03	1.19	0.16	3.87E-02	0.78	0.22	1.27E-01	0.62	0.10	1.33E-01	1.56	0.04	1.00E-20
ARHGAP29	No	Yes	No	Up	1.97	1.83E-19	1.5E-19	2.21	0.15	4.54E-07	1.26	0.04	2.27E-07	1.61	0.12	7.50E-06	0.20	0.11	4.44E-01	0.68	0.14	6.45E-01	0.40	0.20	7.45E-01	1.35	0.11	1.00E-05	0.12	0.03	4.41E-01
ROBO3	No	Yes	No	Up	1.97	1.83E-19	1.5E-19	0.93	0.11	7.99E-03	1.33	0.04	1.00E-20	1.33	0.12	2.13E-03	0.94	0.12	2.12E-03	1.02	0.15	3.72E-02	0.69	0.21	1.43E-01	0.70	0.10	1.11E-01	0.90	0.03	1.14E-06
TOX2	No	Yes	No	Up	1.98	1.83E-19	1.5E-19	0.28	0.10	2.26E-01	0.37	0.04	1.86E-02	1.69	0.13	3.18E-06	1.00	0.13	3.11E-03	1.16	0.16	1.21E-03	1.84	0.28	1.09E-04	1.13	0.10	1.33E-03	0.42	0.03	6.22E-03
MAGED2	No	Yes	No	Up	1.98	1.83E-19	0.00036	0.96	0.11	7.79E-03	0.42	0.04	1.58E-02	0.47	0.10	1.06E-01	1.51	0.14	4.16E-04	1.54	0.17	6.01E-04	1.37	0.25	3.36E-03	0.81	0.10	1.35E-02	0.82	0.03	7.13E-04
BMP7	No	Yes	No	Up	1.98	1.83E-19	1.5E-19	1.49	0.12	4.54E-07	1.12	0.04	1.00E-20	1.75	0.13	1.00E-20	0.52	0.12	3.97E-02	0.84	0.15	1.90E-02	1.03	0.23	1.33E-02	0.28	0.10	2.54E-01	0.87	0.03	9.09E-07
DOK5	No	Yes	No	Up	1.99	1.83E-19	1.5E-19	0.99	0.11	1.31E-03	0.76	0.04	2.71E-04	0.70	0.11	1.30E-02	0.64	0.12	2.34E-02	1.15	0.16	2.33E-01	0.95	0.22	2.41E-01	1.87	0.12	2.27E-07	0.86	0.03	2.73E-06
ELOVL4	No	Yes	Yes	Up	1.99	1.83E-19	1.5E-19	0.65	0.11	2.08E-02	1.04	0.04	3.09E-05	1.28	0.12	3.73E-04	0.93	0.12	4.71E-03	1.55	0.17	2.61E-03	0.83	0.22	7.29E-02	0.71	0.10	4.16E-02	0.98	0.03	1.00E-20
TCF7L1	No	No	No	Up	1.99	1.83E-19	validated	0.75	0.11	4.27E-03	0.46	0.04	6.50E-03	1.29	0.12	9.20E-05	0.74	0.12	1.89E-02	2.07	0.20	2.39E-03	1.91	0.29	9.89E-03	0.15	0.10	4.89E-01	0.60	0.03	4.86E-03
DTNA	No	Yes	No	Up	2.00	1.83E-19	1.5E-19	1.95	0.14	1.16E-05	1.10	0.04	1.41E-05	0.72	0.11	9.14E-03	0.85	0.12	2.78E-03	1.04	0.15	4.83E-02	0.68	0.21	1.05E-01	0.91	0.10	1.01E-03	0.72	0.03	2.75E-05
ARFGAP3	Yes	Yes	No	Down	0.48	1.83E-19	1.5E-19	-1.74	0.13	1.00E-05	-0.93	0.04	3.82E-05	-1.00	0.11	4.26E-03	-0.72	0.12	4.12E-02	-1.07	0.15	7.22E-04	-1.30	0.24	9.09E-04	-0.53	0.10	5.29E-02	-1.14	0.04	1.95E-05
HSF2BP	No	Yes	No	Up	2.01	1.14E-05	1.5E-19	1.56	0.12	5.52E-05	0.85	0.04	4.08E-03	1.26	0.12	4.59E-03	0.72	0.12	3.73E-02	1.20	0.16	2.49E-01	0.81	0.22	1.57E-01	1.20	0.10	1.77E-02	0.47	0.03	2.43E-02
AGA	Yes	Yes	No	Down	0.48	1.83E-19	0.00029	-1.10	0.11	2.02E-03	-1.05	0.04	1.70E-05	-0.46	0.10	1.01E-01	-1.51	0.14	7.24E-04	-1.35	0.16	1.44E-03	-1.41	0.25	5.20E-03	-0.58	0.10	3.22E-02	-0.99	0.03	1.88E-04
GPATCH1	No	Yes	No	Up	2.03	9.89E-06	1.5E-05	1.06	0.11	4.36E-03	0.65	0.04	1.34E-02	0.89	0.11	1.35E-02	1.15	0.13	8.97E-03	0.91	0.15	5.34E-02	1.32	0.24	2.41E-02	0.97	0.10	1.90E-02	1.21	0.04	1.47E-03
AGR2	Yes	Yes	No	Down	0.48	1.83E-19	3.1E-05	-1.39	0.12	2.27E-06	-1.27	0.04	1.00E-20	-1.34	0.12	6.13E-06	-0.99	0.13	3.57E-04	-0.75	0.14	2.96E-02	-0.64	0.21	7.79E-02	-0.99	0.10	1.36E-04	-1.07	0.04	1.00E-20
PPT2	No	Yes	No	Up	2.03	1.14E-05	0.00534	0.90	0.11	9.18E-03	1.23	0.04	1.98E-05	0.42	0.10	2.98E-01	1.19	0.13	6.17E-03	1.39	0.17	2.40E-02	1.25	0.24	7.94E-02	1.27	0.10	2.07E-02	0.54	0.03	4.00E-02
ARHGAP26	Yes	Yes	Yes	Down	0.48	1.83E-19	6.7E-06	-1.11	0.11	3.02E-04	-0.91	0.04	9.31E-06	-1.64	0.12	7.04E-06	-0.99	0.13	1.20E-03	-0.78	0.15	2.74E-01	-0.67	0.21	4.37E-01	-1.69	0.11	1.14E-06	-0.70	0.03	3.54E-05
ICA1	Yes	Yes	No	Down	0.48	1.83E-19	0.00858	-1.07	0.11	1.24E-03	-1.19	0.04	4.54E-07	-0.79	0.11	1.31E-02	-2.16	0.17	2.27E-07	-0.91	0.15	5.09E-03	-1.39	0.25	2.45E-03	-0.96	0.10	7.74E-03	-0.07	0.03	6.26E-01
ERICH3	Yes	Yes	No	Down	0.48	1.83E-19	1.5E-19	-1.33	0.12	2.00E-05	-1.02	0.04	9.09E-07	-1.01	0.11	2.48E-04	-1.24	0.13	3.93E-05	-0.81	0.15	1.70E-02	-0.86	0.22	8.17E-02	-1.34	0.11	1.82E-06	-0.95	0.03	1.00E-20
CTSH	Yes	Yes	No	Down	0.48	1.83E-19	2E-05	-0.87	0.11	1.78E-03	-1.13	0.04	6.82E-07	-0.51	0.11	6.11E-02	-1.10	0.13	1.99E-03	-1.42	0.17	4.13E-05	-1.37	0.25	3.84E-04	-1.27	0.10	6.27E-05	-0.91	0.03	3.32E-05
TMEM62	Yes	Yes	No	Down	0.47	1.83E-19	3.1E-05	-0.78	0.11	1.79E-02	-1.21	0.04	1.36E-06	-1.60	0.12	2.02E-04	-1.17	0.13	1.98E-03	-1.15	0.16	1.77E-03	-1.44	0.25	1.20E-03	-1.28	0.10	6.69E-04	-0.03	0.03	8.32E-01
NME5	Yes	Yes	No	Down	0.47	1.83E-19	9.5E-05	-0.82	0.11	2.24E-03	-1.10	0.04	4.54E-07	-0.80	0.11	4.72E-03	-1.17	0.13	1.46E-03	-1.44	0.17	4.92E-04	-1.51	0.26	5.17E-03	-0.71	0.10	7.29E-03	-1.11	0.04	1.00E-20
SIL1	Yes	Yes	No	Down	0.47	1.83E-19	0.00284	-0.52	0.10	1.05E-01	-0.46	0.04	2.67E-02	-1.44	0.12	2.96E-03	-1.64	0.15	1.02E-03	-0.56	0.14	3.52E-02	-1.84	0.28	7.27E-05	-1.26	0.10	7.15E-04	-0.95	0.03	7.33E-04
CIB1	Yes	Yes	No	Down	0.47	1.83E-19	2E-05	-1.07	0.11	3.45E-03	-1.38	0.04	4.54E-07	-1.48	0.12	2.65E-04	-1.11	0.13	4.55E-03	-1.18	0.16	2.52E-04	-0.71	0.21	2.53E-02	-0.72	0.10	2.20E-02	-1.04	0.04	4.77E-05
INTS10	Yes	Yes	No	Down	0.47	1.83E-19	1.5E-19	-1.90	0.13	8.72E-05	-1.01	0.04	1.50E-04	-1.29	0.12	2.02E-03	-0.84	0.12	4.45E-02	-0.60	0.14	3.71E-02	-1.55	0.26	8.48E-04	-1.02	0.10	1.38E-03	-0.48	0.03	1.04E-02
SOX17	Yes	No	No	Down	0.47	1.83E-19	0.00482	-0.84	0.11	3.16E-03	-0.85	0.04	3.18E-06	-0.82	0.11	2.56E-03	-1.07	0.13	3.40E-04	-1.57	0.17	1.48E-05	-1.09	0.23	2.57E-03	-1.25	0.10	2.23E-05	-1.23	0.04	1.00E-20

Supplementary Table 2. Details for 2,053 genes differentially expressed between EEC and NEEC, identified by microarray meta-analysis.

EEC - endometrioid endometrial cancer, NEEC - non-endometrioid endometrial cancer, SMD - Standardised Mean Difference, FDR - False Discovery Rate, Var - Variance

Gene	145 gene list	1253 gene list	Associated with EEC-specific survival?	Up/Down Regulated in NEEC	average standardised fold change	Microarray Meta FDR	RNASeq FDR	Individual microarray study results																							
								TCGA			E-MTAB-2532			E-GEOD-56026			GSE32507			GSE24537			E-GEOD-23518			E-GEOD-17025			E-GEOD-2109		
								SMD	Var	P-value	SMD	Var	P-value	SMD	Var	P-value	SMD	Var	P-value	SMD	Var	P-value	SMD	Var	P-value	SMD	Var	P-value	SMD	Var	P-value
CHMP4A	Yes	No	No	Down	0.47	1.83E-19	0.02308	-0.85	0.11	1.68E-02	-0.55	0.04	2.31E-02	-0.41	0.10	2.04E-01	-0.07	0.11	8.84E-01	-2.18	0.21	1.02E-04	-2.72	0.38	9.29E-05	-1.17	0.10	5.25E-04	-0.79	0.03	1.29E-02
STX18	Yes	Yes	No	Down	0.47	1.83E-19	0.00026	-0.91	0.11	1.18E-03	-0.99	0.04	1.36E-06	-0.76	0.11	4.40E-03	-1.58	0.15	8.86E-05	-1.07	0.15	4.27E-03	-1.10	0.23	2.76E-02	-1.36	0.11	1.16E-05	-1.04	0.04	1.00E-20
PTPN3	Yes	Yes	No	Down	0.47	1.83E-19	2.6E-05	-0.88	0.11	7.97E-03	-1.13	0.04	1.36E-06	-0.95	0.11	2.72E-03	-1.66	0.15	6.84E-05	-0.98	0.15	5.03E-02	-1.39	0.25	2.37E-02	-0.66	0.10	3.48E-02	-1.16	0.04	9.09E-07
SGSM3	Yes	Yes	No	Down	0.47	1.83E-19	4.1E-05	-1.06	0.11	6.54E-03	-0.83	0.04	4.23E-04	-1.66	0.12	1.54E-03	-1.35	0.14	7.81E-03	-0.71	0.14	1.39E-01	-1.13	0.23	7.47E-02	-1.17	0.10	3.57E-03	-0.90	0.03	8.41E-04
SERINC5	Yes	Yes	No	Down	0.47	1.83E-19	0.00082	-1.11	0.11	1.74E-03	-0.89	0.04	1.34E-05	-1.55	0.12	1.22E-04	-1.26	0.13	4.21E-04	-0.75	0.14	1.75E-01	-0.79	0.22	3.10E-01	-1.16	0.10	3.86E-04	-1.32	0.04	1.00E-20
DNAJC10	Yes	Yes	No	Down	0.46	1.83E-19	0.00025	-0.95	0.11	2.31E-03	-0.90	0.04	2.02E-05	-1.27	0.12	1.06E-04	-1.50	0.14	2.96E-04	-1.30	0.16	1.27E-04	-0.71	0.21	2.55E-02	-1.27	0.10	2.25E-05	-1.02	0.04	1.14E-06
RNF183	Yes	Yes	No	Down	0.46	1.83E-19	1.5E-19	-0.98	0.11	4.40E-04	-1.19	0.04	1.00E-20	-1.16	0.11	1.27E-04	-1.68	0.15	4.54E-07	-1.20	0.16	2.41E-03	-1.29	0.24	1.09E-02	-0.72	0.10	4.24E-03	-0.79	0.03	9.09E-06
CYB561	Yes	Yes	No	Down	0.45	1.83E-19	0.00038	-1.59	0.12	1.96E-04	-1.00	0.04	1.20E-05	-1.31	0.12	1.16E-03	-1.86	0.16	3.43E-05	-1.36	0.16	3.26E-04	-0.68	0.21	5.90E-02	-0.40	0.10	1.59E-01	-0.95	0.03	8.34E-05
KIF13B	Yes	Yes	No	Down	0.45	1.83E-19	1.5E-19	-1.09	0.11	2.89E-03	-1.27	0.04	6.82E-07	-1.42	0.12	2.12E-04	-1.54	0.14	2.21E-04	-0.69	0.14	3.12E-02	-1.29	0.24	2.56E-03	-0.86	0.10	6.28E-03	-1.01	0.04	5.23E-05
ELP3	Yes	Yes	No	Down	0.45	1.83E-19	1.5E-19	-1.14	0.11	3.18E-04	-0.90	0.04	7.27E-06	-1.05	0.11	4.86E-04	-1.36	0.14	3.22E-04	-1.28	0.16	1.47E-04	-1.02	0.23	4.43E-03	-1.72	0.11	1.14E-06	-0.71	0.03	3.53E-04
PIGR	Yes	Yes	No	Down	0.45	1.83E-19	1.5E-19	-1.51	0.12	6.82E-07	-1.54	0.04	1.00E-20	-1.32	0.12	2.73E-06	-1.44	0.14	6.82E-07	-0.48	0.14	4.61E-02	-0.82	0.22	1.04E-02	-0.99	0.10	6.29E-05	-1.08	0.04	1.00E-20
THAP8	No	Yes	No	Up	2.15	2.64E-05	0.00022	1.25	0.12	8.85E-04	0.65	0.04	7.25E-03	0.68	0.11	1.10E-01	1.52	0.14	1.70E-03	0.99	0.15	3.35E-01	1.70	0.27	1.76E-01	1.01	0.10	4.76E-02	1.05	0.04	1.42E-03
TMPRSS2	Yes	Yes	No	Down	0.45	1.83E-19	7.7E-05	-0.91	0.11	1.11E-03	-1.10	0.04	4.54E-07	-0.95	0.11	1.58E-03	-1.75	0.15	7.27E-06	-1.37	0.16	6.11E-04	-1.14	0.23	8.44E-03	-1.20	0.10	5.32E-04	-0.79	0.03	2.04E-06
FBP1	Yes	Yes	Yes	Down	0.45	1.83E-19	1.5E-19	-1.27	0.12	3.86E-05	-1.27	0.04	1.00E-20	-1.22	0.11	3.92E-04	-0.92	0.12	2.24E-03	-1.26	0.16	4.32E-04	-1.53	0.26	5.52E-04	-1.23	0.10	2.46E-04	-0.63	0.03	1.26E-03
ST6GALNAC1	Yes	No	Yes	Down	0.45	1.83E-19	0.00444	-1.52	0.12	4.54E-07	-1.15	0.04	1.00E-20	-1.53	0.12	1.59E-06	-1.01	0.13	3.61E-04	-1.07	0.15	1.95E-03	-0.77	0.21	5.68E-02	-1.19	0.10	3.32E-05	-1.09	0.04	1.00E-20
RASSF6	Yes	Yes	No	Down	0.44	1.83E-19	1.5E-19	-1.11	0.11	1.44E-03	-0.87	0.04	2.26E-04	-1.66	0.12	1.36E-06	-1.44	0.14	2.73E-05	-1.06	0.15	1.54E-01	-1.19	0.24	1.19E-01	-0.76	0.10	4.14E-03	-1.28	0.04	1.00E-20
C11ORF52	Yes	Yes	No	Down	0.44	1.83E-19	1.5E-19	-1.28	0.12	2.51E-04	-1.02	0.04	2.04E-06	-1.08	0.11	8.92E-04	-0.91	0.12	3.37E-03	-1.16	0.16	1.06E-02	-1.23	0.24	9.06E-02	-1.60	0.11	9.77E-06	-1.11	0.04	6.82E-07
CITED4	Yes	Yes	No	Down	0.44	1.83E-19	1.5E-19	-1.63	0.13	2.04E-06	-0.96	0.04	7.72E-06	-0.67	0.11	3.07E-02	-1.86	0.16	4.32E-06	-1.14	0.16	4.11E-04	-1.54	0.26	1.67E-04	-0.54	0.10	1.84E-01	-1.07	0.04	1.00E-20
EYA2	Yes	Yes	No	Down	0.44	1.83E-19	1.5E-19	-1.63	0.13	4.54E-07	-1.15	0.04	1.00E-20	-1.10	0.11	1.09E-04	-0.87	0.12	1.86E-03	-1.39	0.17	4.84E-05	-0.85	0.22	1.02E-02	-1.93	0.12	1.00E-20	-0.56	0.03	4.26E-04
ENTPD3	Yes	Yes	No	Down	0.43	1.83E-19	1.5E-19	-1.35	0.12	1.70E-05	-1.30	0.04	1.00E-20	-0.71	0.11	5.57E-03	-0.74	0.12	5.03E-03	-1.18	0.16	2.18E-03	-1.92	0.29	2.29E-04	-1.31	0.11	6.59E-06	-1.12	0.04	1.00E-20
TMC5	Yes	Yes	Yes	Down	0.43	1.83E-19	1.5E-19	-1.87	0.13	4.54E-07	-1.29	0.04	1.00E-20	-0.69	0.11	4.93E-03	-2.20	0.18	1.00E-20	-0.67	0.14	1.39E-01	-0.99	0.22	8.58E-02	-1.10	0.10	3.77E-05	-0.87	0.03	1.00E-20
TJP3	Yes	Yes	No	Down	0.43	1.83E-19	0.00041	-1.05	0.11	1.68E-03	-1.30	0.04	1.00E-20	-1.48	0.12	9.16E-05	-1.60	0.15	4.84E-05	-0.92	0.15	3.30E-03	-1.38	0.25	9.19E-04	-0.90	0.10	3.72E-03	-1.06	0.04	1.00E-20
CRELD2	Yes	Yes	No	Down	0.43	1.83E-19	1.5E-19	-1.36	0.12	5.75E-04	-1.16	0.04	1.20E-05	-1.19	0.11	2.25E-03	-1.60	0.15	1.64E-03	-0.88	0.15	3.61E-03	-1.13	0.23	2.61E-03	-1.34	0.11	3.36E-04	-1.05	0.04	9.47E-05
TMCC2	No	Yes	No	Up	2.20	3.58E-06	2E-05	0.87	0.11	7.83E-03	1.05	0.04	2.98E-05	1.10	0.11	2.85E-02	1.40	0.14	2.09E-04	1.29	0.16	3.70E-01	1.78	0.28	2.04E-01	1.33	0.11	8.70E-03	0.29	0.03	7.88E-02
FBXO16	Yes	Yes	No	Down	0.43	1.83E-19	1.5E-19	-1.49	0.12	1.41E-04	-1.12	0.04	2.73E-06	-1.68	0.13	2.45E-05	-1.38	0.14	2.25E-04	-0.86	0.15	1.08E-01	-1.08	0.23	1.86E-01	-1.51	0.11	1.33E-04	-0.64	0.03	4.29E-03
SPA17	Yes	Yes	No	Down	0.43	1.83E-19	4E-05	-1.61	0.13	5.45E-06	-1.03	0.04	1.59E-06	-0.59	0.11	2.87E-02	-1.82	0.16	1.46E-04	-1.29	0.16	2.44E-04	-1.31	0.24	2.17E-03	-1.27	0.10	2.66E-05	-0.94	0.03	3.18E-06
NEURL2	No	No	No	Up	2.21	1.14E-05	1.5E-19	1.26	0.12	1.77E-03	0.82	0.04	1.78E-03	1.04	0.11	2.34E-02	0.84	0.12	1.29E-02	2.05	0.20	7.73E-02	1.82	0.28	9.52E-02	0.58	0.10	1.95E-01	0.73	0.03	5.29E-04
SLC47A1	Yes	Yes	Yes	Down	0.42	1.83E-19	1.5E-19	-1.89	0.13	1.00E-20	-1.31	0.04	1.00E-20	-0.68	0.11	4.70E-03	-1.12	0.13	1.51E-04	-1.16	0.16	2.91E-04	-1.33	0.24	5.02E-04	-1.13	0.10	1.02E-05	-1.28	0.04	1.00E-20
IL20RA	Yes	Yes	No	Down	0.42	1.83E-19	1.5E-19	-2.00	0.14	1.00E-20	-1.60	0.04	1.00E-20	-1.07	0.11	1.17E-04	-0.85	0.12	1.58E-03	-0.83	0.15	1.64E-02	-1.01	0.23	2.44E-02	-1.58	0.11	1.82E-06	-1.08	0.04	1.00E-20
DHRS7B	Yes	Yes	No	Down	0.41	1.83E-19	6.7E-06	-0.53	0.10	1.07E-01	-0.84	0.04	3.19E-04	-1.67	0.13	9.70E-05	-1.56	0.15	2.83E-03	-1.09	0.15	7.68E-03	-1.57	0.26	2.27E-03	-1.43	0.11	1.34E-04	-1.50	0.04	1.00E-20
GALNT4	Yes	Yes	No	Down	0.41	1.83E-19	1.5E-19	-1.37	0.12	2.79E-05	-1.22	0.04	1.00E-20	-1.65	0.12	1.59E-06	-1.31	0.14	7.75E-05	-1.30	0.16	2.56E-04	-0.89	0.22	1.26E-02	-1.31	0.11	1.66E-05	-1.16	0.04	1.00E-20
STAP2	Yes	Yes	No	Down	0.41	1.83E-19	1.5E-19	-1.05	0.11	8.29E-04	-1.23	0.04	4.54E-07	-1.20	0.11	1.70E-03	-1.28	0.13	5.24E-04	-0.97	0.15	1.78E-02	-1.07	0.23	1.68E-02	-2.42	0.13	2.27E-07	-1.11	0.04	1.14E-05
HGD	Yes	Yes	No	Down	0.40	1.83E-19	1.5E-19	-1.11	0.11	4.86E-05	-1.29	0.04	1.00E-20	-1.59	0.12	1.59E-06	-1.90	0.16	1.00E-20	-0.80	0.15	7.49E-03	-1.76	0.28	3.11E-04	-1.05	0.10	2.35E-04	-1.03	0.04	1.00E-20
SCGB2A1	Yes	Yes	Yes	Down	0.40	1.83E-19	1.5E-19	-1.32	0.12	2.50E-06	-1.29	0.04	1.00E-20	-1.32	0.12	4.32E-06	-1.14	0.13	3.20E-05	-1.75	0.18	1.59E-06	-1.49	0.26	1.13E-04	-1.03	0.10	5.91E-05	-1.25	0.04	1.00E-20
CAPS	Yes	Yes	No	Down	0.40	1.83E-19	1.5E-19	-1.10	0.11	6.52E-05	-1.64	0.04	1.00E-20	-1.11	0.11	9.45E-05	-1.35	0.14	6.20E-05	-1.15	0.16	2.62E-04	-1.42	0.25	3.36E-04	-1.61	0.11	4.54E-07	-1.25	0.04	1.00E-20
MGST2	Yes	Yes	No	Down	0.40	1.83E-19	0.00117	-1.55	0.12	1.87E-04	-1.16	0.04	2.27E-06	-2.11	0.14	3.68E-05	-1.04	0.13	3.41E-03	-1.19	0.16	3.03E-04	-1.88	0.29	1.79E-05	-0.56	0.10	6.69E-02	-1.22	0.04	3.41E-06
CEACAM1	Yes	Yes	No	Down	0.39	1.83E-19	1.5E-19	-1.33	0.12	3.11E-05	-1.58	0.04	1.00E-20	-1.90	0.13	1.00E-20	-0.79	0.12	3.67E-03	-1.19	0.16	4.71E-04	-1.42	0.25	8.89E-04	-1.42	0.11	2.73E-06	-1.16	0.04	1.00E-20
MARVELD2	Yes	No	No	Down	0.39	1.83E-19	validated	-1.48	0.12	1.93E-04	-1.21	0.04	6.82E-07	-0.78	0.11	1.19E-02	-1.76	0.15	1.66E-05	-1.67	0.18	2.67E-03	-1.51	0.26	8.77E-03	-1.10	0.10	1.19E-03	-1.28	0.04	1.00E-20
SAT1	Yes	Yes	No	Down	0.38	1.83E-19	1.5E-19	-1.11	0.11	3.47E-04	-1.50	0.04	1.00E-20	-1.57	0.12	2.79E-05	-0.60	0.12	4.88E-02	-1.74	0.18	2.27E-06	-1.42	0.25	2.09E-04	-2.01	0.12	1.00E-20	-1.37	0.04	1.00E-20
MLPH	Yes	Yes	No	Down	0.38	1.83E-19	1.5E-19	-1.74	0.13	4.54E-07	-1.57	0.04	1.00E-20	-0.87	0.11	2.10E-03	-2.03	0.17	1.00E-20	-1.11	0.16	4.44E-04	-1.34	0.25	5.94E-04	-1.45	0.11	1.82E-06	-1.19	0.04	1.00E-20
SPATA18	Yes	Yes	No	Down	0.37	1.83E-19	1.5E-19	-1.44	0.12	2.54E-05	-1.34	0.04	1.00E-20	-1.37	0.12	3.86E-06	-1.91	0.16	4.54E-07	-1.26	0.16	4.73E-04	-1.29	0.24	2.98E-03	-1.82	0.11	1.00E-20	-0.94	0.03	1.00E-20
TRPM4	Yes	Yes	Yes	Down	0.37	1.83E-19	1.5E-19	-1.82	0.13	4.54E-07	-1.13	0.04	4.54E-07	-1.83	0.13	3.18E-06	-1.94	0.16	7.72E-06	-0.81	0.15	5.45E-03	-1.36	0.25	4.02E-03	-1.19	0.10	4.43E-04	-1.43	0.04	1.00E-20
ASRGL1	Yes	Yes	No	Down	0.37	1.83E-19	1.1E-05	-1.72	0.13	4.54E-07	-1.08	0.04	1.00E-20	-1.35	0.12	1.82E-05	-1.52	0.14	1.52E-05	-1.58	0.17	3.63E-05	-1.55	0.26	3.77E-04	-1.54	0.11	2.04E-06	-1.17	0.04	1.00E-20
TFF3	Yes	Yes	Yes	Down	0.37	1.83E-19	1.5E-19	-2.03	0.14	1.00E-20	-1.80	0.05	1.00E-20	-1.80	0.13	1.00E-20	-1.12	0.13	5.34E-05	-0.88	0.15	1.91E-03	-1.33	0.24	3.46E-04	-1.49	0.11	4.54E-07	-1.10	0.04	1.00E-20
MYO5C	Yes	Yes	No	Down	0.37	1.83E-19	0.00014	-1.06	0.11	1.62E-03	-1.48	0.04	1.00E-20	-1.94	0.13	8.18E-06	-1.59	0.15	2.98E-05	-1.46	0.17	9.56E-05	-1.49	0.26	4.82E-04	-0.88	0.10	1.03E-02	-1.65	0.04	1.00E-20
ESR1	Yes	Yes	No	Down	0.36	1.83E-19	1.5E-19	-2.06	0.14	1.00E-20	-1.22	0.04	1.00E-20	-1.25	0.12	3.82E-05	-1.81	0.16	1.00E-20	-1.15	0.16	3.28E-04	-1.14	0.23	1.88E-03	-2.14	0.12	1.00E-20	-0.98	0.03	1.00E-20
ATP2C2	Yes	Yes	No	Down	0.36	1.83E-19	1.5E-19	-1.80	0.13	4.54E-07	-1.41	0.04	1.00E-20	-1.76	0.13	4.54E-07	-1.70	0.15	1.36E-06	-1.22	0.16	1.09E-02	-1.05	0.23	1.81E-02	-1.87	0.12	4.54E-07	-1.04	0.04	1.00E-20
PGR	Yes	Yes	Yes	Down	0.35	1.83E-19	1.5E-19	-1.92	0.14	1.00E-20	-1.54	0.04	1.00E-20	-0.93	0.11	2.44E-04	-1.58	0.15	4.54E-07	-1.38	0.17	5.23E-04	-1.85	0.29	2.20E-03	-1.62	0.11	1.00E-20	-1.23	0.04	1.00E-20
SPDEF	Yes	Yes	Yes	Down	0.34	1.83E-19	1.5E-19	-2.04	0.14	1.00E-20	-2.14	0.05	1.00E-20	-1.38	0.12	2.32E-05	-1.47	0.14	1.61E-05	-1.18	0.16	4.71E-04	-1.18	0.23	2.29E-03	-1.51	0.11	6.59E-06	-1.48	0.04	1.00E-20
PLPP2	Yes	Yes	No	Down	0.33	1.83E-19	1.5E-19	-2.57	0.16	1.00E-20	-1.64	0.04	1.00E-20	-1.00	0.11	6.52E-04	-1.57	0.15	1.93E-05	-1.48	0.17	3.92E-04	-1.27	0.24	3.34E-03	-1.80	0.11	2.27E-07	-1.52	0.04	1.00E-20
KIAA1324	Yes	Yes	Yes	Down	0.30	1.83E-19	1.5E-19	-3.23	0.20	1.00E-20	-1.84	0.05	1.00E-20	-1.40	0.12	2.27E-06	-1.37	0.14	1.59E-06	-1.45	0.17	2.61E-05	-1.50	0.26	1.49E-04	-1.68	0.11	1.00E-20	-1.56	0.04	1.00E-20
FOXA2	Yes	Yes	No	Down	0.29	1.83E-19	1.5E-19	-2.84	0.18	1.00E-20	-1.52	0.04	1.00E-20	-1.45	0.12	3.41E-06	-2.41	0.19	1.00E-20	-1.54	0.17	1.45E-05	-2.08	0.31	2.73E-06	-1.25	0.10	1.36E-05	-1.25	0.04	1.00E-20

Supplementary Table 4. Gene expression microarray studies for endometrial cancer available for analysis.
EC - endometrial cancer

Reference	PMID	Samples	Data retrieval	Comment
Mutter et al 2001 Gynecol Oncol 83:177-185	11606070	10 EC; 4 Normal	Via collaboration	Excluded - no EEC/NEEC information
Moreno-Bueno et al 2003 Cancer Research 63:5697-5702	14522886	35 EC	Via collaboration	
Risinger et al 2003 Cancer Research 63:6-11	12517768	35 EC; 7 Normal	Downloaded from author website: http://home.ccr.cancer.gov/risingerdata1102	
Saidi et al 2004 Oncogene 23:6677-6683	15247901	20 EC; 11 Normal	Provided by authors as supplementary data to publication	Excluded - unable to update probe annotations
Shedden et al 2005 Clin Cancer Res 11:2123-2131	15788657	18 EC	Via collaboration	
Zorn et al 2005 Clin Cancer Res 11(18):6422-6430	16166416	35 EC; 7 Normal	Via collaboration	
Huvila et al 2009 Int J Gynecol Oncol	19823059	13 EC	Via collaboration	Excluded - no EEC/NEEC information
Salvesen et al 2009 PNAS 106(12):4834-9	19261849	57 EC	ArrayExpress accession: E-GEOD-14860	Excluded - samples overlapped with other studies
Levan et al 2010 Gene Expr 14(6):361-70	20635577	45 EC	ArrayExpress accession: E-GEOD-21882	Excluded - no EEC/NEEC information
Mhawch-Fauceglia et al 2010 PLoS One 5(11):e15415	21079744	20 EC	ArrayExpress accession: E-GEOD-23518	
Day et al 2011 BMC Bioinformatics 12:213	21619611	91 EC; 12 Normal	ArrayExpress accession: E-GEOD-17025	
Mhawch-Fauceglia et al 2011 PLoS One 6(3):e18066	21448288	33 EC	GEO accession: GSE24537	
Krakstad et al 2012 PLoS One 7(12):e52795	23300780	69 EC	ArrayExpress accession: E-MTAB-1358	Excluded - samples overlapped with other studies
Mannelqvist et al 2012 BMC Cancer 12:169	22559235	76 EC	ArrayExpress accession: E-MTAB-1007	Excluded - samples overlapped with other studies
Chiyoda et al 2012 Genes Chromosomes Cancer 51(3):229-39	22072501	38 EC; 8 sarcoma	GEO accession: GSE32507	
Tangen et al 2014 PLoS One 9(5):e98069	24849812	198 EC	ArrayExpress accession: E-MTAB-2532	
Kharma et al 2014 Cancer Res 74(22):6519-30	25267067	63 EC	ArrayExpress accession: E-GEOD-56026	
expO	NA	75 EC	ArrayExpress accession: E-GEOD-2109	
Pappa et al 2015 PLoS One 10(11): e0142229	26559525	7 EC; 5 Normal	ArrayExpress accession: E-GEOD-63678	Excluded - no EEC/NEEC information
E-GEOD-36389	unpublished	13 EC; 7 Normal	ArrayExpress accession: E-GEOD-36389	Excluded - no EEC/NEEC information
TCGA	NA	54 EC	Downloaded from TCGA website: https://tcga-data.nci.nih.gov/tcga/	
